# Exploring associations of three evaluative subjective wellbeing measures (Cantril’s ladder, life satisfaction, happiness) with 15 childhood and demographic factors across 22 countries

**DOI:** 10.1038/s41598-026-35777-y

**Published:** 2026-02-10

**Authors:** Tim Lomas, Hayami K. Koga, R. Noah Padgett, James O. Pawelski, Eric S. Kim, Christos A. Makridis, Craig Gundersen, Matt Bradshaw, Noémie Le Pertel, Koichiro Shiba, Chris Felton, John F. Helliwell, Byron R. Johnson, Tyler J. VanderWeele

**Affiliations:** 1 Department of Epidemiology, Harvard T.H. Chan School of Public Health, Boston, MA USA; 2 Human Flourishing Program, Harvard University, Cambridge, MA USA; 3 Department of Biostatistics, Harvard T.H. Chan School of Public Health, Boston, MA USA; 4 Positive Psychology Center, University of Pennsylvania, Philadelphia, PA USA; 5 Department of Psychology, The University of British Columbia, Vancouver, BC Canada; 6 W. P. Carey School of Business, Arizona State University, Tempe, AZ USA; 7 Institute for the Future, University of Nicosia, Nicosia, Cyprus; 8 Department of Economics, Baylor University, Waco, TX USA; 9 Institute for Studies of Religion, Baylor University, Waco, TX USA; 10 Department of Epidemiology, Boston University, Boston, MA USA

**Keywords:** Life evaluation, Life satisfaction, Happiness, Subjective wellbeing, Cross-cultural, Psychology, Health care

## Abstract

Despite a vast literature on subjective wellbeing (SWB), issues remain, including (a) debates around which concepts best represent it, (b) a disjointed understanding of relevant factors, and (c) limited appreciation of cross-national variation regarding (a) and (b). We address these points using data from the Global Flourishing Study on three constructs pertaining to *evaluative* SWB specifically (Cantril’s ladder, life satisfaction, and, perhaps more ambiguously, happiness), examining associations with 15 childhood and demographic factors across 202,898 participants from 22 countries. Key findings include, for (a) life satisfaction being the best performing construct (in correlations with overall flourishing), (b) all factors being significantly associated with all constructs (with the largest variation observed for employment status among demographic factors and self-reported health among childhood factors), and (c) patterns varying substantively across countries (suggesting the general trends are not universal but differ according to local socio-cultural dynamics). The findings advance the methodological, socio-demographic, and cross-national understanding of evaluative SWB.

**Supplementary Information:**

The online version contains supplementary material available at 10.1038/s41598-026-35777-y.

## Introduction

Over recent decades prominent intergovernmental organisations (e.g., UN, WHO, OECD) have advocated for including subjective wellbeing (SWB) indicators alongside traditional economic metrics like GDP when assessing societies and making policy^1–5^. SWB scholarship has three interrelated issues however which limit our understanding and application of this topic. First, there is still uncertainty about how SWB should best be assessed, including regarding its evaluative dimension (E-SWB), with the three most prominent candidates being Cantril’s “ladder”^6^(CL), life satisfaction (LS), and, perhaps more ambiguously, happiness (H). Second, there is a relatively disjointed understanding of factors associated with E-SWB: while one can find analyses of almost every conceivable factor, few studies have a comprehensive array that would allow meaningful comparison among them^7^. Third, while E-SWB has been extensively assessed internationally, there remains a comparatively impoverished understanding of granular national-level variation, especially in relation to the first two issues. Many studies address one or two of these points, but rarely all three together, hence the value of this paper, which examines Wave 1 data on CL, LS, and H in the Global Flourishing Study (GFS), an intended five-year panel study investigating the predictors of flourishing involving 207,919 participants (in Wave 1) from 22 countries (although data from mainland China were not available when the present analyses were conducted, and so here we only include results from 202,898 participants). Before reviewing the three aims of the paper—namely, to address these three issues—we briefly contextualize the research by situating E-SWB within the broader concept of flourishing, though our focus in this paper will mainly be on E-SWB itself.

### Flourishing and SWB

Over recent years “flourishing” has emerged as an overarching construct encompassing the various aspects/forms of wellbeing across different dimensions of life. VanderWeele for example^8^, co-PI of the GFS, defines it as “the relative attainment of a state in which all aspects of a person’s life are good, including the contexts in which that person lives,” and identifies five key domains: happiness and life satisfaction; mental and physical health; meaning and purpose; character and virtue; and close social relationships. To these, a sixth domain, financial and material security, is added as a key means for “secure flourishing” over time. While not exhaustive of flourishing, VanderWeele argues all six are “arguably at least a part of what we mean by flourishing.” Of particular interest here is the first domain, which closely relates to certain understandings of SWB. Regarding SWB itself, there are various definitions in the literature, some broader, some narrower^9^. Arguably the most influential conceptualization is that of Diener and colleagues, which comprises two dimensions: cognitive/evaluative (e.g., LS), and affective (positive and negative affect)^10,11^. Given the myriad conceptualizations of both flourishing and SWB, their relationship inevitably depends on the definitions used. In VanderWeele’s framework, LS and H constitute one domain of flourishing. But in some definitions, SWB—and especially E-SWB—can reflect/encompass subjective assessments of all dimensions of life. Consider the widely used definition of LS by Shin and Johnson (who call it “avowed happiness”) as “a global assessment of a person’s quality of life according to his chosen criteria,”^12^ which potentially offers a summary of flourishing overall. It is beyond our scope to resolve the broader question of the relationship between SWB and flourishing under all definitions of these terms. Our main focus is SWB itself, and specifically E-SWB. Despite a vast literature on E-SWB, the scholarship has three main issues, and although many studies tackle one or two, by addressing all three together, our paper aims to shed new light on them.

### Aim 1: comparing E-SWB items

Although the construct of SWB is well-established, definitions vary. While Diener and colleagues identified two main dimensions as noted above, other conceptualizations can be broader. The OECD for example also include eudaimonia, at least in some operationalizations. The stated definition in their updated 2025 guidelines^13^  emphasizes the two dimensions associated with Diener, presenting SWB as “Good mental states, including all of the various evaluations, positive and negative, that people make of their lives and the affective reactions of people to their experiences” (p.12). However, their executive summary also includes eudaimonia in its description, stating that “Each component of subjective well-being – life evaluation (reflective assessments, such as satisfaction with life), affect (feelings or emotional states) and eudaimonia (a sense of worth, meaning and purpose) – captures a distinct and meaningful facet of people’s subjective life experiences” (p.8). Whatever the formulation though, conceptualizations of SWB invariably include an evaluative component (E-SWB), often referred to generically as life evaluation (LE), and here we use E-SWB and LE interchangeably. There is ongoing debate about how E-SWB should best be *assessed* however, with three main candidates being Cantril’s “ladder”^6^ (CL), life satisfaction (LS), and happiness (H). As with SWB and E-SWB, each has been defined in myriad ways, though it is beyond our scope to review these varied uses, so Table 1 simply presents our preferred definitions to show how we are interpreting these concepts. We would especially highlight the fact that our definition of *E*-SWB is adapted from the OECD definition of SWB as a whole, which contains both an evaluative component (“… including all of the various evaluations, positive and negative, that people make of their lives …”), and an affective component (“… and the affective reactions of people to their experiences.”). For our definition of E-SWB, we therefore omit the latter and just retain the former.

**Table 1:** Definitions and assessments of key concepts.

Concept	Preferred definition	Item in GFS
Flourishing	“The relative attainment of a state in which all aspects of a person’s life are good, including the contexts in which that person lives” (VanderWeele^8^)	Captured together by 12 items as the “Secure Flourish Index”
Subjective wellbeing (SWB)	“Good mental states, including all of the various evaluations, positive and negative, that people make of their lives and the affective reactions of people to their experiences” (OECD^13^)	Numerous
Evaluative subjective wellbeing (E-SWB)	“All of the various evaluations, positive and negative, that people make of their lives” (adapted from OECD^13^)	CL, LS, and H
Life evaluation	Same as E-SWB	CL, LS, and H
Cantril’s ladder	See item	“Please imagine a ladder with steps numbered from zero at the bottom to ten at the top. The top of the ladder represents the best possible life for you, and the bottom of the ladder represents the worst possible life for you. On which step of the ladder would you say you personally feel you stand at this time?” [0 = Worst possible, to 10 = Best possible]
Life satisfaction	“A global assessment of a person’s quality of life according to his chosen criteria” (Shin & Johnson^12^, which is used as the definition in the Satisfaction with Life Scale^14^)	“Overall, how satisfied are you with life as a whole these days?” [from 0 = Not satisfied with your life at all, to 10 = Completely satisfied with your life]
Happiness	Depends on framing	“In general, how happy or unhappy do you usually feel?” [from 0 = Extremely unhappy to 10 = Extremely happy]

We should note that the reason we haven’t provided a simple definition of H is that it is a particularly ambiguous and contested concept^15^, and its meaning fundamentally depends on the framing. Diener for instance reviewed definitions of H and grouped these into three types: normative (i.e., invoking “external criteria such as virtue”); evaluative (e.g., Shin and Johnson’s operationalization, cited above); and affective (i.e., involving “pleasant emotional experience”)^16^. Given this conceptual breadth, in a 2009 paper with Oishi and Lucas, Diener wrote “we use the term happiness interchangeably with subjective wellbeing or the subjective evaluation of one’s life.”^17^ However, they also state that they personally prefer to interpret/use H above all as an *evaluative* concept: while they “conceptualize happiness as being hierarchically organized to emphasize complexity of the concept,” the “highest level of the abstraction is … a summary judgment of one’s life. That is, we do not use the term happiness to refer to the momentary feeling state of happiness. Rather, we use this term to refer to a relatively stable feeling of happiness one has towards his or her life” (p.347). Similarly, the World Happiness Report (WHR)^18^ interprets H as an evaluative concept. As Helliwell explains^19^, the WHR “justifies its title by arguing that there are two quite different uses of the word happiness. One is to describe the emotion of happiness, as in questions about happiness yesterday. The second use is judgmental, asking whether one is happy about something.” Drawing on linguistic philosophy^20^, he argues that “any potential ambiguity is resolved by the logic of the conversation in which the word is used,” with survey research showing that answers to “the emotion question depend on what was going on the day before, while happiness with life reflects a longer-term judgment more based on the overall pattern of life”^21^. We would suggest that the wording in the GFS, shown in the Table above, means the item falls into this second category. As such, in addition to CL and LS, H—especially in the way it is framed in the GFS—also constitutes a candidate for assessing E-SWB.

Most relevantly here though, there remains debate around *which* concept offers the best single assessment of E-SWB: the OECD recommend LS, for instance, while the WHR^18^ harnesses the CL item in the Gallup World Poll (GWP). It may well be of course that these concepts all capture subtly different aspects of E-SWB, and so the question of preference depends on what one seeks to measure. Moreover, in a practical sense, the solution to arriving at the best assessment of E-SWB could simply be to include multiple items. In a 2010 chapter, for example, Helliwell and colleagues used the first three waves of the GWP to compare CL and LS items to ascertain “what constitutes a good life,” and found that “an average of the two measures” provides “a clearer picture than either measure on its own.”^22^ Even though the items are actually “related in very similar ways to the underlying structural determinants” of E-SWB, and both constitute good “evaluations of life as a whole,” since they are framed differently and asked at different points in the GWP, their average has “a higher signal-to-noise ratio than either on its own.” Helliwell has also argued that H too functions similarly—at least when framed as a “happy with life” question, such as in the European Social Survey—and that it is possible to “triangulate the evidence to show the rough comparability of all three questions, when assessed by their consistency in estimating what supports better lives.”^19^ However, given the space constraints in many surveys, assessments tend to pick just one. In that case, it is currently unclear *which* might be the best to choose—especially as there has been relatively little research directly comparing the items, given that many surveys *do* just include one. While the GFS cannot definitively answer this question—since it depends on what one seeks to measure, as noted above—it can at least shed some light on it by directly comparing the items, including in terms of their association with flourishing.

### Aim 2: exploring the relative contribution of factors

A second aim of the paper is to offer a comprehensive assessment of the relative strength of myriad factors associated with E-SWB. This is helpful vis-a-vis the first issue (differentiating E-SWB items), but is also valuable in its own right. There is a vast literature on factors linked to E-SWB, including: systemic factors such as national economics^23^, governance quality^24^, political/economic freedom^25^, community infrastructure^26^, and social capital^27^; demographic factors like income^28^, age^29^, sex^30^, sexuality^31^, and race/ethnicity^32^; and childhood factors such as Adverse Childhood Experiences^33,34^. One issue however with many studies is a relatively limited coverage of factors, with most publications tending to focus on just a handful, and often on just one key factor (together with relevant confounders). An example of the latter is an analysis of age (SWB “across the lifespan”) that controls for five socio-demographic characteristics (gender, employment, marital status, education, and health)^35^. The literature overall is consequently somewhat disjointed. There are analyses of almost any conceivable factor, from childhood health^36^ and socioeconomic status^37^ to adult employment^38^ and immigration status^39^, so many “puzzle pieces” (i.e., analyses of factors) are on the table. But with most papers only including a few “pieces,” it is hard to gauge the relative strength of their associations and hence better ascertain the overall picture (per the metaphor above of “puzzle pieces”), which requires comparing factors in the same analysis with the same population. As such, we seek to harness the fact that the GFS includes data on 15 factors selected as potentially relevant to all aspects of flourishing—analysed across a series of papers in relation to all flourishing outcomes in the GFS questionnaire^40^—to gain a fuller appreciation of the relative contribution of a wide range of factors to E-SWB.

### Aim 3: examining granular cross-national differences

A third aim is to conduct a granular exploration of national variation, both for the three outcomes and the 15 factors. There is already an extensive literature examining international variation on E-SWB, harnessing datasets like the World Values Survey, European Social Survey, and the GWP. A search on Google Scholar in November 2025 for the combined phrases “Gallup World Poll” and “subjective well-being,” for example, returned 6,140 results. Thus, although the bias towards relatively “WEIRD” populations highlighted by Henrich and colleagues^41^ in 2010 arguably still remains an issue^42^ (albeit one that is gradually improving^43,44^), research into E-SWB may be among the notable exceptions. That the GFS covers 22 countries in itself is therefore unremarkable. However, its analytic approach *is* quite unusual, in that it effectively constitutes 22 separate country-specific studies, the results of which are meta-analysed to identify overall patterns. Consequently, we can access and provide detailed data for each country, facilitating granular understanding of cross-national variation. Moreover, the combination of all three contributions makes our study particularly valuable, with each contribution magnifying the impact of the others. Few studies with granular international coverage for example also assess three E-SWB concepts and/or 15 factors, and vice versa. This means we are not only able to compare the three concepts (aim 1) and 15 factors (aim 2), but also explore national variation across both of these, which enriches our understanding in relation to both aims (e.g., our knowledge regarding any particular factor is enhanced by an appreciation of cross-cultural variation in its association with E-SWB).

## Methods

This Methods section has been adapted from VanderWeele and colleagues^45^, with further detail available elsewhere^40,46–53^. The methods of the current article are intended to align with these works to provide methodological consistency for comparability of results across papers. The GFS involves a 109-item questionnaire^40^ involving: (a) questions covering the six domains of VanderWeele’s flourishing framework^8^, which in addition to items pertaining to E-SWB include ones on health, meaning, character, relationships, and financial/material security; and (b) other demographic, social, economic, political, religious, personality, childhood, community, health, and wellbeing variables. Among the (b) items, questions pertaining to 15 childhood and demographic factors were selected to be analysed across an extensive series of papers, each focused on different flourishing outcomes in the GFS. The present paper concentrates on three E-SWB outcomes specifically, reporting on demographic^54^ and childhood^55^ factors associated with CL, LS, and H in Wave 1, allowing for comparison of results reported elsewhere (see VanderWeele and colleagues^56^ for an example of comparing across papers).

### Data

The GFS is a study of 207,919 participants (in Wave 1) from 22 geographically and culturally diverse countries, with nationally representative sampling within each country, including: Argentina, Australia, Brazil, China—including separately both Hong Kong (S.A.R. of China) and the mainland, meaning the GFS includes *23 *distinct populations—Egypt, Germany, India, Indonesia, Israel, Japan, Kenya, Mexico, Nigeria, Philippines, Poland, South Africa, Spain, Sweden, Tanzania, Turkey, UK, and US. The countries were selected to: (a) maximize coverage of the world’s population, (b) ensure geographic, cultural, and religious diversity, and (c) prioritize feasibility and existing data collection infrastructure. Data collection was carried out by Gallup. Data for Wave 1 were collected principally during 2023, with some countries beginning data collection in 2022, and exact dates varying by country^52^, as detailed in Supplementary Table S25a. However, Wave 1 data for mainland China were collected in March/April 2024 (Ritter et al., 2025), and were not available when the present analyses were conducted, meaning that the current paper does not include mainland China and features only 202,898 participants (rather than the full Wave 1 complement of 207,919). At least four additional waves of panel data on these participants are being collected annually from 2024 to 2027. The precise sampling design to ensure nationally representative samples varied by country and further details are available (https://osf.io/k2s7u)^52^. The data are publicly available through the Center for Open Science (https://www.cos.io/gfs-access-data).

### Measures

#### Outcome variables

The GFS includes three separate items pertaining to E-SWB: (1) CL – “Please imagine a ladder with steps numbered from zero at the bottom to ten at the top. The top of the ladder represents the best possible life for you, and the bottom of the ladder represents the worst possible life for you. On which step of the ladder would you say you personally feel you stand at this time?” [0 = Worst possible, to 10 = Best possible]; (2) LS – “Overall, how satisfied are you with life as a whole these days?” [from 0 = Not satisfied with your life at all, to 10 = Completely satisfied with your life]; and (3) H – “In general, how happy or unhappy do you usually feel?” [from 0 = Extremely unhappy to 10 = Extremely happy]. Our report investigates mean and variability differences in these outcomes across demographic and childhood factors. Wave 1 of the GFS involves participants first completing an intake questionnaire featuring 43 items (mainly gathering demographic information), followed by a second annual questionnaire (i.e., to be completed every year), involving a further 66 items (covering all different aspects of flourishing). The three outcomes in the present paper are the first (CL), third (H), and fourth (LS) items of the annual questionnaire.

#### Variables for demographic variation analyses

The demographic factors are standard variables one can find in most empirical surveys of this nature. Continuous age was classified as 18–24, 25–29, 30–39, 40–49, 50–59, 60–69, 70–79, and 80 or older. Gender was assessed as male, female, or other. Marital status was assessed as single/never married, married, separated, divorced, widowed, and domestic partner. Employment was assessed as employed, self-employed, retired, student, homemaker, unemployed and searching for a job, and other. Education was assessed as up to 8 years, 9–15 years, and 16+ years. Religious service attendance was assessed as more than once/week, once/week, one-to-three times/month, a few times/year, or never. Immigration status was dichotomously assessed with: “Were you born in this country, or not?” Religious tradition/affiliation was assessed with categories of Christianity, Islam, Hinduism, Buddhism, Judaism, Sikhism, Baha’i, Jainism, Shinto, Taoism, Confucianism, Primal/Animist/Folk religion, Spiritism, African-Derived, some other religion, or no religion/atheist/agnostic; precise response categories varied by country^53^. Racial/ethnic identity was assessed in some, but not all, countries, with response categories varying by country. For additional details on the assessments see the COS GFS codebook^57^ or Crabtree et al.^46^

#### Variables for childhood analyses

The childhood questions were selected based on prior literature concerning longitudinal associations of childhood factors with subsequent health and wellbeing. These factors include variables which past literature has indicated have beneficial associations with subsequent wellbeing (e.g., good relationship with parents, religious service attendance, financial security), along with questions that cover the two major domains of adverse childhood experiences: threat (assessed by an item on abuse) and neglect (assessed by an item asking about feeling like an outsider in one’s family). Relationship with mother during childhood was assessed with the question: “Please think about your relationship with your mother when you were growing up. In general, would you say that relationship was very good, somewhat good, somewhat bad, or very bad?” Responses were dichotomized to very/somewhat good versus very/somewhat bad. An analogous variable was used for relationship with father. “Does not apply” was treated as a dichotomous control variable for respondents who did not have a mother or father due to death or absence. Parental marital status during childhood was assessed with responses of married, divorced, never married, and one or both had died. Financial status was measured with: “Which one of these phrases comes closest to your own feelings about your family’s household income when you were growing up, such as when you were around 12 years old?” Responses were lived comfortably, got by, found it difficult, and found it very difficult. Abuse was assessed with yes/no responses to “Were you ever physically or sexually abused when you were growing up?” Participants were separately asked: “When you were growing up, did you feel like an outsider in your family?” Childhood health was assessed by: “In general, how was your health when you were growing up? Was it excellent, very good, good, fair, or poor?” Immigration status was assessed with: “Were you born in this country, or not?” Religious service attendance during childhood was assessed with: “How often did you attend religious services or worship at a temple, mosque, shrine, church, or other religious building when you were around 12 years old?” with responses of at least once/week, one-to-three times/month, less than once/month, or never. Childhood religious tradition/affiliation had response categories of Christianity, Islam, Hinduism, Buddhism, Judaism, Sikhism, Baha’i, Jainism, Shinto, Taoism, Confucianism, Primal/Animist/Folk religion, Spiritism, African-Derived, some other religion, or no religion/atheist/agnostic; response categories varied by country^53^. When the category no religion/atheist/agnostic had more than 5% of the within country sample size, this was used as the reference category; otherwise, the most prominent religious group was used. Additionally, all religious categories endorsed by less than 3% of the within country sample size were collapsed into a single religious category. For inclusion in the childhood regression analyses, race/ethnic identity was collapsed as a binary variable of whether an individual was in the most prominent group versus a minority group (race plurality).

### Statistical analysis

Descriptive statistics for the full sample, weighted to be nationally representative within each country, were estimated for each of the demographic and childhood experience variables. Nationally representative means for CL, LS, and H were estimated separately for each country and ordered from highest to lowest, along with 95% confidence intervals, standard deviations, and Gini coefficients. Variation in means in CL, LS, and H scores across categories of demographic variables (see *Variables for Demographic Variation Analyses *section) were estimated^46^. A weighted linear regression model with complex survey adjusted standard errors was fit by regressing each outcome on all the aforementioned childhood variables (see *Variables for Childhood Analyses *section) simultaneously^50^. All analyses were initially conducted by country (see Supplementary Tables). Primary results pooled across country-specific estimates using random effects meta-analyses^58,59^ along with 95% confidence intervals, standard errors, upper and lower limits of a prediction interval across countries, estimate proportions of effects across countries with effect sizes larger than 0.1 and smaller than −0.1, heterogeneity (τ), and I^2^where appropriate for a given outcome/analysis, for evidence concerning variation within a particular estimate across countries^60^. Discussion of the rationale underpinning the choice of a meta-analytic approach (over multilevel modelling) can be found in Padgett et al.^61,62^ Forest plots of estimates are available in the Supplementary files. All meta-analyses were conducted in R^63^ using the metafor package^56^. Within each country, a global test of variation (or association) of outcomes across levels of each particular demographic variable was conducted, and a pooled p-value^64^ across countries reported concerning evidence for variation within any country. Bonferroni corrected p-value thresholds are provided based on the number of demographic or childhood variables^65,66^. For each childhood factor, we calculated E-values to evaluate the sensitivity of results to unmeasured confounding. An E-value is the minimum strength of the association an unmeasured confounder must have with both the outcome and the factor, above and beyond all measured covariates, for an unmeasured confounder to explain away an association^67^. Religious affiliation and racial/ethnic identity were not included in the meta-analyses because these variables were not measured consistently across all 22 countries. As a supplementary analysis, population weighted meta-analyses were also conducted. All analyses were pre-registered with Center for Open Science prior to data access, with separate registrations by construct (albeit with CL and LS combined) and focal analysis: CL and LS (childhood: https://osf.io/8nvw6; demographic: https://osf.io/b8z59); and H (childhood: https://osf.io/shnp6; demographics: https://osf.io/46vr3). An unplanned analysis to estimate the intraclass correlation based on the results from the ordered means analysis was also conducted. The ICC was approximated from the results presented in Table 3 by estimating (a) the sample variance in the means and (b) the average within country variance, then the ICC was approximated by ICC = a/(a + b). The ICC is interpreted as the proportion of the variable in an outcome that is explained by mean differences on that outcome on a grouping variable

#### Missing data and multiple imputation

All missing variables were imputed using multivariate imputation by chained equations, with five imputed datasets generated^68,55^. The imputation model incorporated the criterion/outcome variable, all demographic or childhood experience characteristics, including race/ethnicity and religious affiliation when available, and sampling weights. The sampling weights were included as a variable in the imputation models to allow for specific variable missingness to be related to probability of study inclusion. The Gallup-provided sampling weights incorporate nonresponse and poststratification adjustments which helps to account for missingness being related to nonresponse of specific subgroups. To account for variations in the assessment of certain variables across countries (e.g., race/ethnicity and religious affiliation), we conducted the imputation process separately for each country. The within-country imputation approach ensured that the imputation model accurately reflects country-specific contexts and assessment methods. The percent of missing data for all variables is reported in our Supplementary Tables by country.

#### Supplemental post-hoc analyses

Complete-case analyses were conducted to replicate all primary analyses (country-specific and meta-analytic); these are reported in our Online Supplement, first in terms of providing versions of the main tables based on complete case analysis (Tables S1a-g), and then for each country individually (Tables e-g for each country). The meta-analytic pooled correlations among CL, LS, and H, and of these variables with indicators of flourishing, were computed. The means of each outcome were additionally computed by world region (WEIRD, non-WEIRD, and by continent).

#### Accounting for complex sampling design

The GFS used different sampling schemes across countries based on availability of existing panels and recruitment needs^52^. All analyses accounted for the complex survey design components by including weights, primary sampling units, and strata. Additional methodological detail, including accounting for the complex sampling design, is provided elsewhere^48^.

## Results

### Descriptive statistics

Table 2 provides the distribution of descriptive statistics (weighted counts and proportions). The GFS assessed 15 socio-demographic factors: four demographic, eight childhood, and three pertaining to both (age/birth cohort, gender, and immigration status, analysed/presented below both as demographic and childhood factors). Most participants were: married (52%), of 9–15 years of education (57%), born in their country of residence (94%), and employed (39%). Counts and proportions for demographic characteristics weighted to be representative of each country’s population are reported in Supplemental Tables S2a-S23a.

**Table 2:** Demographic characteristics of the GFS sample (Wave 1).

Characteristic		*N* = 202,898
Demographic characteristics		
Age group		
18–24	27,007 (13%)	
25–29	20,700 (10%)	
30–39	40,256 (20%)	
40–49	34,464 (17%)	
50–59	31,793 (16%)	
60–69	27,763 (14%)	
70–79	16,776 (8.3%)	
80 or older	4,119 (2.0%)	
(Missing)	20 (< 0.1%)	
Gender		
Male	98,411 (49%)	
Female	103,488 (51%)	
Other	602 (0.3%)	
(Missing)	397 (0.2%)	
Current Marital status		
Married	107,354 (53%)	
Separated	5,195 (2.6%)	
Divorced	11,654 (5.7%)	
Widowed	9,823 (4.8%)	
Single, never married	52,115 (26%)	
Domestic Partner	14,931 (7.4%)	
(Missing)	1,826 (0.9%)	
Employment status		
Employed for an employer	78,815 (39%)	
Self-employed	36,362 (18%)	
Retired	29,303 (14%)	
Student	10,726 (5.3%)	
Homemaker	21,677 (11%)	
Unemployed and looking for a job	16,790 (8.3%)	
None of these/Other	8,431 (4.2%)	
(Missing)	793 (0.4%)	
Current Religious service attendance		
More than 1/week	26,537 (13%)	
1/week	39,157 (19%)	
1–3/month	19,749 (9.7%)	
A few times a year	41,436 (20%)	
Never	75,297 (37%)	
(Missing)	722 (0.4%)	
Education		
Up to 8 years	45,078 (22%)	
9–15 years	115,097 (57%)	
16 + years	42,578 (21%)	
(Missing)	146 (< 0.1%)	
Immigration status		
Born in this country	190,998 (94%)	
Born in another country	9,791 (4.8%)	
(Missing)	2,110 (1.0%)	
Childhood experiences and characteristics		
Relationship with mother growing up		
Very good	127,836 (63%)	
Somewhat good	52,439 (26%)	
Somewhat bad	11,060 (5.5%)	
Very bad	4,642 (2.3%)	
Does not apply	5,965 (2.9%)	
(Missing)	956 (0.5%)	
Relationship with father growing up		
Very good	107,742 (53%)	
Somewhat good	55,714 (27%)	
Somewhat bad	15,807 (7.8%)	
Very bad	8,278 (4.1%)	
Does not apply	13,985 (6.9%)	
(Missing)	1,372 (0.7%)	
Parent marital status at age 12		
Parents married	152,001 (75%)	
Divorced	17,726 (8.7%)	
Parents were never married	15,534 (7.7%)	
One or both parents had died	7,794 (3.8%)	
(Missing)	9,843 (4.9%)	
Subjective financial status of family growing up		
Lived comfortably	70,861 (35%)	
Got by	82,905 (41%)	
Found it difficult	35,852 (18%)	
Found it very difficult	12,606 (6.2%)	
(Missing)	674 (0.3%)	
Abuse		
Yes	29,139 (14%)	
No	167,279 (82%)	
(Missing)	6,479 (3.2%)	
Outsider growing up		
Yes	28,732 (14%)	
No	170,577 (84%)	
(Missing)	3,589 (1.8%)	
Self-rated health growing up		
Excellent	67,121 (33%)	
Very good	63,086 (31%)	
Good	47,378 (23%)	
Fair	19,877 (9.8%)	
Poor	4,906 (2.4%)	
(Missing)	530 (0.3%)	
Age 12 religious service attendance		
At least 1/week	83,237 (41%)	
1–3/month	33,308 (16%)	
< 1/month	36,928 (18%)	
Never	47,445 (23%)	
(Missing)	1,980 (1.0%)	
Country		
Argentina	6,724 (3.3%)	
Australia	3,844 (1.9%)	
Brazil	13,204 (6.5%)	
Egypt	4,729 (2.3%)	
Germany	9,506 (4.7%)	
Hong Kong (S.A.R. of China)	3,012 (1.5%)	
India	12,765 (6.3%)	
Indonesia	6,992 (3.4%)	
Israel	3,669 (1.8%)	
Japan	20,543 (10%)	
Kenya	11,389 (5.6%)	
Mexico	5,776 (2.8%)	
Nigeria	6,827 (3.4%)	
Philippines	5,292 (2.6%)	
Poland	10,389 (5.1%)	
South Africa	2,651 (1.3%)	
Spain	6,290 (3.1%)	
Sweden	15,068 (7.4%)	
Tanzania	9,075 (4.5%)	
Turkey	1,473 (0.7%)	
United Kingdom	5,368 (2.6%)	
United States	38,312 (19%)	

*S.A.R.* Special administrative region.

### Country level E-SWB

Country averages on E-SWB are reported in Table 3. The mean score is reported with associated 95% confidence intervals, country-level standard deviation, and Gini coefficient of inequality. The approximate intraclass correlation coefficients are 9.9% (CL), 7.7% (LS), and 5.6% (H), indicating that greater than 90% of the variability in scores cannot be explained by mean differences in countries, leading to nuanced differences within countries.

**Table 3:** Countries ordered by means on E-SWB.

	Cantril’s ladder					Life satisfaction					Happiness				
Rank	Country	Mean	95% CI	SD	Gini	Country	Mean	95% CI	SD	Gini	Country	Mean	95% CI	SD	Gini
1.	Israel	7.33	(7.18, 7.48)	1.81	0.13	Indonesia	7.99	(7.91, 8.07)	2.29	0.15	Indonesia	8.04	(7.97, 8.11)	2.19	0.14
2.	Sweden	7.20	(7.16, 7.23)	1.76	0.13	Mexico	7.85	(7.78, 7.92)	2.14	0.14	Mexico	7.79	(7.72, 7.85)	2.03	0.14
3.	Poland	7.12	(7.04, 7.21)	1.62	0.13	Egypt	7.69	(7.58, 7.79)	2.83	0.19	Israel	7.76	(7.64, 7.88)	1.65	0.12
4.	Mexico	7.10	(7.03, 7.17)	2.11	0.16	Poland	7.52	(7.43, 7.62)	1.73	0.12	Poland	7.55	(7.46, 7.65)	1.60	0.11
5.	Indonesia	6.97	(6.87, 7.06)	2.52	0.20	Philippines	7.50	(7.42, 7.59)	2.43	0.17	Argentina	7.36	(7.29, 7.44)	2.14	0.16
6.	United States	6.94	(6.89, 6.99)	1.85	0.15	Israel	7.47	(7.31, 7.64)	2.00	0.14	Brazil	7.33	(7.28, 7.39)	2.30	0.17
7.	Hong Kong	6.85	(6.75, 6.94)	2.02	0.16	Argentina	7.22	(7.13, 7.30)	2.38	0.18	Philippines	7.33	(7.24, 7.41)	2.33	0.17
8.	Australia	6.79	(6.71, 6.86)	1.77	0.14	Brazil	7.15	(7.10, 7.21)	2.48	0.19	Kenya	7.27	(7.19, 7.36)	2.96	0.22
9.	Argentina	6.75	(6.67, 6.83)	2.18	0.18	Sweden	7.09	(7.05, 7.13)	2.07	0.16	Hong Kong	7.16	(7.07, 7.26)	2.00	0.15
10.	Germany	6.74	(6.69, 6.78)	1.82	0.15	Hong Kong	7.03	(6.93, 7.13)	2.08	0.16	Nigeria	7.06	(6.95, 7.17)	2.59	0.20
11.	Spain	6.67	(6.60, 6.73)	1.93	0.16	India	7.00	(6.92, 7.09)	3.47	0.26	Sweden	7.03	(6.99, 7.07)	1.96	0.15
12.	Brazil	6.59	(6.54, 6.64)	2.28	0.19	Germany	6.93	(6.88, 6.98)	2.08	0.16	United States	7.01	(6.96, 7.06)	1.92	0.15
13.	United Kingdom	6.56	(6.48, 6.63)	2.01	0.17	United States	6.86	(6.80, 6.91)	2.14	0.17	South Africa	6.95	(6.80, 7.11)	2.65	0.21
14.	Philippines	6.38	(6.30, 6.46)	2.40	0.21	Spain	6.79	(6.72, 6.86)	2.18	0.17	Spain	6.92	(6.86, 6.99)	2.00	0.16
15.	South Africa	6.11	(5.92, 6.29)	2.84	0.26	Australia	6.72	(6.63, 6.81)	2.10	0.17	Germany	6.90	(6.85, 6.95)	1.91	0.15
16.	Japan	5.90	(5.86, 5.93)	2.15	0.20	Nigeria	6.51	(6.38, 6.64)	2.86	0.24	Australia	6.88	(6.81, 6.96)	1.82	0.14
17.	Nigeria	5.73	(5.60, 5.85)	2.79	0.28	United Kingdom	6.49	(6.40, 6.58)	2.31	0.20	United Kingdom	6.71	(6.63, 6.79)	2.08	0.17
18.	India	5.63	(5.53, 5.72)	3.58	0.36	South Africa	6.36	(6.20, 6.52)	2.81	0.25	Tanzania	6.58	(6.46, 6.70)	3.26	0.27
19.	Kenya	5.51	(5.41, 5.61)	3.28	0.34	Japan	6.03	(5.99, 6.07)	2.32	0.21	India	6.48	(6.39, 6.57)	3.56	0.30
20.	Turkey	5.18	(5.02, 5.35)	2.56	0.28	Kenya	5.97	(5.87, 6.07)	3.51	0.33	Japan	6.22	(6.18, 6.25)	2.13	0.19
21.	Egypt	5.04	(4.93, 5.15)	2.87	0.32	Tanzania	5.33	(5.17, 5.50)	3.77	0.40	Egypt	6.18	(6.05, 6.31)	2.94	0.27
22.	Tanzania	4.40	(4.24, 4.56)	3.30	0.42	Turkey	5.19	(4.98, 5.40)	3.24	0.36	Turkey	5.54	(5.35, 5.73)	2.93	0.30

### Variation in E-SWB among demographic groups

Meta-analytic estimates based on subgroups of demographic characteristics are presented in Tables 4 (CL), 5 (LS), and 6 (H). On average, higher E-SWB is associated with: being retired (CL = 6.53/LS = 7.14/H = 7.19), especially relative to those unemployed and job-seeking (5.59/5.97/6.30); being married (6.54/7.12/7.23), especially relative to those separated (5.89/6.31/6.56); attending religious services, especially at least once weekly (6.80/7.40/7.54) relative to never attending (6.12/6.55/6.71); and more education, especially over 16 years (6.64/7.00/7.16) compared to less than eight (6.20/6.84/6.95). Additionally, three factors could be interpreted either as childhood or demographic variables: age/birth cohort, gender, and immigration status. Of these, higher E-SWB is associated with: older age, though levels are fairly high at 18–24 (6.35/6.78/6.96), falling to their lowest at 40–49 (6.18/6.70/6.86), then peaking at 80+ (6.83/7.17/7.39); being female (6.38/6.89/7.03) rather than male (6.31/6.82/6.98), especially relative to the very small percentage reporting their gender as “other” (5.98/5.91/5.95); and living in one’s country of birth (6.34/6.85/7.01) compared to those born elsewhere (6.36/6.81/6.87), although only marginally, and not for CL. Note too though that, for all categories and outcomes, country-level averages varied by at least 0.14 points (gender “other” for CL) and up to 1.12 (“none/other” for employment status for LS), where variability was evaluated with tau (standard deviation country of means). The global p-value was significant (< 0.001) for E-SWB across all demographic characteristics, indicating that in at least one country every demographic characteristic had mean differences on these outcomes among categories. However, levels were similar across demographic categories within at least one country for all characteristics, as shown in the Supplementary country-specific tables.

**Table 4:** Cantril’s ladder meta-analysis of means by demographic category.

				Prediction interval				
Variable category	Est	95% CI	SE	LL	UL	Heterogeneity (τ)		Global p-value
Age group								< 0.001**
18–24	6.35	(6.07,6.62)	0.14	4.99	7.69	0.65	98.1	
25–29	6.31	(6.00,6.62)	0.16	4.75	7.55	0.73	98.2	
30–39	6.22	(5.86,6.57)	0.18	4.32	7.43	0.85	99.3	
40–49	6.18	(5.81,6.54)	0.19	4.15	7.31	0.87	99.2	
50–59	6.32	(5.96,6.68)	0.18	3.97	7.26	0.85	99.2	
60–69	6.43	(6.04,6.82)	0.20	3.88	7.59	0.92	99.3	
70–79	6.51	(6.09,6.94)	0.22	3.45	7.88	0.97	99.0	
80 or older	6.83	(6.42,7.23)	0.21	4.73	7.98	0.80	92.9	
Gender								< 0.001**
Male	6.31	(5.95,6.67)	0.18	4.29	7.46	0.85	99.7	
Female	6.38	(6.07,6.69)	0.16	4.52	7.21	0.74	99.6	
Other	5.98	(5.70,6.26)	0.14	5.68	6.21	0.14	8.6	
Marital status								< 0.001**
Married	6.54	(6.15,6.93)	0.20	4.35	7.73	0.94	99.8	
Separated	5.89	(5.54,6.24)	0.18	3.97	6.85	0.77	93.3	
Divorced	5.93	(5.51,6.36)	0.22	3.62	7.01	0.97	98.2	
Widowed	6.33	(5.91,6.75)	0.21	3.85	7.53	0.98	97.4	
Domestic partner	6.35	(6.04,6.65)	0.15	4.80	7.15	0.64	97.8	
Single, never married	6.15	(5.88,6.42)	0.14	4.79	7.44	0.65	99.0	
Employment status								< 0.001**
Employed for an employer	6.41	(6.07,6.74)	0.17	4.80	7.45	0.80	99.6	
Self-employed	6.40	(6.02,6.78)	0.19	4.37	7.61	0.90	99.1	
Retired	6.53	(6.11,6.94)	0.21	3.69	7.72	0.96	99.3	
Student	6.41	(6.16,6.66)	0.13	5.37	7.84	0.58	95.8	
Homemaker	6.26	(5.95,6.57)	0.16	4.36	7.12	0.72	97.6	
Unemployed and looking for a job	5.59	(5.31,5.88)	0.14	4.31	6.56	0.64	95.5	
None of these/other	5.94	(5.58,6.31)	0.19	3.74	7.61	0.81	95.3	
Education								< 0.001**
Up to 8 years	6.20	(5.83,6.56)	0.19	4.22	7.37	0.86	98.4	
9–15 years	6.33	(6.04,6.63)	0.15	4.95	7.37	0.70	99.6	
16 + years	6.64	(6.36,6.93)	0.14	5.22	7.39	0.67	99.4	
Religious service attendance								< 0.001**
> 1/week	6.80	(6.33,7.27)	0.24	4.36	9.02	1.12	99.1	
1/week	6.60	(6.22,6.97)	0.19	4.41	7.53	0.90	99.1	
1–3/month	6.41	(6.06,6.77)	0.18	4.63	7.27	0.83	98.2	
A few times a year	6.33	(5.99,6.67)	0.18	4.37	7.46	0.82	99.4	
Never	6.12	(5.78,6.46)	0.17	4.40	7.08	0.80	99.6	
Immigration status								< 0.001**
Born in this country	6.34	(6.01,6.68)	0.17	4.41	7.43	0.80	99.8	
Born in another country	6.36	(6.05,6.68)	0.16	4.75	7.14	0.67	95.3	

*N* = 202,898. *p <.05; **p <.007 (Bonferroni corrected threshold); ^ǂ^Group is very small (< 0.1% of the observed sample) within several countries leading to large uncertainty in this estimate—be cautious about interpreting this estimate; LL = lower limits of prediction interval; UL = upper limit of prediction interval; prediction interval is the range of likely values of the estimate for a randomly selected country; τ is the standard deviation of the distribution of means across countries, which is an indicator of cross-national heterogeneity; *I*^2^ is an estimate of the variability in means due to heterogeneity across countries vs. sampling variability which is not uncommonly nearly 100% when there is precision in the estimated mean within country; and the Global *p*-value corresponds to a test of the null hypothesis that there are no differences between the groups for that socio-demographic characteristic in all of the 22 countries.

**Table 5:** Life satisfaction meta-analysis of means by demographic category.

				Prediction interval				
Variable category	Est	95% CI	SE	LL	UL	Heterogeneity (τ)		Global p-value
Age group								< 0.001**
18–24	6.78	(6.43,7.12)	0.18	5.04	8.00	0.82	98.5	
25–29	6.74	(6.37,7.10)	0.19	4.94	8.16	0.87	98.5	
30–39	6.72	(6.37,7.07)	0.18	5.06	8.10	0.83	99.1	
40–49	6.70	(6.35,7.06)	0.18	4.83	7.98	0.85	99.0	
50–59	6.83	(6.51,7.16)	0.17	4.74	7.97	0.76	98.8	
60–69	7.02	(6.71,7.33)	0.16	5.33	8.02	0.72	98.6	
70–79	7.15	(6.83,7.46)	0.16	5.53	8.20	0.69	98.0	
80 or older	7.17	(6.77,7.57)	0.20	4.78	8.09	0.82	92.6	
Gender								< 0.001**
Male	6.82	(6.49,7.16)	0.17	4.94	7.91	0.80	99.6	
Female	6.89	(6.59,7.18)	0.15	5.35	8.09	0.70	99.5	
Other	5.91	(5.45,6.36)	0.23	5.07	6.90	0.55	53.9	
Marital status								< 0.001**
Married	7.12	(6.79,7.44)	0.17	5.35	8.16	0.78	99.6	
Separated	6.31	(5.95,6.67)	0.18	4.24	7.33	0.79	92.7	
Divorced	6.42	(6.07,6.77)	0.18	4.62	7.47	0.76	96.4	
Widowed	6.97	(6.66,7.28)	0.16	5.34	7.94	0.71	94.6	
Domestic partner	6.70	(6.37,7.03)	0.17	5.14	7.96	0.71	97.7	
Single, never married	6.54	(6.18,6.90)	0.18	4.86	7.77	0.85	99.3	
Employment status								< 0.001**
Employed for an employer	6.89	(6.60,7.19)	0.15	5.27	7.85	0.69	99.4	
Self-employed	6.93	(6.59,7.26)	0.17	5.17	8.09	0.78	98.7	
Retired	7.14	(6.82,7.45)	0.16	5.50	8.39	0.73	98.6	
Student	6.81	(6.47,7.15)	0.17	4.92	8.17	0.80	97.1	
Homemaker	6.94	(6.65,7.23)	0.15	5.64	8.16	0.67	97.1	
Unemployed and looking for a job	5.97	(5.57,6.37)	0.20	4.32	7.44	0.94	97.5	
None of these/other	6.31	(5.82,6.81)	0.25	4.03	8.40	1.13	97.0	
Education								< 0.001**
Up to 8 years	6.84	(6.49,7.18)	0.18	5.20	8.05	0.80	98.2	
9–15 years	6.83	(6.51,7.15)	0.16	5.04	7.93	0.76	99.6	
16 + years	7.00	(6.71,7.30)	0.15	5.52	7.97	0.69	99.3	
Religious service attendance								< 0.001**
> 1/week	7.40	(7.02,7.78)	0.19	5.39	8.84	0.90	98.5	
1/week	7.16	(6.86,7.46)	0.15	5.38	8.02	0.71	98.5	
1–3/month	6.92	(6.61,7.23)	0.16	5.36	7.96	0.73	97.5	
A few times a year	6.76	(6.41,7.12)	0.18	4.55	7.86	0.84	99.2	
Never	6.55	(6.23,6.86)	0.16	4.60	7.93	0.74	99.4	
Immigration status								< 0.001**
Born in this country	6.85	(6.54,7.17)	0.16	5.20	7.99	0.75	99.7	
Born in another country	6.81	(6.61,7.01)	0.10	6.02	7.30	0.38	84.0	

*N* = 202,898. *p <.05; **p <.007 (Bonferroni corrected threshold); ^ǂ^Group is very small (< 0.1% of the observed sample) within several countries leading to large uncertainty in this estimate—be cautious about interpreting this estimate; LL = lower limits of prediction interval; UL = upper limit of prediction interval; prediction interval is the range of likely values of the estimate for a randomly selected country; τ is the standard deviation of the distribution of means across countries, which is an indicator of cross-national heterogeneity; *I*^2^ is an estimate of the variability in means due to heterogeneity across countries vs. sampling variability which is not uncommonly nearly 100% when there is precision in the estimated mean within country; and the Global *p*-value corresponds to a test of the null hypothesis that there are no differences between the groups for that socio-demographic characteristic in all of the 22 countries.

**Table 6:** Happiness meta-analysis of means by demographic category.

				Prediction interval				
Variable category	Est	95% CI	SE	LL	UL	Heterogeneity (τ)		Global p-value
Age group								< 0.001**
18–24	6.96	(6.66,7.26)	0.15	5.50	8.07	0.71	98.4	
25–29	6.94	(6.65,7.22)	0.14	5.50	8.16	0.66	97.8	
30–39	6.89	(6.61,7.18)	0.15	5.31	8.11	0.68	98.9	
40–49	6.86	(6.59,7.14)	0.14	5.42	8.05	0.66	98.6	
50–59	6.97	(6.71,7.22)	0.13	5.87	7.88	0.60	98.4	
60–69	7.09	(6.84,7.34)	0.13	5.97	7.94	0.57	98.2	
70–79	7.26	(7.01,7.50)	0.13	5.96	7.96	0.53	97.1	
80 or older	7.40	(7.13,7.67)	0.14	5.87	8.06	0.50	85.6	
Gender								< 0.001**
Male	6.98	(6.73,7.24)	0.13	5.45	7.93	0.62	99.4	
Female	7.03	(6.80,7.26)	0.12	5.71	8.16	0.54	99.2	
Other	5.95	(5.45,6.44)	0.25	4.95	6.98	0.64	62.7	
Marital status								< 0.001**
Married	7.23	(6.98,7.49)	0.13	5.68	8.14	0.61	99.5	
Separated	6.56	(6.28,6.84)	0.14	5.39	7.34	0.60	89.4	
Divorced	6.58	(6.26,6.91)	0.17	4.84	7.61	0.72	96.8	
Widowed	6.92	(6.61,7.22)	0.15	5.15	8.00	0.69	95.2	
Domestic partner	7.01	(6.75,7.27)	0.13	6.15	8.01	0.55	96.8	
Single, never married	6.73	(6.43,7.03)	0.15	5.30	7.88	0.72	99.2	
Employment status								< 0.001**
Employed for an employer	7.04	(6.79,7.29)	0.13	5.72	7.91	0.60	99.3	
Self-employed	7.09	(6.84,7.34)	0.13	5.73	8.08	0.60	98.1	
Retired	7.19	(6.94,7.43)	0.13	5.93	8.04	0.56	98.0	
Student	6.94	(6.64,7.24)	0.15	5.46	8.21	0.70	97.0	
Homemaker	7.00	(6.75,7.25)	0.13	5.67	8.27	0.57	96.6	
Unemployed and looking for a job	6.30	(5.94,6.66)	0.18	4.56	7.49	0.84	97.5	
None of these/other	6.54	(6.18,6.89)	0.18	5.14	8.17	0.78	95.0	
Education								< 0.001**
Up to 8 years	6.95	(6.67,7.24)	0.15	5.61	8.11	0.66	97.7	
9–15 years	7.01	(6.76,7.25)	0.13	5.43	7.99	0.58	99.5	
16 + years	7.16	(6.95,7.36)	0.10	5.78	7.92	0.48	98.7	
Religious service attendance								< 0.001**
> 1/week	7.54	(7.21,7.87)	0.17	6.10	9.22	0.77	98.3	
1/week	7.29	(7.06,7.51)	0.12	5.95	8.04	0.53	97.7	
1–3/month	7.10	(6.86,7.33)	0.12	5.83	7.95	0.54	96.4	
A few times a year	6.92	(6.65,7.18)	0.14	5.03	7.84	0.63	98.9	
Never	6.71	(6.45,6.96)	0.13	4.92	7.88	0.59	99.2	
Immigration status								< 0.001**
Born in this country	7.01	(6.76,7.25)	0.13	5.56	8.04	0.59	99.6	
Born in another country	6.87	(6.61,7.14)	0.13	5.61	7.64	0.55	93.2	

*N* = 202,898. *p <.05; **p <.007 (Bonferroni corrected threshold); ^ǂ^Group is very small (< 0.1% of the observed sample) within several countries leading to large uncertainty in this estimate—be cautious about interpreting this estimate; LL = lower limits of prediction interval; UL = upper limit of prediction interval; prediction interval is the range of likely values of the estimate for a randomly selected country; τ is the standard deviation of the distribution of means across countries, which is an indicator of cross-national heterogeneity; *I*^2^ is an estimate of the variability in means due to heterogeneity across countries vs. sampling variability which is not uncommonly nearly 100% when there is precision in the estimated mean within country; and the Global *p*-value corresponds to a test of the null hypothesis that there are no differences between the groups for that socio-demographic characteristic in all of the 22 countries.

### Childhood experiences associated with E-SWB

Meta-analytic estimates of how childhood experiences are associated with E-SWB are shown in Tables 7 (CL), 8 (LS), and 9 (H). Of these factors, higher E-SWB is associated with: “excellent” self-rated health (RRs = 0.40/0.46/0.50) relative to “good,” especially in contrast to “poor” (−0.40/−0.46/−0.41); a subjective financial status of “living comfortably” (0.29/0.25/0.23) relative those who “got by,” especially contrasted with those who “found it very difficult” (−0.42/−0.31/−0.31); not experiencing abuse (relative to those who did: −0.25/−0.39/−0.33); not feeling like an outsider (relative to those who did: −0.16/−0.29/−0.28); attending religious services, especially once weekly (0.22/0.21/0.27), relative to those who never did; a good relationship with one’s mother (0.17/0.21/0.25) and father (0.18/0.19/0.13); and parents being married, especially relative to parents being single and never married (−0.14/−0.13/−0.13). Note: these Tables also feature the three factors interpretable as either demographic or childhood variables, which were also analysed/presented as demographic factors above. Regarding age, in a childhood factor context this can be understood as pertaining to birth cohort (the time-period people were born/raised in). Some effects were differentially associated with E-SWB depending on the country. The effect of divorced parents was more likely negative (an estimated 41% of effects below −0.10), for example, but could be positive on average in some countries (an estimated 27% of effects above 0.10), conditional on relationship with parents and other variables. Additional country specific effects are reported in Tables S2c-S23c, and more information on the heterogeneity of effects are reported in the Supplemental forest plots (Figures S30).

**Table 7:** Random effects meta-analysis of regressing Cantril’s ladder on childhood predictors.

					Estimated proportion of effects by threshold				
Variable	Category	Est	95% CI	SE	< −0.10	> 0.10	Heterogeneity (τ)		Global p-value
Relationship with mother	(Ref: Very bad/somewhat bad)								< 0.001**
	Very good/somewhat good	0.17	(0.10,0.23)	0.04	0.00	0.82	0.07	23.1	
Relationship with father	(Ref: Very bad/somewhat bad)								< 0.001**
	Very good/somewhat good	0.18	(0.11,0.24)	0.03	0.00	0.77	0.09	39.4	
Parent marital status	(Ref: Parents married)								< 0.001**
	Divorced	−0.02	(−0.08,0.04)	0.03	0.05	0.00	0.06	20.0	
	Single, never married	−0.14	(−0.27,−0.01)	0.07	0.55	0.18	0.24	75.8	
	One or both parents had died	−0.18	(−0.28,−0.09)	0.05	0.77	0.00	0.11	25.9	
Subjective financial status of family growing up	(Ref: Got by)								< 0.001**
	Lived comfortably	0.29	(0.20,0.38)	0.05	0.05	0.82	0.20	88.8	
	Found it difficult	−0.17	(−0.23,−0.11)	0.03	0.73	0.00	0.11	54.5	
	Found it very difficult	−0.42	(−0.55,−0.29)	0.07	0.95	0.00	0.24	64.4	
Abuse	(Ref: No)								< 0.001**
	Yes	−0.25	(−0.34,−0.16)	0.04	0.90	0.05	0.16	70.8	
Outsider growing up	(Ref: No)								< 0.001**
	Yes	−0.16	(−0.25,−0.07)	0.05	0.59	0.05	0.18	72.4	
Self-rated health growing up	(Ref: Good)								< 0.001**
	Excellent	0.40	(0.26,0.55)	0.07	0.05	0.91	0.33	92.1	
	Very good	0.24	(0.15,0.33)	0.05	0.00	0.73	0.18	81.7	
	Fair	−0.25	(−0.33,−0.17)	0.04	0.91	0.00	0.12	47.3	
	Poor	−0.40	(−0.59,−0.22)	0.09	0.82	0.09	0.31	58.4	
Immigration status	(Ref: Born in this country)								< 0.001**
	Born in another country	0.09	(−0.02,0.20)	0.05	0.18	0.36	0.15	48.5	
Age 12 religious service attendance	(Ref: Never)								< 0.001**
	At least 1/week	0.22	(0.11,0.33)	0.05	0.09	0.82	0.20	74.2	
	1–3/month	0.23	(0.12,0.34)	0.06	0.05	0.73	0.21	75.1	
	< 1/month	0.12	(0.06,0.17)	0.03	0.00	0.55	0.06	31.2	
Year of birth	(Ref: 1998–2005; age 18–24)								< 0.001**
	1993–1998; age 25–29	−0.00	(−0.08,0.07)	0.04	0.23	0.23	0.12	45.9	
	1983–1993; age 30–39	−0.06	(−0.19,0.07)	0.07	0.41	0.27	0.29	87.2	
	1973–1983; age 40–49	−0.11	(−0.26,0.04)	0.08	0.55	0.23	0.34	89.1	
	1963–1973; age 50–59	0.03	(−0.14,0.19)	0.08	0.32	0.45	0.36	89.1	
	1953–1963; age 60–69	0.14	(−0.08,0.36)	0.11	0.27	0.50	0.49	92.2	
	1943–1953; age 70–79	0.20	(−0.10,0.50)	0.15	0.27	0.50	0.65	93.5	
	1943 or earlier; age 80+	0.27	(−0.10,0.65)	0.19	0.27	0.64	0.75	89.3	
Gender	(Ref: Male)								< 0.001**
	Female	0.12	(0.03,0.20)	0.04	0.09	0.55	0.19	90.7	
	Other	0.01	(−0.56,0.58)	0.29	0.56	0.39	1.08	90.7	

*N* = 202,898. *p <.05; **p <.004 (Bonferroni corrected threshold); ^ǂ^Group is very small (< 0.1% of the observed sample) within several countries leading large uncertainty in this estimate—be cautious about interpreting this estimate; CI = confidence interval; the estimated proportion of effects is the estimated proportion of effects above (or below) a threshold based on the calibrated effect sizes (Mathur & VanderWeele, 2020); *I*^2^ is an estimate of the variability in means due to heterogeneity across countries vs. sampling variability; the Global *p*-value corresponds to the joint test of the null hypothesis that the country-specific joint parameter Wald tests (all parameters within variable groups are zero) are all null in all 22 countries; and additional details of heterogeneity of effects are available in the forest plots of our online supplemental material.

**Table 8:** Random effects meta-analysis of regressing life satisfaction on childhood predictors.

					Estimated proportion of effects by threshold				
Variable	Category	Est	95% CI	SE	< −0.10	> 0.10	Heterogeneity (τ)		Global p-value
Relationship with mother	(Ref: Very bad/somewhat bad)								0.009*
	Very good/somewhat good	0.21	(0.12,0.29)	0.04	0.00	0.86	0.10	29.9	
Relationship with father	(Ref: Very bad/somewhat bad)								< 0.001**
	Very good/somewhat good	0.19	(0.10,0.27)	0.04	0.00	0.73	0.13	54.2	
Parent marital status	(Ref: Parents married)								< 0.001**
	Divorced	−0.04	(−0.17,0.09)	0.06	0.36	0.18	0.25	77.3	
	Single, never married	−0.13	(−0.25,−0.01)	0.06	0.45	0.18	0.22	69.7	
	One or both parents had died	−0.08	(−0.24,0.08)	0.08	0.45	0.23	0.30	71.1	
Subjective financial status of family growing up	(Ref: Got by)								< 0.001**
	Lived comfortably	0.25	(0.17,0.33)	0.04	0.00	0.82	0.16	81.6	
	Found it difficult	−0.14	(−0.22,−0.07)	0.04	0.68	0.09	0.14	65.3	
	Found it very difficult	−0.31	(−0.46,−0.16)	0.08	0.77	0.00	0.27	66.7	
Abuse	(Ref: No)								< 0.001**
	Yes	−0.39	(−0.48,−0.30)	0.04	0.95	0.00	0.15	64.7	
Outsider growing up	(Ref: No)								< 0.001**
	Yes	−0.29	(−0.39,−0.20)	0.05	0.82	0.00	0.18	68.8	
Self-rated health growing up	(Ref: Good)								< 0.001**
	Excellent	0.46	(0.29,0.62)	0.08	0.00	0.82	0.37	93.0	
	Very good	0.25	(0.15,0.35)	0.05	0.05	0.68	0.21	83.1	
	Fair	−0.33	(−0.41,−0.26)	0.04	1.00	0.00	0.10	32.4	
	Poor	−0.46	(−0.70,−0.22)	0.12	0.86	0.09	0.47	73.5	
Immigration status	(Ref: Born in this country)								< 0.001**
	Born in another country	0.09	(−0.08,0.26)	0.09	0.18	0.50	0.30	75.2	
Age 12 religious service attendance	(Ref: Never)								< 0.001**
	At least 1/week	0.21	(0.03,0.38)	0.09	0.18	0.77	0.37	88.2	
	1–3/month	0.16	(−0.01,0.32)	0.08	0.23	0.64	0.34	86.7	
	< 1/month	0.05	(−0.09,0.19)	0.07	0.18	0.41	0.28	87.5	
Year of birth	(Ref: 1998–2005; age 18–24)								< 0.001**
	1993–1998; age 25–29	−0.00	(−0.10,0.09)	0.05	0.32	0.27	0.17	60.9	
	1983–1993; age 30–39	−0.00	(−0.15,0.14)	0.08	0.27	0.41	0.33	87.9	
	1973–1983; age 40–49	−0.03	(−0.22,0.16)	0.10	0.36	0.45	0.43	91.6	
	1963–1973; age 50–59	0.08	(−0.15,0.30)	0.11	0.32	0.59	0.50	93.0	
	1953–1963; age 60–69	0.24	(0.01,0.46)	0.11	0.23	0.64	0.49	91.1	
	1943–1953; age 70–79	0.33	(0.02,0.65)	0.16	0.27	0.59	0.69	93.2	
	1943 or earlier; age 80+	0.44	(0.01,0.88)	0.22	0.27	0.68	0.94	91.3	
Gender	(Ref: Male)								< 0.001**
	Female	0.12	(0.03,0.21)	0.05	0.09	0.50	0.20	90.5	
	Other	−0.29	(−0.63,0.05)	0.17	0.61	0.17	0.53	63.0	

*N* = 202,898. *p <.05; **p <.004 (Bonferroni corrected threshold); ^ǂ^Group is very small (< 0.1% of the observed sample) within several countries leading large uncertainty in this estimate—be cautious about interpreting this estimate; CI = confidence interval; the estimated proportion of effects is the estimated proportion of effects above (or below) a threshold based on the calibrated effect sizes (Mathur & VanderWeele, 2020); *I*^2^ is an estimate of the variability in means due to heterogeneity across countries vs. sampling variability; the Global *p*-value corresponds to the joint test of the null hypothesis that the country-specific joint parameter Wald tests (all parameters within variable groups are zero) are all null in all 22 countries; and additional details of heterogeneity of effects are available in the forest plots of our online supplemental material.

**Table 9:** Random effects meta-analysis of regressing happiness on childhood predictors.

					Estimated proportion of effects by threshold				
Variable	Category	Est	95% CI	SE	< −0.10	> 0.10	Heterogeneity (τ)		Global p-value
Relationship with mother	(Ref: Very bad/somewhat bad)								< 0.001**
	Very good/somewhat good	0.25	(0.15,0.36)	0.05	0.05	0.86	0.17	62.4	
Relationship with father	(Ref: Very bad/somewhat bad)								< 0.001**
	Very good/somewhat good	0.13	(0.03,0.22)	0.05	0.09	0.55	0.16	69.7	
Parent marital status	(Ref: Parents married)								< 0.001**
	Divorced	−0.04	(−0.15,0.08)	0.06	0.45	0.27	0.23	77.4	
	Single, never married	−0.13	(−0.22,−0.04)	0.05	0.55	0.05	0.15	52.4	
	One or both parents had died	−0.10	(−0.23,0.02)	0.06	0.50	0.14	0.22	57.8	
Subjective financial status of family growing up	(Ref: Got by)								< 0.001**
	Lived comfortably	0.23	(0.16,0.30)	0.04	0.00	0.77	0.15	80.6	
	Found it difficult	−0.12	(−0.18,−0.05)	0.03	0.59	0.05	0.12	60.9	
	Found it very difficult	−0.31	(−0.42,−0.21)	0.05	0.86	0.00	0.17	45.9	
Abuse	(Ref: No)								< 0.001**
	Yes	−0.33	(−0.42,−0.24)	0.04	0.95	0.05	0.16	69.5	
Outsider growing up	(Ref: No)								< 0.001**
	Yes	−0.28	(−0.37,−0.20)	0.04	0.91	0.00	0.15	65.0	
Self-rated health growing up	(Ref: Good)								< 0.001**
	Excellent	0.50	(0.34,0.66)	0.08	0.00	0.86	0.37	93.8	
	Very good	0.27	(0.17,0.36)	0.05	0.00	0.77	0.20	83.8	
	Fair	−0.28	(−0.36,−0.20)	0.04	0.95	0.00	0.13	50.2	
	Poor	−0.41	(−0.62,−0.20)	0.11	0.82	0.14	0.38	67.8	
Immigration status	(Ref: Born in this country)								< 0.001**
	Born in another country	0.01	(−0.14,0.16)	0.08	0.41	0.32	0.26	73.5	
Age 12 religious service attendance	(Ref: Never)								< 0.001**
	At least 1/week	0.27	(0.14,0.40)	0.07	0.09	0.82	0.26	80.9	
	1–3/month	0.23	(0.09,0.36)	0.07	0.14	0.64	0.27	82.7	
	< 1/month	0.12	(0.06,0.18)	0.03	0.00	0.55	0.09	45.9	
Year of birth	(Ref: 1998–2005; age 18–24)								< 0.001**
	1993–1998; age 25–29	−0.01	(−0.11,0.09)	0.05	0.23	0.36	0.20	71.0	
	1983–1993; age 30–39	−0.01	(−0.14,0.11)	0.07	0.27	0.32	0.28	86.9	
	1973–1983; age 40–49	−0.05	(−0.22,0.12)	0.09	0.32	0.50	0.39	91.5	
	1963–1973; age 50–59	0.03	(−0.19,0.25)	0.11	0.23	0.55	0.50	94.1	
	1953–1963; age 60–69	0.14	(−0.10,0.38)	0.12	0.27	0.55	0.54	93.7	
	1943–1953; age 70–79	0.27	(−0.04,0.58)	0.16	0.32	0.59	0.69	94.3	
	1943 or earlier; age 80+^ǂ^	0.46	(0.10,0.81)	0.18	0.32	0.64	0.72	88.5	
Gender	(Ref: Male)								< 0.001**
	Female	0.10	(0.03,0.17)	0.03	0.05	0.55	0.14	84.0	
	Other^ǂ^	−0.69	(−0.98,−0.40)	0.15	0.94	0.00	0.39	51.4	

*N* = 202,898. *p <.05; **p <.004 (Bonferroni corrected threshold); ^ǂ^Group is very small (< 0.1% of the observed sample) within several countries leading large uncertainty in this estimate—be cautious about interpreting this estimate; CI = confidence interval; the estimated proportion of effects is the estimated proportion of effects above (or below) a threshold based on the calibrated effect sizes (Mathur & VanderWeele, 2020); *I*^2^ is an estimate of the variability in means due to heterogeneity across countries vs. sampling variability; the Global *p*-value corresponds to the joint test of the null hypothesis that the country-specific joint parameter Wald tests (all parameters within variable groups are zero) are all null in all 22 countries; and additional details of heterogeneity of effects are available in the forest plots of our online supplemental material.

### Sensitivity of childhood variables to unmeasured confounding

An important consideration in the childhood results is the sensitivity of estimates to unmeasured confounding, as reported in Tables 10 (CL), 11 (LS), and 12 (H). Some were moderately robust. For example, to explain away the association between excellent (versus good) self-rated health in childhood with adult H, an unmeasured confounder associated with both excellent health and higher H with risk ratios of 1.73 each, above and beyond measured covariates, could suffice, but weaker joint confounder associations could not; to shift the 95% confidence interval to include the null, an unmeasured confounder associated with both excellent health and higher H with risk ratios of 1.55 each, above and beyond measured covariates, could suffice, but weaker joint confounder associations could not. However, other associations were less robust. Sensitivity of associations for country specific analyses are reported in Tables S2d-S23d.

**Table 10:** Sensitivity of meta-analyzed childhood predictors of Cantril’s ladder to unmeasured confounding.

Variable	Category	E-value for estimate	E-value for 95% CI
Relationship with mother	(Ref: Very bad/somewhat bad)		
	Very good/somewhat good	1.33	1.24
Relationship with father	(Ref: Very bad/somewhat bad)		
	Very good/somewhat good	1.35	1.26
Parent marital status	(Ref: Parents married)		
	Divorced	1.09	1.00
	Single, never married	1.30	1.07
	One or both parents had died	1.35	1.22
Subjective financial status of family	(Ref: Got by)		
growing up	Lived comfortably	1.49	1.38
	Found it difficult	1.34	1.25
	Found it very difficult	1.63	1.48
Abuse	(Ref: No)		
	Yes	1.44	1.33
Outsider growing up	(Ref: No)		
	Yes	1.32	1.19
Self-rated health growing up	(Ref: Good)		
	Excellent	1.62	1.45
	Very good	1.43	1.32
	Fair	1.44	1.34
	Poor	1.62	1.40
Immigration status	(Ref: Born in this country)		
	Born in another country	1.23	1.00
Age 12 religious service attendance	(Ref: Never)		
	At least 1/week	1.40	1.26
	1–3/month	1.41	1.27
	< 1/month	1.27	1.18
Year of birth	(Ref: 1998–2005; age 18–24)		
	1993–1998; age 25–29	1.03	1.00
	1983–1993; age 30–39	1.18	1.00
	1973–1983; age 40–49	1.26	1.00
	1963–1973; age 50–59	1.11	1.00
	1953–1963; age 60–69	1.30	1.00
	1943–1953; age 70–79	1.37	1.00
	1943 or earlier; age 80+^ǂ^	1.47	1.00
Gender	(Ref: Male)		
	Female	1.27	1.12
	Other^ǂ^	1.08	1.00

*N* = 202,898; the E-value is the minimum strength of the association an unmeasured confounder must have with both the outcome and the predictor, above and beyond all measured covariates, for an unmeasured confounder to explain away an association (VanderWeele & Ding, 2017, p. 269–270); and ^ǂ^Group is very small (< 0.1% of the observed sample) within several countries potentially large uncertainty in this estimate—be cautious about interpreting this estimate.

**Table 11:** Sensitivity of meta-analyzed childhood predictors of life satisfaction to unmeasured confounding.

Variable	Category	E-value for estimate	E-value for 95% CI
Relationship with mother	(Ref: Very bad/somewhat bad)		
	Very good/somewhat good	1.37	1.26
Relationship with father	(Ref: Very bad/somewhat bad)		
	Very good/somewhat good	1.34	1.24
Parent marital status	(Ref: Parents married)		
	Divorced	1.14	1.00
	Single, never married	1.27	1.05
	One or both parents had died	1.21	1.00
Subjective financial status of family	(Ref: Got by)		
Growing up	Lived comfortably	1.42	1.33
	Found it difficult	1.29	1.18
	Found it very difficult	1.49	1.32
Abuse	(Ref: No)		
	Yes	1.57	1.48
Outsider growing up	(Ref: No)		
	Yes	1.46	1.35
Self-rated health growing up	(Ref: Good)		
	Excellent	1.64	1.46
	Very good	1.42	1.30
	Fair	1.51	1.43
	Poor	1.65	1.38
Immigration status	(Ref: Born in this country)		
	Born in another country	1.21	1.00
Age 12 religious service attendance	(Ref: Never)		
	At least 1/week	1.37	1.12
	1–3/month	1.31	1.00
	< 1/month	1.16	1.00
Year of birth	(Ref: 1998–2005; age 18–24)		
	1993–1998; age 25–29	1.03	1.00
	1983–1993; age 30–39	1.04	1.00
	1973–1983; age 40–49	1.12	1.00
	1963–1973; age 50–59	1.20	1.00
	1953–1963; age 60–69	1.40	1.08
	1943–1953; age 70–79	1.51	1.09
	1943 or earlier; age 80+^ǂ^	1.63	1.05
Gender	(Ref: Male)		
	Female	1.26	1.11
	Other^ǂ^	1.46	1.00

*N* = 202,898; the E-value is the minimum strength of the association an unmeasured confounder must have with both the outcome and the predictor, above and beyond all measured covariates, for an unmeasured confounder to explain away an association (VanderWeele & Ding, 2017, p. 269–270); and ^ǂ^Group is very small (< 0.1% of the observed sample) within several countries potentially large uncertainty in this estimate—be cautious about interpreting this estimate.

**Table 12:** Sensitivity of meta-analyzed childhood predictors of happiness to unmeasured confounding.

Variable	Category	E-value for estimate	E-value for 95% CI
Relationship with mother	(Ref: Very bad/somewhat bad)		
	Very good/somewhat good	1.45	1.31
Relationship with father	(Ref: Very bad/somewhat bad)		
	Very good/somewhat good	1.28	1.13
Parent marital status	(Ref: Parents married)		
	Divorced	1.13	1.00
	Single, never married	1.28	1.13
	One or both parents had died	1.25	1.00
Subjective financial status of family	(Ref: Got by)		
growing up	Lived comfortably	1.41	1.32
	Found it difficult	1.27	1.17
	Found it very difficult	1.52	1.39
Abuse	(Ref: No)		
	Yes	1.54	1.43
Outsider growing up	(Ref: No)		
	Yes	1.48	1.38
Self-rated health growing up	(Ref: Good)		
	Excellent	1.73	1.55
	Very good	1.46	1.35
	Fair	1.48	1.38
	Poor	1.63	1.38
Immigration status	(Ref: Born in this country)		
	Born in another country	1.05	1.00
Age 12 religious service attendance	(Ref: Never)		
	At least 1/week	1.47	1.31
	1–3/month	1.42	1.24
	< 1/month	1.27	1.17
Year of birth	(Ref: 1998–2005; age 18–24)		
	1993–1998; age 25–29	1.06	1.00
	1983–1993; age 30–39	1.08	1.00
	1973–1983; age 40–49	1.16	1.00
	1963–1973; age 50–59	1.12	1.00
	1953–1963; age 60–69	1.30	1.00
	1943–1953; age 70–79	1.47	1.00
	1943 or earlier; age 80+^ǂ^	1.68	1.25
Gender	(Ref: Male)		
	Female	1.24	1.13
	Other^ǂ^	1.95	1.62

*N* = 202,898; the E-value is the minimum strength of the association an unmeasured confounder must have with both the outcome and the predictor, above and beyond all measured covariates, for an unmeasured confounder to explain away an association (VanderWeele & Ding, 2017, p. 269–270); and ^ǂ^Group is very small (< 0.1% of the observed sample) within several countries potentially large uncertainty in this estimate—be cautious about interpreting this estimate.

### Post-Hoc analyses

Finally, we conducted various post-hoc analyses to help us interpret the data, including analyzing the three E-SWB items in terms of: (a) their correlations with flourishing in the GFS; (b) by world region; and (c) their correlations with GDP-per-capita and Gini.

Firstly then, we computed the meta-analytic pooled correlations among the E-SWB outcomes and the flourishing items in VanderWeele’s “Secure Flourish Index” (which itself includes two of our E-SWB items, namely LS and H). Both LS and H had much stronger average meta-analytic mean correlation coefficients with all other items (both 0.45) compared to CL (0.38). Likewise, when separated by domain (excluding the first domain, since this comprises LS and H themselves), LS and H had stronger correlations for all other main domains (with H marginally higher than LS on five, and vice versa on two, with one equal). Relatedly, LS-H were more tightly correlated (0.69) than CL-LS or CL-H (both 0.57). With the additional sixth domain, LS also had the strongest correlations, closely followed this time by CL then H.

Second, we explored the levels of E-SWB across world regions, including in terms of those classified as relatively WEIRD versus non-WEIRD. As with the correlations above, LS and H often seem closely related while CL captures something different: countries categorized as relatively non-WEIRD collectively have lower average CL (5.81) and higher LS (7.02) and H (6.74), while relatively WEIRD nations have less obvious clustering (6.60/7.03/7.16). Thirdly, we gathered data from the World Bank for each country in terms of GDP-per-capita and Gini (Table 15) and explored their correlation with E-SWB (Table 16). In terms of GDP-per-capita, once again, LS and H seem closely related—with near zero correlations for LS (0.05) and H (−0.08)—while CL stood apart (0.58 correlation). However, in terms of Gini, CL and LS were closer together (−0.11 and − 0.20), while H was somewhat distinct (0.07).

**Table 13:** Correlations between E-SWB and flourishing domains

	Cantril’s ladder	Life satisfaction	Happiness	Physical health	Mental health	Life worthwhile	Life purpose	Promote good	Delay gratification	Relationships (content)	Relationships (satisfied)	Financial security	Material security
Cantril’s ladder													
Life satisfaction	**0.57**												
Happiness	**0.57**	**0.69**											
Physical health	**0.37**	**0.41**	**0.43**										
Mental health	**0.39**	**0.49**	**0.51**	0.53									
Life worthwhile	**0.46**	**0.61**	**0.58**	0.40	0.49								
Life purpose	**0.34**	**0.42**	**0.43**	0.35	0.45	0.50							
Promote good	**0.28**	**0.33**	**0.35**	0.33	0.39	0.40	0.43						
Delay gratification	**0.23**	**0.27**	**0.28**	0.27	0.29	0.30	0.35	0.42					
Relationships (content)	**0.35**	**0.44**	**0.45**	0.32	0.43	0.44	0.47	0.37	0.29				
Relationships (satisfied)	**0.36**	**0.46**	**0.46**	0.33	0.42	0.44	0.45	0.35	0.28	0.70			
Financial security	**0.34**	**0.35**	**0.33**	0.26	0.27	0.28	0.22	0.16	0.13	0.23	0.25		
Material security	**0.31**	**0.34**	**0.32**	0.24	0.26	0.27	0.22	0.16	0.13	0.23	0.24	0.70	

**Table 14:** World region estimates of E-SWB.

Region	Cantril’s ladder		Life satisfaction		Happiness	
	Mean	95% CI	Mean	95% CI	Mean	95% CI
WEIRD*	6.80	(6.77,6.82)	7.03	(7.00,7.05)	7.16	(7.14,7.19)
Non-WEIRD*	5.81	(5.75,5.88)	7.02	(6.97,7.07)	6.74	(6.69,6.80)
Africa	5.46	(5.39,5.53)	6.54	(6.47,6.61)	6.82	(6.76,6.88)
Asia	5.87	(5.80,5.94)	7.01	(6.95,7.07)	6.69	(6.62,6.75)
Europe	6.76	(6.72,6.79)	6.88	(6.85,6.92)	6.96	(6.93,6.99)
North America	6.98	(6.94,7.02)	7.11	(7.07,7.16)	7.21	(7.17,7.25)
South America	6.62	(6.58,6.66)	7.16	(7.12,7.21)	7.34	(7.29,7.38)
Australia	6.79	(6.71,6.87)	6.72	(6.63,6.81)	6.88	(6.81,6.95)

All estimates are based on the country-specific overall means reported in Table 3 and pooled using population weighted meta-analyses. Country groupings are as follows. WEIRD: Australia, Argentina, Brazil, Germany, Israel, Mexico, Poland, South Africa, Spain, Sweden, US, UK; Non-WEIRD: Egypt, Hong Kong, India, Indonesia, Japan, Kenya, Nigeria, Philippines, Tanzania, Turkey. Then, grouping countries by continent: Europe: Germany, Poland, Spain, Sweden, UK; North America: US, Mexico; South America: Argentina, Brazil; Africa: Egypt, Kenya, Nigeria, South Africa, Tanzania; Asia: Hong Kong, India, Indonesia, Israel, Japan, Philippines, Turkey; Oceana: Australia. Weights used for countries are reported in Padgett et al.^47^ *As also noted in the Discussion, we regard these labels as problematic and do not wish to reify any kind of binary distinction, and concur with Ghai^54^ that “It’s time to reimagine sample diversity and retire the WEIRD dichotomy.” Ghai argues that “The world’s population does not split neatly into two groups, WEIRD and non-WEIRD people” and that because “the non-WEIRD brush does not do justice to the complexity of human lives” it is important for “behavioural science to ensure that samples represent human diversity.” One issue with this binary that is particularly pertinent here is that many countries are not easily placed in either category. One option might be to simply group countries by continent, placing African and Asian countries in the non-WEIRD category and the other continents in the WEIRD category. However, countries like South Africa and Israel are often positioned as relatively Western, even if that characterization is itself contentious, while conversely it is sometimes ambiguous or contested whether South America should be considered Western or not. For the sake of our calculation, we have included these two countries and South America in the WEIRD category. However, we are not particularly confident or enthusiastic about the country groupings deployed here, and would invite readers to hold the categorizations relatively lightly and just use them as rough, imperfect classifications for illustrative purposes.

## Discussion

This paper makes three useful contributions to the already-extensive literature on E-SWB: (1) shedding light on whether CL, LS or H “best” represents E-SWB—at least in terms of their association with flourishing; (2) providing expansive coverage of 15 factors associated with E-SWB; and (3) allowing granular exploration of cross-national variation. Taken in isolation each contribution may not be remarkable (though (1) *is* quite rare), but the combination of all three is arguably unique. Moreover, each contribution magnifies the value of the others; for example, our understanding of the contribution of the 15 factors is deepened by us also being able to explore cross-national variation in that regard. Here we delve into each contribution in turn, before then considering various limitations of the study.

### Contribution 1: comparing E-SWB items

Our first contribution is comparing three options for assessing E-SWB. Here we briefly note which item(s) we recommend, then delve a little into the reasoning behind these choices, and finally reflect on the validity of our H item as a gauge of E-SWB. Firstly then, in short, we would recommend that, if needing to pick just one E-SWB item (e.g., if space in a survey is limited), then LS and H are both better than CL, but LS is perhaps the best (given that H is potentially ambiguous). Then, if space allows for two items, then *CL* would be a good second addition (since LS and H seem very closely related, while CL taps into somewhat different aspects of E-SWB). Ideally though one would include all three items: we concur with Helliwell that while each can offer a good overall gauge of E-SWB, they have subtle differences, so an average of all three would provide “a clearer picture than either measure on its own.”^22^ Another caveat is that the preferability of LS pertains more to instances where one is initiating a new survey; in cases where surveys are already in place and use CL, then it may be worth retaining the existing methodology for the sake of consistency. In the case of the WHR, for instance, any advantage gained by switching from CL to LS (e.g., in terms of better indexing flourishing) would be outweighed by the​ disadvantage of losing the longitudinal dimension of the WHR having already tracked CL for well over a decade. But absent such considerations, our data do suggest that LS may be the best single gauge of E-SWB. In terms of how we reached this conclusion, this was through, (a) comparing the items’ associations with flourishing, and (b) examining the differential country rankings. In terms of (a) then, we explored the relative associations of CL, LS, and H with flourishing, as gauged by VanderWeele’s 12-item Secure Flourish Index (though note that LS and H themselves constitute one of the five main domains). In that regard, as shown in Table 13, both LS and H had much stronger average meta-analytic mean correlation coefficients with all other items compared to CL (with the only exception being financial security, in which although LS still had the strongest correlation, it was closely followed by *CL* then H). Relatedly, LS-H were more tightly correlated than CL-LS or CL-H.

**Table 15:** GDP per capita (PPP, current international $) and Gini Index.

Country	GDP per capita, PPP^1^	GDP ranking	Gini Index^2^	Gini ranking
Argentina	29,362.7	12	40.7	15
Australia	69,114.7	5	34.3	9
Brazil	20,584.4	14	52.0	20
Egypt	18,816.3	15	31.9	3
Germany	69,338.3	4	32.4	4
Hong Kong	71,481.6	2	-	-
India	10,175.8	19	32.8	6
Indonesia	15,612.8	17	36.1	11
Israel	53,434.0	7	37.9	12
Japan	50,206.6	9	32.9	7
Kenya	6,323.5	20	38.7	13
Mexico	25,601.6	13	43.5	18
Nigeria	6,318.2	21	35.1	10
Philippines	10,755.5	18	40.7	16
Poland	49,464.0	10	28.5	1
South Africa	15,847.4	16	63.5	21
Spain	52,779.2	8	33.9	8
Sweden	70,206.6	3	29.8	2
Tanzania	3,972.6	22	40.5	14
Türkiye	44,151.0	11	44.4	19
UK	58,906.2	1	32.4	5
US	81,695.2	6	41.3	17

^1^World Bank | World Development Indicators database: https://data.worldbank.org/indicator/NY.GDP.PCAP.PP.CD

^2^World Bank | World Development Indicators database: https://data.worldbank.org/indicator/si.pov.gini

A second lens through which to compare the items is the relative performance of countries on them. While the data are complex, the main pattern observed is that, as with the correlations above, LS and H often seem closely related (e.g., scores for countries tend to track together), while CL captures something different. There are exceptions, (e.g., Egypt), but this pattern holds across many countries. It also applies when grouping countries by region (see Table 14), especially those categorized as relatively non-WEIRD—even while we are wary of this binary^54^ and had difficulty grouping the countries, as footnoted in the Table—which have lower average CL and higher LS and H, while relatively WEIRD nations have less obvious clustering. Moreover, these patterns are evident when our data is compared with GDP-per-capita (see Table 15), with a strong correlation with CL (corroborating prior research^69^), but near zero for LS and H. The close linkage of CL and GDP has also recently been illustrated by Natural Language Processing analyses of respondents’ interpretation of the CL item, which suggests the ladder metaphor encourages people to think of power, achievement, and success and to evaluate their life in those terms^70^. Notably though, despite LS having a near-zero correlation with GDP-per-capita, it still had the strongest correlation with personal financial/material security in the GFS, suggesting such security is only weakly related to GDP per se. Further nuance regarding economics is also provided by correlations with Gini, where CL and LS were closer together, while H was somewhat distinct. Given that higher Gini scores mean greater inequality, this implies that more equal places are liable to higher CL and LS, but perhaps counterintuitively slightly lower H (though these correlations are very weak), which demands further attention in future work.

**Table 16:** Estimated correlations (95% CI) with GDP per capita and Gini Index

Indicator	GDP		Gini index	
Correlation	Product moment	Rank	Product moment	Rank
Cantril’s ladder	0.58 (0.21,0.80)	0.58 (0.21,0.80)	-0.11 (-0.52,0.34)	0.13 (-0.32,0.53)
Life satisfaction	0.05 (-0.38,0.46)	0.04 (-0.39,0.45)	-0.20 (-0.58,0.26)	0.16 (-0.29,0.55)
Happiness	-0.08 (-0.48,0.36)	-0.08 (-0.49,0.35)	0.07 (-0.38,0.48)	-0.24 (-0.61,0.21)

An interesting comparison which illustrates the difference between the items is Sweden versus Indonesia, in which the relative performance of the constructs is reversed. Sweden does very well on CL (2nd in our data and the 2024 WHR^18^), but is only mid-ranked for LS and H. By contrast, although Indonesia excels on all three, it is first for both LS and H (with nearly identical scores), and “only” fifth on CL (with a rather lower score). Given that CL seems to particularly tap into financial/material security, and likewise strongly correlates with GDP-per-capita, it is relevant then that Sweden fares very well on the latter and Gini and Indonesia less so. Further context to this comparison between Sweden and Indonesia, and by extension, CL versus LS/H, is provided by an analysis of GWP data comparing 145 countries (for most items) on 38 metrics pertaining to wellbeing^71^. Overall, Sweden did better on variables relating to standards of living, like feeling stable and secure (ranked 1^st^, vs. Indonesia at 66), having money for shelter (1 vs. 93), and satisfaction with standard of living (2 vs. 53). Indonesia by contrast excelled on items pertaining to the other flourishing domains, including: physical and mental health (e.g., well-rested: Indonesia 4th, Sweden 64th); character and virtue (e.g., volunteering: 1 vs. 109); and social relationships (e.g., opportunities to make friends: 5 vs. 23). Given the correlations with the flourishing domains, it becomes understandable why Indonesia would fare so well on LS and H whereas Sweden does better on CL.

Finally, we should briefly acknowledge that the suitability of H as a gauge of E-SWB is not a *given*, since it is commonly used/interpreted as an affective concept. However, not only can it also have evaluative aspects or connotations—and indeed Diener himself favoured this usage^17^—our data here also support this interpretation. As discussed below regarding age, for instance, E-SWB is widely regarded as relatively U-shaped over the lifespan while positive affect tends to decline^28^; significantly, here H followed the same somewhat U-shaped pattern as CL and LS (though we suggest below it may actually be more "J-shaped"). We thus regard H as a viable candidate for assessing E-SWB, including even *our* item specifically. Despite asking how happy participants “feel” (as opposed to a more explicit cognitive framing like “are you happy with your life?”), the qualifier “usually” takes the item into the conceptual territory described by Diener and colleagues as evaluative (“a relatively stable feeling of happiness one has towards his or her life”), while more immediate qualifiers like “right now” or “yesterday” might invite an affective interpretation (“the momentary feeling state of happiness”), and we agree with Helliwell^19^ that this very temporal framing is what shifts the balance between evaluative and affective understandings of H. Alternatively though, the phrase “usually feels” could conceivably prompt participants to think about their general genetically-influenced temperamental “baseline” of positive affect^72,73^. As such, we recognize some people may object to interpreting our H item as evaluative, in which case, in terms of adjudicating between different E-SWB items, they could just use our data to decide between CL and LS. And to reiterate, in that regard we suggest that LS might be preferable, based on it offering a better gauge of flourishing in our data.

### Contribution 2: exploring the relative contribution of factors

Our second key contribution is comprehensive coverage of 15 E-SWB-related factors. While each has a substantial literature exploring their association with E-SWB, rarely are so many considered together, which allows one to ascertain their *relative* strength of association. Here we briefly note the main patterns, before delving a little into the three factors with the greatest variation—employment status, childhood health, and age—to illustrate and contextualize the contribution of our findings.

Regarding the main patterns, in headline terms, of the purely demographic factors, higher E-SWB is associated with: being retired, especially relative to those unemployed and job-seeking; being married, especially relative to those separated; attending religious services, especially at least once weekly relative to never attending; and more education, especially over 16 years compared to less than eight. Then, of the childhood factors, higher E-SWB is associated with: “excellent” self-rated health relative to “good,” especially in contrast to “poor”; a subjective financial status of “living comfortably” relative those who “got by,” especially contrasted with those who “found it very difficult”; not experiencing abuse (relative to those who did); not feeling like an outsider (relative to those who did); attending religious services, especially once weekly, relative to those who never did; having had a good relationship with one’s mother and father; and one’s parents being married, especially relative to parents being single and never married. Finally, three factors could be interpreted either as childhood or demographic variables: age/birth cohort, gender, and immigration status. Of these, higher E-SWB is associated with: older age, though levels are fairly high at 18–24, falling to their lowest at 40–49, then peaking at 80+; being female rather than male, especially relative to the small percentage reporting their gender as “other”; and living in one’s country of birth compared to those born elsewhere, although only marginally, and not for CL. Behind these headline summaries, each factor has a substantial literature which our findings support and/or refine in various ways. It is beyond our scope to consider this scholarship for all factors, so in each category we just delve briefly into the factor with the greatest variation to illustrate the contribution of our findings.

The demographic factor with the greatest variation was employment status. As such, our findings align with an extensive literature showing the impact of working patterns on wellbeing, with numerous reviews indicating that employment generally has a positive impact on E-SWB^38,74^. Notably though, the highest E-SWB was among retirees, who are technically “out of work,” which corroborates other studies finding a potential SWB boon to retirement, even if the association is complex^75^. There are of course interactions with age, given E-SWB generally increases into older age, as discussed below. Nevertheless, our data on employment suggests it is not necessarily lacking employment per se that is detrimental to E-SWB, but needing work yet failing to find it, with E-SWB relatively unaffected if people are materially secure and not wanting/needing work^76,77^.

Of the childhood factors, the one with the strongest association was self-rated health. Here we must emphasize that our findings rely on retrospective assessments, which are subject to recall bias (e.g., one analysis found nearly half their sample revised their ratings during a 10-year period^78^). Moreover, while we adjusted associations for other potential childhood factors, residual confounding may be present. However, we reported E-values^67^ to assess the robustness of our findings to unmeasured confounding, and the high values (e.g., 1.73 for “excellent health” and H) suggest the observed associations *are *relatively robust. Moreover, many longitudinal studies have assessed this association prospectively and show childhood health is associated with multiple aspects of adult life^79^, including E-SWB^80^. Despite the limitations of retrospective assessments, the observed patterns regarding self-reported health therefore likely point towards associations that are genuine.

Finally, of the three factors that could be interpreted as either demographic and/or childhood, the most prominent was age. In that regard, our results broadly support the relatively well-established view that E-SWB is somewhat “U-shaped,” declining into middle-age before rising again into older age^29^. The finding has generated lively debate though^81^. One must be careful of selection bias for example: a longitudinal analysis in the US suggested that LS actually declined after 65 (due to health issues and widowing), and that apparent higher LS in older age is due to those with high LS being more likely to participate in surveys. The pattern is also subject to cultural variation^82^, as discussed below. The metric also matters: an analysis of GWP data found that although CL showed a slight U-shaped pattern, positive affect decreased with age globally and across regions^28^. In our data though H aligned with CL and LS in rising into in older age, corroborating our suggestion that our H item does indeed index E-SWB. Notably though, rather than being U-shaped, our trends are more “J-shaped,” with lower E-SWB in younger compared to older participants (e.g., those 80+ had scores 0.5 higher than those 18–24). Even if selection bias applies to the over-80s or even over-70s, 18–24 year olds also fared worse than those 60–70, and even than those 50–59 on LS and H (albeit very slightly). One wonders whether this is a cohort effect, echoing recent research suggesting the left-hand side of the U is flattening lately (hence being a "J" shape) with younger people faring worse compared to people of similar age in earlier eras^81,83^. We also note though that our sample does not contain data on minors, and for a fuller understanding of these patterns such populations need to also be factored in, as indeed do any changes in these patterns over time (which is not possible with our cross-sectional analysis of Wave 1 GFS data here, but which future waves will allow).

### Contribution 3: examining granular cross-national differences

A third contribution is the study’s cross-national coverage. In itself this is not remarkable, with an extensive literature exploring international variation in E-SWB using datasets like the GWP. But its particular value here is in amplifying the first two contributions, permitting granular exploration of national variation regarding both the three outcomes and the 15 factors. In that respect, trends for the factors across the countries collectively, elucidated above, are not uniform but have striking nuances and exceptions, as detailed in the Supplementary tables, suggesting the trends are contingent on socio-cultural factors. Here we illustrate these nuances by revisiting the three factors highlighted above as having the strongest associations with E-SWB (employment, childhood health, and age).

Firstly though, let us emphasize that the data highlight the liabilities of generalizing from WEIRD to non-WEIRD places. A basic comparison of countries that could be classified as WEIRD vs. non-WEIRD (Table 14) shows the former score much higher on CL, while the levels are closer for H and essentially equal for LS. Moreover, part of our reservations with the WEIRD vs. non-WEIRD dichotomy is our data show considerable heterogeneity among countries commonly classed in either category, as exemplified by nations usually described as non-WEIRD being ranked top (Indonesia) and bottom (Turkey) for both LS and H. This heterogeneity is also shown by the differences between continents: Africa and Asia might both usually be classed as non-WEIRD, for example, but Asia fares better on CL and LS, while Africa does better on H. Such internal diversity highlights the limits of these labels, which obscure the complexity of international variation.

Turning to the three factors discussed above, there was considerable variation, with numerous countries bucking the overall terms. Regarding employment status, retirees were not the most prosperous everywhere, faring less well—on at least one E-SWB item, and sometimes all three—than people employed for an employer in 11 countries (Argentina, Egypt, Germany, Hong Kong, Indonesia, Israel, Kenya, Poland, South Africa, Spain, and Tanzania), and even worse than the unemployed in Kenya and Tanzania. Conversely, while the unemployed fared worst in most countries, exceptions—again on at least one item, and sometimes all three—included the employed in India, self-employed people in Egypt and Philippines, homemakers in Tanzania and South Africa, and “other” in nine places (Argentina, Australia, Hong Kong, Kenya, Nigeria, Philippines, Poland, Tanzania, and the UK). Other variation concerns the range of values. Comparing those unemployed, employed, and retirees, some countries had only narrow differences between these (especially Kenya, Egypt, and Philippines), while others had far larger ranges (notably Australia, Japan, Sweden, and the US), indicating stronger links between employment status and E-SWB among the latter.

There is also potential interaction with age in the association between employment status (particularly retirement) and E-SWB. While older age is generally associated with higher E-SWB, as noted above, regional variations make this relationship complex. No significant associations with age were observed for all E-SWB outcomes in Mexico and Spain, and only for some in Argentina, Indonesia, Nigeria, Philippines, Turkey, and South Africa. Further, some countries deviated from the J-shaped pattern, with linear decreases with age in Israel, Poland, and Tanzania. There was also variation in the range of scores within countries, further suggesting the relationship with age is conditioned by location, with the differences being smallest in Mexico and largest in Australia (where those 18–24 scored lowest and those 80+ the highest). Returning to the question of retirement, these findings highlight the complexity of the employment-SWB relationship, aligning with research indicating the associations are inconsistent^84^, and that factors such as economic resources^85^ and social relationships^86^ affect retirees’ E-SWB^87^.

Finally, self-rated childhood health also showed considerable variation. First, relative to the difference in outcomes comparing poor and excellent childhood health in the pooled meta-analysis, there was real variation in range, with Egypt the narrowest and Hong Kong the largest, indicating greater association between childhood health and adult E-SWB in the latter. Further research is needed to explain such regional variation, but it may involve factors like economic conditions, healthcare provisions, and levels of inequality. Childhood health might plausibly have a smaller impact on adult E-SWB in wealthier countries because they can invest more in healthcare to mitigate the adverse effects of poor childhood health^87^. GDP considerations alone though may be insufficient to explain regional differences, given that childhood health generates more variation in Hong Kong than Egypt (since the former is wealthier). It is likely therefore that factors such as socio-economic equality also contribute, and greater inequality may amplify the negative effects of poor childhood health, especially in countries without good universal healthcare coverage^88,89^. There were also intriguing patterns that are harder to explain and require further investigation too, above all that in some countries the effect estimates seemed “out of order.” Overall, relative to people with “good” childhood health, people with worse health had lower E-SWB while people with very good or excellent health had higher. Individually though only 13 countries conformed to this linear rising trend (Argentina, Australia, Brazil, Hong Kong, Indonesia, Japan, Kenya, Nigeria, Philippines, Sweden, Tanzania, UK, and US). In the remaining countries, this pattern was subverted in various ways. For example, people with poor health fared better—on at least one outcome, and sometimes all three—than those with good health in Germany, Israel, Mexico, Poland, and Spain. The reasons behind these findings are unclear. One possible explanation is that poor childhood health may encourage individuals to develop qualities that might contribute to E-SWB in adulthood, including psychological (e.g., resilience)^90^ and social resources (e.g., supportive childhood friends)^61^. However, why this association is found in certain countries and not others remains an open question.

### Limitations

This study has various limitations, including regarding: (1) the complexities of translation; (2) variability between assessments here and those in other surveys; (3) childhood experiences only being assessed retrospectively; (4) that our constructs were only assessed with single item measures; and (5) potential order and survey theme effects. Let’s briefly consider each in turn.

First, comparing and ranking countries can be problematic for various reasons^62^, perhaps above all the complexities of language. Translation is difficult, and it can be hard to find exact equivalents for terms across languages^91^. In empirical cross-country comparisons, cultural and linguistic nuances may thus influence results. While Gallup employs a well-established TRAPD (translation, review, adjudication, pretesting, and documentation) model^92^ to ensure accuracy, and the translation process for the GFS involved experts in relevant languages^40^, perfect equivalence cannot be guaranteed. Subtle differences may mean terms assess slightly different outcomes across languages, a limitation requiring further qualitative investigation to fully ascertain. Given this and other considerations noted below, the primary goal of the GFS was not direct cross-cultural comparison per se but separate within-country analyses of 22 closely related cohort studies, followed by meta-analyses across countries. This approach does not assume items are interpreted identically across countries but are relatively closely related (just as a meta-analysis of similar interventions may differ in specific administration, dose, etc.). While cross-country comparisons are possible, they should be interpreted with caution.

Other factors also influence assessment across countries^93^, including cultural norms, item interpretation, response scales, sample characteristics, and seasonal variations from different data collection times. For instance, comparing our CL data (gathered mostly in 2023) with the 2024 WHR^18^, which aggregates GWP data from 2021 to 2023 (see Table S25a), we observe some consistency in certain countries (e.g., Germany scored 6.74 in our data and 6.72 in the WHR). Notable discrepancies are seen elsewhere however, with differences greater than 1.00 in several (e.g., Hong Kong scored 6.85 in our data, but 5.32 in the WHR). These discrepancies may result from parameters including sampling timeframes, participant selection, and modes of interviews (e.g., web vs. phone). Regarding data collection windows, for example, the GFS is broader (up to 18 months in Australia), while the GWP is generally only 3–4 months. Seasonal patterns or local socio-political events could thus differentially affect GFS and GWP data. The 2023 GWP data for Israel, for example, was collected after the traumatic October 7th attacks (10.17.23–12.2.23), whereas most GFS data was before (11.7.22–11.23.23), resulting in less influence from the event and its aftermath. Even without such impactful situations, within-country CL can fluctuate significantly. While the GWP is an annual snapshot, Gallup also collects CL data monthly in the US, with considerable variation sometimes within a single year^94^. Another factor is participant selection. The GWP is a one-off survey, whereas the GFS is a five-year (minimum) longitudinal study, and conceivably those willing to commit to a long-term survey may have higher CL scores than one-off participants. This explanation is supported by CL scores in the GFS being generally higher than those in the GWP (with a few exceptions). Using 2023 GWP data, the CL mean across all 142 GWP countries is 5.60, whereas the GWP mean across the 22 GFS countries is 5.91, while the GFS overall mean itself is 6.34. There is thus a substantial standardized mean difference between the GFS and GWP (Table S25b), calculated as either 0.29 (if including all GWP countries) or 0.17 (if only including GFS countries in the GWP). GFS countries may also have higher CL scores due to being on average more prosperous, since unlike the GWP the GFS has no low-income countries (only lower-middle-, upper-middle-, and high-income ones). For all these reasons, comparing *across* datasets can be problematic, meaning one cannot definitively determine *the* E-SWB of a given nation.

Third, while we constructed a synthetic longitudinal study by retrospectively assessing childhood experiences, our cross-sectional design here partially limits definitive conclusions about causality. Such synthetic longitudinal designs may also be subject to recall bias. However, for recall bias to completely explain away the observed associations would require the effect of current E-SWB on biasing retrospective assessments of childhood factors to be at least as strong as the observed associations themselves^95^, and some were quite substantial. Moreover, although we adjusted associations for these childhood factors for other potential childhood factors, residual confounding may still be present. We reported E-values for the childhood analysis to assess the robustness of findings to unmeasured confounding for each factor and some of these associations were at least moderately robust. Moreover, some factors may lie on the causal pathway linking others to adult E-SWB. Adjusting for such mediators may lead to conservative estimates of the associations. Penultimately, our three E-SWB constructs were assessed using one-item measures, which likely does not fully capture their full complexity. Future studies could use multi-item measures for higher validity and reliability. There are always trade-offs though in survey research between depth and breadth. Using multi-item scales limits the number of constructs assessed, and the GFS team decided that, overall from the perspective of the GFS as a whole, the benefits of including more constructs outweighed the limitations of single-item measures^59^.

Finally, the three outcomes are situated close together in the annual GFS questionnaire as the first (CL), third (H), and fourth (LS) items (with the second item being anticipated CL in five years). Such proximity potentially inflates the differences between the items, given that asking similar questions in sequence can make participants think they are supposed to give different answers^96^. That said, there was tighter correlation between the consecutive items (H-LS: 0.69) than those separated by an item (CL-H: 0.57), which perhaps argues against that critique. But even if the critique has merit, the answers are still informative, even if one must interpret them with caution, since if someone *does* feel compelled to give a different answer, the *direction *they do so is meaningful. To return to Indonesia versus Sweden, for instance, having first given their CL score, people in the former tend to give higher scores for LS and H, whereas people in the latter give lower scores. Even if people in Indonesia may not have CL levels “1” lower than LS/H, and people in Sweden “1” higher—whatever increment “1” might mean—it seems reasonable to conclude people in Indonesia do genuinely have lower CL than LS/H, and vice versa for Sweden. It has also already been established that the overall theme of a survey can affect not just E-SWB levels but also the relative importance given to different variables used to explain them^70^. The focus of the GFS may help account for some of the differences found between the results above and those in other surveys. This consideration gives us one more reason for needing further research comparing alternative measures in a range of different survey contexts.

## Conclusion

The value of measuring E-SWB is increasingly recognized, including by governmental organizations that recommend its utility in guiding policy. The literature is constrained by various issues however, to which this study makes three key interrelated contributions, as summarized here through the lens of potential practical implications.

The first contribution is directly comparing three candidates for indexing E-SWB. Most striking were their associations with flourishing, with LS and H having stronger correlations across all domains of VanderWeele’s framework (not counting the first domain, as this is constituted by LS and H themselves), while CL only attained relative parity on the additional domain of financial/material security. Given that stakeholders (e.g., policy makers) may look to articles like this for guidance on which measures to use and how to interpret them, here are some tentative recommendations. Overall, if choosing just one item, we suggest LS, given its strong correlations with the flourishing domains. This is preferred to H, despite the latter having similarly strong correlations, mainly because of the ambiguity still attached to H. Although our data suggests it can indeed function as an evaluative item, it does nevertheless have a double meaning, and some respondents may still interpret it in affective terms, so we cannot be *certain* it indexes E-SWB (despite seeming to here). Then, if space allows for another item, we recommend CL, mainly because H tracks LS closely, whereas CL seemingly captures different experiential terrain, particularly around financial/material stability, so is a useful complement/counterpart to LS.

A second contribution is an expansive coverage of 15 socio-demographic and childhood factors, and while all were significantly associated with E-SWB, considering them together shows their relative strength of association. Regarding practical implications, our analysis highlights people who might be particularly at risk of low E-SWB, indicating where policy/intervention efforts to improve E-SWB may most effectively be targeted. Demographically our data suggests E-SWB is liable to be lowest in people who are: unemployed and looking for a job; separated; not attendees of religious services; of less than eight years education; 40–49; male (or especially “other” gender); and an immigrant. It would also likely be lower for people whose childhood was characterized by: poor health; very difficult financial situations; abuse; feeling like an outsider; never attending religious services; poor relationships with one’s mother and/or father; and parents who were single and never married. Moreover, while each factor itself is meaningful, from an intersectional perspective^97^, the more categories someone belongs to, the lower their E-SWB is likely to be. The findings thus suggest that targeted interventions, such as employment support, could enhance E-SWB, especially for people facing multiple vulnerabilities. While not all factors are amenable to intervention/policy, focusing on those that are may provide meaningful improvements, particularly for people most in need. Most analyses of E-SWB-related factors show steeper improvement trajectories for those worse off, as for example with income satiation^28^, where the impact of income gains on E-SWB diminishes the richer people are. While one would want to improve E-SWB for all, it may be more effective, and more morally worthy, to prioritize helping those with the lowest E-SWB. This must be done sensitively though, avoiding both stigmatizing populations with the lowest E-SWB and making everyone else feel they are being treated unfairly.

Finally, the third contribution is our cross-national coverage, which although not novel itself accentuates the first two contributions, permitting granular exploration of national variation regarding both the three outcomes and the 15 factors, which is relevant to the conclusions above. Regarding the implications of our analysis of the factors, for instance, policy interventions require accounting for local dynamics (e.g., which people particularly need help). In that respect, while it is customary for papers to plead for more research, this concern is especially justified regarding the cross-cultural implications of this study. There is particular need, for instance, for utilizing other methodologies, especially qualitative techniques, to delve into the first and third issues (the meaning of the items and cross-cultural variation in these meanings). Above we cited for example a recent analysis of CL which suggests the metaphor encourages people to think of power, achievement, and success^70^. That study was only in English and on just one measure though. It would thus be instructive to conduct comparable analyses in other languages and on the other E-SWB constructs, thereby helping us further understand this vitally important topic.

## Supplementary Information

Below is the link to the electronic supplementary material.

Supplementary Material 1

## Data Availability

Data that support the findings of this article are openly available on the Open Science Framework (Wave 1 non-sensitive Global data: https://osf.io/sm4cd/), and are available from February 2024 - March 2026 via preregistration and publicly from then onwards. Please see https://www.cos.io/gfs-access-data for more information about data access.

## Code availability

Code in multiple software is openly available in an online repository^49^ for the demographic and childhood analyses (https://doi.org/10.17605/osf.io/vbype).
