# Supplementary material for: Exploring associations of three evaluative subjective wellbeing measures (Cantril’s ladder, life satisfaction, happiness) with 15 childhood and demographic factors across 22 countries
Source: Sci Rep. 2026 Feb 10;16:8025. doi: 10.1038/s41598-026-35777-y (PMC12957326; doi:10.1038/s41598-026-35777-y)
Supplement: Supplementary file 1 — Supplementary Material 1 [file 41598_2026_35777_MOESM1_ESM.pdf]

Online Supplement

Contents

Pages	Content
2—2	Notes, caveats, and interpretation tips
3—8	Tables S1a-g: Main table versions based on complete case analysis
9—28	Table S2a-g. Country specific results for Argentina
29—52	Table S3a-g. Country specific results for Australia
53—72	Table S4a-g. Country specific results for Brazil
73—92	Table S5a-g. Country specific results for Egypt
93—112	Table S6a-g. Country specific results for Germany
113—136	Table S7a-g. Country specific results for Hong Kong (S.A.R. of China)
137—156	Table S8a-g. Country specific results for India
157—176	Table S9a-g. Country specific results for Indonesia
177—196	Table S10a-g. Country specific results for Israel
197—216	Table S11a-g. Country specific results for Japan
217—236	Table S12a-g. Country specific results for Kenya
237—256	Table S13a-g. Country specific results for Mexico
257—276	Table S14a-g. Country specific results for Nigeria
277—298	Table S15a-g. Country specific results for the Philippines
299—218	Table S16a-g. Country specific results for Poland
319—338	Table S17a-g. Country specific results for South Africa
339—358	Table S18a-g. Country specific results for Spain
359—378	Table S19a-g. Country specific results for Sweden
379—398	Table S20a-g. Country specific results for Tanzania
399—418	Table S21a-g. Country specific results for Turkey
419—438	Table S22a-g. Country specific results for the United Kingdom
439—458	Table S23a-g. Country specific results for the United States
459—465	Tables S24a-c. Population weighted meta-analysis results
466—468	Table S25a-b. CL data in the GFS, GWP, and WHR
469—471	Tables S26a-c. SWB and employment status across countries
472—474	Tables S27a-c. SWB and childhood health across countries
475—477	Figures 28a-f. Scatter plots for GDP per capita and Gini Index
478—497	Figures 29(1-102). Forest plots for demographic variation analyses
494—495	Figures 30(1-81). Forest plots for childhood predictor analyses
508—509	Figures 31a-c. Pairwise standardized mean differences in country means

Important Notes and Caveats

This online supplement to the Global Flourishing Study paper on Cantril's Ladder, life satisfaction, and happiness has several important caveats to interpretation for the demographic variation analyses and childhood predictor analyses.

For the demographic variation analyses, estimating the within country group means can be unstable when the group size is small (<1%) of the country sample size. In such cases, the uncertainty in the estimate leads to a multiple imputation adjusted degrees of freedom less than 1. This means there is not enough information to evaluate the uncertainty in the estimate. We flagged such cases with a "*". Interval estimates for the mean of continuous outcomes (ranged 0-10) were based on a Wald-type confidence interval where items were treated as continuous which could rarely lead to intervals exceeding the bounds of the observed range of values; in such cases we have truncate the limits to be within the range of observed values, and such cases are marked with a "ǂ".

For the childhood predictor analyses, analyses are based on data using a retrospective (recall) approach to obtain information about the respondents' lives at age 12. Several the childhood characteristics such as relationship quality with parents, subjective financial status growing up, etc. are necessarily highly related. This led to multicollinearity issues so some effects may be unstable. Additionally, in rare instances the confidence interval of the effect estimate can contradict the reported global p-value (e.g., for the single-category effects of relationship with mother). In such cases, the reported confidence interval is more robust with corrected degrees of freedom from the pooling across multiple imputations, whereas the global p-value is based on a Wald-type test and is less robust to uncertainty attributable to multiple imputation.

Comparing results across countries should be done with caution due to possible measurement non-invariance and differences in translation.

Tables S1a-g: Main table versions based on complete case analysis

Table S1a. Comparison of complete case estimates of means of each outcome by country and rate of missing by country

		Cantril's Ladder		Life Satisfaction		Happiness	
		Mean (SE) [SD]	(Missing)	Mean (SE) [SD]	(Missing)	Mean (SE) [SD]	(Missing)
Overall	N = 202,898[1]	6.4171 (0.0096) [2.4633]	516 (0.3%)	6.8251 (0.0102) [2.5994]	694 (0.3%)	6.9883 (0.0090) [2.3761]	399 (0.2%)
Argentina	N = 6,724[1]	6.7477 (0.0398) [2.1851]	7 (0.1%)	7.2153 (0.0429) [2.3766]	18 (0.3%)	7.3599 (0.0388) [2.1405]	17 (0.3%)
Australia	N = 3,844[1]	6.7846 (0.0372) [1.7743]	3 (<0.1%)	6.7188 (0.0450) [2.1047]	8 (0.2%)	6.8827 (0.0387) [1.8179]	5 (0.1%)
Brazil	N = 13,204[1]	6.5909 (0.0272) [2.2820]	18 (0.1%)	7.1529 (0.0288) [2.4763]	33 (0.3%)	7.3342 (0.0267) [2.3010]	37 (0.3%)
Egypt	N = 4,729[1]	5.0435 (0.0554) [2.8661]	33 (0.7%)	7.6869 (0.0548) [2.8347]	28 (0.6%)	6.1783 (0.0656) [2.9426]	19 (0.4%)
Germany	N = 9,506[1]	6.7385 (0.0236) [1.8187]	8 (<0.1%)	6.9289 (0.0271) [2.0805]	32 (0.3%)	6.9023 (0.0250) [1.9147]	14 (0.1%)
Hong Kong	N = 3,012[1]	6.8459 (0.0488) [2.0206]	1 (<0.1%)	7.0252 (0.0506) [2.0844]	51 (1.7%)	7.1630 (0.0470) [1.9970]	5 (0.2%)
India	N = 12,765[1]	5.6256 (0.0466) [3.5810]	133 (1.0%)	7.0034 (0.0445) [3.4670]	59 (0.5%)	6.4764 (0.0460) [3.5587]	38 (0.3%)
Indonesia	N = 6,992[1]	6.9690 (0.0479) [2.5242]	38 (0.5%)	7.9907 (0.0414) [2.2888]	19 (0.3%)	8.0423 (0.0369) [2.1951]	23 (0.3%)
Israel	N = 3,669[1]	7.3308 (0.0762) [1.8109]	3 (<0.1%)	7.4734 (0.0856) [2.0001]	10 (0.3%)	7.7605 (0.0622) [1.6516]	4 (<0.1%)
Japan	N = 20,543[1]	5.9241 (0.0172) [2.1438]	79 (0.4%)	6.0567 (0.0185) [2.3086]	120 (0.6%)	6.2176 (0.0171) [2.1335]	76 (0.4%)
Kenya	N = 11,389[1]	5.5096 (0.0498) [3.2760]	18 (0.2%)	5.9678 (0.0496) [3.5116]	14 (0.1%)	7.2740 (0.0430) [2.9643]	12 (0.1%)
Mexico	N = 5,776[1]	7.1018 (0.0345) [2.1147]	12 (0.2%)	7.8497 (0.0369) [2.1389]	15 (0.3%)	7.7862 (0.0340) [2.0297]	19 (0.3%)
Nigeria	N = 6,827[1]	5.7275 (0.0631) [2.7888]	19 (0.3%)	6.5096 (0.0649) [2.8611]	16 (0.2%)	7.0633 (0.0559) [2.5879]	12 (0.2%)
Philippines	N = 5,292[1]	6.3843 (0.0406) [2.3958]	4 (<0.1%)	7.5011 (0.0440) [2.4277]	5 (0.1%)	7.3259 (0.0421) [2.3335]	3 (<0.1%)
Poland	N = 10,389[1]	7.1220 (0.0423) [1.6153]	24 (0.2%)	7.5240 (0.0506) [1.7300]	110 (1.1%)	7.5535 (0.0467) [1.5957]	15 (0.1%)
South Africa	N = 2,651[1]	6.1068 (0.0935) [2.8385]	6 (0.2%)	6.3620 (0.0807) [2.8117]	10 (0.4%)	6.9547 (0.0801) [2.6526]	1 (<0.1%)
Spain	N = 6,290[1]	6.6661 (0.0323) [1.9256]	7 (0.1%)	6.7882 (0.0366) [2.1770]	16 (0.3%)	6.9214 (0.0331) [2.0032]	16 (0.3%)
Sweden	N = 15,068[1]	7.1979 (0.0171) [1.7565]	45 (0.3%)	7.0925 (0.0201) [2.0703]	34 (0.2%)	7.0316 (0.0192) [1.9620]	38 (0.3%)
Tanzania	N = 9,075[1]	4.4015 (0.0829) [3.3032]	17 (0.2%)	5.3339 (0.0846) [3.7732]	10 (0.1%)	6.5767 (0.0607) [3.2633]	13 (0.1%)
Turkey	N = 1,473[1]	5.1803 (0.0856) [2.5627]	3 (0.2%)	5.1877 (0.1083) [3.2408]	5 (0.4%)	5.5395 (0.0981) [2.9330]	1 (<0.1%)
United Kingdom	N = 5,368[1]	6.5561 (0.0392) [2.0059]	15 (0.3%)	6.4908 (0.0456) [2.3075]	38 (0.7%)	6.7061 (0.0405) [2.0832]	11 (0.2%)
United States	N = 38,312[1]	6.9361 (0.0261) [1.8543]	21 (<0.1%)	6.8552 (0.0298) [2.1422]	41 (0.1%)	7.0094 (0.0265) [1.9209]	20 (<0.1%)

Table S1b. Random effects meta-analyzed demographic variation for Cantril's Ladder based on complete case data

Variable	Category	Est	95% CI	SE	Prediction Interval LL	Prediction Interval UL	Heterogeneity (τ)	I^2	Global p-value
Age group									<.001**
	18-24	6.52	(6.29,6.76)	0.12	5.59	7.73	0.55	97.5	
	25-29	6.52	(6.29,6.76)	0.12	5.59	7.58	0.55	97.1	
	30-39	6.47	(6.21,6.72)	0.13	5.30	7.49	0.61	98.8	
	40-49	6.46	(6.20,6.71)	0.13	5.29	7.35	0.61	98.6	
	50-59	6.61	(6.37,6.84)	0.12	5.30	7.30	0.55	98.3	
	60-69	6.74	(6.48,6.99)	0.13	5.32	7.60	0.59	98.3	
	70-79	6.88	(6.64,7.13)	0.12	5.68	7.88	0.52	96.9	
	80 or older	7.14	(6.89,7.40)	0.13	6.33	7.98	0.45	80.4	
Gender									<.001**
	Male	6.55	(6.29,6.81)	0.13	5.27	7.51	0.62	99.4	
	Female	6.63	(6.42,6.84)	0.11	5.62	7.29	0.50	99.2	
	Other	6.16	(5.74,6.57)	0.21	4.93	7.53	0.63	66.5	
Marital status									<.001**
	Married	6.78	(6.50,7.06)	0.14	5.47	7.73	0.67	99.6	
	Separated	6.21	(5.98,6.45)	0.12	5.10	6.94	0.49	86.5	
	Divorced	6.28	(6.00,6.57)	0.15	5.02	7.22	0.62	96.2	
	Widowed	6.71	(6.47,6.94)	0.12	5.60	7.52	0.52	92.3	
	Domestic partner	6.54	(6.34,6.75)	0.10	5.62	7.16	0.42	95.4	
	Single, never married	6.35	(6.11,6.58)	0.12	5.27	7.50	0.56	98.8	
Employment status									<.001**
	Employed for an employer	6.60	(6.34,6.85)	0.13	5.35	7.47	0.60	99.4	
	Self-employed	6.65	(6.36,6.94)	0.15	5.31	7.77	0.68	98.7	
	Retired	6.80	(6.53,7.07)	0.14	5.19	7.72	0.62	98.5	
	Student	6.53	(6.30,6.77)	0.12	5.44	7.94	0.54	95.4	
	Homemaker	6.55	(6.35,6.75)	0.10	5.59	7.27	0.46	95.0	
	Unemployed and looking for a job	5.97	(5.74,6.21)	0.12	4.71	6.76	0.53	94.9	
	None of these/other	6.32	(6.04,6.59)	0.14	5.13	7.64	0.59	93.0	
Education									<.001**
	Up to 8 years	6.54	(6.29,6.79)	0.13	5.43	7.38	0.58	97.1	
	9-15 years	6.52	(6.29,6.75)	0.12	5.42	7.44	0.55	99.5	
	16+ years	6.73	(6.48,6.99)	0.13	5.40	7.42	0.60	99.3	
Religious service attendance									<.001**
	>1/week	7.06	(6.70,7.42)	0.18	5.42	9.02	0.85	98.6	
	1/week	6.82	(6.56,7.09)	0.13	5.46	7.54	0.62	98.3	
	1-3/month	6.65	(6.39,6.90)	0.13	5.43	7.29	0.59	96.9	
	A few times a year	6.53	(6.27,6.78)	0.13	5.27	7.47	0.60	98.9	
	Never	6.42	(6.19,6.66)	0.12	4.91	7.16	0.54	99.2	
Immigration status									<.001**
	Born in another country	6.58	(6.34,6.83)	0.12	5.45	7.15	0.52	93.2	
	Born in this country	6.59	(6.36,6.83)	0.12	5.45	7.49	0.56	99.6	

Table S1c. Random effects meta-analyzed demographic variation for life satisfaction based on complete case data

Variable	Category	Est	95% CI	SE	Prediction Interval LL	Prediction Interval UL	Heterogeneity (τ)	I^2	Global p-value
Age group									<.001**
	18-24	7.06	(6.74,7.39)	0.17	5.61	8.24	0.77	98.6	
	25-29	7.06	(6.75,7.36)	0.15	5.71	8.28	0.72	98.2	
	30-39	7.05	(6.77,7.32)	0.14	5.92	8.28	0.65	98.8	
	40-49	7.05	(6.80,7.30)	0.13	5.85	8.15	0.60	98.4	
	50-59	7.18	(6.95,7.40)	0.12	5.96	8.10	0.53	98.1	
	60-69	7.36	(7.14,7.57)	0.11	6.49	8.27	0.49	97.4	
	70-79	7.49	(7.28,7.71)	0.11	6.93	8.51	0.45	95.8	
	80 or older	7.54	(7.17,7.92)	0.19	4.80	8.75	0.82	93.7	
Gender									<.001**
	Male	7.15	(6.91,7.39)	0.12	5.95	8.11	0.57	99.3	
	Female	7.21	(6.98,7.44)	0.12	6.19	8.22	0.54	99.3	
	Other	6.14	(5.84,6.45)	0.16	5.70	6.90	0.35	38.5	
Marital status									<.001**
	Married	7.40	(7.19,7.62)	0.11	6.23	8.24	0.52	99.3	
	Separated	6.75	(6.48,7.01)	0.14	5.72	7.82	0.56	88.3	
	Divorced	6.84	(6.59,7.09)	0.13	5.96	7.89	0.54	94.3	
	Widowed	7.36	(7.15,7.57)	0.11	6.66	8.20	0.46	90.5	
	Domestic partner	7.02	(6.76,7.27)	0.13	6.14	8.04	0.54	96.7	
	Single, never married	6.87	(6.54,7.21)	0.17	5.47	8.03	0.79	99.3	
Employment status									<.001**
	Employed for an employer	7.15	(6.92,7.38)	0.12	6.03	7.97	0.54	99.2	
	Self-employed	7.25	(7.01,7.49)	0.12	6.12	8.24	0.57	98.0	
	Retired	7.41	(7.18,7.63)	0.11	6.49	8.40	0.51	97.6	
	Student	7.04	(6.71,7.37)	0.17	5.37	8.29	0.77	97.3	
	Homemaker	7.26	(7.02,7.51)	0.12	6.47	8.31	0.56	96.9	
	Unemployed and looking for a job	6.53	(6.16,6.89)	0.19	5.00	7.82	0.85	97.8	
	None of these/other	6.85	(6.44,7.25)	0.21	5.20	8.50	0.93	96.8	
Education									<.001**
	Up to 8 years	7.26	(7.00,7.51)	0.13	5.76	8.27	0.59	97.5	
	9-15 years	7.13	(6.88,7.38)	0.13	5.94	8.11	0.59	99.5	
	16+ years	7.18	(6.93,7.42)	0.13	5.88	8.03	0.58	99.1	
Religious service attendance									<.001**
	>1/week	7.72	(7.47,7.97)	0.13	6.61	8.83	0.59	97.4	
	1/week	7.45	(7.26,7.64)	0.10	6.51	8.20	0.44	96.7	
	1-3/month	7.21	(6.99,7.44)	0.12	6.04	8.12	0.53	96.3	
	A few times a year	7.06	(6.81,7.31)	0.13	5.44	7.96	0.59	98.7	
	Never	6.94	(6.69,7.19)	0.13	5.46	8.11	0.58	99.1	
Immigration status									<.001**
	Born in another country	7.08	(6.90,7.25)	0.09	6.43	7.87	0.33	83.4	
	Born in this country	7.18	(6.94,7.41)	0.12	6.04	8.17	0.56	99.6	

Table S1d. Random effects meta-analyzed demographic variation for happiness based on complete case data

Variable	Category	Est	95% CI	SE	Prediction Interval LL	Prediction Interval UL	Heterogeneity (τ)	I^2	Global p-value
Age group									<.001**
	18-24	7.12	(6.82,7.42)	0.15	5.83	8.11	0.71	98.6	
	25-29	7.14	(6.88,7.40)	0.13	6.06	8.28	0.61	97.8	
	30-39	7.11	(6.87,7.36)	0.13	6.02	8.19	0.58	98.7	
	40-49	7.09	(6.85,7.33)	0.12	5.94	8.26	0.57	98.4	
	50-59	7.20	(6.99,7.41)	0.11	6.05	8.01	0.49	97.9	
	60-69	7.33	(7.16,7.51)	0.09	6.54	8.15	0.40	96.7	
	70-79	7.50	(7.34,7.66)	0.08	6.82	8.01	0.32	92.9	
	80 or older	7.54	(7.27,7.82)	0.14	5.86	8.34	0.54	87.9	
Gender									<.001**
	Male	7.19	(6.97,7.41)	0.11	6.00	8.07	0.52	99.2	
	Female	7.24	(7.04,7.44)	0.10	6.26	8.27	0.48	99.2	
	Other	6.10	(5.69,6.51)	0.21	5.23	7.38	0.59	60.8	
Marital status									<.001**
	Married	7.43	(7.23,7.63)	0.10	6.27	8.24	0.47	99.2	
	Separated	6.84	(6.59,7.09)	0.13	5.96	7.71	0.53	88.8	
	Divorced	6.89	(6.66,7.13)	0.12	5.99	7.72	0.49	94.3	
	Widowed	7.22	(7.04,7.41)	0.10	6.61	8.06	0.40	88.7	
	Domestic partner	7.16	(6.90,7.42)	0.13	6.27	8.44	0.55	97.2	
	Single, never married	6.94	(6.64,7.23)	0.15	5.59	7.99	0.71	99.3	
Employment status									<.001**
	Employed for an employer	7.21	(7.00,7.43)	0.11	6.17	8.03	0.50	99.1	
	Self-employed	7.30	(7.09,7.50)	0.11	6.42	8.18	0.48	97.6	
	Retired	7.38	(7.18,7.58)	0.10	6.39	8.15	0.45	97.2	
	Student	7.06	(6.75,7.36)	0.16	5.66	8.27	0.71	97.4	
	Homemaker	7.25	(7.03,7.47)	0.11	6.31	8.38	0.51	96.4	
	Unemployed and looking for a job	6.63	(6.27,6.99)	0.18	5.15	7.82	0.84	98.0	
	None of these/other	6.95	(6.61,7.29)	0.17	5.44	8.17	0.76	95.8	
Education									<.001**
	Up to 8 years	7.25	(7.00,7.50)	0.13	5.89	8.31	0.59	97.7	
	9-15 years	7.17	(6.96,7.39)	0.11	6.05	8.07	0.51	99.4	
	16+ years	7.24	(7.05,7.44)	0.10	6.02	7.95	0.45	98.7	
Religious service attendance									<.001**
	>1/week	7.75	(7.49,8.01)	0.13	6.62	9.22	0.61	97.8	
	1/week	7.49	(7.31,7.67)	0.09	6.45	8.15	0.41	96.8	
	1-3/month	7.27	(7.08,7.46)	0.10	6.29	8.06	0.44	95.4	
	A few times a year	7.10	(6.89,7.31)	0.11	5.59	7.88	0.49	98.3	
	Never	6.97	(6.75,7.20)	0.11	5.55	8.11	0.52	99.1	
Immigration status									<.001**
	Born in another country	7.08	(6.84,7.32)	0.12	6.27	8.08	0.50	92.9	
	Born in this country	7.22	(7.01,7.43)	0.11	6.12	8.17	0.50	99.6	

Table S1e. Random effects meta-analyzed childhood predictor results for Cantril's Ladder based on complete case data

Variable	Category	Est	95% CI	SE	Estimated Proportion of Est < -0.10	Estimated Proportion of Est > 0.10	Heterogeneity (τ)	I^2	Global p-value
Relationship with mother	(Ref: Very bad/somewhat bad)								0.004*
	Very good/somewhat good	0.10	(0.05,0.16)	0.03	0.00	0.50	0.04	8.4	
Relationship with father	(Ref: Very bad/somewhat bad)								<.001**
	Very good/somewhat good	0.17	(0.11,0.22)	0.03	0.00	0.73	0.07	32.8	
Parent marital status	(Ref: Parents married)								0.035*
	No, one or both of them had died	-0.05	(-0.13,0.03)	0.04	0.00	0.00	<.01ǂ	<0.1ǂ	
	Single, never married	-0.08	(-0.20,0.05)	0.06	0.50	0.23	0.19	58.6	
	Yes, married	0.02	(-0.05,0.09)	0.03	0.09	0.27	0.09	39.7	
Subjective financial status of family growing up	(Ref: Got by)								<.001**
	Lived comfortably	0.29	(0.21,0.38)	0.04	0.00	0.86	0.19	88.6	
	Found it difficult	-0.13	(-0.20,-0.07)	0.03	0.64	0.05	0.11	61.3	
	Found it very difficult	-0.29	(-0.42,-0.15)	0.07	0.77	0.05	0.27	71.2	
Abuse	(Ref: No)								<.001**
	Yes	-0.21	(-0.32,-0.11)	0.05	0.86	0.10	0.21	82.3	
Outsider growing up	(Ref: No)								<.001**
	Yes	-0.10	(-0.19,-0.01)	0.05	0.41	0.09	0.17	74.4	
Self-rated health growing up	(Ref: Good)								<.001**
	Excellent	0.41	(0.25,0.57)	0.08	0.05	0.91	0.37	94.4	
	Very good	0.24	(0.15,0.32)	0.04	0.00	0.82	0.17	81.1	
	Fair	-0.22	(-0.29,-0.14)	0.04	0.77	0.00	0.11	47.7	
	Poor	-0.20	(-0.36,-0.03)	0.08	0.59	0.14	0.28	57.1	
Immigration status	(Ref: Born in this country)								<.001**
	Born in another country	0.07	(-0.03,0.18)	0.05	0.18	0.27	0.15	51.3	
Age 12 religious service attendance	(Ref: Never)								<.001**
	At least 1/week	0.20	(0.13,0.27)	0.04	0.00	0.82	0.11	47.6	
	1-3/month	0.20	(0.11,0.29)	0.04	0.00	0.73	0.15	65.2	
	Less than 1/month	0.08	(0.05,0.12)	0.02	0.00	0.00	<.01ǂ	<0.1ǂ	
Year of birth	(Ref: 1998-2005; age 18-24)								<.001**
	1993-1998; age 25-29	0.02	(-0.05,0.08)	0.03	0.09	0.23	0.09	38.2	
	1983-1993; age 30-39	-0.01	(-0.11,0.10)	0.05	0.36	0.27	0.22	81.8	
	1973-1983; age 40-49	-0.03	(-0.15,0.10)	0.07	0.36	0.41	0.28	86.1	
	1963-1973; age 50-59	0.11	(-0.02,0.24)	0.07	0.23	0.55	0.27	84.1	
	1953-1963; age 60-69	0.25	(0.07,0.43)	0.09	0.23	0.55	0.39	89.4	
	1943-1953; age 70-79	0.33	(0.09,0.57)	0.12	0.14	0.64	0.51	90.6	
	1943 or earlier; age 80+	0.36	(0.02,0.70)	0.17	0.18	0.73	0.68	88.0	
Gender	(Ref: Male)								<.001**
	Female	-0.12	(-0.21,-0.04)	0.04	0.55	0.09	0.18	90.6	
	Other	-0.14	(-0.71,0.43)	0.29	0.61	0.33	1.09	92.1	

Table S1f. Random effects meta-analyzed childhood predictor results for life satisfaction based on complete case data

Variable	Category	Est	95% CI	SE	Estimated Proportion of Est < -0.10	Estimated Proportion of Est > 0.10	Heterogeneity (τ)	I^2	Global p-value
Relationship with mother	(Ref: Very bad/somewhat bad)								0.076
	Very good/somewhat good	0.11	(0.06,0.16)	0.03	0.00	0.73	0.02	1.6	
Relationship with father	(Ref: Very bad/somewhat bad)								<.001**
	Very good/somewhat good	0.17	(0.10,0.24)	0.04	0.00	0.68	0.10	45.8	
Parent marital status	(Ref: Parents married)								<.001**
	No, one or both of them had died	0.03	(-0.10,0.15)	0.06	0.23	0.32	0.20	48.3	
	Single, never married	-0.02	(-0.18,0.14)	0.08	0.45	0.32	0.30	75.9	
	Yes, married	0.06	(-0.04,0.17)	0.05	0.14	0.41	0.19	71.2	
Subjective financial status of family growing up	(Ref: Got by)								<.001**
	Lived comfortably	0.26	(0.18,0.33)	0.04	0.00	0.86	0.17	84.9	
	Found it difficult	-0.07	(-0.14,-0.01)	0.03	0.45	0.09	0.11	59.4	
	Found it very difficult	-0.14	(-0.25,-0.02)	0.06	0.59	0.14	0.20	58.1	
Abuse	(Ref: No)								<.001**
	Yes	-0.29	(-0.38,-0.19)	0.05	0.86	0.05	0.18	75.5	
Outsider growing up	(Ref: No)								<.001**
	Yes	-0.20	(-0.29,-0.11)	0.05	0.73	0.05	0.18	73.5	
Self-rated health growing up	(Ref: Good)								<.001**
	Excellent	0.47	(0.30,0.64)	0.09	0.00	0.91	0.38	94.4	
	Very good	0.24	(0.14,0.33)	0.05	0.05	0.68	0.20	84.4	
	Fair	-0.26	(-0.33,-0.19)	0.03	1.00	0.00	0.09	32.7	
	Poor	-0.20	(-0.37,-0.04)	0.08	0.68	0.09	0.27	53.2	
Immigration status	(Ref: Born in this country)								<.001**
	Born in another country	0.06	(-0.10,0.23)	0.08	0.27	0.41	0.30	78.8	
Age 12 religious service attendance	(Ref: Never)								<.001**
	At least 1/week	0.19	(0.05,0.33)	0.07	0.23	0.73	0.29	85.3	
	1-3/month	0.13	(0.02,0.25)	0.06	0.23	0.55	0.22	76.1	
	Less than 1/month	0.05	(-0.04,0.13)	0.04	0.09	0.32	0.15	70.6	
Year of birth	(Ref: 1998-2005; age 18-24)								<.001**
	1993-1998; age 25-29	0.01	(-0.08,0.10)	0.05	0.32	0.36	0.17	64.0	
	1983-1993; age 30-39	0.02	(-0.12,0.15)	0.07	0.27	0.45	0.30	88.2	
	1973-1983; age 40-49	0.01	(-0.16,0.19)	0.09	0.36	0.50	0.40	91.9	
	1963-1973; age 50-59	0.13	(-0.08,0.33)	0.11	0.32	0.59	0.47	93.6	
	1953-1963; age 60-69	0.28	(0.06,0.50)	0.11	0.23	0.68	0.49	92.4	
	1943-1953; age 70-79	0.40	(0.11,0.68)	0.15	0.32	0.64	0.63	93.3	
	1943 or earlier; age 80+	0.50	(0.13,0.88)	0.19	0.23	0.73	0.80	90.3	
Gender	(Ref: Male)								<.001**
	Female	-0.11	(-0.18,-0.03)	0.04	0.50	0.14	0.16	87.3	
	Other	-0.28	(-0.82,0.26)	0.28	0.61	0.22	1.07	91.3	

Tables S2a-g: Argentina

Table S2a. Nationally representative descriptive statistics for Argentina

Characteristic	N = 6,724[1]
**Age group**	
1998-2005; age 18-24	1,108 (16%)
1993-1998; age 25-29	719 (11%)
1983-1993; age 30-39	1,432 (21%)
1973-1983; age 40-49	1,254 (19%)
1963-1973; age 50-59	1,014 (15%)
1953-1963; age 60-69	730 (11%)
1943-1953; age 70-79	356 (5.3%)
1943 or earlier; age 80+	112 (1.7%)
(Missing)	0 (0%)
**Gender**	
Male	3,143 (47%)
Female	3,542 (53%)
Other	21 (0.3%)
(Missing)	18 (0.3%)
**Race/Ethnicity**	
Asian	43 (0.6%)
Black	95 (1.4%)
Indigenous	129 (1.9%)
Mestizo(a)	1,801 (27%)
Mullato(a)	75 (1.1%)
Other	104 (1.5%)
White	3,406 (51%)
(Missing)	1,070 (16%)
**Respondent Marital status**	
Married	1,565 (23%)
Separated	455 (6.8%)
Divorced	321 (4.8%)
Widowed	401 (6.0%)
Single, never married	2,381 (35%)
Domestic Partner	1,514 (23%)
(Missing)	88 (1.3%)
**Employment**	
Employed for an employer	2,440 (36%)
Self-employed	1,748 (26%)
Retired	773 (11%)
Student	354 (5.3%)
Homemaker	639 (9.5%)
Unemployed and looking for a job	569 (8.5%)
None of these/Other	179 (2.7%)
(Missing)	22 (0.3%)
**Religious service attendance as an adult (now)**	
More than 1/week	532 (7.9%)
1/week	773 (12%)
1-3/month	461 (6.8%)
A few times a year	1,949 (29%)
Never	2,982 (44%)
(Missing)	27 (0.4%)
**Education (years)**	
Up to 8 years	2,263 (34%)
9-15 years	3,823 (57%)
16+ years	635 (9.4%)
(Missing)	3 (<0.1%)
**Immigration status**	
Born in this country	6,346 (94%)
Born in another country	348 (5.2%)
(Missing)	29 (0.4%)
**Religious affiliation as an adult (now)**	
Christianity	4,992 (74%)
Islam	9 (0.1%)
Hinduism	6 (<0.1%)

Characteristic	N = 6,724[1]
Buddhism	35 (0.5%)
Judaism	40 (0.6%)
Sikhism	0 (<0.1%)
Baha'i	0 (0%)
Jainism	0 (0%)
Shinto	0 (0%)
Taoism	2 (<0.1%)
Confucianism	0 (<0.1%)
Primal, Animist, or Folk religion	19 (0.3%)
Spiritism	0 (0%)
Umbanda, Candomble, and other African-derived religions	0 (0%)
Chinese folk/traditional religion	0 (0%)
Some other religion	156 (2.3%)
No religion/Atheist/Agnostic	1,352 (20%)
(Missing)	111 (1.7%)
**Relationship with mother growing up**	
Very good	4,463 (66%)
Somewhat good	1,436 (21%)
Somewhat bad	299 (4.4%)
Very bad	216 (3.2%)
Does not apply	273 (4.1%)
(Missing)	36 (0.5%)
**Relationship with father growing up**	
Very good	3,612 (54%)
Somewhat good	1,537 (23%)
Somewhat bad	440 (6.5%)
Very bad	401 (6.0%)
Does not apply	694 (10%)
(Missing)	39 (0.6%)
**Parent marital status at age 12**	
Parents married	4,110 (61%)
Divorced	637 (9.5%)
Parents were never married	1,368 (20%)
One or both parents had died	199 (3.0%)
(Missing)	410 (6.1%)
**Subjective financial status of family growing up**	
Lived comfortably	2,042 (30%)
Got by	2,305 (34%)
Found it difficult	1,789 (27%)
Found it very difficult	569 (8.5%)
(Missing)	19 (0.3%)
**Abuse**	
Yes	1,302 (19%)
No	5,271 (78%)
(Missing)	151 (2.2%)
**Outsider growing up**	
Yes	1,165 (17%)
No	5,458 (81%)
(Missing)	101 (1.5%)
**Self-rated health growing up**	
Excellent	2,402 (36%)
Very good	1,819 (27%)
Good	1,830 (27%)
Fair	505 (7.5%)
Poor	156 (2.3%)
(Missing)	12 (0.2%)
**Age 12 religious service attendance**	
At least 1/week	2,601 (39%)
1-3/month	1,204 (18%)
<1/month	1,059 (16%)
Never	1,808 (27%)
(Missing)	53 (0.8%)
**Religious affiliation at age 12**	
Christianity	5,805 (86%)
Islam	11 (0.2%)

**Characteristic**	**N = 6,724**[1]
Hinduism	2 (<0.1%)
Buddhism	3 (<0.1%)
Judaism	51 (0.8%)
Sikhism	5 (<0.1%)
Baha'i	0 (0%)
Jainism	0 (0%)
Shinto	0 (0%)
Taoism	1 (<0.1%)
Confucianism	0 (0%)
Primal, Animist, or Folk religion	17 (0.2%)
Spiritism	0 (0%)
Umbanda, Candomble, and other African-derived religions	0 (0%)
Chinese folk/traditional religion	0 (0%)
Some other religion	10 (0.2%)
No religion/Atheist/Agnostic	697 (10%)
(Missing)	122 (1.8%)

[1]n (%)

Table S2b. Means by demographic category for Argentina

		Cantril's Ladder				Life Satisfaction				Happiness			
Variable	Category	Mean	95% CI	SE	Global p-value	Mean	95% CI	SE	Global p-value	Mean	95% CI	SE	Global p-value
Age group	18-24	6.80	(6.62,6.98)	0.09	0.868	6.98	(6.76,7.19)	0.11	< .001	7.20	(7.01,7.38)	0.10	0.054
	25-29	6.85	(6.59,7.10)	0.13		7.09	(6.81,7.37)	0.14		7.39	(7.16,7.62)	0.12	
	30-39	6.73	(6.57,6.89)	0.08		7.02	(6.84,7.20)	0.09		7.33	(7.17,7.50)	0.08	
	40-49	6.80	(6.62,6.97)	0.09		7.47	(7.30,7.64)	0.09		7.60	(7.44,7.76)	0.08	
	50-59	6.67	(6.48,6.85)	0.09		7.44	(7.26,7.62)	0.09		7.43	(7.25,7.61)	0.09	
	60-69	6.65	(6.40,6.90)	0.13		7.24	(6.98,7.51)	0.13		7.31	(7.08,7.54)	0.12	
	70-79	6.63	(6.23,7.04)	0.21		7.28	(6.87,7.68)	0.20		7.08	(6.66,7.51)	0.22	
	80 or older	6.96	(6.19,7.72)	0.39		7.60	(6.72,8.48)	0.44		7.13	(6.45,7.81)	0.34	
Gender	Male	6.75	(6.64,6.86)	0.06	0.197	7.27	(7.15,7.39)	0.06	0.131	7.38	(7.26,7.49)	0.06	0.767
	Female	6.75	(6.64,6.86)	0.06		7.17	(7.05,7.29)	0.06		7.35	(7.25,7.45)	0.05	
	Other	5.79	(4.66,6.92)	0.53		6.15	(4.74,7.56)	0.66		6.81	(5.03,8.59)	0.84	
Marital status	Married	6.98	(6.82,7.13)	0.08	0.004	7.53	(7.36,7.70)	0.09	< .001	7.70	(7.55,7.85)	0.08	< .001
	Separated	6.84	(6.56,7.13)	0.15		7.08	(6.77,7.39)	0.16		7.25	(6.97,7.53)	0.14	
	Divorced	6.56	(6.21,6.92)	0.18		7.15	(6.81,7.48)	0.17		7.28	(6.95,7.62)	0.17	
	Widowed	6.67	(6.29,7.04)	0.19		7.52	(7.20,7.83)	0.16		7.20	(6.86,7.55)	0.17	
	Never	6.57	(6.44,6.71)	0.07		6.94	(6.79,7.09)	0.08		7.05	(6.91,7.18)	0.07	
	Domestic Partner	6.82	(6.67,6.97)	0.08		7.29	(7.14,7.45)	0.08		7.60	(7.45,7.74)	0.07	
Employment	Employed for an employer	6.94	(6.83,7.06)	0.06	< .001	7.31	(7.19,7.44)	0.06	< .001	7.48	(7.37,7.59)	0.06	< .001
	Self-employed	6.73	(6.58,6.88)	0.08		7.35	(7.19,7.50)	0.08		7.49	(7.34,7.63)	0.07	
	Retired	6.71	(6.44,6.97)	0.13		7.28	(7.00,7.56)	0.14		7.15	(6.89,7.41)	0.13	
	Student	6.78	(6.52,7.03)	0.13		6.90	(6.61,7.20)	0.15		7.07	(6.79,7.35)	0.14	
	Homemaker	6.82	(6.55,7.09)	0.14		7.41	(7.11,7.70)	0.15		7.59	(7.34,7.84)	0.13	
	Unemployed and looking for a job	6.11	(5.80,6.42)	0.16		6.50	(6.17,6.84)	0.17		6.88	(6.57,7.19)	0.16	
	None of these/Other	6.15	(5.53,6.77)	0.31		6.48	(5.87,7.09)	0.31		6.77	(6.16,7.37)	0.31	
Religious service attendance	More than 1/week	7.38	(7.06,7.69)	0.16	< .001	8.45	(8.17,8.73)	0.14	< .001	8.52	(8.30,8.74)	0.11	< .001
	1/week	7.21	(7.00,7.42)	0.11		7.66	(7.43,7.90)	0.12		7.64	(7.41,7.88)	0.12	
	1-3/month	6.72	(6.39,7.06)	0.17		7.18	(6.85,7.50)	0.17		7.42	(7.16,7.68)	0.13	
	A few times a year	6.76	(6.63,6.90)	0.07		7.22	(7.07,7.37)	0.08		7.44	(7.30,7.57)	0.07	
	Never	6.51	(6.39,6.63)	0.06		6.88	(6.75,7.01)	0.06		7.03	(6.91,7.14)	0.06	
Education	Up to 8 years	6.69	(6.51,6.88)	0.09	< .001	7.44	(7.25,7.63)	0.10	0.004	7.49	(7.31,7.67)	0.09	0.134
	9-15 years	6.73	(6.65,6.81)	0.04		7.08	(6.99,7.17)	0.05		7.29	(7.21,7.37)	0.04	

Variable	Category	Cantril's Ladder				Life Satisfaction				Happiness			
		Mean	95% CI	SE	Global p-value	Mean	95% CI	SE	Global p-value	Mean	95% CI	SE	Global p-value
	16+ years	7.05	(6.90,7.19)	0.08		7.21	(7.03,7.39)	0.09		7.33	(7.16,7.49)	0.08	
Immigration status	Born in this country	6.75	(6.67,6.83)	0.04	0.754	7.21	(7.12,7.29)	0.04	0.427	7.36	(7.28,7.44)	0.04	0.703
	Born in another country	6.68	(6.27,7.10)	0.21		7.37	(6.97,7.77)	0.20		7.42	(7.10,7.74)	0.16	
Religious affiliation	Christianity	6.81	(6.71,6.90)	0.05	< .001	7.36	(7.26,7.46)	0.05	< .001	7.47	(7.38,7.55)	0.04	< .001
	Islam	7.81	(4.65,10.0‡)	1.19		8.39	(5.83,10.0‡)	1.11		8.97	(7.67,10.0‡)	0.54	
	Hinduism	7.83	*	*		5.39	*	*		6.96	*	*	
	Buddhism	6.94	(6.34,7.54)	0.29		7.11	(6.38,7.84)	0.34		7.45	(6.87,8.04)	0.28	
	Judaism	7.57	(6.75,8.39)	0.40		7.30	(6.54,8.06)	0.37		6.62	(5.12,8.12)	0.73	
	Sikhism	8.00	*	*		8.00	*	*		9.00	*	*	
	Taoism	7.19	*	*		7.42	*	*		7.35	*	*	
	Confucianism	0.00	*	*		6.00	*	*		6.00	*	*	
	Primal, Animist, or Folk religion	7.22	(5.32,9.13)	0.78		7.13	(4.86,9.41)	0.91		8.24	(6.48,10.00)	0.71	
	Some other religion	6.35	(5.70,7.00)	0.33		7.16	(6.52,7.80)	0.32		7.83	(7.38,8.28)	0.23	
	No religion/Atheist /Agnostic	6.54	(6.38,6.69)	0.08		6.70	(6.52,6.87)	0.09		6.92	(6.76,7.08)	0.08	
Race/Ethnicity	Asian	7.09	(5.67,8.51)	0.69	0.012	7.58	(6.12,9.04)	0.71	< .001	7.61	(6.35,8.87)	0.61	0.015
	Black	7.60	(6.89,8.30)	0.35		8.55	(7.71,9.39)	0.41		8.46	(7.77,9.16)	0.35	
	Indigenous	6.77	(5.99,7.55)	0.38		7.29	(6.53,8.05)	0.37		7.14	(6.47,7.81)	0.34	
	Mestizo(a)	6.71	(6.56,6.86)	0.07		7.27	(7.11,7.43)	0.08		7.38	(7.24,7.51)	0.07	
	Mullato(a)	7.36	(6.72,8.01)	0.32		7.57	(6.88,8.27)	0.35		7.67	(7.03,8.32)	0.32	
	White	6.74	(6.64,6.84)	0.05		7.16	(7.05,7.27)	0.06		7.32	(7.22,7.42)	0.05	
	Other	6.24	(5.49,6.98)	0.37		6.33	(5.55,7.10)	0.39		7.18	(6.56,7.80)	0.31	

Table S2c. Childhood predictors regression analysis results for Argentina

		Cantril's Ladder				Life Satisfaction				Happiness			
Variable	Category	Est	95% CI	SE	Global p-value	Est	95% CI	SE	Global p-value	Est	95% CI	SE	Global p-value
Relationship with mother	(Ref: Very bad/somewhat bad)				0.100				<.001				<.001
	Very good/somewhat good	0.26	(-0.05, 0.58)	0.16		0.61	(0.24, 0.97)	0.19		0.58	(0.27, 0.89)	0.16	
Relationship with father	(Ref: Very bad/somewhat bad)				0.242				0.206				0.301
	Very good/somewhat good	0.14	(-0.11, 0.39)	0.13		0.16	(-0.09, 0.41)	0.13		0.13	(-0.12, 0.38)	0.13	
Parent marital status	(Ref: Parents married)				0.351				0.528				0.760
	Divorced	-0.08	(-0.33, 0.18)	0.13		0.01	(-0.27, 0.28)	0.14		-0.11	(-0.36, 0.15)	0.13	
	Parents were never married	0.04	(-0.18, 0.27)	0.12		0.16	(-0.09, 0.41)	0.13		0.00	(-0.23, 0.23)	0.12	
	One or both parents had died	-0.39	(-0.91, 0.13)	0.27		0.11	(-0.40, 0.62)	0.26		-0.15	(-0.69, 0.38)	0.27	
Subjective financial status of family growing up	(Ref: Got by)				<.001				<.001				<.001
	Lived comfortably	0.39	(0.21, 0.56)	0.09		0.42	(0.24, 0.61)	0.09		0.27	(0.10, 0.44)	0.09	
	Found it difficult	-0.14	(-0.34, 0.06)	0.10		-0.11	(-0.31, 0.10)	0.11		-0.11	(-0.30, 0.08)	0.10	
	Found it very difficult	-0.45	(-0.79, -0.10)	0.18		0.18	(-0.20, 0.56)	0.20		0.02	(-0.34, 0.39)	0.19	
Abuse	(Ref: No)				0.260				<.001				0.021
	Yes	-0.12	(-0.32, 0.09)	0.11		-0.51	(-0.74, -0.28)	0.12		-0.24	(-0.44, -0.04)	0.10	
Outsider growing up	(Ref: No)				0.015				0.011				0.014
	Yes	-0.30	(-0.55, -0.06)	0.13		-0.34	(-0.61, -0.08)	0.14		-0.30	(-0.55, -0.05)	0.13	
Self-rated health growing up	(Ref: Good)				0.001				<.001				<.001
	Excellent	0.35	(0.16, 0.55)	0.10		0.34	(0.13, 0.55)	0.11		0.39	(0.19, 0.59)	0.10	

Variable	Category	Cantril's Ladder				Life Satisfaction				Happiness			
		Est	95% CI	SE	Global p-value	Est	95% CI	SE	Global p-value	Est	95% CI	SE	Global p-value
	Very good	0.16	(-0.04, 0.35)	0.10		0.09	(-0.12, 0.30)	0.11		0.04	(-0.15, 0.24)	0.10	
	Fair	-0.05	(-0.39, 0.28)	0.17		-0.21	(-0.61, 0.20)	0.21		-0.12	(-0.48, 0.23)	0.18	
	Poor	-0.56	(-1.34, 0.22)	0.40		-0.90	(-1.74, -0.06)	0.43		-0.74	(-1.54, 0.07)	0.41	
Immigration status	(Ref: Born in this country)				0.764				0.443				0.654
	Born in another country	-0.06	(-0.47, 0.35)	0.21		0.15	(-0.23, 0.53)	0.20		0.07	(-0.24, 0.38)	0.16	
Age 12 religious service attendance	(Ref: Never)				0.802				0.052				0.058
	At least 1/week	0.10	(-0.12, 0.31)	0.11		0.16	(-0.06, 0.39)	0.11		0.20	(-0.00, 0.41)	0.11	
	1-3/month	0.02	(-0.22, 0.26)	0.12		-0.14	(-0.40, 0.11)	0.13		0.07	(-0.16, 0.30)	0.12	
	< 1/month	0.05	(-0.17, 0.27)	0.11		-0.02	(-0.26, 0.22)	0.12		-0.06	(-0.29, 0.17)	0.12	
Year of birth	(Ref: 1998-2005; current age: 18-24)				0.973				<.001				0.043
	1993-1998; age 25-29	0.03	(-0.26, 0.33)	0.15		0.05	(-0.28, 0.39)	0.17		0.13	(-0.15, 0.40)	0.14	
	1983-1993; age 30-39	-0.03	(-0.26, 0.20)	0.12		0.05	(-0.23, 0.32)	0.14		0.11	(-0.13, 0.35)	0.12	
	1973-1983; age 40-49	0.04	(-0.20, 0.29)	0.13		0.50	(0.23, 0.78)	0.14		0.37	(0.13, 0.61)	0.12	
	1963-1973; age 50-59	-0.06	(-0.32, 0.21)	0.14		0.51	(0.22, 0.80)	0.15		0.21	(-0.05, 0.47)	0.13	
	1953-1963; age 60-69	-0.10	(-0.41, 0.20)	0.16		0.26	(-0.07, 0.60)	0.17		0.06	(-0.23, 0.36)	0.15	
	1943-1953; age 70-79	-0.12	(-0.56, 0.33)	0.23		0.32	(-0.11, 0.75)	0.22		-0.15	(-0.59, 0.29)	0.23	
	1943 or earlier; age 80+	0.11	(-0.67, 0.89)	0.40		0.45	(-0.37, 1.27)	0.42		-0.24	(-0.91, 0.42)	0.34	
Gender	(Ref: Male)				0.157				0.589				0.461
	Female	0.09	(-0.06, 0.24)	0.08		0.05	(-0.12, 0.21)	0.08		0.09	(-0.06, 0.24)	0.08	
	Other	-0.81	(-1.91, 0.29)	0.56		-0.46	(-1.60, 0.68)	0.58		-0.17	(-1.47, 1.14)	0.67	
Religious affiliation	(Ref: No religion/Atheist /Agnostic)				0.217				0.843				0.772
	Christianity	0.01	(-0.26, 0.27)	0.13		0.01	(-0.28, 0.31)	0.15		0.07	(-0.18, 0.33)	0.13	

		Cantril’s Ladder				Life Satisfaction				Happiness			
Variable	Category	Est	95% CI	SE	Global p-value	Est	95% CI	SE	Global p-value	Est	95% CI	SE	Global p-value
	Collapsed affiliations with prevalence<3%	0.44	(-0.11, 0.99)	0.28		0.14	(-0.45, 0.73)	0.30		-0.09	(-0.91, 0.73)	0.42	
Race/ethnicity	(Ref: Plurality group)				0.065				0.002				0.021
	Non-plurality groups	0.14	(-0.09, 0.36)	0.11		0.27	(0.08, 0.45)	0.09		0.18	(0.02, 0.34)	0.08	

Table S2d. Sensitivity to unmeasured confounding of childhood predictors in Argentina

Variable	Category	Cantril's Ladder		Life Satisfaction		Happiness	
		E-value for Estimate	E-value for 95% CI	E-value for Estimate	E-value for 95% CI	E-value for Estimate	E-value for 95% CI
Relationship with mother	(Ref: Very bad/somewhat bad)						
	Very good/somewhat good	1.47	1.00	1.84	1.42	1.88	1.49
Relationship with father	(Ref: Very bad/somewhat bad)						
	Very good/somewhat good	1.31	1.00	1.32	1.00	1.31	1.00
Parent marital status	(Ref: Parents married)						
	Divorced	1.21	1.00	1.06	1.00	1.27	1.00
	Parents were never married	1.15	1.00	1.32	1.00	1.01	1.00
	One or both parents had died	1.63	1.00	1.26	1.00	1.33	1.00
Subjective financial status of family growing up	(Ref: Got by)						
	Lived comfortably	1.63	1.41	1.63	1.42	1.49	1.26
	Found it difficult	1.31	1.00	1.25	1.00	1.27	1.00
	Found it very difficult	1.70	1.26	1.35	1.00	1.11	1.00
Abuse	(Ref: No)						
	Yes	1.28	1.00	1.73	1.46	1.45	1.14
Outsider growing up	(Ref: No)						
	Yes	1.53	1.18	1.54	1.21	1.53	1.17
Self-rated health growing up	(Ref: Good)						
	Excellent	1.59	1.34	1.54	1.28	1.64	1.39
	Very good	1.34	1.00	1.22	1.00	1.16	1.00
	Fair	1.17	1.00	1.38	1.00	1.29	1.00
	Poor	1.84	1.00	2.17	1.18	2.08	1.00
Immigration status	(Ref: Born in this country)						
	Born in another country	1.19	1.00	1.31	1.00	1.20	1.00
Age 12 religious service attendance	(Ref: Never)						
	At least 1/week	1.25	1.00	1.33	1.00	1.40	1.00
	1-3/month	1.10	1.00	1.30	1.00	1.21	1.00
	< 1/month	1.16	1.00	1.10	1.00	1.19	1.00
Year of birth	(Ref: 1998-2005; current age: 18-24)						
	1993-1998; age 25-29	1.14	1.00	1.17	1.00	1.30	1.00
	1983-1993; age 30-39	1.13	1.00	1.15	1.00	1.27	1.00
	1973-1983; age 40-49	1.15	1.00	1.72	1.41	1.62	1.30
	1963-1973; age 50-59	1.18	1.00	1.73	1.40	1.41	1.00

Variable	Category	Cantril's Ladder		Life Satisfaction		Happiness	
		E-value for Estimate	E-value for 95% CI	E-value for Estimate	E-value for 95% CI	E-value for Estimate	E-value for 95% CI
	1953-1963; age 60-69	1.26	1.00	1.45	1.00	1.19	1.00
	1943-1953; age 70-79	1.28	1.00	1.51	1.00	1.33	1.00
	1943 or earlier; age 80+	1.26	1.00	1.66	1.00	1.46	1.00
Gender	(Ref: Male)						
	Female	1.24	1.00	1.15	1.00	1.24	1.00
	Other	2.15	1.00	1.67	1.00	1.35	1.00
Religious affiliation	(Ref: No religion/Atheist/Agnostic)						
	Christianity	1.05	1.00	1.08	1.00	1.21	1.00
	Collapsed affiliations with prevalence<3%	1.69	1.00	1.30	1.00	1.24	1.00
Race/ethnicity	(Ref: Plurality group)						
	Non-plurality groups	1.31	1.00	1.45	1.22	1.37	1.11

Table S2e. Complete-case supplemental analysis of means by demographic category for Argentina

		Cantril's Ladder				Life Satisfaction				Happiness			
Variable	Category	Mean	95% CI	SE	Global p-value	Mean	95% CI	SE	Global p-value	Mean	95% CI	SE	Global p-value
Age group	18-24	6.88	(6.71,7.05)	0.09	0.619	7.12	(6.92,7.33)	0.10	< .001	7.31	(7.14,7.48)	0.09	0.039
	25-29	7.00	(6.77,7.23)	0.12		7.40	(7.17,7.62)	0.11		7.51	(7.29,7.72)	0.11	
	30-39	6.85	(6.70,6.99)	0.07		7.24	(7.08,7.39)	0.08		7.46	(7.31,7.60)	0.07	
	40-49	6.95	(6.80,7.10)	0.08		7.58	(7.42,7.75)	0.08		7.70	(7.55,7.85)	0.08	
	50-59	6.76	(6.58,6.94)	0.09		7.59	(7.43,7.76)	0.08		7.55	(7.40,7.71)	0.08	
	60-69	6.83	(6.61,7.05)	0.11		7.44	(7.21,7.67)	0.12		7.38	(7.15,7.60)	0.11	
	70-79	6.78	(6.43,7.14)	0.18		7.41	(7.05,7.77)	0.18		7.39	(7.05,7.74)	0.17	
	80 or older	7.22	(6.58,7.86)	0.32		8.17	(7.70,8.64)	0.24		7.32	(6.73,7.91)	0.30	
Gender	Female	6.90	(6.80,7.00)	0.05	0.106	7.40	(7.30,7.50)	0.05	0.253	7.48	(7.38,7.57)	0.05	0.689
	Male	6.86	(6.76,6.96)	0.05		7.41	(7.30,7.52)	0.06		7.50	(7.39,7.60)	0.05	
	Other	5.79	(4.65,6.93)	0.54		6.32	(4.92,7.71)	0.66		6.79	(5.01,8.58)	0.84	
Marital status	Divorced	6.77	(6.48,7.06)	0.15	0.010	7.28	(6.97,7.58)	0.15	< .001	7.51	(7.26,7.75)	0.12	< .001
	Domestic partner	6.92	(6.78,7.05)	0.07		7.42	(7.28,7.56)	0.07		7.67	(7.54,7.80)	0.07	
	Married	7.08	(6.94,7.22)	0.07		7.71	(7.56,7.86)	0.08		7.82	(7.68,7.95)	0.07	
	Separated	6.90	(6.62,7.19)	0.15		7.19	(6.90,7.49)	0.15		7.28	(7.00,7.56)	0.14	
	Single/Never been married	6.73	(6.61,6.85)	0.06		7.20	(7.07,7.33)	0.07		7.21	(7.09,7.32)	0.06	
	Widowed	6.84	(6.51,7.17)	0.17		7.60	(7.29,7.91)	0.16		7.32	(7.00,7.64)	0.16	
Employment	Employed for an employer	7.00	(6.89,7.11)	0.06	0.009	7.41	(7.29,7.53)	0.06	< .001	7.55	(7.44,7.66)	0.05	0.002
	Homemaker	6.93	(6.68,7.19)	0.13		7.65	(7.39,7.92)	0.13		7.72	(7.49,7.95)	0.12	
	None of these/Other	6.57	(6.04,7.11)	0.27		7.10	(6.64,7.56)	0.23		7.34	(6.94,7.74)	0.20	
	Retired	6.90	(6.67,7.13)	0.12		7.53	(7.30,7.76)	0.12		7.32	(7.09,7.55)	0.12	
	Self-employed	6.84	(6.71,6.98)	0.07		7.48	(7.33,7.62)	0.07		7.55	(7.41,7.68)	0.07	
	Student	6.81	(6.56,7.06)	0.13		7.06	(6.80,7.32)	0.13		7.10	(6.82,7.37)	0.14	
	Unemployed and looking for a job	6.44	(6.18,6.71)	0.13		6.94	(6.66,7.23)	0.15		7.22	(6.97,7.46)	0.13	
Religious service attendance	A few times a year	6.88	(6.76,7.00)	0.06	< .001	7.40	(7.27,7.53)	0.07	< .001	7.53	(7.41,7.65)	0.06	< .001
	More than once a week	7.51	(7.22,7.79)	0.14		8.60	(8.39,8.81)	0.11		8.55	(8.33,8.76)	0.11	
	Never	6.63	(6.53,6.74)	0.05		7.09	(6.98,7.20)	0.06		7.18	(7.08,7.28)	0.05	
	Once a week	7.28	(7.07,7.49)	0.11		7.73	(7.49,7.96)	0.12		7.80	(7.59,8.02)	0.11	

Variable	Category	Cantril's Ladder				Life Satisfaction				Happiness			
		Mean	95% CI	SE	Global p-value	Mean	95% CI	SE	Global p-value	Mean	95% CI	SE	Global p-value
	One to three times a month	7.00	(6.75,7.26)	0.13		7.42	(7.14,7.70)	0.14		7.47	(7.22,7.72)	0.13	
Education	Up to 8 years	6.94	(6.78,7.10)	0.08	0.005	7.73	(7.56,7.89)	0.08	< .001	7.71	(7.55,7.87)	0.08	< .001
	16+ years	7.06	(6.92,7.21)	0.07		7.30	(7.13,7.47)	0.09		7.35	(7.19,7.51)	0.08	
	9 to 15 years	6.81	(6.73,6.88)	0.04		7.23	(7.15,7.31)	0.04		7.37	(7.30,7.44)	0.04	
Immigration status	Born in another country	6.91	(6.54,7.27)	0.19	0.866	7.53	(7.16,7.90)	0.19	0.461	7.45	(7.12,7.77)	0.16	0.819
	Born in this country	6.87	(6.80,6.94)	0.04		7.39	(7.32,7.47)	0.04		7.48	(7.41,7.56)	0.04	
Religious affiliation	Buddhism	6.93	(6.31,7.54)	0.29	< .001	7.18	(6.61,7.74)	0.27	< .001	7.44	(6.89,8.00)	0.27	< .001
	Christianity	6.93	(6.85,7.02)	0.04		7.53	(7.44,7.62)	0.04		7.60	(7.52,7.68)	0.04	
	Confucianism	0.00	*	*		6.00	*	*		6.00	*	*	
	Hinduism	7.83	*	*		5.35	*	*		6.94	*	*	
	Islam	7.80	(4.99,10.62)	1.23		8.39	(5.83,10.95)	1.11		8.98	(7.73,10.23)	0.54	
	Judaism	7.48	(6.72,8.23)	0.37		7.43	(6.67,8.18)	0.37		7.28	(6.47,8.10)	0.40	
	No religion/Atheist/Agnostic	6.64	(6.50,6.79)	0.07		6.93	(6.78,7.08)	0.08		7.01	(6.85,7.16)	0.08	
	Primal, Animist, or Folk religion	7.10	(5.14,9.07)	0.78		7.13	(4.86,9.41)	0.91		8.25	(6.45,10.05)	0.72	
	Sikhism	8.00	*	*		8.00	*	*		9.00	*	*	
	Some other religion	6.72	(6.23,7.20)	0.25		7.42	(6.84,7.99)	0.29		7.90	(7.44,8.35)	0.23	
	Taoism	7.21	*	*		7.42	*	*		7.33	*	*	

Table S2f. Complete-case supplemental analysis of childhood predictors regression analysis results for Argentina

		Cantril's Ladder				Life Satisfaction				Happiness			
Variable	Category	Est	95% CI	SE	Global p-value	Est	95% CI	SE	Global p-value	Est	95% CI	SE	Global p-value
Relationship with mother	(Ref: Very bad/somewhat bad)				0.268				0.011				<.001
	Very good/somewhat good	0.17	(-0.13,0.46)	0.15		0.40	(0.09,0.71)	0.16		0.52	(0.25,0.80)	0.14	
Relationship with father	(Ref: Very bad/somewhat bad)				0.387				0.198				0.626
	Very good/somewhat good	0.10	(-0.12,0.31)	0.11		0.15	(-0.08,0.38)	0.12		0.06	(-0.17,0.28)	0.12	
Parent marital status	(Ref: Parents married)				0.250				0.208				0.188
	No, one or both of them had died	-0.08	(-0.48,0.32)	0.20		0.31	(-0.09,0.71)	0.20		0.13	(-0.28,0.55)	0.21	
	No, they were never married	0.22	(-0.04,0.48)	0.13		0.29	(0.00,0.58)	0.15		0.28	(0.03,0.53)	0.13	
	Yes, married	0.10	(-0.13,0.34)	0.12		0.18	(-0.08,0.45)	0.13		0.20	(-0.03,0.44)	0.12	
Subjective financial status of family growing up	(Ref: Got by)				<.001				<.001				<.001
	Found it difficult	-0.09	(-0.26,0.09)	0.09		-0.19	(-0.37,0.00)	0.10		-0.12	(-0.29,0.05)	0.09	
	Found it very difficult	-0.40	(-0.70,-0.09)	0.16		0.29	(-0.04,0.61)	0.17		0.10	(-0.23,0.43)	0.17	
	Lived comfortably	0.37	(0.21,0.53)	0.08		0.32	(0.15,0.48)	0.09		0.26	(0.11,0.41)	0.08	
Abuse	(Ref: No)				0.009				<.001				<.001
	Yes	-0.25	(-0.44,-0.06)	0.09		-0.47	(-0.67,-0.26)	0.10		-0.34	(-0.52,-0.15)	0.10	
Outsider growing up	(Ref: No)				0.047				0.069				0.025
	Yes	-0.23	(-0.45,-0.00)	0.11		-0.22	(-0.46,0.02)	0.12		-0.26	(-0.48,-0.03)	0.11	

		Cantril's Ladder				Life Satisfaction				Happiness			
Variable	Category	Est	95% CI	SE	Global p-value	Est	95% CI	SE	Global p-value	Est	95% CI	SE	Global p-value
Self-rated health growing up	(Ref: Good)				<.001				<.001				<.001
	Excellent	0.35	(0.17,0.53)	0.09		0.34	(0.15,0.54)	0.10		0.45	(0.26,0.63)	0.09	
	Fair	-0.02	(-0.31,0.26)	0.14		0.01	(-0.34,0.35)	0.18		0.00	(-0.31,0.32)	0.16	
	Poor	0.01	(-0.62,0.65)	0.32		-0.45	(-1.17,0.28)	0.37		-0.14	(-0.80,0.53)	0.34	
	Very good	0.09	(-0.09,0.28)	0.09		-0.01	(-0.20,0.19)	0.10		0.04	(-0.14,0.22)	0.09	
Immigration status	(Ref: Born in this country)				0.849				0.479				0.932
	Born in another country	0.04	(-0.33,0.40)	0.19		0.13	(-0.23,0.49)	0.18		-0.01	(-0.32,0.29)	0.16	
Age 12 religious service attendance	(Ref: Never)				0.373				0.035				0.011
	At least once a week	0.12	(-0.07,0.31)	0.10		0.12	(-0.08,0.32)	0.10		0.21	(0.02,0.39)	0.09	
	Less than once a month	-0.02	(-0.23,0.19)	0.11		-0.11	(-0.34,0.11)	0.12		-0.08	(-0.29,0.13)	0.11	
	One to three times a month	-0.01	(-0.23,0.22)	0.11		-0.14	(-0.37,0.09)	0.12		0.02	(-0.19,0.23)	0.11	
Year of birth	(Ref: 1998-2005; current age: 18-24)				0.748				<.001				0.019
	1993-1998; age 25-29	0.12	(-0.15,0.39)	0.14		0.23	(-0.06,0.53)	0.15		0.16	(-0.09,0.42)	0.13	
	1983-1993; age 30-39	0.01	(-0.21,0.23)	0.11		0.11	(-0.14,0.37)	0.13		0.15	(-0.07,0.38)	0.11	
	1973-1983; age 40-49	0.13	(-0.10,0.35)	0.11		0.46	(0.20,0.73)	0.14		0.39	(0.17,0.61)	0.11	
	1963-1973; age 50-59	-0.05	(-0.30,0.20)	0.13		0.48	(0.21,0.75)	0.14		0.25	(0.02,0.48)	0.12	
	1953-1963; age 60-69	0.00	(-0.28,0.28)	0.14		0.29	(-0.02,0.61)	0.16		0.05	(-0.23,0.33)	0.14	
	1943-1953; age 70-79	-0.06	(-0.45,0.34)	0.20		0.27	(-0.14,0.69)	0.21		0.08	(-0.29,0.44)	0.19	

		Cantril's Ladder				Life Satisfaction				Happiness			
Variable	Category	Est	95% CI	SE	Global p-value	Est	95% CI	SE	Global p-value	Est	95% CI	SE	Global p-value
	1943 or earlier; age 80+	0.30	(-0.33,0.93)	0.32		0.82	(0.28,1.36)	0.27		-0.15	(-0.71,0.42)	0.29	
Gender	(Ref: Male)				0.062				0.210				0.371
	Male	-0.12	(-0.26,0.01)	0.07		-0.10	(-0.25,0.05)	0.08		-0.09	(-0.23,0.05)	0.07	
	Other	-0.95	(-2.07,0.17)	0.57		-0.67	(-1.80,0.45)	0.57		-0.37	(-1.71,0.97)	0.68	
Religious affiliation	(Ref: No religion/Atheist/Agnostic)				0.543				0.987				0.814
	Christianity	-0.02	(-0.26,0.21)	0.12		-0.01	(-0.27,0.25)	0.13		0.05	(-0.18,0.29)	0.12	
	Christianity	-0.02	(-0.26,0.21)	0.12		-0.01	(-0.27,0.25)	0.13		0.05	(-0.18,0.29)	0.12	
	Collapsed affiliations with prevalence<3%	0.26	(-0.29,0.81)	0.28		-0.04	(-0.59,0.50)	0.28		0.17	(-0.37,0.70)	0.27	
Race/ethnicity	(Ref: Plurality group)				0.148				<.001				0.059
	Non-plurality groups	0.11	(-0.04,0.25)	0.07		0.25	(0.10,0.40)	0.08		0.13	(-0.00,0.27)	0.07	

Table S2g. Complete-case supplemental analysis of sensitivity to unmeasured confounding of childhood predictors in Argentina

		Cantril's Ladder		Life Satisfaction		Happiness	
Variable	Category	E-value for Estimate	E-value for 95% CI	E-value for Estimate	E-value for 95% CI	E-value for Estimate	E-value for 95% CI
Relationship with mother	(Ref: Very bad/somewhat bad)						
	Very good/somewhat good	1.37	1.00	1.67	1.25	1.88	1.49
Relationship with father	(Ref: Very bad/somewhat bad)						
	Very good/somewhat good	1.26	1.00	1.33	1.00	1.19	1.00
Parent marital status	(Ref: Parents married)						
	No, one or both of them had died	1.23	1.00	1.55	1.00	1.33	1.00
	No, they were never married	1.44	1.00	1.53	1.05	1.54	1.12
	Yes, married	1.27	1.00	1.38	1.00	1.43	1.00
Subjective financial status of family growing up	(Ref: Got by)						
	Found it difficult	1.24	1.00	1.38	1.00	1.31	1.00
	Found it very difficult	1.69	1.25	1.52	1.00	1.27	1.00
	Lived comfortably	1.65	1.43	1.56	1.33	1.51	1.29
Abuse	(Ref: No)						
	Yes	1.49	1.20	1.75	1.49	1.62	1.35
Outsider growing up	(Ref: No)						
	Yes	1.45	1.04	1.43	1.00	1.51	1.14
Self-rated health growing up	(Ref: Good)						
	Excellent	1.63	1.38	1.59	1.34	1.77	1.52
	Fair	1.11	1.00	1.05	1.00	1.04	1.00
	Poor	1.08	1.00	1.72	1.00	1.33	1.00
	Very good	1.25	1.00	1.05	1.00	1.15	1.00
Immigration status	(Ref: Born in this country)						
	Born in another country	1.15	1.00	1.31	1.00	1.09	1.00
Age 12 religious service attendance	(Ref: Never)						
	At least once a week	1.30	1.00	1.29	1.00	1.44	1.12
	Less than once a month	1.11	1.00	1.28	1.00	1.24	1.00
	One to three times a month	1.05	1.00	1.32	1.00	1.11	1.00
Year of birth	(Ref: 1998-2005; current age: 18-24)						

Variable	Category	Cantril's Ladder		Life Satisfaction		Happiness	
		E-value for Estimate	E-value for 95% CI	E-value for Estimate	E-value for 95% CI	E-value for Estimate	E-value for 95% CI
	1993-1998; age 25-29	1.30	1.00	1.45	1.00	1.37	1.00
	1983-1993; age 30-39	1.07	1.00	1.28	1.00	1.36	1.00
	1973-1983; age 40-49	1.31	1.00	1.74	1.40	1.70	1.38
	1963-1973; age 50-59	1.18	1.00	1.76	1.42	1.50	1.10
	1953-1963; age 60-69	1.01	1.00	1.53	1.00	1.17	1.00
	1943-1953; age 70-79	1.19	1.00	1.50	1.00	1.23	1.00
	1943 or earlier; age 80+	1.55	1.00	2.20	1.51	1.35	1.00
Gender	(Ref: Male)						
	Male	1.31	1.00	1.26	1.00	1.26	1.00
	Other	2.46	1.00	2.01	1.00	1.67	1.00
Religious affiliation	(Ref: No religion/Atheist/Agnostic)						
	Christianity	1.12	1.00	1.08	1.00	1.18	1.00
	Christianity	1.12	1.00	1.08	1.00	1.18	1.00
	Collapsed affiliations with prevalence<3%	1.50	1.00	1.16	1.00	1.38	1.00
Race/ethnicity	(Ref: Plurality group)						
	Non-plurality groups	1.28	1.00	1.47	1.26	1.33	1.00

Tables S3a-g: Australia

Table S3a. Nationally representative descriptive statistics for Australia

Characteristic	N = 3,844[1]
**Age group**	
1998-2005; age 18-24	345 (9.0%)
1993-1998; age 25-29	282 (7.3%)
1983-1993; age 30-39	641 (17%)
1973-1983; age 40-49	618 (16%)
1963-1973; age 50-59	691 (18%)
1953-1963; age 60-69	589 (15%)
1943-1953; age 70-79	498 (13%)
1943 or earlier; age 80+	178 (4.6%)
(Missing)	2 (<0.1%)
**Gender**	
Male	1,861 (48%)
Female	1,941 (50%)
Other	36 (0.9%)
(Missing)	6 (0.2%)
**Race/Ethnicity**	
Aboriginal	53 (1.4%)
Australian	1,946 (51%)
Australian British/European	1,047 (27%)
Chinese	75 (1.9%)
Indian	58 (1.5%)
Japanese	1 (<0.1%)
Malay	11 (0.3%)
New Zealander	91 (2.4%)
Other	163 (4.2%)
Other European	357 (9.3%)
Russian	7 (0.2%)
Samoan	4 (0.1%)
Sinhalese	1 (<0.1%)
Spanish	2 (<0.1%)
Sri Lankan Moor	1 (<0.1%)
Sri Lankan Tamil	7 (0.2%)
Vietnamese	7 (0.2%)
(Missing)	14 (0.4%)
**Respondent Marital status**	
Married	1,797 (47%)
Separated	158 (4.1%)
Divorced	332 (8.6%)
Widowed	215 (5.6%)
Single, never married	855 (22%)
Domestic Partner	450 (12%)
(Missing)	38 (1.0%)
**Employment**	
Employed for an employer	1,881 (49%)
Self-employed	380 (9.9%)
Retired	912 (24%)
Student	190 (5.0%)
Homemaker	137 (3.6%)
Unemployed and looking for a job	134 (3.5%)
None of these/Other	206 (5.4%)
(Missing)	4 (0.1%)
**Religious service attendance as an adult (now)**	
More than 1/week	162 (4.2%)
1/week	299 (7.8%)
1-3/month	135 (3.5%)
A few times a year	656 (17%)
Never	2,584 (67%)
(Missing)	7 (0.2%)
**Education (years)**	
Up to 8 years	70 (1.8%)

Characteristic	N = 3,844[1]
9-15 years	2,434 (63%)
16+ years	1,330 (35%)
(Missing)	10 (0.3%)
**Immigration status**	
Born in this country	2,953 (77%)
Born in another country	885 (23%)
(Missing)	6 (0.2%)
**Religious affiliation as an adult (now)**	
Christianity	1,592 (41%)
Islam	45 (1.2%)
Hinduism	31 (0.8%)
Buddhism	36 (0.9%)
Judaism	26 (0.7%)
Sikhism	8 (0.2%)
Baha'i	7 (0.2%)
Jainism	0 (0%)
Shinto	0 (0%)
Taoism	5 (0.1%)
Confucianism	0 (0%)
Primal, Animist, or Folk religion	23 (0.6%)
Spiritism	0 (0%)
Umbanda, Candomble, and other African-derived religions	0 (0%)
Chinese folk/traditional religion	0 (0%)
Some other religion	39 (1.0%)
No religion/Atheist/Agnostic	2,020 (53%)
(Missing)	15 (0.4%)
**Relationship with mother growing up**	
Very good	2,554 (66%)
Somewhat good	925 (24%)
Somewhat bad	218 (5.7%)
Very bad	107 (2.8%)
Does not apply	32 (0.8%)
(Missing)	7 (0.2%)
**Relationship with father growing up**	
Very good	2,032 (53%)
Somewhat good	1,144 (30%)
Somewhat bad	315 (8.2%)
Very bad	196 (5.1%)
Does not apply	148 (3.9%)
(Missing)	9 (0.2%)
**Parent marital status at age 12**	
Parents married	3,048 (79%)
Divorced	462 (12%)
Parents were never married	187 (4.9%)
One or both parents had died	96 (2.5%)
(Missing)	52 (1.4%)
**Subjective financial status of family growing up**	
Lived comfortably	1,756 (46%)
Got by	1,496 (39%)
Found it difficult	422 (11%)
Found it very difficult	154 (4.0%)
(Missing)	16 (0.4%)
**Abuse**	
Yes	995 (26%)
No	2,790 (73%)
(Missing)	59 (1.5%)
**Outsider growing up**	
Yes	756 (20%)
No	3,062 (80%)
(Missing)	26 (0.7%)
**Self-rated health growing up**	
Excellent	1,736 (45%)
Very good	1,087 (28%)
Good	603 (16%)
Fair	308 (8.0%)

Characteristic	N = 3,844[1]
Poor	106 (2.8%)
(Missing)	4 (<0.1%)
**Age 12 religious service attendance**	
At least 1/week	1,362 (35%)
1-3/month	486 (13%)
<1/month	600 (16%)
Never	1,307 (34%)
(Missing)	90 (2.3%)
**Religious affiliation at age 12**	
Christianity	2,678 (70%)
Islam	48 (1.2%)
Hinduism	39 (1.0%)
Buddhism	16 (0.4%)
Judaism	29 (0.8%)
Sikhism	6 (0.2%)
Baha'i	5 (0.1%)
Jainism	0 (0%)
Shinto	0 (0%)
Taoism	1 (<0.1%)
Confucianism	0 (0%)
Primal, Animist, or Folk religion	4 (<0.1%)
Spiritism	0 (0%)
Umbanda, Candomble, and other African-derived religions	0 (0%)
Chinese folk/traditional religion	0 (0%)
Some other religion	8 (0.2%)
No religion/Atheist/Agnostic	990 (26%)
(Missing)	21 (0.5%)

[1]n (%)

Table S3b. Means by demographic category for Australia

Variable	Category	Cantril's Ladder				Life Satisfaction				Happiness			
		Mean	95% CI	SE	Global p-value	Mean	95% CI	SE	Global p-value	Mean	95% CI	SE	Global p-value
Age group	18-24	6.09	(5.82,6.35)	0.14	< .001	5.88	(5.53,6.23)	0.18	< .001	6.13	(5.84,6.42)	0.15	< .001
	25-29	6.32	(6.04,6.61)	0.15		6.05	(5.69,6.40)	0.18		6.30	(6.00,6.61)	0.15	
	30-39	6.65	(6.47,6.84)	0.09		6.39	(6.14,6.63)	0.12		6.56	(6.35,6.76)	0.11	
	40-49	6.65	(6.47,6.84)	0.09		6.63	(6.41,6.84)	0.11		6.78	(6.61,6.95)	0.09	
	50-59	6.58	(6.41,6.75)	0.09		6.52	(6.33,6.72)	0.10		6.64	(6.46,6.82)	0.09	
	60-69	7.04	(6.90,7.19)	0.07		7.08	(6.91,7.25)	0.09		7.25	(7.11,7.39)	0.07	
	70-79	7.52	(7.36,7.69)	0.09		7.66	(7.47,7.85)	0.10		7.74	(7.57,7.91)	0.09	
	80 or older	7.67	(7.36,7.98)	0.16		7.91	(7.68,8.15)	0.12		8.07	(7.86,8.28)	0.11	
Gender	Male	6.81	(6.71,6.91)	0.05	0.142	6.68	(6.55,6.81)	0.06	0.009	6.86	(6.75,6.97)	0.05	0.004
	Female	6.77	(6.67,6.88)	0.05		6.78	(6.66,6.91)	0.06		6.92	(6.82,7.03)	0.05	
	Other	6.19	(5.55,6.83)	0.31		5.29	(4.25,6.32)	0.51		5.66	(4.87,6.45)	0.39	
Marital status	Married	7.29	(7.21,7.38)	0.04	< .001	7.35	(7.25,7.44)	0.05	< .001	7.37	(7.29,7.46)	0.04	< .001
	Separated	5.75	(5.30,6.21)	0.23		5.53	(5.03,6.03)	0.25		6.01	(5.55,6.47)	0.23	
	Divorced	6.44	(6.19,6.68)	0.12		6.45	(6.17,6.73)	0.14		6.68	(6.44,6.91)	0.12	
	Widowed	7.31	(7.04,7.58)	0.14		7.43	(7.14,7.72)	0.15		7.57	(7.32,7.82)	0.13	
	Never	6.04	(5.86,6.21)	0.09		5.66	(5.45,5.88)	0.11		6.05	(5.87,6.24)	0.09	
	Domestic Partner	6.54	(6.32,6.76)	0.11		6.51	(6.23,6.78)	0.14		6.63	(6.39,6.86)	0.12	
Employment	Employed for an employer	6.74	(6.65,6.83)	0.05	< .001	6.66	(6.55,6.77)	0.06	< .001	6.80	(6.70,6.89)	0.05	< .001
	Self-employed	6.99	(6.76,7.22)	0.12		6.98	(6.72,7.23)	0.13		7.11	(6.90,7.32)	0.11	
	Retired	7.36	(7.23,7.50)	0.07		7.49	(7.34,7.64)	0.08		7.61	(7.48,7.74)	0.07	
	Student	6.26	(5.92,6.61)	0.17		6.06	(5.60,6.52)	0.23		6.17	(5.80,6.54)	0.19	
	Homemaker	6.59	(6.13,7.04)	0.23		6.62	(6.14,7.11)	0.24		6.71	(6.27,7.16)	0.22	
	Unemployed and looking for a job	5.54	(4.99,6.08)	0.28		5.02	(4.38,5.65)	0.32		5.71	(5.16,6.26)	0.28	
	None of these/Other	5.67	(5.26,6.07)	0.21		5.14	(4.61,5.68)	0.27		5.54	(5.06,6.01)	0.24	
Religious service attendance	More than 1/week	7.72	(7.41,8.03)	0.16	< .001	7.77	(7.40,8.15)	0.19	< .001	7.78	(7.45,8.12)	0.17	< .001
	1/week	7.17	(6.94,7.41)	0.12		7.21	(6.91,7.51)	0.15		7.24	(6.99,7.49)	0.13	
	1-3/month	6.98	(6.67,7.28)	0.15		6.77	(6.36,7.18)	0.21		6.92	(6.58,7.25)	0.17	
	A few times a year	6.79	(6.62,6.96)	0.09		6.77	(6.57,6.97)	0.10		7.04	(6.88,7.20)	0.08	
	Never	6.67	(6.58,6.76)	0.05		6.58	(6.47,6.69)	0.06		6.74	(6.65,6.84)	0.05	
Education	Up to 8 years	7.40	(6.50,8.30)	0.45	< .001	7.46	(6.64,8.28)	0.41	< .001	7.98	(7.31,8.64)	0.33	< .001
	9-15 years	6.64	(6.54,6.75)	0.05		6.58	(6.45,6.70)	0.06		6.79	(6.69,6.90)	0.05	

Variable	Category	Cantril's Ladder				Life Satisfaction				Happiness			
		Mean	95% CI	SE	Global p-value	Mean	95% CI	SE	Global p-value	Mean	95% CI	SE	Global p-value
	16+ years	7.01	(6.93,7.09)	0.04		6.94	(6.84,7.04)	0.05		6.99	(6.90,7.08)	0.05	
Immigration status	Born in this country	6.73	(6.65,6.82)	0.04	0.009	6.66	(6.55,6.76)	0.05	0.006	6.83	(6.74,6.92)	0.05	0.008
	Born in another country	6.95	(6.81,7.09)	0.07		6.93	(6.76,7.10)	0.09		7.06	(6.91,7.20)	0.07	
Religious affiliation	Christianity	7.05	(6.95,7.15)	0.05	< .001	7.02	(6.90,7.15)	0.07	< .001	7.17	(7.06,7.28)	0.06	< .001
	Islam	6.34	(5.35,7.33)	0.49		6.39	(5.13,7.65)	0.62		6.38	(5.39,7.37)	0.49	
	Hinduism	6.29	(5.18,7.41)	0.53		6.45	(5.42,7.49)	0.50		6.85	(6.08,7.62)	0.37	
	Buddhism	6.80	(6.25,7.34)	0.26		7.18	(6.50,7.85)	0.33		7.22	(6.66,7.77)	0.27	
	Judaism	6.79	(6.25,7.34)	0.25		7.08	(6.51,7.65)	0.26		6.83	(6.27,7.38)	0.25	
	Sikhism	5.76	*	*		6.09	*	*		5.80	*	*	
	Baha'i	8.53	*	*		8.55	*	*		8.77	*	*	
	Taoism	7.02	*	*		6.97	*	*		7.20	*	*	
	Primal, Animist, or Folk religion	6.20	(5.34,7.06)	0.38		6.34	(5.35,7.34)	0.44		5.95	(4.65,7.25)	0.58	
	Some other religion	6.86	(5.76,7.95)	0.53		6.95	(5.83,8.06)	0.54		6.96	(5.95,7.97)	0.49	
	No religion/Atheist /Agnostic	6.59	(6.49,6.70)	0.05		6.48	(6.35,6.60)	0.06		6.66	(6.56,6.77)	0.05	
Race/Ethnicity	Aboriginal	5.92	(4.83,7.00)	0.53	< .001	5.96	(4.68,7.25)	0.63	< .001	5.95	(4.73,7.17)	0.60	< .001
	Australian	6.77	(6.67,6.88)	0.05		6.80	(6.68,6.92)	0.06		6.94	(6.83,7.05)	0.05	
	Australian British/European	6.84	(6.71,6.97)	0.07		6.60	(6.43,6.77)	0.09		6.82	(6.68,6.96)	0.07	
	Chinese	6.66	(6.29,7.02)	0.18		6.46	(5.89,7.03)	0.29		6.83	(6.41,7.26)	0.21	
	Indian	6.71	(5.97,7.44)	0.36		6.76	(5.97,7.55)	0.39		6.75	(6.21,7.30)	0.27	
	Japanese	7.91	*	*		7.54	*	*		8.00	*	*	
	Malay	7.28	*	*		7.42	*	*		7.56	*	*	
	Sinhalese	8.00	*	*		6.39	*	*		7.00	*	*	
	Spanish	6.80	*	*		7.19	*	*		7.54	*	*	
	Sri Lankan Moor	7.33	*	*		8.39	*	*		7.65	*	*	
	Sri Lankan Tamil	7.31	*	*		6.56	*	*		6.10	*	*	
	Vietnamese	7.56	*	*		7.59	*	*		6.77	*	*	
	Russian	7.05	*	*		7.41	*	*		7.54	*	*	
	Samoan	5.00	*	*		2.44	*	*		5.22	*	*	

Variable	Category	Cantril's Ladder				Life Satisfaction				Happiness			
		Mean	95% CI	SE	Global p-value	Mean	95% CI	SE	Global p-value	Mean	95% CI	SE	Global p-value
	New Zealander	6.67	(6.11,7.23)	0.28		6.91	(6.22,7.60)	0.35		6.76	(6.14,7.39)	0.31	
	Other European	7.01	(6.82,7.21)	0.10		6.87	(6.62,7.12)	0.13		7.09	(6.87,7.31)	0.11	
	Other	6.43	(6.03,6.83)	0.20		6.45	(6.02,6.87)	0.22		6.56	(6.19,6.93)	0.19	

Table S3c. Childhood predictors regression analysis results for Australia

Variable	Category	Cantril's Ladder				Life Satisfaction				Happiness			
		Est	95% CI	SE	Global p-value	Est	95% CI	SE	Global p-value	Est	95% CI	SE	Global p-value
Relationship with mother	(Ref: Very bad/somewhat bad)				0.465				0.694				0.507
	Very good/somewhat good	0.11	(-0.19, 0.42)	0.16		-0.07	(-0.44, 0.29)	0.19		0.11	(-0.22, 0.44)	0.17	
Relationship with father	(Ref: Very bad/somewhat bad)				0.519				0.629				0.322
	Very good/somewhat good	0.09	(-0.18, 0.35)	0.13		0.06	(-0.25, 0.38)	0.16		0.13	(-0.13, 0.40)	0.13	
Parent marital status	(Ref: Parents married)				0.551				0.188				0.515
	Divorced	0.06	(-0.19, 0.32)	0.13		-0.12	(-0.42, 0.18)	0.15		0.02	(-0.25, 0.29)	0.14	
	Parents were never married	0.22	(-0.21, 0.64)	0.22		0.39	(-0.07, 0.86)	0.23		0.20	(-0.20, 0.60)	0.20	
	One or both parents had died	0.23	(-0.21, 0.66)	0.22		0.20	(-0.32, 0.71)	0.26		0.25	(-0.18, 0.67)	0.22	
Subjective financial status of family growing up	(Ref: Got by)				0.002				0.110				0.096
	Lived comfortably	0.18	(0.04, 0.33)	0.07		0.13	(-0.04, 0.31)	0.09		0.15	(-0.00, 0.29)	0.08	
	Found it difficult	0.11	(-0.13, 0.35)	0.12		0.10	(-0.18, 0.39)	0.15		0.03	(-0.22, 0.29)	0.13	
	Found it very difficult	-0.49	(-0.89, -0.10)	0.20		-0.40	(-0.90, 0.11)	0.26		-0.26	(-0.69, 0.16)	0.22	
Abuse	(Ref: No)				<.001				<.001				<.001
	Yes	-0.33	(-0.52, -0.15)	0.09		-0.45	(-0.67, -0.24)	0.11		-0.38	(-0.57, -0.19)	0.10	
Outsider growing up	(Ref: No)				<.001				<.001				<.001
	Yes	-0.72	(-0.94, -0.50)	0.11		-0.88	(-1.14, -0.62)	0.13		-0.61	(-0.84, -0.39)	0.11	
Self-rated health growing up	(Ref: Good)				<.001				<.001				<.001
	Excellent	0.45	(0.25, 0.65)	0.10		0.67	(0.42, 0.92)	0.13		0.57	(0.35, 0.78)	0.11	

Variable	Category	Cantril's Ladder				Life Satisfaction				Happiness			
		Est	95% CI	SE	Global p-value	Est	95% CI	SE	Global p-value	Est	95% CI	SE	Global p-value
	Very good	0.08	(-0.13, 0.30)	0.11		0.21	(-0.05, 0.47)	0.13		0.16	(-0.06, 0.39)	0.11	
	Fair	-0.27	(-0.60, 0.06)	0.17		-0.34	(-0.78, 0.09)	0.22		-0.17	(-0.53, 0.19)	0.18	
	Poor	-0.43	(-1.10, 0.23)	0.34		-0.33	(-1.02, 0.35)	0.35		-0.41	(-0.93, 0.11)	0.27	
Immigration status	(Ref: Born in this country)				0.422				0.068				0.090
	Born in another country	0.07	(-0.10, 0.25)	0.09		0.19	(-0.02, 0.40)	0.11		0.16	(-0.03, 0.34)	0.09	
Age 12 religious service attendance	(Ref: Never)				0.373				0.325				0.483
	At least 1/week	0.16	(-0.03, 0.35)	0.10		0.16	(-0.07, 0.40)	0.12		0.14	(-0.08, 0.35)	0.11	
	1-3/month	0.08	(-0.14, 0.31)	0.11		-0.04	(-0.33, 0.25)	0.15		0.09	(-0.16, 0.33)	0.12	
	< 1/month	0.12	(-0.11, 0.34)	0.11		0.08	(-0.19, 0.35)	0.14		0.01	(-0.22, 0.24)	0.12	
Year of birth	(Ref: 1998-2005; current age: 18-24)				<.001				<.001				<.001
	1993-1998; age 25-29	0.27	(-0.10, 0.64)	0.19		0.20	(-0.26, 0.66)	0.23		0.17	(-0.23, 0.57)	0.20	
	1983-1993; age 30-39	0.54	(0.24, 0.85)	0.15		0.47	(0.07, 0.86)	0.20		0.36	(0.03, 0.69)	0.17	
	1973-1983; age 40-49	0.50	(0.19, 0.81)	0.16		0.67	(0.28, 1.06)	0.20		0.54	(0.22, 0.87)	0.16	
	1963-1973; age 50-59	0.37	(0.06, 0.68)	0.16		0.46	(0.08, 0.84)	0.20		0.33	(0.00, 0.66)	0.17	
	1953-1963; age 60-69	0.80	(0.50, 1.10)	0.15		0.97	(0.59, 1.35)	0.19		0.91	(0.59, 1.23)	0.16	
	1943-1953; age 70-79	1.20	(0.88, 1.51)	0.16		1.44	(1.04, 1.83)	0.20		1.31	(0.98, 1.65)	0.17	
	1943 or earlier; age 80+	1.29	(0.91, 1.67)	0.19		1.67	(1.27, 2.06)	0.20		1.61	(1.27, 1.94)	0.17	
Gender	(Ref: Male)				0.563				0.060				0.161
	Female	0.03	(-0.11, 0.17)	0.07		0.19	(0.02, 0.35)	0.08		0.13	(-0.02, 0.27)	0.07	
	Other	0.30	(-0.27, 0.88)	0.29		-0.28	(-1.29, 0.74)	0.52		-0.19	(-0.91, 0.53)	0.37	
Religious affiliation	(Ref: No religion/Atheist /Agnostic)				0.112				0.653				0.371
	Christianity	0.15	(-0.05, 0.34)	0.10		0.09	(-0.15, 0.32)	0.12		0.07	(-0.13, 0.28)	0.10	

Variable	Category	Cantril's Ladder				Life Satisfaction				Happiness			
		Est	95% CI	SE	Global p-value	Est	95% CI	SE	Global p-value	Est	95% CI	SE	Global p-value
Race/ethnicity	Collapsed affiliations with prevalence<3%	-0.17	(-0.60, 0.25)	0.22		-0.07	(-0.57, 0.43)	0.25		-0.19	(-0.62, 0.24)	0.22	
	(Ref: Plurality group)				0.145				0.410				0.586
	Non-plurality groups	0.11	(-0.04, 0.26)	0.08		-0.07	(-0.26, 0.12)	0.10		-0.04	(-0.20, 0.13)	0.08	

Table S3d. Sensitivity to unmeasured confounding of childhood predictors in Australia

		Cantril's Ladder		Life Satisfaction		Happiness	
Variable	Category	E-value for Estimate	E-value for 95% CI	E-value for Estimate	E-value for 95% CI	E-value for Estimate	E-value for 95% CI
Relationship with mother	(Ref: Very bad/somewhat bad)						
	Very good/somewhat good	1.31	1.00	1.21	1.00	1.30	1.00
Relationship with father	(Ref: Very bad/somewhat bad)						
	Very good/somewhat good	1.26	1.00	1.20	1.00	1.34	1.00
Parent marital status	(Ref: Parents married)						
	Divorced	1.22	1.00	1.29	1.00	1.12	1.00
	Parents were never married	1.48	1.00	1.66	1.00	1.45	1.00
	One or both parents had died	1.49	1.00	1.40	1.00	1.52	1.00
Subjective financial status of family growing up	(Ref: Got by)						
	Lived comfortably	1.43	1.16	1.31	1.00	1.36	1.00
	Found it difficult	1.31	1.00	1.26	1.00	1.15	1.00
	Found it very difficult	1.90	1.28	1.66	1.00	1.54	1.00
Abuse	(Ref: No)						
	Yes	1.66	1.38	1.73	1.45	1.71	1.43
Outsider growing up	(Ref: No)						
	Yes	2.25	1.91	2.29	1.95	2.05	1.72
Self-rated health growing up	(Ref: Good)						
	Excellent	1.83	1.53	2.01	1.69	1.99	1.67
	Very good	1.26	1.00	1.41	1.00	1.39	1.00

Variable	Category	Cantril's Ladder E-value for Estimate	Cantril's Ladder E-value for 95% CI	Life Satisfaction E-value for Estimate	Life Satisfaction E-value for 95% CI	Happiness E-value for Estimate	Happiness E-value for 95% CI
	Fair	1.56	1.00	1.59	1.00	1.40	1.00
	Poor	1.80	1.00	1.58	1.00	1.75	1.00
Immigration status	(Ref: Born in this country)						
	Born in another country	1.23	1.00	1.39	1.00	1.38	1.00
Age 12 religious service attendance	(Ref: Never)						
	At least 1/week	1.39	1.00	1.35	1.00	1.34	1.00
	1-3/month	1.26	1.00	1.15	1.00	1.26	1.00
	< 1/month	1.32	1.00	1.23	1.00	1.08	1.00
Year of birth	(Ref: 1998-2005; current age: 18-24)						
	1993-1998; age 25-29	1.56	1.00	1.40	1.00	1.40	1.00
	1983-1993; age 30-39	1.97	1.52	1.75	1.21	1.69	1.15
	1973-1983; age 40-49	1.91	1.44	2.00	1.51	1.95	1.48
	1963-1973; age 50-59	1.71	1.22	1.74	1.22	1.65	1.06
	1953-1963; age 60-69	2.38	1.90	2.42	1.91	2.53	2.03
	1943-1953; age 70-79	3.10	2.52	3.13	2.51	3.27	2.65
	1943 or earlier; age 80+	3.28	2.56	3.53	2.86	3.90	3.19
Gender	(Ref: Male)						
	Female	1.14	1.00	1.39	1.11	1.33	1.00
	Other	1.61	1.00	1.50	1.00	1.43	1.00
Religious affiliation	(Ref: No religion/Atheist/Agnostic)						
	Christianity	1.37	1.00	1.24	1.00	1.23	1.00

		Cantril's Ladder		Life Satisfaction		Happiness	
Variable	Category	E-value for Estimate	E-value for 95% CI	E-value for Estimate	E-value for 95% CI	E-value for Estimate	E-value for 95% CI
Race/ethnicity	Collapsed affiliations with prevalence<3%	1.41	1.00	1.21	1.00	1.43	1.00
	(Ref: Plurality group)						
	Non-plurality groups	1.31	1.00	1.21	1.00	1.16	1.00

Table S3e. Complete-case supplemental analysis of means by demographic category for Australia

		Cantril's Ladder				Life Satisfaction				Happiness			
Variable	Category	Mean	95% CI	SE	Global p-value	Mean	95% CI	SE	Global p-value	Mean	95% CI	SE	Global p-value
Age group	18-24	6.09	(5.82,6.35)	0.14	< .001	5.91	(5.57,6.25)	0.17	< .001	6.13	(5.84,6.42)	0.15	< .001
	25-29	6.32	(6.04,6.61)	0.15		6.21	(5.90,6.52)	0.16		6.34	(6.04,6.64)	0.15	
	30-39	6.68	(6.50,6.86)	0.09		6.50	(6.28,6.73)	0.11		6.60	(6.41,6.80)	0.10	
	40-49	6.70	(6.53,6.88)	0.09		6.71	(6.50,6.91)	0.10		6.81	(6.64,6.97)	0.08	
	50-59	6.63	(6.46,6.79)	0.08		6.70	(6.55,6.86)	0.08		6.74	(6.57,6.90)	0.08	
	60-69	7.06	(6.91,7.20)	0.07		7.16	(7.01,7.32)	0.08		7.26	(7.12,7.40)	0.07	
	70-79	7.55	(7.39,7.72)	0.08		7.67	(7.48,7.86)	0.10		7.75	(7.58,7.92)	0.09	
	80 or older	7.67	(7.36,7.99)	0.16		7.93	(7.70,8.16)	0.12		8.07	(7.86,8.29)	0.11	
Gender	Female	6.81	(6.71,6.91)	0.05	0.148	6.88	(6.77,6.99)	0.06	0.007	6.97	(6.87,7.08)	0.05	0.003
	Male	6.83	(6.72,6.93)	0.05		6.79	(6.67,6.90)	0.06		6.88	(6.78,6.99)	0.05	
	Other	6.23	(5.62,6.84)	0.30		5.38	(4.38,6.38)	0.49		5.71	(4.95,6.48)	0.37	
Marital status	Divorced	6.48	(6.24,6.71)	0.12	< .001	6.59	(6.33,6.84)	0.13	< .001	6.69	(6.46,6.92)	0.12	< .001
	Domestic partner	6.60	(6.40,6.81)	0.10		6.61	(6.36,6.86)	0.13		6.63	(6.39,6.86)	0.12	
	Married	7.30	(7.22,7.39)	0.04		7.35	(7.25,7.44)	0.05		7.38	(7.29,7.46)	0.04	
	Separated	5.90	(5.47,6.32)	0.21		5.94	(5.52,6.36)	0.21		6.28	(5.89,6.66)	0.19	
	Single/Never been married	6.05	(5.88,6.22)	0.09		5.87	(5.68,6.06)	0.10		6.14	(5.96,6.31)	0.09	
	Widowed	7.31	(7.04,7.57)	0.14		7.44	(7.16,7.73)	0.14		7.55	(7.31,7.80)	0.12	
Employment	Employed for an employer	6.76	(6.67,6.85)	0.05	< .001	6.71	(6.60,6.81)	0.05	< .001	6.81	(6.72,6.91)	0.05	< .001
	Homemaker	6.58	(6.12,7.03)	0.23		6.62	(6.14,7.11)	0.24		6.71	(6.27,7.16)	0.22	
	None of these/Other	5.72	(5.31,6.12)	0.21		5.65	(5.18,6.11)	0.24		5.86	(5.44,6.28)	0.21	
	Retired	7.37	(7.24,7.51)	0.07		7.57	(7.43,7.71)	0.07		7.62	(7.50,7.75)	0.06	
	Self-employed	7.07	(6.87,7.28)	0.10		7.04	(6.79,7.29)	0.13		7.15	(6.94,7.35)	0.11	
	Student	6.27	(5.92,6.61)	0.17		6.24	(5.84,6.64)	0.20		6.22	(5.86,6.58)	0.18	
	Unemployed and looking for a job	5.65	(5.15,6.16)	0.26		5.29	(4.70,5.89)	0.30		5.71	(5.16,6.26)	0.28	
Religious service attendance	A few times a year	6.83	(6.67,6.99)	0.08	< .001	6.87	(6.68,7.05)	0.09	< .001	7.08	(6.92,7.23)	0.08	< .001
	More than once a week	7.72	(7.40,8.03)	0.16		7.77	(7.39,8.15)	0.19		7.77	(7.44,8.11)	0.17	
	Never	6.70	(6.61,6.78)	0.05		6.69	(6.59,6.79)	0.05		6.78	(6.69,6.87)	0.05	
	Once a week	7.20	(6.97,7.43)	0.12		7.34	(7.08,7.60)	0.13		7.31	(7.09,7.53)	0.11	

		Cantril's Ladder				Life Satisfaction				Happiness			
Variable	Category	Mean	95% CI	SE	Global p-value	Mean	95% CI	SE	Global p-value	Mean	95% CI	SE	Global p-value
	One to three times a month	7.00	(6.70,7.30)	0.15		6.81	(6.40,7.22)	0.21		6.92	(6.59,7.26)	0.17	
Education	Up to 8 years	7.40	(6.50,8.30)	0.45	< .001	7.47	(6.64,8.30)	0.42	0.002	7.98	(7.31,8.65)	0.33	< .001
	16+ years	7.01	(6.93,7.10)	0.04		6.97	(6.87,7.07)	0.05		7.00	(6.91,7.09)	0.05	
	9 to 15 years	6.68	(6.58,6.78)	0.05		6.72	(6.60,6.83)	0.06		6.84	(6.74,6.95)	0.05	
Immigration status	Born in another country	6.96	(6.82,7.10)	0.07	0.017	7.05	(6.91,7.19)	0.07	< .001	7.08	(6.94,7.22)	0.07	0.010
	Born in this country	6.77	(6.68,6.85)	0.04		6.75	(6.65,6.85)	0.05		6.87	(6.78,6.95)	0.04	
Religious affiliation	Buddhism	6.80	(6.25,7.35)	0.26	< .001	7.18	(6.50,7.85)	0.33	< .001	7.21	(6.65,7.77)	0.27	< .001
	Christianity	7.06	(6.96,7.16)	0.05		7.10	(6.98,7.22)	0.06		7.20	(7.10,7.31)	0.05	
	Hinduism	6.27	(5.23,7.31)	0.50		6.51	(5.51,7.50)	0.48		6.93	(6.21,7.65)	0.35	
	Islam	6.71	(5.96,7.45)	0.36		6.76	(5.65,7.87)	0.54		6.74	(5.99,7.49)	0.37	
	Judaism	6.79	(6.24,7.34)	0.25		7.07	(6.49,7.64)	0.26		6.81	(6.25,7.37)	0.26	
	No religion/Atheist/Agnostic	6.63	(6.53,6.73)	0.05		6.59	(6.48,6.71)	0.06		6.70	(6.60,6.80)	0.05	
	Primal, Animist, or Folk religion	6.20	(5.34,7.06)	0.38		6.34	(5.35,7.34)	0.44		5.94	(4.64,7.25)	0.58	
	Sikhism	5.76	*	*		6.09	*	*		5.80	*	*	
	Some other religion	6.91	(5.81,8.02)	0.54		7.01	(5.89,8.13)	0.54		6.96	(5.95,7.97)	0.49	
	Taoism	7.02	*	*		6.97	*	*		7.20	*	*	
	Baha'i	8.53	*	*		8.56	*	*		8.77	*	*	

Table S3f. Complete-case supplemental analysis of childhood predictors regression analysis results for Australia

		Cantril's Ladder				Life Satisfaction				Happiness			
Variable	Category	Est	95% CI	SE	Global p-value	Est	95% CI	SE	Global p-value	Est	95% CI	SE	Global p-value
Relationship with mother	(Ref: Very bad/somewhat bad)				0.348				0.464				0.782
	Very good/somewhat good	0.14	(-0.15,0.43)	0.15		-0.12	(-0.46,0.21)	0.17		0.04	(-0.26,0.35)	0.16	
Relationship with father	(Ref: Very bad/somewhat bad)				0.893				0.671				0.529
	Very good/somewhat good	0.02	(-0.23,0.27)	0.13		0.06	(-0.22,0.34)	0.14		0.08	(-0.17,0.32)	0.12	
Parent marital status	(Ref: Parents married)				0.469				0.213				0.635
	No, one or both of them had died	0.14	(-0.30,0.58)	0.22		0.15	(-0.39,0.69)	0.27		0.16	(-0.27,0.60)	0.22	
	No, they were never married	0.20	(-0.23,0.63)	0.22		0.50	(0.04,0.96)	0.24		0.13	(-0.29,0.56)	0.22	
	Yes, married	-0.07	(-0.31,0.17)	0.12		0.14	(-0.13,0.42)	0.14		-0.04	(-0.29,0.21)	0.13	
Subjective financial status of family growing up	(Ref: Got by)				0.003				0.070				0.132
	Found it difficult	0.08	(-0.15,0.32)	0.12		0.14	(-0.10,0.39)	0.13		0.00	(-0.24,0.24)	0.12	
	Found it very difficult	-0.55	(-0.95,-0.16)	0.20		-0.48	(-0.97,0.00)	0.25		-0.34	(-0.75,0.07)	0.21	
	Lived comfortably	0.15	(0.01,0.29)	0.07		0.09	(-0.07,0.25)	0.08		0.11	(-0.04,0.25)	0.07	
Abuse	(Ref: No)				<.001				<.001				<.001
	Yes	-0.36	(-0.54,-0.18)	0.09		-0.43	(-0.64,-0.23)	0.10		-0.39	(-0.57,-0.21)	0.09	
Outsider growing up	(Ref: No)				<.001				<.001				<.001
	Yes	-0.62	(-0.83,-0.42)	0.10		-0.70	(-0.93,-0.46)	0.12		-0.51	(-0.72,-0.29)	0.11	

		Cantril's Ladder				Life Satisfaction				Happiness			
Variable	Category	Est	95% CI	SE	Global p-value	Est	95% CI	SE	Global p-value	Est	95% CI	SE	Global p-value
Self-rated health growing up	(Ref: Good)				<.001				<.001				<.001
	Excellent	0.47	(0.27,0.67)	0.10		0.60	(0.38,0.82)	0.11		0.55	(0.35,0.75)	0.10	
	Fair	-0.27	(-0.60,0.05)	0.17		-0.31	(-0.69,0.07)	0.19		-0.19	(-0.53,0.15)	0.17	
	Poor	-0.39	(-1.05,0.28)	0.34		-0.41	(-1.08,0.26)	0.34		-0.42	(-0.93,0.10)	0.26	
	Very good	0.12	(-0.09,0.33)	0.11		0.15	(-0.08,0.38)	0.12		0.16	(-0.05,0.36)	0.11	
Immigration status	(Ref: Born in this country)				0.272				0.198				0.501
	Born in another country	0.11	(-0.08,0.30)	0.10		0.13	(-0.07,0.33)	0.10		0.07	(-0.13,0.26)	0.10	
Age 12 religious service attendance	(Ref: Never)				0.212				0.162				0.490
	At least once a week	0.20	(0.01,0.39)	0.10		0.17	(-0.04,0.37)	0.10		0.12	(-0.06,0.31)	0.10	
	Less than once a month	0.15	(-0.07,0.36)	0.11		0.07	(-0.17,0.31)	0.12		-0.01	(-0.22,0.21)	0.11	
	One to three times a month	0.14	(-0.08,0.35)	0.11		-0.08	(-0.34,0.18)	0.13		0.05	(-0.18,0.28)	0.12	
Year of birth	(Ref: 1998-2005; current age: 18-24)				<.001				<.001				<.001
	1993-1998; age 25-29	0.26	(-0.11,0.63)	0.19		0.30	(-0.13,0.73)	0.22		0.20	(-0.19,0.59)	0.20	
	1983-1993; age 30-39	0.56	(0.26,0.86)	0.15		0.55	(0.17,0.93)	0.19		0.40	(0.08,0.73)	0.16	
	1973-1983; age 40-49	0.55	(0.25,0.85)	0.15		0.73	(0.36,1.11)	0.19		0.58	(0.26,0.89)	0.16	
	1963-1973; age 50-59	0.41	(0.11,0.71)	0.15		0.61	(0.25,0.98)	0.19		0.43	(0.11,0.75)	0.16	
	1953-1963; age 60-69	0.82	(0.52,1.11)	0.15		1.05	(0.68,1.43)	0.19		0.95	(0.63,1.26)	0.16	
	1943-1953; age 70-79	1.22	(0.91,1.53)	0.16		1.47	(1.08,1.87)	0.20		1.36	(1.03,1.69)	0.17	

		Cantril's Ladder				Life Satisfaction				Happiness			
Variable	Category	Est	95% CI	SE	Global p-value	Est	95% CI	SE	Global p-value	Est	95% CI	SE	Global p-value
	1943 or earlier; age 80+	1.29	(0.91,1.67)	0.19		1.71	(1.31,2.10)	0.20		1.65	(1.31,1.98)	0.17	
Gender	(Ref: Male)				0.505				0.080				0.091
	Male	-0.04	(-0.17,0.09)	0.07		-0.16	(-0.31,-0.00)	0.08		-0.14	(-0.28,-0.01)	0.07	
	Other	0.28	(-0.29,0.84)	0.29		-0.55	(-1.52,0.42)	0.49		-0.32	(-1.01,0.37)	0.35	
Religious affiliation	(Ref: No religion/Atheist/Agnostic)				0.282				0.608				0.423
	Christianity	0.12	(-0.07,0.30)	0.10		0.07	(-0.13,0.28)	0.11		0.09	(-0.10,0.28)	0.10	
	Christianity	0.12	(-0.07,0.30)	0.10		0.07	(-0.13,0.28)	0.11		0.09	(-0.10,0.28)	0.10	
	Collapsed affiliations with prevalence<3%	-0.11	(-0.50,0.29)	0.20		-0.10	(-0.56,0.36)	0.24		-0.11	(-0.50,0.29)	0.20	
Race/ethnicity	(Ref: Plurality group)				0.952				0.332				0.284
	Non-plurality groups	-0.01	(-0.21,0.20)	0.11		0.11	(-0.11,0.33)	0.11		0.11	(-0.09,0.31)	0.10	

Table S3g. Complete-case supplemental analysis of sensitivity to unmeasured confounding of childhood predictors in Australia

Variable	Category	Cantril's Ladder		Life Satisfaction		Happiness	
		E-value for Estimate	E-value for 95% CI	E-value for Estimate	E-value for 95% CI	E-value for Estimate	E-value for 95% CI
Relationship with mother	(Ref: Very bad/somewhat bad)						
	Very good/somewhat good	1.36	1.00	1.31	1.00	1.18	1.00
Relationship with father	(Ref: Very bad/somewhat bad)						
	Very good/somewhat good	1.10	1.00	1.20	1.00	1.25	1.00
Parent marital status	(Ref: Parents married)						
	No, one or both of them had died	1.36	1.00	1.35	1.00	1.40	1.00
	No, they were never married	1.47	1.00	1.84	1.15	1.35	1.00
	Yes, married	1.23	1.00	1.34	1.00	1.17	1.00
Subjective financial status of family growing up	(Ref: Got by)						
	Found it difficult	1.26	1.00	1.34	1.00	1.05	1.00
	Found it very difficult	2.01	1.39	1.82	1.00	1.67	1.00
	Lived comfortably	1.38	1.08	1.26	1.00	1.30	1.00
Abuse	(Ref: No)						
	Yes	1.71	1.44	1.75	1.47	1.75	1.47
Outsider growing up	(Ref: No)						
	Yes	2.12	1.80	2.12	1.79	1.93	1.60
Self-rated health growing up	(Ref: Good)						
	Excellent	1.89	1.58	1.97	1.68	2.00	1.69
	Fair	1.58	1.00	1.58	1.00	1.44	1.00
	Poor	1.75	1.00	1.72	1.00	1.79	1.00
	Very good	1.33	1.00	1.35	1.00	1.39	1.00
Immigration status	(Ref: Born in this country)						
	Born in another country	1.31	1.00	1.32	1.00	1.22	1.00
Age 12 religious service attendance	(Ref: Never)						
	At least once a week	1.47	1.09	1.38	1.00	1.33	1.00
	Less than once a month	1.38	1.00	1.22	1.00	1.06	1.00
	One to three times a month	1.36	1.00	1.23	1.00	1.18	1.00
Year of birth	(Ref: 1998-2005; current age: 18-24)						

Variable	Category	Cantril's Ladder		Life Satisfaction		Happiness	
		E-value for Estimate	E-value for 95% CI	E-value for Estimate	E-value for 95% CI	E-value for Estimate	E-value for 95% CI
	1993-1998; age 25-29	1.56	1.00	1.57	1.00	1.45	1.00
	1983-1993; age 30-39	2.03	1.56	1.90	1.38	1.77	1.26
	1973-1983; age 40-49	2.00	1.54	2.17	1.65	2.04	1.56
	1963-1973; age 50-59	1.79	1.31	1.99	1.49	1.81	1.30
	1953-1963; age 60-69	2.45	1.96	2.65	2.09	2.65	2.13
	1943-1953; age 70-79	3.21	2.61	3.39	2.69	3.46	2.80
	1943 or earlier; age 80+	3.35	2.61	3.86	3.09	4.13	3.37
Gender	(Ref: Male)						
	Male	1.17	1.00	1.36	1.05	1.36	1.06
	Other	1.58	1.00	1.91	1.00	1.64	1.00
Religious affiliation	(Ref: No religion/Atheist/Agnostic)						
	Christianity	1.32	1.00	1.23	1.00	1.27	1.00
	Christianity	1.32	1.00	1.23	1.00	1.27	1.00
	Collapsed affiliations with prevalence<3%	1.30	1.00	1.27	1.00	1.30	1.00
Race/ethnicity	(Ref: Plurality group)						
	Non-plurality groups	1.06	1.00	1.29	1.00	1.31	1.00

Tables S4a-g: Brazil

Table S4a. Nationally representative descriptive statistics for Brazil

Characteristic	N = 13,204[1]
**Age group**	
1998-2005; age 18-24	1,986 (15%)
1993-1998; age 25-29	1,468 (11%)
1983-1993; age 30-39	2,908 (22%)
1973-1983; age 40-49	2,638 (20%)
1963-1973; age 50-59	2,131 (16%)
1953-1963; age 60-69	1,435 (11%)
1943-1953; age 70-79	510 (3.9%)
1943 or earlier; age 80+	126 (1.0%)
(Missing)	0 (0%)
**Gender**	
Male	6,320 (48%)
Female	6,820 (52%)
Other	35 (0.3%)
(Missing)	30 (0.2%)
**Race/Ethnicity**	
Amarela	238 (1.8%)
Branca	5,169 (39%)
Indígena	131 (1.0%)
Other	61 (0.5%)
Parda	5,125 (39%)
Preta	1,615 (12%)
(Missing)	865 (6.6%)
**Respondent Marital status**	
Married	4,646 (35%)
Separated	594 (4.5%)
Divorced	865 (6.5%)
Widowed	408 (3.1%)
Single, never married	4,347 (33%)
Domestic Partner	2,081 (16%)
(Missing)	263 (2.0%)
**Employment**	
Employed for an employer	3,756 (28%)
Self-employed	2,918 (22%)
Retired	1,536 (12%)
Student	624 (4.7%)
Homemaker	1,305 (9.9%)
Unemployed and looking for a job	2,419 (18%)
None of these/Other	448 (3.4%)
(Missing)	199 (1.5%)
**Religious service attendance as an adult (now)**	
More than 1/week	2,386 (18%)
1/week	2,272 (17%)
1-3/month	1,398 (11%)
A few times a year	3,978 (30%)
Never	3,110 (24%)
(Missing)	61 (0.5%)
**Education (years)**	
Up to 8 years	3,139 (24%)
9-15 years	7,665 (58%)
16+ years	2,390 (18%)
(Missing)	10 (<0.1%)
**Immigration status**	
Born in this country	12,688 (96%)
Born in another country	153 (1.2%)
(Missing)	363 (2.7%)
**Religious affiliation as an adult (now)**	
Christianity	9,911 (75%)
Islam	6 (<0.1%)
Hinduism	1 (<0.1%)
Buddhism	37 (0.3%)

Characteristic	N = 13,204[1]
Judaism	31 (0.2%)
Sikhism	0 (0%)
Baha'i	2 (<0.1%)
Jainism	2 (<0.1%)
Shinto	1 (<0.1%)
Taoism	1 (<0.1%)
Confucianism	6 (<0.1%)
Primal, Animist, or Folk religion	15 (0.1%)
Spiritism	696 (5.3%)
Umbanda, Candomble, and other African-derived religions	525 (4.0%)
Chinese folk/traditional religion	0 (0%)
Some other religion	144 (1.1%)
No religion/Atheist/Agnostic	1,712 (13%)
(Missing)	113 (0.9%)
**Relationship with mother growing up**	
Very good	8,369 (63%)
Somewhat good	3,559 (27%)
Somewhat bad	483 (3.7%)
Very bad	214 (1.6%)
Does not apply	507 (3.8%)
(Missing)	73 (0.6%)
**Relationship with father growing up**	
Very good	6,364 (48%)
Somewhat good	3,654 (28%)
Somewhat bad	1,035 (7.8%)
Very bad	756 (5.7%)
Does not apply	1,303 (9.9%)
(Missing)	93 (0.7%)
**Parent marital status at age 12**	
Parents married	8,546 (65%)
Divorced	1,384 (10%)
Parents were never married	1,985 (15%)
One or both parents had died	508 (3.8%)
(Missing)	781 (5.9%)
**Subjective financial status of family growing up**	
Lived comfortably	4,998 (38%)
Got by	4,616 (35%)
Found it difficult	2,484 (19%)
Found it very difficult	1,027 (7.8%)
(Missing)	79 (0.6%)
**Abuse**	
Yes	2,606 (20%)
No	10,147 (77%)
(Missing)	451 (3.4%)
**Outsider growing up**	
Yes	1,659 (13%)
No	11,234 (85%)
(Missing)	311 (2.4%)
**Self-rated health growing up**	
Excellent	5,312 (40%)
Very good	3,392 (26%)
Good	2,873 (22%)
Fair	1,368 (10%)
Poor	228 (1.7%)
(Missing)	30 (0.2%)
**Age 12 religious service attendance**	
At least 1/week	6,306 (48%)
1-3/month	2,491 (19%)
<1/month	2,629 (20%)
Never	1,707 (13%)
(Missing)	71 (0.5%)
**Religious affiliation at age 12**	
Christianity	11,403 (86%)
Islam	15 (0.1%)
Hinduism	1 (<0.1%)

Characteristic	N = 13,204[1]
Buddhism	27 (0.2%)
Judaism	40 (0.3%)
Sikhism	0 (0%)
Baha'i	1 (<0.1%)
Jainism	4 (<0.1%)
Shinto	4 (<0.1%)
Taoism	1 (<0.1%)
Confucianism	7 (<0.1%)
Primal, Animist, or Folk religion	17 (0.1%)
Spiritism	336 (2.5%)
Umbanda, Candomble, and other African-derived religions	262 (2.0%)
Chinese folk/traditional religion	0 (0%)
Some other religion	87 (0.7%)
No religion/Atheist/Agnostic	908 (6.9%)
(Missing)	94 (0.7%)

[1]n (%)

Table S4b. Means by demographic category for Brazil

Variable	Category	Cantril's Ladder Mean	95% CI	SE	Global p-value	Life Satisfaction Mean	95% CI	SE	Global p-value	Happiness Mean	95% CI	SE	Global p-value
Age group	18-24	6.28	(6.17,6.40)	0.06	< .001	6.64	(6.50,6.77)	0.07	< .001	7.03	(6.91,7.15)	0.06	< .001
	25-29	6.31	(6.16,6.47)	0.08		6.87	(6.70,7.03)	0.08		7.11	(6.95,7.27)	0.08	
	30-39	6.56	(6.47,6.66)	0.05		7.14	(7.04,7.25)	0.05		7.33	(7.23,7.43)	0.05	
	40-49	6.64	(6.53,6.75)	0.06		7.20	(7.07,7.32)	0.06		7.32	(7.21,7.44)	0.06	
	50-59	6.56	(6.42,6.71)	0.07		7.28	(7.13,7.42)	0.08		7.37	(7.23,7.52)	0.08	
	60-69	7.05	(6.87,7.24)	0.09		7.47	(7.26,7.68)	0.11		7.68	(7.51,7.85)	0.09	
	70-79	7.17	(6.80,7.54)	0.19		8.22	(7.90,8.53)	0.16		7.99	(7.69,8.28)	0.15	
	80 or older	7.12	(6.30,7.93)	0.41		7.94	(7.23,8.65)	0.36		7.87	(7.17,8.57)	0.35	
Gender	Male	6.62	(6.54,6.70)	0.04	0.261	7.27	(7.19,7.36)	0.04	< .001	7.42	(7.34,7.49)	0.04	0.011
	Female	6.57	(6.49,6.64)	0.04		7.04	(6.96,7.12)	0.04		7.26	(7.19,7.33)	0.04	
	Other	6.01	(5.07,6.95)	0.46		6.70	(5.51,7.89)	0.58		6.94	(5.85,8.02)	0.53	
Marital status	Married	7.06	(6.97,7.15)	0.04	< .001	7.71	(7.63,7.80)	0.04	< .001	7.83	(7.75,7.91)	0.04	< .001
	Separated	5.80	(5.51,6.09)	0.15		6.47	(6.18,6.77)	0.15		6.74	(6.45,7.03)	0.15	
	Divorced	6.50	(6.29,6.71)	0.11		6.93	(6.69,7.17)	0.12		7.26	(7.06,7.46)	0.10	
	Widowed	7.06	(6.67,7.45)	0.20		7.51	(7.09,7.93)	0.21		7.66	(7.31,8.01)	0.18	
	Never	6.24	(6.15,6.33)	0.05		6.71	(6.61,6.81)	0.05		6.92	(6.82,7.02)	0.05	
	Domestic Partner	6.44	(6.33,6.56)	0.06		7.06	(6.92,7.19)	0.07		7.24	(7.12,7.36)	0.06	
Employment	Employed for an employer	6.83	(6.76,6.91)	0.04	< .001	7.38	(7.29,7.47)	0.04	< .001	7.51	(7.43,7.59)	0.04	< .001
	Self-employed	6.66	(6.56,6.76)	0.05		7.25	(7.14,7.36)	0.05		7.44	(7.33,7.54)	0.05	
	Retired	7.18	(6.99,7.37)	0.10		7.79	(7.60,7.98)	0.10		7.82	(7.66,7.99)	0.09	
	Student	6.59	(6.41,6.78)	0.09		6.98	(6.75,7.20)	0.11		7.23	(7.03,7.44)	0.10	
	Homemaker	6.60	(6.41,6.78)	0.10		7.18	(6.97,7.38)	0.10		7.40	(7.22,7.58)	0.09	
	Unemployed and looking for a job	5.78	(5.64,5.92)	0.07		6.34	(6.19,6.49)	0.08		6.65	(6.51,6.79)	0.07	
	None of these/Other	6.44	(6.12,6.77)	0.17		7.01	(6.68,7.35)	0.17		7.19	(6.87,7.51)	0.16	
Religious service attendance	More than 1/week	7.12	(6.99,7.26)	0.07	< .001	7.79	(7.65,7.92)	0.07	< .001	7.96	(7.84,8.09)	0.06	< .001
	1/week	6.77	(6.64,6.90)	0.07		7.38	(7.25,7.52)	0.07		7.56	(7.44,7.68)	0.06	
	1-3/month	6.51	(6.35,6.67)	0.08		7.17	(7.00,7.34)	0.09		7.34	(7.18,7.49)	0.08	
	A few times a year	6.50	(6.42,6.58)	0.04		7.10	(7.01,7.19)	0.05		7.25	(7.17,7.34)	0.04	
	Never	6.21	(6.10,6.32)	0.06		6.56	(6.43,6.68)	0.06		6.79	(6.67,6.91)	0.06	
Education	Up to 8 years	6.53	(6.40,6.67)	0.07	< .001	7.34	(7.20,7.48)	0.07	< .001	7.48	(7.35,7.61)	0.07	< .001
	9-15 years	6.48	(6.42,6.55)	0.03		7.02	(6.95,7.09)	0.04		7.24	(7.17,7.31)	0.03	

Variable	Category	Cantril's Ladder				Life Satisfaction				Happiness			
		Mean	95% CI	SE	Global p-value	Mean	95% CI	SE	Global p-value	Mean	95% CI	SE	Global p-value
	16+ years	7.02	(6.93,7.11)	0.05		7.33	(7.23,7.43)	0.05		7.44	(7.34,7.54)	0.05	
Immigration status	Born in this country	6.58	(6.53,6.64)	0.03	0.018	7.15	(7.09,7.21)	0.03	0.508	7.33	(7.28,7.38)	0.03	0.466
	Born in another country	7.09	(6.66,7.51)	0.22		7.29	(6.74,7.83)	0.27		7.50	(7.04,7.97)	0.24	
Religious affiliation	Christianity	6.68	(6.62,6.74)	0.03	< .001	7.31	(7.25,7.38)	0.03	< .001	7.48	(7.42,7.54)	0.03	< .001
	Islam	7.80	*	*		6.77	*	*		7.19	*	*	
	Hinduism	6.23	*	*		4.24	*	*		4.93	*	*	
	Buddhism	6.92	(6.13,7.71)	0.38		6.79	(5.90,7.68)	0.43		7.24	(6.42,8.06)	0.39	
	Judaism	6.42	(5.56,7.28)	0.40		5.28	(3.26,7.31)	0.94		6.01	(3.93,8.09)	0.96	
	Baha'i	10.00	*	*		10.00	*	*		9.65	*	*	
	Jainism	5.89	*	*		3.83	*	*		6.89	*	*	
	Shinto	7.33	*	*		6.34	*	*		6.33	*	*	
	Taoism	7.08	*	*		7.50	*	*		7.55	*	*	
	Confucianism	7.83	*	*		7.31	*	*		7.48	*	*	
	Primal, Animist, or Folk religion	7.58	*	*		7.14	*	*		8.01	*	*	
	Spiritism	6.56	(6.33,6.79)	0.12		7.01	(6.76,7.26)	0.13		7.14	(6.90,7.37)	0.12	
	Umbanda, Candomble, and other African-derived religions	6.31	(6.07,6.54)	0.12		6.70	(6.43,6.98)	0.14		6.95	(6.67,7.23)	0.14	
	Some other religion	6.56	(6.08,7.04)	0.24		7.10	(6.51,7.68)	0.30		6.90	(6.37,7.42)	0.27	
	No religion/Atheist /Agnostic	6.14	(6.00,6.28)	0.07		6.46	(6.30,6.62)	0.08		6.76	(6.62,6.90)	0.07	
Race/Ethnicity	Branca	6.70	(6.62,6.78)	0.04	0.003	7.17	(7.08,7.26)	0.05	0.070	7.33	(7.25,7.42)	0.04	0.049
	Preta	6.50	(6.36,6.65)	0.07		7.05	(6.90,7.21)	0.08		7.41	(7.26,7.56)	0.07	
	Parda	6.54	(6.46,6.62)	0.04		7.20	(7.11,7.29)	0.04		7.35	(7.27,7.43)	0.04	
	Amarela	6.11	(5.68,6.53)	0.21		6.69	(6.24,7.15)	0.23		6.73	(6.26,7.20)	0.24	
	Indígena	6.26	(5.66,6.87)	0.30		6.70	(6.07,7.32)	0.31		6.97	(6.30,7.64)	0.33	
	Other	6.84	(5.97,7.72)	0.44		7.35	(6.60,8.09)	0.37		6.96	(5.81,8.12)	0.58	

Table S4c. Childhood predictors regression analysis results for Brazil

		Cantril's Ladder				Life Satisfaction				Happiness			
Variable	Category	Est	95% CI	SE	Global p-value	Est	95% CI	SE	Global p-value	Est	95% CI	SE	Global p-value
Relationship with mother	(Ref: Very bad/somewhat bad)				0.058				0.088				0.019
	Very good/somewhat good	0.22	(-0.01, 0.45)	0.12		0.21	(-0.05, 0.48)	0.14		0.28	(0.04, 0.52)	0.12	
Relationship with father	(Ref: Very bad/somewhat bad)				<.001				<.001				<.001
	Very good/somewhat good	0.37	(0.22, 0.51)	0.07		0.57	(0.40, 0.73)	0.08		0.46	(0.31, 0.62)	0.08	
Parent marital status	(Ref: Parents married)				0.025				0.478				0.269
	Divorced	-0.14	(-0.30, 0.02)	0.08		-0.04	(-0.20, 0.12)	0.08		-0.11	(-0.27, 0.06)	0.08	
	Parents were never married	-0.23	(-0.39, -0.06)	0.08		-0.10	(-0.27, 0.08)	0.09		0.03	(-0.14, 0.20)	0.09	
	One or both parents had died	-0.14	(-0.46, 0.18)	0.16		-0.21	(-0.56, 0.13)	0.18		-0.18	(-0.49, 0.13)	0.16	
Subjective financial status of family growing up	(Ref: Got by)				<.001				<.001				<.001
	Lived comfortably	0.44	(0.32, 0.55)	0.06		0.30	(0.18, 0.43)	0.06		0.28	(0.17, 0.40)	0.06	
	Found it difficult	-0.19	(-0.35, -0.04)	0.08		-0.06	(-0.23, 0.11)	0.09		-0.09	(-0.24, 0.06)	0.08	
	Found it very difficult	-0.31	(-0.57, -0.06)	0.13		-0.02	(-0.29, 0.25)	0.14		-0.24	(-0.50, 0.02)	0.13	
Abuse	(Ref: No)				<.001				<.001				<.001
	Yes	-0.23	(-0.36, -0.09)	0.07		-0.48	(-0.63, -0.33)	0.08		-0.38	(-0.52, -0.24)	0.07	
Outsider growing up	(Ref: No)				0.064				<.001				<.001
	Yes	-0.15	(-0.30, 0.01)	0.08		-0.57	(-0.76, -0.39)	0.09		-0.55	(-0.71, -0.39)	0.08	
Self-rated health growing up	(Ref: Good)				<.001				<.001				<.001
	Excellent	0.26	(0.12, 0.40)	0.07		0.31	(0.17, 0.45)	0.07		0.41	(0.27, 0.54)	0.07	

		Cantril's Ladder				Life Satisfaction				Happiness			
Variable	Category	Est	95% CI	SE	Global p-value	Est	95% CI	SE	Global p-value	Est	95% CI	SE	Global p-value
	Very good	0.07	(-0.07, 0.21)	0.07		0.00	(-0.15, 0.15)	0.08		0.07	(-0.07, 0.22)	0.07	
	Fair	-0.42	(-0.63, -0.20)	0.11		-0.47	(-0.70, -0.24)	0.12		-0.33	(-0.54, -0.11)	0.11	
	Poor	-0.57	(-1.08, -0.07)	0.26		-0.75	(-1.33, -0.17)	0.30		-0.18	(-0.67, 0.30)	0.25	
Immigration status	(Ref: Born in this country)				0.067				0.806				0.627
	Born in another country	0.37	(-0.04, 0.78)	0.21		0.02	(-0.47, 0.52)	0.25		0.10	(-0.33, 0.54)	0.22	
Age 12 religious service attendance	(Ref: Never)				0.164				0.139				0.036
	At least 1/week	0.19	(0.01, 0.38)	0.10		0.24	(0.03, 0.44)	0.11		0.26	(0.07, 0.46)	0.10	
	1-3/month	0.13	(-0.08, 0.34)	0.11		0.23	(-0.00, 0.45)	0.12		0.19	(-0.02, 0.40)	0.11	
	< 1/month	0.10	(-0.10, 0.30)	0.10		0.17	(-0.05, 0.39)	0.11		0.15	(-0.05, 0.36)	0.10	
Year of birth	(Ref: 1998-2005; current age: 18-24)				<.001				<.001				<.001
	1993-1998; age 25-29	0.03	(-0.15, 0.22)	0.09		0.18	(-0.03, 0.38)	0.10		0.03	(-0.16, 0.22)	0.10	
	1983-1993; age 30-39	0.29	(0.14, 0.44)	0.08		0.43	(0.27, 0.60)	0.08		0.24	(0.09, 0.39)	0.08	
	1973-1983; age 40-49	0.34	(0.18, 0.50)	0.08		0.45	(0.28, 0.63)	0.09		0.23	(0.06, 0.39)	0.08	
	1963-1973; age 50-59	0.22	(0.04, 0.41)	0.09		0.48	(0.28, 0.68)	0.10		0.24	(0.04, 0.43)	0.10	
	1953-1963; age 60-69	0.69	(0.47, 0.91)	0.11		0.65	(0.40, 0.90)	0.13		0.53	(0.31, 0.74)	0.11	
	1943-1953; age 70-79	0.75	(0.36, 1.14)	0.20		1.40	(1.06, 1.74)	0.17		0.84	(0.53, 1.15)	0.16	
	1943 or earlier; age 80+	0.78	(-0.02, 1.58)	0.41		1.13	(0.40, 1.86)	0.37		0.77	(0.04, 1.50)	0.37	
Gender	(Ref: Male)				0.013				0.810				0.331
	Female	0.15	(0.04, 0.25)	0.05		0.03	(-0.08, 0.15)	0.06		0.08	(-0.03, 0.18)	0.05	
	Other	-0.37	(-1.37, 0.64)	0.51		-0.11	(-1.21, 0.99)	0.56		-0.10	(-1.22, 1.03)	0.57	
Religious affiliation	(Ref: No religion/Atheist /Agnostic)				0.482				0.339				0.768
	Christianity	0.06	(-0.18, 0.30)	0.12		0.11	(-0.13, 0.34)	0.12		-0.05	(-0.27, 0.17)	0.11	

		Cantril's Ladder				Life Satisfaction				Happiness			
Variable	Category	Est	95% CI	SE	Global p-value	Est	95% CI	SE	Global p-value	Est	95% CI	SE	Global p-value
	Collapsed affiliations with prevalence<3%	0.17	(-0.14, 0.47)	0.15		-0.03	(-0.35, 0.28)	0.16		-0.11	(-0.40, 0.19)	0.15	
Race/ethnicity	(Ref: Plurality group)				0.424				<.001				<.001
	Non-plurality groups	0.04	(-0.07, 0.15)	0.06		0.21	(0.09, 0.33)	0.06		0.19	(0.08, 0.30)	0.06	

Table S4d. Sensitivity to unmeasured confounding of childhood predictors in Brazil

		Cantril's Ladder		Life Satisfaction		Happiness	
Variable	Category	E-value for Estimate	E-value for 95% CI	E-value for Estimate	E-value for 95% CI	E-value for Estimate	E-value for 95% CI
Relationship with mother	(Ref: Very bad/somewhat bad)						
	Very good/somewhat good	1.40	1.00	1.38	1.00	1.48	1.15
Relationship with father	(Ref: Very bad/somewhat bad)						
	Very good/somewhat good	1.58	1.41	1.77	1.59	1.69	1.51
Parent marital status	(Ref: Parents married)						
	Divorced	1.31	1.00	1.14	1.00	1.25	1.00
	Parents were never married	1.42	1.19	1.23	1.00	1.12	1.00
	One or both parents had died	1.31	1.00	1.38	1.00	1.36	1.00
Subjective financial status of family growing up	(Ref: Got by)						
	Lived comfortably	1.67	1.53	1.48	1.34	1.48	1.34
	Found it difficult	1.38	1.14	1.17	1.00	1.23	1.00
	Found it very difficult	1.52	1.18	1.09	1.00	1.43	1.00
Abuse	(Ref: No)						
	Yes	1.42	1.23	1.67	1.51	1.60	1.43
Outsider growing up	(Ref: No)						
	Yes	1.31	1.00	1.77	1.58	1.79	1.61
Self-rated health growing up	(Ref: Good)						
	Excellent	1.46	1.28	1.49	1.33	1.63	1.47
	Very good	1.19	1.00	1.01	1.00	1.20	1.00

Variable	Category	Cantril's Ladder		Life Satisfaction		Happiness	
		E-value for Estimate	E-value for 95% CI	E-value for Estimate	E-value for 95% CI	E-value for Estimate	E-value for 95% CI
	Fair	1.64	1.38	1.67	1.41	1.53	1.26
	Poor	1.82	1.19	1.96	1.32	1.36	1.00
Immigration status	(Ref: Born in this country)						
	Born in another country	1.59	1.00	1.10	1.00	1.25	1.00
Age 12 religious service attendance	(Ref: Never)						
	At least 1/week	1.37	1.05	1.41	1.12	1.46	1.20
	1-3/month	1.29	1.00	1.39	1.00	1.37	1.00
	< 1/month	1.25	1.00	1.33	1.00	1.32	1.00
Year of birth	(Ref: 1998-2005; current age: 18-24)						
	1993-1998; age 25-29	1.13	1.00	1.33	1.00	1.11	1.00
	1983-1993; age 30-39	1.49	1.31	1.62	1.44	1.43	1.23
	1973-1983; age 40-49	1.55	1.35	1.64	1.45	1.42	1.19
	1963-1973; age 50-59	1.41	1.14	1.67	1.45	1.43	1.15
	1953-1963; age 60-69	1.96	1.70	1.85	1.59	1.76	1.52
	1943-1953; age 70-79	2.03	1.58	2.74	2.32	2.14	1.77
	1943 or earlier; age 80+	2.07	1.00	2.40	1.59	2.05	1.14
Gender	(Ref: Male)						
	Female	1.31	1.15	1.12	1.00	1.21	1.00
	Other	1.59	1.00	1.25	1.00	1.24	1.00
Religious affiliation	(Ref: No religion/Atheist/Agnostic)						
	Christianity	1.18	1.00	1.24	1.00	1.16	1.00

Variable	Category	Cantril's Ladder		Life Satisfaction		Happiness	
		E-value for Estimate	E-value for 95% CI	E-value for Estimate	E-value for 95% CI	E-value for Estimate	E-value for 95% CI
	Collapsed affiliations with prevalence<3%	1.34	1.00	1.12	1.00	1.25	1.00
Race/ethnicity	(Ref: Plurality group)						
	Non-plurality groups	1.14	1.00	1.37	1.21	1.36	1.21

Table S4e. Complete-case supplemental analysis of means by demographic category for Brazil

		Cantril's Ladder				Life Satisfaction				Happiness			
Variable	Category	Mean	95% CI	SE	Global p-value	Mean	95% CI	SE	Global p-value	Mean	95% CI	SE	Global p-value
Age group	18-24	6.39	(6.28,6.50)	0.05	< .001	6.86	(6.74,6.99)	0.06	< .001	7.14	(7.03,7.26)	0.06	< .001
	25-29	6.43	(6.28,6.58)	0.08		7.12	(6.97,7.26)	0.07		7.30	(7.17,7.44)	0.07	
	30-39	6.66	(6.57,6.75)	0.05		7.34	(7.25,7.43)	0.05		7.46	(7.37,7.55)	0.05	
	40-49	6.76	(6.65,6.86)	0.05		7.42	(7.31,7.52)	0.05		7.47	(7.37,7.57)	0.05	
	50-59	6.75	(6.62,6.87)	0.07		7.55	(7.43,7.67)	0.06		7.59	(7.47,7.72)	0.07	
	60-69	7.18	(7.01,7.35)	0.09		7.76	(7.59,7.93)	0.09		7.78	(7.62,7.93)	0.08	
	70-79	7.33	(7.00,7.66)	0.17		8.35	(8.08,8.62)	0.14		8.05	(7.78,8.32)	0.14	
	80 or older	7.35	(6.66,8.05)	0.35		8.21	(7.72,8.70)	0.25		8.42	(7.94,8.90)	0.24	
Gender	Female	6.71	(6.64,6.77)	0.03	0.846	7.33	(7.26,7.40)	0.03	0.074	7.42	(7.36,7.48)	0.03	0.020
	Male	6.72	(6.65,6.79)	0.04		7.45	(7.37,7.52)	0.04		7.55	(7.48,7.62)	0.04	
	Other	6.55	(5.90,7.20)	0.32		7.31	(6.47,8.15)	0.41		7.56	(6.90,8.22)	0.32	
Marital status	Divorced	6.63	(6.44,6.82)	0.10	< .001	7.26	(7.07,7.45)	0.10	< .001	7.45	(7.28,7.61)	0.08	< .001
	Domestic partner	6.56	(6.45,6.66)	0.05		7.30	(7.19,7.41)	0.06		7.36	(7.26,7.46)	0.05	
	Married	7.13	(7.05,7.21)	0.04		7.82	(7.74,7.90)	0.04		7.87	(7.80,7.94)	0.04	
	Separated	5.98	(5.71,6.25)	0.14		6.83	(6.57,7.08)	0.13		6.97	(6.73,7.22)	0.12	
	Single/Never been married	6.40	(6.32,6.48)	0.04		6.99	(6.90,7.08)	0.05		7.16	(7.07,7.24)	0.04	
	Widowed	7.32	(7.00,7.65)	0.17		8.02	(7.73,8.30)	0.15		7.90	(7.60,8.21)	0.16	
Employment	Employed for an employer	6.86	(6.78,6.94)	0.04	< .001	7.50	(7.42,7.58)	0.04	< .001	7.58	(7.50,7.65)	0.04	< .001
	Homemaker	6.76	(6.59,6.94)	0.09		7.56	(7.40,7.72)	0.08		7.61	(7.45,7.76)	0.08	
	None of these/Other	6.71	(6.45,6.97)	0.13		7.32	(7.05,7.58)	0.13		7.45	(7.18,7.71)	0.13	
	Retired	7.32	(7.16,7.49)	0.09		8.01	(7.85,8.16)	0.08		7.97	(7.82,8.11)	0.07	
	Self-employed	6.74	(6.65,6.84)	0.05		7.41	(7.32,7.51)	0.05		7.56	(7.47,7.65)	0.05	
	Student	6.64	(6.46,6.83)	0.09		7.04	(6.83,7.26)	0.11		7.27	(7.07,7.46)	0.10	
	Unemployed and looking for a job	6.04	(5.92,6.17)	0.06		6.77	(6.64,6.90)	0.07		6.93	(6.81,7.06)	0.06	
Religious service attendance	A few times a year	6.58	(6.50,6.66)	0.04	< .001	7.28	(7.20,7.36)	0.04	< .001	7.36	(7.28,7.44)	0.04	< .001
	More than once a week	7.29	(7.17,7.41)	0.06		8.01	(7.89,8.12)	0.06		8.12	(8.01,8.22)	0.05	
	Never	6.35	(6.25,6.45)	0.05		6.86	(6.75,6.97)	0.06		6.99	(6.89,7.10)	0.05	
	Once a week	6.90	(6.78,7.01)	0.06		7.60	(7.48,7.71)	0.06		7.71	(7.60,7.82)	0.06	

Variable	Category	Cantril's Ladder				Life Satisfaction				Happiness			
		Mean	95% CI	SE	Global p-value	Mean	95% CI	SE	Global p-value	Mean	95% CI	SE	Global p-value
	One to three times a month	6.65	(6.49,6.80)	0.08		7.43	(7.29,7.57)	0.07		7.48	(7.34,7.62)	0.07	
Education	Up to 8 years	6.80	(6.67,6.92)	0.06	< .001	7.72	(7.61,7.84)	0.06	< .001	7.76	(7.65,7.87)	0.06	< .001
	16+ years	7.04	(6.95,7.14)	0.05		7.41	(7.32,7.51)	0.05		7.50	(7.41,7.60)	0.05	
	9 to 15 years	6.58	(6.52,6.64)	0.03		7.24	(7.18,7.31)	0.03		7.37	(7.31,7.43)	0.03	
Immigration status	Born in another country	7.14	(6.79,7.49)	0.18	0.016	7.51	(7.10,7.92)	0.21	0.533	7.48	(7.08,7.87)	0.20	0.975
	Born in this country	6.71	(6.66,6.76)	0.03		7.38	(7.33,7.43)	0.03		7.48	(7.44,7.53)	0.02	
Religious affiliation	Buddhism	6.92	(6.14,7.71)	0.38	< .001	6.79	(5.90,7.68)	0.43	< .001	7.24	(6.42,8.07)	0.39	< .001
	Christianity	6.81	(6.75,6.86)	0.03		7.53	(7.48,7.59)	0.03		7.62	(7.57,7.67)	0.03	
	Confucianism	8.43	*	*		7.99	*	*		8.05	*	*	
	Hinduism	6.23	*	*		3.23	*	*		4.93	*	*	
	Islam	7.80	*	*		8.25	*	*		8.77	*	*	
	Judaism	6.42	(5.55,7.29)	0.40		6.18	(4.63,7.74)	0.72		6.90	(5.59,8.21)	0.61	
	No religion/Atheist/Agnostic	6.27	(6.14,6.40)	0.07		6.75	(6.61,6.89)	0.07		6.92	(6.79,7.05)	0.07	
	Primal, Animist, or Folk religion	7.58	(0.00,662663905793892526444559466496.00)	0.66		7.14	(0.00,870137853411920474970584514560.00)	0.87		8.01	(0.00,559925846069562741118560370688.00)	0.56	
	Some other religion	6.62	(6.14,7.10)	0.24		7.21	(6.61,7.80)	0.30		6.95	(6.42,7.48)	0.27	
	Taoism	7.08	*	*		7.64	*	*		7.53	*	*	
	Baha'i	10.00	*	*		10.00	*	*		9.65	*	*	
	Jainism	5.89	*	*		3.83	*	*		6.89	*	*	
	Shinto	7.33	*	*		6.34	*	*		6.33	*	*	
	Spiritism	6.70	(6.49,6.91)	0.11		7.24	(7.03,7.44)	0.10		7.28	(7.06,7.49)	0.11	
	Umbanda, Candomblé, and other African-derived religions	6.42	(6.19,6.65)	0.12		7.00	(6.77,7.24)	0.12		7.20	(6.98,7.42)	0.11	

Table S4f. Complete-case supplemental analysis of childhood predictors regression analysis results for Brazil

		Cantril's Ladder				Life Satisfaction				Happiness			
Variable	Category	Est	95% CI	SE	Global p-value	Est	95% CI	SE	Global p-value	Est	95% CI	SE	Global p-value
Relationship with mother	(Ref: Very bad/somewhat bad)				0.365				0.720				0.098
	Very good/somewhat good	0.09	(-0.11,0.29)	0.10		0.04	(-0.17,0.25)	0.11		0.17	(-0.03,0.38)	0.10	
Relationship with father	(Ref: Very bad/somewhat bad)				<.001				<.001				<.001
	Very good/somewhat good	0.33	(0.19,0.46)	0.07		0.44	(0.30,0.57)	0.07		0.37	(0.24,0.51)	0.07	
Parent marital status	(Ref: Parents married)				0.007				0.418				0.522
	No, one or both of them had died	0.00	(-0.28,0.28)	0.14		-0.06	(-0.34,0.22)	0.14		-0.02	(-0.27,0.23)	0.13	
	No, they were never married	-0.06	(-0.23,0.12)	0.09		-0.01	(-0.19,0.16)	0.09		0.08	(-0.09,0.25)	0.09	
	Yes, married	0.18	(0.04,0.32)	0.07		0.08	(-0.06,0.23)	0.07		0.09	(-0.05,0.23)	0.07	
Subjective financial status of family growing up	(Ref: Got by)				<.001				<.001				<.001
	Found it difficult	-0.19	(-0.33,-0.05)	0.07		-0.06	(-0.20,0.09)	0.07		-0.04	(-0.17,0.10)	0.07	
	Found it very difficult	-0.07	(-0.28,0.15)	0.11		0.21	(-0.00,0.42)	0.11		0.07	(-0.14,0.28)	0.11	
	Lived comfortably	0.42	(0.31,0.53)	0.06		0.21	(0.10,0.32)	0.06		0.28	(0.18,0.39)	0.05	
Abuse	(Ref: No)				<.001				<.001				<.001
	Yes	-0.24	(-0.36,-0.11)	0.06		-0.45	(-0.57,-0.32)	0.07		-0.43	(-0.56,-0.31)	0.06	
Outsider growing up	(Ref: No)				0.363				<.001				<.001
	Yes	-0.07	(-0.21,0.08)	0.07		-0.44	(-0.59,-0.29)	0.08		-0.49	(-0.64,-0.35)	0.07	

		Cantril's Ladder				Life Satisfaction				Happiness			
Variable	Category	Est	95% CI	SE	Global p-value	Est	95% CI	SE	Global p-value	Est	95% CI	SE	Global p-value
Self-rated health growing up	(Ref: Good)				<.001				<.001				<.001
	Excellent	0.26	(0.13,0.39)	0.07		0.38	(0.25,0.50)	0.06		0.41	(0.29,0.53)	0.06	
	Fair	-0.37	(-0.56,-0.17)	0.10		-0.30	(-0.50,-0.11)	0.10		-0.26	(-0.45,-0.07)	0.10	
	Poor	-0.40	(-0.86,0.07)	0.24		-0.29	(-0.80,0.22)	0.26		0.05	(-0.37,0.47)	0.21	
	Very good	0.03	(-0.10,0.16)	0.07		0.04	(-0.09,0.17)	0.07		0.07	(-0.06,0.20)	0.07	
Immigration status	(Ref: Born in this country)				0.040				0.692				0.746
	Born in another country	0.35	(0.02,0.69)	0.17		0.08	(-0.31,0.47)	0.20		-0.06	(-0.44,0.31)	0.19	
Age 12 religious service attendance	(Ref: Never)				0.096				0.143				0.018
	At least once a week	0.17	(0.00,0.34)	0.09		0.08	(-0.09,0.26)	0.09		0.16	(-0.00,0.33)	0.08	
	Less than once a month	0.05	(-0.13,0.24)	0.09		-0.05	(-0.24,0.13)	0.09		-0.01	(-0.19,0.17)	0.09	
	One to three times a month	0.11	(-0.08,0.29)	0.10		0.02	(-0.17,0.22)	0.10		0.07	(-0.11,0.26)	0.09	
Year of birth	(Ref: 1998-2005; current age: 18-24)				<.001				<.001				<.001
	1993-1998; age 25-29	0.05	(-0.12,0.23)	0.09		0.21	(0.03,0.40)	0.09		0.13	(-0.04,0.29)	0.09	
	1983-1993; age 30-39	0.28	(0.14,0.42)	0.07		0.41	(0.26,0.56)	0.08		0.26	(0.12,0.40)	0.07	
	1973-1983; age 40-49	0.36	(0.21,0.51)	0.08		0.46	(0.30,0.62)	0.08		0.25	(0.10,0.40)	0.08	
	1963-1973; age 50-59	0.31	(0.14,0.48)	0.09		0.54	(0.37,0.72)	0.09		0.34	(0.16,0.51)	0.09	
	1953-1963; age 60-69	0.73	(0.53,0.94)	0.10		0.74	(0.52,0.96)	0.11		0.50	(0.31,0.70)	0.10	
	1943-1953; age 70-79	0.85	(0.49,1.20)	0.18		1.32	(1.02,1.62)	0.15		0.78	(0.49,1.07)	0.15	

		Cantril's Ladder				Life Satisfaction				Happiness			
Variable	Category	Est	95% CI	SE	Global p-value	Est	95% CI	SE	Global p-value	Est	95% CI	SE	Global p-value
	1943 or earlier; age 80+	0.92	(0.21,1.62)	0.36		1.19	(0.64,1.74)	0.28		1.19	(0.65,1.73)	0.27	
Gender	(Ref: Male)				0.006				0.182				0.215
	Male	-0.16	(-0.26,-0.06)	0.05		-0.09	(-0.19,0.01)	0.05		-0.07	(-0.17,0.02)	0.05	
	Other	-0.14	(-0.95,0.67)	0.41		0.15	(-0.58,0.88)	0.37		0.32	(-0.46,1.10)	0.40	
Religious affiliation	(Ref: No religion/Atheist/Agnostic)				0.692				0.514				0.932
	Christianity	-0.02	(-0.24,0.19)	0.11		0.09	(-0.12,0.29)	0.10		-0.03	(-0.22,0.17)	0.10	
	Christianity	-0.02	(-0.24,0.19)	0.11		0.09	(-0.12,0.29)	0.10		-0.03	(-0.22,0.17)	0.10	
	Collapsed affiliations with prevalence<3%	0.06	(-0.22,0.34)	0.14		-0.00	(-0.28,0.28)	0.14		-0.00	(-0.26,0.26)	0.13	
Race/ethnicity	(Ref: Plurality group)				0.111				<.001				<.001
	Non-plurality groups	0.08	(-0.02,0.18)	0.05		0.22	(0.12,0.31)	0.05		0.22	(0.12,0.31)	0.05	

Table S4g. Complete-case supplemental analysis of sensitivity to unmeasured confounding of childhood predictors in Brazil

		Cantril's Ladder		Life Satisfaction		Happiness	
Variable	Category	E-value for Estimate	E-value for 95% CI	E-value for Estimate	E-value for 95% CI	E-value for Estimate	E-value for 95% CI
Relationship with mother	(Ref: Very bad/somewhat bad)						
	Very good/somewhat good	1.24	1.00	1.15	1.00	1.37	1.00
Relationship with father	(Ref: Very bad/somewhat bad)						
	Very good/somewhat good	1.57	1.39	1.70	1.53	1.64	1.47
Parent marital status	(Ref: Parents married)						
	No, one or both of them had died	1.02	1.00	1.19	1.00	1.10	1.00
	No, they were never married	1.18	1.00	1.07	1.00	1.23	1.00
	Yes, married	1.38	1.14	1.23	1.00	1.25	1.00
Subjective financial status of family growing up	(Ref: Got by)						
	Found it difficult	1.39	1.17	1.18	1.00	1.15	1.00
	Found it very difficult	1.20	1.00	1.41	1.00	1.20	1.00
	Lived comfortably	1.69	1.55	1.41	1.25	1.52	1.38
Abuse	(Ref: No)						
	Yes	1.45	1.28	1.71	1.55	1.71	1.55
Outsider growing up	(Ref: No)						
	Yes	1.20	1.00	1.70	1.51	1.79	1.61
Self-rated health growing up	(Ref: Good)						
	Excellent	1.48	1.31	1.62	1.47	1.69	1.53
	Fair	1.62	1.36	1.53	1.27	1.49	1.22
	Poor	1.66	1.00	1.52	1.00	1.18	1.00
	Very good	1.13	1.00	1.15	1.00	1.21	1.00
Immigration status	(Ref: Born in this country)						
	Born in another country	1.60	1.09	1.22	1.00	1.20	1.00
Age 12 religious service attendance	(Ref: Never)						
	At least once a week	1.37	1.04	1.23	1.00	1.36	1.00
	Less than once a month	1.17	1.00	1.18	1.00	1.06	1.00
	One to three times a month	1.27	1.00	1.11	1.00	1.22	1.00
Year of birth	(Ref: 1998-2005; current age: 18-24)						

Variable	Category	Cantril's Ladder		Life Satisfaction		Happiness	
		E-value for Estimate	E-value for 95% CI	E-value for Estimate	E-value for 95% CI	E-value for Estimate	E-value for 95% CI
	1993-1998; age 25-29	1.18	1.00	1.42	1.13	1.30	1.00
	1983-1993; age 30-39	1.51	1.32	1.66	1.47	1.49	1.30
	1973-1983; age 40-49	1.61	1.41	1.72	1.53	1.47	1.26
	1963-1973; age 50-59	1.54	1.32	1.83	1.61	1.59	1.36
	1953-1963; age 60-69	2.08	1.82	2.08	1.80	1.80	1.55
	1943-1953; age 70-79	2.23	1.77	2.89	2.45	2.17	1.79
	1943 or earlier; age 80+	2.33	1.42	2.69	1.95	2.77	2.00
Gender	(Ref: Male)						
	Male	1.35	1.19	1.24	1.00	1.22	1.00
	Other	1.32	1.00	1.33	1.00	1.56	1.00
Religious affiliation	(Ref: No religion/Atheist/Agnostic)						
	Christianity	1.11	1.00	1.23	1.00	1.12	1.00
	Christianity	1.11	1.00	1.23	1.00	1.12	1.00
	Collapsed affiliations with prevalence<3%	1.19	1.00	1.03	1.00	1.00	1.00
Race/ethnicity	(Ref: Plurality group)						
	Non-plurality groups	1.22	1.00	1.42	1.28	1.44	1.30

Tables S5a-g: Egypt

Table S5a. Nationally representative descriptive statistics for Egypt

Characteristic	N = 4,729[1]
**Age group**	
1998-2005; age 18-24	960 (20%)
1993-1998; age 25-29	607 (13%)
1983-1993; age 30-39	1,204 (25%)
1973-1983; age 40-49	897 (19%)
1963-1973; age 50-59	613 (13%)
1953-1963; age 60-69	387 (8.2%)
1943-1953; age 70-79	54 (1.1%)
1943 or earlier; age 80+	7 (0.2%)
(Missing)	0 (0%)
**Gender**	
Male	2,394 (51%)
Female	2,334 (49%)
Other	0 (0%)
(Missing)	0 (<0.1%)
**Race/Ethnicity**	
Arab	4,585 (97%)
Bedouin Arab	4 (<0.1%)
Greek	1 (<0.1%)
Nubian	27 (0.6%)
Turkish	9 (0.2%)
(Missing)	102 (2.2%)
**Respondent Marital status**	
Married	3,387 (72%)
Separated	39 (0.8%)
Divorced	101 (2.1%)
Widowed	238 (5.0%)
Single, never married	947 (20%)
Domestic Partner	0 (0%)
(Missing)	17 (0.4%)
**Employment**	
Employed for an employer	1,267 (27%)
Self-employed	892 (19%)
Retired	253 (5.4%)
Student	297 (6.3%)
Homemaker	1,772 (37%)
Unemployed and looking for a job	224 (4.7%)
None of these/Other	21 (0.4%)
(Missing)	3 (<0.1%)
**Religious service attendance as an adult (now)**	
More than 1/week	839 (18%)
1/week	960 (20%)
1-3/month	368 (7.8%)
A few times a year	458 (9.7%)
Never	2,091 (44%)
(Missing)	12 (0.3%)
**Education (years)**	
Up to 8 years	2,486 (53%)
9-15 years	1,599 (34%)
16+ years	643 (14%)
(Missing)	1 (<0.1%)
**Immigration status**	
Born in this country	4,713 (100%)
Born in another country	16 (0.3%)
(Missing)	1 (<0.1%)
**Religious affiliation as an adult (now)**	
Christianity	120 (2.5%)
Islam	4,607 (97%)
Hinduism	0 (0%)
Buddhism	0 (0%)
Judaism	0 (0%)

Characteristic	N = 4,729[1]
Sikhism	0 (0%)
Baha'i	0 (0%)
Jainism	0 (0%)
Shinto	0 (0%)
Taoism	0 (<0.1%)
Confucianism	0 (0%)
Primal, Animist, or Folk religion	0 (0%)
Spiritism	0 (0%)
Umbanda, Candomble, and other African-derived religions	0 (0%)
Chinese folk/traditional religion	0 (0%)
Some other religion	0 (0%)
No religion/Atheist/Agnostic	0 (0%)
(Missing)	1 (<0.1%)
**Relationship with mother growing up**	
Very good	4,110 (87%)
Somewhat good	505 (11%)
Somewhat bad	21 (0.4%)
Very bad	10 (0.2%)
Does not apply	83 (1.8%)
(Missing)	0 (0%)
**Relationship with father growing up**	
Very good	3,713 (79%)
Somewhat good	683 (14%)
Somewhat bad	56 (1.2%)
Very bad	30 (0.6%)
Does not apply	233 (4.9%)
(Missing)	14 (0.3%)
**Parent marital status at age 12**	
Parents married	4,049 (86%)
Divorced	131 (2.8%)
Parents were never married	9 (0.2%)
One or both parents had died	485 (10%)
(Missing)	55 (1.2%)
**Subjective financial status of family growing up**	
Lived comfortably	1,251 (26%)
Got by	2,352 (50%)
Found it difficult	857 (18%)
Found it very difficult	268 (5.7%)
(Missing)	1 (<0.1%)
**Abuse**	
Yes	405 (8.6%)
No	4,293 (91%)
(Missing)	30 (0.6%)
**Outsider growing up**	
Yes	260 (5.5%)
No	4,456 (94%)
(Missing)	13 (0.3%)
**Self-rated health growing up**	
Excellent	2,687 (57%)
Very good	1,174 (25%)
Good	497 (11%)
Fair	265 (5.6%)
Poor	106 (2.2%)
(Missing)	1 (<0.1%)
**Age 12 religious service attendance**	
At least 1/week	2,307 (49%)
1-3/month	570 (12%)
<1/month	629 (13%)
Never	1,165 (25%)
(Missing)	57 (1.2%)
**Religious affiliation at age 12**	
Christianity	123 (2.6%)
Islam	4,602 (97%)
Hinduism	0 (0%)
Buddhism	0 (0%)

**Characteristic**	**N = 4,729**[1]
Judaism	0 (0%)
Sikhism	0 (0%)
Baha'i	0 (0%)
Jainism	1 (<0.1%)
Shinto	0 (0%)
Taoism	0 (<0.1%)
Confucianism	0 (0%)
Primal, Animist, or Folk religion	0 (0%)
Spiritism	0 (0%)
Umbanda, Candomble, and other African-derived religions	0 (0%)
Chinese folk/traditional religion	0 (0%)
Some other religion	0 (0%)
No religion/Atheist/Agnostic	0 (0%)
(Missing)	3 (<0.1%)

[1]n (%)

Table S5b. Means by demographic category for Egypt

Variable	Category	Cantril's Ladder				Life Satisfaction				Happiness			
		Mean	95% CI	SE	Global p-value	Mean	95% CI	SE	Global p-value	Mean	95% CI	SE	Global p-value
Age group	18-24	5.43	(5.21,5.65)	0.11	< .001	7.87	(7.64,8.10)	0.12	0.038	6.32	(6.07,6.57)	0.13	0.013
	25-29	5.14	(4.83,5.46)	0.16		7.75	(7.50,8.01)	0.13		6.28	(6.03,6.54)	0.13	
	30-39	4.75	(4.58,4.92)	0.09		7.61	(7.44,7.78)	0.09		5.89	(5.68,6.10)	0.11	
	40-49	4.75	(4.53,4.98)	0.12		7.60	(7.38,7.82)	0.11		6.21	(5.99,6.43)	0.11	
	50-59	5.12	(4.87,5.36)	0.13		7.60	(7.33,7.86)	0.14		6.23	(5.93,6.52)	0.15	
	60-69	5.30	(4.87,5.74)	0.22		7.82	(7.46,8.18)	0.18		6.27	(5.86,6.67)	0.21	
	70-79	5.65	(4.38,6.91)	0.63		7.15	(5.79,8.50)	0.67		6.96	(5.81,8.12)	0.57	
	80 or older	5.24	*	*		8.89	*	*		7.12	*	*	
Gender	Male	4.79	(4.63,4.96)	0.08	< .001	7.48	(7.32,7.63)	0.08	< .001	6.04	(5.86,6.21)	0.09	0.006
	Female	5.30	(5.18,5.42)	0.06		7.91	(7.77,8.04)	0.07		6.32	(6.18,6.47)	0.07	
Marital status	Married	5.00	(4.87,5.12)	0.06	0.004	7.67	(7.55,7.80)	0.06	0.450	6.28	(6.13,6.43)	0.08	< .001
	Separated	4.26	(3.18,5.34)	0.53		7.45	(6.07,8.83)	0.68		4.91	(3.70,6.12)	0.59	
	Divorced	4.59	(3.95,5.24)	0.33		7.69	(6.91,8.47)	0.39		4.88	(4.12,5.64)	0.38	
	Widowed	4.65	(4.23,5.06)	0.21		8.00	(7.63,8.37)	0.19		5.71	(5.28,6.14)	0.22	
	Never	5.39	(5.15,5.63)	0.12		7.69	(7.48,7.90)	0.11		6.12	(5.88,6.35)	0.12	
Employment	Employed for an employer	4.96	(4.74,5.18)	0.11	< .001	7.56	(7.34,7.77)	0.11	0.024	6.06	(5.82,6.30)	0.12	0.148
	Self-employed	4.70	(4.46,4.94)	0.12		7.72	(7.48,7.96)	0.12		6.03	(5.77,6.29)	0.13	
	Retired	4.92	(4.35,5.49)	0.29		7.43	(6.95,7.91)	0.24		6.30	(5.79,6.80)	0.26	
	Student	5.60	(5.27,5.94)	0.17		7.90	(7.52,8.28)	0.19		6.40	(6.10,6.70)	0.15	
	Homemaker	5.23	(5.08,5.38)	0.08		7.83	(7.67,7.98)	0.08		6.29	(6.13,6.46)	0.08	
	Unemployed and looking for a job	4.78	(4.33,5.22)	0.23		7.19	(6.72,7.67)	0.24		6.04	(5.58,6.50)	0.23	
	None of these/Other	5.21	(3.50,6.93)	0.78		8.59	(7.19,9.98)	0.61		6.95	(5.63,8.27)	0.60	
Religious service attendance	More than 1/week	5.37	(5.10,5.63)	0.13	0.052	7.62	(7.36,7.88)	0.13	0.230	6.28	(6.01,6.56)	0.14	0.917
	1/week	4.89	(4.62,5.16)	0.14		7.59	(7.37,7.81)	0.11		6.11	(5.83,6.39)	0.14	
	1-3/month	4.83	(4.50,5.16)	0.17		7.42	(6.98,7.86)	0.22		6.14	(5.75,6.53)	0.20	
	A few times a year	5.14	(4.85,5.43)	0.15		7.86	(7.56,8.17)	0.15		6.16	(5.87,6.45)	0.15	
	Never	5.00	(4.85,5.15)	0.08		7.77	(7.63,7.91)	0.07		6.18	(6.01,6.34)	0.08	
Education	Up to 8 years	4.76	(4.60,4.91)	0.08	< .001	7.53	(7.36,7.70)	0.09	0.004	6.00	(5.80,6.20)	0.10	< .001
	9-15 years	5.21	(5.07,5.34)	0.07		7.85	(7.73,7.97)	0.06		6.21	(6.09,6.34)	0.06	
	16+ years	5.75	(5.55,5.95)	0.10		7.91	(7.67,8.16)	0.12		6.78	(6.54,7.02)	0.12	
Immigration status	Born in this country	5.04	(4.94,5.15)	0.06	0.635	7.69	(7.58,7.80)	0.05	0.635	6.18	(6.05,6.31)	0.07	0.275

Variable	Category	Cantril's Ladder				Life Satisfaction				Happiness			
		Mean	95% CI	SE	Global p-value	Mean	95% CI	SE	Global p-value	Mean	95% CI	SE	Global p-value
	Born in another country	4.96	(3.34,6.59)	0.69		7.35	(5.81,8.90)	0.70		5.27	(3.44,7.10)	0.83	
Religious affiliation	Christianity	5.49	(4.86,6.11)	0.32	< .001	7.14	(6.29,7.99)	0.43	< .001	6.29	(5.60,6.99)	0.35	< .001
	Islam	5.03	(4.92,5.14)	0.06		7.71	(7.60,7.81)	0.06		6.18	(6.05,6.31)	0.07	
	Taoism	2.00	*	*		2.00	*	*		0.00	*	*	
Race/Ethnicity	Arab	5.04	(4.94,5.15)	0.05	0.011	7.69	(7.58,7.80)	0.05	< .001	6.18	(6.05,6.31)	0.07	< .001
	Turkish	5.63	(0.81,10.0ǂ)	1.40		7.56	(2.02,10.0ǂ)	1.68		5.39	(1.39,9.39)	1.16	
	Greek	5.00	*	*		8.26	*	*		5.00	*	*	
	Bedouin Arab	4.97	*	*		4.34	*	*		3.71	*	*	
	Nubian	4.97	(4.33,5.60)	0.30		8.15	(7.76,8.55)	0.19		6.93	(6.51,7.36)	0.20	

Table S5c. Childhood predictors regression analysis results for Egypt

		Cantril's Ladder				Life Satisfaction				Happiness			
Variable	Category	Est	95% CI	SE	Global p-value	Est	95% CI	SE	Global p-value	Est	95% CI	SE	Global p-value
Relationship with mother	(Ref: Very bad/somewhat bad)				0.387				0.884				0.207
	Very good/somewhat good	0.49	(-0.62, 1.60)	0.56		-0.07	(-1.22, 1.07)	0.58		0.75	(-0.41, 1.91)	0.59	
Relationship with father	(Ref: Very bad/somewhat bad)				0.430				0.326				0.054
	Very good/somewhat good	-0.25	(-0.91, 0.40)	0.33		-0.34	(-1.04, 0.37)	0.36		-0.56	(-1.12, 0.01)	0.29	
Parent marital status	(Ref: Parents married)				0.097				0.919				0.852
	Divorced	-0.28	(-0.94, 0.39)	0.34		0.07	(-0.59, 0.73)	0.34		-0.24	(-0.95, 0.47)	0.36	
	Parents were never married	1.21	(-0.06, 2.48)	0.64		0.20	(-0.79, 1.20)	0.51		-0.06	(-1.05, 0.93)	0.50	
	One or both parents had died	-0.29	(-0.67, 0.09)	0.19		0.11	(-0.31, 0.54)	0.22		-0.08	(-0.50, 0.35)	0.22	
Subjective financial status of family growing up	(Ref: Got by)				0.009				0.127				0.004
	Lived comfortably	0.15	(-0.08, 0.38)	0.12		0.03	(-0.22, 0.28)	0.13		0.25	(0.01, 0.49)	0.12	
	Found it difficult	-0.38	(-0.64, -0.12)	0.13		-0.32	(-0.64, 0.00)	0.17		-0.32	(-0.62, -0.02)	0.15	
	Found it very difficult	-0.15	(-0.53, 0.24)	0.20		0.06	(-0.34, 0.46)	0.20		-0.25	(-0.68, 0.17)	0.22	
Abuse	(Ref: No)				0.656				0.045				0.019
	Yes	-0.08	(-0.43, 0.27)	0.18		-0.40	(-0.78, -0.01)	0.20		-0.43	(-0.78, -0.08)	0.18	
Outsider growing up	(Ref: No)				0.944				0.767				0.310
	Yes	0.01	(-0.38, 0.40)	0.20		0.06	(-0.32, 0.44)	0.19		0.21	(-0.19, 0.61)	0.20	
Self-rated health growing up	(Ref: Good)				0.590				0.517				0.263
	Excellent	0.10	(-0.21, 0.42)	0.16		-0.07	(-0.34, 0.19)	0.14		0.05	(-0.26, 0.37)	0.16	

		Cantril's Ladder				Life Satisfaction				Happiness			
Variable	Category	Est	95% CI	SE	Global p-value	Est	95% CI	SE	Global p-value	Est	95% CI	SE	Global p-value
	Very good	-0.04	(-0.35, 0.28)	0.16		-0.27	(-0.60, 0.06)	0.17		-0.02	(-0.34, 0.30)	0.16	
	Fair	-0.15	(-0.62, 0.31)	0.24		-0.07	(-0.50, 0.37)	0.22		-0.41	(-0.87, 0.05)	0.23	
	Poor	-0.27	(-0.93, 0.40)	0.34		-0.07	(-0.77, 0.63)	0.36		-0.20	(-0.94, 0.55)	0.38	
Immigration status	(Ref: Born in this country)				0.523				0.592				0.240
	Born in another country	-0.29	(-1.65, 1.08)	0.69		-0.40	(-1.88, 1.07)	0.75		-0.97	(-2.58, 0.64)	0.82	
Age 12 religious service attendance	(Ref: Never)				<.001				0.029				0.032
	At least 1/week	0.47	(0.25, 0.69)	0.11		0.42	(0.15, 0.70)	0.14		0.39	(0.13, 0.65)	0.13	
	1-3/month	0.20	(-0.11, 0.52)	0.16		0.11	(-0.21, 0.44)	0.16		0.16	(-0.19, 0.51)	0.18	
	< 1/month	0.05	(-0.26, 0.36)	0.16		0.21	(-0.11, 0.53)	0.16		0.08	(-0.23, 0.39)	0.16	
Year of birth	(Ref: 1998-2005; current age: 18-24)				<.001				0.114				0.021
	1993-1998; age 25-29	-0.30	(-0.66, 0.06)	0.19		-0.09	(-0.42, 0.24)	0.17		-0.03	(-0.38, 0.32)	0.18	
	1983-1993; age 30-39	-0.65	(-0.91, -0.38)	0.14		-0.24	(-0.51, 0.03)	0.14		-0.40	(-0.69, -0.10)	0.15	
	1973-1983; age 40-49	-0.62	(-0.95, -0.29)	0.17		-0.23	(-0.53, 0.07)	0.15		-0.07	(-0.37, 0.23)	0.15	
	1963-1973; age 50-59	-0.21	(-0.55, 0.13)	0.18		-0.20	(-0.53, 0.13)	0.17		-0.04	(-0.42, 0.34)	0.19	
	1953-1963; age 60-69	0.03	(-0.48, 0.54)	0.26		0.04	(-0.38, 0.46)	0.21		0.03	(-0.43, 0.49)	0.23	
	1943-1953; age 70-79	0.24	(-1.01, 1.50)	0.64		-0.64	(-1.90, 0.63)	0.65		0.61	(-0.50, 1.73)	0.57	
	1943 or earlier; age 80+	-0.15	(-3.06, 2.75)	1.48		1.02	(0.01, 2.03)	0.52		0.87	(-0.31, 2.04)	0.60	
Gender	(Ref: Male)				<.001				<.001				<.001
	Female	0.71	(0.48, 0.94)	0.12		0.55	(0.32, 0.77)	0.11		0.44	(0.21, 0.67)	0.12	
Religious affiliation	(Ref: Islam)				0.234				0.130				0.827
	Collapsed affiliations with prevalence<3%	0.34	(-0.22, 0.90)	0.29		-0.64	(-1.46, 0.18)	0.42		-0.05	(-0.77, 0.67)	0.37	
Race/ethnicity	(Ref: Plurality group)				0.656				0.903				0.858

Variable	Category	Cantril’s Ladder				Life Satisfaction				Happiness			
		Est	95% CI	SE	Global p-value	Est	95% CI	SE	Global p-value	Est	95% CI	SE	Global p-value
	Non-plurality groups	0.16	(-0.55, 0.88)	0.37		0.07	(-1.10, 1.24)	0.60		0.10	(-1.11, 1.31)	0.62	

Table S5d. Sensitivity to unmeasured confounding of childhood predictors in Egypt

		Cantril's Ladder		Life Satisfaction		Happiness	
Variable	Category	E-value for Estimate	E-value for 95% CI	E-value for Estimate	E-value for 95% CI	E-value for Estimate	E-value for 95% CI
Relationship with mother	(Ref: Very bad/somewhat bad)						
	Very good/somewhat good	1.61	1.00	1.18	1.00	1.84	1.00
Relationship with father	(Ref: Very bad/somewhat bad)						
	Very good/somewhat good	1.38	1.00	1.47	1.00	1.66	1.00
Parent marital status	(Ref: Parents married)						
	Divorced	1.41	1.00	1.17	1.00	1.37	1.00
	Parents were never married	2.30	1.00	1.33	1.00	1.16	1.00
	One or both parents had died	1.42	1.00	1.23	1.00	1.18	1.00
Subjective financial status of family growing up	(Ref: Got by)						
	Lived comfortably	1.28	1.00	1.12	1.00	1.37	1.06
	Found it difficult	1.51	1.24	1.45	1.00	1.44	1.09
	Found it very difficult	1.27	1.00	1.16	1.00	1.38	1.00
Abuse	(Ref: No)						
	Yes	1.19	1.00	1.53	1.06	1.54	1.18
Outsider growing up	(Ref: No)						
	Yes	1.07	1.00	1.15	1.00	1.33	1.00
Self-rated health growing up	(Ref: Good)						
	Excellent	1.22	1.00	1.18	1.00	1.15	1.00
	Very good	1.12	1.00	1.41	1.00	1.09	1.00

Variable	Category	Cantril's Ladder		Life Satisfaction		Happiness	
		E-value for Estimate	E-value for 95% CI	E-value for Estimate	E-value for 95% CI	E-value for Estimate	E-value for 95% CI
	Fair	1.28	1.00	1.17	1.00	1.53	1.00
	Poor	1.40	1.00	1.17	1.00	1.32	1.00
Immigration status	(Ref: Born in this country)						
	Born in another country	1.42	1.00	1.54	1.00	2.04	1.00
Age 12 religious service attendance	(Ref: Never)						
	At least 1/week	1.59	1.38	1.55	1.27	1.51	1.25
	1-3/month	1.33	1.00	1.23	1.00	1.28	1.00
	< 1/month	1.15	1.00	1.34	1.00	1.18	1.00
Year of birth	(Ref: 1998-2005; current age: 18-24)						
	1993-1998; age 25-29	1.43	1.00	1.20	1.00	1.10	1.00
	1983-1993; age 30-39	1.76	1.51	1.37	1.00	1.52	1.22
	1973-1983; age 40-49	1.73	1.43	1.36	1.00	1.17	1.00
	1963-1973; age 50-59	1.34	1.00	1.33	1.00	1.13	1.00
	1953-1963; age 60-69	1.11	1.00	1.13	1.00	1.11	1.00
	1943-1953; age 70-79	1.37	1.00	1.75	1.00	1.71	1.00
	1943 or earlier; age 80+	1.28	1.00	2.12	1.07	1.94	1.00
Gender	(Ref: Male)						
	Female	1.81	1.60	1.67	1.46	1.55	1.33
Religious affiliation	(Ref: Islam)						
	Collapsed affiliations with prevalence<3%	1.47	1.00	1.76	1.00	1.14	1.00
Race/ethnicity	(Ref: Plurality group)						
	Non-plurality groups	1.29	1.00	1.17	1.00	1.21	1.00

Table S5e. Complete-case supplemental analysis of means by demographic category for Egypt

		Cantril's Ladder				Life Satisfaction				Happiness			
Variable	Category	Mean	95% CI	SE	Global p-value	Mean	95% CI	SE	Global p-value	Mean	95% CI	SE	Global p-value
Age group	18-24	5.76	(5.55,5.97)	0.11	< .001	8.14	(7.92,8.35)	0.11	0.072	6.67	(6.44,6.91)	0.12	0.039
	25-29	5.64	(5.37,5.91)	0.14		7.91	(7.65,8.17)	0.13		6.57	(6.34,6.80)	0.12	
	30-39	5.32	(5.17,5.48)	0.08		7.94	(7.78,8.10)	0.08		6.46	(6.29,6.63)	0.09	
	40-49	5.30	(5.12,5.48)	0.09		7.81	(7.62,8.01)	0.10		6.57	(6.36,6.77)	0.11	
	50-59	5.73	(5.51,5.95)	0.11		7.80	(7.57,8.03)	0.12		6.66	(6.43,6.89)	0.12	
	60-69	6.09	(5.72,6.46)	0.19		8.01	(7.69,8.33)	0.16		6.79	(6.42,7.16)	0.19	
	70-79	6.66	(5.44,7.88)	0.61		7.79	(6.74,8.83)	0.52		7.64	(6.81,8.47)	0.41	
	80 or older	5.24	(0.00,1898111 30036.61)	1.53		8.89	(0.00,5045130 6891.75)	0.41		7.12	(0.00,7740632 2003.55)	0.63	
Gender	Female	5.81	(5.71,5.92)	0.05	< .001	8.09	(7.97,8.21)	0.06	0.001	6.73	(6.62,6.85)	0.06	0.002
	Male	5.35	(5.19,5.50)	0.08		7.79	(7.65,7.93)	0.07		6.48	(6.33,6.62)	0.07	
Marital status	Divorced	5.49	(5.00,5.99)	0.25	0.299	7.94	(7.15,8.72)	0.40	0.402	5.85	(5.21,6.49)	0.32	0.046
	Married	5.54	(5.43,5.65)	0.06		7.91	(7.80,8.02)	0.06		6.65	(6.53,6.77)	0.06	
	Separated	5.01	(4.02,6.01)	0.49		8.13	(6.99,9.28)	0.56		5.65	(4.59,6.70)	0.52	
	Single/Never been married	5.75	(5.54,5.97)	0.11		7.95	(7.76,8.14)	0.10		6.55	(6.34,6.77)	0.11	
	Widowed	5.51	(5.10,5.92)	0.21		8.25	(7.93,8.57)	0.16		6.55	(6.21,6.89)	0.17	
Employment	Employed for an employer	5.44	(5.25,5.64)	0.10	0.006	7.85	(7.65,8.05)	0.10	0.055	6.51	(6.29,6.73)	0.11	0.332
	Homemaker	5.76	(5.62,5.89)	0.07		8.06	(7.93,8.19)	0.07		6.71	(6.57,6.85)	0.07	
	None of these/Other	5.69	(4.16,7.21)	0.70		8.75	(7.59,9.91)	0.53		6.95	(5.63,8.27)	0.60	
	Retired	5.78	(5.27,6.28)	0.26		7.69	(7.24,8.14)	0.23		6.75	(6.31,7.19)	0.22	
	Self-employed	5.29	(5.07,5.51)	0.11		7.97	(7.75,8.18)	0.11		6.49	(6.28,6.70)	0.11	
	Student	5.82	(5.52,6.12)	0.15		8.10	(7.78,8.43)	0.16		6.66	(6.40,6.93)	0.14	
	Unemployed and looking for a job	5.46	(5.06,5.86)	0.20		7.40	(6.93,7.86)	0.24		6.51	(6.10,6.92)	0.21	
Religious service attendance	A few times a year	5.47	(5.22,5.73)	0.13	0.061	7.94	(7.65,8.23)	0.15	0.448	6.49	(6.24,6.75)	0.13	0.598
	More than once a week	5.90	(5.66,6.13)	0.12		7.94	(7.69,8.18)	0.12		6.73	(6.51,6.96)	0.11	
	Never	5.57	(5.44,5.71)	0.07		8.01	(7.89,8.13)	0.06		6.62	(6.47,6.76)	0.07	
	Once a week	5.44	(5.19,5.69)	0.13		7.86	(7.65,8.06)	0.10		6.54	(6.30,6.77)	0.12	
	One to three times a month	5.39	(5.07,5.70)	0.16		7.76	(7.39,8.13)	0.19		6.55	(6.20,6.91)	0.18	
Education	Up to 8 years	5.45	(5.30,5.59)	0.07	< .001	7.80	(7.65,7.95)	0.08	0.003	6.56	(6.40,6.72)	0.08	0.021

		Cantril's Ladder				Life Satisfaction				Happiness			
Variable	Category	Mean	95% CI	SE	Global p-value	Mean	95% CI	SE	Global p-value	Mean	95% CI	SE	Global p-value
	16+ years	5.99	(5.82,6.16)	0.09		8.04	(7.82,8.27)	0.11		6.89	(6.67,7.11)	0.11	
	9 to 15 years	5.60	(5.48,5.72)	0.06		8.12	(8.01,8.23)	0.06		6.56	(6.44,6.67)	0.06	
Immigration status	Born in another country	4.88	(3.62,6.15)	0.58	0.237	7.35	(5.81,8.90)	0.70	0.409	6.08	(4.57,7.59)	0.69	0.447
	Born in this country	5.58	(5.48,5.68)	0.05		7.94	(7.85,8.04)	0.05		6.61	(6.50,6.71)	0.05	
Religious affiliation	Christianity	6.03	(5.41,6.65)	0.31	< .001	7.41	(6.59,8.22)	0.41	< .001	6.65	(6.00,7.30)	0.33	0.893
	Islam	5.57	(5.47,5.67)	0.05		7.96	(7.86,8.05)	0.05		6.60	(6.50,6.71)	0.05	
	Taoism	2.00	(2.00,2.00)	0.00		2.00	(2.00,2.00)	0.00		0.00	(0.00,0.00)	0.00	

Table S5f. Complete-case supplemental analysis of childhood predictors regression analysis results for Egypt

		Cantril's Ladder				Life Satisfaction				Happiness			
Variable	Category	Est	95% CI	SE	Global p-value	Est	95% CI	SE	Global p-value	Est	95% CI	SE	Global p-value
Relationship with mother	(Ref: Very bad/somewhat bad)				0.439				0.973				0.761
	Very good/somewhat good	0.42	(-0.63,1.47)	0.54		-0.02	(-1.13,1.09)	0.57		0.17	(-0.93,1.27)	0.56	
Relationship with father	(Ref: Very bad/somewhat bad)				0.614				0.167				0.191
	Very good/somewhat good	-0.15	(-0.75,0.44)	0.30		-0.45	(-1.07,0.18)	0.32		-0.36	(-0.90,0.17)	0.27	
Parent marital status	(Ref: Parents married)				0.394				0.908				0.822
	No, one or both of them had died	0.18	(-0.42,0.78)	0.31		0.10	(-0.51,0.72)	0.31		0.00	(-0.55,0.56)	0.28	
	No, they were never married	1.06	(-0.16,2.27)	0.62		-0.01	(-1.11,1.09)	0.56		0.09	(-0.74,0.92)	0.42	
	Yes, married	0.29	(-0.30,0.88)	0.30		-0.03	(-0.60,0.54)	0.29		0.16	(-0.38,0.70)	0.28	
Subjective financial status of family growing up	(Ref: Got by)				0.009				0.130				0.104
	Found it difficult	-0.27	(-0.49,-0.05)	0.11		-0.25	(-0.55,0.04)	0.15		-0.15	(-0.43,0.13)	0.14	
	Found it very difficult	-0.27	(-0.68,0.13)	0.21		-0.04	(-0.43,0.35)	0.20		-0.32	(-0.73,0.10)	0.21	
	Lived comfortably	0.14	(-0.06,0.34)	0.10		0.10	(-0.11,0.32)	0.11		0.17	(-0.05,0.40)	0.11	
Abuse	(Ref: No)				0.881				0.359				0.074
	Yes	-0.03	(-0.35,0.30)	0.17		-0.16	(-0.51,0.18)	0.18		-0.34	(-0.71,0.03)	0.19	
Outsider growing up	(Ref: No)				0.954				0.928				0.298
	Yes	-0.01	(-0.40,0.37)	0.20		0.02	(-0.36,0.40)	0.19		0.20	(-0.17,0.57)	0.19	

		Cantril's Ladder				Life Satisfaction				Happiness			
Variable	Category	Est	95% CI	SE	Global p-value	Est	95% CI	SE	Global p-value	Est	95% CI	SE	Global p-value
Self-rated health growing up	(Ref: Good)				0.687				0.527				0.156
	Excellent	0.12	(-0.14,0.39)	0.14		-0.08	(-0.33,0.18)	0.13		0.06	(-0.21,0.34)	0.14	
	Fair	-0.03	(-0.47,0.40)	0.22		-0.11	(-0.51,0.29)	0.20		-0.43	(-0.84,-0.02)	0.21	
	Poor	-0.12	(-0.71,0.47)	0.30		-0.01	(-0.66,0.63)	0.33		0.04	(-0.58,0.65)	0.31	
	Very good	0.03	(-0.25,0.31)	0.14		-0.25	(-0.57,0.07)	0.16		0.01	(-0.29,0.31)	0.15	
Immigration status	(Ref: Born in this country)				0.192				0.390				0.425
	Born in another country	-0.76	(-1.89,0.37)	0.58		-0.65	(-2.12,0.82)	0.75		-0.57	(-1.95,0.82)	0.70	
Age 12 religious service attendance	(Ref: Never)				0.002				0.039				0.168
	At least once a week	0.40	(0.20,0.61)	0.10		0.39	(0.13,0.65)	0.13		0.25	(0.03,0.47)	0.11	
	Less than once a month	-0.00	(-0.28,0.28)	0.14		0.22	(-0.06,0.50)	0.14		0.09	(-0.16,0.34)	0.13	
	One to three times a month	0.20	(-0.08,0.49)	0.14		0.22	(-0.08,0.53)	0.16		0.07	(-0.22,0.36)	0.15	
Year of birth	(Ref: 1998-2005; current age: 18-24)				<.001				0.129				0.056
	1993-1998; age 25-29	-0.12	(-0.45,0.21)	0.17		-0.22	(-0.55,0.12)	0.17		-0.10	(-0.43,0.23)	0.17	
	1983-1993; age 30-39	-0.41	(-0.65,-0.16)	0.13		-0.18	(-0.43,0.08)	0.13		-0.20	(-0.48,0.07)	0.14	
	1973-1983; age 40-49	-0.41	(-0.71,-0.12)	0.15		-0.28	(-0.56,0.00)	0.14		-0.08	(-0.37,0.21)	0.15	
	1963-1973; age 50-59	0.07	(-0.22,0.36)	0.15		-0.27	(-0.57,0.03)	0.15		0.02	(-0.29,0.33)	0.16	
	1953-1963; age 60-69	0.44	(-0.00,0.88)	0.23		-0.05	(-0.42,0.32)	0.19		0.16	(-0.25,0.57)	0.21	
	1943-1953; age 70-79	0.89	(-0.41,2.19)	0.66		-0.28	(-1.26,0.69)	0.50		0.91	(0.02,1.79)	0.45	

		Cantril's Ladder				Life Satisfaction				Happiness			
Variable	Category	Est	95% CI	SE	Global p-value	Est	95% CI	SE	Global p-value	Est	95% CI	SE	Global p-value
	1943 or earlier; age 80+	-0.35	(-3.19,2.49)	1.45		0.83	(-0.12,1.79)	0.49		0.56	(-0.60,1.73)	0.60	
Gender	(Ref: Male)				<.001				<.001				<.001
	Male	-0.66	(-0.85,-0.46)	0.10		-0.43	(-0.63,-0.23)	0.10		-0.34	(-0.53,-0.15)	0.10	
Religious affiliation	(Ref: Islam)				0.273				0.133				0.961
	Collapsed affiliations with prevalence<3 %	0.34	(-0.27,0.95)	0.31		-0.62	(-1.41,0.18)	0.41		-0.02	(-0.62,0.59)	0.31	
Race/ethnicity	(Ref: Plurality group)				0.630				0.679				0.937
	Non-plurality groups	-0.20	(-1.00,0.60)	0.41		0.19	(-0.69,1.06)	0.45		0.04	(-1.01,1.09)	0.53	

Table S5g. Complete-case supplemental analysis of sensitivity to unmeasured confounding of childhood predictors in Egypt

		Cantril's Ladder		Life Satisfaction		Happiness	
Variable	Category	E-value for Estimate	E-value for 95% CI	E-value for Estimate	E-value for 95% CI	E-value for Estimate	E-value for 95% CI
Relationship with mother	(Ref: Very bad/somewhat bad)						
	Very good/somewhat good	1.61	1.00	1.09	1.00	1.32	1.00
Relationship with father	(Ref: Very bad/somewhat bad)						
	Very good/somewhat good	1.31	1.00	1.63	1.00	1.53	1.00
Parent marital status	(Ref: Parents married)						
	No, one or both of them had died	1.34	1.00	1.24	1.00	1.02	1.00
	No, they were never married	2.31	1.00	1.07	1.00	1.21	1.00
	Yes, married	1.46	1.00	1.12	1.00	1.31	1.00
Subjective financial status of family growing up	(Ref: Got by)						
	Found it difficult	1.45	1.16	1.42	1.00	1.30	1.00
	Found it very difficult	1.45	1.00	1.13	1.00	1.49	1.00
	Lived comfortably	1.29	1.00	1.24	1.00	1.33	1.00
Abuse	(Ref: No)						
	Yes	1.11	1.00	1.31	1.00	1.51	1.00
Outsider growing up	(Ref: No)						
	Yes	1.07	1.00	1.09	1.00	1.36	1.00
Self-rated health growing up	(Ref: Good)						
	Excellent	1.27	1.00	1.20	1.00	1.18	1.00
	Fair	1.12	1.00	1.25	1.00	1.61	1.09
	Poor	1.26	1.00	1.07	1.00	1.13	1.00
	Very good	1.12	1.00	1.42	1.00	1.06	1.00
Immigration status	(Ref: Born in this country)						
	Born in another country	1.98	1.00	1.85	1.00	1.75	1.00
Age 12 religious service attendance	(Ref: Never)						
	At least once a week	1.59	1.37	1.57	1.28	1.41	1.11
	Less than once a month	1.04	1.00	1.38	1.00	1.22	1.00
	One to three times a month	1.37	1.00	1.39	1.00	1.18	1.00
Year of birth	(Ref: 1998-2005; current age: 18-24)						

Variable	Category	Cantril's Ladder		Life Satisfaction		Happiness	
		E-value for Estimate	E-value for 95% CI	E-value for Estimate	E-value for 95% CI	E-value for Estimate	E-value for 95% CI
	1993-1998; age 25-29	1.27	1.00	1.38	1.00	1.23	1.00
	1983-1993; age 30-39	1.60	1.32	1.33	1.00	1.36	1.00
	1973-1983; age 40-49	1.60	1.26	1.45	1.00	1.20	1.00
	1963-1973; age 50-59	1.19	1.00	1.44	1.00	1.10	1.00
	1953-1963; age 60-69	1.63	1.00	1.15	1.00	1.31	1.00
	1943-1953; age 70-79	2.12	1.00	1.46	1.00	2.11	1.10
	1943 or earlier; age 80+	1.54	1.00	2.05	1.00	1.75	1.00
Gender	(Ref: Male)						
	Male	1.87	1.66	1.61	1.39	1.51	1.30
Religious affiliation	(Ref: Islam)						
	Collapsed affiliations with prevalence<3%	1.53	1.00	1.81	1.00	1.08	1.00
Race/ethnicity	(Ref: Plurality group)						
	Non-plurality groups	1.36	1.00	1.34	1.00	1.14	1.00

Tables S6a-g: Germany

Table S6a. Nationally representative descriptive statistics for Germany

Characteristic	N = 9,506[1]
**Age group**	
1998-2005; age 18-24	829 (8.7%)
1993-1998; age 25-29	774 (8.1%)
1983-1993; age 30-39	1,438 (15%)
1973-1983; age 40-49	1,494 (16%)
1963-1973; age 50-59	1,729 (18%)
1953-1963; age 60-69	1,915 (20%)
1943-1953; age 70-79	1,137 (12%)
1943 or earlier; age 80+	190 (2.0%)
(Missing)	0 (0%)
**Gender**	
Male	4,641 (49%)
Female	4,843 (51%)
Other	11 (0.1%)
(Missing)	11 (0.1%)
**Respondent Marital status**	
Married	4,784 (50%)
Separated	219 (2.3%)
Divorced	767 (8.1%)
Widowed	409 (4.3%)
Single, never married	2,627 (28%)
Domestic Partner	619 (6.5%)
(Missing)	81 (0.9%)
**Employment**	
Employed for an employer	4,950 (52%)
Self-employed	712 (7.5%)
Retired	2,480 (26%)
Student	605 (6.4%)
Homemaker	251 (2.6%)
Unemployed and looking for a job	288 (3.0%)
None of these/Other	204 (2.1%)
(Missing)	14 (0.2%)
**Religious service attendance as an adult (now)**	
More than 1/week	285 (3.0%)
1/week	424 (4.5%)
1-3/month	550 (5.8%)
A few times a year	2,362 (25%)
Never	5,876 (62%)
(Missing)	9 (<0.1%)
**Education (years)**	
Up to 8 years	235 (2.5%)
9-15 years	6,094 (64%)
16+ years	3,164 (33%)
(Missing)	13 (0.1%)
**Immigration status**	
Born in this country	8,722 (92%)
Born in another country	744 (7.8%)
(Missing)	40 (0.4%)
**Religious affiliation as an adult (now)**	
Christianity	5,052 (53%)
Islam	351 (3.7%)
Hinduism	12 (0.1%)
Buddhism	51 (0.5%)
Judaism	19 (0.2%)
Sikhism	5 (<0.1%)
Baha'i	3 (<0.1%)
Jainism	0 (0%)
Shinto	2 (<0.1%)
Taoism	0 (<0.1%)
Confucianism	4 (<0.1%)
Primal, Animist, or Folk religion	34 (0.4%)

Characteristic	N = 9,506[1]
Spiritism	0 (0%)
Umbanda, Candomble, and other African-derived religions	0 (0%)
Chinese folk/traditional religion	0 (0%)
Some other religion	60 (0.6%)
No religion/Atheist/Agnostic	3,815 (40%)
(Missing)	99 (1.0%)
**Relationship with mother growing up**	
Very good	5,497 (58%)
Somewhat good	3,031 (32%)
Somewhat bad	496 (5.2%)
Very bad	187 (2.0%)
Does not apply	241 (2.5%)
(Missing)	54 (0.6%)
**Relationship with father growing up**	
Very good	4,652 (49%)
Somewhat good	3,012 (32%)
Somewhat bad	846 (8.9%)
Very bad	385 (4.0%)
Does not apply	538 (5.7%)
(Missing)	73 (0.8%)
**Parent marital status at age 12**	
Parents married	7,620 (80%)
Divorced	927 (9.8%)
Parents were never married	578 (6.1%)
One or both parents had died	245 (2.6%)
(Missing)	136 (1.4%)
**Subjective financial status of family growing up**	
Lived comfortably	3,177 (33%)
Got by	4,508 (47%)
Found it difficult	1,481 (16%)
Found it very difficult	314 (3.3%)
(Missing)	26 (0.3%)
**Abuse**	
Yes	1,086 (11%)
No	8,321 (88%)
(Missing)	99 (1.0%)
**Outsider growing up**	
Yes	1,105 (12%)
No	8,262 (87%)
(Missing)	139 (1.5%)
**Self-rated health growing up**	
Excellent	2,633 (28%)
Very good	3,518 (37%)
Good	2,582 (27%)
Fair	612 (6.4%)
Poor	134 (1.4%)
(Missing)	26 (0.3%)
**Age 12 religious service attendance**	
At least 1/week	1,943 (20%)
1-3/month	1,899 (20%)
<1/month	2,887 (30%)
Never	2,749 (29%)
(Missing)	27 (0.3%)
**Religious affiliation at age 12**	
Christianity	5,751 (61%)
Islam	350 (3.7%)
Hinduism	15 (0.2%)
Buddhism	25 (0.3%)
Judaism	18 (0.2%)
Sikhism	5 (<0.1%)
Baha'i	2 (<0.1%)
Jainism	1 (<0.1%)
Shinto	0 (0%)
Taoism	0 (0%)
Confucianism	4 (<0.1%)

Characteristic	N = 9,506[1]
Primal, Animist, or Folk religion	19 (0.2%)
Spiritism	0 (0%)
Umbanda, Candomble, and other African-derived religions	0 (0%)
Chinese folk/traditional religion	0 (0%)
Some other religion	67 (0.7%)
No religion/Atheist/Agnostic	3,163 (33%)
(Missing)	85 (0.9%)

[1]n (%)

Table S6b. Means by demographic category for Germany

		Cantril's Ladder				Life Satisfaction				Happiness			
Variable	Category	Mean	95% CI	SE	Global p-value	Mean	95% CI	SE	Global p-value	Mean	95% CI	SE	Global p-value
Age group	18-24	6.56	(6.39,6.72)	0.08	0.001	6.54	(6.34,6.74)	0.10	< .001	6.59	(6.40,6.78)	0.10	< .001
	25-29	6.79	(6.66,6.93)	0.07		6.80	(6.63,6.98)	0.09		6.83	(6.66,6.99)	0.08	
	30-39	6.85	(6.75,6.95)	0.05		6.93	(6.81,7.05)	0.06		6.93	(6.82,7.05)	0.06	
	40-49	6.68	(6.57,6.80)	0.06		6.79	(6.65,6.92)	0.07		6.74	(6.62,6.86)	0.06	
	50-59	6.64	(6.52,6.75)	0.06		6.86	(6.74,6.99)	0.06		6.87	(6.76,6.99)	0.06	
	60-69	6.76	(6.66,6.87)	0.05		7.07	(6.95,7.19)	0.06		7.01	(6.90,7.11)	0.05	
	70-79	6.79	(6.64,6.95)	0.08		7.23	(7.07,7.39)	0.08		7.11	(6.95,7.26)	0.08	
	80 or older	7.22	(6.92,7.53)	0.15		7.69	(7.34,8.04)	0.18		7.59	(7.33,7.84)	0.13	
Gender	Male	6.77	(6.70,6.83)	0.03	0.038	6.98	(6.90,7.05)	0.04	0.028	6.93	(6.86,6.99)	0.04	0.022
	Female	6.71	(6.65,6.78)	0.03		6.89	(6.81,6.96)	0.04		6.88	(6.81,6.95)	0.04	
	Other	5.53	(4.19,6.87)	0.54		5.95	(4.79,7.11)	0.47		5.39	(3.95,6.82)	0.58	
Marital status	Married	6.98	(6.91,7.04)	0.03	< .001	7.25	(7.18,7.32)	0.04	< .001	7.20	(7.13,7.26)	0.03	< .001
	Separated	6.51	(6.27,6.75)	0.12		6.54	(6.22,6.86)	0.16		6.56	(6.27,6.85)	0.15	
	Divorced	6.20	(6.01,6.39)	0.10		6.54	(6.34,6.75)	0.10		6.51	(6.31,6.71)	0.10	
	Widowed	6.85	(6.63,7.06)	0.11		6.97	(6.72,7.22)	0.13		6.96	(6.75,7.17)	0.11	
	Never	6.45	(6.36,6.54)	0.04		6.48	(6.37,6.58)	0.05		6.49	(6.39,6.59)	0.05	
	Domestic Partner	6.81	(6.65,6.97)	0.08		6.98	(6.80,7.16)	0.09		6.95	(6.78,7.11)	0.08	
Employment	Employed for an employer	6.84	(6.78,6.90)	0.03	< .001	7.01	(6.94,7.08)	0.04	< .001	6.97	(6.91,7.04)	0.03	< .001
	Self-employed	6.96	(6.81,7.10)	0.07		7.10	(6.92,7.28)	0.09		7.09	(6.92,7.25)	0.08	
	Retired	6.71	(6.61,6.81)	0.05		7.06	(6.95,7.16)	0.06		6.98	(6.88,7.08)	0.05	
	Student	6.73	(6.55,6.91)	0.09		6.69	(6.47,6.92)	0.11		6.68	(6.48,6.89)	0.10	
	Homemaker	6.14	(5.81,6.46)	0.17		6.46	(6.11,6.81)	0.18		6.46	(6.10,6.81)	0.18	
	Unemployed and looking for a job	5.46	(5.14,5.77)	0.16		5.43	(5.09,5.77)	0.17		5.81	(5.48,6.14)	0.17	
	None of these/Other	6.38	(5.99,6.77)	0.20		6.15	(5.73,6.58)	0.22		6.29	(5.88,6.69)	0.21	
Religious service attendance	More than 1/week	7.16	(6.91,7.41)	0.13	< .001	7.47	(7.17,7.76)	0.15	< .001	7.43	(7.14,7.72)	0.15	< .001
	1/week	7.18	(6.98,7.39)	0.11		7.29	(7.05,7.54)	0.13		7.28	(7.06,7.49)	0.11	
	1-3/month	7.01	(6.83,7.19)	0.09		7.27	(7.06,7.47)	0.10		7.15	(6.96,7.34)	0.10	
	A few times a year	7.00	(6.91,7.09)	0.04		7.21	(7.11,7.31)	0.05		7.12	(7.03,7.21)	0.05	
	Never	6.56	(6.50,6.62)	0.03		6.73	(6.66,6.80)	0.04		6.74	(6.68,6.80)	0.03	
Education	Up to 8 years	6.18	(5.83,6.54)	0.18	< .001	6.34	(5.97,6.71)	0.19	< .001	6.34	(6.00,6.69)	0.17	< .001
	9-15 years	6.55	(6.49,6.61)	0.03		6.78	(6.71,6.84)	0.03		6.77	(6.71,6.83)	0.03	

Variable	Category	Cantril's Ladder				Life Satisfaction				Happiness			
		Mean	95% CI	SE	Global p-value	Mean	95% CI	SE	Global p-value	Mean	95% CI	SE	Global p-value
	16+ years	7.14	(7.07,7.21)	0.04		7.27	(7.18,7.35)	0.04		7.20	(7.12,7.28)	0.04	
Immigration status	Born in this country	6.75	(6.70,6.80)	0.02	0.134	6.95	(6.89,7.00)	0.03	0.032	6.90	(6.85,6.96)	0.03	0.712
	Born in another country	6.61	(6.44,6.79)	0.09		6.72	(6.53,6.92)	0.10		6.87	(6.69,7.05)	0.09	
Religious affiliation	Christianity	6.82	(6.75,6.88)	0.03	< .001	7.05	(6.98,7.12)	0.03	< .001	7.00	(6.94,7.06)	0.03	< .001
	Islam	6.55	(6.29,6.81)	0.13		6.63	(6.31,6.95)	0.16		6.63	(6.32,6.94)	0.16	
	Hinduism	6.07	*	*		6.84	*	*		6.67	*	*	
	Buddhism	6.57	(5.99,7.14)	0.28		7.20	(6.68,7.73)	0.26		7.34	(6.84,7.84)	0.25	
	Judaism	6.65	(5.52,7.78)	0.44		7.09	(5.96,8.22)	0.42		7.56	(6.30,8.82)	0.47	
	Sikhism	8.30	*	*		8.21	*	*		8.28	*	*	
	Baha'i	8.71	*	*		8.71	*	*		8.71	*	*	
	Shinto	6.00	*	*		5.00	*	*		4.00	*	*	
	Taoism	8.00	*	*		9.00	*	*		8.61	*	*	
	Confucianism	7.39	*	*		8.46	*	*		8.00	*	*	
	Primal, Animist, or Folk religion	5.95	(5.06,6.83)	0.42		5.54	(4.26,6.82)	0.61		6.27	(5.22,7.33)	0.50	
	Some other religion	5.53	(4.32,6.73)	0.60		5.75	(4.50,7.00)	0.62		5.87	(4.59,7.15)	0.64	
	No religion/Atheist /Agnostic	6.68	(6.61,6.75)	0.04		6.82	(6.74,6.91)	0.04		6.81	(6.73,6.88)	0.04	

Table S6c. Childhood predictors regression analysis results for Germany

		Cantril's Ladder				Life Satisfaction				Happiness			
Variable	Category	Est	95% CI	SE	Global p-value	Est	95% CI	SE	Global p-value	Est	95% CI	SE	Global p-value
Relationship with mother	(Ref: Very bad/somewhat bad)				0.375				0.014				0.029
	Very good/somewhat good	0.09	(-0.11, 0.30)	0.10		0.27	(0.05, 0.49)	0.11		0.23	(0.02, 0.44)	0.11	
Relationship with father	(Ref: Very bad/somewhat bad)				0.368				0.596				0.494
	Very good/somewhat good	0.07	(-0.09, 0.23)	0.08		0.05	(-0.13, 0.23)	0.09		-0.06	(-0.22, 0.11)	0.08	
Parent marital status	(Ref: Parents married)				0.018				0.027				0.015
	Divorced	-0.13	(-0.28, 0.03)	0.08		-0.19	(-0.37, -0.01)	0.09		-0.17	(-0.34, 0.00)	0.09	
	Parents were never married	-0.31	(-0.53, -0.09)	0.11		-0.28	(-0.52, -0.05)	0.12		-0.28	(-0.51, -0.06)	0.12	
	One or both parents had died	-0.12	(-0.41, 0.16)	0.14		-0.11	(-0.43, 0.20)	0.16		-0.21	(-0.50, 0.08)	0.15	
Subjective financial status of family growing up	(Ref: Got by)				<.001				<.001				0.004
	Lived comfortably	0.24	(0.13, 0.35)	0.06		0.22	(0.09, 0.34)	0.06		0.15	(0.03, 0.27)	0.06	
	Found it difficult	-0.16	(-0.30, -0.01)	0.07		-0.15	(-0.32, 0.02)	0.09		-0.10	(-0.25, 0.05)	0.08	
	Found it very difficult	-0.38	(-0.71, -0.05)	0.17		-0.33	(-0.68, 0.01)	0.18		-0.31	(-0.62, 0.01)	0.16	
Abuse	(Ref: No)				<.001				<.001				<.001
	Yes	-0.30	(-0.46, -0.15)	0.08		-0.34	(-0.52, -0.17)	0.09		-0.28	(-0.45, -0.12)	0.08	
Outsider growing up	(Ref: No)				<.001				<.001				<.001
	Yes	-0.45	(-0.60, -0.30)	0.08		-0.48	(-0.64, -0.32)	0.08		-0.47	(-0.63, -0.31)	0.08	
Self-rated health growing up	(Ref: Good)				<.001				<.001				<.001
	Excellent	0.40	(0.26, 0.54)	0.07		0.60	(0.44, 0.76)	0.08		0.68	(0.53, 0.82)	0.07	

		Cantril's Ladder				Life Satisfaction				Happiness			
Variable	Category	Est	95% CI	SE	Global p-value	Est	95% CI	SE	Global p-value	Est	95% CI	SE	Global p-value
	Very good	0.17	(0.05, 0.29)	0.06		0.32	(0.19, 0.45)	0.07		0.35	(0.23, 0.48)	0.06	
	Fair	-0.30	(-0.52, -0.08)	0.11		-0.44	(-0.69, -0.20)	0.13		-0.35	(-0.58, -0.12)	0.12	
	Poor	0.45	(-0.08, 0.97)	0.27		0.43	(-0.07, 0.93)	0.26		0.37	(-0.15, 0.88)	0.26	
Immigration status	(Ref: Born in this country)				0.102				0.147				0.006
	Born in another country	0.16	(-0.03, 0.35)	0.10		0.16	(-0.06, 0.37)	0.11		0.27	(0.08, 0.47)	0.10	
Age 12 religious service attendance	(Ref: Never)				<.001				<.001				<.001
	At least 1/week	0.41	(0.27, 0.55)	0.07		0.47	(0.30, 0.63)	0.08		0.39	(0.24, 0.54)	0.08	
	1-3/month	0.33	(0.20, 0.47)	0.07		0.30	(0.14, 0.46)	0.08		0.24	(0.09, 0.38)	0.07	
	< 1/month	0.27	(0.15, 0.39)	0.06		0.27	(0.12, 0.41)	0.07		0.20	(0.07, 0.32)	0.07	
Year of birth	(Ref: 1998-2005; current age: 18-24)				<.001				<.001				<.001
	1993-1998; age 25-29	0.22	(0.01, 0.43)	0.11		0.24	(-0.02, 0.50)	0.13		0.21	(-0.03, 0.45)	0.12	
	1983-1993; age 30-39	0.25	(0.06, 0.44)	0.10		0.33	(0.10, 0.55)	0.11		0.28	(0.07, 0.49)	0.11	
	1973-1983; age 40-49	0.11	(-0.09, 0.31)	0.10		0.22	(-0.01, 0.46)	0.12		0.12	(-0.09, 0.34)	0.11	
	1963-1973; age 50-59	0.07	(-0.13, 0.27)	0.10		0.31	(0.08, 0.54)	0.12		0.27	(0.06, 0.48)	0.11	
	1953-1963; age 60-69	0.24	(0.04, 0.44)	0.10		0.57	(0.34, 0.80)	0.12		0.45	(0.24, 0.66)	0.11	
	1943-1953; age 70-79	0.24	(0.01, 0.47)	0.12		0.69	(0.44, 0.94)	0.13		0.53	(0.30, 0.77)	0.12	
	1943 or earlier; age 80+	0.71	(0.37, 1.05)	0.17		1.16	(0.76, 1.56)	0.20		1.04	(0.72, 1.36)	0.16	
Gender	(Ref: Male)				0.762				0.834				0.545
	Female	-0.01	(-0.10, 0.08)	0.05		-0.03	(-0.13, 0.08)	0.05		0.01	(-0.09, 0.11)	0.05	
	Other	-0.38	(-1.46, 0.70)	0.55		0.15	(-0.83, 1.12)	0.50		-0.52	(-1.48, 0.45)	0.49	
Religious affiliation	(Ref: No religion/Atheist /Agnostic)				0.038				0.085				0.267
	Islam	-0.36	(-0.64, -0.08)	0.14		-0.38	(-0.73, -0.03)	0.18		-0.32	(-0.64, 0.00)	0.16	
	Christianity	-0.08	(-0.18, 0.03)	0.05		0.02	(-0.11, 0.14)	0.06		-0.02	(-0.13, 0.09)	0.06	

Variable	Category	Cantril's Ladder				Life Satisfaction				Happiness			
		Est	95% CI	SE	Global p-value	Est	95% CI	SE	Global p-value	Est	95% CI	SE	Global p-value
Race/ethnicity	Collapsed affiliations with prevalence<3% (Ref: Plurality group)	-0.45	(-1.03, 0.13)	0.30		-0.34	(-0.93, 0.26)	0.30		-0.12	(-0.73, 0.49)	0.31	

Table S6d. Sensitivity to unmeasured confounding of childhood predictors in Germany

		Cantril's Ladder		Life Satisfaction		Happiness	
Variable	Category	E-value for Estimate	E-value for 95% CI	E-value for Estimate	E-value for 95% CI	E-value for Estimate	E-value for 95% CI
Relationship with mother	(Ref: Very bad/somewhat bad)						
	Very good/somewhat good	1.27	1.00	1.50	1.17	1.48	1.11
Relationship with father	(Ref: Very bad/somewhat bad)						
	Very good/somewhat good	1.23	1.00	1.16	1.00	1.19	1.00
Parent marital status	(Ref: Parents married)						
	Divorced	1.33	1.00	1.40	1.07	1.38	1.00
	Parents were never married	1.61	1.27	1.52	1.17	1.55	1.20
	One or both parents had died	1.32	1.00	1.28	1.00	1.45	1.00
Subjective financial status of family growing up	(Ref: Got by)						
	Lived comfortably	1.51	1.33	1.43	1.25	1.36	1.15
	Found it difficult	1.38	1.08	1.34	1.00	1.27	1.00
	Found it very difficult	1.72	1.19	1.58	1.00	1.58	1.00
Abuse	(Ref: No)						
	Yes	1.60	1.37	1.60	1.37	1.55	1.31
Outsider growing up	(Ref: No)						
	Yes	1.81	1.60	1.77	1.56	1.81	1.59
Self-rated health growing up	(Ref: Good)						
	Excellent	1.74	1.53	1.92	1.72	2.10	1.89
	Very good	1.41	1.20	1.57	1.39	1.65	1.48

Variable	Category	Cantril's Ladder		Life Satisfaction		Happiness	
		E-value for Estimate	E-value for 95% CI	E-value for Estimate	E-value for 95% CI	E-value for Estimate	E-value for 95% CI
	Fair	1.60	1.25	1.72	1.40	1.64	1.31
	Poor	1.81	1.00	1.71	1.00	1.67	1.00
Immigration status	(Ref: Born in this country)						
	Born in another country	1.38	1.00	1.35	1.00	1.54	1.23
Age 12 religious service attendance	(Ref: Never)						
	At least 1/week	1.76	1.55	1.75	1.55	1.70	1.49
	1-3/month	1.64	1.44	1.53	1.32	1.49	1.26
	< 1/month	1.55	1.37	1.49	1.30	1.42	1.22
Year of birth	(Ref: 1998-2005; current age: 18-24)						
	1993-1998; age 25-29	1.48	1.08	1.46	1.00	1.45	1.00
	1983-1993; age 30-39	1.53	1.21	1.58	1.27	1.55	1.23
	1973-1983; age 40-49	1.30	1.00	1.44	1.00	1.31	1.00
	1963-1973; age 50-59	1.23	1.00	1.55	1.23	1.53	1.19
	1953-1963; age 60-69	1.51	1.17	1.89	1.59	1.78	1.49
	1943-1953; age 70-79	1.51	1.08	2.04	1.72	1.90	1.57
	1943 or earlier; age 80+	2.20	1.70	2.71	2.14	2.66	2.17
Gender	(Ref: Male)						
	Female	1.09	1.00	1.12	1.00	1.08	1.00
	Other	1.71	1.00	1.33	1.00	1.88	1.00
Religious affiliation	(Ref: No religion/Atheist/Agnostic)						
	Islam	1.68	1.24	1.64	1.14	1.60	1.00
	Christianity	1.24	1.00	1.10	1.00	1.10	1.00

Variable	Category	Cantril's Ladder		Life Satisfaction		Happiness	
		E-value for Estimate	E-value for 95% CI	E-value for Estimate	E-value for 95% CI	E-value for Estimate	E-value for 95% CI
Race/ethnicity	Collapsed affiliations with prevalence<3% (Ref: Plurality group)	1.81	1.00	1.59	1.00	1.31	1.00

Table S6e. Complete-case supplemental analysis of means by demographic category for Germany

		Cantril's Ladder				Life Satisfaction				Happiness			
Variable	Category	Mean	95% CI	SE	Global p-value	Mean	95% CI	SE	Global p-value	Mean	95% CI	SE	Global p-value
Age group	18-24	6.57	(6.40,6.73)	0.08	< .001	6.63	(6.44,6.83)	0.10	< .001	6.67	(6.50,6.85)	0.09	< .001
	25-29	6.80	(6.66,6.93)	0.07		6.88	(6.71,7.05)	0.09		6.90	(6.74,7.05)	0.08	
	30-39	6.88	(6.79,6.98)	0.05		6.96	(6.85,7.08)	0.06		6.97	(6.86,7.08)	0.06	
	40-49	6.72	(6.61,6.83)	0.06		6.85	(6.72,6.98)	0.07		6.79	(6.68,6.91)	0.06	
	50-59	6.67	(6.56,6.78)	0.06		6.94	(6.82,7.06)	0.06		6.94	(6.82,7.05)	0.06	
	60-69	6.79	(6.69,6.89)	0.05		7.13	(7.01,7.24)	0.06		7.04	(6.94,7.14)	0.05	
	70-79	6.86	(6.71,7.01)	0.08		7.31	(7.16,7.46)	0.08		7.17	(7.03,7.31)	0.07	
	80 or older	7.22	(6.92,7.53)	0.15		7.79	(7.49,8.09)	0.15		7.59	(7.33,7.84)	0.13	
Gender	Female	6.75	(6.69,6.82)	0.03	0.052	6.95	(6.88,7.02)	0.04	0.024	6.94	(6.87,7.00)	0.03	0.047
	Male	6.79	(6.73,6.85)	0.03		7.04	(6.97,7.12)	0.04		6.98	(6.91,7.04)	0.03	
	Other	5.53	(4.19,6.87)	0.54		6.08	(4.97,7.19)	0.45		5.69	(4.34,7.05)	0.55	
Marital status	Divorced	6.33	(6.16,6.51)	0.09	< .001	6.73	(6.55,6.91)	0.09	< .001	6.68	(6.51,6.86)	0.09	< .001
	Domestic partner	6.81	(6.65,6.97)	0.08		7.02	(6.84,7.19)	0.09		6.97	(6.81,7.13)	0.08	
	Married	6.99	(6.93,7.05)	0.03		7.28	(7.21,7.34)	0.04		7.21	(7.15,7.28)	0.03	
	Separated	6.52	(6.28,6.76)	0.12		6.60	(6.29,6.92)	0.16		6.60	(6.31,6.89)	0.15	
	Single/Never been married	6.48	(6.40,6.57)	0.04		6.57	(6.48,6.67)	0.05		6.57	(6.48,6.66)	0.05	
	Widowed	6.93	(6.72,7.13)	0.10		7.07	(6.83,7.30)	0.12		7.02	(6.81,7.23)	0.11	
Employment	Employed for an employer	6.85	(6.79,6.91)	0.03	< .001	7.04	(6.97,7.11)	0.03	< .001	7.00	(6.94,7.07)	0.03	< .001
	Homemaker	6.24	(5.92,6.55)	0.16		6.63	(6.30,6.96)	0.17		6.68	(6.35,7.00)	0.16	
	None of these/Other	6.58	(6.27,6.89)	0.16		6.53	(6.20,6.87)	0.17		6.57	(6.24,6.90)	0.17	
	Retired	6.76	(6.66,6.86)	0.05		7.15	(7.04,7.25)	0.05		7.03	(6.94,7.12)	0.05	
	Self-employed	6.97	(6.82,7.11)	0.07		7.12	(6.93,7.30)	0.09		7.10	(6.93,7.26)	0.08	
	Student	6.73	(6.55,6.91)	0.09		6.71	(6.49,6.93)	0.11		6.70	(6.50,6.91)	0.10	
	Unemployed and looking for a job	5.61	(5.32,5.89)	0.15		5.82	(5.52,6.11)	0.15		6.08	(5.80,6.36)	0.14	
Religious service attendance	A few times a year	7.00	(6.91,7.09)	0.04	< .001	7.22	(7.12,7.31)	0.05	< .001	7.13	(7.04,7.22)	0.05	< .001
	More than once a week	7.17	(6.93,7.42)	0.12		7.48	(7.19,7.77)	0.15		7.43	(7.15,7.72)	0.15	
	Never	6.60	(6.55,6.66)	0.03		6.83	(6.77,6.90)	0.03		6.82	(6.76,6.87)	0.03	
	Once a week	7.18	(6.98,7.39)	0.11		7.32	(7.07,7.57)	0.13		7.30	(7.08,7.51)	0.11	

		Cantril's Ladder				Life Satisfaction				Happiness			
Variable	Category	Mean	95% CI	SE	Global p-value	Mean	95% CI	SE	Global p-value	Mean	95% CI	SE	Global p-value
	One to three times a month	7.02	(6.84,7.20)	0.09		7.29	(7.09,7.49)	0.10		7.16	(6.97,7.36)	0.10	
Education	Up to 8 years	6.31	(6.00,6.63)	0.16	< .001	6.51	(6.19,6.83)	0.16	< .001	6.50	(6.20,6.79)	0.15	< .001
	16+ years	7.15	(7.08,7.22)	0.03		7.30	(7.22,7.38)	0.04		7.21	(7.14,7.29)	0.04	
	9 to 15 years	6.59	(6.53,6.65)	0.03		6.85	(6.79,6.92)	0.03		6.84	(6.78,6.90)	0.03	
Immigration status	Born in another country	6.64	(6.47,6.81)	0.09	0.109	6.78	(6.59,6.98)	0.10	0.023	6.91	(6.73,7.09)	0.09	0.616
	Born in this country	6.78	(6.74,6.83)	0.02		7.01	(6.96,7.07)	0.03		6.96	(6.91,7.01)	0.02	
Religious affiliation	Buddhism	6.57	(5.99,7.14)	0.28	< .001	7.25	(6.73,7.77)	0.26	< .001	7.39	(6.90,7.89)	0.24	< .001
	Christianity	6.84	(6.78,6.90)	0.03		7.10	(7.03,7.17)	0.03		7.05	(6.98,7.11)	0.03	
	Confucianism	7.59	*	*		8.59	*	*		8.00	*	*	
	Hinduism	6.13	(0.00,31.32)	0.78		6.84	(0.00,28.65)	0.67		6.67	(0.00,20.12)	0.41	
	Islam	6.59	(6.35,6.83)	0.12		6.66	(6.36,6.96)	0.15		6.70	(6.41,6.99)	0.15	
	Judaism	6.75	(5.60,7.90)	0.42		7.09	(5.96,8.22)	0.42		7.56	(6.30,8.82)	0.47	
	No religion/Atheist/Agnostic	6.71	(6.64,6.78)	0.04		6.90	(6.82,6.98)	0.04		6.86	(6.78,6.93)	0.04	
	Primal, Animist, or Folk religion	5.94	(5.05,6.83)	0.43		5.54	(4.25,6.82)	0.61		6.27	(5.22,7.33)	0.50	
	Sikhism	8.30	*	*		8.21	*	*		8.28	*	*	
	Some other religion	6.24	(5.37,7.10)	0.43		6.56	(5.70,7.42)	0.43		6.62	(5.72,7.52)	0.45	
	Taoism	8.00	*	*		9.00	*	*		9.00	*	*	
	Baha'i	8.71	*	*		8.71	*	*		8.71	*	*	
	Shinto	6.00	*	*		5.00	*	*		4.00	*	*	

Table S6f. Complete-case supplemental analysis of childhood predictors regression analysis results for Germany

		Cantril's Ladder				Life Satisfaction				Happiness			
Variable	Category	Est	95% CI	SE	Global p-value	Est	95% CI	SE	Global p-value	Est	95% CI	SE	Global p-value
Relationship with mother	(Ref: Very bad/somewhat bad)				0.480				0.022				0.141
	Very good/somewhat good	0.07	(-0.12,0.26)	0.10		0.24	(0.03,0.44)	0.10		0.15	(-0.05,0.35)	0.10	
Relationship with father	(Ref: Very bad/somewhat bad)				0.517				0.766				0.499
	Very good/somewhat good	0.05	(-0.10,0.20)	0.08		0.02	(-0.14,0.19)	0.08		-0.05	(-0.20,0.10)	0.07	
Parent marital status	(Ref: Parents married)				0.123				0.051				0.093
	No, one or both of them had died	0.09	(-0.21,0.38)	0.15		0.00	(-0.33,0.34)	0.17		0.02	(-0.29,0.33)	0.16	
	No, they were never married	-0.02	(-0.25,0.21)	0.12		-0.03	(-0.29,0.22)	0.13		0.01	(-0.22,0.24)	0.12	
	Yes, married	0.15	(-0.01,0.30)	0.08		0.18	(0.01,0.35)	0.09		0.16	(0.00,0.32)	0.08	
Subjective financial status of family growing up	(Ref: Got by)				<.001				<.001				0.005
	Found it difficult	-0.16	(-0.30,-0.02)	0.07		-0.14	(-0.29,0.02)	0.08		-0.08	(-0.23,0.06)	0.07	
	Found it very difficult	-0.27	(-0.58,0.04)	0.16		-0.16	(-0.48,0.16)	0.16		-0.18	(-0.48,0.11)	0.15	
	Lived comfortably	0.26	(0.16,0.37)	0.05		0.23	(0.11,0.35)	0.06		0.16	(0.05,0.27)	0.06	
Abuse	(Ref: No)				<.001				<.001				0.001
	Yes	-0.28	(-0.43,-0.13)	0.08		-0.29	(-0.46,-0.13)	0.09		-0.26	(-0.42,-0.11)	0.08	
Outsider growing up	(Ref: No)				<.001				<.001				<.001
	Yes	-0.44	(-0.58,-0.30)	0.07		-0.46	(-0.61,-0.31)	0.08		-0.45	(-0.61,-0.30)	0.08	

Variable	Category	Cantril's Ladder				Life Satisfaction				Happiness			
		Est	95% CI	SE	Global p-value	Est	95% CI	SE	Global p-value	Est	95% CI	SE	Global p-value
Self-rated health growing up	(Ref: Good)				<.001				<.001				<.001
	Excellent	0.42	(0.29,0.56)	0.07		0.61	(0.47,0.76)	0.08		0.70	(0.57,0.84)	0.07	
	Fair	-0.21	(-0.42,-0.01)	0.10		-0.35	(-0.58,-0.12)	0.12		-0.29	(-0.50,-0.08)	0.11	
	Poor	0.66	(0.17,1.15)	0.25		0.54	(0.07,1.01)	0.24		0.44	(-0.05,0.94)	0.25	
	Very good	0.17	(0.06,0.29)	0.06		0.29	(0.16,0.42)	0.07		0.35	(0.23,0.47)	0.06	
Immigration status	(Ref: Born in this country)				0.368				0.479				0.043
	Born in another country	0.08	(-0.10,0.27)	0.09		0.07	(-0.13,0.28)	0.10		0.20	(0.01,0.39)	0.10	
Age 12 religious service attendance	(Ref: Never)				<.001				<.001				<.001
	At least once a week	0.35	(0.21,0.49)	0.07		0.37	(0.22,0.52)	0.08		0.32	(0.18,0.46)	0.07	
	Less than once a month	0.21	(0.09,0.33)	0.06		0.17	(0.04,0.30)	0.07		0.11	(-0.01,0.23)	0.06	
	One to three times a month	0.27	(0.14,0.40)	0.07		0.20	(0.05,0.36)	0.08		0.18	(0.04,0.32)	0.07	
Year of birth	(Ref: 1998-2005; current age: 18-24)				<.001				<.001				<.001
	1993-1998; age 25-29	0.22	(0.01,0.42)	0.11		0.23	(-0.01,0.47)	0.12		0.21	(-0.01,0.44)	0.11	
	1983-1993; age 30-39	0.28	(0.10,0.47)	0.09		0.29	(0.08,0.50)	0.11		0.25	(0.05,0.45)	0.10	
	1973-1983; age 40-49	0.15	(-0.05,0.34)	0.10		0.21	(-0.01,0.43)	0.11		0.11	(-0.09,0.31)	0.10	
	1963-1973; age 50-59	0.11	(-0.08,0.31)	0.10		0.30	(0.08,0.52)	0.11		0.26	(0.06,0.46)	0.10	
	1953-1963; age 60-69	0.29	(0.09,0.48)	0.10		0.56	(0.34,0.77)	0.11		0.43	(0.23,0.63)	0.10	
	1943-1953; age 70-79	0.32	(0.09,0.54)	0.11		0.70	(0.46,0.94)	0.12		0.54	(0.31,0.76)	0.11	

Variable	Category	Cantril's Ladder				Life Satisfaction				Happiness			
		Est	95% CI	SE	Global p-value	Est	95% CI	SE	Global p-value	Est	95% CI	SE	Global p-value
	1943 or earlier; age 80+	0.72	(0.39,1.06)	0.17		1.19	(0.84,1.54)	0.18		0.98	(0.66,1.29)	0.16	
Gender	(Ref: Male)				0.600				0.751				0.601
	Male	-0.00	(-0.09,0.08)	0.05		0.04	(-0.06,0.14)	0.05		-0.01	(-0.10,0.08)	0.05	
	Other	-0.53	(-1.56,0.50)	0.53		0.06	(-0.78,0.90)	0.43		-0.46	(-1.36,0.44)	0.46	
Religious affiliation	(Ref: No religion/Atheist/Agnostic)				0.244				0.199				0.286
	Christianity	-0.04	(-0.15,0.06)	0.05		0.03	(-0.08,0.15)	0.06		-0.00	(-0.11,0.10)	0.05	
	Christianity	-0.04	(-0.15,0.06)	0.05		0.03	(-0.08,0.15)	0.06		-0.00	(-0.11,0.10)	0.05	
	Islam	-0.26	(-0.51,-0.01)	0.13		-0.31	(-0.64,0.01)	0.17		-0.24	(-0.54,0.06)	0.15	
	Collapsed affiliations with prevalence<3%	-0.09	(-0.52,0.34)	0.22		0.02	(-0.42,0.45)	0.22		0.23	(-0.21,0.66)	0.22	
Race/ethnicity	(Ref: Plurality group)												

Table S6g. Complete-case supplemental analysis of sensitivity to unmeasured confounding of childhood predictors in Germany

		Cantril's Ladder		Life Satisfaction		Happiness	
Variable	Category	E-value for Estimate	E-value for 95% CI	E-value for Estimate	E-value for 95% CI	E-value for Estimate	E-value for 95% CI
Relationship with mother	(Ref: Very bad/somewhat bad)						
	Very good/somewhat good	1.23	1.00	1.48	1.14	1.37	1.00
Relationship with father	(Ref: Very bad/somewhat bad)						
	Very good/somewhat good	1.19	1.00	1.12	1.00	1.19	1.00
Parent marital status	(Ref: Parents married)						
	No, one or both of them had died	1.26	1.00	1.05	1.00	1.11	1.00
	No, they were never married	1.11	1.00	1.14	1.00	1.07	1.00
	Yes, married	1.37	1.00	1.39	1.06	1.39	1.04
Subjective financial status of family growing up	(Ref: Got by)						
	Found it difficult	1.39	1.11	1.33	1.00	1.25	1.00
	Found it very difficult	1.56	1.00	1.36	1.00	1.42	1.00
	Lived comfortably	1.55	1.39	1.46	1.28	1.38	1.19
Abuse	(Ref: No)						
	Yes	1.58	1.34	1.55	1.31	1.54	1.29
Outsider growing up	(Ref: No)						
	Yes	1.83	1.62	1.78	1.57	1.82	1.60
Self-rated health growing up	(Ref: Good)						
	Excellent	1.80	1.59	1.98	1.78	2.19	1.98
	Fair	1.48	1.07	1.63	1.30	1.58	1.24
	Poor	2.16	1.41	1.88	1.22	1.80	1.00
	Very good	1.41	1.20	1.55	1.36	1.67	1.49
Immigration status	(Ref: Born in this country)						
	Born in another country	1.26	1.00	1.22	1.00	1.44	1.06
Age 12 religious service attendance	(Ref: Never)						
	At least once a week	1.68	1.47	1.66	1.45	1.62	1.42
	Less than once a month	1.47	1.27	1.38	1.15	1.30	1.00
	One to three times a month	1.57	1.37	1.43	1.18	1.41	1.16
Year of birth	(Ref: 1998-2005; current age: 18-24)						

Variable	Category	Cantril's Ladder		Life Satisfaction		Happiness	
		E-value for Estimate	E-value for 95% CI	E-value for Estimate	E-value for 95% CI	E-value for Estimate	E-value for 95% CI
	1993-1998; age 25-29	1.48	1.08	1.46	1.00	1.46	1.00
	1983-1993; age 30-39	1.58	1.28	1.55	1.24	1.52	1.19
	1973-1983; age 40-49	1.37	1.00	1.43	1.00	1.30	1.00
	1963-1973; age 50-59	1.31	1.00	1.56	1.24	1.54	1.21
	1953-1963; age 60-69	1.59	1.27	1.90	1.61	1.78	1.49
	1943-1953; age 70-79	1.64	1.28	2.10	1.78	1.94	1.62
	1943 or earlier; age 80+	2.26	1.74	2.85	2.31	2.64	2.13
Gender	(Ref: Male)						
	Male	1.05	1.00	1.15	1.00	1.07	1.00
	Other	1.96	1.00	1.20	1.00	1.83	1.00
Religious affiliation	(Ref: No religion/Atheist/Agnostic)						
	Christianity	1.17	1.00	1.14	1.00	1.05	1.00
	Christianity	1.17	1.00	1.14	1.00	1.05	1.00
	Islam	1.55	1.06	1.58	1.00	1.51	1.00
	Collapsed affiliations with prevalence<3%	1.27	1.00	1.10	1.00	1.48	1.00
Race/ethnicity	(Ref: Plurality group)						

Tables S7a-g: Hong Kong

Table S7a. Nationally representative descriptive statistics for Hong Kong

**Characteristic**	**N = 3,012**[1]
**Age group**	
1998-2005; age 18-24	217 (7.2%)
1993-1998; age 25-29	198 (6.6%)
1983-1993; age 30-39	507 (17%)
1973-1983; age 40-49	580 (19%)
1963-1973; age 50-59	711 (24%)
1953-1963; age 60-69	620 (21%)
1943-1953; age 70-79	164 (5.5%)
1943 or earlier; age 80+	15 (0.5%)
(Missing)	0 (0%)
**Gender**	
Male	1,390 (46%)
Female	1,620 (54%)
Other	2 (<0.1%)
(Missing)	0 (0%)
**Race/Ethnicity**	
Chinese (Cantonese)	1,930 (64%)
Chinese (Chaoshan)	201 (6.7%)
Chinese (Fujianese)	117 (3.9%)
Chinese (Hakka)	121 (4.0%)
Chinese (Other ethnicity)	264 (8.8%)
Chinese (Shanghainese)	89 (2.9%)
East Asian (Korean, Japanese)	10 (0.3%)
Other	4 (0.1%)
South Asian (Indian, Nepalese, Pakistani)	17 (0.6%)
Southeast Asian (Filipino, Indonesian, Thailand)	46 (1.5%)
Taiwanese	14 (0.4%)
White	15 (0.5%)
(Missing)	184 (6.1%)
**Respondent Marital status**	
Married	2,080 (69%)
Separated	21 (0.7%)
Divorced	105 (3.5%)
Widowed	45 (1.5%)
Single, never married	723 (24%)
Domestic Partner	37 (1.2%)
(Missing)	1 (<0.1%)
**Employment**	
Employed for an employer	2,056 (68%)
Self-employed	245 (8.1%)
Retired	423 (14%)
Student	55 (1.8%)
Homemaker	114 (3.8%)
Unemployed and looking for a job	62 (2.0%)
None of these/Other	39 (1.3%)
(Missing)	18 (0.6%)
**Religious service attendance as an adult (now)**	
More than 1/week	237 (7.9%)
1/week	567 (19%)
1-3/month	332 (11%)
A few times a year	543 (18%)
Never	1,332 (44%)
(Missing)	1 (<0.1%)
**Education (years)**	
Up to 8 years	433 (14%)
9-15 years	2,031 (67%)
16+ years	547 (18%)
(Missing)	0 (0%)
**Immigration status**	
Born in this country	2,637 (88%)
Born in another country	321 (11%)

Characteristic	N = 3,012[1]
(Missing)	53 (1.8%)
**Religious affiliation as an adult (now)**	
Christianity	757 (25%)
Islam	86 (2.8%)
Hinduism	20 (0.7%)
Buddhism	349 (12%)
Judaism	10 (0.3%)
Sikhism	2 (<0.1%)
Baha'i	3 (<0.1%)
Jainism	1 (<0.1%)
Shinto	19 (0.6%)
Taoism	97 (3.2%)
Confucianism	11 (0.4%)
Primal, Animist, or Folk religion	27 (0.9%)
Spiritism	0 (0%)
Umbanda, Candomble, and other African-derived religions	0 (0%)
Chinese folk/traditional religion	106 (3.5%)
Some other religion	4 (0.1%)
No religion/Atheist/Agnostic	1,518 (50%)
(Missing)	5 (0.2%)
**Relationship with mother growing up**	
Very good	1,077 (36%)
Somewhat good	1,164 (39%)
Somewhat bad	293 (9.7%)
Very bad	49 (1.6%)
Does not apply	426 (14%)
(Missing)	3 (<0.1%)
**Relationship with father growing up**	
Very good	868 (29%)
Somewhat good	1,089 (36%)
Somewhat bad	393 (13%)
Very bad	102 (3.4%)
Does not apply	557 (19%)
(Missing)	3 (0.1%)
**Parent marital status at age 12**	
Parents married	2,752 (91%)
Divorced	114 (3.8%)
Parents were never married	40 (1.3%)
One or both parents had died	50 (1.7%)
(Missing)	56 (1.8%)
**Subjective financial status of family growing up**	
Lived comfortably	906 (30%)
Got by	1,527 (51%)
Found it difficult	473 (16%)
Found it very difficult	84 (2.8%)
(Missing)	22 (0.7%)
**Abuse**	
Yes	318 (11%)
No	2,688 (89%)
(Missing)	5 (0.2%)
**Outsider growing up**	
Yes	664 (22%)
No	2,224 (74%)
(Missing)	124 (4.1%)
**Self-rated health growing up**	
Excellent	545 (18%)
Very good	1,073 (36%)
Good	863 (29%)
Fair	426 (14%)
Poor	91 (3.0%)
(Missing)	13 (0.4%)
**Age 12 religious service attendance**	
At least 1/week	432 (14%)
1-3/month	528 (18%)
<1/month	753 (25%)

Characteristic	N = 3,012[1]
Never	1,295 (43%)
(Missing)	4 (0.1%)
**Religious affiliation at age 12**	
Christianity	715 (24%)
Islam	86 (2.9%)
Hinduism	27 (0.9%)
Buddhism	323 (11%)
Judaism	16 (0.5%)
Sikhism	4 (0.1%)
Baha'i	0 (0%)
Jainism	1 (<0.1%)
Shinto	18 (0.6%)
Taoism	81 (2.7%)
Confucianism	10 (0.3%)
Primal, Animist, or Folk religion	15 (0.5%)
Spiritism	0 (0%)
Umbanda, Candomble, and other African-derived religions	0 (0%)
Chinese folk/traditional religion	108 (3.6%)
Some other religion	5 (0.2%)
No religion/Atheist/Agnostic	1,601 (53%)
(Missing)	1 (<0.1%)

[1]n (%)

Table S7b. Means by demographic category for Hong Kong

		Cantril's Ladder				Life Satisfaction				Happiness			
Variable	Category	Mean	95% CI	SE	Global p-value	Mean	95% CI	SE	Global p-value	Mean	95% CI	SE	Global p-value
Age group	18-24	6.94	(6.70,7.18)	0.12	< .001	7.03	(6.77,7.30)	0.13	< .001	7.22	(6.99,7.46)	0.12	< .001
	25-29	6.60	(6.15,7.05)	0.23		6.43	(6.04,6.82)	0.20		6.81	(6.33,7.29)	0.24	
	30-39	6.23	(6.01,6.44)	0.11		6.31	(6.09,6.53)	0.11		6.55	(6.33,6.78)	0.11	
	40-49	6.76	(6.56,6.97)	0.10		6.87	(6.66,7.08)	0.11		7.03	(6.82,7.23)	0.10	
	50-59	7.26	(7.11,7.41)	0.08		7.38	(7.21,7.56)	0.09		7.49	(7.34,7.64)	0.08	
	60-69	7.07	(6.81,7.33)	0.13		7.52	(7.25,7.79)	0.14		7.46	(7.23,7.69)	0.12	
	70-79	6.76	(6.20,7.31)	0.28		7.32	(6.86,7.78)	0.23		7.50	(7.07,7.94)	0.22	
	80 or older	5.00	*	*		4.65	(3.92,5.38)	0.28		5.65	(4.92,6.38)	0.28	
Gender	Male	6.90	(6.76,7.04)	0.07	< .001	7.06	(6.91,7.20)	0.07	0.245	7.24	(7.11,7.36)	0.07	< .001
	Female	6.80	(6.67,6.93)	0.07		7.01	(6.87,7.14)	0.07		7.10	(6.97,7.24)	0.07	
	Other	5.45	*	*		5.91	*	*		5.45	*	*	
Marital status	Married	7.21	(7.10,7.33)	0.06	< .001	7.39	(7.27,7.51)	0.06	< .001	7.51	(7.40,7.62)	0.06	< .001
	Separated	6.57	(4.09,9.05)	1.14		7.06	(6.21,7.92)	0.39		7.72	(6.30,9.13)	0.65	
	Divorced	6.88	(6.18,7.58)	0.35		6.74	(6.08,7.39)	0.33		7.05	(6.47,7.64)	0.29	
	Widowed	6.66	(5.69,7.63)	0.48		7.51	(6.76,8.26)	0.35		6.77	(6.12,7.43)	0.32	
	Never	5.83	(5.65,6.01)	0.09		6.04	(5.85,6.24)	0.10		6.22	(6.03,6.41)	0.10	
	Domestic Partner	6.27	(5.31,7.23)	0.47		6.32	(5.77,6.86)	0.27		6.37	(5.78,6.96)	0.29	
Employment	Employed for an employer	6.92	(6.82,7.01)	0.05	< .001	7.07	(6.96,7.17)	0.05	< .001	7.23	(7.13,7.32)	0.05	< .001
	Self-employed	7.50	(7.06,7.94)	0.22		7.36	(6.90,7.83)	0.24		7.70	(7.37,8.03)	0.17	
	Retired	6.60	(6.26,6.94)	0.17		7.20	(6.88,7.52)	0.16		7.12	(6.84,7.41)	0.15	
	Student	6.23	(5.72,6.74)	0.25		6.65	(6.11,7.20)	0.27		6.64	(6.10,7.17)	0.26	
	Homemaker	6.51	(5.99,7.03)	0.26		6.70	(6.11,7.29)	0.30		6.94	(6.40,7.48)	0.27	
	Unemployed and looking for a job	5.63	(4.74,6.53)	0.45		5.54	(4.77,6.31)	0.39		5.68	(4.84,6.53)	0.42	
	None of these/Other	5.39	(3.71,7.07)	0.82		4.89	(3.47,6.32)	0.70		4.66	(2.79,6.52)	0.91	
Religious service attendance	More than 1/week	9.03	(8.82,9.25)	0.11	< .001	8.85	(8.63,9.07)	0.11	< .001	9.23	(9.07,9.40)	0.09	< .001
	1/week	7.35	(7.12,7.57)	0.12		7.49	(7.25,7.74)	0.12		7.56	(7.31,7.81)	0.13	
	1-3/month	7.28	(7.02,7.54)	0.13		7.18	(6.89,7.47)	0.15		7.61	(7.40,7.82)	0.10	
	A few times a year	6.59	(6.33,6.85)	0.13		6.68	(6.43,6.94)	0.13		6.83	(6.61,7.05)	0.11	
	Never	6.24	(6.11,6.37)	0.07		6.61	(6.47,6.75)	0.07		6.65	(6.52,6.79)	0.07	
Education	Up to 8 years	7.19	(6.84,7.54)	0.18	< .001	7.35	(6.99,7.71)	0.18	< .001	7.49	(7.15,7.83)	0.17	< .001
	9-15 years	6.86	(6.75,6.98)	0.06		7.05	(6.93,7.17)	0.06		7.20	(7.10,7.31)	0.05	

Variable	Category	Cantril's Ladder				Life Satisfaction				Happiness			
		Mean	95% CI	SE	Global p-value	Mean	95% CI	SE	Global p-value	Mean	95% CI	SE	Global p-value
	16+ years	6.51	(6.34,6.67)	0.08		6.69	(6.51,6.87)	0.09		6.77	(6.59,6.95)	0.09	
Immigration status	Born in this country	6.92	(6.83,7.01)	0.05	0.002	7.07	(6.97,7.17)	0.05	0.045	7.22	(7.13,7.31)	0.05	0.012
	Born in another country	6.24	(5.81,6.66)	0.22		6.66	(6.26,7.06)	0.20		6.68	(6.26,7.09)	0.21	
Religious affiliation	Christianity	7.04	(6.84,7.24)	0.10	< .001	7.09	(6.88,7.30)	0.11	< .001	7.30	(7.12,7.47)	0.09	< .001
	Islam	7.69	(6.82,8.55)	0.44		7.47	(6.48,8.46)	0.50		7.86	(6.73,8.99)	0.57	
	Hinduism	8.28	(7.06,9.51)	0.39		7.29	(4.88,9.70)	0.76		8.23	(7.21,9.26)	0.32	
	Buddhism	7.57	(7.25,7.89)	0.16		7.66	(7.36,7.95)	0.15		7.85	(7.57,8.13)	0.14	
	Judaism	8.17	*	*		7.59	*	*		8.74	*	*	
	Sikhism	9.08	*	*		8.82	*	*		9.26	*	*	
	Baha'i	8.87	*	*		8.40	*	*		8.91	*	*	
	Jainism	10.00	*	*		8.00	*	*		10.00	*	*	
	Shinto	7.71	(6.68,8.73)	0.24		8.44	(7.39,9.49)	0.24		8.44	(7.24,9.63)	0.28	
	Taoism	7.02	(6.46,7.58)	0.28		6.62	(5.98,7.25)	0.32		6.85	(6.26,7.44)	0.30	
	Confucianism	7.66	*	*		7.74	*	*		7.34	*	*	
	Primal, Animist, or Folk religion	5.96	(5.03,6.89)	0.41		6.44	(5.42,7.45)	0.45		6.34	(5.31,7.37)	0.46	
	Chinese folk/traditional religion	7.29	(6.93,7.65)	0.18		7.48	(7.07,7.88)	0.20		7.69	(7.37,8.02)	0.16	
	Some other religion	9.23	*	*		3.51	*	*		5.18	*	*	
	No religion/Atheist /Agnostic	6.45	(6.33,6.57)	0.06		6.81	(6.68,6.94)	0.06		6.85	(6.73,6.97)	0.06	
Race/Ethnicity	White	7.39	(4.41,10.0ǂ)	0.51	< .001	6.62	(4.55,8.70)	0.50	0.484	6.28	(2.58,9.98)	0.70	0.016
	Other	6.42	*	*		6.42	*	*		6.92	*	*	
	Chinese (Cantonese)	6.81	(6.70,6.92)	0.06		7.00	(6.89,7.12)	0.06		7.13	(7.02,7.24)	0.06	
	Chinese (Chaoshan)	7.13	(6.80,7.46)	0.17		7.28	(6.89,7.66)	0.19		7.49	(7.16,7.82)	0.17	
	Chinese (Fujianese)	7.05	(6.60,7.51)	0.23		6.97	(6.54,7.40)	0.22		7.18	(6.75,7.61)	0.22	
	Chinese (Hakka)	6.56	(6.10,7.02)	0.23		6.64	(6.18,7.10)	0.23		6.72	(6.20,7.23)	0.25	
	Chinese (Shanghainese)	7.08	(6.32,7.85)	0.39		7.06	(6.41,7.71)	0.32		7.22	(6.48,7.96)	0.37	

		Cantril's Ladder				Life Satisfaction				Happiness			
Variable	Category	Mean	95% CI	SE	Global p-value	Mean	95% CI	SE	Global p-value	Mean	95% CI	SE	Global p-value
	Chinese (Other ethnicity)	6.81	(6.48,7.13)	0.16		7.17	(6.74,7.60)	0.21		7.20	(6.91,7.49)	0.15	
	East Asian (Korean, Japanese)	8.39	(4.76,10.0ǂ)	0.63		7.10	(3.39,10.0ǂ)	0.68		8.87	(4.81,10.0ǂ)	0.76	
	Southeast Asian (Filipino, Indonesian, Thailand)	6.83	(5.00,8.66)	0.90		7.34	(5.58,9.10)	0.86		7.37	(5.29,9.45)	1.00	
	South Asian (Indian, Nepalese, Pakistani)	6.05	(1.92,10.0ǂ)	1.30		6.90	(3.50,10.0ǂ)	0.89		7.73	(6.20,9.26)	0.51	
	Taiwanese	7.84	(6.21,9.47)	0.17		7.75	*	*		8.08	(2.99,10.0ǂ)	0.46	

Table S7c. Childhood predictors regression analysis results for Hong Kong

		Cantril's Ladder				Life Satisfaction				Happiness			
Variable	Category	Est	95% CI	SE	Global p-value	Est	95% CI	SE	Global p-value	Est	95% CI	SE	Global p-value
Relationship with mother	(Ref: Very bad/somewhat bad)				0.729				0.133				0.202
	Very good/somewhat good	0.04	(-0.18, 0.25)	0.11		0.20	(-0.07, 0.46)	0.13		0.15	(-0.08, 0.38)	0.12	
Relationship with father	(Ref: Very bad/somewhat bad)				0.006				0.157				0.008
	Very good/somewhat good	0.30	(0.08, 0.52)	0.11		0.18	(-0.07, 0.43)	0.13		0.32	(0.08, 0.55)	0.12	
Parent marital status	(Ref: Parents married)				0.254				0.654				0.469
	Divorced	0.08	(-0.42, 0.59)	0.26		-0.13	(-0.61, 0.35)	0.25		-0.12	(-0.62, 0.38)	0.25	
	Parents were never married	0.64	(-0.11, 1.38)	0.38		0.45	(-0.40, 1.30)	0.43		0.47	(-0.20, 1.14)	0.34	
	One or both parents had died	-0.24	(-0.82, 0.33)	0.29		-0.23	(-1.10, 0.64)	0.44		-0.23	(-1.02, 0.57)	0.41	
Subjective financial status of family growing up	(Ref: Got by)				<.001				<.001				<.001
	Lived comfortably	0.93	(0.72, 1.13)	0.10		0.82	(0.60, 1.04)	0.11		0.79	(0.61, 0.98)	0.10	
	Found it difficult	-0.49	(-0.78, -0.20)	0.15		-0.48	(-0.79, -0.17)	0.16		-0.24	(-0.51, 0.02)	0.14	
	Found it very difficult	-1.32	(-1.90, -0.75)	0.29		-0.81	(-1.48, -0.14)	0.34		-0.47	(-1.21, 0.27)	0.38	
Abuse	(Ref: No)				0.303				0.689				0.858
	Yes	0.14	(-0.13, 0.41)	0.14		-0.05	(-0.33, 0.22)	0.14		-0.03	(-0.32, 0.27)	0.15	
Outsider growing up	(Ref: No)				0.065				0.069				0.808
	Yes	0.18	(-0.01, 0.38)	0.10		-0.21	(-0.46, 0.03)	0.12		-0.02	(-0.23, 0.20)	0.11	
Self-rated health growing up	(Ref: Good)				<.001				<.001				<.001
	Excellent	1.37	(1.01, 1.74)	0.18		1.50	(1.13, 1.87)	0.19		1.58	(1.28, 1.88)	0.15	

Variable	Category	Cantril's Ladder				Life Satisfaction				Happiness			
		Est	95% CI	SE	Global p-value	Est	95% CI	SE	Global p-value	Est	95% CI	SE	Global p-value
	Very good	0.69	(0.51, 0.87)	0.09		0.75	(0.53, 0.97)	0.11		0.81	(0.62, 1.01)	0.10	
	Fair	-0.60	(-0.86, -0.34)	0.13		-0.72	(-1.01, -0.44)	0.15		-0.74	(-1.00, -0.49)	0.13	
	Poor	-1.44	(-2.22, -0.66)	0.40		-1.14	(-1.92, -0.36)	0.40		-1.53	(-2.42, -0.63)	0.46	
Immigration status	(Ref: Born in this country)				0.120				0.935				0.200
	Born in another country	-0.26	(-0.58, 0.07)	0.17		0.00	(-0.33, 0.33)	0.17		-0.22	(-0.55, 0.12)	0.17	
Age 12 religious service attendance	(Ref: Never)				<.001				<.001				<.001
	At least 1/week	0.61	(0.29, 0.93)	0.16		0.57	(0.19, 0.95)	0.19		0.62	(0.30, 0.94)	0.16	
	1-3/month	0.81	(0.57, 1.05)	0.12		0.55	(0.27, 0.83)	0.14		0.73	(0.45, 1.00)	0.14	
	< 1/month	0.12	(-0.10, 0.34)	0.11		0.02	(-0.21, 0.24)	0.11		0.20	(-0.01, 0.41)	0.11	
Year of birth	(Ref: 1998-2005; current age: 18-24)				<.001				<.001				<.001
	1993-1998; age 25-29	-0.04	(-0.37, 0.30)	0.17		-0.24	(-0.58, 0.09)	0.17		-0.05	(-0.39, 0.28)	0.17	
	1983-1993; age 30-39	-0.04	(-0.29, 0.21)	0.13		-0.10	(-0.38, 0.19)	0.15		-0.01	(-0.27, 0.25)	0.13	
	1973-1983; age 40-49	0.23	(-0.02, 0.47)	0.12		0.22	(-0.05, 0.49)	0.14		0.22	(-0.01, 0.46)	0.12	
	1963-1973; age 50-59	0.55	(0.32, 0.78)	0.12		0.54	(0.27, 0.81)	0.14		0.48	(0.25, 0.71)	0.12	
	1953-1963; age 60-69	0.78	(0.47, 1.09)	0.16		1.01	(0.67, 1.36)	0.18		0.81	(0.53, 1.08)	0.14	
	1943-1953; age 70-79	0.96	(0.44, 1.48)	0.27		1.13	(0.54, 1.72)	0.30		1.31	(0.79, 1.83)	0.26	
	1943 or earlier; age 80+	-1.51	(-2.01, -1.01)	0.26		-1.77	(-2.75, -0.78)	0.50		-1.02	(-2.01, -0.04)	0.50	
Gender	(Ref: Male)				<.001				<.001				<.001
	Female	0.12	(-0.02, 0.26)	0.07		0.21	(0.06, 0.37)	0.08		0.14	(-0.00, 0.29)	0.07	
	Other	-1.16	(-1.46, -0.87)	0.15		-0.86	(-1.39, -0.33)	0.27		-1.51	(-1.81, -1.21)	0.15	
Religious affiliation	(Ref: No religion/Atheist /Agnostic)				0.023				0.220				0.250
	Buddhism	0.13	(-0.15, 0.40)	0.14		-0.04	(-0.33, 0.26)	0.15		0.04	(-0.23, 0.31)	0.14	

Variable	Category	Cantril's Ladder				Life Satisfaction				Happiness			
		Est	95% CI	SE	Global p-value	Est	95% CI	SE	Global p-value	Est	95% CI	SE	Global p-value
	Chinese folk/traditional religion	0.08	(-0.20, 0.37)	0.15		0.16	(-0.15, 0.47)	0.16		0.07	(-0.23, 0.37)	0.15	
	Christianity	-0.02	(-0.30, 0.26)	0.14		-0.22	(-0.53, 0.10)	0.16		-0.15	(-0.40, 0.10)	0.13	
	Collapsed affiliations with prevalence<3%	0.42	(0.13, 0.71)	0.15		0.10	(-0.24, 0.44)	0.17		0.19	(-0.20, 0.58)	0.20	
Race/ethnicity	(Ref: Plurality group)				0.740				0.644				0.310
	Non-plurality groups	0.01	(-0.16, 0.19)	0.09		0.04	(-0.16, 0.23)	0.10		0.08	(-0.08, 0.25)	0.08	

Table S7d. Sensitivity to unmeasured confounding of childhood predictors in Hong Kong

		Cantril's Ladder		Life Satisfaction		Happiness	
Variable	Category	E-value for Estimate	E-value for 95% CI	E-value for Estimate	E-value for 95% CI	E-value for Estimate	E-value for 95% CI
Relationship with mother	(Ref: Very bad/somewhat bad)						
	Very good/somewhat good	1.15	1.00	1.40	1.00	1.35	1.00
Relationship with father	(Ref: Very bad/somewhat bad)						
	Very good/somewhat good	1.55	1.24	1.38	1.00	1.58	1.23
Parent marital status	(Ref: Parents married)						
	Divorced	1.24	1.00	1.31	1.00	1.30	1.00
	Parents were never married	2.00	1.00	1.73	1.00	1.78	1.00
	One or both parents had died	1.47	1.00	1.45	1.00	1.46	1.00
Subjective financial status of family growing up	(Ref: Got by)						
	Lived comfortably	2.41	2.12	2.22	1.93	2.23	1.97
	Found it difficult	1.80	1.42	1.77	1.37	1.48	1.00
	Found it very difficult	3.03	2.15	2.21	1.33	1.79	1.00
Abuse	(Ref: No)						
	Yes	1.33	1.00	1.18	1.00	1.12	1.00
Outsider growing up	(Ref: No)						
	Yes	1.39	1.00	1.43	1.00	1.10	1.00
Self-rated health growing up	(Ref: Good)						
	Excellent	3.12	2.53	3.26	2.66	3.53	2.99
	Very good	2.07	1.82	2.12	1.84	2.26	1.98

Variable	Category	Cantril's Ladder		Life Satisfaction		Happiness	
		E-value for Estimate	E-value for 95% CI	E-value for Estimate	E-value for 95% CI	E-value for Estimate	E-value for 95% CI
	Fair	1.95	1.60	2.08	1.72	2.16	1.81
	Poor	3.23	2.03	2.68	1.62	3.42	2.01
Immigration status	(Ref: Born in this country)						
	Born in another country	1.49	1.00	1.04	1.00	1.45	1.00
Age 12 religious service attendance	(Ref: Never)						
	At least 1/week	1.96	1.54	1.89	1.40	1.98	1.55
	1-3/month	2.24	1.91	1.86	1.50	2.13	1.76
	< 1/month	1.30	1.00	1.10	1.00	1.42	1.00
Year of birth	(Ref: 1998-2005; current age: 18-24)						
	1993-1998; age 25-29	1.14	1.00	1.46	1.00	1.18	1.00
	1983-1993; age 30-39	1.16	1.00	1.25	1.00	1.07	1.00
	1973-1983; age 40-49	1.45	1.00	1.43	1.00	1.45	1.00
	1963-1973; age 50-59	1.89	1.58	1.85	1.50	1.79	1.49
	1953-1963; age 60-69	2.19	1.78	2.49	2.01	2.25	1.87
	1943-1953; age 70-79	2.45	1.73	2.66	1.84	3.03	2.22
	1943 or earlier; age 80+	3.36	2.53	3.75	2.17	2.56	1.15
Gender	(Ref: Male)						
	Female	1.30	1.00	1.42	1.18	1.34	1.00
	Other	2.77	2.32	2.27	1.58	3.39	2.86
Religious affiliation	(Ref: No religion/Atheist/Agnostic)						
	Buddhism	1.31	1.00	1.14	1.00	1.15	1.00

Variable	Category	Cantril's Ladder		Life Satisfaction		Happiness	
		E-value for Estimate	E-value for 95% CI	E-value for Estimate	E-value for 95% CI	E-value for Estimate	E-value for 95% CI
	Chinese folk/traditional religion	1.24	1.00	1.35	1.00	1.22	1.00
	Christianity	1.11	1.00	1.43	1.00	1.35	1.00
	Collapsed affiliations with prevalence<3%	1.71	1.32	1.26	1.00	1.40	1.00
Race/ethnicity	(Ref: Plurality group)						
	Non-plurality groups	1.08	1.00	1.14	1.00	1.24	1.00

Table S7e. Complete-case supplemental analysis of means by demographic category for Hong Kong

		Cantril's Ladder				Life Satisfaction				Happiness			
Variable	Category	Mean	95% CI	SE	Global p-value	Mean	95% CI	SE	Global p-value	Mean	95% CI	SE	Global p-value
Age group	18-24	6.94	(6.70,7.18)	0.12	< .001	7.06	(6.80,7.33)	0.13	< .001	7.26	(7.03,7.49)	0.12	< .001
	25-29	6.60	(6.15,7.05)	0.23		6.47	(6.08,6.87)	0.20		7.04	(6.71,7.36)	0.16	
	30-39	6.31	(6.10,6.52)	0.10		6.45	(6.24,6.65)	0.10		6.69	(6.48,6.90)	0.11	
	40-49	6.87	(6.67,7.06)	0.10		7.02	(6.83,7.21)	0.10		7.16	(6.97,7.34)	0.09	
	50-59	7.30	(7.15,7.45)	0.08		7.42	(7.24,7.60)	0.09		7.52	(7.37,7.67)	0.08	
	60-69	7.13	(6.89,7.36)	0.12		7.59	(7.34,7.84)	0.13		7.46	(7.22,7.69)	0.12	
	70-79	6.76	(6.20,7.31)	0.28		7.31	(6.87,7.76)	0.23		7.50	(7.07,7.94)	0.22	
	80 or older	5.00	(5.00,5.00)	0.00		4.65	(3.92,5.38)	0.28		5.65	(4.92,6.38)	0.28	
Gender	Female	6.83	(6.70,6.96)	0.07	< .001	7.06	(6.92,7.19)	0.07	0.114	7.18	(7.06,7.30)	0.06	< .001
	Male	6.98	(6.86,7.11)	0.06		7.17	(7.04,7.30)	0.07		7.31	(7.19,7.44)	0.06	
	Other	5.45	(0.00,1044.03)	0.35		5.91	(0.00,2083.06)	0.70		5.45	(0.00,1044.03)	0.35	
Marital status	Divorced	6.95	(6.28,7.63)	0.34	< .001	6.83	(6.20,7.46)	0.32	< .001	7.14	(6.58,7.70)	0.28	< .001
	Domestic partner	6.42	(5.52,7.32)	0.44		6.30	(5.74,6.85)	0.27		6.37	(5.78,6.96)	0.29	
	Married	7.25	(7.15,7.36)	0.05		7.43	(7.31,7.54)	0.06		7.55	(7.45,7.65)	0.05	
	Separated	6.57	(4.09,9.05)	1.14		7.06	(6.21,7.92)	0.39		7.72	(6.30,9.13)	0.65	
	Single/Never been married	5.92	(5.75,6.09)	0.09		6.22	(6.05,6.40)	0.09		6.40	(6.23,6.57)	0.09	
	Widowed	6.66	(5.69,7.63)	0.48		7.50	(6.98,8.02)	0.26		6.77	(6.12,7.43)	0.32	
Employment	Employed for an employer	6.95	(6.85,7.04)	0.05	< .001	7.12	(7.02,7.22)	0.05	< .001	7.27	(7.18,7.37)	0.05	< .001
	Homemaker	6.62	(6.14,7.10)	0.24		6.74	(6.17,7.31)	0.29		6.97	(6.43,7.50)	0.27	
	None of these/Other	5.77	(4.09,7.46)	0.82		5.40	(3.98,6.82)	0.69		6.19	(5.22,7.17)	0.48	
	Retired	6.59	(6.24,6.93)	0.17		7.18	(6.86,7.49)	0.16		7.12	(6.83,7.41)	0.15	
	Self-employed	7.80	(7.50,8.10)	0.15		7.77	(7.45,8.09)	0.16		7.90	(7.62,8.19)	0.15	
	Student	6.33	(5.86,6.80)	0.23		6.64	(6.09,7.20)	0.28		6.64	(6.10,7.17)	0.26	
	Unemployed and looking for a job	5.77	(4.90,6.65)	0.44		5.69	(4.93,6.46)	0.38		5.90	(5.09,6.70)	0.40	
Religious service attendance	A few times a year	6.68	(6.45,6.91)	0.12	< .001	6.78	(6.56,7.00)	0.11	< .001	6.87	(6.65,7.09)	0.11	< .001
	More than once a week	9.03	(8.82,9.25)	0.11		8.85	(8.62,9.07)	0.11		9.23	(9.07,9.40)	0.09	
	Never	6.32	(6.19,6.44)	0.06		6.74	(6.60,6.87)	0.07		6.75	(6.63,6.88)	0.06	
	Once a week	7.36	(7.13,7.59)	0.12		7.49	(7.24,7.73)	0.13		7.66	(7.46,7.87)	0.11	

Variable	Category	Cantril's Ladder				Life Satisfaction				Happiness			
		Mean	95% CI	SE	Global p-value	Mean	95% CI	SE	Global p-value	Mean	95% CI	SE	Global p-value
	One to three times a month	7.28	(7.02,7.54)	0.13		7.19	(6.90,7.48)	0.15		7.62	(7.42,7.83)	0.10	
Education	Up to 8 years	7.19	(6.84,7.54)	0.18	< .001	7.36	(7.00,7.72)	0.18	< .001	7.58	(7.29,7.88)	0.15	< .001
	16+ years	6.58	(6.43,6.73)	0.08		6.77	(6.60,6.94)	0.09		6.87	(6.71,7.04)	0.08	
	9 to 15 years	6.93	(6.82,7.03)	0.05		7.14	(7.04,7.25)	0.05		7.26	(7.16,7.36)	0.05	
Immigration status	Born in another country	6.28	(5.86,6.70)	0.22	0.002	6.73	(6.34,7.13)	0.20	0.044	6.83	(6.47,7.19)	0.18	0.016
	Born in this country	6.98	(6.89,7.07)	0.04		7.15	(7.06,7.25)	0.05		7.29	(7.20,7.37)	0.04	
Religious affiliation	Buddhism	7.57	(7.25,7.89)	0.16	< .001	7.68	(7.39,7.98)	0.15	< .001	7.88	(7.60,8.16)	0.14	< .001
	Christianity	7.15	(6.98,7.33)	0.09		7.21	(7.03,7.40)	0.10		7.40	(7.24,7.57)	0.08	
	Confucianism	7.63	*	*		7.74	*	*		7.34	*	*	
	Hinduism	8.28	(7.06,9.51)	0.39		7.29	(4.88,9.70)	0.76		8.23	(7.21,9.26)	0.32	
	Islam	7.69	(6.82,8.55)	0.44		7.47	(6.44,8.50)	0.52		8.36	(7.79,8.93)	0.29	
	Judaism	8.17	*	*		7.59	*	*		8.74	*	*	
	No religion/Atheist/Agnostic	6.50	(6.38,6.62)	0.06		6.88	(6.75,7.00)	0.06		6.90	(6.78,7.02)	0.06	
	Primal, Animist, or Folk religion	6.31	(5.73,6.89)	0.26		6.81	(6.16,7.46)	0.29		6.71	(6.02,7.39)	0.30	
	Sikhism	9.08	*	*		8.82	*	*		9.26	*	*	
	Some other religion	9.23	*	*		6.00	*	*		8.84	*	*	
	Taoism	7.08	(6.53,7.64)	0.28		6.65	(6.01,7.29)	0.32		6.89	(6.30,7.48)	0.30	
	Baha'i	8.86	*	*		8.36	*	*		8.91	*	*	
	Jainism	10.00	*	*		8.00	*	*		10.00	*	*	
	Shinto	7.71	(6.68,8.73)	0.24		8.44	(7.39,9.49)	0.24		8.44	(7.24,9.63)	0.28	
	Chinese folk/traditional religion	7.29	(6.92,7.65)	0.18		7.48	(7.08,7.88)	0.20		7.69	(7.37,8.02)	0.16	

Table S7f. Complete-case supplemental analysis of childhood predictors regression analysis results for Hong Kong

		Cantril's Ladder				Life Satisfaction				Happiness			
Variable	Category	Est	95% CI	SE	Global p-value	Est	95% CI	SE	Global p-value	Est	95% CI	SE	Global p-value
Relationship with mother	(Ref: Very bad/somewhat bad)				0.541				0.138				0.123
	Very good/somewhat good	0.07	(-0.15,0.28)	0.11		0.19	(-0.06,0.44)	0.13		0.17	(-0.05,0.40)	0.11	
Relationship with father	(Ref: Very bad/somewhat bad)				0.005				0.147				0.003
	Very good/somewhat good	0.30	(0.09,0.51)	0.11		0.18	(-0.06,0.43)	0.13		0.33	(0.11,0.55)	0.11	
Parent marital status	(Ref: Parents married)				0.164				0.686				0.529
	No, one or both of them had died	-0.18	(-0.79,0.43)	0.31		-0.13	(-1.04,0.77)	0.46		-0.14	(-0.98,0.71)	0.43	
	No, they were never married	0.41	(-0.39,1.20)	0.40		0.39	(-0.50,1.28)	0.45		0.34	(-0.36,1.04)	0.36	
	Yes, married	-0.26	(-0.71,0.19)	0.23		-0.08	(-0.49,0.34)	0.21		-0.07	(-0.51,0.36)	0.22	
Subjective financial status of family growing up	(Ref: Got by)				<.001				<.001				<.001
	Found it difficult	-0.52	(-0.80,-0.24)	0.14		-0.55	(-0.84,-0.26)	0.15		-0.23	(-0.47,0.02)	0.12	
	Found it very difficult	-1.33	(-1.86,-0.81)	0.27		-0.68	(-1.27,-0.09)	0.30		-0.36	(-0.98,0.27)	0.32	
	Lived comfortably	0.92	(0.75,1.08)	0.09		0.82	(0.64,1.00)	0.09		0.87	(0.69,1.04)	0.09	
Abuse	(Ref: No)				0.094				0.425				0.128
	Yes	0.22	(-0.04,0.48)	0.13		0.10	(-0.15,0.34)	0.12		0.19	(-0.05,0.43)	0.12	
Outsider growing up	(Ref: No)				0.049				0.090				0.822
	Yes	0.18	(0.00,0.36)	0.09		-0.19	(-0.41,0.03)	0.11		-0.02	(-0.21,0.17)	0.10	

		Cantril's Ladder				Life Satisfaction				Happiness			
Variable	Category	Est	95% CI	SE	Global p-value	Est	95% CI	SE	Global p-value	Est	95% CI	SE	Global p-value
Self-rated health growing up	(Ref: Good)				<.001				<.001				<.001
	Excellent	1.51	(1.25,1.77)	0.13		1.59	(1.33,1.85)	0.13		1.60	(1.31,1.89)	0.15	
	Fair	-0.51	(-0.77,-0.26)	0.13		-0.63	(-0.90,-0.36)	0.14		-0.65	(-0.89,-0.40)	0.12	
	Poor	-1.12	(-1.84,-0.39)	0.37		-0.73	(-1.46,0.00)	0.37		-0.73	(-1.34,-0.11)	0.32	
	Very good	0.68	(0.50,0.85)	0.09		0.74	(0.53,0.95)	0.11		0.80	(0.61,0.99)	0.10	
Immigration status	(Ref: Born in this country)				0.056				0.718				0.160
	Born in another country	-0.31	(-0.63,0.01)	0.16		-0.06	(-0.38,0.26)	0.16		-0.22	(-0.52,0.09)	0.16	
Age 12 religious service attendance	(Ref: Never)				<.001				0.003				<.001
	At least once a week	0.48	(0.21,0.76)	0.14		0.42	(0.10,0.74)	0.16		0.48	(0.19,0.77)	0.15	
	Less than once a month	0.14	(-0.07,0.34)	0.11		0.00	(-0.20,0.21)	0.10		0.13	(-0.06,0.32)	0.10	
	One to three times a month	0.72	(0.49,0.96)	0.12		0.40	(0.14,0.66)	0.13		0.54	(0.31,0.77)	0.12	
Year of birth	(Ref: 1998-2005; current age: 18-24)				<.001				<.001				<.001
	1993-1998; age 25-29	-0.05	(-0.39,0.29)	0.17		-0.25	(-0.60,0.09)	0.17		0.05	(-0.23,0.33)	0.14	
	1983-1993; age 30-39	0.03	(-0.21,0.27)	0.12		-0.02	(-0.30,0.25)	0.14		0.05	(-0.20,0.29)	0.12	
	1973-1983; age 40-49	0.33	(0.09,0.57)	0.12		0.32	(0.06,0.58)	0.13		0.30	(0.08,0.52)	0.11	
	1963-1973; age 50-59	0.61	(0.38,0.83)	0.11		0.56	(0.30,0.82)	0.13		0.49	(0.27,0.71)	0.11	
	1953-1963; age 60-69	0.85	(0.57,1.12)	0.14		1.06	(0.76,1.37)	0.16		0.75	(0.49,1.02)	0.14	
	1943-1953; age 70-79	0.96	(0.46,1.47)	0.26		1.07	(0.49,1.64)	0.29		1.19	(0.69,1.69)	0.26	

		Cantril's Ladder				Life Satisfaction				Happiness			
Variable	Category	Est	95% CI	SE	Global p-value	Est	95% CI	SE	Global p-value	Est	95% CI	SE	Global p-value
	1943 or earlier; age 80+	-1.47	(-1.92,-1.01)	0.23		-1.66	(-2.58,-0.75)	0.47		-0.97	(-1.88,-0.06)	0.47	
Gender	(Ref: Male)				<.001				<.001				<.001
	Male	-0.08	(-0.21,0.05)	0.07		-0.14	(-0.29,-0.00)	0.07		-0.11	(-0.25,0.03)	0.07	
	Other	-1.18	(-1.42,-0.95)	0.12		-1.02	(-1.59,-0.45)	0.29		-1.63	(-1.90,-1.37)	0.14	
Religious affiliation	(Ref: No religion/Atheist/Agnostic)				0.012				0.460				0.147
	Buddhism	0.12	(-0.16,0.40)	0.14		-0.02	(-0.31,0.27)	0.15		0.09	(-0.18,0.35)	0.13	
	Christianity	0.14	(-0.08,0.35)	0.11		-0.03	(-0.27,0.22)	0.13		0.03	(-0.19,0.25)	0.11	
	Christianity	0.14	(-0.08,0.35)	0.11		-0.03	(-0.27,0.22)	0.13		0.03	(-0.19,0.25)	0.11	
	Chinese folk/traditional religion	0.11	(-0.17,0.39)	0.14		0.21	(-0.10,0.52)	0.16		0.20	(-0.08,0.47)	0.14	
	Collapsed affiliations with prevalence<3%	0.51	(0.23,0.79)	0.14		0.17	(-0.15,0.50)	0.17		0.39	(0.04,0.73)	0.18	
Race/ethnicity	(Ref: Plurality group)												

Table S7g. Complete-case supplemental analysis of sensitivity to unmeasured confounding of childhood predictors in Hong Kong

Variable	Category	Cantril's Ladder		Life Satisfaction		Happiness	
		E-value for Estimate	E-value for 95% CI	E-value for Estimate	E-value for 95% CI	E-value for Estimate	E-value for 95% CI
Relationship with mother	(Ref: Very bad/somewhat bad)						
	Very good/somewhat good	1.21	1.00	1.41	1.00	1.40	1.00
Relationship with father	(Ref: Very bad/somewhat bad)						
	Very good/somewhat good	1.57	1.26	1.40	1.00	1.63	1.30
Parent marital status	(Ref: Parents married)						
	No, one or both of them had died	1.40	1.00	1.32	1.00	1.34	1.00
	No, they were never married	1.71	1.00	1.69	1.00	1.64	1.00
	Yes, married	1.51	1.00	1.23	1.00	1.23	1.00
Subjective financial status of family growing up	(Ref: Got by)						
	Found it difficult	1.87	1.49	1.91	1.51	1.48	1.00
	Found it very difficult	3.15	2.29	2.09	1.26	1.66	1.00
	Lived comfortably	2.45	2.20	2.29	2.03	2.42	2.15
Abuse	(Ref: No)						
	Yes	1.46	1.00	1.27	1.00	1.42	1.00
Outsider growing up	(Ref: No)						
	Yes	1.40	1.02	1.41	1.00	1.12	1.00
Self-rated health growing up	(Ref: Good)						
	Excellent	3.49	3.00	3.62	3.13	3.78	3.19
	Fair	1.87	1.52	2.02	1.65	2.09	1.73
	Poor	2.78	1.70	2.15	1.00	2.20	1.29
	Very good	2.10	1.85	2.18	1.88	2.32	2.04
Immigration status	(Ref: Born in this country)						
	Born in another country	1.58	1.00	1.20	1.00	1.47	1.00
Age 12 religious service attendance	(Ref: Never)						
	At least once a week	1.82	1.44	1.73	1.28	1.84	1.43
	Less than once a month	1.33	1.00	1.04	1.00	1.33	1.00
	One to three times a month	2.16	1.84	1.70	1.34	1.93	1.61
Year of birth	(Ref: 1998-2005; current age: 18-24)						

Variable	Category	Cantril's Ladder		Life Satisfaction		Happiness	
		E-value for Estimate	E-value for 95% CI	E-value for Estimate	E-value for 95% CI	E-value for Estimate	E-value for 95% CI
	1993-1998; age 25-29	1.18	1.00	1.50	1.00	1.19	1.00
	1983-1993; age 30-39	1.13	1.00	1.12	1.00	1.17	1.00
	1973-1983; age 40-49	1.61	1.26	1.59	1.20	1.58	1.24
	1963-1973; age 50-59	1.99	1.68	1.92	1.56	1.85	1.54
	1953-1963; age 60-69	2.34	1.95	2.67	2.20	2.25	1.85
	1943-1953; age 70-79	2.52	1.79	2.67	1.82	2.97	2.14
	1943 or earlier; age 80+	3.40	2.60	3.76	2.19	2.59	1.20
Gender	(Ref: Male)						
	Male	1.24	1.00	1.34	1.02	1.29	1.00
	Other	2.89	2.51	2.60	1.77	3.86	3.30
Religious affiliation	(Ref: No religion/Atheist/Agnostic)						
	Buddhism	1.30	1.00	1.11	1.00	1.25	1.00
	Christianity	1.34	1.00	1.13	1.00	1.14	1.00
	Christianity	1.34	1.00	1.13	1.00	1.14	1.00
	Chinese folk/traditional religion	1.29	1.00	1.44	1.00	1.43	1.00
	Collapsed affiliations with prevalence<3%	1.85	1.47	1.39	1.00	1.71	1.17
Race/ethnicity	(Ref: Plurality group)						

Tables S8a-g: India

Table S8a. Nationally representative descriptive statistics for India

Characteristic	N = 12,765[1]
**Age group**	
1998-2005; age 18-24	2,543 (20%)
1993-1998; age 25-29	1,640 (13%)
1983-1993; age 30-39	3,109 (24%)
1973-1983; age 40-49	2,275 (18%)
1963-1973; age 50-59	1,574 (12%)
1953-1963; age 60-69	1,188 (9.3%)
1943-1953; age 70-79	370 (2.9%)
1943 or earlier; age 80+	67 (0.5%)
(Missing)	0 (0%)
**Gender**	
Male	6,473 (51%)
Female	6,292 (49%)
Other	0 (0%)
(Missing)	0 (0%)
**Race/Ethnicity**	
General	3,538 (28%)
Other backward caste	4,177 (33%)
Schedule caste	3,599 (28%)
Schedule tribe	1,185 (9.3%)
(Missing)	267 (2.1%)
**Respondent Marital status**	
Married	9,848 (77%)
Separated	45 (0.4%)
Divorced	25 (0.2%)
Widowed	445 (3.5%)
Single, never married	2,065 (16%)
Domestic Partner	269 (2.1%)
(Missing)	69 (0.5%)
**Employment**	
Employed for an employer	2,660 (21%)
Self-employed	3,401 (27%)
Retired	286 (2.2%)
Student	532 (4.2%)
Homemaker	4,221 (33%)
Unemployed and looking for a job	902 (7.1%)
None of these/Other	715 (5.6%)
(Missing)	48 (0.4%)
**Religious service attendance as an adult (now)**	
More than 1/week	2,875 (23%)
1/week	3,166 (25%)
1-3/month	2,740 (21%)
A few times a year	2,090 (16%)
Never	1,823 (14%)
(Missing)	71 (0.6%)
**Education (years)**	
Up to 8 years	11,422 (89%)
9-15 years	1,194 (9.4%)
16+ years	145 (1.1%)
(Missing)	4 (<0.1%)
**Immigration status**	
Born in this country	12,629 (99%)
Born in another country	110 (0.9%)
(Missing)	26 (0.2%)
**Religious affiliation as an adult (now)**	
Christianity	306 (2.4%)
Islam	1,555 (12%)
Hinduism	10,362 (81%)
Buddhism	230 (1.8%)
Judaism	0 (0%)
Sikhism	127 (1.0%)

Characteristic	N = 12,765[1]
Baha'i	0 (0%)
Jainism	10 (<0.1%)
Shinto	1 (<0.1%)
Taoism	0 (0%)
Confucianism	0 (0%)
Primal, Animist, or Folk religion	30 (0.2%)
Spiritism	0 (0%)
Umbanda, Candomble, and other African-derived religions	0 (0%)
Chinese folk/traditional religion	0 (0%)
Some other religion	67 (0.5%)
No religion/Atheist/Agnostic	13 (0.1%)
(Missing)	62 (0.5%)
**Relationship with mother growing up**	
Very good	11,465 (90%)
Somewhat good	788 (6.2%)
Somewhat bad	88 (0.7%)
Very bad	73 (0.6%)
Does not apply	269 (2.1%)
(Missing)	82 (0.6%)
**Relationship with father growing up**	
Very good	10,923 (86%)
Somewhat good	995 (7.8%)
Somewhat bad	126 (1.0%)
Very bad	100 (0.8%)
Does not apply	481 (3.8%)
(Missing)	141 (1.1%)
**Parent marital status at age 12**	
Parents married	5,578 (44%)
Divorced	236 (1.8%)
Parents were never married	1,055 (8.3%)
One or both parents had died	940 (7.4%)
(Missing)	4,956 (39%)
**Subjective financial status of family growing up**	
Lived comfortably	4,946 (39%)
Got by	3,010 (24%)
Found it difficult	2,703 (21%)
Found it very difficult	2,035 (16%)
(Missing)	70 (0.5%)
**Abuse**	
Yes	1,468 (11%)
No	10,526 (82%)
(Missing)	771 (6.0%)
**Outsider growing up**	
Yes	1,926 (15%)
No	10,780 (84%)
(Missing)	59 (0.5%)
**Self-rated health growing up**	
Excellent	2,182 (17%)
Very good	3,882 (30%)
Good	4,028 (32%)
Fair	2,202 (17%)
Poor	424 (3.3%)
(Missing)	47 (0.4%)
**Age 12 religious service attendance**	
At least 1/week	5,288 (41%)
1-3/month	2,959 (23%)
<1/month	2,719 (21%)
Never	1,478 (12%)
(Missing)	321 (2.5%)
**Religious affiliation at age 12**	
Christianity	254 (2.0%)
Islam	1,550 (12%)
Hinduism	10,417 (82%)
Buddhism	180 (1.4%)
Judaism	0 (0%)

**Characteristic**	**N = 12,765**[1]
Sikhism	126 (1.0%)
Baha'i	0 (0%)
Jainism	9 (<0.1%)
Shinto	4 (<0.1%)
Taoism	0 (0%)
Confucianism	0 (0%)
Primal, Animist, or Folk religion	27 (0.2%)
Spiritism	0 (0%)
Umbanda, Candomble, and other African-derived religions	0 (0%)
Chinese folk/traditional religion	0 (0%)
Some other religion	59 (0.5%)
No religion/Atheist/Agnostic	7 (<0.1%)
(Missing)	131 (1.0%)

[1]n (%)

Table S8b. Means by demographic category for India

Variable	Category	Cantril's Ladder				Life Satisfaction				Happiness			
		Mean	95% CI	SE	Global p-value	Mean	95% CI	SE	Global p-value	Mean	95% CI	SE	Global p-value
Age group	18-24	6.11	(5.91,6.32)	0.10	< .001	7.51	(7.32,7.70)	0.10	< .001	7.18	(6.99,7.36)	0.09	< .001
	25-29	5.65	(5.44,5.85)	0.11		7.13	(6.94,7.33)	0.10		6.63	(6.43,6.82)	0.10	
	30-39	5.54	(5.39,5.69)	0.08		6.94	(6.80,7.08)	0.07		6.43	(6.27,6.58)	0.08	
	40-49	5.40	(5.23,5.57)	0.09		6.81	(6.65,6.97)	0.08		6.23	(6.05,6.40)	0.09	
	50-59	5.33	(5.11,5.55)	0.11		6.62	(6.41,6.83)	0.11		5.99	(5.76,6.21)	0.11	
	60-69	5.49	(5.22,5.75)	0.14		6.75	(6.49,7.02)	0.14		5.98	(5.69,6.27)	0.15	
	70-79	5.77	(5.33,6.22)	0.23		6.92	(6.47,7.38)	0.23		6.53	(6.05,7.02)	0.25	
	80 or older	6.47	(5.29,7.66)	0.59		7.71	(6.53,8.89)	0.59		6.87	(5.57,8.16)	0.65	
Gender	Male	5.40	(5.27,5.52)	0.06	< .001	6.87	(6.75,6.99)	0.06	< .001	6.38	(6.27,6.50)	0.06	0.011
	Female	5.86	(5.74,5.98)	0.06		7.14	(7.03,7.25)	0.06		6.57	(6.45,6.69)	0.06	
Marital status	Married	5.64	(5.55,5.74)	0.05	< .001	7.04	(6.95,7.13)	0.05	< .001	6.48	(6.38,6.57)	0.05	< .001
	Separated	4.29	(2.95,5.64)	0.66		5.40	(3.90,6.90)	0.73		5.34	(3.92,6.76)	0.70	
	Divorced	3.29	(1.67,4.91)	0.76		6.04	(3.63,8.44)	1.14		3.89	(2.09,5.68)	0.85	
	Widowed	4.82	(4.40,5.25)	0.22		5.59	(5.11,6.06)	0.24		5.06	(4.61,5.50)	0.23	
	Never	5.85	(5.66,6.05)	0.10		7.20	(7.00,7.41)	0.10		6.88	(6.69,7.06)	0.10	
	Domestic Partner	5.03	(4.35,5.70)	0.34		6.85	(6.19,7.52)	0.34		6.10	(5.49,6.71)	0.31	
Employment	Employed for an employer	5.20	(5.01,5.38)	0.09	< .001	6.71	(6.54,6.89)	0.09	< .001	6.18	(6.01,6.35)	0.09	< .001
	Self-employed	5.70	(5.54,5.86)	0.08		7.14	(6.97,7.30)	0.08		6.70	(6.54,6.85)	0.08	
	Retired	5.87	(5.37,6.37)	0.25		6.97	(6.42,7.52)	0.28		6.68	(6.14,7.22)	0.27	
	Student	6.20	(5.84,6.56)	0.18		7.20	(6.86,7.54)	0.17		7.00	(6.68,7.32)	0.16	
	Homemaker	5.87	(5.73,6.01)	0.07		7.17	(7.04,7.30)	0.06		6.47	(6.34,6.61)	0.07	
	Unemployed and looking for a job	5.20	(4.90,5.50)	0.15		6.62	(6.30,6.94)	0.16		6.25	(5.96,6.54)	0.15	
	None of these/Other	5.43	(5.05,5.82)	0.20		6.78	(6.42,7.14)	0.18		6.35	(5.96,6.73)	0.19	
Religious service attendance	More than 1/week	5.61	(5.43,5.78)	0.09	0.193	7.01	(6.84,7.18)	0.09	< .001	6.41	(6.24,6.59)	0.09	0.002
	1/week	5.78	(5.61,5.96)	0.09		7.29	(7.13,7.44)	0.08		6.66	(6.50,6.82)	0.08	
	1-3/month	5.62	(5.45,5.79)	0.09		7.05	(6.89,7.22)	0.08		6.59	(6.43,6.75)	0.08	
	A few times a year	5.49	(5.33,5.65)	0.08		6.86	(6.68,7.04)	0.09		6.41	(6.24,6.58)	0.09	
	Never	5.54	(5.32,5.77)	0.11		6.59	(6.36,6.82)	0.12		6.15	(5.93,6.38)	0.11	
Education	Up to 8 years	5.61	(5.51,5.70)	0.05	0.022	6.96	(6.87,7.06)	0.05	< .001	6.41	(6.32,6.51)	0.05	< .001
	9-15 years	5.73	(5.52,5.94)	0.11		7.31	(7.13,7.49)	0.09		6.95	(6.76,7.15)	0.10	
	16+ years	6.20	(5.77,6.63)	0.22		7.51	(7.09,7.93)	0.21		7.53	(7.12,7.94)	0.21	

Variable	Category	Cantril's Ladder				Life Satisfaction				Happiness			
		Mean	95% CI	SE	Global p-value	Mean	95% CI	SE	Global p-value	Mean	95% CI	SE	Global p-value
Immigration status	Born in this country	5.63	(5.54,5.72)	0.05	0.658	7.00	(6.92,7.09)	0.04	0.602	6.49	(6.40,6.58)	0.05	0.003
	Born in another country	5.46	(4.69,6.22)	0.39		6.76	(5.81,7.71)	0.48		5.26	(4.41,6.12)	0.43	
Religious affiliation	Christianity	6.25	(5.80,6.71)	0.23	< .001	7.33	(6.87,7.80)	0.24	< .001	7.33	(6.94,7.71)	0.20	< .001
	Islam	5.65	(5.38,5.92)	0.14		7.04	(6.80,7.28)	0.12		6.27	(6.00,6.54)	0.14	
	Hinduism	5.62	(5.52,5.72)	0.05		6.99	(6.89,7.08)	0.05		6.47	(6.37,6.56)	0.05	
	Buddhism	5.42	(4.80,6.04)	0.31		7.04	(6.46,7.62)	0.29		6.89	(6.33,7.45)	0.29	
	Sikhism	5.13	(4.33,5.92)	0.40		7.05	(6.59,7.51)	0.23		6.65	(6.13,7.17)	0.26	
	Jainism	5.95	*	*		7.15	*	*		6.38	*	*	
	Shinto	10.00	*	*		9.52	*	*		10.00	*	*	
	Primal, Animist, or Folk religion	5.22	(4.45,5.98)	0.36		7.24	(5.84,8.64)	0.67		5.86	(4.85,6.88)	0.48	
	Some other religion	5.34	(4.40,6.28)	0.47		7.20	(6.03,8.36)	0.58		7.40	(6.23,8.57)	0.58	
	No religion/Atheist /Agnostic	4.52	(0.00,10.0ǂ)	1.44		4.97	(0.00,10.0ǂ)	1.34		6.25	(2.00,10.0ǂ)	0.82	
Race/Ethnicity	General	5.67	(5.50,5.83)	0.08	0.005	7.05	(6.90,7.21)	0.08	0.755	6.68	(6.52,6.83)	0.08	0.010
	Other backward caste	5.54	(5.38,5.69)	0.08		7.00	(6.85,7.15)	0.08		6.34	(6.19,6.49)	0.08	
	Schedule caste	5.55	(5.41,5.69)	0.07		6.95	(6.80,7.10)	0.08		6.41	(6.25,6.58)	0.08	
	Schedule tribe	6.05	(5.78,6.32)	0.14		7.03	(6.80,7.25)	0.12		6.53	(6.27,6.80)	0.14	

Table S8c. Childhood predictors regression analysis results for India

		Cantril's Ladder				Life Satisfaction				Happiness			
Variable	Category	Est	95% CI	SE	Global p-value	Est	95% CI	SE	Global p-value	Est	95% CI	SE	Global p-value
Relationship with mother	(Ref: Very bad/somewhat bad)				0.114				0.481				0.726
	Very good/somewhat good	0.46	(-0.11, 1.03)	0.29		0.20	(-0.37, 0.77)	0.29		0.11	(-0.53, 0.76)	0.33	
Relationship with father	(Ref: Very bad/somewhat bad)				0.419				0.605				0.289
	Very good/somewhat good	-0.22	(-0.76, 0.33)	0.28		-0.11	(-0.60, 0.38)	0.25		-0.26	(-0.74, 0.22)	0.25	
Parent marital status	(Ref: Parents married)				<.001				0.001				<.001
	Divorced	-0.14	(-0.76, 0.49)	0.31		0.23	(-0.20, 0.65)	0.22		-0.15	(-0.63, 0.32)	0.24	
	Parents were never married	-0.70	(-0.95, -0.45)	0.13		-0.45	(-0.72, -0.18)	0.13		-0.52	(-0.87, -0.18)	0.17	
	One or both parents had died	-0.54	(-0.81, -0.28)	0.14		-0.03	(-0.29, 0.23)	0.13		-0.36	(-0.68, -0.04)	0.16	
Subjective financial status of family growing up	(Ref: Got by)				<.001				<.001				<.001
	Lived comfortably	0.35	(0.15, 0.55)	0.10		0.22	(0.02, 0.41)	0.10		0.23	(0.02, 0.44)	0.11	
	Found it difficult	-0.24	(-0.47, -0.01)	0.12		-0.28	(-0.50, -0.06)	0.11		-0.36	(-0.60, -0.13)	0.12	
	Found it very difficult	-0.24	(-0.50, 0.02)	0.13		-0.41	(-0.65, -0.18)	0.12		-0.41	(-0.66, -0.15)	0.13	
Abuse	(Ref: No)				0.102				0.011				<.001
	Yes	0.20	(-0.04, 0.44)	0.12		-0.30	(-0.58, -0.02)	0.14		-0.47	(-0.73, -0.21)	0.13	
Outsider growing up	(Ref: No)				0.006				0.172				0.014
	Yes	-0.34	(-0.58, -0.10)	0.12		-0.15	(-0.37, 0.07)	0.11		-0.30	(-0.55, -0.06)	0.12	
Self-rated health growing up	(Ref: Good)				<.001				0.001				<.001
	Excellent	-0.17	(-0.41, 0.07)	0.12		0.03	(-0.21, 0.26)	0.12		0.06	(-0.20, 0.32)	0.13	

		Cantril's Ladder				Life Satisfaction				Happiness			
Variable	Category	Est	95% CI	SE	Global p-value	Est	95% CI	SE	Global p-value	Est	95% CI	SE	Global p-value
	Very good	-0.10	(-0.30, 0.11)	0.10		0.07	(-0.11, 0.25)	0.09		-0.01	(-0.19, 0.18)	0.10	
	Fair	-0.45	(-0.68, -0.22)	0.12		-0.31	(-0.53, -0.08)	0.11		-0.36	(-0.59, -0.13)	0.12	
	Poor	-0.99	(-1.46, -0.51)	0.24		-0.55	(-0.99, -0.11)	0.23		-0.65	(-1.09, -0.22)	0.22	
Immigration status	(Ref: Born in this country)				0.661				0.705				0.022
	Born in another country	-0.16	(-0.89, 0.57)	0.37		-0.15	(-1.04, 0.73)	0.45		-0.97	(-1.82, -0.13)	0.43	
Age 12 religious service attendance	(Ref: Never)				0.649				0.437				0.339
	At least 1/week	0.15	(-0.11, 0.40)	0.13		0.14	(-0.08, 0.37)	0.12		0.22	(-0.04, 0.48)	0.13	
	1-3/month	0.11	(-0.18, 0.39)	0.14		0.17	(-0.09, 0.43)	0.13		0.12	(-0.16, 0.41)	0.15	
	< 1/month	0.06	(-0.21, 0.34)	0.14		0.05	(-0.22, 0.31)	0.14		0.11	(-0.18, 0.40)	0.15	
Year of birth	(Ref: 1998-2005; current age: 18-24)				<.001				<.001				<.001
	1993-1998; age 25-29	-0.42	(-0.69, -0.16)	0.14		-0.35	(-0.60, -0.10)	0.13		-0.52	(-0.77, -0.27)	0.13	
	1983-1993; age 30-39	-0.51	(-0.74, -0.29)	0.12		-0.52	(-0.76, -0.29)	0.12		-0.69	(-0.93, -0.46)	0.12	
	1973-1983; age 40-49	-0.60	(-0.86, -0.34)	0.13		-0.62	(-0.86, -0.38)	0.12		-0.86	(-1.10, -0.61)	0.13	
	1963-1973; age 50-59	-0.61	(-0.91, -0.31)	0.15		-0.76	(-1.04, -0.47)	0.15		-1.04	(-1.33, -0.74)	0.15	
	1953-1963; age 60-69	-0.45	(-0.77, -0.13)	0.16		-0.61	(-0.94, -0.29)	0.17		-1.06	(-1.40, -0.71)	0.18	
	1943-1953; age 70-79	-0.15	(-0.66, 0.36)	0.26		-0.47	(-0.95, 0.01)	0.24		-0.54	(-1.06, -0.03)	0.26	
	1943 or earlier; age 80+	0.31	(-0.85, 1.48)	0.59		0.16	(-0.99, 1.31)	0.59		-0.34	(-1.60, 0.91)	0.64	
Gender	(Ref: Male)				<.001				0.004				0.090
	Female	0.43	(0.27, 0.58)	0.08		0.22	(0.07, 0.37)	0.08		0.13	(-0.02, 0.28)	0.08	
Religious affiliation	(Ref: Hinduism)				0.834				0.308				<.001
	Islam	-0.03	(-0.30, 0.25)	0.14		-0.03	(-0.28, 0.22)	0.13		-0.29	(-0.57, -0.02)	0.14	
	Collapsed affiliations with prevalence<3%	0.09	(-0.25, 0.43)	0.17		0.22	(-0.08, 0.52)	0.15		0.51	(0.22, 0.80)	0.15	

		Cantril's Ladder				Life Satisfaction				Happiness			
Variable	Category	Est	95% CI	SE	Global p-value	Est	95% CI	SE	Global p-value	Est	95% CI	SE	Global p-value
Race/ethnicity	(Ref: Plurality group)				0.130				0.875				0.035
	Non-plurality groups	0.13	(-0.04, 0.30)	0.09		0.01	(-0.16, 0.18)	0.09		0.18	(0.01, 0.36)	0.09	

Table S8d. Sensitivity to unmeasured confounding of childhood predictors in India

Variable	Category	Cantril's Ladder		Life Satisfaction		Happiness	
		E-value for Estimate	E-value for 95% CI	E-value for Estimate	E-value for 95% CI	E-value for Estimate	E-value for 95% CI
Relationship with mother	(Ref: Very bad/somewhat bad)						
	Very good/somewhat good	1.49	1.00	1.29	1.00	1.20	1.00
Relationship with father	(Ref: Very bad/somewhat bad)						
	Very good/somewhat good	1.30	1.00	1.20	1.00	1.34	1.00
Parent marital status	(Ref: Parents married)						
	Divorced	1.23	1.00	1.32	1.00	1.24	1.00
	Parents were never married	1.68	1.49	1.50	1.28	1.55	1.28
	One or both parents had died	1.56	1.35	1.10	1.00	1.42	1.14
Subjective financial status of family growing up	(Ref: Got by)						
	Lived comfortably	1.41	1.24	1.31	1.08	1.31	1.09
	Found it difficult	1.32	1.05	1.36	1.14	1.42	1.22
	Found it very difficult	1.32	1.00	1.47	1.27	1.46	1.24
Abuse	(Ref: No)						
	Yes	1.29	1.00	1.38	1.10	1.51	1.30
Outsider growing up	(Ref: No)						
	Yes	1.40	1.19	1.25	1.00	1.38	1.14
Self-rated health growing up	(Ref: Good)						
	Excellent	1.26	1.00	1.10	1.00	1.14	1.00
	Very good	1.18	1.00	1.16	1.00	1.04	1.00

		Cantril's Ladder		Life Satisfaction		Happiness	
Variable	Category	E-value for Estimate	E-value for 95% CI	E-value for Estimate	E-value for 95% CI	E-value for Estimate	E-value for 95% CI
	Fair	1.49	1.30	1.38	1.17	1.42	1.22
	Poor	1.89	1.54	1.58	1.20	1.65	1.31
Immigration status	(Ref: Born in this country)						
	Born in another country	1.25	1.00	1.25	1.00	1.88	1.22
Age 12 religious service attendance	(Ref: Never)						
	At least 1/week	1.24	1.00	1.24	1.00	1.30	1.00
	1-3/month	1.20	1.00	1.27	1.00	1.22	1.00
	< 1/month	1.15	1.00	1.12	1.00	1.20	1.00
Year of birth	(Ref: 1998-2005; current age: 18-24)						
	1993-1998; age 25-29	1.47	1.25	1.42	1.20	1.54	1.35
	1983-1993; age 30-39	1.54	1.36	1.56	1.38	1.68	1.50
	1973-1983; age 40-49	1.60	1.41	1.63	1.44	1.80	1.61
	1963-1973; age 50-59	1.61	1.38	1.74	1.52	1.93	1.71
	1953-1963; age 60-69	1.49	1.23	1.63	1.37	1.95	1.69
	1943-1953; age 70-79	1.24	1.00	1.51	1.00	1.56	1.09
	1943 or earlier; age 80+	1.38	1.00	1.26	1.00	1.41	1.00
Gender	(Ref: Male)						
	Female	1.47	1.34	1.31	1.16	1.22	1.00
Religious affiliation	(Ref: Hinduism)						
	Islam	1.09	1.00	1.09	1.00	1.37	1.08
	Collapsed affiliations with prevalence<3%	1.18	1.00	1.31	1.00	1.54	1.31
Race/ethnicity	(Ref: Plurality group)						
	Non-plurality groups	1.22	1.00	1.06	1.00	1.27	1.06

Table S8e. Complete-case supplemental analysis of means by demographic category for India

		Cantril's Ladder				Life Satisfaction				Happiness			
Variable	Category	Mean	95% CI	SE	Global p-value	Mean	95% CI	SE	Global p-value	Mean	95% CI	SE	Global p-value
Age group	18-24	6.94	(6.75,7.13)	0.10	< .001	8.24	(8.10,8.38)	0.07	< .001	7.91	(7.75,8.06)	0.08	< .001
	25-29	6.56	(6.37,6.74)	0.10		7.94	(7.79,8.10)	0.08		7.57	(7.41,7.72)	0.08	
	30-39	6.51	(6.38,6.64)	0.07		7.72	(7.60,7.84)	0.06		7.35	(7.23,7.47)	0.06	
	40-49	6.41	(6.25,6.56)	0.08		7.58	(7.45,7.72)	0.07		7.20	(7.06,7.34)	0.07	
	50-59	6.41	(6.22,6.59)	0.09		7.44	(7.26,7.62)	0.09		7.02	(6.84,7.20)	0.09	
	60-69	6.49	(6.24,6.74)	0.13		7.56	(7.32,7.80)	0.12		7.14	(6.91,7.38)	0.12	
	70-79	6.71	(6.31,7.12)	0.21		7.85	(7.50,8.21)	0.18		7.53	(7.15,7.91)	0.19	
	80 or older	7.29	(6.28,8.30)	0.50		8.44	(7.56,9.33)	0.44		7.94	(7.00,8.88)	0.47	
Gender	Female	6.79	(6.68,6.90)	0.06	< .001	7.90	(7.81,8.00)	0.05	< .001	7.58	(7.48,7.68)	0.05	< .001
	Male	6.38	(6.27,6.49)	0.05		7.67	(7.58,7.77)	0.05		7.26	(7.17,7.36)	0.05	
Marital status	Divorced	4.18	(2.44,5.93)	0.82	< .001	7.73	(5.94,9.52)	0.85	< .001	5.76	(4.14,7.37)	0.76	< .001
	Domestic partner	5.97	(5.51,6.42)	0.23		7.56	(6.92,8.19)	0.32		7.07	(6.67,7.48)	0.21	
	Married	6.60	(6.51,6.69)	0.05		7.78	(7.70,7.85)	0.04		7.40	(7.32,7.48)	0.04	
	Separated	5.71	(4.21,7.21)	0.74		6.97	(5.62,8.32)	0.67		7.05	(5.90,8.20)	0.57	
	Single/Never been married	6.72	(6.55,6.89)	0.09		8.04	(7.89,8.19)	0.08		7.67	(7.51,7.82)	0.08	
	Widowed	6.01	(5.63,6.40)	0.20		7.06	(6.68,7.43)	0.19		6.76	(6.37,7.14)	0.19	
Employment	Employed for an employer	6.23	(6.07,6.40)	0.09	< .001	7.55	(7.40,7.71)	0.08	0.006	7.19	(7.04,7.33)	0.08	< .001
	Homemaker	6.79	(6.65,6.92)	0.07		7.87	(7.76,7.97)	0.06		7.52	(7.41,7.63)	0.06	
	None of these/Other	6.63	(6.30,6.97)	0.17		7.78	(7.51,8.06)	0.14		7.44	(7.12,7.75)	0.16	
	Retired	6.46	(5.98,6.94)	0.24		7.63	(7.18,8.09)	0.23		7.39	(6.98,7.80)	0.21	
	Self-employed	6.59	(6.45,6.72)	0.07		7.85	(7.72,7.98)	0.07		7.41	(7.27,7.55)	0.07	
	Student	6.93	(6.64,7.22)	0.15		8.06	(7.83,8.29)	0.12		7.79	(7.57,8.02)	0.12	
	Unemployed and looking for a job	6.41	(6.14,6.69)	0.14		7.76	(7.51,8.01)	0.13		7.42	(7.19,7.65)	0.12	
Religious service attendance	A few times a year	6.38	(6.23,6.52)	0.07	0.015	7.66	(7.52,7.80)	0.07	0.008	7.24	(7.09,7.40)	0.08	0.012
	More than once a week	6.57	(6.41,6.72)	0.08		7.83	(7.69,7.97)	0.07		7.39	(7.25,7.52)	0.07	
	Never	6.63	(6.43,6.84)	0.10		7.63	(7.45,7.81)	0.09		7.34	(7.17,7.51)	0.09	
	Once a week	6.71	(6.57,6.86)	0.07		7.96	(7.84,8.08)	0.06		7.57	(7.44,7.70)	0.07	
	One to three times a month	6.57	(6.42,6.73)	0.08		7.74	(7.61,7.88)	0.07		7.45	(7.31,7.59)	0.07	

		Cantril's Ladder				Life Satisfaction				Happiness			
Variable	Category	Mean	95% CI	SE	Global p-value	Mean	95% CI	SE	Global p-value	Mean	95% CI	SE	Global p-value
Education	Up to 8 years	6.60	(6.52,6.69)	0.05	0.125	7.78	(7.70,7.86)	0.04	0.500	7.40	(7.33,7.48)	0.04	0.057
	16+ years	6.43	(6.01,6.86)	0.21		7.95	(7.61,8.29)	0.17		7.87	(7.49,8.25)	0.19	
	9 to 15 years	6.41	(6.23,6.59)	0.09		7.85	(7.69,8.01)	0.08		7.47	(7.29,7.65)	0.09	
Immigration status	Born in another country	6.19	(5.57,6.81)	0.31	0.208	7.00	(6.04,7.96)	0.49	0.103	6.26	(5.67,6.86)	0.30	< .001
	Born in this country	6.59	(6.50,6.67)	0.04		7.80	(7.72,7.87)	0.04		7.43	(7.35,7.50)	0.04	
Religious affiliation	Buddhism	6.53	(6.07,6.99)	0.23	< .001	7.93	(7.53,8.33)	0.20	< .001	7.56	(7.14,7.97)	0.21	< .001
	Christianity	7.03	(6.55,7.51)	0.24		8.20	(7.85,8.56)	0.18		7.92	(7.54,8.30)	0.19	
	Hinduism	6.58	(6.49,6.67)	0.05		7.78	(7.70,7.85)	0.04		7.41	(7.33,7.49)	0.04	
	Islam	6.58	(6.32,6.84)	0.13		7.83	(7.60,8.06)	0.12		7.37	(7.14,7.60)	0.12	
	No religion/Atheist/Agnostic	4.78	(0.00,12.69)	1.53		6.60	(1.56,11.65)	0.97		6.91	(2.82,11.00)	0.79	
	Primal, Animist, or Folk religion	6.29	(5.26,7.32)	0.49		7.36	(6.06,8.65)	0.61		6.09	(5.16,7.02)	0.44	
	Sikhism	6.43	(5.96,6.89)	0.23		7.43	(6.89,7.98)	0.28		7.28	(6.69,7.87)	0.30	
	Some other religion	6.17	(5.20,7.14)	0.48		7.66	(6.69,8.63)	0.48		7.78	(7.12,8.44)	0.33	
	Jainism	6.29	(0.00,5.00)	0.97		7.23	(0.00,4.00)	0.84		6.90	(0.00,5.00)	0.99	
	Shinto	10.00	*	*		10.00	*	*		10.00	*	*	

Table S8f. Complete-case supplemental analysis of childhood predictors regression analysis results for India

		Cantril's Ladder				Life Satisfaction				Happiness			
Variable	Category	Est	95% CI	SE	Global p-value	Est	95% CI	SE	Global p-value	Est	95% CI	SE	Global p-value
Relationship with mother	(Ref: Very bad/somewhat bad)				0.541				0.297				0.741
	Very good/somewhat good	-0.15	(-0.63,0.33)	0.25		0.22	(-0.19,0.63)	0.21		-0.07	(-0.51,0.36)	0.22	
Relationship with father	(Ref: Very bad/somewhat bad)				0.873				0.509				0.716
	Very good/somewhat good	0.04	(-0.41,0.48)	0.23		0.12	(-0.24,0.49)	0.19		0.07	(-0.32,0.47)	0.20	
Parent marital status	(Ref: Parents married)				0.002				<.001				<.001
	No, one or both of them had died	0.16	(-0.34,0.66)	0.26		0.21	(-0.19,0.61)	0.20		0.05	(-0.33,0.44)	0.20	
	No, they were never married	-0.21	(-0.69,0.27)	0.25		-0.28	(-0.67,0.11)	0.20		-0.40	(-0.78,-0.02)	0.19	
	Yes, married	-0.26	(-0.71,0.19)	0.23		-0.36	(-0.72,-0.00)	0.18		-0.35	(-0.71,0.00)	0.18	
Subjective financial status of family growing up	(Ref: Got by)				<.001				<.001				<.001
	Found it difficult	-0.17	(-0.37,0.03)	0.10		-0.19	(-0.37,-0.01)	0.09		-0.27	(-0.45,-0.08)	0.09	
	Found it very difficult	-0.27	(-0.52,-0.02)	0.13		-0.46	(-0.66,-0.27)	0.10		-0.47	(-0.67,-0.27)	0.10	
	Lived comfortably	0.22	(0.04,0.41)	0.09		0.03	(-0.11,0.18)	0.08		0.04	(-0.12,0.20)	0.08	
Abuse	(Ref: No)				<.001				0.800				0.219
	Yes	0.50	(0.30,0.70)	0.10		0.02	(-0.16,0.21)	0.09		0.12	(-0.07,0.30)	0.10	
Outsider growing up	(Ref: No)				0.592				0.432				0.519
	Yes	0.06	(-0.16,0.27)	0.11		0.07	(-0.10,0.24)	0.09		0.06	(-0.12,0.24)	0.09	

		Cantril's Ladder				Life Satisfaction				Happiness			
Variable	Category	Est	95% CI	SE	Global p-value	Est	95% CI	SE	Global p-value	Est	95% CI	SE	Global p-value
Self-rated health growing up	(Ref: Good)				<.001				0.016				<.001
	Excellent	-0.34	(-0.54,-0.14)	0.10		-0.10	(-0.30,0.10)	0.10		-0.07	(-0.27,0.13)	0.10	
	Fair	-0.34	(-0.53,-0.14)	0.10		-0.21	(-0.38,-0.03)	0.09		-0.30	(-0.48,-0.13)	0.09	
	Poor	-0.61	(-1.02,-0.21)	0.21		-0.30	(-0.68,0.07)	0.19		-0.47	(-0.83,-0.10)	0.19	
	Very good	-0.14	(-0.32,0.04)	0.09		0.06	(-0.09,0.20)	0.07		0.04	(-0.12,0.19)	0.08	
Immigration status	(Ref: Born in this country)				0.356				0.189				<.001
	Born in another country	-0.27	(-0.83,0.30)	0.29		-0.59	(-1.47,0.29)	0.45		-0.90	(-1.43,-0.37)	0.27	
Age 12 religious service attendance	(Ref: Never)				0.757				0.488				0.582
	At least once a week	0.06	(-0.16,0.28)	0.11		0.13	(-0.06,0.32)	0.10		0.05	(-0.15,0.26)	0.10	
	Less than once a month	0.08	(-0.17,0.32)	0.13		0.11	(-0.11,0.33)	0.11		-0.06	(-0.28,0.17)	0.12	
	One to three times a month	0.14	(-0.12,0.39)	0.13		0.16	(-0.05,0.38)	0.11		-0.03	(-0.25,0.18)	0.11	
Year of birth	(Ref: 1998-2005; current age: 18-24)				0.003				<.001				<.001
	1993-1998; age 25-29	-0.35	(-0.60,-0.10)	0.13		-0.29	(-0.49,-0.10)	0.10		-0.33	(-0.53,-0.13)	0.10	
	1983-1993; age 30-39	-0.39	(-0.60,-0.18)	0.11		-0.51	(-0.68,-0.33)	0.09		-0.53	(-0.72,-0.34)	0.10	
	1973-1983; age 40-49	-0.48	(-0.72,-0.24)	0.12		-0.63	(-0.81,-0.45)	0.09		-0.67	(-0.87,-0.47)	0.10	
	1963-1973; age 50-59	-0.47	(-0.73,-0.21)	0.13		-0.76	(-0.99,-0.53)	0.12		-0.84	(-1.07,-0.61)	0.12	
	1953-1963; age 60-69	-0.40	(-0.70,-0.09)	0.15		-0.64	(-0.91,-0.37)	0.14		-0.71	(-0.99,-0.43)	0.14	
	1943-1953; age 70-79	-0.14	(-0.60,0.32)	0.23		-0.36	(-0.74,0.01)	0.19		-0.35	(-0.74,0.04)	0.20	

		Cantril’s Ladder				Life Satisfaction				Happiness			
Variable	Category	Est	95% CI	SE	Global p-value	Est	95% CI	SE	Global p-value	Est	95% CI	SE	Global p-value
	1943 or earlier; age 80+	0.25	(-0.74,1.24)	0.51		0.12	(-0.74,0.98)	0.44		-0.07	(-0.98,0.84)	0.46	
Gender	(Ref: Male)				<.001				<.001				<.001
	Male	-0.41	(-0.55,-0.27)	0.07		-0.21	(-0.33,-0.10)	0.06		-0.30	(-0.42,-0.17)	0.06	
Religious affiliation	(Ref: Hinduism)				0.875				0.714				0.220
	Islam	0.04	(-0.21,0.30)	0.13		0.01	(-0.22,0.24)	0.12		-0.11	(-0.34,0.13)	0.12	
	Collapsed affiliations with prevalence<3%	0.07	(-0.24,0.38)	0.16		0.10	(-0.14,0.35)	0.13		0.17	(-0.08,0.41)	0.13	
Race/ethnicity	(Ref: Plurality group)				0.231				0.778				0.100
	Non-plurality groups	0.09	(-0.06,0.25)	0.08		0.02	(-0.11,0.15)	0.07		0.11	(-0.02,0.25)	0.07	

Table S8g. Complete-case supplemental analysis of sensitivity to unmeasured confounding of childhood predictors in India

		Cantril's Ladder		Life Satisfaction		Happiness	
Variable	Category	E-value for Estimate	E-value for 95% CI	E-value for Estimate	E-value for 95% CI	E-value for Estimate	E-value for 95% CI
Relationship with mother	(Ref: Very bad/somewhat bad)						
	Very good/somewhat good	1.27	1.00	1.36	1.00	1.18	1.00
Relationship with father	(Ref: Very bad/somewhat bad)						
	Very good/somewhat good	1.12	1.00	1.25	1.00	1.18	1.00
Parent marital status	(Ref: Parents married)						
	No, one or both of them had died	1.28	1.00	1.35	1.00	1.15	1.00
	No, they were never married	1.34	1.00	1.43	1.00	1.54	1.10
	Yes, married	1.38	1.00	1.51	1.02	1.50	1.00
Subjective financial status of family growing up	(Ref: Got by)						
	Found it difficult	1.29	1.00	1.33	1.06	1.41	1.20
	Found it very difficult	1.39	1.08	1.61	1.42	1.61	1.42
	Lived comfortably	1.35	1.13	1.12	1.00	1.13	1.00
Abuse	(Ref: No)						
	Yes	1.61	1.42	1.10	1.00	1.24	1.00
Outsider growing up	(Ref: No)						
	Yes	1.15	1.00	1.18	1.00	1.16	1.00
Self-rated health growing up	(Ref: Good)						
	Excellent	1.46	1.25	1.22	1.00	1.18	1.00
	Fair	1.46	1.25	1.35	1.11	1.45	1.26
	Poor	1.71	1.33	1.45	1.00	1.61	1.22
	Very good	1.25	1.00	1.16	1.00	1.12	1.00
Immigration status	(Ref: Born in this country)						
	Born in another country	1.39	1.00	1.74	1.00	2.03	1.51
Age 12 religious service attendance	(Ref: Never)						
	At least once a week	1.16	1.00	1.26	1.00	1.15	1.00
	Less than once a month	1.18	1.00	1.24	1.00	1.16	1.00
	One to three times a month	1.25	1.00	1.30	1.00	1.12	1.00
Year of birth	(Ref: 1998-2005; current age: 18-24)						

Variable	Category	Cantril's Ladder		Life Satisfaction		Happiness	
		E-value for Estimate	E-value for 95% CI	E-value for Estimate	E-value for 95% CI	E-value for Estimate	E-value for 95% CI
	1993-1998; age 25-29	1.47	1.22	1.44	1.22	1.47	1.25
	1983-1993; age 30-39	1.51	1.31	1.66	1.48	1.67	1.49
	1973-1983; age 40-49	1.59	1.36	1.78	1.60	1.80	1.61
	1963-1973; age 50-59	1.58	1.33	1.91	1.68	1.97	1.74
	1953-1963; age 60-69	1.51	1.20	1.79	1.52	1.85	1.58
	1943-1953; age 70-79	1.26	1.00	1.52	1.00	1.49	1.00
	1943 or earlier; age 80+	1.38	1.00	1.25	1.00	1.17	1.00
Gender	(Ref: Male)						
	Male	1.52	1.39	1.36	1.22	1.44	1.31
Religious affiliation	(Ref: Hinduism)						
	Islam	1.13	1.00	1.05	1.00	1.23	1.00
	Collapsed affiliations with prevalence<3%	1.17	1.00	1.23	1.00	1.30	1.00
Race/ethnicity	(Ref: Plurality group)						
	Non-plurality groups	1.20	1.00	1.09	1.00	1.24	1.00

Tables S9a-g: Indonesia

Table S9a. Nationally representative descriptive statistics for Indonesia

Characteristic	N = 6,992[1]
**Age group**	
1998-2005; age 18-24	1,216 (17%)
1993-1998; age 25-29	849 (12%)
1983-1993; age 30-39	1,591 (23%)
1973-1983; age 40-49	1,576 (23%)
1963-1973; age 50-59	1,169 (17%)
1953-1963; age 60-69	490 (7.0%)
1943-1953; age 70-79	83 (1.2%)
1943 or earlier; age 80+	17 (0.2%)
(Missing)	0 (0%)
**Gender**	
Male	3,461 (50%)
Female	3,513 (50%)
Other	7 (<0.1%)
(Missing)	11 (0.2%)
**Race/Ethnicity**	
Bali	69 (1.0%)
Banjar/Melayu Banjar	320 (4.6%)
Batak	165 (2.4%)
Betawi	251 (3.6%)
Bugis	243 (3.5%)
Jawa	2,846 (41%)
Madura	262 (3.7%)
Makasar	91 (1.3%)
Minangkabau	273 (3.9%)
Other	1,262 (18%)
Sunda/Parahyangan	1,172 (17%)
(Missing)	38 (0.5%)
**Respondent Marital status**	
Married	4,846 (69%)
Separated	81 (1.2%)
Divorced	196 (2.8%)
Widowed	425 (6.1%)
Single, never married	1,381 (20%)
Domestic Partner	18 (0.3%)
(Missing)	45 (0.6%)
**Employment**	
Employed for an employer	1,323 (19%)
Self-employed	2,187 (31%)
Retired	78 (1.1%)
Student	272 (3.9%)
Homemaker	2,138 (31%)
Unemployed and looking for a job	529 (7.6%)
None of these/Other	448 (6.4%)
(Missing)	18 (0.3%)
**Religious service attendance as an adult (now)**	
More than 1/week	2,667 (38%)
1/week	2,529 (36%)
1-3/month	786 (11%)
A few times a year	659 (9.4%)
Never	332 (4.8%)
(Missing)	18 (0.3%)
**Education (years)**	
Up to 8 years	3,079 (44%)
9-15 years	3,491 (50%)
16+ years	419 (6.0%)
(Missing)	2 (<0.1%)
**Immigration status**	
Born in this country	6,958 (100%)
Born in another country	34 (0.5%)
(Missing)	0 (0%)

Characteristic	N = 6,992[1]
**Religious affiliation as an adult (now)**	
Christianity	504 (7.2%)
Islam	6,406 (92%)
Hinduism	73 (1.0%)
Buddhism	3 (<0.1%)
Judaism	0 (0%)
Sikhism	0 (0%)
Baha'i	0 (0%)
Jainism	0 (0%)
Shinto	0 (0%)
Taoism	1 (<0.1%)
Confucianism	0 (0%)
Primal, Animist, or Folk religion	0 (0%)
Spiritism	0 (0%)
Umbanda, Candomble, and other African-derived religions	0 (0%)
Chinese folk/traditional religion	0 (0%)
Some other religion	1 (<0.1%)
No religion/Atheist/Agnostic	0 (0%)
(Missing)	4 (<0.1%)
**Relationship with mother growing up**	
Very good	6,238 (89%)
Somewhat good	583 (8.3%)
Somewhat bad	50 (0.7%)
Very bad	26 (0.4%)
Does not apply	68 (1.0%)
(Missing)	27 (0.4%)
**Relationship with father growing up**	
Very good	6,067 (87%)
Somewhat good	628 (9.0%)
Somewhat bad	68 (1.0%)
Very bad	52 (0.7%)
Does not apply	115 (1.6%)
(Missing)	61 (0.9%)
**Parent marital status at age 12**	
Parents married	5,557 (79%)
Divorced	448 (6.4%)
Parents were never married	47 (0.7%)
One or both parents had died	735 (11%)
(Missing)	205 (2.9%)
**Subjective financial status of family growing up**	
Lived comfortably	3,408 (49%)
Got by	2,955 (42%)
Found it difficult	439 (6.3%)
Found it very difficult	181 (2.6%)
(Missing)	9 (0.1%)
**Abuse**	
Yes	486 (6.9%)
No	6,427 (92%)
(Missing)	79 (1.1%)
**Outsider growing up**	
Yes	343 (4.9%)
No	6,639 (95%)
(Missing)	10 (0.1%)
**Self-rated health growing up**	
Excellent	1,246 (18%)
Very good	1,968 (28%)
Good	2,490 (36%)
Fair	1,233 (18%)
Poor	55 (0.8%)
(Missing)	1 (<0.1%)
**Age 12 religious service attendance**	
At least 1/week	5,363 (77%)
1-3/month	973 (14%)
<1/month	329 (4.7%)
Never	275 (3.9%)

Characteristic	N = 6,992[1]
(Missing)	51 (0.7%)
**Religious affiliation at age 12**	
Christianity	528 (7.6%)
Islam	6,373 (91%)
Hinduism	75 (1.1%)
Buddhism	5 (<0.1%)
Judaism	0 (0%)
Sikhism	0 (0%)
Baha'i	0 (0%)
Jainism	1 (<0.1%)
Shinto	0 (0%)
Taoism	0 (<0.1%)
Confucianism	1 (<0.1%)
Primal, Animist, or Folk religion	1 (<0.1%)
Spiritism	0 (0%)
Umbanda, Candomble, and other African-derived religions	0 (0%)
Chinese folk/traditional religion	0 (0%)
Some other religion	0 (0%)
No religion/Atheist/Agnostic	2 (<0.1%)
(Missing)	8 (0.1%)

[1]n (%)

Table S9b. Means by demographic category for Indonesia

Variable	Category	Cantril's Ladder				Life Satisfaction				Happiness			
		Mean	95% CI	SE	Global p-value	Mean	95% CI	SE	Global p-value	Mean	95% CI	SE	Global p-value
Age group	18-24	7.06	(6.90,7.23)	0.08	0.137	7.85	(7.69,8.01)	0.08	0.022	8.02	(7.87,8.16)	0.07	0.483
	25-29	7.11	(6.92,7.30)	0.10		8.17	(8.01,8.33)	0.08		8.17	(8.00,8.34)	0.09	
	30-39	6.96	(6.80,7.12)	0.08		8.10	(7.95,8.26)	0.08		8.12	(7.98,8.26)	0.07	
	40-49	6.78	(6.60,6.95)	0.09		7.98	(7.82,8.14)	0.08		8.06	(7.90,8.21)	0.08	
	50-59	6.97	(6.73,7.21)	0.12		7.89	(7.71,8.08)	0.09		7.89	(7.71,8.08)	0.09	
	60-69	7.14	(6.78,7.50)	0.18		8.04	(7.74,8.35)	0.16		7.99	(7.63,8.35)	0.18	
	70-79	6.98	(6.01,7.94)	0.48		7.73	(7.09,8.38)	0.32		7.82	(7.03,8.62)	0.40	
	80 or older	5.84	(3.51,8.18)	0.98		5.74	(2.93,8.54)	1.18		7.41	(5.72,9.10)	0.71	
Gender	Male	6.75	(6.61,6.88)	0.07	< .001	7.89	(7.77,8.02)	0.06	< .001	7.93	(7.82,8.04)	0.06	0.001
	Female	7.19	(7.08,7.31)	0.06		8.09	(8.00,8.18)	0.05		8.16	(8.07,8.25)	0.05	
	Other	3.43	(0.00,8.03)	1.25		6.06	(3.58,8.53)	0.65		4.84	(0.00,10.0ǂ)	1.88	
Marital status	Married	7.00	(6.89,7.11)	0.06	0.500	8.09	(8.00,8.18)	0.05	< .001	8.14	(8.06,8.22)	0.04	< .001
	Separated	6.24	(5.27,7.20)	0.48		7.35	(6.47,8.24)	0.45		7.20	(6.30,8.10)	0.45	
	Divorced	6.60	(6.06,7.13)	0.27		7.43	(6.87,8.00)	0.29		7.16	(6.56,7.75)	0.30	
	Widowed	7.00	(6.67,7.32)	0.17		7.94	(7.65,8.23)	0.15		8.02	(7.75,8.28)	0.14	
	Never	6.95	(6.79,7.11)	0.08		7.78	(7.63,7.94)	0.08		7.88	(7.74,8.03)	0.08	
	Domestic Partner	7.05	(5.74,8.36)	0.58		7.56	(6.24,8.89)	0.59		8.28	(7.31,9.24)	0.43	
Employment	Employed for an employer	6.89	(6.71,7.08)	0.10	0.002	7.82	(7.63,8.01)	0.10	< .001	7.92	(7.75,8.09)	0.09	< .001
	Self-employed	7.00	(6.86,7.14)	0.07		8.09	(7.97,8.22)	0.06		8.08	(7.96,8.21)	0.06	
	Retired	7.39	(6.93,7.85)	0.23		8.45	(8.07,8.82)	0.19		7.91	(7.47,8.36)	0.22	
	Student	6.92	(6.56,7.28)	0.18		7.72	(7.45,8.00)	0.14		7.74	(7.46,8.02)	0.14	
	Homemaker	7.13	(6.98,7.27)	0.07		8.17	(8.05,8.28)	0.06		8.28	(8.16,8.40)	0.06	
	Unemployed and looking for a job	6.41	(6.07,6.75)	0.17		7.46	(7.15,7.78)	0.16		7.51	(7.17,7.85)	0.17	
	None of these/Other	6.89	(6.52,7.26)	0.19		7.87	(7.56,8.18)	0.16		7.91	(7.66,8.17)	0.13	
Religious service attendance	More than 1/week	7.05	(6.90,7.20)	0.07	0.284	8.10	(7.98,8.21)	0.06	0.002	8.13	(8.00,8.25)	0.06	0.134
	1/week	6.91	(6.77,7.04)	0.07		8.03	(7.90,8.16)	0.07		8.04	(7.91,8.17)	0.07	
	1-3/month	7.05	(6.85,7.26)	0.11		7.82	(7.63,8.01)	0.10		7.96	(7.76,8.16)	0.10	
	A few times a year	6.84	(6.64,7.04)	0.10		7.64	(7.42,7.86)	0.11		7.85	(7.68,8.03)	0.09	
	Never	6.84	(6.47,7.22)	0.19		7.96	(7.63,8.29)	0.17		7.93	(7.58,8.28)	0.18	
Education	Up to 8 years	6.94	(6.77,7.11)	0.09	0.762	8.06	(7.92,8.20)	0.07	0.289	8.12	(7.98,8.25)	0.07	0.168
	9-15 years	6.99	(6.88,7.09)	0.05		7.93	(7.85,8.02)	0.04		7.99	(7.91,8.06)	0.04	

Variable	Category	Cantril's Ladder				Life Satisfaction				Happiness			
		Mean	95% CI	SE	Global p-value	Mean	95% CI	SE	Global p-value	Mean	95% CI	SE	Global p-value
	16+ years	7.03	(6.85,7.21)	0.09		7.98	(7.82,8.14)	0.08		7.93	(7.76,8.10)	0.09	
Immigration status	Born in this country	6.97	(6.88,7.06)	0.05	0.403	7.99	(7.91,8.07)	0.04	0.157	8.04	(7.97,8.12)	0.04	0.656
	Born in another country	6.56	(5.55,7.56)	0.49		7.38	(6.46,8.30)	0.45		7.90	(6.89,8.91)	0.49	
Religious affiliation	Christianity	7.15	(6.73,7.58)	0.22	< .001	7.77	(7.45,8.09)	0.16	< .001	8.06	(7.76,8.37)	0.16	< .001
	Islam	6.97	(6.87,7.06)	0.05		8.02	(7.93,8.10)	0.04		8.04	(7.97,8.12)	0.04	
	Hinduism	5.87	(4.54,7.20)	0.67		7.08	(6.01,8.16)	0.54		7.89	(7.37,8.41)	0.26	
	Buddhism	5.00	*	*		5.00	*	*		6.50	*	*	
	Taoism	5.00	*	*		9.00	*	*		6.00	*	*	
	Some other religion	7.00	*	*		7.00	*	*		7.00	*	*	
Race/Ethnicity	Banjar/Melayu Banjar	6.90	(6.40,7.40)	0.25	< .001	8.00	(7.63,8.37)	0.19	0.110	8.21	(7.91,8.51)	0.15	0.017
	Betawi	7.35	(6.98,7.73)	0.19		8.21	(7.92,8.50)	0.15		8.16	(7.83,8.49)	0.17	
	Bugis	7.42	(6.98,7.87)	0.23		8.21	(7.79,8.62)	0.21		8.37	(8.03,8.71)	0.17	
	Jawa	6.87	(6.72,7.01)	0.07		7.97	(7.84,8.09)	0.06		7.92	(7.80,8.03)	0.06	
	Madura	7.16	(6.72,7.59)	0.22		8.22	(7.79,8.64)	0.22		8.05	(7.72,8.39)	0.17	
	Minangkabau	6.80	(6.30,7.29)	0.25		7.62	(7.16,8.07)	0.23		8.05	(7.61,8.49)	0.22	
	Sunda/Parahyangan	6.92	(6.68,7.16)	0.12		7.93	(7.74,8.13)	0.10		8.01	(7.81,8.21)	0.10	
	Bali	5.84	(4.42,7.26)	0.71		7.08	(5.96,8.20)	0.56		7.93	(7.44,8.43)	0.25	
	Batak	6.91	(6.52,7.29)	0.19		7.84	(7.55,8.13)	0.15		7.72	(7.31,8.13)	0.21	
	Makasar	7.74	(7.40,8.08)	0.17		8.38	(7.93,8.83)	0.23		8.58	(8.14,9.02)	0.22	
	Other	7.11	(6.88,7.33)	0.11		8.08	(7.89,8.28)	0.10		8.23	(8.07,8.40)	0.08	

Table S9c. Childhood predictors regression analysis results for Indonesia

		Cantril's Ladder				Life Satisfaction				Happiness			
Variable	Category	Est	95% CI	SE	Global p-value	Est	95% CI	SE	Global p-value	Est	95% CI	SE	Global p-value
Relationship with mother	(Ref: Very bad/somewhat bad)				0.163				0.060				0.023
	Very good/somewhat good	0.42	(-0.19, 1.03)	0.31		0.74	(-0.03, 1.51)	0.39		0.87	(0.10, 1.64)	0.39	
Relationship with father	(Ref: Very bad/somewhat bad)				0.928				0.822				0.788
	Very good/somewhat good	0.01	(-0.56, 0.57)	0.29		0.05	(-0.43, 0.54)	0.25		-0.05	(-0.54, 0.44)	0.25	
Parent marital status	(Ref: Parents married)				0.213				0.484				0.941
	Divorced	-0.30	(-0.64, 0.04)	0.17		-0.11	(-0.46, 0.25)	0.18		0.04	(-0.28, 0.37)	0.17	
	Parents were never married	-0.15	(-1.12, 0.81)	0.49		0.50	(-0.45, 1.46)	0.48		0.08	(-0.70, 0.86)	0.40	
	One or both parents had died	-0.22	(-0.54, 0.10)	0.16		0.08	(-0.18, 0.34)	0.13		0.07	(-0.21, 0.34)	0.14	
Subjective financial status of family growing up	(Ref: Got by)				<.001				<.001				<.001
	Lived comfortably	0.50	(0.35, 0.65)	0.08		0.44	(0.30, 0.58)	0.07		0.35	(0.21, 0.49)	0.07	
	Found it difficult	-0.45	(-0.82, -0.08)	0.19		-0.19	(-0.56, 0.18)	0.19		-0.44	(-0.80, -0.08)	0.18	
	Found it very difficult	-0.60	(-1.25, 0.04)	0.33		-0.30	(-0.90, 0.31)	0.31		-0.58	(-1.17, 0.01)	0.30	
Abuse	(Ref: No)				<.001				0.001				<.001
	Yes	-0.60	(-0.96, -0.25)	0.18		-0.64	(-1.02, -0.25)	0.20		-0.64	(-1.01, -0.28)	0.19	
Outsider growing up	(Ref: No)				0.848				0.153				0.049
	Yes	-0.03	(-0.45, 0.39)	0.22		-0.32	(-0.75, 0.12)	0.22		-0.41	(-0.83, -0.00)	0.21	
Self-rated health growing up	(Ref: Good)				0.095				0.002				<.001
	Excellent	0.18	(-0.07, 0.44)	0.13		0.27	(0.06, 0.49)	0.11		0.33	(0.16, 0.51)	0.09	

		Cantril's Ladder				Life Satisfaction				Happiness			
Variable	Category	Est	95% CI	SE	Global p-value	Est	95% CI	SE	Global p-value	Est	95% CI	SE	Global p-value
	Very good	-0.02	(-0.21, 0.17)	0.10		0.06	(-0.09, 0.22)	0.08		0.12	(-0.04, 0.28)	0.08	
	Fair	-0.18	(-0.40, 0.04)	0.11		-0.23	(-0.43, -0.03)	0.10		-0.15	(-0.34, 0.05)	0.10	
	Poor	-0.19	(-1.37, 1.00)	0.60		-0.49	(-1.76, 0.78)	0.65		-0.28	(-1.43, 0.86)	0.58	
Immigration status	(Ref: Born in this country)				0.461				0.238				0.867
	Born in another country	-0.39	(-1.42, 0.64)	0.53		-0.55	(-1.57, 0.48)	0.52		-0.06	(-0.95, 0.82)	0.45	
Age 12 religious service attendance	(Ref: Never)				0.591				0.297				0.154
	At least 1/week	-0.24	(-0.59, 0.12)	0.18		-0.19	(-0.50, 0.12)	0.16		0.47	(-0.03, 0.96)	0.25	
	1-3/month	-0.20	(-0.62, 0.22)	0.21		-0.33	(-0.68, 0.03)	0.18		0.53	(-0.03, 1.10)	0.29	
	< 1/month	-0.21	(-0.66, 0.24)	0.23		-0.17	(-0.65, 0.30)	0.24		0.27	(-0.28, 0.83)	0.28	
Year of birth	(Ref: 1998-2005; current age: 18-24)				0.231				0.014				0.701
	1993-1998; age 25-29	0.05	(-0.19, 0.30)	0.12		0.32	(0.12, 0.53)	0.10		0.16	(-0.06, 0.38)	0.11	
	1983-1993; age 30-39	-0.01	(-0.21, 0.19)	0.10		0.33	(0.12, 0.54)	0.11		0.18	(-0.02, 0.37)	0.10	
	1973-1983; age 40-49	-0.14	(-0.36, 0.08)	0.11		0.24	(0.01, 0.46)	0.11		0.16	(-0.05, 0.36)	0.10	
	1963-1973; age 50-59	0.14	(-0.14, 0.43)	0.15		0.21	(-0.03, 0.45)	0.12		0.07	(-0.17, 0.31)	0.12	
	1953-1963; age 60-69	0.37	(-0.03, 0.78)	0.21		0.40	(0.05, 0.75)	0.18		0.21	(-0.17, 0.60)	0.20	
	1943-1953; age 70-79	0.06	(-0.92, 1.04)	0.50		0.01	(-0.59, 0.61)	0.30		-0.03	(-0.80, 0.74)	0.39	
	1943 or earlier; age 80+	-0.46	(-2.42, 1.51)	1.00		-1.67	(-3.94, 0.60)	1.16		0.07	(-1.33, 1.47)	0.71	
Gender	(Ref: Male)				<.001				0.002				0.003
	Female	0.40	(0.24, 0.56)	0.08		0.16	(0.03, 0.30)	0.07		0.20	(0.06, 0.33)	0.07	
	Other	-3.46	(-5.92, -1.01)	1.25		-1.78	(-3.24, -0.32)	0.74		-3.25	(-6.97, 0.46)	1.90	
Religious affiliation	(Ref: Islam)				0.205				0.150				0.828
	Christianity	0.15	(-0.25, 0.56)	0.21		-0.17	(-0.46, 0.13)	0.15		0.03	(-0.24, 0.29)	0.13	
	Collapsed affiliations with prevalence<3%	-1.13	(-2.42, 0.16)	0.66		-0.75	(-1.74, 0.23)	0.50		-0.16	(-0.71, 0.39)	0.28	

		Cantril's Ladder				Life Satisfaction				Happiness			
Variable	Category	Est	95% CI	SE	Global p-value	Est	95% CI	SE	Global p-value	Est	95% CI	SE	Global p-value
Race/ethnicity	(Ref: Plurality group)				0.155				0.574				0.025
	Non-plurality groups	0.13	(-0.07, 0.32)	0.10		0.04	(-0.12, 0.20)	0.08		0.16	(0.01, 0.31)	0.07	

Table S9d. Sensitivity to unmeasured confounding of childhood predictors in Indonesia

Variable	Category	Cantril's Ladder		Life Satisfaction		Happiness	
		E-value for Estimate	E-value for 95% CI	E-value for Estimate	E-value for 95% CI	E-value for Estimate	E-value for 95% CI
Relationship with mother	(Ref: Very bad/somewhat bad)						
	Very good/somewhat good	1.60	1.00	2.02	1.00	2.22	1.25
Relationship with father	(Ref: Very bad/somewhat bad)						
	Very good/somewhat good	1.04	1.00	1.17	1.00	1.17	1.00
Parent marital status	(Ref: Parents married)						
	Divorced	1.47	1.00	1.26	1.00	1.15	1.00
	Parents were never married	1.30	1.00	1.74	1.00	1.21	1.00
	One or both parents had died	1.38	1.00	1.22	1.00	1.20	1.00
Subjective financial status of family growing up	(Ref: Got by)						
	Lived comfortably	1.68	1.52	1.67	1.51	1.58	1.41
	Found it difficult	1.63	1.21	1.38	1.00	1.69	1.22
	Found it very difficult	1.79	1.00	1.50	1.00	1.86	1.00
Abuse	(Ref: No)						
	Yes	1.79	1.41	1.90	1.44	1.94	1.50
Outsider growing up	(Ref: No)						
	Yes	1.12	1.00	1.53	1.00	1.66	1.03
Self-rated health growing up	(Ref: Good)						
	Excellent	1.34	1.00	1.47	1.18	1.56	1.34
	Very good	1.08	1.00	1.19	1.00	1.28	1.00
	Fair	1.33	1.00	1.42	1.13	1.32	1.00
	Poor	1.34	1.00	1.72	1.00	1.50	1.00
Immigration status	(Ref: Born in this country)						
	Born in another country	1.56	1.00	1.79	1.00	1.19	1.00
Age 12 religious service attendance	(Ref: Never)						
	At least 1/week	1.40	1.00	1.37	1.00	1.72	1.00
	1-3/month	1.36	1.00	1.54	1.00	1.80	1.00
	< 1/month	1.37	1.00	1.34	1.00	1.49	1.00
Year of birth	(Ref: 1998-2005; current age: 18-24)						
	1993-1998; age 25-29	1.16	1.00	1.53	1.28	1.33	1.00
	1983-1993; age 30-39	1.06	1.00	1.54	1.28	1.36	1.00
	1973-1983; age 40-49	1.28	1.00	1.43	1.08	1.33	1.00
	1963-1973; age 50-59	1.29	1.00	1.40	1.00	1.21	1.00

Variable	Category	Cantril's Ladder		Life Satisfaction		Happiness	
		E-value for Estimate	E-value for 95% CI	E-value for Estimate	E-value for 95% CI	E-value for Estimate	E-value for 95% CI
	1953-1963; age 60-69	1.55	1.00	1.62	1.16	1.41	1.00
	1943-1953; age 70-79	1.17	1.00	1.07	1.00	1.13	1.00
	1943 or earlier; age 80+	1.64	1.00	3.30	1.00	1.21	1.00
Gender	(Ref: Male)						
	Female	1.58	1.40	1.33	1.11	1.39	1.19
	Other	6.43	2.23	3.48	1.53	7.18	1.00
Religious affiliation	(Ref: Islam)						
	Christianity	1.30	1.00	1.34	1.00	1.12	1.00
	Collapsed affiliations with prevalence<3%	2.37	1.00	2.04	1.00	1.34	1.00
Race/ethnicity	(Ref: Plurality group)						
	Non-plurality groups	1.27	1.00	1.15	1.00	1.34	1.08

Table S9e. Complete-case supplemental analysis of means by demographic category for Indonesia

		Cantril's Ladder				Life Satisfaction				Happiness			
Variable	Category	Mean	95% CI	SE	Global p-value	Mean	95% CI	SE	Global p-value	Mean	95% CI	SE	Global p-value
Age group	18-24	7.12	(6.95,7.28)	0.08	0.181	8.02	(7.87,8.16)	0.07	0.010	8.09	(7.95,8.24)	0.07	0.201
	25-29	7.23	(7.06,7.40)	0.09		8.28	(8.14,8.43)	0.07		8.28	(8.14,8.43)	0.08	
	30-39	7.07	(6.92,7.22)	0.08		8.28	(8.16,8.40)	0.06		8.20	(8.08,8.33)	0.06	
	40-49	6.96	(6.79,7.13)	0.09		8.15	(8.02,8.29)	0.07		8.27	(8.14,8.40)	0.07	
	50-59	7.11	(6.89,7.34)	0.11		8.11	(7.95,8.27)	0.08		8.03	(7.86,8.19)	0.08	
	60-69	7.39	(7.10,7.69)	0.15		8.30	(8.05,8.55)	0.13		8.20	(7.92,8.49)	0.15	
	70-79	7.03	(6.09,7.96)	0.47		7.73	(7.09,8.38)	0.32		7.82	(7.03,8.62)	0.40	
	80 or older	5.84	(3.51,8.18)	0.98		5.74	(2.93,8.54)	1.18		7.41	(5.72,9.10)	0.71	
Gender	Female	7.30	(7.19,7.40)	0.05	< .001	8.23	(8.14,8.31)	0.04	0.001	8.28	(8.19,8.36)	0.04	0.001
	Male	6.90	(6.77,7.03)	0.07		8.11	(8.01,8.21)	0.05		8.07	(7.97,8.17)	0.05	
	Other	3.39	(0.00,8.28)	1.26		6.08	(3.53,8.64)	0.66		4.75	(0.00,12.01)	1.87	
Marital status	Divorced	6.68	(6.16,7.21)	0.27	0.377	7.97	(7.55,8.40)	0.22	0.026	7.79	(7.33,8.25)	0.23	0.006
	Domestic partner	7.02	(5.71,8.33)	0.58		8.49	(7.21,9.76)	0.57		8.73	(7.85,9.60)	0.39	
	Married	7.14	(7.04,7.24)	0.05		8.24	(8.16,8.32)	0.04		8.24	(8.17,8.32)	0.04	
	Separated	6.37	(5.36,7.39)	0.51		8.00	(7.33,8.67)	0.34		7.88	(7.18,8.58)	0.35	
	Single/Never been married	7.04	(6.88,7.19)	0.08		7.99	(7.85,8.13)	0.07		8.00	(7.86,8.14)	0.07	
	Widowed	7.16	(6.86,7.47)	0.15		8.03	(7.75,8.31)	0.14		8.10	(7.84,8.36)	0.13	
Employment	Employed for an employer	6.99	(6.80,7.17)	0.09	0.003	7.97	(7.81,8.13)	0.08	< .001	8.04	(7.89,8.18)	0.07	< .001
	Homemaker	7.28	(7.14,7.41)	0.07		8.32	(8.21,8.42)	0.05		8.39	(8.27,8.50)	0.06	
	None of these/Other	7.02	(6.68,7.36)	0.17		8.18	(7.93,8.43)	0.13		8.10	(7.88,8.32)	0.11	
	Retired	7.42	(7.02,7.82)	0.20		8.45	(8.10,8.81)	0.18		7.97	(7.54,8.41)	0.22	
	Self-employed	7.11	(6.98,7.25)	0.07		8.24	(8.13,8.35)	0.06		8.19	(8.08,8.30)	0.06	
	Student	6.97	(6.63,7.32)	0.17		7.85	(7.59,8.10)	0.13		7.82	(7.56,8.09)	0.13	
	Unemployed and looking for a job	6.69	(6.40,6.98)	0.15		7.84	(7.55,8.14)	0.15		7.84	(7.56,8.12)	0.14	
Religious service attendance	A few times a year	6.90	(6.70,7.09)	0.10	0.039	7.83	(7.64,8.02)	0.10	< .001	7.89	(7.71,8.07)	0.09	0.006
	More than once a week	7.22	(7.08,7.36)	0.07		8.27	(8.17,8.37)	0.05		8.28	(8.18,8.39)	0.05	
	Never	6.90	(6.52,7.29)	0.20		8.15	(7.84,8.46)	0.16		8.17	(7.85,8.48)	0.16	
	Once a week	7.03	(6.90,7.16)	0.07		8.20	(8.09,8.32)	0.06		8.16	(8.04,8.28)	0.06	

Variable	Category	Cantril's Ladder				Life Satisfaction				Happiness			
		Mean	95% CI	SE	Global p-value	Mean	95% CI	SE	Global p-value	Mean	95% CI	SE	Global p-value
	One to three times a month	7.16	(6.97,7.36)	0.10		8.00	(7.83,8.16)	0.08		8.08	(7.90,8.25)	0.09	
Education	Up to 8 years	7.13	(6.97,7.28)	0.08	0.745	8.27	(8.16,8.39)	0.06	0.015	8.32	(8.19,8.44)	0.06	0.001
	16+ years	7.03	(6.85,7.21)	0.09		8.02	(7.86,8.17)	0.08		7.96	(7.80,8.12)	0.08	
	9 to 15 years	7.08	(6.98,7.18)	0.05		8.09	(8.02,8.17)	0.04		8.07	(8.00,8.15)	0.04	
Immigration status	Born in another country	6.94	(6.28,7.59)	0.32	0.613	8.12	(7.54,8.69)	0.28	0.854	8.14	(7.29,8.99)	0.42	0.938
	Born in this country	7.10	(7.01,7.19)	0.05		8.17	(8.10,8.24)	0.03		8.17	(8.11,8.24)	0.03	
Religious affiliation	Buddhism	5.00	*	*	< .001	5.00	*	*	< .001	6.50	*	*	< .001
	Christianity	7.37	(6.99,7.75)	0.19		8.25	(7.96,8.55)	0.15		8.31	(7.97,8.66)	0.18	
	Hinduism	6.32	(5.29,7.35)	0.52		7.79	(7.33,8.24)	0.23		7.89	(7.37,8.41)	0.26	
	Islam	7.09	(7.00,7.18)	0.05		8.17	(8.10,8.24)	0.04		8.17	(8.10,8.23)	0.03	
	Some other religion	7.00	*	*		7.00	*	*		7.00	*	*	
	Taoism	5.00	*	*		9.00	*	*		6.00	*	*	

Table S9f. Complete-case supplemental analysis of childhood predictors regression analysis results for Indonesia

		Cantril's Ladder				Life Satisfaction				Happiness			
Variable	Category	Est	95% CI	SE	Global p-value	Est	95% CI	SE	Global p-value	Est	95% CI	SE	Global p-value
Relationship with mother	(Ref: Very bad/somewhat bad)				0.365				0.274				0.257
	Very good/somewhat good	0.25	(-0.30,0.81)	0.28		0.34	(-0.27,0.95)	0.31		0.34	(-0.25,0.92)	0.30	
Relationship with father	(Ref: Very bad/somewhat bad)				0.698				0.398				0.801
	Very good/somewhat good	0.09	(-0.38,0.56)	0.24		0.19	(-0.25,0.63)	0.22		0.05	(-0.35,0.45)	0.20	
Parent marital status	(Ref: Parents married)				0.193				0.332				0.501
	No, one or both of them had died	0.13	(-0.26,0.52)	0.20		0.13	(-0.21,0.46)	0.17		0.17	(-0.15,0.50)	0.16	
	No, they were never married	-0.32	(-1.35,0.71)	0.53		0.49	(-0.17,1.14)	0.33		-0.16	(-1.10,0.77)	0.48	
	Yes, married	0.28	(-0.04,0.59)	0.16		0.01	(-0.26,0.28)	0.14		0.01	(-0.26,0.27)	0.13	
Subjective financial status of family growing up	(Ref: Got by)				<.001				<.001				<.001
	Found it difficult	-0.48	(-0.80,-0.15)	0.17		-0.09	(-0.40,0.22)	0.16		-0.35	(-0.64,-0.05)	0.15	
	Found it very difficult	-0.81	(-1.42,-0.20)	0.31		-0.22	(-0.72,0.27)	0.25		-0.52	(-1.04,-0.00)	0.27	
	Lived comfortably	0.43	(0.29,0.57)	0.07		0.37	(0.25,0.49)	0.06		0.30	(0.18,0.42)	0.06	
Abuse	(Ref: No)				0.003				0.004				0.063
	Yes	-0.45	(-0.76,-0.15)	0.15		-0.42	(-0.71,-0.14)	0.15		-0.25	(-0.51,0.01)	0.13	
Outsider growing up	(Ref: No)				0.515				0.303				0.033
	Yes	0.12	(-0.25,0.49)	0.19		-0.17	(-0.49,0.15)	0.16		-0.35	(-0.67,-0.03)	0.16	

		Cantril's Ladder				Life Satisfaction				Happiness			
Variable	Category	Est	95% CI	SE	Global p-value	Est	95% CI	SE	Global p-value	Est	95% CI	SE	Global p-value
Self-rated health growing up	(Ref: Good)				0.165				0.003				<.001
	Excellent	0.27	(0.03,0.51)	0.12		0.29	(0.10,0.48)	0.10		0.32	(0.15,0.48)	0.08	
	Fair	-0.03	(-0.25,0.19)	0.11		-0.16	(-0.34,0.02)	0.09		-0.11	(-0.28,0.06)	0.09	
	Poor	0.10	(-1.03,1.23)	0.57		-0.24	(-1.38,0.90)	0.58		0.06	(-0.87,0.99)	0.47	
	Very good	0.09	(-0.09,0.26)	0.09		0.07	(-0.07,0.21)	0.07		0.12	(-0.02,0.26)	0.07	
Immigration status	(Ref: Born in this country)				0.715				0.796				0.868
	Born in another country	-0.12	(-0.79,0.54)	0.34		-0.08	(-0.72,0.55)	0.32		-0.07	(-0.86,0.72)	0.40	
Age 12 religious service attendance	(Ref: Never)				0.939				0.244				0.333
	At least once a week	-0.10	(-0.45,0.26)	0.18		-0.17	(-0.44,0.10)	0.14		0.10	(-0.26,0.46)	0.18	
	Less than once a month	-0.09	(-0.54,0.36)	0.23		-0.12	(-0.51,0.28)	0.20		-0.13	(-0.56,0.31)	0.22	
	One to three times a month	-0.06	(-0.47,0.34)	0.21		-0.29	(-0.59,0.01)	0.15		0.15	(-0.26,0.56)	0.21	
Year of birth	(Ref: 1998-2005; current age: 18-24)				0.037				<.001				0.144
	1993-1998; age 25-29	0.11	(-0.12,0.34)	0.12		0.28	(0.09,0.47)	0.10		0.19	(-0.01,0.39)	0.10	
	1983-1993; age 30-39	0.04	(-0.16,0.23)	0.10		0.34	(0.16,0.52)	0.09		0.17	(-0.01,0.35)	0.09	
	1973-1983; age 40-49	-0.02	(-0.24,0.19)	0.11		0.24	(0.04,0.44)	0.10		0.26	(0.08,0.45)	0.09	
	1963-1973; age 50-59	0.23	(-0.03,0.50)	0.14		0.25	(0.03,0.47)	0.11		0.10	(-0.11,0.32)	0.11	
	1953-1963; age 60-69	0.56	(0.21,0.91)	0.18		0.46	(0.15,0.76)	0.15		0.31	(-0.02,0.64)	0.17	
	1943-1953; age 70-79	0.03	(-0.87,0.93)	0.46		-0.18	(-0.79,0.43)	0.31		-0.13	(-0.90,0.63)	0.39	

Variable	Category	Cantril's Ladder				Life Satisfaction				Happiness			
Variable	Category	Est	95% CI	SE	Global p-value	Est	95% CI	SE	Global p-value	Est	95% CI	SE	Global p-value
	1943 or earlier; age 80+	-0.44	(-2.35,1.46)	0.97		-1.91	(-4.14,0.31)	1.13		-0.04	(-1.40,1.33)	0.70	
Gender	(Ref: Male)				<.001				0.005				0.003
	Male	-0.36	(-0.51,-0.22)	0.08		-0.09	(-0.21,0.03)	0.06		-0.18	(-0.31,-0.05)	0.07	
	Other	-3.95	(-6.43,-1.47)	1.27		-2.08	(-3.44,-0.71)	0.70		-3.49	(-7.17,0.20)	1.88	
Religious affiliation	(Ref: Islam)				0.191				0.292				0.384
	Christianity	0.18	(-0.18,0.54)	0.18		0.07	(-0.22,0.35)	0.15		0.10	(-0.20,0.40)	0.15	
	Christianity	0.18	(-0.18,0.54)	0.18		0.07	(-0.22,0.35)	0.15		0.10	(-0.20,0.40)	0.15	
	Collapsed affiliations with prevalence<3%	-0.90	(-1.99,0.18)	0.55		-0.37	(-0.84,0.10)	0.24		-0.32	(-0.83,0.20)	0.26	
Race/ethnicity	(Ref: Plurality group)				0.008				0.008				<.001
	Non-plurality groups	0.24	(0.06,0.42)	0.09		0.19	(0.05,0.33)	0.07		0.23	(0.09,0.36)	0.07	

Table S9g. Complete-case supplemental analysis of sensitivity to unmeasured confounding of childhood predictors in Indonesia

		Cantril's Ladder		Life Satisfaction		Happiness	
Variable	Category	E-value for Estimate	E-value for 95% CI	E-value for Estimate	E-value for 95% CI	E-value for Estimate	E-value for 95% CI
Relationship with mother	(Ref: Very bad/somewhat bad)						
	Very good/somewhat good	1.44	1.00	1.62	1.00	1.62	1.00
Relationship with father	(Ref: Very bad/somewhat bad)						
	Very good/somewhat good	1.23	1.00	1.41	1.00	1.18	1.00
Parent marital status	(Ref: Parents married)						
	No, one or both of them had died	1.29	1.00	1.31	1.00	1.39	1.00
	No, they were never married	1.52	1.00	1.81	1.00	1.37	1.00
	Yes, married	1.46	1.00	1.07	1.00	1.06	1.00
Subjective financial status of family growing up	(Ref: Got by)						
	Found it difficult	1.70	1.32	1.25	1.00	1.63	1.18
	Found it very difficult	2.07	1.37	1.45	1.00	1.86	1.03
	Lived comfortably	1.64	1.48	1.65	1.49	1.56	1.39
Abuse	(Ref: No)						
	Yes	1.67	1.32	1.73	1.33	1.49	1.00
Outsider growing up	(Ref: No)						
	Yes	1.27	1.00	1.38	1.00	1.63	1.13
Self-rated health growing up	(Ref: Good)						
	Excellent	1.46	1.12	1.54	1.26	1.59	1.36
	Fair	1.12	1.00	1.36	1.00	1.28	1.00
	Poor	1.24	1.00	1.48	1.00	1.19	1.00
	Very good	1.22	1.00	1.21	1.00	1.30	1.00
Immigration status	(Ref: Born in this country)						
	Born in another country	1.28	1.00	1.24	1.00	1.21	1.00
Age 12 religious service attendance	(Ref: Never)						
	At least once a week	1.24	1.00	1.38	1.00	1.27	1.00
	Less than once a month	1.23	1.00	1.29	1.00	1.32	1.00
	One to three times a month	1.19	1.00	1.54	1.00	1.35	1.00
Year of birth	(Ref: 1998-2005; current age: 18-24)						

Variable	Category	Cantril’s Ladder		Life Satisfaction		Happiness	
		E-value for Estimate	E-value for 95% CI	E-value for Estimate	E-value for 95% CI	E-value for Estimate	E-value for 95% CI
	1993-1998; age 25-29	1.26	1.00	1.53	1.26	1.41	1.00
	1983-1993; age 30-39	1.13	1.00	1.62	1.37	1.38	1.00
	1973-1983; age 40-49	1.10	1.00	1.47	1.15	1.51	1.24
	1963-1973; age 50-59	1.41	1.00	1.49	1.13	1.28	1.00
	1953-1963; age 60-69	1.79	1.39	1.77	1.36	1.58	1.00
	1943-1953; age 70-79	1.13	1.00	1.39	1.00	1.33	1.00
	1943 or earlier; age 80+	1.66	1.00	4.26	1.00	1.15	1.00
Gender	(Ref: Male)						
	Male	1.57	1.39	1.26	1.00	1.40	1.19
	Other	8.63	2.92	4.64	2.12	9.58	1.00
Religious affiliation	(Ref: Islam)						
	Christianity	1.35	1.00	1.21	1.00	1.27	1.00
	Christianity	1.35	1.00	1.21	1.00	1.27	1.00
	Collapsed affiliations with prevalence<3%	2.18	1.00	1.66	1.00	1.59	1.00
Race/ethnicity	(Ref: Plurality group)						
	Non-plurality groups	1.42	1.18	1.41	1.18	1.46	1.26

Tables S10a-g: Israel

Table S10a. Nationally representative descriptive statistics for Israel

Characteristic	N = 3,669[1]
**Age group**	
1998-2005; age 18-24	553 (15%)
1993-1998; age 25-29	407 (11%)
1983-1993; age 30-39	666 (18%)
1973-1983; age 40-49	616 (17%)
1963-1973; age 50-59	542 (15%)
1953-1963; age 60-69	469 (13%)
1943-1953; age 70-79	336 (9.2%)
1943 or earlier; age 80+	79 (2.2%)
(Missing)	0 (0%)
**Gender**	
Male	1,791 (49%)
Female	1,872 (51%)
Other	0 (<0.1%)
(Missing)	6 (0.2%)
**Race/Ethnicity**	
Arab	674 (18%)
Jewish	2,926 (80%)
Other	39 (1.1%)
(Missing)	30 (0.8%)
**Respondent Marital status**	
Married	2,056 (56%)
Separated	48 (1.3%)
Divorced	258 (7.0%)
Widowed	212 (5.8%)
Single, never married	834 (23%)
Domestic Partner	193 (5.3%)
(Missing)	69 (1.9%)
**Employment**	
Employed for an employer	1,793 (49%)
Self-employed	424 (12%)
Retired	576 (16%)
Student	388 (11%)
Homemaker	211 (5.7%)
Unemployed and looking for a job	148 (4.0%)
None of these/Other	118 (3.2%)
(Missing)	10 (0.3%)
**Religious service attendance as an adult (now)**	
More than 1/week	649 (18%)
1/week	495 (14%)
1-3/month	374 (10%)
A few times a year	1,014 (28%)
Never	1,122 (31%)
(Missing)	14 (0.4%)
**Education (years)**	
Up to 8 years	224 (6.1%)
9-15 years	1,517 (41%)
16+ years	1,926 (52%)
(Missing)	2 (<0.1%)
**Immigration status**	
Born in this country	2,796 (76%)
Born in another country	868 (24%)
(Missing)	5 (0.1%)
**Religious affiliation as an adult (now)**	
Christianity	39 (1.1%)
Islam	656 (18%)
Hinduism	0 (0%)
Buddhism	0 (0%)
Judaism	2,897 (79%)
Sikhism	0 (0%)
Baha'i	2 (<0.1%)

Characteristic	N = 3,669[1]
Jainism	0 (0%)
Shinto	0 (0%)
Taoism	1 (<0.1%)
Confucianism	0 (0%)
Primal, Animist, or Folk religion	1 (<0.1%)
Spiritism	0 (0%)
Umbanda, Candomble, and other African-derived religions	0 (0%)
Chinese folk/traditional religion	0 (0%)
Some other religion	5 (0.1%)
No religion/Atheist/Agnostic	64 (1.7%)
(Missing)	4 (0.1%)
**Relationship with mother growing up**	
Very good	2,686 (73%)
Somewhat good	793 (22%)
Somewhat bad	110 (3.0%)
Very bad	18 (0.5%)
Does not apply	45 (1.2%)
(Missing)	17 (0.5%)
**Relationship with father growing up**	
Very good	2,290 (62%)
Somewhat good	912 (25%)
Somewhat bad	234 (6.4%)
Very bad	37 (1.0%)
Does not apply	171 (4.7%)
(Missing)	25 (0.7%)
**Parent marital status at age 12**	
Parents married	3,172 (86%)
Divorced	284 (7.8%)
Parents were never married	36 (1.0%)
One or both parents had died	130 (3.5%)
(Missing)	47 (1.3%)
**Subjective financial status of family growing up**	
Lived comfortably	923 (25%)
Got by	1,822 (50%)
Found it difficult	667 (18%)
Found it very difficult	239 (6.5%)
(Missing)	17 (0.5%)
**Abuse**	
Yes	0 (0%)
No	0 (0%)
(Missing)	3,669 (100%)
**Outsider growing up**	
Yes	371 (10%)
No	3,228 (88%)
(Missing)	70 (1.9%)
**Self-rated health growing up**	
Excellent	1,785 (49%)
Very good	1,284 (35%)
Good	480 (13%)
Fair	105 (2.9%)
Poor	6 (0.2%)
(Missing)	8 (0.2%)
**Age 12 religious service attendance**	
At least 1/week	867 (24%)
1-3/month	435 (12%)
<1/month	810 (22%)
Never	1,539 (42%)
(Missing)	17 (0.5%)
**Religious affiliation at age 12**	
Christianity	60 (1.6%)
Islam	647 (18%)
Hinduism	0 (0%)
Buddhism	0 (0%)
Judaism	2,873 (78%)
Sikhism	1 (<0.1%)

**Characteristic**	**N = 3,669**[1]
Baha'i	1 (<0.1%)
Jainism	0 (0%)
Shinto	0 (0%)
Taoism	0 (0%)
Confucianism	0 (0%)
Primal, Animist, or Folk religion	3 (<0.1%)
Spiritism	0 (0%)
Umbanda, Candomble, and other African-derived religions	0 (0%)
Chinese folk/traditional religion	0 (0%)
Some other religion	5 (0.1%)
No religion/Atheist/Agnostic	69 (1.9%)
(Missing)	10 (0.3%)

[1]n (%)

Table S10b. Means by demographic category for Israel

Variable	Category	Cantril's Ladder				Life Satisfaction				Happiness			
		Mean	95% CI	SE	Global p-value	Mean	95% CI	SE	Global p-value	Mean	95% CI	SE	Global p-value
Age group	18-24	7.71	(7.46,7.97)	0.13	< .001	8.01	(7.79,8.22)	0.11	< .001	8.08	(7.85,8.32)	0.12	< .001
	25-29	7.56	(7.35,7.77)	0.11		7.92	(7.70,8.15)	0.11		7.94	(7.73,8.15)	0.11	
	30-39	7.44	(7.23,7.65)	0.11		7.47	(7.24,7.70)	0.12		7.80	(7.61,7.98)	0.09	
	40-49	7.31	(7.11,7.52)	0.11		7.37	(7.13,7.61)	0.12		7.81	(7.63,7.99)	0.09	
	50-59	7.25	(7.03,7.47)	0.11		7.21	(6.94,7.47)	0.13		7.63	(7.44,7.82)	0.10	
	60-69	7.33	(7.11,7.55)	0.11		7.53	(7.31,7.75)	0.11		7.76	(7.57,7.94)	0.09	
	70-79	6.67	(6.35,6.98)	0.16		6.76	(6.38,7.14)	0.19		7.24	(7.01,7.48)	0.12	
	80 or older	6.09	(5.54,6.64)	0.28		6.76	(6.05,7.48)	0.36		6.97	(6.46,7.49)	0.26	
Gender	Male	7.47	(7.31,7.62)	0.08	< .001	7.56	(7.39,7.72)	0.08	< .001	7.84	(7.71,7.98)	0.07	< .001
	Female	7.20	(7.04,7.37)	0.08		7.39	(7.20,7.59)	0.10		7.68	(7.55,7.81)	0.07	
	Other	9.00	*	*		7.00	*	*		7.00	*	*	
Marital status	Married	7.52	(7.36,7.67)	0.08	< .001	7.65	(7.47,7.84)	0.09	< .001	7.98	(7.86,8.10)	0.06	< .001
	Separated	6.61	(6.03,7.19)	0.29		6.88	(6.31,7.46)	0.29		7.08	(6.63,7.53)	0.22	
	Divorced	6.63	(6.31,6.95)	0.16		6.43	(6.03,6.82)	0.20		6.97	(6.68,7.27)	0.15	
	Widowed	6.50	(6.17,6.84)	0.17		6.88	(6.51,7.26)	0.19		6.89	(6.58,7.20)	0.16	
	Never	7.46	(7.26,7.66)	0.10		7.69	(7.50,7.88)	0.10		7.74	(7.54,7.93)	0.10	
	Domestic Partner	6.85	(6.43,7.26)	0.21		6.81	(6.41,7.20)	0.20		7.71	(7.45,7.96)	0.13	
Employment	Employed for an employer	7.46	(7.31,7.60)	0.07	< .001	7.54	(7.37,7.71)	0.09	< .001	7.84	(7.71,7.96)	0.06	< .001
	Self-employed	7.39	(7.12,7.65)	0.13		7.29	(6.96,7.61)	0.16		7.82	(7.54,8.11)	0.14	
	Retired	6.79	(6.51,7.07)	0.14		7.03	(6.69,7.36)	0.17		7.39	(7.17,7.61)	0.11	
	Student	7.89	(7.59,8.20)	0.15		8.19	(7.91,8.47)	0.14		8.24	(7.94,8.54)	0.15	
	Homemaker	6.94	(6.68,7.20)	0.13		7.51	(7.10,7.92)	0.21		7.37	(7.07,7.66)	0.15	
	Unemployed and looking for a job	6.60	(6.17,7.04)	0.22		6.80	(6.39,7.22)	0.21		6.99	(6.52,7.45)	0.23	
	None of these/Other	7.65	(7.31,7.99)	0.17		7.74	(7.37,8.11)	0.19		8.20	(7.89,8.52)	0.16	
Religious service attendance	More than 1/week	8.11	(7.90,8.32)	0.11	< .001	8.44	(8.24,8.64)	0.10	< .001	8.55	(8.34,8.77)	0.11	< .001
	1/week	7.52	(7.30,7.73)	0.11		7.78	(7.48,8.08)	0.15		7.97	(7.75,8.20)	0.11	
	1-3/month	7.28	(7.05,7.51)	0.12		7.55	(7.31,7.80)	0.12		7.73	(7.55,7.92)	0.09	
	A few times a year	7.06	(6.85,7.26)	0.10		7.23	(7.04,7.42)	0.10		7.52	(7.34,7.70)	0.09	
	Never	7.06	(6.82,7.31)	0.13		6.97	(6.68,7.27)	0.15		7.43	(7.26,7.61)	0.09	
Education	Up to 8 years	6.53	(6.19,6.86)	0.17	< .001	7.11	(6.69,7.53)	0.21	0.048	7.04	(6.74,7.34)	0.15	< .001
	9-15 years	7.38	(7.21,7.54)	0.08		7.56	(7.39,7.74)	0.09		7.79	(7.64,7.94)	0.08	

Variable	Category	Cantril's Ladder				Life Satisfaction				Happiness			
		Mean	95% CI	SE	Global p-value	Mean	95% CI	SE	Global p-value	Mean	95% CI	SE	Global p-value
	16+ years	7.39	(7.22,7.56)	0.09		7.44	(7.24,7.65)	0.10		7.82	(7.68,7.96)	0.07	
Immigration status	Born in this country	7.44	(7.29,7.59)	0.08	< .001	7.62	(7.45,7.80)	0.09	< .001	7.89	(7.77,8.01)	0.06	< .001
	Born in another country	6.99	(6.77,7.21)	0.11		7.00	(6.79,7.22)	0.11		7.34	(7.14,7.53)	0.10	
Religious affiliation	Christianity	6.64	(6.04,7.25)	0.30	< .001	7.20	(6.35,8.05)	0.42	< .001	7.06	(6.63,7.50)	0.21	< .001
	Islam	6.92	(6.68,7.15)	0.12		7.27	(7.03,7.51)	0.12		7.07	(6.82,7.33)	0.13	
	Judaism	7.44	(7.26,7.61)	0.09		7.53	(7.33,7.73)	0.10		7.93	(7.80,8.06)	0.07	
	Baha'i	10.00	*	*		10.00	*	*		10.00	*	*	
	Taoism	5.00	*	*		4.00	*	*		5.00	*	*	
	Primal, Animist, or Folk religion	8.00	*	*		9.00	*	*		8.00	*	*	
	Some other religion	6.98	(5.89,8.08)	0.55		7.10	(6.18,8.01)	0.46		7.10	(6.18,8.01)	0.46	
	No religion/Atheist /Agnostic	7.13	(6.58,7.67)	0.27		7.24	(6.73,7.75)	0.25		7.58	(6.99,8.17)	0.30	
Race/Ethnicity	Arab	6.88	(6.66,7.11)	0.11	< .001	7.27	(7.03,7.50)	0.12	0.271	7.06	(6.82,7.31)	0.12	< .001
	Jewish	7.43	(7.26,7.61)	0.09		7.52	(7.32,7.72)	0.10		7.92	(7.79,8.05)	0.07	
	Other	7.56	(7.08,8.03)	0.24		7.49	(6.90,8.08)	0.29		7.83	(7.30,8.36)	0.26	

Table S10c. Childhood predictors regression analysis results for Israel

		Cantril's Ladder				Life Satisfaction				Happiness			
Variable	Category	Est	95% CI	SE	Global p-value	Est	95% CI	SE	Global p-value	Est	95% CI	SE	Global p-value
Relationship with mother	(Ref: Very bad/somewhat bad)				0.203				0.160				0.919
	Very good/somewhat good	0.27	(-0.15, 0.69)	0.21		0.33	(-0.13, 0.80)	0.24		0.01	(-0.34, 0.35)	0.17	
Relationship with father	(Ref: Very bad/somewhat bad)				0.173				0.190				0.601
	Very good/somewhat good	0.26	(-0.11, 0.63)	0.19		0.24	(-0.12, 0.61)	0.19		0.08	(-0.22, 0.37)	0.15	
Parent marital status	(Ref: Parents married)				0.031				<.001				0.003
	Divorced	-0.14	(-0.43, 0.15)	0.15		-0.66	(-1.03, -0.28)	0.19		-0.49	(-0.80, -0.18)	0.16	
	Parents were never married	-0.71	(-1.61, 0.19)	0.44		-0.03	(-0.60, 0.55)	0.29		-0.11	(-0.61, 0.39)	0.25	
	One or both parents had died	-0.44	(-0.87, -0.01)	0.22		-0.53	(-0.96, -0.11)	0.22		-0.41	(-0.82, -0.01)	0.20	
Subjective financial status of family growing up	(Ref: Got by)				0.641				0.086				0.893
	Lived comfortably	0.06	(-0.11, 0.23)	0.09		0.12	(-0.07, 0.31)	0.10		0.05	(-0.12, 0.22)	0.09	
	Found it difficult	0.02	(-0.16, 0.19)	0.09		0.18	(-0.01, 0.37)	0.10		0.03	(-0.12, 0.19)	0.08	
	Found it very difficult	-0.19	(-0.57, 0.19)	0.19		-0.21	(-0.61, 0.20)	0.21		-0.05	(-0.33, 0.23)	0.14	
Outsider growing up	(Ref: No)				0.360				0.479				0.172
	Yes	-0.13	(-0.42, 0.16)	0.15		-0.10	(-0.41, 0.21)	0.16		-0.17	(-0.40, 0.07)	0.12	
Self-rated health growing up	(Ref: Good)				0.003				0.004				0.014
	Excellent	0.51	(0.23, 0.78)	0.14		0.54	(0.21, 0.86)	0.17		0.44	(0.20, 0.69)	0.13	
	Very good	0.34	(0.08, 0.60)	0.13		0.42	(0.14, 0.69)	0.14		0.36	(0.13, 0.59)	0.12	
	Fair	-0.00	(-0.49, 0.49)	0.25		-0.19	(-0.71, 0.34)	0.27		-0.07	(-0.44, 0.31)	0.19	

Variable	Category	Cantril's Ladder				Life Satisfaction				Happiness			
		Est	95% CI	SE	Global p-value	Est	95% CI	SE	Global p-value	Est	95% CI	SE	Global p-value
	Poor	0.38	(-0.83, 1.60)	0.62		0.15	(-0.93, 1.23)	0.55		0.58	(-1.51, 2.66)	1.06	
Immigration status	(Ref: Born in this country)				0.184				0.062				<.001
	Born in another country	-0.15	(-0.37, 0.07)	0.11		-0.24	(-0.50, 0.01)	0.13		-0.40	(-0.62, -0.18)	0.11	
Age 12 religious service attendance	(Ref: Never)				0.056				<.001				0.002
	At least 1/week	0.28	(0.05, 0.52)	0.12		0.43	(0.18, 0.68)	0.13		0.41	(0.19, 0.63)	0.11	
	1-3/month	0.33	(0.06, 0.59)	0.13		0.42	(0.11, 0.73)	0.16		0.30	(0.06, 0.54)	0.12	
	< 1/month	0.20	(0.00, 0.41)	0.10		0.50	(0.29, 0.72)	0.11		0.28	(0.13, 0.43)	0.08	
Year of birth	(Ref: 1998-2005; current age: 18-24)				<.001				<.001				<.001
	1993-1998; age 25-29	-0.15	(-0.38, 0.08)	0.12		-0.08	(-0.34, 0.18)	0.13		-0.16	(-0.38, 0.07)	0.12	
	1983-1993; age 30-39	-0.22	(-0.52, 0.08)	0.15		-0.42	(-0.69, -0.15)	0.14		-0.20	(-0.43, 0.03)	0.12	
	1973-1983; age 40-49	-0.33	(-0.63, -0.03)	0.15		-0.51	(-0.82, -0.21)	0.15		-0.18	(-0.43, 0.07)	0.13	
	1963-1973; age 50-59	-0.33	(-0.62, -0.04)	0.15		-0.63	(-0.95, -0.31)	0.16		-0.34	(-0.58, -0.11)	0.12	
	1953-1963; age 60-69	-0.24	(-0.54, 0.06)	0.15		-0.28	(-0.56, -0.01)	0.14		-0.21	(-0.47, 0.04)	0.13	
	1943-1953; age 70-79	-0.87	(-1.22, -0.52)	0.18		-1.00	(-1.40, -0.61)	0.20		-0.67	(-0.96, -0.38)	0.15	
	1943 or earlier; age 80+	-1.30	(-1.86, -0.73)	0.29		-0.82	(-1.56, -0.08)	0.38		-0.80	(-1.31, -0.29)	0.26	
Gender	(Ref: Male)				<.001				0.527				0.023
	Female	-0.21	(-0.33, -0.09)	0.06		-0.07	(-0.22, 0.08)	0.08		-0.08	(-0.20, 0.05)	0.06	
	Other	1.94	(1.43, 2.45)	0.26		0.15	(-0.40, 0.69)	0.28		-0.57	(-1.12, -0.01)	0.28	
Religious affiliation	(Ref: Judaism)				0.631				0.722				0.762
	Islam	0.20	(-0.48, 0.88)	0.35		-0.05	(-0.83, 0.72)	0.40		-0.20	(-0.91, 0.51)	0.36	
	Collapsed affiliations with prevalence<3%	-0.04	(-0.39, 0.31)	0.18		0.12	(-0.30, 0.54)	0.21		-0.12	(-0.50, 0.26)	0.19	
Race/ethnicity	(Ref: Plurality group)				0.004				0.247				0.006

		Cantril's Ladder				Life Satisfaction				Happiness			
Variable	Category	Est	95% CI	SE	Global p-value	Est	95% CI	SE	Global p-value	Est	95% CI	SE	Global p-value
Abuse	Non-plurality groups (Ref: No)	-0.92	(-1.56, -0.27)	0.33		-0.39	(-1.09, 0.30)	0.35		-0.89	(-1.58, -0.21)	0.35	

Table S10d. Sensitivity to unmeasured confounding of childhood predictors in Israel

		Cantril's Ladder		Life Satisfaction		Happiness	
Variable	Category	E-value for Estimate	E-value for 95% CI	E-value for Estimate	E-value for 95% CI	E-value for Estimate	E-value for 95% CI
Relationship with mother	(Ref: Very bad/somewhat bad)						
	Very good/somewhat good	1.55	1.00	1.60	1.00	1.06	1.00
Relationship with father	(Ref: Very bad/somewhat bad)						
	Very good/somewhat good	1.54	1.00	1.48	1.00	1.25	1.00
Parent marital status	(Ref: Parents married)						
	Divorced	1.35	1.00	2.03	1.53	1.95	1.45
	Parents were never married	2.21	1.00	1.12	1.00	1.32	1.00
	One or both parents had died	1.80	1.09	1.87	1.29	1.82	1.09
Subjective financial status of family growing up	(Ref: Got by)						
	Lived comfortably	1.20	1.00	1.30	1.00	1.19	1.00
	Found it difficult	1.10	1.00	1.39	1.00	1.15	1.00
	Found it very difficult	1.44	1.00	1.43	1.00	1.21	1.00
Outsider growing up	(Ref: No)						
	Yes	1.34	1.00	1.26	1.00	1.42	1.00
Self-rated health growing up	(Ref: Good)						
	Excellent	1.90	1.50	1.87	1.44	1.87	1.47
	Very good	1.66	1.26	1.71	1.33	1.74	1.35
	Fair	1.03	1.00	1.40	1.00	1.23	1.00
	Poor	1.72	1.00	1.35	1.00	2.09	1.00
Immigration status	(Ref: Born in this country)						
	Born in another country	1.37	1.00	1.48	1.00	1.81	1.45
Age 12 religious service attendance	(Ref: Never)						
	At least 1/week	1.57	1.19	1.73	1.39	1.81	1.45
	1-3/month	1.64	1.21	1.71	1.28	1.64	1.22
	< 1/month	1.45	1.04	1.82	1.54	1.61	1.35
Year of birth	(Ref: 1998-2005; current age: 18-24)						
	1993-1998; age 25-29	1.37	1.00	1.23	1.00	1.41	1.00
	1983-1993; age 30-39	1.48	1.00	1.72	1.35	1.47	1.00
	1973-1983; age 40-49	1.64	1.14	1.84	1.44	1.44	1.00
	1963-1973; age 50-59	1.64	1.15	2.00	1.58	1.71	1.31
	1953-1963; age 60-69	1.51	1.00	1.53	1.07	1.50	1.00
	1943-1953; age 70-79	2.47	1.92	2.53	1.96	2.25	1.77

Variable	Category	Cantril's Ladder		Life Satisfaction		Happiness	
		E-value for Estimate	E-value for 95% CI	E-value for Estimate	E-value for 95% CI	E-value for Estimate	E-value for 95% CI
	1943 or earlier; age 80+	3.25	2.25	2.26	1.23	2.48	1.63
Gender	(Ref: Male)						
	Female	1.46	1.26	1.22	1.00	1.25	1.00
	Other	4.74	3.53	1.34	1.00	2.07	1.14
Religious affiliation	(Ref: Judaism)						
	Islam	1.45	1.00	1.18	1.00	1.47	1.00
	Collapsed affiliations with prevalence<3%	1.17	1.00	1.30	1.00	1.34	1.00
Race/ethnicity	(Ref: Plurality group)						
	Non-plurality groups	2.55	1.56	1.68	1.00	2.66	1.50
Abuse	(Ref: No)						

Table S10e. Complete-case supplemental analysis of means by demographic category for Israel

		Cantril's Ladder				Life Satisfaction				Happiness			
Variable	Category	Mean	95% CI	SE	Global p-value	Mean	95% CI	SE	Global p-value	Mean	95% CI	SE	Global p-value
Age group	18-24	7.76	(7.52,8.00)	0.12	< .001	8.07	(7.86,8.27)	0.10	< .001	8.12	(7.91,8.34)	0.11	< .001
	25-29	7.60	(7.40,7.80)	0.10		7.93	(7.71,8.15)	0.11		7.94	(7.73,8.15)	0.11	
	30-39	7.50	(7.32,7.69)	0.09		7.56	(7.35,7.77)	0.11		7.87	(7.72,8.03)	0.08	
	40-49	7.36	(7.17,7.55)	0.10		7.51	(7.31,7.71)	0.10		7.81	(7.63,7.99)	0.09	
	50-59	7.31	(7.10,7.52)	0.11		7.36	(7.13,7.58)	0.11		7.64	(7.46,7.82)	0.09	
	60-69	7.36	(7.14,7.58)	0.11		7.61	(7.39,7.83)	0.11		7.76	(7.57,7.94)	0.09	
	70-79	6.70	(6.40,7.00)	0.15		7.01	(6.69,7.32)	0.16		7.24	(7.01,7.48)	0.12	
	80 or older	6.20	(5.69,6.71)	0.26		7.18	(6.59,7.77)	0.30		7.02	(6.50,7.53)	0.26	
Gender	Female	7.25	(7.10,7.40)	0.08	< .001	7.52	(7.36,7.69)	0.08	< .001	7.69	(7.56,7.82)	0.07	< .001
	Male	7.52	(7.37,7.67)	0.08		7.66	(7.51,7.81)	0.08		7.88	(7.75,8.01)	0.07	
	Other	9.00	(9.00,9.00)	0.00		7.00	(7.00,7.00)	0.00		7.00	(7.00,7.00)	0.00	
Marital status	Divorced	6.74	(6.46,7.03)	0.14	< .001	6.65	(6.32,6.97)	0.17	< .001	7.10	(6.85,7.34)	0.13	< .001
	Domestic partner	6.83	(6.42,7.25)	0.21		7.00	(6.63,7.38)	0.19		7.72	(7.46,7.99)	0.13	
	Married	7.56	(7.42,7.70)	0.07		7.76	(7.61,7.91)	0.08		7.97	(7.85,8.10)	0.06	
	Separated	6.69	(6.18,7.21)	0.26		6.90	(6.38,7.42)	0.26		7.07	(6.65,7.48)	0.21	
	Single/Never been married	7.52	(7.33,7.71)	0.10		7.75	(7.57,7.93)	0.09		7.77	(7.59,7.96)	0.09	
	Widowed	6.58	(6.29,6.87)	0.15		7.09	(6.74,7.44)	0.18		6.99	(6.75,7.24)	0.12	
Employment	Employed for an employer	7.48	(7.35,7.61)	0.07	< .001	7.61	(7.44,7.77)	0.08	< .001	7.84	(7.71,7.96)	0.06	< .001
	Homemaker	6.99	(6.76,7.22)	0.12		7.55	(7.14,7.96)	0.21		7.39	(7.09,7.68)	0.15	
	None of these/Other	7.69	(7.36,8.02)	0.17		7.85	(7.50,8.20)	0.18		8.20	(7.89,8.51)	0.16	
	Retired	6.85	(6.59,7.11)	0.13		7.23	(6.96,7.51)	0.14		7.40	(7.18,7.62)	0.11	
	Self-employed	7.48	(7.24,7.72)	0.12		7.49	(7.22,7.76)	0.14		7.94	(7.72,8.15)	0.11	
	Student	7.99	(7.71,8.27)	0.14		8.31	(8.07,8.55)	0.12		8.29	(8.03,8.55)	0.13	
	Unemployed and looking for a job	6.69	(6.25,7.12)	0.22		6.96	(6.60,7.32)	0.18		7.06	(6.62,7.50)	0.22	
Religious service attendance	A few times a year	7.07	(6.87,7.27)	0.10	< .001	7.29	(7.10,7.47)	0.10	< .001	7.52	(7.34,7.70)	0.09	< .001
	More than once a week	8.14	(7.93,8.34)	0.10		8.47	(8.28,8.67)	0.10		8.60	(8.42,8.79)	0.09	
	Never	7.17	(6.97,7.38)	0.10		7.24	(7.03,7.45)	0.11		7.48	(7.31,7.64)	0.08	
	Once a week	7.55	(7.36,7.74)	0.10		7.81	(7.52,8.10)	0.15		7.96	(7.74,8.19)	0.11	

Variable	Category	Cantril's Ladder				Life Satisfaction				Happiness			
		Mean	95% CI	SE	Global p-value	Mean	95% CI	SE	Global p-value	Mean	95% CI	SE	Global p-value
	One to three times a month	7.30	(7.08,7.53)	0.11		7.58	(7.34,7.82)	0.12		7.73	(7.54,7.92)	0.09	
Education	Up to 8 years	6.53	(6.19,6.86)	0.17	< .001	7.13	(6.71,7.55)	0.21	0.015	7.05	(6.75,7.36)	0.15	< .001
	16+ years	7.43	(7.27,7.59)	0.08		7.57	(7.40,7.74)	0.09		7.84	(7.71,7.97)	0.07	
	9 to 15 years	7.45	(7.30,7.59)	0.07		7.68	(7.53,7.82)	0.07		7.81	(7.67,7.95)	0.07	
Immigration status	Born in another country	7.02	(6.80,7.24)	0.11	< .001	7.12	(6.91,7.32)	0.10	< .001	7.37	(7.19,7.55)	0.09	< .001
	Born in this country	7.49	(7.36,7.63)	0.07		7.73	(7.59,7.88)	0.08		7.91	(7.79,8.02)	0.06	
Religious affiliation	Christianity	6.64	(6.04,7.25)	0.30	< .001	7.20	(6.35,8.05)	0.42	*	7.06	(6.63,7.50)	0.21	< .001
	Islam	6.99	(6.76,7.22)	0.12		7.36	(7.11,7.60)	0.13		7.15	(6.94,7.36)	0.11	
	Judaism	7.48	(7.33,7.64)	0.08		7.65	(7.49,7.82)	0.08		7.94	(7.81,8.07)	0.07	
	No religion/Atheist/Agnostic	7.13	(6.58,7.67)	0.27		7.24	(6.73,7.75)	0.25		7.58	(6.99,8.18)	0.30	
	Primal, Animist, or Folk religion	8.00	*	*		9.00	*	*		8.00	*	*	
	Some other religion	6.98	(5.89,8.08)	0.55		7.10	(6.18,8.01)	0.46		7.10	(6.18,8.01)	0.46	
	Taoism	5.00	*	*		4.00	*	*		5.00	*	*	
	Baha'i	10.00	*	*		10.00	*	*		10.00	*	*	

Table S10f. Complete-case supplemental analysis of childhood predictors regression analysis results for Israel

		Cantril's Ladder				Life Satisfaction				Happiness			
Variable	Category	Est	95% CI	SE	Global p-value	Est	95% CI	SE	Global p-value	Est	95% CI	SE	Global p-value
Relationship with mother	(Ref: Very bad/somewhat bad)				0.190				0.427				0.931
	Very good/somewhat good	0.28	(-0.14,0.71)	0.21		0.17	(-0.24,0.57)	0.21		-0.02	(-0.37,0.33)	0.18	
Relationship with father	(Ref: Very bad/somewhat bad)				0.346				0.797				0.624
	Very good/somewhat good	0.16	(-0.17,0.49)	0.17		0.04	(-0.26,0.34)	0.15		0.07	(-0.22,0.36)	0.15	
Parent marital status	(Ref: Parents married)				0.015				0.002				0.001
	No, one or both of them had died	-0.38	(-0.83,0.08)	0.23		0.09	(-0.40,0.58)	0.25		-0.07	(-0.54,0.40)	0.24	
	No, they were never married	-0.65	(-1.34,0.04)	0.35		0.39	(-0.24,1.01)	0.32		0.27	(-0.26,0.80)	0.27	
	Yes, married	0.09	(-0.20,0.38)	0.15		0.60	(0.26,0.94)	0.17		0.37	(0.13,0.61)	0.12	
Subjective financial status of family growing up	(Ref: Got by)				0.507				0.073				0.859
	Found it difficult	-0.01	(-0.19,0.16)	0.09		0.13	(-0.05,0.30)	0.09		-0.00	(-0.16,0.16)	0.08	
	Found it very difficult	-0.26	(-0.63,0.12)	0.19		-0.36	(-0.75,0.04)	0.20		-0.09	(-0.36,0.18)	0.14	
	Lived comfortably	0.04	(-0.12,0.21)	0.08		0.13	(-0.04,0.31)	0.09		0.05	(-0.10,0.21)	0.08	
Outsider growing up	(Ref: No)				0.239				0.198				0.095
	Yes	-0.16	(-0.42,0.10)	0.13		-0.15	(-0.39,0.08)	0.12		-0.21	(-0.45,0.03)	0.12	
Self-rated health growing up	(Ref: Good)				0.006				0.011				0.014
	Excellent	0.47	(0.21,0.73)	0.13		0.51	(0.18,0.83)	0.16		0.45	(0.20,0.70)	0.13	

Variable	Category	Cantril's Ladder				Life Satisfaction				Happiness			
		Est	95% CI	SE	Global p-value	Est	95% CI	SE	Global p-value	Est	95% CI	SE	Global p-value
	Fair	0.00	(-0.44,0.44)	0.22		-0.13	(-0.52,0.26)	0.20		-0.07	(-0.43,0.28)	0.18	
	Poor	0.28	(-0.91,1.46)	0.61		0.03	(-1.08,1.14)	0.57		0.51	(-1.49,2.51)	1.02	
	Very good	0.33	(0.09,0.56)	0.12		0.42	(0.16,0.69)	0.14		0.34	(0.11,0.57)	0.12	
Immigration status	(Ref: Born in this country)				0.119				0.003				<.001
	Born in another country	-0.17	(-0.37,0.04)	0.11		-0.32	(-0.52,-0.11)	0.10		-0.35	(-0.54,-0.16)	0.10	
Age 12 religious service attendance	(Ref: Never)				0.128				0.002				0.006
	At least once a week	0.24	(0.01,0.47)	0.12		0.32	(0.09,0.55)	0.12		0.35	(0.14,0.56)	0.11	
	Less than once a month	0.16	(-0.05,0.38)	0.11		0.37	(0.18,0.57)	0.10		0.24	(0.09,0.39)	0.08	
	One to three times a month	0.28	(0.02,0.54)	0.13		0.27	(0.00,0.55)	0.14		0.26	(0.02,0.50)	0.12	
Year of birth	(Ref: 1998-2005; current age: 18-24)				<.001				<.001				<.001
	1993-1998; age 25-29	-0.16	(-0.38,0.07)	0.11		-0.13	(-0.38,0.12)	0.13		-0.19	(-0.41,0.03)	0.11	
	1983-1993; age 30-39	-0.21	(-0.48,0.06)	0.14		-0.40	(-0.65,-0.15)	0.13		-0.17	(-0.39,0.04)	0.11	
	1973-1983; age 40-49	-0.33	(-0.61,-0.05)	0.14		-0.44	(-0.71,-0.18)	0.13		-0.22	(-0.45,0.02)	0.12	
	1963-1973; age 50-59	-0.32	(-0.60,-0.03)	0.14		-0.54	(-0.82,-0.25)	0.14		-0.36	(-0.58,-0.14)	0.11	
	1953-1963; age 60-69	-0.27	(-0.56,0.03)	0.15		-0.27	(-0.55,-0.00)	0.14		-0.24	(-0.49,-0.00)	0.12	
	1943-1953; age 70-79	-0.88	(-1.21,-0.56)	0.17		-0.81	(-1.15,-0.46)	0.18		-0.70	(-0.98,-0.42)	0.14	
	1943 or earlier; age 80+	-1.27	(-1.80,-0.74)	0.27		-0.49	(-1.10,0.12)	0.31		-0.84	(-1.34,-0.35)	0.25	
Gender	(Ref: Male)				<.001				0.756				0.006
	Male	0.21	(0.09,0.34)	0.06		0.06	(-0.10,0.21)	0.08		0.11	(-0.01,0.23)	0.06	
	Other	2.09	(1.63,2.55)	0.23		-0.03	(-0.52,0.46)	0.25		-0.52	(-0.95,-0.08)	0.22	

		Cantril's Ladder				Life Satisfaction				Happiness			
Variable	Category	Est	95% CI	SE	Global p-value	Est	95% CI	SE	Global p-value	Est	95% CI	SE	Global p-value
Religious affiliation	(Ref: Judaism)				0.472				0.164				0.026
	Islam	-0.34	(-0.90,0.22)	0.29		-0.62	(-1.25,0.01)	0.32		-0.73	(-1.24,-0.21)	0.26	
	Collapsed affiliations with prevalence<3 %	-0.19	(-0.59,0.21)	0.20		-0.22	(-0.65,0.20)	0.22		-0.30	(-0.71,0.10)	0.21	
Race/ethnicity	(Ref: Plurality group)				0.202				0.616				0.213
	Non-plurality groups	-0.34	(-0.87,0.18)	0.27		0.14	(-0.40,0.67)	0.27		-0.30	(-0.76,0.17)	0.24	
Abuse	(Ref: No)												

Table S10g. Complete-case supplemental analysis of sensitivity to unmeasured confounding of childhood predictors in Israel

		Cantril's Ladder		Life Satisfaction		Happiness	
Variable	Category	E-value for Estimate	E-value for 95% CI	E-value for Estimate	E-value for 95% CI	E-value for Estimate	E-value for 95% CI
Relationship with mother	(Ref: Very bad/somewhat bad)						
	Very good/somewhat good	1.60	1.00	1.40	1.00	1.10	1.00
Relationship with father	(Ref: Very bad/somewhat bad)						
	Very good/somewhat good	1.40	1.00	1.16	1.00	1.25	1.00
Parent marital status	(Ref: Parents married)						
	No, one or both of them had died	1.74	1.00	1.26	1.00	1.25	1.00
	No, they were never married	2.18	1.00	1.74	1.00	1.60	1.00
	Yes, married	1.28	1.00	2.06	1.54	1.77	1.36
Subjective financial status of family growing up	(Ref: Got by)						
	Found it difficult	1.10	1.00	1.34	1.00	1.04	1.00
	Found it very difficult	1.55	1.00	1.69	1.00	1.29	1.00
	Lived comfortably	1.18	1.00	1.34	1.00	1.21	1.00
Outsider growing up	(Ref: No)						
	Yes	1.40	1.00	1.38	1.00	1.50	1.00
Self-rated health growing up	(Ref: Good)						
	Excellent	1.89	1.49	1.91	1.43	1.90	1.48
	Fair	1.02	1.00	1.34	1.00	1.25	1.00
	Poor	1.59	1.00	1.14	1.00	2.01	1.00
	Very good	1.66	1.29	1.79	1.39	1.72	1.33
Immigration status	(Ref: Born in this country)						
	Born in another country	1.41	1.00	1.63	1.31	1.74	1.41
Age 12 religious service attendance	(Ref: Never)						
	At least once a week	1.53	1.10	1.63	1.26	1.73	1.38
	Less than once a month	1.40	1.00	1.71	1.41	1.55	1.29
	One to three times a month	1.59	1.12	1.57	1.05	1.59	1.13
Year of birth	(Ref: 1998-2005; current age: 18-24)						
	1993-1998; age 25-29	1.40	1.00	1.34	1.00	1.47	1.00
	1983-1993; age 30-39	1.48	1.00	1.75	1.37	1.44	1.00

Variable	Category	Cantril's Ladder		Life Satisfaction		Happiness	
		E-value for Estimate	E-value for 95% CI	E-value for Estimate	E-value for 95% CI	E-value for Estimate	E-value for 95% CI
	1973-1983; age 40-49	1.66	1.19	1.82	1.42	1.51	1.00
	1963-1973; age 50-59	1.65	1.16	1.95	1.53	1.75	1.38
	1953-1963; age 60-69	1.57	1.00	1.57	1.02	1.56	1.05
	1943-1953; age 70-79	2.58	2.03	2.38	1.84	2.34	1.86
	1943 or earlier; age 80+	3.33	2.32	1.88	1.00	2.61	1.74
Gender	(Ref: Male)						
	Male	1.49	1.28	1.20	1.00	1.32	1.00
	Other	5.53	4.20	1.15	1.00	2.01	1.26
Religious affiliation	(Ref: Judaism)						
	Islam	1.69	1.00	2.08	1.00	2.39	1.51
	Collapsed affiliations with prevalence<3%	1.45	1.00	1.49	1.00	1.66	1.00
Race/ethnicity	(Ref: Plurality group)						
	Non-plurality groups	1.69	1.00	1.35	1.00	1.65	1.00
Abuse	(Ref: No)						

Tables S11a-g: Japan

Table S11a. Nationally representative descriptive statistics for Japan

Characteristic	N = 20,543[1]
**Age group**	
1998-2005; age 18-24	1,589 (7.7%)
1993-1998; age 25-29	806 (3.9%)
1983-1993; age 30-39	2,851 (14%)
1973-1983; age 40-49	3,363 (16%)
1963-1973; age 50-59	3,770 (18%)
1953-1963; age 60-69	4,118 (20%)
1943-1953; age 70-79	3,554 (17%)
1943 or earlier; age 80+	493 (2.4%)
(Missing)	0 (0%)
**Gender**	
Male	9,847 (48%)
Female	10,602 (52%)
Other	28 (0.1%)
(Missing)	66 (0.3%)
**Respondent Marital status**	
Married	11,837 (58%)
Separated	190 (0.9%)
Divorced	2,126 (10%)
Widowed	1,179 (5.7%)
Single, never married	5,004 (24%)
Domestic Partner	144 (0.7%)
(Missing)	64 (0.3%)
**Employment**	
Employed for an employer	10,853 (53%)
Self-employed	1,748 (8.5%)
Retired	2,535 (12%)
Student	491 (2.4%)
Homemaker	1,276 (6.2%)
Unemployed and looking for a job	622 (3.0%)
None of these/Other	2,983 (15%)
(Missing)	36 (0.2%)
**Religious service attendance as an adult (now)**	
More than 1/week	316 (1.5%)
1/week	348 (1.7%)
1-3/month	862 (4.2%)
A few times a year	3,112 (15%)
Never	15,788 (77%)
(Missing)	117 (0.6%)
**Education (years)**	
Up to 8 years	567 (2.8%)
9-15 years	14,893 (72%)
16+ years	5,083 (25%)
(Missing)	0 (0%)
**Immigration status**	
Born in this country	19,548 (95%)
Born in another country	158 (0.8%)
(Missing)	837 (4.1%)
**Religious affiliation as an adult (now)**	
Christianity	381 (1.9%)
Islam	10 (<0.1%)
Hinduism	5 (<0.1%)
Buddhism	6,709 (33%)
Judaism	10 (<0.1%)
Sikhism	6 (<0.1%)
Baha'i	2 (<0.1%)
Jainism	11 (<0.1%)
Shinto	469 (2.3%)
Taoism	7 (<0.1%)
Confucianism	17 (<0.1%)
Primal, Animist, or Folk religion	19 (<0.1%)

Characteristic	N = 20,543[1]
Spiritism	0 (0%)
Umbanda, Candomble, and other African-derived religions	0 (0%)
Chinese folk/traditional religion	0 (0%)
Some other religion	46 (0.2%)
No religion/Atheist/Agnostic	12,497 (61%)
(Missing)	355 (1.7%)
**Relationship with mother growing up**	
Very good	5,630 (27%)
Somewhat good	9,461 (46%)
Somewhat bad	2,750 (13%)
Very bad	799 (3.9%)
Does not apply	1,838 (8.9%)
(Missing)	66 (0.3%)
**Relationship with father growing up**	
Very good	4,156 (20%)
Somewhat good	9,081 (44%)
Somewhat bad	3,446 (17%)
Very bad	1,223 (6.0%)
Does not apply	2,580 (13%)
(Missing)	57 (0.3%)
**Parent marital status at age 12**	
Parents married	17,713 (86%)
Divorced	1,127 (5.5%)
Parents were never married	591 (2.9%)
One or both parents had died	754 (3.7%)
(Missing)	359 (1.7%)
**Subjective financial status of family growing up**	
Lived comfortably	8,320 (41%)
Got by	8,799 (43%)
Found it difficult	2,398 (12%)
Found it very difficult	973 (4.7%)
(Missing)	52 (0.3%)
**Abuse**	
Yes	1,482 (7.2%)
No	18,964 (92%)
(Missing)	96 (0.5%)
**Outsider growing up**	
Yes	1,963 (9.6%)
No	17,136 (83%)
(Missing)	1,444 (7.0%)
**Self-rated health growing up**	
Excellent	2,711 (13%)
Very good	7,106 (35%)
Good	6,689 (33%)
Fair	3,199 (16%)
Poor	758 (3.7%)
(Missing)	80 (0.4%)
**Age 12 religious service attendance**	
At least 1/week	398 (1.9%)
1-3/month	883 (4.3%)
<1/month	5,023 (24%)
Never	14,117 (69%)
(Missing)	123 (0.6%)
**Religious affiliation at age 12**	
Christianity	343 (1.7%)
Islam	7 (<0.1%)
Hinduism	4 (<0.1%)
Buddhism	6,536 (32%)
Judaism	0 (0%)
Sikhism	0 (0%)
Baha'i	7 (<0.1%)
Jainism	1 (<0.1%)
Shinto	382 (1.9%)
Taoism	14 (<0.1%)
Confucianism	25 (0.1%)

Characteristic	N = 20,543[1]
Primal, Animist, or Folk religion	13 (<0.1%)
Spiritism	0 (0%)
Umbanda, Candomble, and other African-derived religions	0 (0%)
Chinese folk/traditional religion	0 (0%)
Some other religion	46 (0.2%)
No religion/Atheist/Agnostic	12,950 (63%)
(Missing)	215 (1.0%)

[1]n (%)

Table S11b. Means by demographic category for Japan

Variable	Category	Cantril's Ladder				Life Satisfaction				Happiness			
		Mean	95% CI	SE	Global p-value	Mean	95% CI	SE	Global p-value	Mean	95% CI	SE	Global p-value
Age group	18-24	5.57	(5.44,5.70)	0.07	< .001	5.75	(5.60,5.89)	0.07	< .001	5.98	(5.85,6.11)	0.07	< .001
	25-29	5.57	(5.40,5.73)	0.08		5.63	(5.45,5.81)	0.09		5.94	(5.78,6.11)	0.08	
	30-39	5.49	(5.40,5.59)	0.05		5.64	(5.54,5.74)	0.05		5.90	(5.80,5.99)	0.05	
	40-49	5.49	(5.40,5.58)	0.05		5.58	(5.48,5.67)	0.05		5.80	(5.71,5.89)	0.05	
	50-59	5.68	(5.60,5.75)	0.04		5.72	(5.63,5.80)	0.04		5.90	(5.82,5.97)	0.04	
	60-69	6.21	(6.14,6.28)	0.04		6.36	(6.29,6.44)	0.04		6.46	(6.39,6.53)	0.04	
	70-79	6.70	(6.63,6.77)	0.04		6.93	(6.86,7.00)	0.04		6.96	(6.90,7.03)	0.03	
	80 or older	6.95	(6.74,7.15)	0.10		7.20	(6.98,7.42)	0.11		7.14	(6.94,7.34)	0.10	
Gender	Male	5.72	(5.67,5.77)	0.02	< .001	5.82	(5.77,5.87)	0.03	< .001	5.99	(5.94,6.04)	0.02	< .001
	Female	6.11	(6.06,6.16)	0.02		6.27	(6.22,6.33)	0.03		6.43	(6.38,6.48)	0.02	
	Other	5.63	(4.82,6.44)	0.39		5.92	(4.95,6.89)	0.47		5.56	(4.54,6.59)	0.49	
Marital status	Married	6.31	(6.27,6.36)	0.02	< .001	6.47	(6.43,6.51)	0.02	< .001	6.60	(6.56,6.64)	0.02	< .001
	Separated	5.70	(5.24,6.17)	0.24		5.71	(5.24,6.19)	0.24		5.84	(5.40,6.29)	0.22	
	Divorced	5.53	(5.41,5.66)	0.06		5.69	(5.55,5.83)	0.07		5.83	(5.71,5.96)	0.06	
	Widowed	6.54	(6.38,6.70)	0.08		6.77	(6.61,6.94)	0.08		6.85	(6.70,7.00)	0.08	
	Never	5.04	(4.98,5.11)	0.03		5.09	(5.02,5.16)	0.03		5.35	(5.29,5.41)	0.03	
	Domestic Partner	5.37	(4.92,5.81)	0.22		5.66	(5.13,6.19)	0.27		6.07	(5.60,6.55)	0.24	
Employment	Employed for an employer	5.75	(5.70,5.80)	0.03	< .001	5.87	(5.82,5.93)	0.03	< .001	6.05	(6.00,6.10)	0.03	< .001
	Self-employed	5.92	(5.80,6.04)	0.06		5.99	(5.85,6.12)	0.07		6.27	(6.15,6.40)	0.06	
	Retired	6.49	(6.40,6.57)	0.04		6.69	(6.61,6.78)	0.04		6.72	(6.64,6.80)	0.04	
	Student	6.08	(5.90,6.26)	0.09		6.25	(6.04,6.45)	0.10		6.29	(6.11,6.48)	0.09	
	Homemaker	6.39	(6.27,6.50)	0.06		6.54	(6.42,6.66)	0.06		6.68	(6.56,6.79)	0.06	
	Unemployed and looking for a job	4.24	(4.06,4.41)	0.09		4.27	(4.08,4.46)	0.10		4.65	(4.47,4.83)	0.09	
	None of these/Other	6.21	(6.13,6.29)	0.04		6.36	(6.28,6.44)	0.04		6.48	(6.41,6.56)	0.04	
Religious service attendance	More than 1/week	6.91	(6.66,7.16)	0.13	< .001	7.07	(6.78,7.36)	0.15	< .001	7.13	(6.85,7.40)	0.14	< .001
	1/week	6.94	(6.71,7.17)	0.12		7.05	(6.80,7.30)	0.13		7.14	(6.90,7.37)	0.12	
	1-3/month	6.15	(5.99,6.31)	0.08		6.19	(6.03,6.36)	0.09		6.38	(6.22,6.54)	0.08	
	A few times a year	6.10	(6.02,6.18)	0.04		6.24	(6.15,6.33)	0.04		6.40	(6.32,6.48)	0.04	
	Never	5.83	(5.80,5.87)	0.02		5.97	(5.93,6.01)	0.02		6.13	(6.10,6.17)	0.02	
Education	Up to 8 years	5.18	(4.94,5.43)	0.12	< .001	5.28	(5.03,5.54)	0.13	< .001	5.58	(5.35,5.82)	0.12	< .001
	9-15 years	5.80	(5.76,5.84)	0.02		5.95	(5.90,5.99)	0.02		6.12	(6.08,6.16)	0.02	

		Cantril's Ladder				Life Satisfaction				Happiness			
Variable	Category	Mean	95% CI	SE	Global p-value	Mean	95% CI	SE	Global p-value	Mean	95% CI	SE	Global p-value
	16+ years	6.37	(6.30,6.43)	0.03		6.47	(6.40,6.54)	0.04		6.59	(6.52,6.65)	0.03	
Immigration status	Born in this country	5.92	(5.89,5.96)	0.02	0.594	6.06	(6.02,6.09)	0.02	0.414	6.22	(6.18,6.25)	0.02	0.474
	Born in another country	6.02	(5.63,6.40)	0.19		6.20	(5.81,6.59)	0.20		6.10	(5.75,6.45)	0.18	
Religious affiliation	Christianity	6.69	(6.46,6.93)	0.12	< .001	6.79	(6.54,7.04)	0.13	< .001	7.00	(6.77,7.23)	0.12	< .001
	Islam	6.05	*	*		5.41	*	*		6.01	*	*	
	Hinduism	6.07	*	*		8.62	*	*		6.37	*	*	
	Buddhism	6.24	(6.18,6.30)	0.03		6.38	(6.32,6.44)	0.03		6.49	(6.44,6.55)	0.03	
	Judaism	6.88	*	*		5.75	*	*		6.74	*	*	
	Sikhism	6.65	*	*		6.00	*	*		6.67	*	*	
	Baha'i	5.25	*	*		4.68	*	*		5.00	*	*	
	Jainism	6.57	(5.52,7.61)	0.51		8.27	(6.97,9.57)	0.64		6.39	(4.63,8.16)	0.87	
	Shinto	6.15	(5.92,6.37)	0.11		6.20	(5.95,6.44)	0.13		6.40	(6.18,6.63)	0.11	
	Taoism	6.90	*	*		7.12	*	*		7.30	*	*	
	Confucianism	7.10	(4.93,9.28)	0.42		6.53	(3.59,9.48)	0.53		6.55	(3.34,9.77)	0.55	
	Primal, Animist, or Folk religion	6.68	(4.96,8.39)	0.54		6.99	(5.13,8.84)	0.57		6.80	(5.03,8.56)	0.55	
	Some other religion	4.94	(3.82,6.06)	0.55		5.07	(3.88,6.26)	0.58		5.96	(5.25,6.67)	0.35	
	No religion/Atheist /Agnostic	5.72	(5.68,5.76)	0.02		5.86	(5.81,5.90)	0.02		6.04	(5.99,6.08)	0.02	

Table S11c. Childhood predictors regression analysis results for Japan

Variable	Category	Cantril's Ladder				Life Satisfaction				Happiness			
		Est	95% CI	SE	Global p-value	Est	95% CI	SE	Global p-value	Est	95% CI	SE	Global p-value
Relationship with mother	(Ref: Very bad/somewhat bad)				0.174				0.022				<.001
	Very good/somewhat good	0.06	(-0.03, 0.16)	0.05		0.12	(0.01, 0.23)	0.05		0.22	(0.12, 0.31)	0.05	
Relationship with father	(Ref: Very bad/somewhat bad)				<.001				<.001				<.001
	Very good/somewhat good	0.39	(0.31, 0.48)	0.04		0.41	(0.32, 0.51)	0.05		0.33	(0.25, 0.42)	0.04	
Parent marital status	(Ref: Parents married)				0.148				0.067				0.003
	Divorced	0.15	(-0.01, 0.30)	0.08		0.17	(0.00, 0.35)	0.09		0.27	(0.11, 0.43)	0.08	
	Parents were never married	-0.03	(-0.22, 0.17)	0.10		-0.05	(-0.26, 0.16)	0.11		-0.11	(-0.30, 0.08)	0.10	
	One or both parents had died	0.12	(-0.07, 0.31)	0.09		0.17	(-0.02, 0.35)	0.10		0.10	(-0.08, 0.28)	0.09	
Subjective financial status of family growing up	(Ref: Got by)				<.001				<.001				<.001
	Lived comfortably	0.44	(0.37, 0.52)	0.04		0.44	(0.36, 0.51)	0.04		0.42	(0.35, 0.49)	0.04	
	Found it difficult	-0.22	(-0.32, -0.11)	0.06		-0.21	(-0.32, -0.09)	0.06		-0.17	(-0.27, -0.06)	0.05	
	Found it very difficult	-0.69	(-0.88, -0.51)	0.10		-0.70	(-0.90, -0.49)	0.10		-0.57	(-0.77, -0.38)	0.10	
Abuse	(Ref: No)				<.001				0.002				0.008
	Yes	-0.25	(-0.40, -0.10)	0.07		-0.26	(-0.42, -0.10)	0.08		-0.20	(-0.35, -0.05)	0.08	
Outsider growing up	(Ref: No)				0.307				0.344				0.023
	Yes	-0.06	(-0.20, 0.07)	0.07		-0.06	(-0.20, 0.08)	0.07		-0.14	(-0.28, -0.01)	0.07	
Self-rated health growing up	(Ref: Good)				<.001				<.001				<.001
	Excellent	0.98	(0.86, 1.09)	0.06		1.10	(0.98, 1.22)	0.06		1.10	(0.99, 1.21)	0.06	

		Cantril's Ladder				Life Satisfaction				Happiness			
Variable	Category	Est	95% CI	SE	Global p-value	Est	95% CI	SE	Global p-value	Est	95% CI	SE	Global p-value
	Very good	0.46	(0.39, 0.54)	0.04		0.51	(0.43, 0.59)	0.04		0.50	(0.43, 0.57)	0.04	
	Fair	-0.47	(-0.57, -0.38)	0.05		-0.51	(-0.61, -0.40)	0.05		-0.47	(-0.57, -0.37)	0.05	
	Poor	-0.91	(-1.12, -0.70)	0.11		-0.95	(-1.18, -0.72)	0.12		-0.85	(-1.06, -0.64)	0.11	
Immigration status	(Ref: Born in this country)				0.027				0.016				0.245
	Born in another country	0.39	(0.02, 0.77)	0.19		0.42	(0.06, 0.78)	0.18		0.20	(-0.16, 0.57)	0.19	
Age 12 religious service attendance	(Ref: Never)				<.001				<.001				<.001
	At least 1/week	0.32	(0.07, 0.57)	0.13		0.26	(-0.01, 0.53)	0.14		0.36	(0.13, 0.59)	0.12	
	1-3/month	0.60	(0.45, 0.75)	0.08		0.54	(0.38, 0.70)	0.08		0.56	(0.41, 0.71)	0.08	
	< 1/month	0.13	(0.06, 0.21)	0.04		0.14	(0.06, 0.22)	0.04		0.17	(0.09, 0.24)	0.04	
Year of birth	(Ref: 1998-2005; current age: 18-24)				<.001				<.001				<.001
	1993-1998; age 25-29	0.06	(-0.13, 0.25)	0.10		-0.07	(-0.28, 0.14)	0.11		0.01	(-0.18, 0.20)	0.10	
	1983-1993; age 30-39	0.02	(-0.13, 0.17)	0.08		-0.00	(-0.16, 0.16)	0.08		0.02	(-0.13, 0.17)	0.08	
	1973-1983; age 40-49	-0.01	(-0.16, 0.14)	0.08		-0.09	(-0.25, 0.07)	0.08		-0.11	(-0.26, 0.03)	0.08	
	1963-1973; age 50-59	0.21	(0.07, 0.35)	0.07		0.08	(-0.07, 0.24)	0.08		0.01	(-0.13, 0.15)	0.07	
	1953-1963; age 60-69	0.65	(0.51, 0.78)	0.07		0.63	(0.47, 0.78)	0.08		0.48	(0.34, 0.61)	0.07	
	1943-1953; age 70-79	1.07	(0.93, 1.21)	0.07		1.12	(0.97, 1.27)	0.08		0.91	(0.77, 1.04)	0.07	
	1943 or earlier; age 80+	1.37	(1.13, 1.60)	0.12		1.45	(1.19, 1.70)	0.13		1.15	(0.92, 1.38)	0.12	
Gender	(Ref: Male)				<.001				<.001				<.001
	Female	0.32	(0.26, 0.38)	0.03		0.38	(0.32, 0.45)	0.03		0.38	(0.32, 0.44)	0.03	
	Other	0.79	(0.01, 1.57)	0.40		1.09	(0.13, 2.05)	0.49		0.42	(-0.60, 1.43)	0.52	
Religious affiliation	(Ref: No religion/Atheist /Agnostic)				<.001				<.001				0.009
	Buddhism	0.16	(0.09, 0.23)	0.04		0.14	(0.06, 0.22)	0.04		0.10	(0.03, 0.17)	0.04	

Variable	Category	Cantril's Ladder				Life Satisfaction				Happiness			
		Est	95% CI	SE	Global p-value	Est	95% CI	SE	Global p-value	Est	95% CI	SE	Global p-value
Race/ethnicity	Collapsed affiliations with prevalence<3% (Ref: Plurality group)	0.21	(0.05, 0.37)	0.08		0.15	(-0.03, 0.33)	0.09		0.12	(-0.04, 0.28)	0.08	

Table S11d. Sensitivity to unmeasured confounding of childhood predictors in Japan

		Cantril's Ladder		Life Satisfaction		Happiness	
Variable	Category	E-value for Estimate	E-value for 95% CI	E-value for Estimate	E-value for 95% CI	E-value for Estimate	E-value for 95% CI
Relationship with mother	(Ref: Very bad/somewhat bad)						
	Very good/somewhat good	1.20	1.00	1.27	1.07	1.42	1.29
Relationship with father	(Ref: Very bad/somewhat bad)						
	Very good/somewhat good	1.64	1.54	1.63	1.52	1.57	1.46
Parent marital status	(Ref: Parents married)						
	Divorced	1.33	1.00	1.35	1.04	1.50	1.28
	Parents were never married	1.12	1.00	1.16	1.00	1.27	1.00
	One or both parents had died	1.29	1.00	1.34	1.00	1.26	1.00
Subjective financial status of family growing up	(Ref: Got by)						
	Lived comfortably	1.71	1.62	1.66	1.57	1.68	1.60
	Found it difficult	1.42	1.27	1.39	1.24	1.35	1.19
	Found it very difficult	2.02	1.78	1.96	1.72	1.87	1.63
Abuse	(Ref: No)						
	Yes	1.47	1.26	1.45	1.24	1.40	1.17
Outsider growing up	(Ref: No)						
	Yes	1.20	1.00	1.19	1.00	1.32	1.08
Self-rated health growing up	(Ref: Good)						
	Excellent	2.40	2.24	2.46	2.30	2.57	2.41
	Very good	1.73	1.64	1.75	1.66	1.78	1.69
	Fair	1.74	1.62	1.74	1.62	1.74	1.62
	Poor	2.30	2.02	2.26	1.99	2.23	1.95
Immigration status	(Ref: Born in this country)						
	Born in another country	1.65	1.11	1.64	1.18	1.41	1.00
Age 12 religious service attendance	(Ref: Never)						
	At least 1/week	1.56	1.21	1.45	1.00	1.61	1.30
	1-3/month	1.90	1.71	1.78	1.59	1.85	1.66
	< 1/month	1.30	1.19	1.30	1.19	1.35	1.25
Year of birth	(Ref: 1998-2005; current age: 18-24)						
	1993-1998; age 25-29	1.19	1.00	1.19	1.00	1.07	1.00
	1983-1993; age 30-39	1.11	1.00	1.01	1.00	1.10	1.00
	1973-1983; age 40-49	1.08	1.00	1.23	1.00	1.28	1.00
	1963-1973; age 50-59	1.41	1.20	1.22	1.00	1.08	1.00

Variable	Category	Cantril's Ladder		Life Satisfaction		Happiness	
		E-value for Estimate	E-value for 95% CI	E-value for Estimate	E-value for 95% CI	E-value for Estimate	E-value for 95% CI
	1953-1963; age 60-69	1.96	1.79	1.88	1.70	1.75	1.58
	1943-1953; age 70-79	2.53	2.33	2.48	2.29	2.31	2.12
	1943 or earlier; age 80+	2.97	2.62	2.93	2.58	2.65	2.32
Gender	(Ref: Male)						
	Female	1.55	1.47	1.60	1.52	1.63	1.55
	Other	2.15	1.07	2.44	1.29	1.68	1.00
Religious affiliation	(Ref: No religion/Atheist/Agnostic)						
	Buddhism	1.34	1.23	1.30	1.18	1.26	1.14
	Collapsed affiliations with prevalence<3%	1.41	1.17	1.31	1.00	1.29	1.00
Race/ethnicity	(Ref: Plurality group)						

Table S11e. Complete-case supplemental analysis of means by demographic category for Japan

		Cantril's Ladder				Life Satisfaction				Happiness			
Variable	Category	Mean	95% CI	SE	Global p-value	Mean	95% CI	SE	Global p-value	Mean	95% CI	SE	Global p-value
Age group	18-24	5.77	(5.64,5.89)	0.06	< .001	6.03	(5.90,6.15)	0.06	< .001	6.16	(6.04,6.29)	0.06	< .001
	25-29	5.74	(5.58,5.89)	0.08		5.93	(5.77,6.09)	0.08		6.10	(5.94,6.26)	0.08	
	30-39	5.65	(5.56,5.74)	0.05		5.91	(5.82,6.01)	0.05		6.07	(5.98,6.16)	0.05	
	40-49	5.68	(5.59,5.76)	0.04		5.85	(5.76,5.94)	0.04		5.97	(5.88,6.06)	0.04	
	50-59	5.82	(5.75,5.89)	0.04		5.95	(5.88,6.03)	0.04		6.04	(5.97,6.12)	0.04	
	60-69	6.29	(6.23,6.36)	0.03		6.49	(6.42,6.56)	0.04		6.53	(6.47,6.60)	0.03	
	70-79	6.73	(6.67,6.80)	0.03		6.98	(6.92,7.05)	0.03		6.99	(6.93,7.05)	0.03	
	80 or older	7.00	(6.80,7.19)	0.10		7.24	(7.03,7.45)	0.11		7.15	(6.95,7.35)	0.10	
Gender	Female	6.22	(6.18,6.27)	0.02	< .001	6.46	(6.41,6.51)	0.02	< .001	6.53	(6.48,6.57)	0.02	< .001
	Male	5.87	(5.82,5.91)	0.02		6.04	(6.00,6.09)	0.02		6.14	(6.09,6.18)	0.02	
	Other	5.87	(5.11,6.64)	0.37		6.20	(5.26,7.14)	0.45		5.97	(5.04,6.90)	0.45	
Marital status	Divorced	5.70	(5.58,5.81)	0.06	< .001	5.95	(5.83,6.08)	0.06	< .001	5.98	(5.87,6.10)	0.06	< .001
	Domestic partner	5.56	(5.14,5.97)	0.21		6.06	(5.58,6.54)	0.24		6.19	(5.72,6.66)	0.24	
	Married	6.38	(6.34,6.42)	0.02		6.58	(6.53,6.62)	0.02		6.66	(6.62,6.70)	0.02	
	Separated	5.96	(5.54,6.39)	0.22		6.01	(5.58,6.44)	0.22		6.06	(5.66,6.45)	0.20	
	Single/Never been married	5.27	(5.21,5.33)	0.03		5.47	(5.41,5.53)	0.03		5.59	(5.53,5.65)	0.03	
	Widowed	6.62	(6.47,6.77)	0.08		6.83	(6.68,6.99)	0.08		6.89	(6.75,7.04)	0.07	
Employment	Employed for an employer	5.87	(5.82,5.92)	0.02	< .001	6.07	(6.02,6.11)	0.02	< .001	6.17	(6.12,6.22)	0.02	< .001
	Homemaker	6.49	(6.38,6.59)	0.05		6.70	(6.59,6.82)	0.06		6.75	(6.64,6.86)	0.05	
	None of these/Other	6.34	(6.26,6.41)	0.04		6.56	(6.49,6.64)	0.04		6.59	(6.52,6.66)	0.04	
	Retired	6.53	(6.45,6.61)	0.04		6.76	(6.68,6.84)	0.04		6.74	(6.67,6.82)	0.04	
	Self-employed	6.07	(5.96,6.18)	0.06		6.23	(6.11,6.35)	0.06		6.42	(6.30,6.53)	0.06	
	Student	6.19	(6.03,6.35)	0.08		6.45	(6.27,6.62)	0.09		6.44	(6.28,6.60)	0.08	
	Unemployed and looking for a job	4.70	(4.54,4.86)	0.08		4.99	(4.82,5.16)	0.09		5.15	(4.99,5.30)	0.08	
Religious service attendance	A few times a year	6.17	(6.09,6.25)	0.04	< .001	6.35	(6.27,6.43)	0.04	< .001	6.45	(6.38,6.53)	0.04	< .001
	More than once a week	6.94	(6.70,7.18)	0.12		7.24	(6.98,7.49)	0.13		7.20	(6.94,7.45)	0.13	
	Never	5.98	(5.94,6.01)	0.02		6.20	(6.16,6.24)	0.02		6.27	(6.24,6.31)	0.02	
	Once a week	6.98	(6.75,7.21)	0.12		7.12	(6.88,7.37)	0.13		7.19	(6.97,7.41)	0.11	

		Cantril's Ladder				Life Satisfaction				Happiness			
Variable	Category	Mean	95% CI	SE	Global p-value	Mean	95% CI	SE	Global p-value	Mean	95% CI	SE	Global p-value
	One to three times a month	6.28	(6.13,6.42)	0.08		6.33	(6.18,6.49)	0.08		6.47	(6.32,6.62)	0.08	
Education	Up to 8 years	5.51	(5.28,5.74)	0.12	< .001	5.73	(5.49,5.97)	0.12	< .001	5.87	(5.65,6.09)	0.11	< .001
	16+ years	6.45	(6.39,6.51)	0.03		6.62	(6.56,6.69)	0.03		6.68	(6.62,6.74)	0.03	
	9 to 15 years	5.93	(5.90,5.97)	0.02		6.16	(6.12,6.19)	0.02		6.24	(6.20,6.28)	0.02	
Immigration status	Born in another country	6.19	(5.83,6.55)	0.18	0.435	6.33	(5.97,6.70)	0.18	0.680	6.23	(5.91,6.55)	0.16	0.499
	Born in this country	6.05	(6.02,6.08)	0.02		6.26	(6.23,6.29)	0.02		6.34	(6.31,6.37)	0.02	
Religious affiliation	Buddhism	6.33	(6.28,6.38)	0.03	< .001	6.53	(6.48,6.59)	0.03	< .001	6.57	(6.52,6.62)	0.03	< .001
	Christianity	6.77	(6.54,6.99)	0.11		6.92	(6.69,7.16)	0.12		7.05	(6.83,7.28)	0.11	
	Confucianism	7.10	(4.62,9.59)	0.43		6.71	(3.68,9.75)	0.52		6.55	(3.34,9.77)	0.55	
	Hinduism	6.31	*	*		8.62	*	*		6.35	*	*	
	Islam	6.44	*	*		5.90	*	*		6.92	*	*	
	Judaism	7.26	*	*		5.88	*	*		6.74	*	*	
	No religion/Atheist/Agnostic	5.87	(5.83,5.91)	0.02		6.09	(6.04,6.13)	0.02		6.19	(6.14,6.23)	0.02	
	Primal, Animist, or Folk religion	6.68	(4.89,8.47)	0.55		6.99	(5.13,8.84)	0.57		6.81	(5.01,8.61)	0.55	
	Sikhism	6.89	*	*		6.21	*	*		6.65	*	*	
	Some other religion	5.75	(4.96,6.54)	0.39		6.13	(5.32,6.94)	0.40		6.06	(5.36,6.75)	0.34	
	Taoism	6.91	*	*		7.12	*	*		7.36	*	*	
	Baha'i	5.68	*	*		4.68	*	*		5.00	*	*	
	Jainism	6.57	(5.52,7.61)	0.51		8.27	(6.97,9.57)	0.64		6.39	(4.63,8.16)	0.87	
	Shinto	6.22	(6.01,6.43)	0.11		6.38	(6.15,6.61)	0.12		6.52	(6.31,6.73)	0.11	

Table S11f. Complete-case supplemental analysis of childhood predictors regression analysis results for Japan

		Cantril's Ladder				Life Satisfaction				Happiness			
Variable	Category	Est	95% CI	SE	Global p-value	Est	95% CI	SE	Global p-value	Est	95% CI	SE	Global p-value
Relationship with mother	(Ref: Very bad/somewhat bad)				0.549				0.051				<.001
	Very good/somewhat good	0.03	(-0.06,0.12)	0.05		0.09	(-0.00,0.19)	0.05		0.19	(0.10,0.28)	0.05	
Relationship with father	(Ref: Very bad/somewhat bad)				<.001				<.001				<.001
	Very good/somewhat good	0.37	(0.29,0.45)	0.04		0.35	(0.27,0.44)	0.04		0.30	(0.22,0.38)	0.04	
Parent marital status	(Ref: Parents married)				0.114				0.145				0.003
	No, one or both of them had died	-0.05	(-0.26,0.16)	0.11		0.04	(-0.18,0.26)	0.11		-0.10	(-0.31,0.11)	0.11	
	No, they were never married	-0.11	(-0.33,0.11)	0.11		-0.14	(-0.37,0.09)	0.12		-0.32	(-0.54,-0.09)	0.11	
	Yes, married	-0.16	(-0.30,-0.02)	0.07		-0.12	(-0.28,0.03)	0.08		-0.25	(-0.40,-0.10)	0.08	
Subjective financial status of family growing up	(Ref: Got by)				<.001				<.001				<.001
	Found it difficult	-0.17	(-0.27,-0.07)	0.05		-0.19	(-0.29,-0.08)	0.05		-0.15	(-0.24,-0.05)	0.05	
	Found it very difficult	-0.37	(-0.54,-0.19)	0.09		-0.30	(-0.49,-0.10)	0.10		-0.27	(-0.45,-0.09)	0.09	
	Lived comfortably	0.45	(0.38,0.52)	0.03		0.45	(0.38,0.52)	0.04		0.45	(0.38,0.51)	0.03	
Abuse	(Ref: No)				0.002				0.017				0.018
	Yes	-0.21	(-0.35,-0.08)	0.07		-0.18	(-0.32,-0.03)	0.07		-0.17	(-0.31,-0.03)	0.07	
Outsider growing up	(Ref: No)				0.174				0.321				0.766
	Yes	0.08	(-0.04,0.20)	0.06		0.06	(-0.06,0.19)	0.06		0.02	(-0.10,0.14)	0.06	

		Cantril's Ladder				Life Satisfaction				Happiness			
Variable	Category	Est	95% CI	SE	Global p-value	Est	95% CI	SE	Global p-value	Est	95% CI	SE	Global p-value
Self-rated health growing up	(Ref: Good)				<.001				<.001				<.001
	Excellent	0.98	(0.88,1.09)	0.05		1.10	(0.99,1.21)	0.06		1.10	(1.00,1.21)	0.05	
	Fair	-0.45	(-0.54,-0.36)	0.05		-0.42	(-0.52,-0.32)	0.05		-0.41	(-0.51,-0.32)	0.05	
	Poor	-0.59	(-0.79,-0.39)	0.10		-0.61	(-0.83,-0.40)	0.11		-0.53	(-0.73,-0.33)	0.10	
	Very good	0.47	(0.40,0.54)	0.04		0.49	(0.42,0.56)	0.04		0.51	(0.44,0.58)	0.04	
Immigration status	(Ref: Born in this country)				0.044				0.069				0.466
	Born in another country	0.35	(0.01,0.69)	0.17		0.31	(-0.02,0.65)	0.17		0.12	(-0.21,0.45)	0.17	
Age 12 religious service attendance	(Ref: Never)				<.001				<.001				<.001
	At least once a week	0.31	(0.08,0.55)	0.12		0.27	(0.03,0.51)	0.12		0.35	(0.13,0.57)	0.11	
	Less than once a month	0.10	(0.03,0.17)	0.04		0.11	(0.04,0.19)	0.04		0.13	(0.06,0.20)	0.04	
	One to three times a month	0.51	(0.37,0.65)	0.07		0.42	(0.27,0.57)	0.08		0.47	(0.33,0.61)	0.07	
Year of birth	(Ref: 1998-2005; current age: 18-24)				<.001				<.001				<.001
	1993-1998; age 25-29	0.04	(-0.14,0.22)	0.09		-0.03	(-0.21,0.15)	0.09		-0.01	(-0.19,0.16)	0.09	
	1983-1993; age 30-39	-0.01	(-0.15,0.13)	0.07		-0.00	(-0.15,0.14)	0.07		0.00	(-0.13,0.14)	0.07	
	1973-1983; age 40-49	-0.00	(-0.14,0.14)	0.07		-0.09	(-0.23,0.05)	0.07		-0.12	(-0.26,0.02)	0.07	
	1963-1973; age 50-59	0.18	(0.05,0.31)	0.07		0.05	(-0.09,0.19)	0.07		-0.01	(-0.14,0.12)	0.07	
	1953-1963; age 60-69	0.59	(0.46,0.72)	0.07		0.52	(0.38,0.65)	0.07		0.42	(0.29,0.54)	0.07	
	1943-1953; age 70-79	0.98	(0.85,1.11)	0.07		0.96	(0.82,1.09)	0.07		0.81	(0.69,0.94)	0.07	

		Cantril's Ladder				Life Satisfaction				Happiness			
Variable	Category	Est	95% CI	SE	Global p-value	Est	95% CI	SE	Global p-value	Est	95% CI	SE	Global p-value
	1943 or earlier; age 80+	1.28	(1.06,1.50)	0.11		1.26	(1.02,1.49)	0.12		1.02	(0.80,1.24)	0.11	
Gender	(Ref: Male)				<.001				<.001				<.001
	Male	-0.29	(-0.35,-0.23)	0.03		-0.35	(-0.41,-0.29)	0.03		-0.33	(-0.39,-0.27)	0.03	
	Other	0.45	(-0.30,1.21)	0.38		0.56	(-0.36,1.48)	0.47		0.23	(-0.68,1.14)	0.46	
Religious affiliation	(Ref: No religion/Atheist/Agnostic)				<.001				<.001				0.019
	Buddhism	0.14	(0.08,0.21)	0.03		0.15	(0.08,0.22)	0.03		0.09	(0.03,0.16)	0.03	
	Collapsed affiliations with prevalence<3%	0.18	(0.02,0.34)	0.08		0.14	(-0.03,0.31)	0.09		0.09	(-0.06,0.25)	0.08	
Race/ethnicity	(Ref: Plurality group)												

Table S11g. Complete-case supplemental analysis of sensitivity to unmeasured confounding of childhood predictors in Japan

		Cantril's Ladder		Life Satisfaction		Happiness	
Variable	Category	E-value for Estimate	E-value for 95% CI	E-value for Estimate	E-value for 95% CI	E-value for Estimate	E-value for 95% CI
Relationship with mother	(Ref: Very bad/somewhat bad)						
	Very good/somewhat good	1.12	1.00	1.25	1.00	1.40	1.27
Relationship with father	(Ref: Very bad/somewhat bad)						
	Very good/somewhat good	1.66	1.55	1.61	1.50	1.57	1.46
Parent marital status	(Ref: Parents married)						
	No, one or both of them had died	1.18	1.00	1.15	1.00	1.27	1.00
	No, they were never married	1.29	1.00	1.32	1.00	1.58	1.26
	Yes, married	1.36	1.10	1.30	1.00	1.50	1.28
Subjective financial status of family growing up	(Ref: Got by)						
	Found it difficult	1.38	1.22	1.39	1.24	1.34	1.17
	Found it very difficult	1.65	1.41	1.54	1.27	1.52	1.25
	Lived comfortably	1.76	1.67	1.74	1.65	1.76	1.67
Abuse	(Ref: No)						
	Yes	1.44	1.23	1.38	1.13	1.38	1.13
Outsider growing up	(Ref: No)						
	Yes	1.24	1.00	1.20	1.00	1.10	1.00
Self-rated health growing up	(Ref: Good)						
	Excellent	2.52	2.36	2.64	2.47	2.71	2.55
	Fair	1.76	1.64	1.70	1.57	1.72	1.59
	Poor	1.95	1.68	1.95	1.67	1.87	1.60
	Very good	1.79	1.70	1.79	1.69	1.84	1.75
Immigration status	(Ref: Born in this country)						
	Born in another country	1.63	1.08	1.56	1.00	1.31	1.00
Age 12 religious service attendance	(Ref: Never)						
	At least once a week	1.58	1.23	1.50	1.13	1.63	1.32
	Less than once a month	1.27	1.13	1.28	1.15	1.32	1.20
	One to three times a month	1.84	1.65	1.70	1.51	1.79	1.60
Year of birth	(Ref: 1998-2005; current age: 18-24)						

Variable	Category	Cantril's Ladder		Life Satisfaction		Happiness	
		E-value for Estimate	E-value for 95% CI	E-value for Estimate	E-value for 95% CI	E-value for Estimate	E-value for 95% CI
	1993-1998; age 25-29	1.15	1.00	1.13	1.00	1.08	1.00
	1983-1993; age 30-39	1.07	1.00	1.04	1.00	1.04	1.00
	1973-1983; age 40-49	1.02	1.00	1.25	1.00	1.30	1.00
	1963-1973; age 50-59	1.39	1.18	1.17	1.00	1.05	1.00
	1953-1963; age 60-69	1.95	1.78	1.83	1.65	1.72	1.55
	1943-1953; age 70-79	2.51	2.31	2.43	2.23	2.27	2.09
	1943 or earlier; age 80+	3.00	2.63	2.88	2.52	2.59	2.25
Gender	(Ref: Male)						
	Male	1.54	1.46	1.61	1.53	1.60	1.52
	Other	1.76	1.00	1.88	1.00	1.46	1.00
Religious affiliation	(Ref: No religion/Atheist/Agnostic)						
	Buddhism	1.34	1.23	1.34	1.23	1.26	1.12
	Collapsed affiliations with prevalence<3%	1.39	1.11	1.32	1.00	1.26	1.00
Race/ethnicity	(Ref: Plurality group)						

Tables S12a-g: Kenya

Table S12a. Nationally representative descriptive statistics for Kenya

Characteristic	N = 11,389[1]
**Age group**	
1998-2005; age 18-24	2,868 (25%)
1993-1998; age 25-29	2,035 (18%)
1983-1993; age 30-39	2,564 (23%)
1973-1983; age 40-49	1,708 (15%)
1963-1973; age 50-59	1,072 (9.4%)
1953-1963; age 60-69	710 (6.2%)
1943-1953; age 70-79	360 (3.2%)
1943 or earlier; age 80+	67 (0.6%)
(Missing)	5 (<0.1%)
**Gender**	
Male	5,567 (49%)
Female	5,813 (51%)
Other	2 (<0.1%)
(Missing)	7 (<0.1%)
**Race/Ethnicity**	
Embu	197 (1.7%)
Kalenjin	1,377 (12%)
Kamba	1,299 (11%)
Kenyan Somali/Somali	396 (3.5%)
Kikuyu	2,119 (19%)
Kisii	789 (6.9%)
Luhya	1,943 (17%)
Luo	1,120 (9.8%)
Maasai	237 (2.1%)
Meru	630 (5.5%)
Miji Kenda tribes	708 (6.2%)
Other	548 (4.8%)
(Missing)	27 (0.2%)
**Respondent Marital status**	
Married	6,626 (58%)
Separated	467 (4.1%)
Divorced	111 (1.0%)
Widowed	464 (4.1%)
Single, never married	3,531 (31%)
Domestic Partner	146 (1.3%)
(Missing)	43 (0.4%)
**Employment**	
Employed for an employer	1,467 (13%)
Self-employed	3,630 (32%)
Retired	319 (2.8%)
Student	1,136 (10.0%)
Homemaker	1,537 (13%)
Unemployed and looking for a job	3,153 (28%)
None of these/Other	138 (1.2%)
(Missing)	9 (<0.1%)
**Religious service attendance as an adult (now)**	
More than 1/week	2,774 (24%)
1/week	6,063 (53%)
1-3/month	1,219 (11%)
A few times a year	855 (7.5%)
Never	465 (4.1%)
(Missing)	13 (0.1%)
**Education (years)**	
Up to 8 years	4,485 (39%)
9-15 years	6,115 (54%)
16+ years	783 (6.9%)
(Missing)	6 (<0.1%)
**Immigration status**	
Born in this country	11,270 (99%)
Born in another country	117 (1.0%)

**Characteristic**	**N = 11,389**[1]
(Missing)	2 (<0.1%)
**Religious affiliation as an adult (now)**	
Christianity	10,334 (91%)
Islam	918 (8.1%)
Hinduism	0 (0%)
Buddhism	1 (<0.1%)
Judaism	3 (<0.1%)
Sikhism	0 (0%)
Baha'i	1 (<0.1%)
Jainism	1 (<0.1%)
Shinto	0 (0%)
Taoism	0 (0%)
Confucianism	3 (<0.1%)
Primal, Animist, or Folk religion	7 (<0.1%)
Spiritism	0 (0%)
Umbanda, Candomble, and other African-derived religions	0 (0%)
Chinese folk/traditional religion	0 (0%)
Some other religion	5 (<0.1%)
No religion/Atheist/Agnostic	108 (0.9%)
(Missing)	9 (<0.1%)
**Relationship with mother growing up**	
Very good	9,418 (83%)
Somewhat good	1,435 (13%)
Somewhat bad	130 (1.1%)
Very bad	100 (0.9%)
Does not apply	240 (2.1%)
(Missing)	66 (0.6%)
**Relationship with father growing up**	
Very good	7,958 (70%)
Somewhat good	1,896 (17%)
Somewhat bad	216 (1.9%)
Very bad	220 (1.9%)
Does not apply	967 (8.5%)
(Missing)	132 (1.2%)
**Parent marital status at age 12**	
Parents married	9,238 (81%)
Divorced	697 (6.1%)
Parents were never married	681 (6.0%)
One or both parents had died	471 (4.1%)
(Missing)	301 (2.6%)
**Subjective financial status of family growing up**	
Lived comfortably	3,026 (27%)
Got by	3,279 (29%)
Found it difficult	4,071 (36%)
Found it very difficult	994 (8.7%)
(Missing)	19 (0.2%)
**Abuse**	
Yes	1,300 (11%)
No	10,039 (88%)
(Missing)	49 (0.4%)
**Outsider growing up**	
Yes	1,223 (11%)
No	10,114 (89%)
(Missing)	52 (0.5%)
**Self-rated health growing up**	
Excellent	4,449 (39%)
Very good	2,598 (23%)
Good	2,582 (23%)
Fair	1,384 (12%)
Poor	349 (3.1%)
(Missing)	26 (0.2%)
**Age 12 religious service attendance**	
At least 1/week	9,189 (81%)
1-3/month	1,687 (15%)
<1/month	236 (2.1%)

**Characteristic**	**N = 11,389**[1]
Never	198 (1.7%)
(Missing)	79 (0.7%)
**Religious affiliation at age 12**	
Christianity	10,369 (91%)
Islam	916 (8.0%)
Hinduism	0 (0%)
Buddhism	5 (<0.1%)
Judaism	6 (<0.1%)
Sikhism	0 (<0.1%)
Baha'i	3 (<0.1%)
Jainism	1 (<0.1%)
Shinto	0 (0%)
Taoism	0 (0%)
Confucianism	0 (0%)
Primal, Animist, or Folk religion	13 (0.1%)
Spiritism	0 (0%)
Umbanda, Candomble, and other African-derived religions	0 (0%)
Chinese folk/traditional religion	0 (0%)
Some other religion	0 (<0.1%)
No religion/Atheist/Agnostic	67 (0.6%)
(Missing)	9 (<0.1%)

[1]n (%)

Table S12b. Means by demographic category for Kenya

		Cantril's Ladder				Life Satisfaction				Happiness			
Variable	Category	Mean	95% CI	SE	Global p-value	Mean	95% CI	SE	Global p-value	Mean	95% CI	SE	Global p-value
Age group	18-24	5.94	(5.81,6.07)	0.07	< .001	6.21	(6.06,6.36)	0.07	< .001	7.67	(7.56,7.79)	0.06	< .001
	25-29	5.67	(5.50,5.83)	0.08		6.03	(5.86,6.21)	0.09		7.32	(7.17,7.46)	0.07	
	30-39	5.38	(5.21,5.55)	0.09		5.76	(5.60,5.92)	0.08		7.18	(7.06,7.31)	0.06	
	40-49	5.06	(4.84,5.29)	0.11		5.45	(5.22,5.69)	0.12		6.88	(6.67,7.09)	0.11	
	50-59	5.30	(5.00,5.59)	0.15		5.99	(5.69,6.29)	0.15		7.03	(6.76,7.30)	0.14	
	60-69	5.28	(4.90,5.66)	0.19		6.55	(6.11,6.99)	0.23		7.15	(6.80,7.49)	0.18	
	70-79	5.37	(4.71,6.04)	0.34		6.23	(5.61,6.85)	0.32		7.25	(6.71,7.79)	0.27	
	80 or older	5.24	(3.80,6.68)	0.72		6.94	(5.66,8.22)	0.64		7.56	(6.20,8.92)	0.68	
Gender	Male	5.51	(5.39,5.64)	0.06	0.017	5.99	(5.87,6.11)	0.06	0.572	7.17	(7.07,7.27)	0.05	0.010
	Female	5.51	(5.38,5.63)	0.06		5.95	(5.82,6.07)	0.06		7.37	(7.26,7.48)	0.06	
	Other	8.85	*	*		7.99	*	*		7.99	*	*	
Marital status	Married	5.44	(5.32,5.57)	0.06	< .001	5.99	(5.87,6.11)	0.06	0.006	7.29	(7.18,7.40)	0.06	< .001
	Separated	5.23	(4.80,5.65)	0.22		5.30	(4.88,5.71)	0.21		6.98	(6.61,7.34)	0.19	
	Divorced	4.88	(4.28,5.49)	0.31		5.59	(4.90,6.27)	0.34		6.58	(5.93,7.24)	0.33	
	Widowed	4.68	(4.17,5.18)	0.26		6.18	(5.70,6.66)	0.25		6.39	(5.93,6.86)	0.24	
	Never	5.79	(5.67,5.91)	0.06		6.02	(5.89,6.15)	0.07		7.43	(7.32,7.54)	0.06	
	Domestic Partner	5.71	(5.13,6.29)	0.29		5.62	(5.03,6.21)	0.30		7.05	(6.42,7.69)	0.32	
Employment	Employed for an employer	5.69	(5.48,5.89)	0.11	< .001	5.96	(5.76,6.15)	0.10	< .001	7.12	(6.95,7.29)	0.09	< .001
	Self-employed	5.39	(5.25,5.54)	0.08		5.98	(5.83,6.14)	0.08		7.17	(7.03,7.31)	0.07	
	Retired	5.48	(4.88,6.07)	0.30		6.22	(5.63,6.80)	0.30		7.12	(6.57,7.66)	0.28	
	Student	6.03	(5.85,6.22)	0.09		6.23	(6.01,6.45)	0.11		7.58	(7.41,7.75)	0.09	
	Homemaker	5.51	(5.28,5.73)	0.11		6.29	(6.05,6.53)	0.12		7.48	(7.26,7.69)	0.11	
	Unemployed and looking for a job	5.38	(5.21,5.55)	0.09		5.69	(5.52,5.86)	0.09		7.29	(7.16,7.43)	0.07	
	None of these/Other	5.35	(4.30,6.39)	0.53		5.72	(4.65,6.80)	0.54		6.72	(5.90,7.54)	0.42	
Religious service attendance	More than 1/week	5.44	(5.28,5.60)	0.08	0.609	5.97	(5.79,6.15)	0.09	0.827	7.20	(7.04,7.37)	0.08	< .001
	1/week	5.56	(5.43,5.68)	0.06		6.00	(5.87,6.13)	0.07		7.41	(7.31,7.52)	0.05	
	1-3/month	5.43	(5.20,5.66)	0.12		5.95	(5.72,6.18)	0.12		7.11	(6.89,7.33)	0.11	
	A few times a year	5.46	(5.21,5.71)	0.13		5.82	(5.53,6.11)	0.15		6.80	(6.55,7.04)	0.12	
	Never	5.62	(5.24,5.99)	0.19		5.87	(5.46,6.28)	0.21		7.22	(6.88,7.55)	0.17	
Education	Up to 8 years	5.38	(5.21,5.55)	0.09	0.084	6.06	(5.89,6.23)	0.09	0.103	7.35	(7.20,7.50)	0.08	0.001
	9-15 years	5.59	(5.48,5.69)	0.05		5.93	(5.83,6.03)	0.05		7.27	(7.18,7.36)	0.05	

Variable	Category	Cantril's Ladder				Life Satisfaction				Happiness			
		Mean	95% CI	SE	Global p-value	Mean	95% CI	SE	Global p-value	Mean	95% CI	SE	Global p-value
	16+ years	5.63	(5.38,5.88)	0.13		5.75	(5.48,6.01)	0.14		6.85	(6.62,7.08)	0.12	
Immigration status	Born in this country	5.51	(5.41,5.61)	0.05	0.817	5.97	(5.87,6.07)	0.05	0.471	7.27	(7.19,7.36)	0.04	0.705
	Born in another country	5.43	(4.72,6.14)	0.36		5.70	(4.98,6.43)	0.37		7.16	(6.56,7.76)	0.30	
Religious affiliation	Christianity	5.47	(5.37,5.58)	0.05	< .001	5.95	(5.84,6.05)	0.05	*	7.28	(7.20,7.37)	0.05	< .001
	Islam	5.95	(5.65,6.24)	0.15		6.28	(5.98,6.59)	0.16		7.20	(6.89,7.51)	0.16	
	Buddhism	5.00	*	*		6.00	*	*		7.00	*	*	
	Judaism	4.50	*	*		10.00	*	*		5.84	*	*	
	Baha'i	10.00	*	*		10.00	*	*		10.00	*	*	
	Jainism	5.00	*	*		7.00	*	*		6.00	*	*	
	Confucianism	10.00	*	*		7.00	*	*		8.00	*	*	
	Primal, Animist, or Folk religion	4.12	(1.12,7.11)	1.50		4.50	(2.05,6.96)	1.23		5.53	(3.13,7.92)	1.20	
	Some other religion	6.70	*	*		6.25	*	*		7.26	*	*	
	No religion/Atheist /Agnostic	5.30	(4.43,6.18)	0.44		5.35	(4.53,6.17)	0.41		7.04	(6.36,7.72)	0.34	
Race/Ethnicity	Luhya	5.42	(5.14,5.70)	0.14	< .001	5.65	(5.41,5.90)	0.12	< .001	7.26	(7.07,7.44)	0.09	< .001
	Luo	5.20	(4.95,5.44)	0.12		5.76	(5.50,6.02)	0.13		6.92	(6.65,7.20)	0.14	
	Kalenjin	5.81	(5.63,6.00)	0.10		6.20	(5.93,6.47)	0.14		7.66	(7.48,7.84)	0.09	
	Kamba	5.57	(5.36,5.78)	0.11		5.93	(5.68,6.17)	0.12		7.61	(7.33,7.90)	0.15	
	Kikuyu	5.37	(5.13,5.62)	0.12		6.01	(5.77,6.26)	0.13		7.08	(6.90,7.26)	0.09	
	Kisii	4.99	(4.78,5.21)	0.11		5.67	(5.36,5.99)	0.16		6.49	(6.29,6.68)	0.10	
	Maasai	6.45	(5.91,6.99)	0.28		6.97	(6.43,7.51)	0.27		8.16	(7.56,8.76)	0.30	
	Meru	5.73	(5.43,6.04)	0.16		6.38	(5.95,6.82)	0.22		7.51	(7.08,7.95)	0.22	
	Kenyan Somali/Somali	6.80	(6.51,7.10)	0.15		6.61	(5.95,7.26)	0.33		7.64	(7.04,8.24)	0.30	
	Miji Kenda tribes	5.34	(4.94,5.73)	0.20		5.98	(5.73,6.24)	0.13		7.21	(6.94,7.49)	0.14	
	Embu	5.57	(5.23,5.92)	0.17		5.49	(4.93,6.05)	0.28		7.02	(6.59,7.46)	0.22	
	Other	5.43	(5.09,5.76)	0.17		6.07	(5.73,6.40)	0.17		7.39	(7.11,7.67)	0.14	

Table S12c. Childhood predictors regression analysis results for Kenya

		Cantril's Ladder				Life Satisfaction				Happiness			
Variable	Category	Est	95% CI	SE	Global p-value	Est	95% CI	SE	Global p-value	Est	95% CI	SE	Global p-value
Relationship with mother	(Ref: Very bad/somewhat bad)				0.633				0.387				0.122
	Very good/somewhat good	0.11	(-0.39, 0.61)	0.25		0.22	(-0.32, 0.76)	0.28		0.33	(-0.10, 0.76)	0.22	
Relationship with father	(Ref: Very bad/somewhat bad)				0.215				0.168				0.132
	Very good/somewhat good	0.24	(-0.14, 0.62)	0.19		0.27	(-0.12, 0.65)	0.20		0.27	(-0.08, 0.62)	0.18	
Parent marital status	(Ref: Parents married)				0.167				0.003				0.003
	Divorced	-0.06	(-0.42, 0.29)	0.18		-0.15	(-0.47, 0.18)	0.16		-0.24	(-0.53, 0.06)	0.15	
	Parents were never married	-0.17	(-0.54, 0.21)	0.19		-0.54	(-0.89, -0.19)	0.18		-0.45	(-0.80, -0.11)	0.18	
	One or both parents had died	-0.42	(-0.82, -0.02)	0.20		-0.31	(-0.71, 0.09)	0.20		-0.42	(-0.80, -0.04)	0.19	
Subjective financial status of family growing up	(Ref: Got by)				<.001				<.001				0.012
	Lived comfortably	0.46	(0.27, 0.66)	0.10		0.26	(0.06, 0.47)	0.10		0.26	(0.09, 0.43)	0.09	
	Found it difficult	-0.12	(-0.31, 0.06)	0.10		-0.31	(-0.50, -0.12)	0.10		0.09	(-0.08, 0.26)	0.09	
	Found it very difficult	-0.14	(-0.45, 0.18)	0.16		-0.21	(-0.57, 0.14)	0.18		-0.03	(-0.31, 0.25)	0.14	
Abuse	(Ref: No)				0.161				0.004				<.001
	Yes	-0.16	(-0.38, 0.06)	0.11		-0.38	(-0.64, -0.13)	0.13		-0.39	(-0.62, -0.17)	0.11	
Outsider growing up	(Ref: No)				0.701				0.060				0.722
	Yes	0.04	(-0.20, 0.29)	0.13		-0.26	(-0.54, 0.01)	0.14		-0.05	(-0.31, 0.22)	0.14	
Self-rated health growing up	(Ref: Good)				0.040				0.003				0.004
	Excellent	0.23	(0.01, 0.44)	0.11		0.08	(-0.13, 0.29)	0.11		0.21	(0.03, 0.39)	0.09	

Variable	Category	Cantril's Ladder				Life Satisfaction				Happiness			
		Est	95% CI	SE	Global p-value	Est	95% CI	SE	Global p-value	Est	95% CI	SE	Global p-value
	Very good	0.25	(0.03, 0.48)	0.12		0.08	(-0.15, 0.30)	0.12		0.15	(-0.05, 0.35)	0.10	
	Fair	-0.03	(-0.32, 0.26)	0.15		-0.24	(-0.51, 0.03)	0.14		-0.08	(-0.32, 0.15)	0.12	
	Poor	-0.18	(-0.73, 0.37)	0.28		-0.62	(-1.18, -0.06)	0.29		-0.55	(-1.08, -0.03)	0.27	
Immigration status	(Ref: Born in this country)				0.737				0.673				0.776
	Born in another country	-0.12	(-0.83, 0.58)	0.36		-0.15	(-0.83, 0.53)	0.35		-0.09	(-0.68, 0.51)	0.30	
Age 12 religious service attendance	(Ref: Never)				0.362				0.021				0.502
	At least 1/week	0.30	(-0.39, 1.00)	0.35		-0.47	(-1.15, 0.20)	0.35		-0.47	(-1.08, 0.14)	0.31	
	1-3/month	0.46	(-0.29, 1.20)	0.38		-0.55	(-1.30, 0.19)	0.38		-0.48	(-1.13, 0.18)	0.34	
	< 1/month	0.50	(-0.29, 1.29)	0.40		-1.17	(-1.98, -0.37)	0.41		-0.46	(-1.18, 0.26)	0.37	
Year of birth	(Ref: 1998-2005; current age: 18-24)				<.001				<.001				<.001
	1993-1998; age 25-29	-0.23	(-0.42, -0.05)	0.10		-0.14	(-0.37, 0.09)	0.12		-0.32	(-0.50, -0.15)	0.09	
	1983-1993; age 30-39	-0.47	(-0.67, -0.27)	0.10		-0.36	(-0.58, -0.15)	0.11		-0.44	(-0.61, -0.28)	0.08	
	1973-1983; age 40-49	-0.77	(-1.03, -0.51)	0.13		-0.68	(-0.93, -0.43)	0.13		-0.74	(-0.97, -0.51)	0.12	
	1963-1973; age 50-59	-0.52	(-0.82, -0.23)	0.15		-0.16	(-0.51, 0.18)	0.18		-0.58	(-0.89, -0.26)	0.16	
	1953-1963; age 60-69	-0.55	(-0.95, -0.15)	0.20		0.36	(-0.11, 0.82)	0.24		-0.50	(-0.87, -0.13)	0.19	
	1943-1953; age 70-79	-0.44	(-1.11, 0.22)	0.34		-0.03	(-0.64, 0.58)	0.31		-0.42	(-0.98, 0.14)	0.28	
	1943 or earlier; age 80+	-0.69	(-2.12, 0.73)	0.73		0.59	(-0.63, 1.82)	0.63		-0.05	(-1.34, 1.25)	0.66	
Gender	(Ref: Male)				0.003				0.522				0.008
	Female	0.03	(-0.12, 0.19)	0.08		-0.03	(-0.18, 0.13)	0.08		0.21	(0.08, 0.35)	0.07	
	Other	3.70	(1.56, 5.85)	1.09		2.54	(-2.10, 7.18)	2.37		0.98	(-3.16, 5.13)	2.11	
Religious affiliation	(Ref: Christianity)				0.042				0.057				0.552
	Islam	0.36	(0.06, 0.66)	0.15		0.36	(0.07, 0.66)	0.15		-0.04	(-0.32, 0.24)	0.14	
	Collapsed affiliations with prevalence<3%	0.57	(-0.39, 1.52)	0.49		0.18	(-0.84, 1.21)	0.52		-0.48	(-1.35, 0.39)	0.44	

Variable	Category	Cantril's Ladder				Life Satisfaction				Happiness			
		Est	95% CI	SE	Global p-value	Est	95% CI	SE	Global p-value	Est	95% CI	SE	Global p-value
Race/ethnicity	(Ref: Plurality group)				0.672				0.429				0.050
	Non-plurality groups	0.05	(-0.20, 0.31)	0.13		-0.10	(-0.35, 0.15)	0.13		0.20	(-0.00, 0.40)	0.10	

Table S12d. Sensitivity to unmeasured confounding of childhood predictors in Kenya

		Cantril's Ladder		Life Satisfaction		Happiness	
Variable	Category	E-value for Estimate	E-value for 95% CI	E-value for Estimate	E-value for 95% CI	E-value for Estimate	E-value for 95% CI
Relationship with mother	(Ref: Very bad/somewhat bad)						
	Very good/somewhat good	1.21	1.00	1.31	1.00	1.45	1.00
Relationship with father	(Ref: Very bad/somewhat bad)						
	Very good/somewhat good	1.34	1.00	1.35	1.00	1.39	1.00
Parent marital status	(Ref: Parents married)						
	Divorced	1.15	1.00	1.24	1.00	1.36	1.00
	Parents were never married	1.27	1.00	1.57	1.29	1.56	1.22
	One or both parents had died	1.50	1.09	1.39	1.00	1.54	1.13
Subjective financial status of family growing up	(Ref: Got by)						
	Lived comfortably	1.53	1.37	1.35	1.14	1.38	1.20
	Found it difficult	1.23	1.00	1.39	1.21	1.20	1.00
	Found it very difficult	1.24	1.00	1.30	1.00	1.11	1.00
Abuse	(Ref: No)						
	Yes	1.26	1.00	1.44	1.22	1.51	1.29
Outsider growing up	(Ref: No)						
	Yes	1.12	1.00	1.35	1.00	1.14	1.00
Self-rated health growing up	(Ref: Good)						
	Excellent	1.33	1.07	1.17	1.00	1.33	1.11
	Very good	1.35	1.10	1.16	1.00	1.27	1.00
	Fair	1.10	1.00	1.33	1.00	1.19	1.00
	Poor	1.28	1.00	1.63	1.15	1.65	1.11
Immigration status	(Ref: Born in this country)						
	Born in another country	1.22	1.00	1.24	1.00	1.19	1.00
Age 12 religious service attendance	(Ref: Never)						
	At least 1/week	1.40	1.00	1.52	1.00	1.58	1.00
	1-3/month	1.53	1.00	1.58	1.00	1.58	1.00
	< 1/month	1.56	1.00	2.05	1.43	1.57	1.00
Year of birth	(Ref: 1998-2005; current age: 18-24)						
	1993-1998; age 25-29	1.33	1.13	1.23	1.00	1.44	1.27
	1983-1993; age 30-39	1.54	1.36	1.43	1.24	1.55	1.40
	1973-1983; age 40-49	1.78	1.57	1.67	1.48	1.82	1.62
	1963-1973; age 50-59	1.58	1.33	1.25	1.00	1.67	1.39

Variable	Category	Cantril's Ladder		Life Satisfaction		Happiness	
		E-value for Estimate	E-value for 95% CI	E-value for Estimate	E-value for 95% CI	E-value for Estimate	E-value for 95% CI
	1953-1963; age 60-69	1.60	1.26	1.42	1.00	1.61	1.25
	1943-1953; age 70-79	1.52	1.00	1.10	1.00	1.53	1.00
	1943 or earlier; age 80+	1.72	1.00	1.61	1.00	1.14	1.00
Gender	(Ref: Male)						
	Female	1.11	1.00	1.09	1.00	1.34	1.18
	Other	5.04	2.46	3.28	1.00	2.04	1.00
Religious affiliation	(Ref: Christianity)						
	Islam	1.44	1.15	1.43	1.15	1.12	1.00
	Collapsed affiliations with prevalence<3%	1.62	1.00	1.27	1.00	1.59	1.00
Race/ethnicity	(Ref: Plurality group)						
	Non-plurality groups	1.14	1.00	1.19	1.00	1.32	1.00

Table S12e. Complete-case supplemental analysis of means by demographic category for Kenya

		Cantril's Ladder				Life Satisfaction				Happiness			
Variable	Category	Mean	95% CI	SE	Global p-value	Mean	95% CI	SE	Global p-value	Mean	95% CI	SE	Global p-value
Age group	18-24	6.50	(6.39,6.62)	0.06	< .001	7.23	(7.11,7.36)	0.06	< .001	7.96	(7.84,8.07)	0.06	< .001
	25-29	6.30	(6.15,6.46)	0.08		7.01	(6.85,7.16)	0.08		7.70	(7.56,7.85)	0.07	
	30-39	6.19	(6.03,6.34)	0.08		6.92	(6.78,7.06)	0.07		7.66	(7.55,7.78)	0.06	
	40-49	5.97	(5.76,6.18)	0.11		6.68	(6.48,6.88)	0.10		7.42	(7.22,7.61)	0.10	
	50-59	6.13	(5.85,6.41)	0.14		7.03	(6.78,7.29)	0.13		7.60	(7.37,7.83)	0.12	
	60-69	6.35	(5.99,6.71)	0.18		7.55	(7.21,7.90)	0.18		7.70	(7.40,8.01)	0.15	
	70-79	6.41	(5.85,6.97)	0.28		7.12	(6.56,7.68)	0.28		7.70	(7.15,8.24)	0.28	
	80 or older	6.87	(5.53,8.20)	0.67		7.92	(6.81,9.04)	0.56		8.14	(6.92,9.35)	0.61	
Gender	Female	6.33	(6.20,6.45)	0.06	0.018	7.13	(7.01,7.24)	0.06	< .001	7.84	(7.74,7.95)	0.05	< .001
	Male	6.22	(6.10,6.34)	0.06		6.96	(6.85,7.08)	0.06		7.57	(7.47,7.67)	0.05	
	Other	9.00	(0.00,816.90)	1.14		10.00	(10.00,10.00)	0.00		10.00	(10.00,10.00)	0.00	
Marital status	Divorced	5.35	(4.75,5.95)	0.30	0.008	6.70	(6.17,7.22)	0.26	0.065	6.99	(6.41,7.57)	0.29	0.004
	Domestic partner	6.22	(5.65,6.79)	0.29		6.68	(6.18,7.18)	0.25		7.54	(7.02,8.06)	0.26	
	Married	6.25	(6.13,6.38)	0.06		7.07	(6.96,7.18)	0.06		7.76	(7.66,7.86)	0.05	
	Separated	6.19	(5.82,6.56)	0.19		6.69	(6.32,7.05)	0.19		7.55	(7.24,7.87)	0.16	
	Single/Never been married	6.38	(6.27,6.49)	0.06		7.02	(6.90,7.15)	0.07		7.75	(7.63,7.86)	0.06	
	Widowed	6.04	(5.59,6.49)	0.23		7.39	(7.00,7.78)	0.20		7.07	(6.64,7.51)	0.22	
Employment	Employed for an employer	6.21	(6.01,6.40)	0.10	0.022	6.71	(6.54,6.88)	0.09	< .001	7.52	(7.36,7.67)	0.08	< .001
	Homemaker	6.45	(6.24,6.67)	0.11		7.45	(7.24,7.66)	0.11		8.01	(7.82,8.19)	0.09	
	None of these/Other	6.54	(5.78,7.30)	0.38		7.08	(6.20,7.97)	0.45		7.65	(6.98,8.32)	0.34	
	Retired	6.49	(5.98,7.01)	0.26		7.03	(6.51,7.54)	0.26		7.65	(7.23,8.08)	0.22	
	Self-employed	6.12	(5.97,6.28)	0.08		7.03	(6.90,7.17)	0.07		7.60	(7.48,7.73)	0.06	
	Student	6.44	(6.27,6.61)	0.09		7.08	(6.90,7.25)	0.09		7.79	(7.63,7.95)	0.08	
	Unemployed and looking for a job	6.30	(6.16,6.44)	0.07		7.01	(6.87,7.16)	0.07		7.76	(7.63,7.89)	0.07	
Religious service attendance	A few times a year	6.03	(5.79,6.27)	0.12	0.020	6.78	(6.55,7.02)	0.12	0.004	7.23	(7.01,7.44)	0.11	< .001
	More than once a week	6.32	(6.16,6.48)	0.08		7.17	(7.02,7.31)	0.07		7.73	(7.60,7.87)	0.07	
	Never	6.48	(6.08,6.89)	0.21		6.93	(6.56,7.29)	0.19		7.55	(7.22,7.89)	0.17	
	Once a week	6.32	(6.21,6.44)	0.06		7.09	(6.97,7.21)	0.06		7.82	(7.72,7.92)	0.05	

Variable	Category	Cantril's Ladder				Life Satisfaction				Happiness			
		Mean	95% CI	SE	Global p-value	Mean	95% CI	SE	Global p-value	Mean	95% CI	SE	Global p-value
	One to three times a month	6.04	(5.83,6.25)	0.11		6.80	(6.61,6.99)	0.10		7.50	(7.33,7.67)	0.09	
Education	Up to 8 years	6.45	(6.29,6.62)	0.09	< .001	7.39	(7.24,7.54)	0.08	< .001	7.97	(7.83,8.10)	0.07	< .001
	16+ years	5.85	(5.61,6.09)	0.12		6.27	(6.04,6.50)	0.12		6.99	(6.78,7.21)	0.11	
	9 to 15 years	6.21	(6.11,6.31)	0.05		6.91	(6.82,7.00)	0.05		7.62	(7.53,7.71)	0.04	
Immigration status	Born in another country	6.35	(5.71,6.99)	0.32	0.816	7.05	(6.39,7.72)	0.34	0.979	8.19	(7.71,8.67)	0.24	0.051
	Born in this country	6.27	(6.17,6.37)	0.05		7.05	(6.95,7.14)	0.05		7.70	(7.62,7.79)	0.04	
Religious affiliation	Buddhism	5.00	*	*	< .001	6.00	*	*	< .001	7.00	*	*	< .001
	Christianity	6.22	(6.11,6.32)	0.05		7.01	(6.92,7.11)	0.05		7.71	(7.62,7.79)	0.04	
	Confucianism	10.00	*	*		7.00	*	*		8.00	*	*	
	Islam	6.92	(6.66,7.19)	0.14		7.45	(7.20,7.70)	0.13		7.81	(7.55,8.06)	0.13	
	Judaism	4.50	*	*		10.00	*	*		5.84	*	*	
	No religion/Atheist/Agnostic	6.08	(5.31,6.86)	0.39		6.80	(6.07,7.53)	0.37		7.47	(6.86,8.07)	0.31	
	Primal, Animist, or Folk religion	5.65	(3.36,7.95)	1.15		4.50	(2.05,6.96)	1.23		5.53	(3.13,7.92)	1.20	
	Some other religion	6.57	*	*		7.88	*	*		8.21	*	*	
	Baha'i	10.00	*	*		10.00	*	*		10.00	*	*	
	Jainism	5.00	*	*		7.00	*	*		6.00	*	*	

Table S12f. Complete-case supplemental analysis of childhood predictors regression analysis results for Kenya

		Cantril's Ladder				Life Satisfaction				Happiness			
Variable	Category	Est	95% CI	SE	Global p-value	Est	95% CI	SE	Global p-value	Est	95% CI	SE	Global p-value
Relationship with mother	(Ref: Very bad/somewhat bad)				0.884				0.348				0.078
	Very good/somewhat good	-0.04	(-0.52,0.45)	0.25		0.22	(-0.24,0.68)	0.23		0.37	(-0.04,0.78)	0.21	
Relationship with father	(Ref: Very bad/somewhat bad)				0.069				0.030				0.158
	Very good/somewhat good	0.30	(-0.02,0.61)	0.16		0.36	(0.04,0.68)	0.16		0.19	(-0.07,0.46)	0.14	
Parent marital status	(Ref: Parents married)				0.195				0.183				0.036
	No, one or both of them had died	-0.31	(-0.75,0.13)	0.22		0.00	(-0.42,0.42)	0.21		-0.06	(-0.41,0.29)	0.18	
	No, they were never married	0.04	(-0.40,0.48)	0.22		-0.13	(-0.50,0.24)	0.19		-0.06	(-0.40,0.27)	0.17	
	Yes, married	0.09	(-0.22,0.39)	0.16		0.13	(-0.12,0.39)	0.13		0.22	(-0.01,0.44)	0.12	
Subjective financial status of family growing up	(Ref: Got by)				<.001				<.001				<.001
	Found it difficult	0.21	(0.04,0.38)	0.09		0.19	(0.03,0.35)	0.08		0.20	(0.05,0.34)	0.07	
	Found it very difficult	0.30	(-0.01,0.62)	0.16		0.38	(0.07,0.69)	0.16		0.28	(0.04,0.51)	0.12	
	Lived comfortably	0.51	(0.35,0.68)	0.08		0.37	(0.22,0.52)	0.08		0.31	(0.16,0.45)	0.08	
Abuse	(Ref: No)				0.426				0.091				0.193
	Yes	-0.09	(-0.31,0.13)	0.11		-0.19	(-0.41,0.03)	0.11		-0.12	(-0.30,0.06)	0.09	
Outsider growing up	(Ref: No)				0.502				0.370				0.715
	Yes	0.08	(-0.15,0.31)	0.12		-0.11	(-0.34,0.13)	0.12		0.04	(-0.17,0.25)	0.11	

		Cantril's Ladder				Life Satisfaction				Happiness			
Variable	Category	Est	95% CI	SE	Global p-value	Est	95% CI	SE	Global p-value	Est	95% CI	SE	Global p-value
Self-rated health growing up	(Ref: Good)				0.003				<.001				0.004
	Excellent	0.24	(0.05,0.43)	0.10		0.30	(0.12,0.48)	0.09		0.27	(0.10,0.44)	0.09	
	Fair	-0.14	(-0.39,0.12)	0.13		-0.20	(-0.44,0.04)	0.12		-0.02	(-0.24,0.20)	0.11	
	Poor	0.11	(-0.38,0.60)	0.25		0.37	(-0.10,0.84)	0.24		-0.04	(-0.48,0.41)	0.23	
	Very good	0.18	(-0.01,0.38)	0.10		0.18	(-0.00,0.36)	0.09		0.19	(0.01,0.38)	0.09	
Immigration status	(Ref: Born in this country)				0.949				0.974				0.085
	Born in another country	-0.02	(-0.65,0.61)	0.32		-0.01	(-0.63,0.61)	0.32		0.42	(-0.06,0.90)	0.25	
Age 12 religious service attendance	(Ref: Never)				0.011				0.132				0.167
	At least once a week	0.31	(-0.30,0.92)	0.31		-0.37	(-1.01,0.26)	0.32		-0.29	(-0.83,0.24)	0.27	
	Less than once a month	0.40	(-0.35,1.15)	0.38		-0.60	(-1.37,0.17)	0.39		-0.15	(-0.77,0.47)	0.32	
	One to three times a month	0.62	(-0.02,1.25)	0.33		-0.20	(-0.86,0.46)	0.34		-0.14	(-0.71,0.42)	0.29	
Year of birth	(Ref: 1998-2005; current age: 18-24)				0.005				<.001				<.001
	1993-1998; age 25-29	-0.19	(-0.36,-0.02)	0.08		-0.22	(-0.39,-0.04)	0.09		-0.24	(-0.41,-0.07)	0.09	
	1983-1993; age 30-39	-0.25	(-0.41,-0.08)	0.08		-0.27	(-0.44,-0.09)	0.09		-0.26	(-0.41,-0.11)	0.08	
	1973-1983; age 40-49	-0.47	(-0.70,-0.23)	0.12		-0.52	(-0.74,-0.31)	0.11		-0.51	(-0.71,-0.30)	0.11	
	1963-1973; age 50-59	-0.28	(-0.57,0.00)	0.15		-0.16	(-0.44,0.12)	0.14		-0.30	(-0.55,-0.05)	0.13	
	1953-1963; age 60-69	-0.04	(-0.42,0.33)	0.19		0.36	(-0.00,0.71)	0.18		-0.20	(-0.50,0.10)	0.15	
	1943-1953; age 70-79	0.03	(-0.51,0.58)	0.28		-0.12	(-0.67,0.43)	0.28		-0.23	(-0.79,0.33)	0.29	

Variable	Category	Cantril's Ladder				Life Satisfaction				Happiness			
		Est	95% CI	SE	Global p-value	Est	95% CI	SE	Global p-value	Est	95% CI	SE	Global p-value
	1943 or earlier; age 80+	0.40	(-0.92,1.71)	0.67		0.65	(-0.44,1.73)	0.55		0.27	(-0.94,1.48)	0.62	
Gender	(Ref: Male)				0.002				<.001				<.001
	Male	-0.14	(-0.29,0.00)	0.08		-0.19	(-0.33,-0.05)	0.07		-0.29	(-0.41,-0.18)	0.06	
	Other	2.98	(1.00,4.96)	1.01		3.31	(2.98,3.64)	0.17		2.19	(1.89,2.50)	0.16	
Religious affiliation	(Ref: Christianity)				<.001				0.005				0.596
	Islam	0.67	(0.37,0.96)	0.15		0.42	(0.16,0.67)	0.13		0.11	(-0.13,0.36)	0.13	
	Collapsed affiliations with prevalence<3 %	0.60	(-0.33,1.53)	0.48		0.31	(-0.64,1.26)	0.48		-0.19	(-1.03,0.65)	0.43	
Race/ethnicity	(Ref: Plurality group)				0.094				0.230				0.017
	Non-plurality groups	0.23	(-0.04,0.50)	0.14		0.14	(-0.09,0.37)	0.12		0.25	(0.05,0.46)	0.10	

Table S12g. Sensitivity to unmeasured confounding of childhood predictors in Kenya

		Cantril's Ladder		Life Satisfaction		Happiness	
Variable	Category	E-value for Estimate	E-value for 95% CI	E-value for Estimate	E-value for 95% CI	E-value for Estimate	E-value for 95% CI
Relationship with mother	(Ref: Very bad/somewhat bad)						
	Very good/somewhat good	1.12	1.00	1.37	1.00	1.56	1.00
Relationship with father	(Ref: Very bad/somewhat bad)						
	Very good/somewhat good	1.44	1.00	1.52	1.13	1.36	1.00
Parent marital status	(Ref: Parents married)						
	No, one or both of them had died	1.46	1.00	1.02	1.00	1.18	1.00
	No, they were never married	1.13	1.00	1.27	1.00	1.18	1.00
	Yes, married	1.20	1.00	1.27	1.00	1.38	1.00
Subjective financial status of family growing up	(Ref: Got by)						
	Found it difficult	1.35	1.13	1.33	1.11	1.37	1.17
	Found it very difficult	1.45	1.00	1.54	1.18	1.46	1.14
	Lived comfortably	1.66	1.50	1.53	1.37	1.49	1.32
Abuse	(Ref: No)						
	Yes	1.21	1.00	1.34	1.00	1.26	1.00
Outsider growing up	(Ref: No)						
	Yes	1.19	1.00	1.24	1.00	1.14	1.00
Self-rated health growing up	(Ref: Good)						
	Excellent	1.39	1.15	1.46	1.25	1.45	1.24
	Fair	1.27	1.00	1.34	1.00	1.10	1.00
	Poor	1.24	1.00	1.53	1.00	1.14	1.00
	Very good	1.32	1.00	1.32	1.00	1.36	1.06
Immigration status	(Ref: Born in this country)						
	Born in another country	1.09	1.00	1.06	1.00	1.62	1.00
Age 12 religious service attendance	(Ref: Never)						
	At least once a week	1.46	1.00	1.53	1.00	1.48	1.00
	Less than once a month	1.55	1.00	1.76	1.00	1.30	1.00
	One to three times a month	1.76	1.00	1.35	1.00	1.30	1.00
Year of birth	(Ref: 1998-2005; current age: 18-24)						

Variable	Category	Cantril’s Ladder		Life Satisfaction		Happiness	
		E-value for Estimate	E-value for 95% CI	E-value for Estimate	E-value for 95% CI	E-value for Estimate	E-value for 95% CI
	1993-1998; age 25-29	1.33	1.10	1.37	1.13	1.41	1.19
	1983-1993; age 30-39	1.39	1.20	1.42	1.21	1.44	1.25
	1973-1983; age 40-49	1.61	1.38	1.68	1.46	1.71	1.48
	1963-1973; age 50-59	1.43	1.00	1.30	1.00	1.48	1.15
	1953-1963; age 60-69	1.14	1.00	1.51	1.00	1.36	1.00
	1943-1953; age 70-79	1.12	1.00	1.25	1.00	1.40	1.00
	1943 or earlier; age 80+	1.55	1.00	1.81	1.00	1.45	1.00
Gender	(Ref: Male)						
	Male	1.28	1.00	1.34	1.15	1.48	1.34
	Other	4.85	2.14	5.72	5.04	3.96	3.46
Religious affiliation	(Ref: Christianity)						
	Islam	1.81	1.52	1.58	1.31	1.26	1.00
	Collapsed affiliations with prevalence<3%	1.74	1.00	1.47	1.00	1.36	1.00
Race/ethnicity	(Ref: Plurality group)						
	Non-plurality groups	1.37	1.00	1.28	1.00	1.43	1.15

Tables S13a-g: Mexico

Table S13a. Nationally representative descriptive statistics for Mexico

Characteristic	N = 5,776[1]
**Age group**	
1998-2005; age 18-24	986 (17%)
1993-1998; age 25-29	623 (11%)
1983-1993; age 30-39	1,312 (23%)
1973-1983; age 40-49	1,027 (18%)
1963-1973; age 50-59	873 (15%)
1953-1963; age 60-69	611 (11%)
1943-1953; age 70-79	277 (4.8%)
1943 or earlier; age 80+	68 (1.2%)
(Missing)	0 (0%)
**Gender**	
Male	2,755 (48%)
Female	2,997 (52%)
Other	3 (<0.1%)
(Missing)	21 (0.4%)
**Race/Ethnicity**	
Black	108 (1.9%)
Indigenous	594 (10%)
Mestizo	2,762 (48%)
Mulatto	63 (1.1%)
Other	339 (5.9%)
White	1,116 (19%)
(Missing)	794 (14%)
**Respondent Marital status**	
Married	2,089 (36%)
Separated	403 (7.0%)
Divorced	230 (4.0%)
Widowed	347 (6.0%)
Single, never married	1,432 (25%)
Domestic Partner	1,109 (19%)
(Missing)	166 (2.9%)
**Employment**	
Employed for an employer	1,921 (33%)
Self-employed	1,091 (19%)
Retired	386 (6.7%)
Student	247 (4.3%)
Homemaker	1,257 (22%)
Unemployed and looking for a job	564 (9.8%)
None of these/Other	169 (2.9%)
(Missing)	141 (2.4%)
**Religious service attendance as an adult (now)**	
More than 1/week	609 (11%)
1/week	1,261 (22%)
1-3/month	676 (12%)
A few times a year	2,054 (36%)
Never	1,134 (20%)
(Missing)	43 (0.7%)
**Education (years)**	
Up to 8 years	1,291 (22%)
9-15 years	3,180 (55%)
16+ years	1,304 (23%)
(Missing)	1 (<0.1%)
**Immigration status**	
Born in this country	5,517 (96%)
Born in another country	108 (1.9%)
(Missing)	151 (2.6%)
**Religious affiliation as an adult (now)**	
Christianity	4,844 (84%)
Islam	2 (<0.1%)
Hinduism	3 (<0.1%)
Buddhism	6 (0.1%)

Characteristic	N = 5,776[1]
Judaism	7 (0.1%)
Sikhism	0 (0%)
Baha'i	1 (<0.1%)
Jainism	1 (<0.1%)
Shinto	2 (<0.1%)
Taoism	4 (<0.1%)
Confucianism	1 (<0.1%)
Primal, Animist, or Folk religion	20 (0.3%)
Spiritism	0 (0%)
Umbanda, Candomble, and other African-derived religions	0 (0%)
Chinese folk/traditional religion	0 (0%)
Some other religion	41 (0.7%)
No religion/Atheist/Agnostic	770 (13%)
(Missing)	75 (1.3%)
**Relationship with mother growing up**	
Very good	3,912 (68%)
Somewhat good	1,340 (23%)
Somewhat bad	177 (3.1%)
Very bad	90 (1.6%)
Does not apply	177 (3.1%)
(Missing)	80 (1.4%)
**Relationship with father growing up**	
Very good	3,089 (53%)
Somewhat good	1,556 (27%)
Somewhat bad	335 (5.8%)
Very bad	267 (4.6%)
Does not apply	470 (8.1%)
(Missing)	60 (1.0%)
**Parent marital status at age 12**	
Parents married	3,999 (69%)
Divorced	341 (5.9%)
Parents were never married	827 (14%)
One or both parents had died	176 (3.0%)
(Missing)	432 (7.5%)
**Subjective financial status of family growing up**	
Lived comfortably	1,775 (31%)
Got by	1,872 (32%)
Found it difficult	1,712 (30%)
Found it very difficult	369 (6.4%)
(Missing)	48 (0.8%)
**Abuse**	
Yes	905 (16%)
No	4,604 (80%)
(Missing)	267 (4.6%)
**Outsider growing up**	
Yes	772 (13%)
No	4,897 (85%)
(Missing)	107 (1.9%)
**Self-rated health growing up**	
Excellent	1,860 (32%)
Very good	1,350 (23%)
Good	1,677 (29%)
Fair	743 (13%)
Poor	133 (2.3%)
(Missing)	14 (0.2%)
**Age 12 religious service attendance**	
At least 1/week	2,514 (44%)
1-3/month	1,162 (20%)
<1/month	1,087 (19%)
Never	944 (16%)
(Missing)	69 (1.2%)
**Religious affiliation at age 12**	
Christianity	5,337 (92%)
Islam	6 (<0.1%)
Hinduism	1 (<0.1%)

Characteristic	N = 5,776[1]
Buddhism	1 (<0.1%)
Judaism	8 (0.1%)
Sikhism	4 (<0.1%)
Baha'i	1 (<0.1%)
Jainism	0 (0%)
Shinto	2 (<0.1%)
Taoism	5 (<0.1%)
Confucianism	0 (0%)
Primal, Animist, or Folk religion	2 (<0.1%)
Spiritism	0 (0%)
Umbanda, Candomble, and other African-derived religions	0 (0%)
Chinese folk/traditional religion	0 (0%)
Some other religion	7 (0.1%)
No religion/Atheist/Agnostic	328 (5.7%)
(Missing)	74 (1.3%)

[1] n (%)

Table S13b. Means by demographic category for Mexico

Variable	Category	Cantril's Ladder				Life Satisfaction				Happiness			
		Mean	95% CI	SE	Global p-value	Mean	95% CI	SE	Global p-value	Mean	95% CI	SE	Global p-value
Age group	18-24	7.06	(6.92,7.20)	0.07	0.896	7.69	(7.54,7.85)	0.08	0.113	7.77	(7.62,7.92)	0.08	0.872
	25-29	7.16	(6.97,7.34)	0.09		7.73	(7.54,7.91)	0.10		7.77	(7.59,7.94)	0.09	
	30-39	7.10	(6.96,7.24)	0.07		7.81	(7.67,7.96)	0.07		7.84	(7.71,7.97)	0.07	
	40-49	7.02	(6.85,7.20)	0.09		7.87	(7.70,8.04)	0.09		7.78	(7.62,7.95)	0.09	
	50-59	7.20	(7.01,7.39)	0.10		7.97	(7.79,8.16)	0.09		7.86	(7.69,8.04)	0.09	
	60-69	7.05	(6.79,7.31)	0.13		7.93	(7.68,8.18)	0.13		7.64	(7.38,7.89)	0.13	
	70-79	7.18	(6.81,7.54)	0.18		8.25	(7.83,8.67)	0.21		7.71	(7.29,8.14)	0.22	
	80 or older	7.23	(6.43,8.03)	0.40		7.69	(6.91,8.48)	0.39		7.72	(7.17,8.26)	0.27	
Gender	Male	7.12	(7.01,7.22)	0.05	0.790	7.91	(7.80,8.02)	0.06	0.099	7.80	(7.69,7.90)	0.05	0.260
	Female	7.09	(7.00,7.18)	0.05		7.79	(7.70,7.89)	0.05		7.78	(7.69,7.87)	0.05	
	Other	7.46	*	*		6.36	*	*		6.56	*	*	
Marital status	Married	7.34	(7.23,7.46)	0.06	< .001	8.16	(8.05,8.27)	0.06	< .001	7.99	(7.88,8.10)	0.06	< .001
	Separated	6.88	(6.61,7.15)	0.14		7.35	(7.06,7.63)	0.14		7.27	(6.99,7.55)	0.14	
	Divorced	7.02	(6.64,7.40)	0.19		7.49	(7.10,7.88)	0.20		7.64	(7.29,8.00)	0.18	
	Widowed	6.89	(6.57,7.21)	0.16		7.85	(7.46,8.23)	0.20		7.69	(7.35,8.03)	0.17	
	Never	7.00	(6.88,7.13)	0.06		7.50	(7.36,7.65)	0.08		7.53	(7.39,7.66)	0.07	
	Domestic Partner	6.94	(6.78,7.10)	0.08		7.97	(7.82,8.11)	0.07		7.98	(7.85,8.11)	0.07	
Employment	Employed for an employer	7.25	(7.14,7.37)	0.06	< .001	7.85	(7.73,7.98)	0.06	< .001	7.84	(7.73,7.96)	0.06	< .001
	Self-employed	7.04	(6.89,7.18)	0.08		7.92	(7.77,8.08)	0.08		7.76	(7.61,7.92)	0.08	
	Retired	7.60	(7.34,7.86)	0.13		8.37	(8.16,8.58)	0.11		8.06	(7.81,8.31)	0.13	
	Student	6.97	(6.66,7.28)	0.16		7.52	(7.20,7.85)	0.16		7.49	(7.20,7.78)	0.15	
	Homemaker	7.07	(6.91,7.23)	0.08		8.00	(7.85,8.15)	0.08		7.98	(7.83,8.12)	0.07	
	Unemployed and looking for a job	6.59	(6.35,6.82)	0.12		7.19	(6.96,7.43)	0.12		7.30	(7.07,7.52)	0.11	
	None of these/Other	6.85	(6.39,7.31)	0.23		7.73	(7.21,8.24)	0.26		7.33	(6.87,7.79)	0.23	
Religious service attendance	More than 1/week	7.40	(7.17,7.64)	0.12	0.009	8.40	(8.19,8.60)	0.10	< .001	8.21	(8.00,8.42)	0.11	< .001
	1/week	7.15	(7.00,7.30)	0.08		7.88	(7.73,8.04)	0.08		7.83	(7.68,7.97)	0.07	
	1-3/month	7.04	(6.82,7.25)	0.11		7.97	(7.75,8.18)	0.11		7.82	(7.63,8.01)	0.09	
	A few times a year	7.11	(7.00,7.22)	0.06		7.87	(7.76,7.98)	0.06		7.82	(7.72,7.92)	0.05	
	Never	6.91	(6.76,7.07)	0.08		7.41	(7.24,7.58)	0.09		7.44	(7.28,7.59)	0.08	
Education	Up to 8 years	6.82	(6.66,6.99)	0.09	< .001	7.90	(7.73,8.07)	0.09	0.699	7.67	(7.51,7.83)	0.08	0.200
	9-15 years	7.09	(7.00,7.18)	0.05		7.85	(7.76,7.94)	0.05		7.83	(7.75,7.92)	0.04	

Variable	Category	Cantril's Ladder				Life Satisfaction				Happiness			
		Mean	95% CI	SE	Global p-value	Mean	95% CI	SE	Global p-value	Mean	95% CI	SE	Global p-value
	16+ years	7.39	(7.26,7.53)	0.07		7.80	(7.66,7.95)	0.07		7.78	(7.65,7.91)	0.07	
Immigration status	Born in this country	7.11	(7.04,7.18)	0.03	0.114	7.87	(7.80,7.94)	0.04	< .001	7.79	(7.73,7.86)	0.03	0.055
	Born in another country	6.68	(6.13,7.22)	0.28		6.80	(6.22,7.38)	0.29		7.31	(6.80,7.82)	0.26	
Religious affiliation	Christianity	7.13	(7.06,7.21)	0.04	< .001	7.94	(7.86,8.02)	0.04	< .001	7.86	(7.79,7.93)	0.04	< .001
	Islam	6.92	*	*		8.58	*	*		7.48	*	*	
	Hinduism	9.35	*	*		9.51	*	*		7.68	*	*	
	Buddhism	4.55	*	*		5.62	*	*		6.46	*	*	
	Judaism	6.80	*	*		7.41	*	*		8.97	*	*	
	Baha'i	6.00	*	*		8.00	*	*		8.00	*	*	
	Jainism	5.14	*	*		2.00	*	*		3.00	*	*	
	Shinto	6.33	*	*		7.33	*	*		7.28	*	*	
	Taoism	9.69	*	*		9.88	*	*		9.88	*	*	
	Confucianism	8.00	*	*		10.00	*	*		8.00	*	*	
	Primal, Animist, or Folk religion	6.20	(4.34,8.06)	0.72		7.00	(4.89,9.12)	0.84		7.28	(5.15,9.42)	0.82	
	Some other religion	7.33	(6.61,8.05)	0.35		7.61	(6.95,8.26)	0.32		7.97	(7.49,8.46)	0.24	
	No religion/Atheist /Agnostic	6.93	(6.75,7.11)	0.09		7.31	(7.13,7.50)	0.09		7.33	(7.16,7.51)	0.09	
Race/Ethnicity	Black	7.15	(6.53,7.76)	0.31	< .001	7.98	(7.37,8.58)	0.30	0.301	7.66	(7.04,8.29)	0.32	0.227
	Indigenous	6.63	(6.40,6.87)	0.12		7.96	(7.76,8.17)	0.10		7.66	(7.44,7.88)	0.11	
	White	7.42	(7.27,7.58)	0.08		7.91	(7.76,8.07)	0.08		7.93	(7.78,8.08)	0.07	
	Mestizo	7.07	(6.97,7.16)	0.05		7.78	(7.69,7.88)	0.05		7.77	(7.68,7.85)	0.04	
	Mulatto	6.89	(6.25,7.53)	0.32		7.62	(6.76,8.49)	0.43		7.43	(6.43,8.42)	0.49	
	Other	7.19	(6.87,7.51)	0.16		8.00	(7.66,8.35)	0.17		7.81	(7.50,8.12)	0.16	

Table S13c. Childhood predictors regression analysis results for Mexico

		Cantril's Ladder				Life Satisfaction				Happiness			
Variable	Category	Est	95% CI	SE	Global p-value	Est	95% CI	SE	Global p-value	Est	95% CI	SE	Global p-value
Relationship with mother	(Ref: Very bad/somewhat bad)				0.045				0.007				<.001
	Very good/somewhat good	0.33	(-0.01, 0.67)	0.17		0.44	(0.10, 0.77)	0.17		0.52	(0.22, 0.82)	0.15	
Relationship with father	(Ref: Very bad/somewhat bad)				0.453				0.427				0.778
	Very good/somewhat good	-0.08	(-0.30, 0.14)	0.11		0.09	(-0.14, 0.32)	0.12		-0.01	(-0.22, 0.20)	0.11	
Parent marital status	(Ref: Parents married)				0.279				0.277				0.047
	Divorced	-0.04	(-0.30, 0.21)	0.13		0.20	(-0.08, 0.47)	0.14		0.22	(-0.02, 0.46)	0.12	
	Parents were never married	-0.10	(-0.32, 0.11)	0.11		0.08	(-0.14, 0.29)	0.11		-0.16	(-0.35, 0.04)	0.10	
	One or both parents had died	-0.39	(-0.85, 0.06)	0.23		0.24	(-0.17, 0.64)	0.20		0.03	(-0.39, 0.45)	0.21	
Subjective financial status of family growing up	(Ref: Got by)				<.001				<.001				<.001
	Lived comfortably	0.29	(0.13, 0.45)	0.08		0.27	(0.10, 0.43)	0.08		0.20	(0.05, 0.36)	0.08	
	Found it difficult	-0.40	(-0.58, -0.22)	0.09		-0.09	(-0.28, 0.09)	0.09		-0.08	(-0.25, 0.10)	0.09	
	Found it very difficult	-1.01	(-1.40, -0.62)	0.20		-0.21	(-0.55, 0.13)	0.17		-0.36	(-0.70, -0.03)	0.17	
Abuse	(Ref: No)				0.035				<.001				<.001
	Yes	-0.23	(-0.45, -0.01)	0.11		-0.48	(-0.70, -0.27)	0.11		-0.39	(-0.59, -0.20)	0.10	
Outsider growing up	(Ref: No)				0.139				0.002				<.001
	Yes	-0.18	(-0.43, 0.07)	0.13		-0.36	(-0.60, -0.12)	0.12		-0.43	(-0.66, -0.20)	0.12	
Self-rated health growing up	(Ref: Good)				<.001				0.004				<.001
	Excellent	0.50	(0.32, 0.68)	0.09		0.30	(0.12, 0.48)	0.09		0.45	(0.28, 0.61)	0.08	

Variable	Category	Cantril's Ladder				Life Satisfaction				Happiness			
		Est	95% CI	SE	Global p-value	Est	95% CI	SE	Global p-value	Est	95% CI	SE	Global p-value
	Very good	0.47	(0.29, 0.65)	0.09		0.23	(0.05, 0.41)	0.09		0.28	(0.11, 0.45)	0.09	
	Fair	0.07	(-0.20, 0.33)	0.13		-0.10	(-0.37, 0.17)	0.14		-0.33	(-0.59, -0.06)	0.13	
	Poor	0.10	(-0.45, 0.65)	0.28		-0.13	(-0.71, 0.45)	0.30		-0.24	(-0.81, 0.32)	0.29	
Immigration status	(Ref: Born in this country)				0.180				<.001				0.134
	Born in another country	-0.36	(-0.88, 0.17)	0.27		-0.98	(-1.56, -0.40)	0.29		-0.34	(-0.81, 0.12)	0.24	
Age 12 religious service attendance	(Ref: Never)				0.932				0.453				0.220
	At least 1/week	0.07	(-0.15, 0.28)	0.11		0.15	(-0.07, 0.38)	0.12		0.16	(-0.05, 0.38)	0.11	
	1-3/month	0.04	(-0.19, 0.28)	0.12		0.05	(-0.20, 0.29)	0.12		0.01	(-0.22, 0.23)	0.12	
	< 1/month	0.06	(-0.17, 0.30)	0.12		0.05	(-0.18, 0.29)	0.12		0.04	(-0.18, 0.26)	0.11	
Year of birth	(Ref: 1998-2005; current age: 18-24)				0.209				0.073				0.852
	1993-1998; age 25-29	0.16	(-0.07, 0.39)	0.12		0.09	(-0.14, 0.32)	0.12		0.03	(-0.19, 0.25)	0.11	
	1983-1993; age 30-39	0.11	(-0.09, 0.31)	0.10		0.13	(-0.07, 0.34)	0.10		0.07	(-0.13, 0.27)	0.10	
	1973-1983; age 40-49	0.08	(-0.15, 0.32)	0.12		0.19	(-0.04, 0.43)	0.12		0.00	(-0.22, 0.23)	0.12	
	1963-1973; age 50-59	0.33	(0.08, 0.57)	0.12		0.32	(0.08, 0.56)	0.12		0.13	(-0.10, 0.37)	0.12	
	1953-1963; age 60-69	0.23	(-0.08, 0.54)	0.16		0.24	(-0.06, 0.54)	0.15		-0.07	(-0.37, 0.22)	0.15	
	1943-1953; age 70-79	0.36	(-0.02, 0.74)	0.19		0.59	(0.15, 1.03)	0.22		-0.00	(-0.45, 0.45)	0.23	
	1943 or earlier; age 80+	0.27	(-0.51, 1.06)	0.40		-0.07	(-0.84, 0.69)	0.39		-0.18	(-0.74, 0.38)	0.28	
Gender	(Ref: Male)				0.136				0.361				0.086
	Female	0.12	(-0.03, 0.27)	0.07		0.01	(-0.14, 0.16)	0.08		0.14	(-0.00, 0.28)	0.07	
	Other	0.92	(-0.56, 2.40)	0.75		-1.24	(-3.13, 0.65)	0.96		-0.54	(-2.11, 1.03)	0.78	
Religious affiliation	(Ref: No religion/Atheist /Agnostic)				0.866				0.013				0.009
	Christianity	0.07	(-0.24, 0.38)	0.16		0.49	(0.16, 0.81)	0.16		0.39	(0.07, 0.71)	0.16	

Variable	Category	Cantril's Ladder				Life Satisfaction				Happiness			
		Est	95% CI	SE	Global p-value	Est	95% CI	SE	Global p-value	Est	95% CI	SE	Global p-value
Race/ethnicity	Collapsed affiliations with prevalence<3%	-0.05	(-1.12, 1.02)	0.55		0.47	(-0.50, 1.45)	0.50		1.00	(0.23, 1.77)	0.39	
	(Ref: Plurality group)				0.391				0.042				0.661
	Non-plurality groups	0.06	(-0.10, 0.22)	0.08		0.15	(0.00, 0.29)	0.07		0.03	(-0.11, 0.16)	0.07	

Table S13d. Sensitivity to unmeasured confounding of childhood predictors in Mexico

		Cantril's Ladder		Life Satisfaction		Happiness	
Variable	Category	E-value for Estimate	E-value for 95% CI	E-value for Estimate	E-value for 95% CI	E-value for Estimate	E-value for 95% CI
Relationship with mother	(Ref: Very bad/somewhat bad)						
	Very good/somewhat good	1.57	1.00	1.70	1.26	1.84	1.45
Relationship with father	(Ref: Very bad/somewhat bad)						
	Very good/somewhat good	1.23	1.00	1.24	1.00	1.06	1.00
Parent marital status	(Ref: Parents married)						
	Divorced	1.16	1.00	1.39	1.00	1.44	1.00
	Parents were never married	1.26	1.00	1.22	1.00	1.35	1.00
	One or both parents had died	1.65	1.00	1.45	1.00	1.13	1.00
Subjective financial status of family growing up	(Ref: Got by)						
	Lived comfortably	1.52	1.30	1.49	1.26	1.42	1.17
	Found it difficult	1.66	1.44	1.25	1.00	1.23	1.00
	Found it very difficult	2.46	1.94	1.41	1.00	1.63	1.12
Abuse	(Ref: No)						
	Yes	1.44	1.06	1.76	1.49	1.67	1.41
Outsider growing up	(Ref: No)						
	Yes	1.38	1.00	1.60	1.28	1.72	1.42
Self-rated health growing up	(Ref: Good)						
	Excellent	1.78	1.56	1.53	1.28	1.75	1.53
	Very good	1.75	1.52	1.44	1.16	1.52	1.28
	Fair	1.20	1.00	1.25	1.00	1.58	1.20
	Poor	1.26	1.00	1.30	1.00	1.48	1.00
Immigration status	(Ref: Born in this country)						
	Born in another country	1.61	1.00	2.41	1.66	1.60	1.00
Age 12 religious service attendance	(Ref: Never)						
	At least 1/week	1.20	1.00	1.34	1.00	1.36	1.00
	1-3/month	1.16	1.00	1.16	1.00	1.05	1.00
	< 1/month	1.20	1.00	1.17	1.00	1.15	1.00
Year of birth	(Ref: 1998-2005; current age: 18-24)						
	1993-1998; age 25-29	1.35	1.00	1.24	1.00	1.13	1.00
	1983-1993; age 30-39	1.27	1.00	1.31	1.00	1.21	1.00
	1973-1983; age 40-49	1.23	1.00	1.39	1.00	1.04	1.00
	1963-1973; age 50-59	1.57	1.23	1.56	1.23	1.32	1.00

Variable	Category	Cantril's Ladder		Life Satisfaction		Happiness	
		E-value for Estimate	E-value for 95% CI	E-value for Estimate	E-value for 95% CI	E-value for Estimate	E-value for 95% CI
	1953-1963; age 60-69	1.44	1.00	1.46	1.00	1.22	1.00
	1943-1953; age 70-79	1.61	1.00	1.89	1.33	1.03	1.00
	1943 or earlier; age 80+	1.50	1.00	1.21	1.00	1.39	1.00
Gender	(Ref: Male)						
	Female	1.29	1.00	1.06	1.00	1.33	1.00
	Other	2.33	1.00	2.78	1.00	1.87	1.00
Religious affiliation	(Ref: No religion/Atheist/Agnostic)						
	Christianity	1.21	1.00	1.76	1.35	1.67	1.22
	Collapsed affiliations with prevalence<3%	1.17	1.00	1.75	1.00	2.50	1.46
Race/ethnicity	(Ref: Plurality group)						
	Non-plurality groups	1.19	1.00	1.33	1.02	1.12	1.00

Table S13e. Complete-case supplemental analysis of means by demographic category for Mexico

		Cantril's Ladder				Life Satisfaction				Happiness			
Variable	Category	Mean	95% CI	SE	Global p-value	Mean	95% CI	SE	Global p-value	Mean	95% CI	SE	Global p-value
Age group	18-24	7.09	(6.96,7.23)	0.07	0.700	7.73	(7.58,7.88)	0.08	< .001	7.78	(7.64,7.93)	0.08	0.862
	25-29	7.22	(7.05,7.40)	0.09		7.80	(7.62,7.97)	0.09		7.85	(7.69,8.01)	0.08	
	30-39	7.22	(7.09,7.34)	0.06		7.96	(7.83,8.08)	0.07		7.94	(7.82,8.05)	0.06	
	40-49	7.14	(6.98,7.30)	0.08		8.01	(7.87,8.15)	0.07		7.88	(7.74,8.03)	0.07	
	50-59	7.31	(7.13,7.48)	0.09		8.11	(7.94,8.27)	0.09		7.92	(7.75,8.09)	0.09	
	60-69	7.21	(6.97,7.44)	0.12		8.05	(7.82,8.29)	0.12		7.83	(7.60,8.07)	0.12	
	70-79	7.30	(6.98,7.63)	0.17		8.55	(8.30,8.80)	0.13		7.94	(7.59,8.29)	0.18	
	80 or older	7.28	(6.47,8.09)	0.40		7.92	(7.25,8.59)	0.34		7.83	(7.32,8.34)	0.26	
Gender	Female	7.18	(7.10,7.27)	0.04	0.795	7.95	(7.86,8.03)	0.04	0.174	7.88	(7.79,7.96)	0.04	0.222
	Male	7.22	(7.12,7.31)	0.05		8.00	(7.90,8.10)	0.05		7.88	(7.78,7.97)	0.05	
	Other	7.46	(0.00,597645754190473942427238400.00)	0.63		6.26	(0.00,953087998198575126139633664.00)	1.01		6.56	(0.00,717095818288886757080432640.00)	0.76	
Marital status	Divorced	7.25	(6.96,7.53)	0.15	< .001	7.75	(7.46,8.04)	0.15	< .001	7.76	(7.46,8.06)	0.15	< .001
	Domestic partner	7.08	(6.94,7.22)	0.07		8.05	(7.92,8.19)	0.07		8.04	(7.92,8.16)	0.06	
	Married	7.41	(7.30,7.52)	0.06		8.24	(8.14,8.34)	0.05		8.06	(7.96,8.17)	0.05	
	Separated	6.96	(6.73,7.20)	0.12		7.48	(7.23,7.73)	0.13		7.39	(7.16,7.63)	0.12	
	Single/Never been married	7.08	(6.96,7.20)	0.06		7.63	(7.50,7.77)	0.07		7.63	(7.50,7.75)	0.06	
	Widowed	7.10	(6.82,7.38)	0.14		8.23	(7.95,8.51)	0.14		7.91	(7.62,8.21)	0.15	
Employment	Employed for an employer	7.33	(7.23,7.44)	0.05	< .001	7.97	(7.86,8.08)	0.06	< .001	7.95	(7.85,8.05)	0.05	< .001
	Homemaker	7.20	(7.06,7.34)	0.07		8.16	(8.03,8.29)	0.07		8.07	(7.93,8.20)	0.07	
	None of these/Other	7.09	(6.70,7.47)	0.20		8.09	(7.72,8.45)	0.18		7.52	(7.07,7.96)	0.23	
	Retired	7.64	(7.38,7.91)	0.13		8.39	(8.18,8.60)	0.11		8.17	(7.97,8.37)	0.10	
	Self-employed	7.10	(6.96,7.23)	0.07		7.97	(7.83,8.12)	0.07		7.81	(7.67,7.95)	0.07	
	Student	6.95	(6.66,7.25)	0.15		7.52	(7.21,7.83)	0.16		7.46	(7.18,7.74)	0.14	
	Unemployed and looking for a job	6.77	(6.56,6.99)	0.11		7.41	(7.20,7.62)	0.11		7.44	(7.23,7.64)	0.11	
Religious service attendance	A few times a year	7.16	(7.06,7.27)	0.05	< .001	7.96	(7.86,8.06)	0.05	< .001	7.86	(7.76,7.95)	0.05	< .001
	More than once a week	7.59	(7.39,7.79)	0.10		8.48	(8.30,8.66)	0.09		8.35	(8.17,8.54)	0.09	

Variable	Category	Cantril's Ladder				Life Satisfaction				Happiness			
		Mean	95% CI	SE	Global p-value	Mean	95% CI	SE	Global p-value	Mean	95% CI	SE	Global p-value
	Never	7.01	(6.87,7.16)	0.07		7.52	(7.36,7.68)	0.08		7.50	(7.34,7.66)	0.08	
	Once a week	7.24	(7.09,7.39)	0.08		8.06	(7.92,8.19)	0.07		7.99	(7.87,8.12)	0.06	
	One to three times a month	7.21	(7.02,7.39)	0.10		8.14	(7.96,8.31)	0.09		7.93	(7.76,8.09)	0.08	
Education	Up to 8 years	7.02	(6.87,7.16)	0.07	< .001	8.14	(7.99,8.28)	0.07	0.013	7.86	(7.72,8.01)	0.07	0.548
	16+ years	7.43	(7.29,7.56)	0.07		7.86	(7.73,7.99)	0.07		7.82	(7.71,7.94)	0.06	
	9 to 15 years	7.18	(7.10,7.26)	0.04		7.95	(7.87,8.03)	0.04		7.90	(7.82,7.98)	0.04	
Immigration status	Born in another country	6.98	(6.51,7.45)	0.24	0.358	7.10	(6.59,7.62)	0.26	< .001	7.56	(7.13,8.00)	0.22	0.154
	Born in this country	7.20	(7.14,7.27)	0.03		7.99	(7.92,8.05)	0.03		7.88	(7.82,7.95)	0.03	
Religious affiliation	Buddhism	4.55	*	*	< .001	5.43	*	*	< .001	6.46	*	*	*
	Christianity	7.23	(7.16,7.30)	0.04		8.06	(7.99,8.13)	0.04		7.95	(7.89,8.02)	0.03	
	Confucianism	8.00	*	*		10.00	*	*		8.00	*	*	
	Hinduism	9.35	*	*		9.51	*	*		7.68	*	*	
	Islam	6.92	*	*		8.58	*	*		7.48	*	*	
	Judaism	6.80	*	*		7.41	*	*		8.97	*	*	
	No religion/Atheist/Agnostic	7.04	(6.87,7.21)	0.09		7.44	(7.27,7.62)	0.09		7.40	(7.23,7.57)	0.09	
	Primal, Animist, or Folk religion	6.14	(4.59,7.70)	0.65		7.17	(5.38,8.96)	0.75		7.35	(5.58,9.12)	0.74	
	Some other religion	7.33	(6.60,8.06)	0.36		7.76	(7.15,8.37)	0.30		7.98	(7.49,8.46)	0.24	
	Taoism	9.69	*	*		9.88	*	*		9.88	*	*	
	Baha'i	6.00	*	*		8.00	*	*		8.00	*	*	
	Jainism	5.00	*	*		2.00	*	*		3.00	*	*	
	Shinto	6.33	*	*		7.33	*	*		7.33	*	*	

Table S13c. Complete-case supplemental analysis of childhood predictors regression analysis results for Mexico

		Cantril's Ladder				Life Satisfaction				Happiness			
Variable	Category	Est	95% CI	SE	Global p-value	Est	95% CI	SE	Global p-value	Est	95% CI	SE	Global p-value
Relationship with mother	(Ref: Very bad/somewhat bad)				0.102				0.126				0.001
	Very good/somewhat good	0.24	(-0.05,0.53)	0.15		0.21	(-0.06,0.48)	0.14		0.42	(0.17,0.68)	0.13	
Relationship with father	(Ref: Very bad/somewhat bad)				0.749				0.276				0.531
	Very good/somewhat good	-0.03	(-0.24,0.17)	0.10		0.12	(-0.09,0.33)	0.11		0.06	(-0.13,0.24)	0.09	
Parent marital status	(Ref: Parents married)				0.688				0.369				0.228
	No, one or both of them had died	-0.04	(-0.38,0.31)	0.18		0.14	(-0.18,0.46)	0.16		-0.06	(-0.37,0.26)	0.16	
	No, they were never married	-0.14	(-0.41,0.13)	0.14		-0.09	(-0.37,0.20)	0.15		-0.25	(-0.51,0.01)	0.13	
	Yes, married	-0.02	(-0.26,0.21)	0.12		-0.08	(-0.32,0.16)	0.12		-0.11	(-0.33,0.10)	0.11	
Subjective financial status of family growing up	(Ref: Got by)				<.001				0.001				<.001
	Found it difficult	-0.31	(-0.47,-0.14)	0.09		0.04	(-0.12,0.20)	0.08		-0.06	(-0.22,0.10)	0.08	
	Found it very difficult	-0.70	(-1.04,-0.36)	0.17		-0.08	(-0.40,0.24)	0.16		-0.13	(-0.43,0.17)	0.15	
	Lived comfortably	0.33	(0.17,0.48)	0.08		0.28	(0.13,0.43)	0.08		0.24	(0.09,0.38)	0.07	
Abuse	(Ref: No)				0.152				<.001				<.001
	Yes	-0.14	(-0.33,0.05)	0.10		-0.38	(-0.56,-0.20)	0.09		-0.38	(-0.56,-0.20)	0.09	
Outsider growing up	(Ref: No)				0.224				0.003				<.001
	Yes	-0.13	(-0.34,0.08)	0.11		-0.31	(-0.51,-0.11)	0.10		-0.37	(-0.57,-0.16)	0.11	

		Cantril's Ladder				Life Satisfaction				Happiness			
Variable	Category	Est	95% CI	SE	Global p-value	Est	95% CI	SE	Global p-value	Est	95% CI	SE	Global p-value
Self-rated health growing up	(Ref: Good)				<.001				<.001				<.001
	Excellent	0.51	(0.34,0.68)	0.09		0.36	(0.20,0.52)	0.08		0.43	(0.27,0.58)	0.08	
	Fair	0.04	(-0.21,0.29)	0.13		-0.09	(-0.33,0.16)	0.13		-0.19	(-0.42,0.05)	0.12	
	Poor	0.17	(-0.32,0.65)	0.25		0.05	(-0.42,0.51)	0.24		-0.05	(-0.54,0.44)	0.25	
	Very good	0.42	(0.25,0.59)	0.09		0.21	(0.04,0.38)	0.09		0.25	(0.09,0.41)	0.08	
Immigration status	(Ref: Born in this country)				0.478				0.002				0.279
	Born in another country	-0.17	(-0.64,0.30)	0.24		-0.79	(-1.29,-0.28)	0.26		-0.23	(-0.64,0.18)	0.21	
Age 12 religious service attendance	(Ref: Never)				0.971				0.156				0.102
	At least once a week	0.02	(-0.18,0.21)	0.10		0.17	(-0.04,0.38)	0.11		0.16	(-0.04,0.35)	0.10	
	Less than once a month	-0.02	(-0.24,0.19)	0.11		0.00	(-0.22,0.22)	0.11		-0.02	(-0.23,0.19)	0.11	
	One to three times a month	0.00	(-0.21,0.22)	0.11		0.07	(-0.15,0.30)	0.11		0.02	(-0.18,0.22)	0.10	
Year of birth	(Ref: 1998-2005; current age: 18-24)				0.086				<.001				0.804
	1993-1998; age 25-29	0.18	(-0.04,0.41)	0.11		0.10	(-0.13,0.32)	0.11		0.09	(-0.12,0.30)	0.11	
	1983-1993; age 30-39	0.18	(-0.01,0.37)	0.10		0.21	(0.02,0.41)	0.10		0.14	(-0.05,0.33)	0.10	
	1973-1983; age 40-49	0.16	(-0.05,0.37)	0.11		0.28	(0.07,0.49)	0.11		0.08	(-0.12,0.29)	0.11	
	1963-1973; age 50-59	0.36	(0.13,0.60)	0.12		0.38	(0.15,0.61)	0.12		0.16	(-0.07,0.39)	0.12	
	1953-1963; age 60-69	0.30	(0.02,0.59)	0.15		0.30	(0.01,0.59)	0.15		0.07	(-0.20,0.34)	0.14	
	1943-1953; age 70-79	0.39	(0.04,0.74)	0.18		0.81	(0.52,1.10)	0.15		0.17	(-0.22,0.56)	0.20	

		Cantril's Ladder				Life Satisfaction				Happiness			
Variable	Category	Est	95% CI	SE	Global p-value	Est	95% CI	SE	Global p-value	Est	95% CI	SE	Global p-value
	1943 or earlier; age 80+	0.27	(-0.50,1.04)	0.39		0.10	(-0.54,0.74)	0.32		-0.12	(-0.63,0.40)	0.26	
Gender	(Ref: Male)				0.289				0.197				0.037
	Male	-0.09	(-0.22,0.05)	0.07		-0.04	(-0.17,0.09)	0.07		-0.13	(-0.26,0.00)	0.07	
	Other	0.64	(-0.73,2.01)	0.70		-1.51	(-3.23,0.22)	0.88		-1.12	(-2.40,0.15)	0.65	
Religious affiliation	(Ref: No religion/Atheist/Agnostic)				0.895				0.027				0.009
	Christianity	0.01	(-0.27,0.29)	0.14		0.41	(0.11,0.71)	0.15		0.36	(0.08,0.64)	0.14	
	Christianity	0.01	(-0.27,0.29)	0.14		0.41	(0.11,0.71)	0.15		0.36	(0.08,0.64)	0.14	
	Collapsed affiliations with prevalence<3%	-0.22	(-1.20,0.76)	0.50		0.47	(-0.47,1.41)	0.48		0.98	(0.24,1.71)	0.38	
Race/ethnicity	(Ref: Plurality group)				0.193				0.078				0.509
	Non-plurality groups	0.09	(-0.05,0.22)	0.07		0.11	(-0.01,0.23)	0.06		0.04	(-0.08,0.16)	0.06	

Table S13g. Complete-case supplemental analysis of sensitivity to unmeasured confounding of childhood predictors in Mexico

		Cantril's Ladder		Life Satisfaction		Happiness	
Variable	Category	E-value for Estimate	E-value for 95% CI	E-value for Estimate	E-value for 95% CI	E-value for Estimate	E-value for 95% CI
Relationship with mother	(Ref: Very bad/somewhat bad)						
	Very good/somewhat good	1.48	1.00	1.44	1.00	1.76	1.39
Relationship with father	(Ref: Very bad/somewhat bad)						
	Very good/somewhat good	1.14	1.00	1.30	1.00	1.20	1.00
Parent marital status	(Ref: Parents married)						
	No, one or both of them had died	1.15	1.00	1.34	1.00	1.20	1.00
	No, they were never married	1.33	1.00	1.25	1.00	1.52	1.00
	Yes, married	1.12	1.00	1.24	1.00	1.30	1.00
Subjective financial status of family growing up	(Ref: Got by)						
	Found it difficult	1.57	1.33	1.16	1.00	1.20	1.00
	Found it very difficult	2.11	1.64	1.23	1.00	1.34	1.00
	Lived comfortably	1.60	1.39	1.55	1.32	1.50	1.27
Abuse	(Ref: No)						
	Yes	1.34	1.00	1.68	1.43	1.70	1.45
Outsider growing up	(Ref: No)						
	Yes	1.32	1.00	1.58	1.29	1.68	1.37
Self-rated health growing up	(Ref: Good)						
	Excellent	1.86	1.63	1.65	1.43	1.77	1.55
	Fair	1.17	1.00	1.25	1.00	1.42	1.00
	Poor	1.38	1.00	1.17	1.00	1.19	1.00
	Very good	1.73	1.50	1.45	1.16	1.52	1.27
Immigration status	(Ref: Born in this country)						
	Born in another country	1.38	1.00	2.26	1.55	1.48	1.00
Age 12 religious service attendance	(Ref: Never)						
	At least once a week	1.10	1.00	1.39	1.00	1.38	1.00
	Less than once a month	1.12	1.00	1.01	1.00	1.11	1.00
	One to three times a month	1.03	1.00	1.22	1.00	1.12	1.00
Year of birth	(Ref: 1998-2005; current age: 18-24)						

Variable	Category	Cantril's Ladder		Life Satisfaction		Happiness	
		E-value for Estimate	E-value for 95% CI	E-value for Estimate	E-value for 95% CI	E-value for Estimate	E-value for 95% CI
	1993-1998; age 25-29	1.40	1.00	1.27	1.00	1.26	1.00
	1983-1993; age 30-39	1.40	1.00	1.45	1.10	1.35	1.00
	1973-1983; age 40-49	1.37	1.00	1.54	1.22	1.25	1.00
	1963-1973; age 50-59	1.65	1.32	1.68	1.36	1.38	1.00
	1953-1963; age 60-69	1.57	1.10	1.57	1.09	1.22	1.00
	1943-1953; age 70-79	1.68	1.16	2.29	1.87	1.40	1.00
	1943 or earlier; age 80+	1.52	1.00	1.27	1.00	1.31	1.00
Gender	(Ref: Male)						
	Male	1.25	1.00	1.16	1.00	1.33	1.00
	Other	2.03	1.00	3.51	1.00	2.86	1.00
Religious affiliation	(Ref: No religion/Atheist/Agnostic)						
	Christianity	1.07	1.00	1.72	1.29	1.67	1.24
	Christianity	1.07	1.00	1.72	1.29	1.67	1.24
	Collapsed affiliations with prevalence<3%	1.45	1.00	1.80	1.00	2.61	1.50
Race/ethnicity	(Ref: Plurality group)						
	Non-plurality groups	1.25	1.00	1.29	1.00	1.16	1.00

Tables S14a-g: Nigeria

Table S14a. Nationally representative descriptive statistics for Nigeria

Characteristic	N = 6,827[1]
**Age group**	
1998-2005; age 18-24	1,533 (22%)
1993-1998; age 25-29	1,193 (17%)
1983-1993; age 30-39	1,943 (28%)
1973-1983; age 40-49	1,059 (16%)
1963-1973; age 50-59	619 (9.1%)
1953-1963; age 60-69	296 (4.3%)
1943-1953; age 70-79	133 (2.0%)
1943 or earlier; age 80+	50 (0.7%)
(Missing)	0 (0%)
**Gender**	
Male	3,371 (49%)
Female	3,456 (51%)
Other	0 (<0.1%)
(Missing)	0 (0%)
**Race/Ethnicity**	
Edo	116 (1.7%)
Efik	48 (0.7%)
Fulani	266 (3.9%)
Hausa	2,342 (34%)
Ibibio	180 (2.6%)
Idoma	61 (0.9%)
Igala	77 (1.1%)
Igbo (Ibo)	1,111 (16%)
Ijaw	110 (1.6%)
Kanuri	31 (0.5%)
Other	1,014 (15%)
Tiv	198 (2.9%)
Urhobo	38 (0.6%)
Yoruba	1,230 (18%)
(Missing)	4 (<0.1%)
**Respondent Marital status**	
Married	4,065 (60%)
Separated	117 (1.7%)
Divorced	71 (1.0%)
Widowed	231 (3.4%)
Single, never married	2,289 (34%)
Domestic Partner	12 (0.2%)
(Missing)	42 (0.6%)
**Employment**	
Employed for an employer	699 (10%)
Self-employed	3,898 (57%)
Retired	178 (2.6%)
Student	650 (9.5%)
Homemaker	499 (7.3%)
Unemployed and looking for a job	684 (10%)
None of these/Other	211 (3.1%)
(Missing)	8 (0.1%)
**Religious service attendance as an adult (now)**	
More than 1/week	4,049 (59%)
1/week	1,895 (28%)
1-3/month	531 (7.8%)
A few times a year	254 (3.7%)
Never	77 (1.1%)
(Missing)	20 (0.3%)
**Education (years)**	
Up to 8 years	2,575 (38%)
9-15 years	4,120 (60%)
16+ years	130 (1.9%)
(Missing)	2 (<0.1%)
**Immigration status**	
Born in this country	6,779 (99%)

Characteristic	N = 6,827[1]
Born in another country	47 (0.7%)
(Missing)	1 (<0.1%)
**Religious affiliation as an adult (now)**	
Christianity	3,476 (51%)
Islam	3,302 (48%)
Hinduism	0 (0%)
Buddhism	0 (0%)
Judaism	0 (0%)
Sikhism	0 (0%)
Baha'i	0 (0%)
Jainism	0 (0%)
Shinto	1 (<0.1%)
Taoism	0 (0%)
Confucianism	0 (<0.1%)
Primal, Animist, or Folk religion	24 (0.3%)
Spiritism	0 (0%)
Umbanda, Candomble, and other African-derived religions	0 (0%)
Chinese folk/traditional religion	0 (0%)
Some other religion	1 (<0.1%)
No religion/Atheist/Agnostic	15 (0.2%)
(Missing)	9 (0.1%)
**Relationship with mother growing up**	
Very good	5,986 (88%)
Somewhat good	648 (9.5%)
Somewhat bad	62 (0.9%)
Very bad	18 (0.3%)
Does not apply	104 (1.5%)
(Missing)	9 (0.1%)
**Relationship with father growing up**	
Very good	5,578 (82%)
Somewhat good	924 (14%)
Somewhat bad	76 (1.1%)
Very bad	43 (0.6%)
Does not apply	177 (2.6%)
(Missing)	29 (0.4%)
**Parent marital status at age 12**	
Parents married	5,568 (82%)
Divorced	307 (4.5%)
Parents were never married	335 (4.9%)
One or both parents had died	462 (6.8%)
(Missing)	154 (2.3%)
**Subjective financial status of family growing up**	
Lived comfortably	2,192 (32%)
Got by	2,381 (35%)
Found it difficult	1,661 (24%)
Found it very difficult	563 (8.3%)
(Missing)	29 (0.4%)
**Abuse**	
Yes	880 (13%)
No	5,851 (86%)
(Missing)	96 (1.4%)
**Outsider growing up**	
Yes	669 (9.8%)
No	6,059 (89%)
(Missing)	99 (1.5%)
**Self-rated health growing up**	
Excellent	2,644 (39%)
Very good	2,613 (38%)
Good	1,152 (17%)
Fair	306 (4.5%)
Poor	98 (1.4%)
(Missing)	14 (0.2%)
**Age 12 religious service attendance**	
At least 1/week	5,907 (87%)
1-3/month	600 (8.8%)

**Characteristic**	**N = 6,827**[1]
<1/month	136 (2.0%)
Never	138 (2.0%)
(Missing)	45 (0.7%)
**Religious affiliation at age 12**	
Christianity	3,463 (51%)
Islam	3,314 (49%)
Hinduism	0 (0%)
Buddhism	0 (<0.1%)
Judaism	0 (0%)
Sikhism	0 (0%)
Baha'i	0 (0%)
Jainism	0 (0%)
Shinto	0 (0%)
Taoism	0 (0%)
Confucianism	0 (<0.1%)
Primal, Animist, or Folk religion	17 (0.3%)
Spiritism	0 (0%)
Umbanda, Candomble, and other African-derived religions	0 (0%)
Chinese folk/traditional religion	0 (0%)
Some other religion	0 (0%)
No religion/Atheist/Agnostic	19 (0.3%)
(Missing)	14 (0.2%)

[1]n (%)

Table S14b. Means by demographic category for Nigeria

Variable	Category	Cantril's Ladder				Life Satisfaction				Happiness			
		Mean	95% CI	SE	Global p-value	Mean	95% CI	SE	Global p-value	Mean	95% CI	SE	Global p-value
Age group	18-24	5.75	(5.55,5.95)	0.10	0.025	6.46	(6.26,6.67)	0.10	0.008	7.00	(6.84,7.17)	0.09	0.285
	25-29	5.84	(5.65,6.03)	0.09		6.50	(6.31,6.69)	0.10		7.11	(6.94,7.28)	0.09	
	30-39	5.64	(5.47,5.82)	0.09		6.50	(6.33,6.67)	0.09		7.02	(6.87,7.18)	0.08	
	40-49	5.47	(5.15,5.79)	0.16		6.36	(6.04,6.68)	0.16		6.91	(6.61,7.22)	0.15	
	50-59	5.91	(5.51,6.30)	0.20		6.82	(6.34,7.30)	0.24		7.34	(6.92,7.76)	0.21	
	60-69	6.04	(5.40,6.67)	0.32		6.62	(5.97,7.28)	0.33		6.93	(6.35,7.51)	0.30	
	70-79	5.64	(4.28,7.00)	0.69		6.22	(5.06,7.38)	0.59		7.74	(6.71,8.77)	0.52	
	80 or older	7.21	(6.00,8.42)	0.60		8.15	(7.28,9.03)	0.43		8.10	(6.91,9.28)	0.59	
Gender	Male	5.68	(5.52,5.84)	0.08	< .001	6.43	(6.27,6.59)	0.08	< .001	6.98	(6.85,7.12)	0.07	< .001
	Female	5.78	(5.62,5.93)	0.08		6.59	(6.44,6.74)	0.08		7.14	(7.01,7.28)	0.07	
	Other	5.00	*	*		7.00	*	*		6.00	*	*	
Marital status	Married	5.65	(5.50,5.80)	0.08	0.202	6.49	(6.34,6.64)	0.08	0.208	6.99	(6.84,7.14)	0.08	0.351
	Separated	5.62	(4.81,6.42)	0.41		5.85	(5.06,6.65)	0.40		7.01	(6.25,7.78)	0.39	
	Divorced	5.42	(4.29,6.55)	0.56		5.58	(4.25,6.90)	0.66		6.72	(5.59,7.85)	0.56	
	Widowed	6.23	(5.63,6.83)	0.30		6.67	(5.97,7.36)	0.35		7.53	(6.81,8.24)	0.36	
	Never	5.83	(5.66,6.00)	0.09		6.60	(6.41,6.78)	0.09		7.16	(7.03,7.29)	0.07	
	Domestic Partner	5.89	(1.30,10.0ǂ)	1.65		5.37	(0.15,10.0ǂ)	1.89		6.46	(1.89,10.0ǂ)	1.64	
Employment	Employed for an employer	5.97	(5.71,6.22)	0.13	0.117	6.54	(6.29,6.79)	0.13	0.307	7.24	(7.03,7.45)	0.11	0.205
	Self-employed	5.68	(5.53,5.84)	0.08		6.58	(6.43,6.74)	0.08		7.09	(6.95,7.24)	0.07	
	Retired	5.99	(5.25,6.72)	0.37		6.63	(5.75,7.52)	0.45		7.51	(6.73,8.28)	0.39	
	Student	5.75	(5.51,6.00)	0.12		6.55	(6.25,6.84)	0.15		7.07	(6.85,7.28)	0.11	
	Homemaker	5.64	(5.26,6.03)	0.19		6.47	(6.07,6.87)	0.20		6.87	(6.50,7.23)	0.19	
	Unemployed and looking for a job	5.53	(5.21,5.86)	0.17		6.11	(5.75,6.48)	0.19		6.93	(6.60,7.26)	0.17	
	None of these/Other	6.33	(5.76,6.90)	0.29		6.27	(5.57,6.97)	0.35		6.46	(5.74,7.18)	0.37	
Religious service attendance	More than 1/week	5.83	(5.68,5.99)	0.08	0.007	6.54	(6.37,6.72)	0.09	0.019	7.11	(6.96,7.26)	0.08	0.083
	1/week	5.71	(5.54,5.89)	0.09		6.66	(6.48,6.85)	0.09		7.13	(6.96,7.29)	0.09	
	1-3/month	5.23	(4.84,5.63)	0.20		6.00	(5.59,6.40)	0.21		6.63	(6.28,6.98)	0.18	
	A few times a year	5.53	(5.14,5.92)	0.20		6.04	(5.54,6.54)	0.25		6.86	(6.44,7.29)	0.21	
	Never	4.67	(3.49,5.85)	0.59		6.17	(5.20,7.15)	0.49		6.65	(5.52,7.78)	0.57	
Education	Up to 8 years	5.63	(5.40,5.85)	0.11	0.130	6.59	(6.37,6.81)	0.11	0.346	7.03	(6.80,7.25)	0.11	0.402
	9-15 years	5.78	(5.67,5.89)	0.06		6.47	(6.34,6.60)	0.07		7.10	(6.99,7.20)	0.05	

Variable	Category	Cantril's Ladder				Life Satisfaction				Happiness			
		Mean	95% CI	SE	Global p-value	Mean	95% CI	SE	Global p-value	Mean	95% CI	SE	Global p-value
	16+ years	6.04	(5.70,6.38)	0.17		6.29	(5.89,6.69)	0.20		6.82	(6.40,7.24)	0.21	
Immigration status	Born in this country	5.73	(5.61,5.85)	0.06	0.816	6.50	(6.38,6.63)	0.07	0.051	7.07	(6.96,7.18)	0.06	0.516
	Born in another country	5.56	(4.10,7.02)	0.73		7.40	(6.20,8.59)	0.57		6.60	(5.18,8.03)	0.70	
Religious affiliation	Christianity	5.80	(5.64,5.95)	0.08	< .001	6.52	(6.36,6.68)	0.08	< .001	7.26	(7.11,7.41)	0.08	< .001
	Islam	5.67	(5.47,5.86)	0.10		6.49	(6.30,6.68)	0.10		6.86	(6.71,7.02)	0.08	
	Shinto	6.00	*	*		7.00	*	*		8.00	*	*	
	Confucianism	5.00	*	*		9.00	*	*		5.00	*	*	
	Primal, Animist, or Folk religion	4.02	(2.06,5.98)	0.92		6.42	(4.03,8.81)	1.11		5.42	(3.27,7.56)	1.00	
	Some other religion	6.00	*	*		7.00	*	*		7.00	*	*	
	No religion/Atheist /Agnostic	5.49	(3.37,7.62)	0.88		7.68	(7.10,8.26)	0.24		7.24	(4.31,10.0ǂ)	1.21	
Race/Ethnicity	Hausa	5.60	(5.36,5.83)	0.12	< .001	6.51	(6.30,6.72)	0.11	< .001	6.83	(6.65,7.02)	0.10	< .001
	Yoruba	6.13	(5.91,6.34)	0.11		6.88	(6.68,7.08)	0.10		7.38	(7.16,7.59)	0.11	
	Igbo (Ibo)	5.33	(5.07,5.58)	0.13		6.52	(6.23,6.80)	0.14		7.26	(7.04,7.49)	0.12	
	Edo	6.16	(5.39,6.93)	0.39		6.93	(6.20,7.66)	0.37		7.68	(7.01,8.35)	0.34	
	Urhobo	7.14	(6.07,8.22)	0.52		6.99	(5.86,8.12)	0.54		7.65	(6.75,8.56)	0.44	
	Fulani	5.81	(5.32,6.31)	0.25		6.30	(5.67,6.93)	0.32		6.74	(6.18,7.29)	0.28	
	Kanuri	6.12	(5.00,7.25)	0.52		5.75	(5.12,6.38)	0.29		5.94	(5.07,6.81)	0.41	
	Tiv	5.98	(5.31,6.65)	0.34		6.82	(6.13,7.51)	0.35		7.35	(6.75,7.96)	0.31	
	Efik	6.36	(5.04,7.68)	0.65		5.62	(3.66,7.58)	0.96		7.46	(5.92,9.00)	0.76	
	Ijaw	5.56	(4.69,6.42)	0.43		5.29	(4.72,5.86)	0.29		6.61	(6.19,7.04)	0.22	
	Igala	5.33	(4.74,5.93)	0.30		5.82	(5.07,6.57)	0.38		6.80	(6.07,7.54)	0.37	
	Ibibio	5.82	(4.84,6.80)	0.50		6.71	(5.65,7.76)	0.53		7.64	(6.69,8.60)	0.48	
	Idoma	5.04	(3.86,6.22)	0.59		5.65	(4.46,6.84)	0.59		6.11	(4.54,7.69)	0.78	
	Other	5.84	(5.53,6.14)	0.16		6.25	(5.99,6.51)	0.13		6.98	(6.73,7.22)	0.12	

Table S14c. Childhood predictors regression analysis results for Nigeria

		Cantril's Ladder				Life Satisfaction				Happiness			
Variable	Category	Est	95% CI	SE	Global p-value	Est	95% CI	SE	Global p-value	Est	95% CI	SE	Global p-value
Relationship with mother	(Ref: Very bad/somewhat bad)				0.228				0.272				0.905
	Very good/somewhat good	0.45	(-0.29, 1.19)	0.38		0.42	(-0.34, 1.18)	0.39		0.01	(-0.62, 0.64)	0.32	
Relationship with father	(Ref: Very bad/somewhat bad)				0.649				0.457				0.228
	Very good/somewhat good	0.15	(-0.55, 0.85)	0.36		-0.24	(-0.89, 0.42)	0.33		-0.43	(-1.13, 0.28)	0.36	
Parent marital status	(Ref: Parents married)				0.008				0.004				0.002
	Divorced	0.31	(-0.19, 0.81)	0.26		0.03	(-0.39, 0.46)	0.22		0.16	(-0.23, 0.54)	0.19	
	Parents were never married	-0.40	(-0.78, -0.03)	0.19		-0.45	(-0.90, -0.01)	0.23		-0.39	(-0.79, 0.01)	0.20	
	One or both parents had died	-0.47	(-0.87, -0.07)	0.20		-0.71	(-1.15, -0.27)	0.23		-0.53	(-0.88, -0.18)	0.18	
Subjective financial status of family growing up	(Ref: Got by)				0.034				0.949				0.512
	Lived comfortably	0.06	(-0.18, 0.30)	0.12		-0.01	(-0.25, 0.24)	0.12		-0.08	(-0.32, 0.16)	0.12	
	Found it difficult	-0.10	(-0.37, 0.17)	0.14		-0.06	(-0.33, 0.20)	0.14		-0.18	(-0.43, 0.06)	0.13	
	Found it very difficult	-0.50	(-0.87, -0.12)	0.19		-0.10	(-0.55, 0.35)	0.23		-0.14	(-0.57, 0.28)	0.22	
Abuse	(Ref: No)				0.139				0.287				0.038
	Yes	-0.22	(-0.51, 0.07)	0.15		0.15	(-0.12, 0.42)	0.14		0.24	(0.01, 0.46)	0.11	
Outsider growing up	(Ref: No)				0.531				0.909				0.200
	Yes	0.11	(-0.24, 0.46)	0.18		-0.00	(-0.35, 0.35)	0.18		-0.20	(-0.52, 0.13)	0.16	
Self-rated health growing up	(Ref: Good)				0.196				0.506				0.154
	Excellent	0.26	(-0.06, 0.58)	0.16		0.20	(-0.13, 0.54)	0.17		0.15	(-0.13, 0.43)	0.14	

		Cantril's Ladder				Life Satisfaction				Happiness			
Variable	Category	Est	95% CI	SE	Global p-value	Est	95% CI	SE	Global p-value	Est	95% CI	SE	Global p-value
	Very good	0.02	(-0.26, 0.30)	0.14		0.03	(-0.32, 0.38)	0.18		-0.11	(-0.38, 0.17)	0.14	
	Fair	-0.26	(-0.77, 0.24)	0.26		-0.10	(-0.57, 0.36)	0.24		-0.18	(-0.69, 0.33)	0.26	
	Poor	-0.13	(-1.07, 0.81)	0.48		-0.02	(-0.88, 0.84)	0.44		-0.27	(-1.00, 0.45)	0.37	
Immigration status	(Ref: Born in this country)				0.974				0.019				0.379
	Born in another country	-0.02	(-1.44, 1.39)	0.72		1.01	(0.10, 1.93)	0.46		-0.62	(-1.99, 0.76)	0.70	
Age 12 religious service attendance	(Ref: Never)				0.799				0.033				0.272
	At least 1/week	0.20	(-0.86, 1.26)	0.54		0.95	(0.25, 1.66)	0.36		0.63	(-0.06, 1.32)	0.35	
	1-3/month	0.05	(-1.06, 1.16)	0.57		0.81	(-0.01, 1.62)	0.42		0.65	(-0.13, 1.43)	0.40	
	< 1/month	0.06	(-1.14, 1.25)	0.61		0.51	(-0.40, 1.42)	0.46		0.67	(-0.15, 1.49)	0.42	
Year of birth	(Ref: 1998-2005; current age: 18-24)				0.021				0.007				0.127
	1993-1998; age 25-29	0.09	(-0.14, 0.31)	0.12		0.07	(-0.17, 0.30)	0.12		0.14	(-0.08, 0.37)	0.11	
	1983-1993; age 30-39	-0.13	(-0.36, 0.09)	0.11		0.04	(-0.20, 0.28)	0.12		0.01	(-0.21, 0.23)	0.11	
	1973-1983; age 40-49	-0.27	(-0.65, 0.10)	0.19		-0.04	(-0.41, 0.34)	0.19		-0.10	(-0.44, 0.24)	0.17	
	1963-1973; age 50-59	0.18	(-0.25, 0.60)	0.21		0.42	(-0.07, 0.91)	0.25		0.36	(-0.07, 0.78)	0.22	
	1953-1963; age 60-69	0.28	(-0.35, 0.91)	0.32		0.21	(-0.48, 0.90)	0.35		-0.14	(-0.74, 0.46)	0.31	
	1943-1953; age 70-79	-0.11	(-1.47, 1.25)	0.69		-0.27	(-1.41, 0.88)	0.58		0.70	(-0.34, 1.74)	0.53	
	1943 or earlier; age 80+	1.31	(0.21, 2.42)	0.56		1.60	(0.76, 2.44)	0.43		0.95	(0.02, 1.89)	0.48	
Gender	(Ref: Male)				0.025				0.107				<.001
	Female	0.01	(-0.18, 0.21)	0.10		0.15	(-0.04, 0.34)	0.10		0.09	(-0.07, 0.25)	0.08	
	Other	-0.54	(-0.95, -0.13)	0.21		0.39	(0.00, 0.78)	0.20		-1.39	(-1.72, -1.06)	0.17	
Religious affiliation	(Ref: Christianity)				0.588				0.723				0.406
	Islam	0.05	(-0.28, 0.39)	0.17		0.01	(-0.32, 0.33)	0.17		-0.19	(-0.49, 0.11)	0.15	
	Collapsed affiliations with prevalence<3%	-0.77	(-2.37, 0.83)	0.81		0.39	(-0.57, 1.35)	0.49		-0.18	(-1.45, 1.09)	0.65	

Variable	Category	Cantril's Ladder				Life Satisfaction				Happiness			
		Est	95% CI	SE	Global p-value	Est	95% CI	SE	Global p-value	Est	95% CI	SE	Global p-value
Race/ethnicity	(Ref: Plurality group)				0.113				0.825				0.157
	Non-plurality groups	0.25	(-0.11, 0.62)	0.18		0.02	(-0.28, 0.31)	0.15		0.20	(-0.12, 0.51)	0.16	

Table S14d. Sensitivity to unmeasured confounding of childhood predictors in Nigeria

Variable	Category	Cantril's Ladder		Life Satisfaction		Happiness	
		E-value for Estimate	E-value for 95% CI	E-value for Estimate	E-value for 95% CI	E-value for Estimate	E-value for 95% CI
Relationship with mother	(Ref: Very bad/somewhat bad)						
	Very good/somewhat good	1.59	1.00	1.55	1.00	1.05	1.00
Relationship with father	(Ref: Very bad/somewhat bad)						
	Very good/somewhat good	1.28	1.00	1.37	1.00	1.60	1.00
Parent marital status	(Ref: Parents married)						
	Divorced	1.45	1.00	1.11	1.00	1.30	1.00
	Parents were never married	1.54	1.12	1.58	1.05	1.56	1.00
	One or both parents had died	1.60	1.18	1.82	1.40	1.70	1.33
Subjective financial status of family growing up	(Ref: Got by)						
	Lived comfortably	1.16	1.00	1.04	1.00	1.20	1.00
	Found it difficult	1.22	1.00	1.17	1.00	1.33	1.00
	Found it very difficult	1.63	1.25	1.22	1.00	1.29	1.00
Abuse	(Ref: No)						
	Yes	1.36	1.00	1.27	1.00	1.39	1.07
Outsider growing up	(Ref: No)						
	Yes	1.23	1.00	1.03	1.00	1.35	1.00
Self-rated health growing up	(Ref: Good)						
	Excellent	1.40	1.00	1.34	1.00	1.29	1.00
	Very good	1.09	1.00	1.11	1.00	1.24	1.00
	Fair	1.40	1.00	1.22	1.00	1.33	1.00
	Poor	1.25	1.00	1.08	1.00	1.44	1.00
Immigration status	(Ref: Born in this country)						
	Born in another country	1.09	1.00	2.11	1.23	1.79	1.00
Age 12 religious service attendance	(Ref: Never)						
	At least 1/week	1.33	1.00	2.05	1.38	1.80	1.00
	1-3/month	1.15	1.00	1.91	1.00	1.82	1.00
	< 1/month	1.15	1.00	1.63	1.00	1.85	1.00
Year of birth	(Ref: 1998-2005; current age: 18-24)						
	1993-1998; age 25-29	1.20	1.00	1.17	1.00	1.29	1.00
	1983-1993; age 30-39	1.26	1.00	1.12	1.00	1.06	1.00
	1973-1983; age 40-49	1.41	1.00	1.12	1.00	1.23	1.00

Variable	Category	Cantril's Ladder		Life Satisfaction		Happiness	
		E-value for Estimate	E-value for 95% CI	E-value for Estimate	E-value for 95% CI	E-value for Estimate	E-value for 95% CI
	1963-1973; age 50-59	1.31	1.00	1.54	1.00	1.52	1.00
	1953-1963; age 60-69	1.42	1.00	1.34	1.00	1.28	1.00
	1943-1953; age 70-79	1.23	1.00	1.40	1.00	1.88	1.00
	1943 or earlier; age 80+	2.44	1.35	2.72	1.87	2.15	1.10
Gender	(Ref: Male)						
	Female	1.07	1.00	1.27	1.00	1.22	1.00
	Other	1.68	1.26	1.52	1.03	2.64	2.26
Religious affiliation	(Ref: Christianity)						
	Islam	1.15	1.00	1.05	1.00	1.34	1.00
	Collapsed affiliations with prevalence<3%	1.89	1.00	1.52	1.00	1.33	1.00
Race/ethnicity	(Ref: Plurality group)						
	Non-plurality groups	1.39	1.00	1.08	1.00	1.35	1.00

Table S14e. Complete-case supplemental analysis of means by demographic category for Nigeria

		Cantril's Ladder				Life Satisfaction				Happiness			
Variable	Category	Mean	95% CI	SE	Global p-value	Mean	95% CI	SE	Global p-value	Mean	95% CI	SE	Global p-value
Age group	18-24	6.01	(5.82,6.21)	0.10	0.461	6.86	(6.68,7.05)	0.09	0.015	7.24	(7.08,7.41)	0.08	0.057
	25-29	6.07	(5.89,6.25)	0.09		6.77	(6.59,6.95)	0.09		7.19	(7.03,7.36)	0.08	
	30-39	6.01	(5.85,6.17)	0.08		6.92	(6.77,7.07)	0.08		7.25	(7.10,7.39)	0.07	
	40-49	5.98	(5.71,6.25)	0.14		6.92	(6.65,7.19)	0.14		7.27	(7.03,7.51)	0.12	
	50-59	6.18	(5.81,6.55)	0.19		7.32	(6.94,7.70)	0.20		7.74	(7.39,8.09)	0.18	
	60-69	6.34	(5.76,6.91)	0.29		7.08	(6.41,7.75)	0.34		7.09	(6.51,7.68)	0.30	
	70-79	6.14	(4.90,7.39)	0.63		6.61	(5.54,7.69)	0.54		8.00	(7.10,8.90)	0.45	
	80 or older	7.21	(6.00,8.42)	0.60		8.15	(7.28,9.03)	0.43		8.10	(6.91,9.28)	0.59	
Gender	Female	6.10	(5.96,6.24)	0.07	< .001	6.97	(6.82,7.11)	0.07	0.318	7.36	(7.23,7.48)	0.06	< .001
	Male	6.01	(5.87,6.16)	0.07		6.89	(6.75,7.03)	0.07		7.24	(7.11,7.36)	0.06	
	Other	5.00	(5.00,5.00)	0.00		7.00	(7.00,7.00)	0.00		6.00	(6.00,6.00)	0.00	
Marital status	Divorced	5.60	(4.45,6.76)	0.58	0.215	6.66	(5.75,7.57)	0.45	0.815	6.81	(5.67,7.94)	0.57	0.761
	Domestic partner	6.38	(1.87,10.89)	1.62		6.49	(1.32,11.66)	1.86		6.62	(2.04,11.20)	1.65	
	Married	5.99	(5.86,6.12)	0.07		6.91	(6.78,7.04)	0.07		7.27	(7.14,7.41)	0.07	
	Separated	6.22	(5.54,6.91)	0.35		6.59	(5.89,7.29)	0.35		7.45	(6.85,8.05)	0.30	
	Single/Never been married	6.13	(5.96,6.30)	0.09		6.99	(6.83,7.16)	0.09		7.32	(7.19,7.45)	0.07	
	Widowed	6.55	(5.97,7.14)	0.30		6.93	(6.17,7.70)	0.39		7.63	(6.93,8.34)	0.36	
Employment	Employed for an employer	6.31	(6.10,6.53)	0.11	0.189	7.02	(6.81,7.23)	0.11	0.745	7.50	(7.32,7.69)	0.10	0.137
	Homemaker	5.91	(5.57,6.25)	0.17		6.67	(6.30,7.04)	0.19		7.05	(6.68,7.41)	0.18	
	None of these/Other	6.45	(5.88,7.01)	0.29		6.79	(6.09,7.50)	0.36		7.23	(6.61,7.85)	0.31	
	Retired	6.29	(5.56,7.01)	0.37		7.03	(6.27,7.79)	0.38		7.77	(7.11,8.44)	0.34	
	Self-employed	6.03	(5.89,6.16)	0.07		6.97	(6.82,7.11)	0.07		7.30	(7.16,7.43)	0.07	
	Student	6.01	(5.77,6.25)	0.12		6.89	(6.61,7.17)	0.14		7.23	(7.02,7.43)	0.10	
	Unemployed and looking for a job	5.95	(5.68,6.22)	0.14		6.87	(6.58,7.16)	0.15		7.25	(6.97,7.53)	0.14	
Religious service attendance	A few times a year	5.83	(5.44,6.22)	0.20	0.037	6.56	(6.12,7.00)	0.22	0.137	7.15	(6.80,7.50)	0.18	0.057
	More than once a week	6.17	(6.04,6.31)	0.07		6.97	(6.81,7.12)	0.08		7.34	(7.20,7.49)	0.07	
	Never	5.84	(4.71,6.97)	0.57		7.00	(6.13,7.88)	0.44		7.53	(6.64,8.41)	0.44	
	Once a week	5.96	(5.79,6.13)	0.09		6.97	(6.80,7.14)	0.09		7.33	(7.18,7.48)	0.08	

Variable	Category	Cantril's Ladder				Life Satisfaction				Happiness			
		Mean	95% CI	SE	Global p-value	Mean	95% CI	SE	Global p-value	Mean	95% CI	SE	Global p-value
	One to three times a month	5.68	(5.34,6.02)	0.17		6.63	(6.34,6.93)	0.15		6.88	(6.59,7.16)	0.14	
Education	Up to 8 years	5.97	(5.77,6.16)	0.10	0.279	6.93	(6.73,7.13)	0.10	0.119	7.25	(7.04,7.46)	0.11	0.328
	16+ years	6.19	(5.87,6.51)	0.16		6.52	(6.13,6.92)	0.20		7.07	(6.69,7.45)	0.19	
	9 to 15 years	6.11	(6.01,6.21)	0.05		6.94	(6.82,7.06)	0.06		7.34	(7.24,7.43)	0.05	
Immigration status	Born in another country	6.33	(5.19,7.47)	0.57	0.630	7.58	(6.71,8.46)	0.43	0.133	6.60	(5.18,8.03)	0.70	0.323
	Born in this country	6.06	(5.95,6.17)	0.06		6.92	(6.81,7.04)	0.06		7.30	(7.20,7.41)	0.05	
Religious affiliation	Christianity	6.21	(6.07,6.35)	0.07	< .001	7.10	(6.96,7.25)	0.07	< .001	7.59	(7.46,7.72)	0.07	< .001
	Confucianism	5.00	*	*		9.00	*	*		5.00	*	*	
	Islam	5.92	(5.74,6.09)	0.09		6.74	(6.57,6.92)	0.09		7.00	(6.85,7.16)	0.08	
	No religion/Atheist/Agnostic	5.63	(3.48,7.78)	0.89		7.68	(7.10,8.26)	0.24		7.24	(4.31,10.17)	1.21	
	Primal, Animist, or Folk religion	4.84	(3.06,6.63)	0.83		7.59	(5.76,9.42)	0.85		6.43	(4.12,8.73)	1.07	
	Some other religion	6.00	*	*		7.00	*	*		7.00	*	*	
	Shinto	6.00	*	*		7.00	*	*		8.00	*	*	

Table S14f. Complete-case supplemental analysis of childhood predictors regression analysis results for Nigeria

		Cantril's Ladder				Life Satisfaction				Happiness			
Variable	Category	Est	95% CI	SE	Global p-value	Est	95% CI	SE	Global p-value	Est	95% CI	SE	Global p-value
Relationship with mother	(Ref: Very bad/somewhat bad)				0.891				0.696				0.607
	Very good/somewhat good	-0.04	(-0.62,0.54)	0.29		-0.12	(-0.74,0.49)	0.31		-0.14	(-0.69,0.40)	0.28	
Relationship with father	(Ref: Very bad/somewhat bad)				0.611				0.484				0.012
	Very good/somewhat good	0.15	(-0.43,0.74)	0.30		-0.18	(-0.68,0.32)	0.25		-0.57	(-1.01,-0.13)	0.22	
Parent marital status	(Ref: Parents married)				0.022				0.004				<.001
	No, one or both of them had died	-0.46	(-1.03,0.11)	0.29		-0.43	(-1.00,0.13)	0.29		-0.62	(-1.13,-0.10)	0.26	
	No, they were never married	-0.58	(-1.12,-0.04)	0.28		-0.15	(-0.69,0.38)	0.27		-0.42	(-0.91,0.07)	0.25	
	Yes, married	-0.13	(-0.59,0.34)	0.24		0.18	(-0.23,0.60)	0.21		-0.05	(-0.42,0.32)	0.19	
Subjective financial status of family growing up	(Ref: Got by)				0.016				0.603				0.578
	Found it difficult	-0.15	(-0.39,0.09)	0.12		-0.06	(-0.28,0.16)	0.11		-0.11	(-0.33,0.12)	0.11	
	Found it very difficult	-0.43	(-0.78,-0.08)	0.18		-0.15	(-0.56,0.26)	0.21		-0.13	(-0.51,0.25)	0.19	
	Lived comfortably	0.14	(-0.09,0.36)	0.11		0.10	(-0.11,0.32)	0.11		0.05	(-0.17,0.28)	0.12	
Abuse	(Ref: No)				0.165				0.078				0.065
	Yes	-0.20	(-0.48,0.08)	0.14		0.23	(-0.02,0.49)	0.13		0.21	(-0.01,0.43)	0.11	
Outsider growing up	(Ref: No)				0.955				0.768				0.117
	Yes	0.01	(-0.32,0.34)	0.17		-0.05	(-0.36,0.26)	0.16		-0.24	(-0.53,0.06)	0.15	

		Cantril's Ladder				Life Satisfaction				Happiness			
Variable	Category	Est	95% CI	SE	Global p-value	Est	95% CI	SE	Global p-value	Est	95% CI	SE	Global p-value
Self-rated health growing up	(Ref: Good)				0.117				0.796				0.416
	Excellent	0.25	(-0.06,0.55)	0.15		0.09	(-0.22,0.41)	0.16		0.10	(-0.15,0.35)	0.13	
	Fair	-0.30	(-0.77,0.17)	0.24		-0.12	(-0.60,0.35)	0.24		-0.16	(-0.65,0.33)	0.25	
	Poor	0.08	(-0.73,0.88)	0.41		0.23	(-0.49,0.95)	0.37		-0.22	(-0.88,0.44)	0.34	
	Very good	0.08	(-0.18,0.35)	0.14		-0.01	(-0.32,0.29)	0.16		-0.07	(-0.31,0.17)	0.12	
Immigration status	(Ref: Born in this country)				0.429				0.064				0.214
	Born in another country	0.43	(-0.63,1.49)	0.54		0.75	(-0.04,1.54)	0.40		-0.89	(-2.29,0.51)	0.71	
Age 12 religious service attendance	(Ref: Never)				0.861				0.029				0.250
	At least once a week	0.14	(-0.96,1.25)	0.56		1.11	(0.34,1.88)	0.39		0.64	(-0.00,1.29)	0.33	
	Less than once a month	-0.00	(-1.16,1.16)	0.59		0.66	(-0.20,1.51)	0.44		0.69	(-0.08,1.46)	0.39	
	One to three times a month	0.02	(-1.13,1.18)	0.59		0.96	(0.14,1.79)	0.42		0.61	(-0.11,1.33)	0.37	
Year of birth	(Ref: 1998-2005; current age: 18-24)				0.590				0.035				0.056
	1993-1998; age 25-29	0.05	(-0.17,0.27)	0.11		-0.07	(-0.28,0.15)	0.11		-0.02	(-0.24,0.20)	0.11	
	1983-1993; age 30-39	-0.04	(-0.26,0.17)	0.11		0.04	(-0.17,0.24)	0.10		-0.02	(-0.23,0.18)	0.10	
	1973-1983; age 40-49	-0.06	(-0.39,0.26)	0.17		0.05	(-0.26,0.37)	0.16		-0.02	(-0.30,0.26)	0.14	
	1963-1973; age 50-59	0.14	(-0.27,0.54)	0.21		0.46	(0.06,0.85)	0.20		0.47	(0.11,0.84)	0.19	
	1953-1963; age 60-69	0.25	(-0.32,0.81)	0.29		0.13	(-0.54,0.81)	0.35		-0.30	(-0.87,0.28)	0.30	
	1943-1953; age 70-79	0.07	(-1.15,1.29)	0.62		-0.35	(-1.44,0.74)	0.55		0.66	(-0.23,1.54)	0.45	

Variable	Category	Cantril's Ladder				Life Satisfaction				Happiness			
		Est	95% CI	SE	Global p-value	Est	95% CI	SE	Global p-value	Est	95% CI	SE	Global p-value
	1943 or earlier; age 80+	0.96	(-0.16,2.08)	0.57		1.11	(0.28,1.93)	0.42		0.60	(-0.38,1.57)	0.50	
Gender	(Ref: Male)				<.001				0.108				<.001
	Male	-0.01	(-0.18,0.17)	0.09		-0.06	(-0.22,0.11)	0.08		-0.04	(-0.17,0.10)	0.07	
	Other	-1.02	(-1.40,-0.64)	0.19		-0.35	(-0.68,-0.02)	0.17		-1.75	(-2.04,-1.46)	0.15	
Religious affiliation	(Ref: Christianity)				0.879				0.103				0.022
	Islam	-0.07	(-0.36,0.22)	0.15		-0.22	(-0.51,0.06)	0.15		-0.40	(-0.68,-0.12)	0.14	
	Collapsed affiliations with prevalence<3 %	-0.05	(-1.35,1.24)	0.66		0.54	(-0.24,1.33)	0.40		0.20	(-0.88,1.28)	0.55	
Race/ethnicity	(Ref: Plurality group)				0.066				0.363				0.205
	Non-plurality groups	0.30	(-0.02,0.61)	0.16		0.13	(-0.15,0.41)	0.14		0.19	(-0.10,0.49)	0.15	

Table S14g. Complete-case supplemental analysis of sensitivity to unmeasured confounding of childhood predictors in Nigeria

		Cantril's Ladder		Life Satisfaction		Happiness	
Variable	Category	E-value for Estimate	E-value for 95% CI	E-value for Estimate	E-value for 95% CI	E-value for Estimate	E-value for 95% CI
Relationship with mother	(Ref: Very bad/somewhat bad)						
	Very good/somewhat good	1.14	1.00	1.27	1.00	1.31	1.00
Relationship with father	(Ref: Very bad/somewhat bad)						
	Very good/somewhat good	1.30	1.00	1.34	1.00	1.82	1.29
Parent marital status	(Ref: Parents married)						
	No, one or both of them had died	1.65	1.00	1.63	1.00	1.87	1.25
	No, they were never married	1.77	1.13	1.31	1.00	1.65	1.00
	Yes, married	1.27	1.00	1.35	1.00	1.16	1.00
Subjective financial status of family growing up	(Ref: Got by)						
	Found it difficult	1.30	1.00	1.18	1.00	1.26	1.00
	Found it very difficult	1.61	1.20	1.31	1.00	1.29	1.00
	Lived comfortably	1.28	1.00	1.24	1.00	1.17	1.00
Abuse	(Ref: No)						
	Yes	1.36	1.00	1.41	1.00	1.39	1.00
Outsider growing up	(Ref: No)						
	Yes	1.06	1.00	1.15	1.00	1.43	1.00
Self-rated health growing up	(Ref: Good)						
	Excellent	1.42	1.00	1.23	1.00	1.25	1.00
	Fair	1.47	1.00	1.27	1.00	1.33	1.00
	Poor	1.20	1.00	1.40	1.00	1.40	1.00
	Very good	1.21	1.00	1.08	1.00	1.19	1.00
Immigration status	(Ref: Born in this country)						
	Born in another country	1.62	1.00	1.98	1.00	2.21	1.00
Age 12 religious service attendance	(Ref: Never)						
	At least once a week	1.29	1.00	2.41	1.54	1.91	1.00
	Less than once a month	1.01	1.00	1.88	1.00	1.97	1.00
	One to three times a month	1.11	1.00	2.23	1.29	1.87	1.00
Year of birth	(Ref: 1998-2005; current age: 18-24)						

Variable	Category	Cantril's Ladder		Life Satisfaction		Happiness	
		E-value for Estimate	E-value for 95% CI	E-value for Estimate	E-value for 95% CI	E-value for Estimate	E-value for 95% CI
	1993-1998; age 25-29	1.15	1.00	1.18	1.00	1.10	1.00
	1983-1993; age 30-39	1.15	1.00	1.13	1.00	1.10	1.00
	1973-1983; age 40-49	1.18	1.00	1.17	1.00	1.10	1.00
	1963-1973; age 50-59	1.28	1.00	1.66	1.18	1.71	1.26
	1953-1963; age 60-69	1.42	1.00	1.29	1.00	1.50	1.00
	1943-1953; age 70-79	1.19	1.00	1.54	1.00	1.93	1.00
	1943 or earlier; age 80+	2.19	1.00	2.41	1.47	1.85	1.00
Gender	(Ref: Male)						
	Male	1.06	1.00	1.17	1.00	1.13	1.00
	Other	2.26	1.84	1.55	1.11	3.44	2.98
Religious affiliation	(Ref: Christianity)						
	Islam	1.19	1.00	1.40	1.00	1.62	1.28
	Collapsed affiliations with prevalence<3%	1.16	1.00	1.76	1.00	1.39	1.00
Race/ethnicity	(Ref: Plurality group)						
	Non-plurality groups	1.47	1.00	1.28	1.00	1.37	1.00

Tables S15a-g: Philippines

Table S15a. Nationally representative descriptive statistics for Philippines

Characteristic	N = 5,292[1]
**Age group**	
1998-2005; age 18-24	1,073 (20%)
1993-1998; age 25-29	695 (13%)
1983-1993; age 30-39	1,160 (22%)
1973-1983; age 40-49	972 (18%)
1963-1973; age 50-59	732 (14%)
1953-1963; age 60-69	495 (9.4%)
1943-1953; age 70-79	143 (2.7%)
1943 or earlier; age 80+	23 (0.4%)
(Missing)	0 (0%)
**Gender**	
Male	2,625 (50%)
Female	2,643 (50%)
Other	13 (0.2%)
(Missing)	11 (0.2%)
**Race/Ethnicity**	
Aeta	1 (<0.1%)
Badjao	2 (<0.1%)
Bicolano/Bikolano	300 (5.7%)
Cebuano	656 (12%)
Chinese-Filipino	3 (<0.1%)
Igorot	42 (0.8%)
Ilocano/Ilokano	429 (8.1%)
Ilonggo/Hiligaynon	428 (8.1%)
Kapampangan	107 (2.0%)
Maguindanaoan	84 (1.6%)
Mangyan	2 (<0.1%)
Maranao	39 (0.7%)
Masbateno	54 (1.0%)
Other	244 (4.6%)
Pangasinense	107 (2.0%)
Tagalog	1,691 (32%)
Tausug	94 (1.8%)
Visayan/Bisaya	739 (14%)
Waray	216 (4.1%)
Zamboangueno	51 (1.0%)
(Missing)	3 (<0.1%)
**Respondent Marital status**	
Married	2,385 (45%)
Separated	249 (4.7%)
Divorced	9 (0.2%)
Widowed	274 (5.2%)
Single, never married	1,206 (23%)
Domestic Partner	1,152 (22%)
(Missing)	16 (0.3%)
**Employment**	
Employed for an employer	1,350 (26%)
Self-employed	1,379 (26%)
Retired	158 (3.0%)
Student	585 (11%)
Homemaker	1,049 (20%)
Unemployed and looking for a job	658 (12%)
None of these/Other	113 (2.1%)
(Missing)	0 (0%)
**Religious service attendance as an adult (now)**	
More than 1/week	844 (16%)
1/week	1,929 (36%)
1-3/month	1,374 (26%)
A few times a year	929 (18%)
Never	210 (4.0%)
(Missing)	6 (0.1%)

Characteristic	N = 5,292[1]
**Education (years)**	
Up to 8 years	1,188 (22%)
9-15 years	3,722 (70%)
16+ years	381 (7.2%)
(Missing)	1 (<0.1%)
**Immigration status**	
Born in this country	5,284 (100%)
Born in another country	8 (0.1%)
(Missing)	0 (0%)
**Religious affiliation as an adult (now)**	
Christianity	4,914 (93%)
Islam	297 (5.6%)
Hinduism	0 (0%)
Buddhism	4 (<0.1%)
Judaism	4 (<0.1%)
Sikhism	0 (0%)
Baha'i	1 (<0.1%)
Jainism	0 (0%)
Shinto	0 (0%)
Taoism	0 (0%)
Confucianism	0 (0%)
Primal, Animist, or Folk religion	5 (<0.1%)
Spiritism	0 (0%)
Umbanda, Candomble, and other African-derived religions	0 (0%)
Chinese folk/traditional religion	0 (0%)
Some other religion	35 (0.7%)
No religion/Atheist/Agnostic	23 (0.4%)
(Missing)	9 (0.2%)
**Relationship with mother growing up**	
Very good	3,333 (63%)
Somewhat good	1,703 (32%)
Somewhat bad	124 (2.3%)
Very bad	39 (0.7%)
Does not apply	59 (1.1%)
(Missing)	35 (0.7%)
**Relationship with father growing up**	
Very good	3,443 (65%)
Somewhat good	1,429 (27%)
Somewhat bad	159 (3.0%)
Very bad	58 (1.1%)
Does not apply	108 (2.0%)
(Missing)	95 (1.8%)
**Parent marital status at age 12**	
Parents married	4,575 (86%)
Divorced	64 (1.2%)
Parents were never married	517 (9.8%)
One or both parents had died	51 (1.0%)
(Missing)	85 (1.6%)
**Subjective financial status of family growing up**	
Lived comfortably	937 (18%)
Got by	3,006 (57%)
Found it difficult	1,055 (20%)
Found it very difficult	291 (5.5%)
(Missing)	3 (<0.1%)
**Abuse**	
Yes	420 (7.9%)
No	4,837 (91%)
(Missing)	35 (0.7%)
**Outsider growing up**	
Yes	395 (7.5%)
No	4,884 (92%)
(Missing)	13 (0.2%)
**Self-rated health growing up**	
Excellent	1,041 (20%)
Very good	559 (11%)

**Characteristic**	**N = 5,292**[1]
Good	2,174 (41%)
Fair	1,246 (24%)
Poor	272 (5.1%)
(Missing)	0 (<0.1%)
**Age 12 religious service attendance**	
At least 1/week	2,453 (46%)
1-3/month	1,699 (32%)
<1/month	892 (17%)
Never	201 (3.8%)
(Missing)	47 (0.9%)
**Religious affiliation at age 12**	
Christianity	4,968 (94%)
Islam	276 (5.2%)
Hinduism	0 (0%)
Buddhism	1 (<0.1%)
Judaism	0 (0%)
Sikhism	4 (<0.1%)
Baha'i	1 (<0.1%)
Jainism	0 (0%)
Shinto	0 (0%)
Taoism	0 (0%)
Confucianism	0 (0%)
Primal, Animist, or Folk religion	14 (0.3%)
Spiritism	0 (0%)
Umbanda, Candomble, and other African-derived religions	0 (0%)
Chinese folk/traditional religion	0 (0%)
Some other religion	9 (0.2%)
No religion/Atheist/Agnostic	9 (0.2%)
(Missing)	11 (0.2%)

[1]n (%)

Table S15b. Means by demographic category for Philippines

		Cantril's Ladder				Life Satisfaction				Happiness			
Variable	Category	Mean	95% CI	SE	Global p-value	Mean	95% CI	SE	Global p-value	Mean	95% CI	SE	Global p-value
Age group	18-24	6.61	(6.44,6.79)	0.09	< .001	7.72	(7.55,7.89)	0.09	0.002	7.39	(7.23,7.55)	0.08	0.246
	25-29	6.56	(6.33,6.78)	0.11		7.74	(7.50,7.97)	0.12		7.48	(7.23,7.74)	0.13	
	30-39	6.32	(6.15,6.48)	0.08		7.62	(7.47,7.77)	0.08		7.36	(7.21,7.51)	0.08	
	40-49	6.08	(5.91,6.24)	0.08		7.33	(7.16,7.51)	0.09		7.18	(7.02,7.34)	0.08	
	50-59	6.51	(6.29,6.73)	0.11		7.28	(7.03,7.52)	0.13		7.20	(6.98,7.42)	0.11	
	60-69	6.25	(5.93,6.57)	0.16		7.13	(6.76,7.50)	0.19		7.31	(6.98,7.64)	0.17	
	70-79	6.22	(5.69,6.75)	0.27		7.55	(6.88,8.21)	0.34		7.37	(6.91,7.82)	0.23	
	80 or older	6.57	(5.19,7.96)	0.64		6.21	(4.23,8.18)	0.91		8.13	(6.88,9.38)	0.58	
Gender	Male	6.31	(6.17,6.45)	0.07	0.192	7.46	(7.32,7.60)	0.07	0.081	7.30	(7.16,7.44)	0.07	0.325
	Female	6.46	(6.37,6.55)	0.05		7.55	(7.45,7.64)	0.05		7.35	(7.27,7.44)	0.04	
	Other	6.19	(4.70,7.69)	0.64		6.44	(5.21,7.68)	0.53		6.56	(5.19,7.92)	0.58	
Marital status	Married	6.37	(6.25,6.50)	0.06	0.298	7.52	(7.39,7.66)	0.07	0.002	7.36	(7.24,7.49)	0.06	0.119
	Separated	6.19	(5.84,6.53)	0.18		6.83	(6.47,7.20)	0.18		6.83	(6.48,7.18)	0.18	
	Divorced	5.35	(1.39,9.32)	0.82		6.45	(2.37,10.0‡)	0.85		7.05	(2.82,10.0‡)	0.88	
	Widowed	6.41	(6.06,6.77)	0.18		7.42	(7.07,7.78)	0.18		7.45	(7.11,7.78)	0.17	
	Never	6.51	(6.34,6.67)	0.08		7.53	(7.37,7.69)	0.08		7.30	(7.14,7.46)	0.08	
	Domestic Partner	6.32	(6.16,6.48)	0.08		7.60	(7.45,7.75)	0.08		7.36	(7.20,7.52)	0.08	
Employment	Employed for an employer	6.41	(6.24,6.58)	0.09	0.799	7.64	(7.48,7.81)	0.08	0.057	7.38	(7.21,7.55)	0.09	0.360
	Self-employed	6.34	(6.17,6.50)	0.08		7.39	(7.21,7.57)	0.09		7.35	(7.19,7.51)	0.08	
	Retired	6.44	(5.88,7.00)	0.29		7.77	(7.22,8.32)	0.28		7.51	(6.95,8.08)	0.29	
	Student	6.51	(6.30,6.71)	0.10		7.58	(7.39,7.78)	0.10		7.31	(7.13,7.49)	0.09	
	Homemaker	6.33	(6.18,6.49)	0.08		7.57	(7.41,7.72)	0.08		7.34	(7.19,7.49)	0.08	
	Unemployed and looking for a job	6.36	(6.10,6.61)	0.13		7.28	(7.01,7.55)	0.14		7.20	(6.95,7.45)	0.13	
	None of these/Other	6.52	(5.99,7.05)	0.27		7.11	(6.63,7.59)	0.24		6.81	(6.30,7.32)	0.26	
Religious service attendance	More than 1/week	6.25	(6.04,6.46)	0.11	0.002	7.74	(7.53,7.96)	0.11	< .001	7.42	(7.21,7.64)	0.11	< .001
	1/week	6.57	(6.44,6.70)	0.07		7.69	(7.55,7.82)	0.07		7.58	(7.45,7.70)	0.06	
	1-3/month	6.35	(6.19,6.52)	0.08		7.48	(7.32,7.65)	0.08		7.24	(7.09,7.39)	0.08	
	A few times a year	6.21	(6.05,6.37)	0.08		7.13	(6.95,7.31)	0.09		7.00	(6.82,7.18)	0.09	
	Never	6.16	(5.63,6.69)	0.27		6.61	(6.08,7.15)	0.27		6.65	(6.11,7.19)	0.27	
Education	Up to 8 years	6.38	(6.17,6.59)	0.11	0.046	7.43	(7.22,7.65)	0.11	0.423	7.31	(7.11,7.51)	0.10	0.415
	9-15 years	6.35	(6.27,6.44)	0.04		7.51	(7.42,7.60)	0.05		7.31	(7.22,7.40)	0.05	

Variable	Category	Cantril's Ladder				Life Satisfaction				Happiness			
		Mean	95% CI	SE	Global p-value	Mean	95% CI	SE	Global p-value	Mean	95% CI	SE	Global p-value
	16+ years	6.68	(6.43,6.93)	0.13		7.66	(7.39,7.92)	0.13		7.48	(7.24,7.73)	0.12	
Immigration status	Born in this country	6.39	(6.31,6.46)	0.04	0.159	7.50	(7.42,7.59)	0.04	0.074	7.33	(7.24,7.41)	0.04	0.105
	Born in another country	5.55	(3.88,7.22)	0.59		6.03	(3.67,8.38)	0.82		6.08	(3.91,8.26)	0.76	
Religious affiliation	Christianity	6.39	(6.31,6.47)	0.04	< .001	7.51	(7.42,7.60)	0.05	< .001	7.32	(7.24,7.41)	0.04	< .001
	Islam	6.20	(5.75,6.65)	0.23		7.40	(7.01,7.79)	0.20		7.40	(7.05,7.75)	0.18	
	Buddhism	4.03	*	*		7.93	*	*		8.58	*	*	
	Judaism	5.87	*	*		8.65	*	*		5.13	*	*	
	Baha'i	6.00	*	*		6.00	*	*		6.00	*	*	
	Primal, Animist, or Folk religion	8.71	(6.63,10.0‡)	0.96		2.92	(0.18,5.65)	1.38		3.79	(1.45,6.14)	1.19	
	Some other religion	6.85	(5.95,7.75)	0.44		7.65	(6.94,8.36)	0.34		7.52	(6.80,8.23)	0.35	
	No religion/Atheist /Agnostic	7.07	(5.64,8.49)	0.65		8.10	(7.09,9.12)	0.47		8.06	(7.04,9.08)	0.47	
Race/Ethnicity	Tagalog	6.57	(6.42,6.71)	0.07	< .001	7.63	(7.50,7.76)	0.07	< .001	7.44	(7.31,7.57)	0.06	< .001
	Cebuano	6.39	(6.18,6.60)	0.11		7.23	(7.02,7.45)	0.11		7.36	(7.13,7.60)	0.12	
	Ilocano/Ilokano	6.30	(6.02,6.58)	0.14		7.69	(7.42,7.97)	0.14		7.55	(7.23,7.87)	0.16	
	Visayan/Bisaya	6.48	(6.30,6.67)	0.09		7.56	(7.35,7.77)	0.11		7.27	(7.06,7.49)	0.11	
	Ilonggo/Hiligaynon	6.42	(6.16,6.69)	0.14		7.44	(7.07,7.81)	0.19		7.17	(6.85,7.49)	0.16	
	Bicolano/Bikolano	6.01	(5.63,6.39)	0.19		7.31	(6.98,7.64)	0.17		6.97	(6.66,7.27)	0.16	
	Waray	5.86	(5.50,6.22)	0.18		6.90	(6.43,7.36)	0.24		6.72	(6.36,7.08)	0.18	
	Tausug	6.51	(5.61,7.41)	0.45		7.83	(7.09,8.57)	0.37		7.85	(7.38,8.32)	0.24	
	Maranao	5.77	(4.87,6.68)	0.43		6.65	(5.84,7.46)	0.38		6.67	(6.12,7.21)	0.26	
	Maguindanaoan	5.94	(5.25,6.64)	0.35		7.65	(7.13,8.17)	0.26		7.55	(6.99,8.12)	0.28	
	Chinese-Filipino	8.04	*	*		8.52	*	*		8.52	*	*	
	Kapampangan	6.26	(5.69,6.83)	0.29		7.64	(7.25,8.04)	0.20		7.33	(6.85,7.80)	0.24	
	Pangasinense	6.33	(5.46,7.21)	0.44		8.27	(7.84,8.69)	0.21		7.59	(6.95,8.22)	0.32	
	Zamboangueno	6.73	(6.26,7.20)	0.23		7.99	(6.79,9.19)	0.59		7.51	(6.92,8.11)	0.29	
	Masbateno	6.22	(5.31,7.12)	0.44		6.53	(5.16,7.91)	0.68		7.59	(6.79,8.38)	0.39	
	Aeta	7.06	*	*		3.39	*	*		4.47	*	*	
	Igorot	6.31	(6.03,6.59)	0.13		7.55	(6.72,8.38)	0.40		7.41	(6.61,8.22)	0.39	

Variable	Category	Cantril’s Ladder				Life Satisfaction				Happiness			
		Mean	95% CI	SE	Global p-value	Mean	95% CI	SE	Global p-value	Mean	95% CI	SE	Global p-value
	Mangyan	3.68	*	*		5.95	*	*		4.32	*	*	
	Badjao	5.04	*	*		3.59	*	*		7.03	*	*	
	Other	6.04	(5.68,6.41)	0.18		7.42	(6.88,7.95)	0.27		7.08	(6.63,7.53)	0.23	

Table S15c. Childhood predictors regression analysis results for Philippines

		Cantril's Ladder				Life Satisfaction				Happiness			
Variable	Category	Est	95% CI	SE	Global p-value	Est	95% CI	SE	Global p-value	Est	95% CI	SE	Global p-value
Relationship with mother	(Ref: Very bad/somewhat bad)				0.523				0.574				0.205
	Very good/somewhat good	0.17	(-0.36, 0.69)	0.27		0.13	(-0.34, 0.61)	0.24		0.27	(-0.15, 0.69)	0.22	
Relationship with father	(Ref: Very bad/somewhat bad)				0.240				0.269				0.574
	Very good/somewhat good	0.21	(-0.15, 0.57)	0.18		0.23	(-0.19, 0.66)	0.22		0.10	(-0.26, 0.46)	0.18	
Parent marital status	(Ref: Parents married)				0.889				0.018				0.008
	Divorced	-0.16	(-0.90, 0.58)	0.38		-0.21	(-0.94, 0.53)	0.38		-0.44	(-0.99, 0.12)	0.28	
	Parents were never married	-0.08	(-0.33, 0.18)	0.13		-0.00	(-0.25, 0.25)	0.13		-0.01	(-0.27, 0.25)	0.13	
	One or both parents had died	0.02	(-0.91, 0.95)	0.48		0.96	(0.33, 1.60)	0.32		0.87	(0.25, 1.49)	0.31	
Subjective financial status of family growing up	(Ref: Got by)				<.001				<.001				<.001
	Lived comfortably	0.37	(0.17, 0.58)	0.10		0.42	(0.20, 0.65)	0.11		0.32	(0.11, 0.53)	0.11	
	Found it difficult	-0.25	(-0.47, -0.03)	0.11		-0.30	(-0.52, -0.08)	0.11		-0.31	(-0.53, -0.08)	0.12	
	Found it very difficult	-0.14	(-0.54, 0.26)	0.20		-0.62	(-1.04, -0.21)	0.21		-0.75	(-1.14, -0.35)	0.20	
Abuse	(Ref: No)				0.022				0.064				0.564
	Yes	-0.40	(-0.75, -0.06)	0.18		-0.34	(-0.71, 0.02)	0.19		-0.10	(-0.44, 0.24)	0.17	
Outsider growing up	(Ref: No)				0.562				0.315				0.063
	Yes	0.10	(-0.24, 0.44)	0.17		-0.19	(-0.55, 0.18)	0.19		-0.32	(-0.66, 0.02)	0.17	
Self-rated health growing up	(Ref: Good)				0.073				<.001				<.001
	Excellent	0.17	(-0.05, 0.39)	0.11		0.14	(-0.06, 0.34)	0.10		0.28	(0.08, 0.47)	0.10	

Variable	Category	Cantril's Ladder Est	95% CI	SE	Global p-value	Life Satisfaction Est	95% CI	SE	Global p-value	Happiness Est	95% CI	SE	Global p-value
	Very good	-0.05	(-0.32, 0.23)	0.14		0.15	(-0.12, 0.42)	0.14		0.31	(0.08, 0.54)	0.12	
	Fair	-0.14	(-0.34, 0.07)	0.10		-0.54	(-0.76, -0.31)	0.11		-0.35	(-0.56, -0.15)	0.10	
	Poor	-0.32	(-0.71, 0.06)	0.20		-0.50	(-0.93, -0.08)	0.21		-0.42	(-0.81, -0.03)	0.20	
Immigration status	(Ref: Born in this country)				0.185				0.073				0.045
	Born in another country	-0.90	(-2.24, 0.43)	0.68		-1.50	(-3.14, 0.14)	0.84		-1.28	(-2.53, -0.03)	0.64	
Age 12 religious service attendance	(Ref: Never)				0.842				0.937				0.702
	At least 1/week	-0.10	(-0.55, 0.34)	0.23		-0.12	(-0.74, 0.50)	0.32		-0.04	(-0.59, 0.52)	0.28	
	1-3/month	-0.17	(-0.63, 0.29)	0.23		-0.14	(-0.75, 0.48)	0.31		-0.02	(-0.57, 0.53)	0.28	
	< 1/month	-0.11	(-0.58, 0.36)	0.24		-0.08	(-0.73, 0.57)	0.33		0.09	(-0.48, 0.66)	0.29	
Year of birth	(Ref: 1998-2005; current age: 18-24)				0.002				0.067				0.539
	1993-1998; age 25-29	-0.06	(-0.33, 0.21)	0.14		0.01	(-0.27, 0.29)	0.14		0.10	(-0.19, 0.39)	0.15	
	1983-1993; age 30-39	-0.26	(-0.51, -0.01)	0.13		-0.04	(-0.26, 0.19)	0.11		0.06	(-0.14, 0.26)	0.10	
	1973-1983; age 40-49	-0.48	(-0.71, -0.24)	0.12		-0.28	(-0.53, -0.03)	0.13		-0.08	(-0.31, 0.15)	0.12	
	1963-1973; age 50-59	-0.03	(-0.32, 0.26)	0.15		-0.31	(-0.61, -0.00)	0.16		-0.01	(-0.28, 0.25)	0.14	
	1953-1963; age 60-69	-0.26	(-0.63, 0.11)	0.19		-0.43	(-0.83, -0.02)	0.21		0.13	(-0.23, 0.50)	0.19	
	1943-1953; age 70-79	-0.35	(-0.92, 0.22)	0.29		-0.02	(-0.70, 0.67)	0.35		0.18	(-0.32, 0.68)	0.25	
	1943 or earlier; age 80+	0.09	(-1.25, 1.43)	0.68		-1.33	(-3.03, 0.37)	0.87		1.00	(-0.25, 2.24)	0.63	
Gender	(Ref: Male)				0.199				0.077				0.327
	Female	0.15	(-0.02, 0.32)	0.09		0.08	(-0.08, 0.24)	0.08		0.06	(-0.10, 0.22)	0.08	
	Other	-0.10	(-1.26, 1.06)	0.59		-1.02	(-2.07, 0.04)	0.54		-0.70	(-1.87, 0.47)	0.60	
Religious affiliation	(Ref: Christianity)				0.629				0.937				0.447
	Islam	-0.04	(-0.52, 0.44)	0.24		-0.06	(-0.46, 0.35)	0.21		0.19	(-0.18, 0.56)	0.19	
	Collapsed affiliations with prevalence<3%	0.79	(-0.84, 2.41)	0.83		-0.16	(-1.52, 1.20)	0.69		0.47	(-0.69, 1.63)	0.59	

Variable	Category	Cantril's Ladder				Life Satisfaction				Happiness			
		Est	95% CI	SE	Global p-value	Est	95% CI	SE	Global p-value	Est	95% CI	SE	Global p-value
Race/ethnicity	(Ref: Plurality group)				0.016				0.306				0.189
	Non-plurality groups	-0.21	(-0.38, -0.04)	0.09		-0.09	(-0.25, 0.08)	0.08		-0.10	(-0.26, 0.06)	0.08	

Table S15d. Sensitivity to unmeasured confounding of childhood predictors in Philippines

		Cantril's Ladder		Life Satisfaction		Happiness	
Variable	Category	E-value for Estimate	E-value for 95% CI	E-value for Estimate	E-value for 95% CI	E-value for Estimate	E-value for 95% CI
Relationship with mother	(Ref: Very bad/somewhat bad)						
	Very good/somewhat good	1.33	1.00	1.28	1.00	1.46	1.00
Relationship with father	(Ref: Very bad/somewhat bad)						
	Very good/somewhat good	1.38	1.00	1.41	1.00	1.24	1.00
Parent marital status	(Ref: Parents married)						
	Divorced	1.32	1.00	1.38	1.00	1.66	1.00
	Parents were never married	1.21	1.00	1.03	1.00	1.08	1.00
	One or both parents had died	1.09	1.00	2.22	1.52	2.16	1.45
Subjective financial status of family growing up	(Ref: Got by)						
	Lived comfortably	1.57	1.33	1.62	1.37	1.52	1.25
	Found it difficult	1.43	1.13	1.48	1.21	1.51	1.22
	Found it very difficult	1.29	1.00	1.84	1.38	2.01	1.56
Abuse	(Ref: No)						
	Yes	1.61	1.17	1.53	1.00	1.24	1.00
Outsider growing up	(Ref: No)						
	Yes	1.24	1.00	1.35	1.00	1.52	1.00
Self-rated health growing up	(Ref: Good)						
	Excellent	1.34	1.00	1.30	1.00	1.47	1.22
	Very good	1.15	1.00	1.31	1.00	1.51	1.21
	Fair	1.29	1.00	1.74	1.50	1.56	1.31
	Poor	1.51	1.00	1.71	1.22	1.64	1.13
Immigration status	(Ref: Born in this country)						
	Born in another country	2.17	1.00	2.91	1.00	2.68	1.13
Age 12 religious service attendance	(Ref: Never)						
	At least 1/week	1.24	1.00	1.27	1.00	1.13	1.00
	1-3/month	1.34	1.00	1.29	1.00	1.09	1.00
	< 1/month	1.26	1.00	1.21	1.00	1.23	1.00
Year of birth	(Ref: 1998-2005; current age: 18-24)						
	1993-1998; age 25-29	1.17	1.00	1.07	1.00	1.24	1.00
	1983-1993; age 30-39	1.44	1.06	1.13	1.00	1.17	1.00
	1973-1983; age 40-49	1.69	1.42	1.46	1.12	1.21	1.00
	1963-1973; age 50-59	1.12	1.00	1.49	1.03	1.07	1.00

Variable	Category	Cantril's Ladder		Life Satisfaction		Happiness	
		E-value for Estimate	E-value for 95% CI	E-value for Estimate	E-value for 95% CI	E-value for Estimate	E-value for 95% CI
	1953-1963; age 60-69	1.44	1.00	1.63	1.11	1.29	1.00
	1943-1953; age 70-79	1.54	1.00	1.09	1.00	1.36	1.00
	1943 or earlier; age 80+	1.22	1.00	2.68	1.00	2.31	1.00
Gender	(Ref: Male)						
	Female	1.31	1.00	1.21	1.00	1.18	1.00
	Other	1.24	1.00	2.29	1.00	1.96	1.00
Religious affiliation	(Ref: Christianity)						
	Islam	1.13	1.00	1.17	1.00	1.36	1.00
	Collapsed affiliations with prevalence<3%	2.03	1.00	1.32	1.00	1.69	1.00
Race/ethnicity	(Ref: Plurality group)						
	Non-plurality groups	1.38	1.14	1.22	1.00	1.25	1.00

Table S15e. Complete-case supplemental analysis of means by demographic category for Philippines

		Cantril's Ladder				Life Satisfaction				Happiness			
Variable	Category	Mean	95% CI	SE	Global p-value	Mean	95% CI	SE	Global p-value	Mean	95% CI	SE	Global p-value
Age group	18-24	6.65	(6.47,6.82)	0.09	< .001	7.83	(7.67,7.98)	0.08	0.006	7.48	(7.32,7.63)	0.08	0.026
	25-29	6.57	(6.35,6.80)	0.11		7.82	(7.59,8.05)	0.12		7.58	(7.35,7.82)	0.12	
	30-39	6.35	(6.19,6.52)	0.08		7.78	(7.64,7.92)	0.07		7.46	(7.32,7.60)	0.07	
	40-49	6.11	(5.95,6.28)	0.08		7.45	(7.29,7.61)	0.08		7.31	(7.16,7.46)	0.08	
	50-59	6.56	(6.34,6.77)	0.11		7.50	(7.30,7.71)	0.10		7.31	(7.09,7.52)	0.11	
	60-69	6.33	(6.02,6.64)	0.16		7.52	(7.23,7.81)	0.15		7.63	(7.38,7.89)	0.13	
	70-79	6.25	(5.73,6.78)	0.27		8.07	(7.62,8.51)	0.22		7.72	(7.34,8.11)	0.20	
	80 or older	6.57	(5.19,7.96)	0.64		7.49	(6.39,8.59)	0.51		8.57	(7.60,9.55)	0.45	
Gender	Female	6.51	(6.42,6.60)	0.05	0.129	7.71	(7.63,7.80)	0.04	0.045	7.46	(7.38,7.55)	0.04	0.273
	Male	6.34	(6.20,6.47)	0.07		7.65	(7.52,7.77)	0.06		7.45	(7.33,7.58)	0.06	
	Other	6.21	(4.70,7.71)	0.64		6.43	(5.17,7.70)	0.54		6.52	(5.14,7.89)	0.59	
Marital status	Divorced	5.35	(1.39,9.32)	0.82	0.273	6.87	(3.15,10.60)	0.77	0.004	7.52	(3.67,11.37)	0.80	0.074
	Domestic partner	6.34	(6.18,6.51)	0.08		7.71	(7.57,7.86)	0.07		7.45	(7.29,7.60)	0.08	
	Married	6.43	(6.31,6.55)	0.06		7.75	(7.64,7.86)	0.06		7.51	(7.40,7.62)	0.06	
	Separated	6.21	(5.86,6.56)	0.18		7.00	(6.66,7.35)	0.18		7.00	(6.67,7.33)	0.17	
	Single/Never been married	6.54	(6.37,6.70)	0.08		7.63	(7.49,7.78)	0.07		7.41	(7.26,7.56)	0.07	
	Widowed	6.45	(6.09,6.80)	0.18		7.73	(7.43,8.03)	0.15		7.65	(7.32,7.99)	0.17	
Employment	Employed for an employer	6.44	(6.27,6.61)	0.09	0.805	7.75	(7.60,7.90)	0.08	0.149	7.49	(7.34,7.65)	0.08	0.036
	Homemaker	6.39	(6.24,6.54)	0.08		7.73	(7.60,7.86)	0.07		7.52	(7.38,7.66)	0.07	
	None of these/Other	6.52	(5.99,7.05)	0.27		7.18	(6.71,7.66)	0.24		6.92	(6.43,7.41)	0.25	
	Retired	6.48	(5.93,7.04)	0.28		7.99	(7.52,8.45)	0.23		7.84	(7.44,8.24)	0.20	
	Self-employed	6.36	(6.20,6.53)	0.08		7.65	(7.51,7.79)	0.07		7.47	(7.32,7.61)	0.07	
	Student	6.56	(6.35,6.76)	0.10		7.64	(7.45,7.83)	0.10		7.36	(7.17,7.54)	0.09	
	Unemployed and looking for a job	6.41	(6.17,6.66)	0.12		7.54	(7.31,7.78)	0.12		7.36	(7.14,7.58)	0.11	
Religious service attendance	A few times a year	6.24	(6.08,6.40)	0.08	0.002	7.31	(7.15,7.48)	0.08	< .001	7.12	(6.94,7.29)	0.09	< .001
	More than once a week	6.29	(6.09,6.50)	0.10		7.96	(7.77,8.14)	0.09		7.53	(7.33,7.72)	0.10	
	Never	6.21	(5.68,6.74)	0.27		6.99	(6.51,7.47)	0.24		7.00	(6.54,7.46)	0.23	
	Once a week	6.60	(6.47,6.72)	0.06		7.81	(7.69,7.94)	0.06		7.68	(7.56,7.80)	0.06	

Variable	Category	Cantril's Ladder				Life Satisfaction				Happiness			
		Mean	95% CI	SE	Global p-value	Mean	95% CI	SE	Global p-value	Mean	95% CI	SE	Global p-value
	One to three times a month	6.41	(6.25,6.57)	0.08		7.66	(7.53,7.80)	0.07		7.40	(7.27,7.52)	0.06	
Education	Up to 8 years	6.47	(6.26,6.68)	0.11	0.042	7.76	(7.58,7.95)	0.10	0.441	7.60	(7.43,7.77)	0.09	0.101
	16+ years	6.69	(6.44,6.94)	0.13		7.73	(7.46,8.00)	0.14		7.51	(7.27,7.75)	0.12	
	9 to 15 years	6.38	(6.29,6.47)	0.04		7.65	(7.57,7.72)	0.04		7.41	(7.32,7.49)	0.04	
Immigration status	Born in another country	5.55	(3.88,7.22)	0.59	0.141	6.03	(3.67,8.38)	0.82	0.046	6.08	(3.91,8.26)	0.76	0.073
	Born in this country	6.42	(6.35,6.50)	0.04		7.68	(7.61,7.75)	0.04		7.46	(7.38,7.54)	0.04	
Religious affiliation	Buddhism	4.03	*	*	< .001	7.93	*	*	< .001	8.58	*	*	< .001
	Christianity	6.43	(6.35,6.51)	0.04		7.68	(7.60,7.76)	0.04		7.45	(7.38,7.53)	0.04	
	Islam	6.23	(5.79,6.68)	0.23		7.72	(7.37,8.07)	0.18		7.54	(7.23,7.86)	0.16	
	Judaism	5.87	*	*		8.65	*	*		5.13	*	*	
	No religion/Atheist/Agnostic	7.07	(5.64,8.49)	0.65		8.08	(7.06,9.11)	0.47		8.27	(7.37,9.17)	0.41	
	Primal, Animist, or Folk religion	8.63	(6.66,10.60)	1.00		2.92	(0.18,5.65)	1.38		3.79	(1.45,6.14)	1.19	
	Some other religion	6.88	(5.98,7.78)	0.43		7.65	(6.94,8.36)	0.34		7.52	(6.80,8.23)	0.35	
	Baha'i	6.00	*	*		6.00	*	*		6.00	*	*	

Table S15f. Complete-case supplemental analysis of childhood predictors regression analysis results for Philippines

		Cantril's Ladder				Life Satisfaction				Happiness			
Variable	Category	Est	95% CI	SE	Global p-value	Est	95% CI	SE	Global p-value	Est	95% CI	SE	Global p-value
Relationship with mother	(Ref: Very bad/somewhat bad)				0.887				0.784				0.288
	Very good/somewhat good	0.04	(-0.47,0.55)	0.26		0.06	(-0.37,0.49)	0.22		0.22	(-0.18,0.62)	0.21	
Relationship with father	(Ref: Very bad/somewhat bad)				0.322				0.913				0.729
	Very good/somewhat good	0.18	(-0.17,0.53)	0.18		0.02	(-0.32,0.36)	0.17		0.06	(-0.28,0.41)	0.18	
Parent marital status	(Ref: Parents married)				0.717				0.084				0.037
	No, one or both of them had died	0.54	(-0.57,1.64)	0.56		1.10	(0.18,2.01)	0.47		1.12	(0.36,1.88)	0.39	
	No, they were never married	0.12	(-0.61,0.86)	0.38		0.35	(-0.41,1.10)	0.38		0.43	(-0.12,0.99)	0.28	
	Yes, married	0.22	(-0.49,0.94)	0.37		0.36	(-0.36,1.08)	0.37		0.47	(-0.03,0.97)	0.25	
Subjective financial status of family growing up	(Ref: Got by)				<.001				<.001				<.001
	Found it difficult	-0.20	(-0.42,0.01)	0.11		-0.17	(-0.37,0.03)	0.10		-0.21	(-0.42,0.00)	0.11	
	Found it very difficult	0.05	(-0.32,0.42)	0.19		-0.20	(-0.56,0.15)	0.18		-0.33	(-0.66,0.00)	0.17	
	Lived comfortably	0.40	(0.20,0.60)	0.10		0.42	(0.22,0.62)	0.10		0.33	(0.14,0.53)	0.10	
Abuse	(Ref: No)				0.062				0.333				0.588
	Yes	-0.32	(-0.65,0.02)	0.17		-0.15	(-0.46,0.16)	0.16		-0.08	(-0.38,0.22)	0.15	
Outsider growing up	(Ref: No)				0.784				0.478				0.244
	Yes	0.05	(-0.29,0.38)	0.17		-0.11	(-0.43,0.20)	0.16		-0.17	(-0.45,0.11)	0.14	

		Cantril's Ladder				Life Satisfaction				Happiness			
Variable	Category	Est	95% CI	SE	Global p-value	Est	95% CI	SE	Global p-value	Est	95% CI	SE	Global p-value
Self-rated health growing up	(Ref: Good)				0.067				<.001				<.001
	Excellent	0.16	(-0.06,0.38)	0.11		0.20	(0.02,0.37)	0.09		0.26	(0.08,0.45)	0.09	
	Fair	-0.14	(-0.34,0.06)	0.10		-0.42	(-0.63,-0.21)	0.11		-0.31	(-0.50,-0.13)	0.09	
	Poor	-0.37	(-0.74,0.01)	0.19		-0.52	(-0.88,-0.16)	0.18		-0.54	(-0.90,-0.19)	0.18	
	Very good	-0.06	(-0.33,0.21)	0.14		0.13	(-0.11,0.36)	0.12		0.21	(-0.02,0.45)	0.12	
Immigration status	(Ref: Born in this country)				0.172				0.040				0.032
	Born in another country	-0.93	(-2.27,0.40)	0.68		-1.70	(-3.32,-0.08)	0.83		-1.41	(-2.70,-0.12)	0.66	
Age 12 religious service attendance	(Ref: Never)				0.755				0.747				0.923
	At least once a week	-0.08	(-0.53,0.36)	0.23		-0.23	(-0.72,0.25)	0.25		0.04	(-0.47,0.55)	0.26	
	Less than once a month	-0.08	(-0.54,0.38)	0.23		-0.24	(-0.75,0.27)	0.26		0.07	(-0.45,0.60)	0.27	
	One to three times a month	-0.17	(-0.63,0.29)	0.23		-0.26	(-0.75,0.22)	0.25		0.00	(-0.50,0.51)	0.26	
Year of birth	(Ref: 1998-2005; current age: 18-24)				0.002				0.046				0.030
	1993-1998; age 25-29	-0.07	(-0.33,0.20)	0.14		-0.01	(-0.28,0.27)	0.14		0.11	(-0.16,0.38)	0.14	
	1983-1993; age 30-39	-0.26	(-0.50,-0.01)	0.13		0.01	(-0.19,0.22)	0.10		0.05	(-0.14,0.23)	0.09	
	1973-1983; age 40-49	-0.48	(-0.71,-0.24)	0.12		-0.27	(-0.50,-0.05)	0.12		-0.07	(-0.28,0.15)	0.11	
	1963-1973; age 50-59	-0.03	(-0.30,0.25)	0.14		-0.21	(-0.47,0.06)	0.13		-0.03	(-0.28,0.22)	0.13	
	1953-1963; age 60-69	-0.23	(-0.60,0.13)	0.19		-0.17	(-0.52,0.17)	0.18		0.32	(0.00,0.63)	0.16	
	1943-1953; age 70-79	-0.34	(-0.91,0.23)	0.29		0.38	(-0.09,0.86)	0.24		0.40	(-0.04,0.83)	0.22	

		Cantril's Ladder				Life Satisfaction				Happiness			
Variable	Category	Est	95% CI	SE	Global p-value	Est	95% CI	SE	Global p-value	Est	95% CI	SE	Global p-value
	1943 or earlier; age 80+	0.01	(-1.33,1.36)	0.69		-0.28	(-1.23,0.66)	0.48		1.24	(0.25,2.24)	0.51	
Gender	(Ref: Male)				0.129				0.040				0.323
	Male	-0.17	(-0.33,0.00)	0.09		-0.07	(-0.22,0.07)	0.07		-0.01	(-0.16,0.13)	0.07	
	Other	-0.33	(-1.50,0.85)	0.60		-1.25	(-2.30,-0.21)	0.53		-0.86	(-2.00,0.28)	0.58	
Religious affiliation	(Ref: Christianity)				0.641				0.730				0.505
	Islam	-0.06	(-0.54,0.41)	0.24		0.11	(-0.23,0.46)	0.17		0.18	(-0.15,0.50)	0.17	
	Collapsed affiliations with prevalence<3%	0.76	(-0.88,2.39)	0.83		-0.29	(-1.62,1.04)	0.68		0.27	(-0.84,1.39)	0.57	
Race/ethnicity	(Ref: Plurality group)				0.012				0.731				0.475
	Non-plurality groups	-0.22	(-0.39,-0.05)	0.09		-0.03	(-0.18,0.12)	0.08		-0.05	(-0.20,0.09)	0.07	

Table S15g. Complete-case supplemental analysis of sensitivity to unmeasured confounding of childhood predictors in Philippines

		Cantril's Ladder		Life Satisfaction		Happiness	
Variable	Category	E-value for Estimate	E-value for 95% CI	E-value for Estimate	E-value for 95% CI	E-value for Estimate	E-value for 95% CI
Relationship with mother	(Ref: Very bad/somewhat bad)						
	Very good/somewhat good	1.14	1.00	1.19	1.00	1.43	1.00
Relationship with father	(Ref: Very bad/somewhat bad)						
	Very good/somewhat good	1.35	1.00	1.10	1.00	1.19	1.00
Parent marital status	(Ref: Parents married)						
	No, one or both of them had died	1.77	1.00	2.55	1.38	2.60	1.61
	No, they were never married	1.27	1.00	1.58	1.00	1.70	1.00
	Yes, married	1.40	1.00	1.60	1.00	1.74	1.00
Subjective financial status of family growing up	(Ref: Got by)						
	Found it difficult	1.38	1.00	1.36	1.00	1.41	1.00
	Found it very difficult	1.16	1.00	1.40	1.00	1.57	1.00
	Lived comfortably	1.61	1.37	1.67	1.42	1.57	1.32
Abuse	(Ref: No)						
	Yes	1.52	1.00	1.33	1.00	1.23	1.00
Outsider growing up	(Ref: No)						
	Yes	1.15	1.00	1.28	1.00	1.35	1.00
Self-rated health growing up	(Ref: Good)						
	Excellent	1.32	1.00	1.39	1.11	1.48	1.22
	Fair	1.30	1.00	1.67	1.41	1.55	1.30
	Poor	1.57	1.00	1.79	1.34	1.83	1.38
	Very good	1.18	1.00	1.30	1.00	1.42	1.00
Immigration status	(Ref: Born in this country)						
	Born in another country	2.22	1.00	3.51	1.22	3.05	1.29
Age 12 religious service attendance	(Ref: Never)						
	At least once a week	1.22	1.00	1.44	1.00	1.15	1.00
	Less than once a month	1.21	1.00	1.44	1.00	1.21	1.00
	One to three times a month	1.34	1.00	1.48	1.00	1.04	1.00
Year of birth	(Ref: 1998-2005; current age: 18-24)						

Variable	Category	Cantril's Ladder		Life Satisfaction		Happiness	
		E-value for Estimate	E-value for 95% CI	E-value for Estimate	E-value for 95% CI	E-value for Estimate	E-value for 95% CI
	1993-1998; age 25-29	1.19	1.00	1.05	1.00	1.27	1.00
	1983-1993; age 30-39	1.44	1.07	1.08	1.00	1.16	1.00
	1973-1983; age 40-49	1.70	1.43	1.49	1.16	1.20	1.00
	1963-1973; age 50-59	1.11	1.00	1.41	1.00	1.13	1.00
	1953-1963; age 60-69	1.42	1.00	1.36	1.00	1.55	1.04
	1943-1953; age 70-79	1.54	1.00	1.63	1.00	1.65	1.00
	1943 or earlier; age 80+	1.08	1.00	1.50	1.00	2.79	1.47
Gender	(Ref: Male)						
	Male	1.33	1.00	1.21	1.00	1.08	1.00
	Other	1.52	1.00	2.78	1.41	2.24	1.00
Religious affiliation	(Ref: Christianity)						
	Islam	1.19	1.00	1.28	1.00	1.37	1.00
	Collapsed affiliations with prevalence<3%	2.01	1.00	1.51	1.00	1.50	1.00
Race/ethnicity	(Ref: Plurality group)						
	Non-plurality groups	1.40	1.16	1.12	1.00	1.18	1.00

Tables S16a-g: Poland

Table S16a. Nationally representative descriptive statistics for Poland

**Characteristic**	**N = 10,389**[1]
**Age group**	
1998-2005; age 18-24	955 (9.2%)
1993-1998; age 25-29	761 (7.3%)
1983-1993; age 30-39	2,159 (21%)
1973-1983; age 40-49	1,956 (19%)
1963-1973; age 50-59	1,670 (16%)
1953-1963; age 60-69	1,909 (18%)
1943-1953; age 70-79	833 (8.0%)
1943 or earlier; age 80+	145 (1.4%)
(Missing)	1 (<0.1%)
**Gender**	
Male	4,974 (48%)
Female	5,387 (52%)
Other	3 (<0.1%)
(Missing)	26 (0.2%)
**Race/Ethnicity**	
Belarussian	2 (<0.1%)
German	4 (<0.1%)
Kashubians	3 (<0.1%)
Other	4 (<0.1%)
Polish	10,309 (99%)
Silesia	14 (0.1%)
Ukrainian	38 (0.4%)
(Missing)	14 (0.1%)
**Respondent Marital status**	
Married	6,065 (58%)
Separated	111 (1.1%)
Divorced	529 (5.1%)
Widowed	990 (9.5%)
Single, never married	1,811 (17%)
Domestic Partner	504 (4.8%)
(Missing)	379 (3.6%)
**Employment**	
Employed for an employer	5,837 (56%)
Self-employed	686 (6.6%)
Retired	2,434 (23%)
Student	515 (5.0%)
Homemaker	338 (3.3%)
Unemployed and looking for a job	284 (2.7%)
None of these/Other	169 (1.6%)
(Missing)	126 (1.2%)
**Religious service attendance as an adult (now)**	
More than 1/week	305 (2.9%)
1/week	3,263 (31%)
1-3/month	2,081 (20%)
A few times a year	3,064 (29%)
Never	1,597 (15%)
(Missing)	78 (0.8%)
**Education (years)**	
Up to 8 years	1,238 (12%)
9-15 years	6,130 (59%)
16+ years	3,020 (29%)
(Missing)	1 (<0.1%)
**Immigration status**	
Born in this country	10,258 (99%)
Born in another country	108 (1.0%)
(Missing)	23 (0.2%)
**Religious affiliation as an adult (now)**	
Christianity	9,378 (90%)
Islam	2 (<0.1%)
Hinduism	0 (0%)

Characteristic	N = 10,389[1]
Buddhism	2 (<0.1%)
Judaism	0 (0%)
Sikhism	1 (<0.1%)
Baha'i	0 (0%)
Jainism	3 (<0.1%)
Shinto	1 (<0.1%)
Taoism	0 (0%)
Confucianism	0 (0%)
Primal, Animist, or Folk religion	11 (0.1%)
Spiritism	0 (0%)
Umbanda, Candomble, and other African-derived religions	0 (0%)
Chinese folk/traditional religion	0 (0%)
Some other religion	0 (0%)
No religion/Atheist/Agnostic	942 (9.1%)
(Missing)	50 (0.5%)
**Relationship with mother growing up**	
Very good	4,879 (47%)
Somewhat good	4,973 (48%)
Somewhat bad	285 (2.7%)
Very bad	58 (0.6%)
Does not apply	80 (0.8%)
(Missing)	112 (1.1%)
**Relationship with father growing up**	
Very good	4,231 (41%)
Somewhat good	4,984 (48%)
Somewhat bad	516 (5.0%)
Very bad	78 (0.7%)
Does not apply	407 (3.9%)
(Missing)	173 (1.7%)
**Parent marital status at age 12**	
Parents married	8,972 (86%)
Divorced	587 (5.7%)
Parents were never married	193 (1.9%)
One or both parents had died	313 (3.0%)
(Missing)	324 (3.1%)
**Subjective financial status of family growing up**	
Lived comfortably	1,384 (13%)
Got by	6,257 (60%)
Found it difficult	2,133 (21%)
Found it very difficult	509 (4.9%)
(Missing)	106 (1.0%)
**Abuse**	
Yes	325 (3.1%)
No	10,009 (96%)
(Missing)	55 (0.5%)
**Outsider growing up**	
Yes	490 (4.7%)
No	9,615 (93%)
(Missing)	284 (2.7%)
**Self-rated health growing up**	
Excellent	2,676 (26%)
Very good	5,371 (52%)
Good	1,779 (17%)
Fair	406 (3.9%)
Poor	123 (1.2%)
(Missing)	34 (0.3%)
**Age 12 religious service attendance**	
At least 1/week	4,751 (46%)
1-3/month	2,689 (26%)
<1/month	2,161 (21%)
Never	354 (3.4%)
(Missing)	434 (4.2%)
**Religious affiliation at age 12**	
Christianity	9,861 (95%)
Islam	3 (<0.1%)

**Characteristic**	**N = 10,389**[1]
Hinduism	0 (0%)
Buddhism	2 (<0.1%)
Judaism	0 (0%)
Sikhism	1 (<0.1%)
Baha'i	0 (0%)
Jainism	0 (0%)
Shinto	0 (0%)
Taoism	0 (0%)
Confucianism	0 (0%)
Primal, Animist, or Folk religion	5 (<0.1%)
Spiritism	0 (0%)
Umbanda, Candomble, and other African-derived religions	0 (0%)
Chinese folk/traditional religion	0 (0%)
Some other religion	0 (0%)
No religion/Atheist/Agnostic	482 (4.6%)
(Missing)	35 (0.3%)

[1]n (%)

Table S16b. Means by demographic category for Poland

		Cantril's Ladder				Life Satisfaction				Happiness			
Variable	Category	Mean	95% CI	SE	Global p-value	Mean	95% CI	SE	Global p-value	Mean	95% CI	SE	Global p-value
Age group	18-24	7.04	(6.83,7.25)	0.11	0.609	7.87	(7.62,8.11)	0.12	< .001	7.83	(7.64,8.02)	0.09	< .001
	25-29	6.99	(6.84,7.13)	0.07		7.73	(7.57,7.88)	0.08		7.73	(7.58,7.87)	0.07	
	30-39	7.11	(6.98,7.24)	0.07		7.60	(7.47,7.74)	0.07		7.63	(7.48,7.77)	0.07	
	40-49	7.12	(6.98,7.25)	0.07		7.45	(7.29,7.61)	0.08		7.53	(7.39,7.67)	0.07	
	50-59	7.10	(6.98,7.22)	0.06		7.41	(7.27,7.54)	0.07		7.42	(7.28,7.56)	0.07	
	60-69	7.24	(7.07,7.41)	0.09		7.52	(7.36,7.68)	0.08		7.54	(7.40,7.67)	0.07	
	70-79	7.13	(6.87,7.39)	0.13		7.32	(7.04,7.59)	0.14		7.35	(7.08,7.61)	0.14	
	80 or older	7.24	(6.62,7.86)	0.31		6.69	(5.64,7.74)	0.53		7.05	(6.22,7.88)	0.42	
Gender	Male	7.14	(7.04,7.24)	0.05	0.551	7.55	(7.43,7.66)	0.06	0.345	7.57	(7.46,7.69)	0.06	0.383
	Female	7.10	(7.00,7.20)	0.05		7.50	(7.39,7.62)	0.06		7.54	(7.44,7.64)	0.05	
	Other	6.18	*	*		6.02	*	*		5.89	*	*	
Marital status	Married	7.26	(7.17,7.35)	0.05	< .001	7.65	(7.54,7.75)	0.05	< .001	7.69	(7.59,7.79)	0.05	< .001
	Separated	6.65	(6.26,7.05)	0.20		7.16	(6.79,7.53)	0.19		7.02	(6.65,7.39)	0.19	
	Divorced	6.65	(6.42,6.87)	0.11		6.94	(6.67,7.20)	0.14		7.09	(6.86,7.33)	0.12	
	Widowed	7.12	(6.89,7.36)	0.12		7.20	(6.94,7.45)	0.13		7.18	(6.96,7.40)	0.11	
	Never	6.87	(6.72,7.03)	0.08		7.51	(7.33,7.70)	0.09		7.50	(7.34,7.66)	0.08	
	Domestic Partner	6.96	(6.81,7.12)	0.08		7.45	(7.27,7.63)	0.09		7.44	(7.26,7.63)	0.09	
Employment	Employed for an employer	7.14	(7.05,7.23)	0.05	< .001	7.59	(7.49,7.69)	0.05	< .001	7.61	(7.51,7.71)	0.05	< .001
	Self-employed	7.26	(7.12,7.39)	0.07		7.61	(7.45,7.77)	0.08		7.68	(7.54,7.82)	0.07	
	Retired	7.20	(7.04,7.37)	0.08		7.35	(7.17,7.52)	0.09		7.43	(7.28,7.58)	0.08	
	Student	7.24	(6.92,7.56)	0.16		8.19	(7.90,8.48)	0.15		8.13	(7.88,8.38)	0.13	
	Homemaker	6.94	(6.59,7.30)	0.18		7.51	(7.14,7.88)	0.19		7.37	(6.92,7.83)	0.23	
	Unemployed and looking for a job	6.26	(5.76,6.75)	0.25		6.61	(6.02,7.21)	0.30		6.88	(6.43,7.34)	0.23	
	None of these/Other	6.31	(5.94,6.69)	0.19		6.99	(6.56,7.41)	0.21		6.73	(6.36,7.10)	0.19	
Religious service attendance	More than 1/week	7.15	(6.62,7.68)	0.27	0.003	7.46	(6.89,8.04)	0.29	< .001	7.71	(7.27,8.14)	0.22	< .001
	1/week	7.30	(7.17,7.42)	0.07		7.77	(7.65,7.89)	0.06		7.76	(7.65,7.88)	0.06	
	1-3/month	7.13	(6.99,7.27)	0.07		7.60	(7.44,7.77)	0.08		7.59	(7.42,7.76)	0.09	
	A few times a year	7.03	(6.92,7.13)	0.05		7.44	(7.31,7.57)	0.07		7.49	(7.38,7.61)	0.06	
	Never	6.93	(6.75,7.11)	0.09		7.09	(6.92,7.27)	0.09		7.17	(6.99,7.34)	0.09	
Education	Up to 8 years	6.83	(6.51,7.15)	0.16	< .001	7.09	(6.69,7.48)	0.20	0.009	7.09	(6.74,7.43)	0.18	< .001
	9-15 years	7.09	(7.00,7.18)	0.05		7.55	(7.46,7.64)	0.05		7.58	(7.49,7.66)	0.05	

Variable	Category	Cantril's Ladder				Life Satisfaction				Happiness			
		Mean	95% CI	SE	Global p-value	Mean	95% CI	SE	Global p-value	Mean	95% CI	SE	Global p-value
	16+ years	7.30	(7.21,7.40)	0.05		7.65	(7.55,7.76)	0.05		7.70	(7.61,7.79)	0.05	
Immigration status	Born in this country	7.13	(7.04,7.21)	0.04	0.031	7.53	(7.43,7.63)	0.05	0.022	7.56	(7.47,7.65)	0.05	0.015
	Born in another country	6.56	(6.04,7.08)	0.26		6.82	(6.21,7.43)	0.31		6.91	(6.39,7.44)	0.26	
Religious affiliation	Christianity	7.14	(7.05,7.23)	0.04	< .001	7.53	(7.43,7.64)	0.05	< .001	7.56	(7.47,7.66)	0.05	< .001
	Islam	5.70	*	*		8.32	*	*		8.00	*	*	
	Buddhism	6.76	*	*		4.96	*	*		6.54	*	*	
	Sikhism	7.00	*	*		5.00	*	*		6.00	*	*	
	Jainism	7.00	*	*		8.00	*	*		7.00	*	*	
	Shinto	6.00	*	*		5.00	*	*		6.00	*	*	
	Primal, Animist, or Folk religion	5.60	(2.33,8.86)	0.42		5.87	(2.64,9.11)	0.47		6.18	(3.75,8.61)	0.39	
	No religion/Atheist /Agnostic	6.98	(6.82,7.13)	0.08		7.46	(7.30,7.63)	0.08		7.48	(7.33,7.62)	0.07	
Race/Ethnicity	Polish	7.13	(7.04,7.21)	0.04	0.012	7.53	(7.43,7.63)	0.05	< .001	7.56	(7.47,7.65)	0.05	< .001
	German	6.54	*	*		7.69	*	*		7.06	*	*	
	Belarussian	6.59	*	*		6.95	*	*		7.42	*	*	
	Ukrainian	5.99	(5.18,6.80)	0.40		6.07	(5.36,6.78)	0.35		6.34	(5.55,7.12)	0.38	
	Silesia	7.82	(6.82,8.81)	0.39		8.92	(8.32,9.52)	0.24		8.44	(7.84,9.05)	0.24	
	Kashubians	7.03	*	*		7.01	*	*		7.74	*	*	
	Other	6.01	(4.36,7.67)	0.81		5.74	(4.14,7.33)	0.78		6.93	(5.82,8.04)	0.51	

Table S16c. Childhood predictors regression analysis results for Poland

Variable	Category	Cantril's Ladder				Life Satisfaction				Happiness			
		Est	95% CI	SE	Global p-value	Est	95% CI	SE	Global p-value	Est	95% CI	SE	Global p-value
Relationship with mother	(Ref: Very bad/somewhat bad)				0.068				0.374				0.216
	Very good/somewhat good	0.32	(-0.03, 0.68)	0.18		0.15	(-0.30, 0.60)	0.22		0.22	(-0.14, 0.58)	0.19	
Relationship with father	(Ref: Very bad/somewhat bad)				0.018				0.009				0.004
	Very good/somewhat good	0.29	(0.04, 0.54)	0.13		0.45	(0.10, 0.80)	0.18		0.37	(0.11, 0.63)	0.13	
Parent marital status	(Ref: Parents married)				0.046				<.001				<.001
	Divorced	-0.15	(-0.38, 0.07)	0.11		-0.44	(-0.66, -0.23)	0.11		-0.28	(-0.49, -0.07)	0.11	
	Parents were never married	-0.42	(-0.85, 0.00)	0.21		-0.71	(-1.22, -0.19)	0.25		-0.43	(-0.84, -0.02)	0.20	
	One or both parents had died	-0.02	(-0.38, 0.34)	0.18		-0.06	(-0.44, 0.32)	0.19		-0.25	(-0.63, 0.12)	0.19	
Subjective financial status of family growing up	(Ref: Got by)				0.727				0.553				0.283
	Lived comfortably	0.01	(-0.14, 0.16)	0.08		-0.00	(-0.15, 0.14)	0.07		0.07	(-0.06, 0.20)	0.07	
	Found it difficult	-0.07	(-0.21, 0.06)	0.07		-0.08	(-0.22, 0.06)	0.07		-0.09	(-0.22, 0.04)	0.07	
	Found it very difficult	-0.08	(-0.46, 0.30)	0.19		0.08	(-0.31, 0.47)	0.20		-0.14	(-0.50, 0.22)	0.18	
Abuse	(Ref: No)				0.130				0.005				<.001
	Yes	-0.25	(-0.58, 0.08)	0.17		-0.49	(-0.84, -0.14)	0.18		-0.69	(-1.04, -0.33)	0.18	
Outsider growing up	(Ref: No)				0.374				0.054				0.190
	Yes	-0.11	(-0.38, 0.15)	0.13		-0.26	(-0.62, 0.09)	0.18		-0.16	(-0.42, 0.10)	0.13	
Self-rated health growing up	(Ref: Good)				<.001				<.001				<.001
	Excellent	0.53	(0.35, 0.71)	0.09		0.70	(0.51, 0.89)	0.10		0.65	(0.47, 0.84)	0.09	

Variable	Category	Cantril's Ladder				Life Satisfaction				Happiness			
		Est	95% CI	SE	Global p-value	Est	95% CI	SE	Global p-value	Est	95% CI	SE	Global p-value
	Very good	0.27	(0.11, 0.43)	0.08		0.27	(0.11, 0.44)	0.08		0.30	(0.15, 0.46)	0.08	
	Fair	-0.34	(-0.65, -0.03)	0.16		-0.29	(-0.68, 0.10)	0.20		-0.38	(-0.79, 0.03)	0.21	
	Poor	0.41	(-0.31, 1.12)	0.36		-0.04	(-0.85, 0.77)	0.41		0.43	(-0.23, 1.08)	0.33	
Immigration status	(Ref: Born in this country)				0.472				0.598				0.587
	Born in another country	-0.19	(-0.70, 0.33)	0.26		-0.18	(-0.87, 0.51)	0.35		-0.16	(-0.72, 0.41)	0.29	
Age 12 religious service attendance	(Ref: Never)				<.001				<.001				<.001
	At least 1/week	0.65	(0.34, 0.96)	0.16		1.03	(0.64, 1.42)	0.20		1.00	(0.68, 1.32)	0.16	
	1-3/month	0.49	(0.18, 0.80)	0.16		0.73	(0.34, 1.11)	0.20		0.74	(0.39, 1.08)	0.17	
	< 1/month	0.44	(0.12, 0.77)	0.16		0.45	(0.05, 0.84)	0.20		0.48	(0.15, 0.81)	0.17	
Year of birth	(Ref: 1998-2005; current age: 18-24)				0.605				<.001				<.001
	1993-1998; age 25-29	-0.04	(-0.25, 0.17)	0.11		-0.14	(-0.38, 0.09)	0.12		-0.10	(-0.28, 0.08)	0.09	
	1983-1993; age 30-39	0.09	(-0.13, 0.31)	0.11		-0.27	(-0.49, -0.06)	0.11		-0.19	(-0.35, -0.02)	0.09	
	1973-1983; age 40-49	0.10	(-0.12, 0.32)	0.11		-0.43	(-0.69, -0.18)	0.13		-0.28	(-0.46, -0.10)	0.09	
	1963-1973; age 50-59	0.10	(-0.12, 0.32)	0.11		-0.47	(-0.72, -0.23)	0.13		-0.39	(-0.58, -0.20)	0.10	
	1953-1963; age 60-69	0.19	(-0.08, 0.47)	0.14		-0.47	(-0.75, -0.20)	0.14		-0.35	(-0.55, -0.14)	0.11	
	1943-1953; age 70-79	0.11	(-0.21, 0.44)	0.17		-0.63	(-1.00, -0.26)	0.19		-0.50	(-0.80, -0.19)	0.16	
	1943 or earlier; age 80+	0.25	(-0.36, 0.87)	0.31		-1.20	(-2.11, -0.30)	0.46		-0.75	(-1.64, 0.14)	0.45	
Gender	(Ref: Male)				0.356				0.173				0.204
	Female	-0.07	(-0.17, 0.03)	0.05		-0.07	(-0.17, 0.03)	0.05		-0.06	(-0.16, 0.04)	0.05	
	Other	-0.76	(-2.98, 1.46)	1.13		-1.51	(-4.01, 0.99)	1.28		-1.54	(-3.90, 0.82)	1.20	
Religious affiliation	(Ref: No religion/Atheist /Agnostic)				0.133				0.006				0.004
	Christianity	-0.08	(-0.35, 0.19)	0.14		-0.34	(-0.67, -0.01)	0.17		-0.39	(-0.69, -0.10)	0.15	

Variable	Category	Cantril's Ladder				Life Satisfaction				Happiness			
		Est	95% CI	SE	Global p-value	Est	95% CI	SE	Global p-value	Est	95% CI	SE	Global p-value
Race/ethnicity	Collapsed affiliations with prevalence<3% (Ref: Plurality group)	-1.05	(-2.08, -0.02)	0.53	0.299	-1.55	(-2.86, -0.24)	0.65	0.451	-1.18	(-2.06, -0.29)	0.45	0.391
	Non-plurality groups	-0.36	(-1.05, 0.32)	0.35		-0.34	(-1.21, 0.54)	0.45		-0.31	(-1.02, 0.40)	0.36	

Table S16d. Sensitivity to unmeasured confounding of childhood predictors in Poland

Variable	Category	Cantril's Ladder		Life Satisfaction		Happiness	
		E-value for Estimate	E-value for 95% CI	E-value for Estimate	E-value for 95% CI	E-value for Estimate	E-value for 95% CI
Relationship with mother	(Ref: Very bad/somewhat bad)						
	Very good/somewhat good	1.69	1.00	1.38	1.00	1.52	1.00
Relationship with father	(Ref: Very bad/somewhat bad)						
	Very good/somewhat good	1.63	1.17	1.85	1.30	1.77	1.33
Parent marital status	(Ref: Parents married)						
	Divorced	1.40	1.00	1.84	1.51	1.62	1.24
	Parents were never married	1.86	1.06	2.26	1.49	1.88	1.17
	One or both parents had died	1.12	1.00	1.21	1.00	1.58	1.00
Subjective financial status of family growing up	(Ref: Got by)						
	Lived comfortably	1.08	1.00	1.02	1.00	1.24	1.00
	Found it difficult	1.25	1.00	1.26	1.00	1.29	1.00
	Found it very difficult	1.27	1.00	1.25	1.00	1.39	1.00
Abuse	(Ref: No)						
	Yes	1.57	1.00	1.91	1.37	2.32	1.71
Outsider growing up	(Ref: No)						
	Yes	1.33	1.00	1.56	1.00	1.42	1.00
Self-rated health growing up	(Ref: Good)						
	Excellent	2.03	1.74	2.25	1.95	2.26	1.94
	Very good	1.61	1.33	1.57	1.30	1.66	1.39
	Fair	1.72	1.14	1.60	1.00	1.79	1.00
	Poor	1.82	1.00	1.17	1.00	1.87	1.00
Immigration status	(Ref: Born in this country)						
	Born in another country	1.46	1.00	1.43	1.00	1.41	1.00
Age 12 religious service attendance	(Ref: Never)						
	At least 1/week	2.24	1.73	2.83	2.16	2.93	2.31
	1-3/month	1.96	1.45	2.29	1.69	2.41	1.81
	< 1/month	1.89	1.35	1.84	1.20	1.96	1.40
Year of birth	(Ref: 1998-2005; current age: 18-24)						
	1993-1998; age 25-29	1.18	1.00	1.36	1.00	1.31	1.00
	1983-1993; age 30-39	1.29	1.00	1.57	1.21	1.47	1.12
	1973-1983; age 40-49	1.31	1.00	1.82	1.42	1.62	1.30
	1963-1973; age 50-59	1.31	1.00	1.88	1.51	1.80	1.48

Variable	Category	Cantril's Ladder		Life Satisfaction		Happiness	
		E-value for Estimate	E-value for 95% CI	E-value for Estimate	E-value for 95% CI	E-value for Estimate	E-value for 95% CI
	1953-1963; age 60-69	1.47	1.00	1.89	1.47	1.73	1.38
	1943-1953; age 70-79	1.33	1.00	2.13	1.56	1.99	1.48
	1943 or earlier; age 80+	1.57	1.00	3.17	1.62	2.44	1.00
Gender	(Ref: Male)						
	Female	1.24	1.00	1.24	1.00	1.23	1.00
	Other	2.44	1.00	3.85	1.00	4.25	1.00
Religious affiliation	(Ref: No religion/Atheist/Agnostic)						
	Christianity	1.27	1.00	1.68	1.09	1.81	1.31
	Collapsed affiliations with prevalence<3%	3.01	1.12	3.95	1.57	3.32	1.64
Race/ethnicity	(Ref: Plurality group)						
	Non-plurality groups	1.76	1.00	1.67	1.00	1.67	1.00

Table S16e. Complete-case supplemental analysis of means by demographic category for Poland

		Cantril's Ladder				Life Satisfaction				Happiness			
Variable	Category	Mean	95% CI	SE	Global p-value	Mean	95% CI	SE	Global p-value	Mean	95% CI	SE	Global p-value
Age group	18-24	7.04	(6.83,7.25)	0.11	0.539	7.94	(7.72,8.15)	0.11	< .001	7.84	(7.66,8.03)	0.09	< .001
	25-29	7.00	(6.86,7.14)	0.07		7.75	(7.59,7.90)	0.08		7.74	(7.59,7.88)	0.07	
	30-39	7.11	(6.98,7.24)	0.07		7.61	(7.48,7.74)	0.07		7.65	(7.52,7.79)	0.07	
	40-49	7.15	(7.03,7.27)	0.06		7.45	(7.29,7.61)	0.08		7.53	(7.39,7.67)	0.07	
	50-59	7.10	(6.98,7.22)	0.06		7.41	(7.28,7.55)	0.07		7.42	(7.28,7.56)	0.07	
	60-69	7.25	(7.08,7.42)	0.09		7.53	(7.37,7.69)	0.08		7.55	(7.41,7.68)	0.07	
	70-79	7.19	(6.94,7.44)	0.13		7.41	(7.17,7.64)	0.12		7.43	(7.18,7.69)	0.13	
	80 or older	7.23	(6.61,7.86)	0.32		7.06	(6.26,7.86)	0.40		7.05	(6.22,7.88)	0.42	
Gender	Female	7.13	(7.03,7.22)	0.05	0.678	7.54	(7.43,7.65)	0.05	0.397	7.56	(7.46,7.65)	0.05	0.390
	Male	7.15	(7.05,7.24)	0.05		7.57	(7.45,7.68)	0.06		7.58	(7.48,7.69)	0.06	
	Other	6.18	(0.00,1082410<br>88225460286<br>46559401305<br>11393394126<br>86674124591<br>52359896640<br>11994412573<br>81483459379<br>2.00)	1.15		6.02	(0.00,1167096<br>16651078450<br>73358784541<br>74786547080<br>48981945064<br>71655915038<br>15098240901<br>78642037964<br>8.00)	1.24		5.89	(0.00,1235787<br>90580129243<br>16227493679<br>19319721026<br>47563458565<br>49036319338<br>97129474372<br>85100067225<br>6.00)	1.31	
Marital status	Divorced	6.72	(6.49,6.95)	0.12	< .001	7.01	(6.75,7.27)	0.13	< .001	7.15	(6.92,7.37)	0.12	< .001
	Domestic partner	6.99	(6.84,7.15)	0.08		7.44	(7.26,7.62)	0.09		7.47	(7.28,7.65)	0.09	
	Married	7.27	(7.18,7.36)	0.05		7.64	(7.54,7.75)	0.05		7.69	(7.59,7.79)	0.05	
	Separated	6.68	(6.31,7.05)	0.19		7.18	(6.81,7.56)	0.19		7.05	(6.69,7.42)	0.19	
	Single/Never been married	6.89	(6.73,7.05)	0.08		7.58	(7.41,7.76)	0.09		7.55	(7.40,7.70)	0.07	
	Widowed	7.10	(6.88,7.33)	0.11		7.31	(7.10,7.51)	0.10		7.21	(7.00,7.42)	0.11	
Employment	Employed for an employer	7.14	(7.06,7.23)	0.05	< .001	7.60	(7.49,7.70)	0.05	< .001	7.62	(7.51,7.72)	0.05	< .001
	Homemaker	6.92	(6.56,7.28)	0.18		7.50	(7.13,7.86)	0.19		7.54	(7.19,7.89)	0.18	
	None of these/Other	6.31	(5.95,6.67)	0.18		7.02	(6.60,7.43)	0.21		6.73	(6.37,7.09)	0.18	
	Retired	7.22	(7.05,7.38)	0.08		7.41	(7.25,7.57)	0.08		7.45	(7.31,7.60)	0.07	
	Self-employed	7.27	(7.14,7.40)	0.07		7.62	(7.47,7.77)	0.08		7.68	(7.54,7.83)	0.07	
	Student	7.24	(6.93,7.56)	0.16		8.19	(7.90,8.48)	0.15		8.13	(7.89,8.38)	0.13	
	Unemployed and looking for a job	6.49	(6.13,6.84)	0.18		6.84	(6.33,7.34)	0.26		6.90	(6.44,7.35)	0.23	

Variable	Category	Cantril's Ladder				Life Satisfaction				Happiness			
		Mean	95% CI	SE	Global p-value	Mean	95% CI	SE	Global p-value	Mean	95% CI	SE	Global p-value
Religious service attendance	A few times a year	7.03	(6.93,7.13)	0.05	0.005	7.46	(7.33,7.59)	0.07	< .001	7.49	(7.38,7.60)	0.06	< .001
	More than once a week	7.17	(6.63,7.71)	0.27		7.68	(7.26,8.09)	0.21		7.92	(7.56,8.27)	0.18	
	Never	7.00	(6.84,7.16)	0.08		7.13	(6.96,7.30)	0.09		7.23	(7.08,7.38)	0.08	
	Once a week	7.31	(7.18,7.44)	0.06		7.78	(7.66,7.89)	0.06		7.77	(7.65,7.89)	0.06	
	One to three times a month	7.13	(6.98,7.27)	0.07		7.64	(7.49,7.79)	0.08		7.59	(7.42,7.76)	0.09	
Education	Up to 8 years	6.88	(6.57,7.19)	0.16	< .001	7.22	(6.87,7.57)	0.18	0.022	7.18	(6.85,7.51)	0.17	0.002
	16+ years	7.31	(7.22,7.40)	0.05		7.66	(7.56,7.77)	0.05		7.71	(7.61,7.80)	0.05	
	9 to 15 years	7.10	(7.01,7.19)	0.05		7.56	(7.47,7.65)	0.05		7.58	(7.49,7.67)	0.05	
Immigration status	Born in another country	6.56	(6.04,7.08)	0.26	0.027	6.82	(6.21,7.43)	0.31	0.018	6.91	(6.39,7.44)	0.26	0.013
	Born in this country	7.14	(7.06,7.22)	0.04		7.56	(7.46,7.65)	0.05		7.58	(7.49,7.67)	0.05	
Religious affiliation	Buddhism	6.76	*	*	< .001	4.96	*	*	< .001	6.54	*	*	< .001
	Christianity	7.15	(7.07,7.24)	0.04		7.56	(7.46,7.66)	0.05		7.58	(7.49,7.68)	0.05	
	Islam	5.68	*	*		8.32	*	*		8.00	*	*	
	No religion/Atheist/Agnostic	6.97	(6.81,7.13)	0.08		7.48	(7.32,7.64)	0.08		7.45	(7.30,7.61)	0.08	
	Primal, Animist, or Folk religion	5.67	(3.57,7.77)	0.33		5.78	(2.53,9.03)	0.52		6.18	(3.75,8.61)	0.39	
	Sikhism	7.00	*	*		5.00	*	*		6.00	*	*	
	Jainism	7.00	*	*		8.00	*	*		7.00	*	*	
	Shinto	6.00	*	*		5.00	*	*		6.00	*	*	

Table S16f. Complete-case supplemental analysis of childhood predictors regression analysis results for Poland

		Cantril's Ladder				Life Satisfaction				Happiness			
Variable	Category	Est	95% CI	SE	Global p-value	Est	95% CI	SE	Global p-value	Est	95% CI	SE	Global p-value
Relationship with mother	(Ref: Very bad/somewhat bad)				0.078				0.342				0.125
	Very good/somewhat good	0.34	(-0.04,0.71)	0.19		0.18	(-0.19,0.56)	0.19		0.29	(-0.08,0.65)	0.19	
Relationship with father	(Ref: Very bad/somewhat bad)				0.032				0.010				0.007
	Very good/somewhat good	0.27	(0.02,0.52)	0.13		0.44	(0.11,0.77)	0.17		0.35	(0.09,0.60)	0.13	
Parent marital status	(Ref: Parents married)				0.392				<.001				<.001
	No, one or both of them had died	0.15	(-0.21,0.51)	0.18		0.40	(0.05,0.76)	0.18		0.01	(-0.36,0.38)	0.19	
	No, they were never married	-0.08	(-0.55,0.38)	0.24		-0.22	(-0.61,0.16)	0.20		-0.15	(-0.46,0.16)	0.16	
	Yes, married	0.15	(-0.06,0.35)	0.10		0.47	(0.26,0.67)	0.10		0.28	(0.09,0.46)	0.09	
Subjective financial status of family growing up	(Ref: Got by)				0.778				0.862				0.383
	Found it difficult	-0.07	(-0.20,0.06)	0.07		-0.05	(-0.18,0.07)	0.06		-0.08	(-0.20,0.04)	0.06	
	Found it very difficult	0.01	(-0.28,0.30)	0.15		-0.01	(-0.40,0.37)	0.20		-0.12	(-0.45,0.22)	0.17	
	Lived comfortably	0.00	(-0.14,0.15)	0.07		-0.01	(-0.14,0.12)	0.07		0.06	(-0.07,0.19)	0.07	
Abuse	(Ref: No)				0.135				0.009				<.001
	Yes	-0.25	(-0.59,0.08)	0.17		-0.45	(-0.78,-0.11)	0.17		-0.55	(-0.87,-0.24)	0.16	
Outsider growing up	(Ref: No)				0.151				0.028				0.087
	Yes	-0.19	(-0.44,0.07)	0.13		-0.33	(-0.63,-0.04)	0.15		-0.22	(-0.47,0.03)	0.13	

		Cantril's Ladder				Life Satisfaction				Happiness			
Variable	Category	Est	95% CI	SE	Global p-value	Est	95% CI	SE	Global p-value	Est	95% CI	SE	Global p-value
Self-rated health growing up	(Ref: Good)				<.001				<.001				<.001
	Excellent	0.53	(0.36,0.69)	0.08		0.67	(0.50,0.85)	0.09		0.66	(0.48,0.84)	0.09	
	Fair	-0.41	(-0.70,-0.11)	0.15		-0.21	(-0.51,0.10)	0.16		-0.30	(-0.62,0.03)	0.17	
	Poor	0.32	(-0.40,1.04)	0.37		-0.14	(-0.98,0.71)	0.43		0.38	(-0.23,0.99)	0.31	
	Very good	0.27	(0.13,0.41)	0.07		0.24	(0.09,0.39)	0.07		0.32	(0.17,0.46)	0.08	
Immigration status	(Ref: Born in this country)				0.404				0.562				0.475
	Born in another country	-0.21	(-0.71,0.29)	0.25		-0.20	(-0.87,0.47)	0.34		-0.21	(-0.77,0.36)	0.29	
Age 12 religious service attendance	(Ref: Never)				0.003				<.001				<.001
	At least once a week	0.49	(0.19,0.78)	0.15		1.07	(0.67,1.47)	0.20		0.76	(0.50,1.02)	0.13	
	Less than once a month	0.29	(-0.02,0.60)	0.16		0.50	(0.11,0.90)	0.20		0.26	(-0.00,0.52)	0.13	
	One to three times a month	0.33	(0.03,0.63)	0.15		0.76	(0.36,1.15)	0.20		0.48	(0.21,0.75)	0.14	
Year of birth	(Ref: 1998-2005; current age: 18-24)				0.591				<.001				<.001
	1993-1998; age 25-29	-0.03	(-0.24,0.19)	0.11		-0.19	(-0.40,0.02)	0.11		-0.10	(-0.28,0.08)	0.09	
	1983-1993; age 30-39	0.09	(-0.13,0.31)	0.11		-0.33	(-0.51,-0.14)	0.10		-0.18	(-0.34,-0.02)	0.08	
	1973-1983; age 40-49	0.14	(-0.08,0.35)	0.11		-0.49	(-0.71,-0.27)	0.11		-0.29	(-0.47,-0.11)	0.09	
	1963-1973; age 50-59	0.10	(-0.13,0.32)	0.11		-0.54	(-0.74,-0.34)	0.10		-0.41	(-0.59,-0.22)	0.10	
	1953-1963; age 60-69	0.20	(-0.08,0.47)	0.14		-0.53	(-0.77,-0.28)	0.12		-0.35	(-0.56,-0.15)	0.10	
	1943-1953; age 70-79	0.17	(-0.15,0.48)	0.16		-0.61	(-0.90,-0.31)	0.15		-0.42	(-0.71,-0.13)	0.15	

		Cantril's Ladder				Life Satisfaction				Happiness			
Variable	Category	Est	95% CI	SE	Global p-value	Est	95% CI	SE	Global p-value	Est	95% CI	SE	Global p-value
	1943 or earlier; age 80+	0.25	(-0.37,0.86)	0.31		-0.87	(-1.57,-0.17)	0.36		-0.78	(-1.67,0.11)	0.45	
Gender	(Ref: Male)				0.591				0.197				0.249
	Male	0.04	(-0.06,0.14)	0.05		0.06	(-0.04,0.16)	0.05		0.05	(-0.04,0.14)	0.05	
	Other	-0.72	(-2.87,1.44)	1.10		-1.53	(-3.97,0.90)	1.24		-1.58	(-4.12,0.95)	1.29	
Religious affiliation	(Ref: No religion/Atheist/Agnostic)				0.154				0.021				0.115
	Christianity	0.06	(-0.16,0.29)	0.12		-0.20	(-0.47,0.07)	0.14		-0.10	(-0.31,0.10)	0.10	
	Christianity	0.06	(-0.16,0.29)	0.12		-0.20	(-0.47,0.07)	0.14		-0.10	(-0.31,0.10)	0.10	
	Collapsed affiliations with prevalence<3 %	-0.90	(-1.96,0.15)	0.54		-1.47	(-2.57,-0.37)	0.56		-0.90	(-1.76,-0.04)	0.44	
Race/ethnicity	(Ref: Plurality group)				0.315				0.402				0.437
	Non-plurality groups	-0.35	(-1.02,0.33)	0.34		-0.37	(-1.23,0.49)	0.44		-0.27	(-0.95,0.41)	0.35	

Table S16g. Complete-case supplemental analysis of sensitivity to unmeasured confounding of childhood predictors in Poland

		Cantril's Ladder		Life Satisfaction		Happiness	
Variable	Category	E-value for Estimate	E-value for 95% CI	E-value for Estimate	E-value for 95% CI	E-value for Estimate	E-value for 95% CI
Relationship with mother	(Ref: Very bad/somewhat bad)						
	Very good/somewhat good	1.72	1.00	1.44	1.00	1.65	1.00
Relationship with father	(Ref: Very bad/somewhat bad)						
	Very good/somewhat good	1.61	1.13	1.86	1.31	1.75	1.30
Parent marital status	(Ref: Parents married)						
	No, one or both of them had died	1.41	1.00	1.80	1.19	1.09	1.00
	No, they were never married	1.28	1.00	1.51	1.00	1.41	1.00
	Yes, married	1.40	1.00	1.90	1.57	1.63	1.30
Subjective financial status of family growing up	(Ref: Got by)						
	Found it difficult	1.24	1.00	1.20	1.00	1.27	1.00
	Found it very difficult	1.07	1.00	1.09	1.00	1.35	1.00
	Lived comfortably	1.04	1.00	1.07	1.00	1.23	1.00
Abuse	(Ref: No)						
	Yes	1.58	1.00	1.87	1.32	2.11	1.57
Outsider growing up	(Ref: No)						
	Yes	1.47	1.00	1.69	1.16	1.53	1.00
Self-rated health growing up	(Ref: Good)						
	Excellent	2.05	1.77	2.24	1.95	2.31	1.98
	Fair	1.84	1.34	1.48	1.00	1.67	1.00
	Poor	1.69	1.00	1.36	1.00	1.80	1.00
	Very good	1.61	1.36	1.54	1.29	1.70	1.44
Immigration status	(Ref: Born in this country)						
	Born in another country	1.51	1.00	1.47	1.00	1.51	1.00
Age 12 religious service attendance	(Ref: Never)						
	At least once a week	1.97	1.47	2.98	2.24	2.49	2.02
	Less than once a month	1.64	1.00	1.96	1.31	1.60	1.00
	One to three times a month	1.71	1.16	2.39	1.74	1.98	1.52
Year of birth	(Ref: 1998-2005; current age: 18-24)						

Variable	Category	Cantril's Ladder		Life Satisfaction		Happiness	
		E-value for Estimate	E-value for 95% CI	E-value for Estimate	E-value for 95% CI	E-value for Estimate	E-value for 95% CI
	1993-1998; age 25-29	1.14	1.00	1.46	1.00	1.31	1.00
	1983-1993; age 30-39	1.28	1.00	1.68	1.37	1.46	1.12
	1973-1983; age 40-49	1.38	1.00	1.94	1.59	1.65	1.33
	1963-1973; age 50-59	1.30	1.00	2.02	1.70	1.85	1.53
	1953-1963; age 60-69	1.48	1.00	2.00	1.61	1.76	1.41
	1943-1953; age 70-79	1.43	1.00	2.13	1.65	1.88	1.38
	1943 or earlier; age 80+	1.57	1.00	2.59	1.43	2.53	1.00
Gender	(Ref: Male)						
	Male	1.18	1.00	1.22	1.00	1.21	1.00
	Other	2.39	1.00	4.03	1.00	4.49	1.00
Religious affiliation	(Ref: No religion/Atheist/Agnostic)						
	Christianity	1.23	1.00	1.47	1.00	1.32	1.00
	Christianity	1.23	1.00	1.47	1.00	1.32	1.00
	Collapsed affiliations with prevalence<3%	2.75	1.00	3.87	1.74	2.77	1.19
Race/ethnicity	(Ref: Plurality group)						
	Non-plurality groups	1.74	1.00	1.74	1.00	1.62	1.00

Tables S17a-g: South Africa

Table S17a. Nationally representative descriptive statistics for South Africa

Characteristic	N = 2,651[1]
**Age group**	
1998-2005; age 18-24	461 (17%)
1993-1998; age 25-29	364 (14%)
1983-1993; age 30-39	655 (25%)
1973-1983; age 40-49	522 (20%)
1963-1973; age 50-59	309 (12%)
1953-1963; age 60-69	195 (7.4%)
1943-1953; age 70-79	120 (4.5%)
1943 or earlier; age 80+	17 (0.6%)
(Missing)	9 (0.3%)
**Gender**	
Male	1,288 (49%)
Female	1,356 (51%)
Other	2 (<0.1%)
(Missing)	4 (0.2%)
**Race/Ethnicity**	
Asian/Indian	6 (0.2%)
Black	2,381 (90%)
Colored	252 (9.5%)
Other	1 (<0.1%)
White	8 (0.3%)
(Missing)	3 (0.1%)
**Respondent Marital status**	
Married	539 (20%)
Separated	76 (2.9%)
Divorced	51 (1.9%)
Widowed	133 (5.0%)
Single, never married	1,561 (59%)
Domestic Partner	264 (10.0%)
(Missing)	28 (1.0%)
**Employment**	
Employed for an employer	569 (21%)
Self-employed	412 (16%)
Retired	243 (9.2%)
Student	204 (7.7%)
Homemaker	137 (5.2%)
Unemployed and looking for a job	1,008 (38%)
None of these/Other	74 (2.8%)
(Missing)	3 (0.1%)
**Religious service attendance as an adult (now)**	
More than 1/week	414 (16%)
1/week	891 (34%)
1-3/month	574 (22%)
A few times a year	431 (16%)
Never	334 (13%)
(Missing)	7 (0.3%)
**Education (years)**	
Up to 8 years	668 (25%)
9-15 years	1,796 (68%)
16+ years	183 (6.9%)
(Missing)	4 (0.2%)
**Immigration status**	
Born in this country	2,511 (95%)
Born in another country	139 (5.2%)
(Missing)	1 (<0.1%)
**Religious affiliation as an adult (now)**	
Christianity	2,163 (82%)
Islam	62 (2.3%)
Hinduism	1 (<0.1%)
Buddhism	12 (0.5%)
Judaism	0 (0%)

Characteristic	N = 2,651[1]
Sikhism	0 (0%)
Baha'i	0 (0%)
Jainism	2 (<0.1%)
Shinto	2 (<0.1%)
Taoism	1 (<0.1%)
Confucianism	0 (0%)
Primal, Animist, or Folk religion	127 (4.8%)
Spiritism	0 (0%)
Umbanda, Candomble, and other African-derived religions	0 (0%)
Chinese folk/traditional religion	0 (0%)
Some other religion	5 (0.2%)
No religion/Atheist/Agnostic	253 (9.6%)
(Missing)	23 (0.9%)
**Relationship with mother growing up**	
Very good	2,186 (82%)
Somewhat good	263 (9.9%)
Somewhat bad	51 (1.9%)
Very bad	39 (1.5%)
Does not apply	90 (3.4%)
(Missing)	21 (0.8%)
**Relationship with father growing up**	
Very good	1,656 (62%)
Somewhat good	333 (13%)
Somewhat bad	86 (3.3%)
Very bad	159 (6.0%)
Does not apply	331 (12%)
(Missing)	85 (3.2%)
**Parent marital status at age 12**	
Parents married	1,321 (50%)
Divorced	131 (5.0%)
Parents were never married	904 (34%)
One or both parents had died	140 (5.3%)
(Missing)	155 (5.8%)
**Subjective financial status of family growing up**	
Lived comfortably	1,050 (40%)
Got by	875 (33%)
Found it difficult	432 (16%)
Found it very difficult	289 (11%)
(Missing)	5 (0.2%)
**Abuse**	
Yes	450 (17%)
No	2,149 (81%)
(Missing)	52 (2.0%)
**Outsider growing up**	
Yes	434 (16%)
No	2,211 (83%)
(Missing)	6 (0.2%)
**Self-rated health growing up**	
Excellent	1,225 (46%)
Very good	590 (22%)
Good	370 (14%)
Fair	266 (10%)
Poor	183 (6.9%)
(Missing)	17 (0.6%)
**Age 12 religious service attendance**	
At least 1/week	1,681 (63%)
1-3/month	552 (21%)
<1/month	175 (6.6%)
Never	217 (8.2%)
(Missing)	26 (1.0%)
**Religious affiliation at age 12**	
Christianity	2,323 (88%)
Islam	52 (2.0%)
Hinduism	2 (<0.1%)
Buddhism	11 (0.4%)

Characteristic	N = 2,651[1]
Judaism	0 (0%)
Sikhism	0 (0%)
Baha'i	0 (0%)
Jainism	0 (0%)
Shinto	2 (<0.1%)
Taoism	1 (<0.1%)
Confucianism	0 (0%)
Primal, Animist, or Folk religion	117 (4.4%)
Spiritism	0 (0%)
Umbanda, Candomble, and other African-derived religions	0 (0%)
Chinese folk/traditional religion	0 (0%)
Some other religion	7 (0.3%)
No religion/Atheist/Agnostic	107 (4.1%)
(Missing)	27 (1.0%)

[1]n (%)

Table S17b. Means by demographic category for South Africa

		Cantril's Ladder				Life Satisfaction				Happiness			
Variable	Category	Mean	95% CI	SE	Global p-value	Mean	95% CI	SE	Global p-value	Mean	95% CI	SE	Global p-value
Age group	18-24	6.34	(6.04,6.64)	0.15	0.641	6.47	(6.17,6.77)	0.15	0.013	7.20	(6.89,7.52)	0.16	0.121
	25-29	6.14	(5.85,6.43)	0.15		6.18	(5.82,6.54)	0.18		6.77	(6.49,7.04)	0.14	
	30-39	6.24	(5.97,6.51)	0.14		6.44	(6.18,6.71)	0.14		7.07	(6.83,7.30)	0.12	
	40-49	5.99	(5.66,6.32)	0.17		6.14	(5.84,6.45)	0.16		6.59	(6.30,6.89)	0.15	
	50-59	5.85	(5.27,6.44)	0.30		6.46	(5.92,7.00)	0.27		7.04	(6.57,7.51)	0.24	
	60-69	5.75	(4.96,6.54)	0.40		6.27	(5.50,7.04)	0.39		6.97	(6.16,7.78)	0.41	
	70-79	6.11	(5.02,7.19)	0.55		6.74	(5.72,7.75)	0.51		7.16	(6.10,8.23)	0.54	
	80 or older	5.90	(3.39,8.40)	1.04		7.45	(6.68,8.23)	0.32		7.92	(4.88,10.0ǂ)	1.27	
Gender	Male	6.01	(5.77,6.25)	0.12	0.309	6.37	(6.15,6.60)	0.12	0.957	6.96	(6.75,7.16)	0.10	0.471
	Female	6.19	(5.98,6.41)	0.11		6.35	(6.16,6.54)	0.10		6.95	(6.74,7.16)	0.11	
	Other	6.62	*	*		6.29	*	*		6.19	*	*	
Marital status	Married	6.37	(5.98,6.76)	0.20	0.403	6.70	(6.35,7.06)	0.18	0.031	7.18	(6.81,7.56)	0.19	0.297
	Separated	6.37	(5.48,7.25)	0.44		6.80	(5.85,7.75)	0.48		7.29	(6.56,8.02)	0.37	
	Divorced	5.20	(3.96,6.43)	0.61		5.29	(4.11,6.47)	0.59		5.99	(4.72,7.26)	0.63	
	Widowed	6.28	(5.40,7.17)	0.45		6.85	(5.90,7.81)	0.48		7.09	(6.13,8.05)	0.49	
	Never	6.07	(5.88,6.25)	0.10		6.24	(6.06,6.42)	0.09		6.90	(6.74,7.07)	0.08	
	Domestic Partner	5.80	(5.25,6.35)	0.28		6.23	(5.77,6.69)	0.23		6.81	(6.34,7.28)	0.24	
Employment	Employed for an employer	6.53	(6.25,6.81)	0.14	0.019	6.73	(6.45,7.01)	0.14	0.005	7.22	(6.99,7.44)	0.12	0.057
	Self-employed	6.13	(5.78,6.48)	0.18		6.45	(6.11,6.80)	0.18		6.85	(6.46,7.23)	0.20	
	Retired	5.94	(5.24,6.64)	0.35		6.57	(5.89,7.24)	0.34		7.16	(6.44,7.88)	0.37	
	Student	6.37	(5.96,6.78)	0.21		6.54	(6.09,6.99)	0.23		7.16	(6.76,7.55)	0.20	
	Homemaker	5.72	(4.96,6.49)	0.39		6.17	(5.45,6.90)	0.37		6.90	(6.27,7.53)	0.32	
	Unemployed and looking for a job	5.89	(5.63,6.15)	0.13		6.04	(5.82,6.26)	0.11		6.74	(6.52,6.95)	0.11	
	None of these/Other	6.19	(4.83,7.55)	0.68		6.60	(5.43,7.77)	0.58		7.34	(6.04,8.65)	0.65	
Religious service attendance	More than 1/week	5.98	(5.56,6.40)	0.21	0.049	6.60	(6.19,7.00)	0.20	0.016	7.34	(6.98,7.70)	0.18	0.049
	1/week	6.21	(5.92,6.50)	0.15		6.61	(6.36,6.86)	0.13		7.01	(6.76,7.26)	0.13	
	1-3/month	5.88	(5.55,6.21)	0.17		6.34	(6.03,6.65)	0.16		6.89	(6.55,7.23)	0.17	
	A few times a year	6.42	(6.16,6.69)	0.13		6.03	(5.68,6.37)	0.17		6.62	(6.29,6.94)	0.17	
	Never	5.96	(5.53,6.38)	0.22		5.89	(5.42,6.35)	0.24		6.88	(6.51,7.24)	0.18	
Education	Up to 8 years	5.87	(5.37,6.38)	0.26	0.241	6.55	(6.14,6.96)	0.21	0.379	6.87	(6.45,7.28)	0.21	0.826
	9-15 years	6.16	(6.01,6.31)	0.08		6.28	(6.13,6.43)	0.08		6.98	(6.83,7.12)	0.07	

Variable	Category	Cantril's Ladder				Life Satisfaction				Happiness			
		Mean	95% CI	SE	Global p-value	Mean	95% CI	SE	Global p-value	Mean	95% CI	SE	Global p-value
	16+ years	6.43	(6.01,6.84)	0.21		6.46	(6.04,6.89)	0.22		7.05	(6.63,7.46)	0.21	
Immigration status	Born in this country	6.10	(5.92,6.28)	0.09	0.937	6.36	(6.20,6.52)	0.08	0.836	6.97	(6.81,7.13)	0.08	0.449
	Born in another country	6.14	(5.33,6.94)	0.41		6.44	(5.64,7.25)	0.41		6.69	(5.98,7.40)	0.36	
Religious affiliation	Christianity	6.11	(5.91,6.31)	0.10	< .001	6.37	(6.20,6.54)	0.09	< .001	6.97	(6.80,7.14)	0.09	< .001
	Islam	6.02	(5.50,6.54)	0.26		7.39	(6.44,8.35)	0.47		6.52	(5.28,7.75)	0.61	
	Hinduism	6.00	*	*		7.00	*	*		8.00	*	*	
	Buddhism	6.17	(2.61,9.73)	0.30		7.02	(0.93,10.0ǂ)	0.65		6.68	(1.34,10.0ǂ)	0.68	
	Jainism	9.00	*	*		8.00	*	*		7.00	*	*	
	Shinto	8.51	*	*		8.81	*	*		8.81	*	*	
	Taoism	10.00	*	*		6.00	*	*		10.00	*	*	
	Primal, Animist, or Folk religion	5.57	(4.84,6.30)	0.37		5.83	(4.97,6.68)	0.43		6.39	(5.48,7.30)	0.46	
	Some other religion	3.96	*	*		5.35	*	*		6.92	*	*	
	No religion/Atheist /Agnostic	6.30	(5.84,6.77)	0.24		6.25	(5.83,6.66)	0.21		7.19	(6.81,7.57)	0.19	
Race/Ethnicity	Black	6.12	(5.95,6.30)	0.09	< .001	6.36	(6.20,6.51)	0.08	< .001	6.96	(6.80,7.13)	0.08	< .001
	White	8.36	(2.08,10.0ǂ)	1.29		5.93	(3.88,7.97)	0.42		6.41	(4.51,8.30)	0.39	
	Asian/Indian	5.14	*	*		7.86	*	*		9.71	*	*	
	Colored	5.90	(4.93,6.87)	0.49		6.36	(5.58,7.14)	0.40		6.81	(6.14,7.48)	0.34	
	Other	5.00	*	*		9.00	*	*		10.00	*	*	

Table S17c. Childhood predictors regression analysis results for South Africa

		Cantril's Ladder				Life Satisfaction				Happiness			
Variable	Category	Est	95% CI	SE	Global p-value	Est	95% CI	SE	Global p-value	Est	95% CI	SE	Global p-value
Relationship with mother	(Ref: Very bad/somewhat bad)				0.201				0.038				0.118
	Very good/somewhat good	0.45	(-0.24, 1.14)	0.35		0.85	(0.05, 1.65)	0.41		0.54	(-0.15, 1.24)	0.35	
Relationship with father	(Ref: Very bad/somewhat bad)				0.317				0.147				0.369
	Very good/somewhat good	0.23	(-0.23, 0.69)	0.24		0.30	(-0.11, 0.71)	0.21		0.19	(-0.23, 0.61)	0.21	
Parent marital status	(Ref: Parents married)				0.168				0.958				0.328
	Divorced	0.67	(-0.05, 1.38)	0.36		0.05	(-0.62, 0.72)	0.34		0.32	(-0.21, 0.85)	0.27	
	Parents were never married	-0.03	(-0.35, 0.29)	0.16		0.04	(-0.25, 0.33)	0.15		-0.14	(-0.42, 0.15)	0.15	
	One or both parents had died	0.31	(-0.37, 0.98)	0.34		0.15	(-0.63, 0.92)	0.39		0.09	(-0.60, 0.79)	0.35	
Subjective financial status of family growing up	(Ref: Got by)				0.067				0.100				0.223
	Lived comfortably	-0.22	(-0.55, 0.11)	0.17		0.15	(-0.16, 0.45)	0.15		0.03	(-0.28, 0.33)	0.15	
	Found it difficult	-0.51	(-0.98, -0.05)	0.24		-0.39	(-0.84, 0.05)	0.23		-0.27	(-0.67, 0.13)	0.20	
	Found it very difficult	-0.67	(-1.27, -0.07)	0.31		-0.18	(-0.81, 0.45)	0.32		-0.55	(-1.18, 0.08)	0.32	
Abuse	(Ref: No)				<.001				<.001				0.005
	Yes	-0.87	(-1.27, -0.48)	0.20		-0.67	(-1.05, -0.28)	0.20		-0.58	(-0.98, -0.18)	0.20	
Outsider growing up	(Ref: No)				0.491				0.654				0.919
	Yes	-0.15	(-0.58, 0.28)	0.22		0.09	(-0.30, 0.48)	0.20		0.02	(-0.39, 0.43)	0.21	
Self-rated health growing up	(Ref: Good)				0.134				0.375				0.255
	Excellent	-0.13	(-0.54, 0.29)	0.21		0.12	(-0.29, 0.53)	0.21		0.26	(-0.18, 0.70)	0.22	

Variable	Category	Cantril's Ladder				Life Satisfaction				Happiness			
		Est	95% CI	SE	Global p-value	Est	95% CI	SE	Global p-value	Est	95% CI	SE	Global p-value
	Very good	0.02	(-0.51, 0.54)	0.27		-0.03	(-0.51, 0.46)	0.25		0.03	(-0.48, 0.54)	0.26	
	Fair	-0.51	(-1.14, 0.12)	0.32		-0.42	(-1.05, 0.20)	0.32		0.03	(-0.55, 0.62)	0.30	
	Poor	-0.65	(-1.30, 0.00)	0.33		-0.11	(-0.74, 0.52)	0.32		-0.32	(-0.96, 0.32)	0.32	
Immigration status	(Ref: Born in this country)				0.978				0.895				0.423
	Born in another country	-0.01	(-0.81, 0.79)	0.41		0.05	(-0.75, 0.85)	0.41		-0.30	(-1.04, 0.44)	0.38	
Age 12 religious service attendance	(Ref: Never)				0.022				0.030				0.169
	At least 1/week	-0.81	(-1.40, -0.22)	0.30		-0.80	(-1.46, -0.15)	0.33		-0.62	(-1.25, 0.01)	0.32	
	1-3/month	-0.44	(-1.06, 0.18)	0.32		-0.55	(-1.24, 0.13)	0.35		-0.34	(-0.95, 0.27)	0.31	
	< 1/month	-0.97	(-1.76, -0.18)	0.40		-1.11	(-1.95, -0.28)	0.43		-0.73	(-1.57, 0.11)	0.43	
Year of birth	(Ref: 1998-2005; current age: 18-24)				0.897				0.103				0.236
	1993-1998; age 25-29	-0.15	(-0.55, 0.25)	0.20		-0.25	(-0.70, 0.19)	0.23		-0.41	(-0.85, 0.03)	0.22	
	1983-1993; age 30-39	-0.02	(-0.39, 0.35)	0.19		-0.02	(-0.42, 0.38)	0.20		-0.07	(-0.46, 0.31)	0.20	
	1973-1983; age 40-49	-0.20	(-0.60, 0.21)	0.21		-0.26	(-0.68, 0.15)	0.21		-0.48	(-0.91, -0.05)	0.22	
	1963-1973; age 50-59	-0.26	(-0.81, 0.29)	0.28		0.12	(-0.47, 0.71)	0.30		-0.03	(-0.59, 0.53)	0.28	
	1953-1963; age 60-69	-0.41	(-1.25, 0.42)	0.43		-0.15	(-0.98, 0.67)	0.42		-0.11	(-0.94, 0.73)	0.43	
	1943-1953; age 70-79	-0.01	(-1.04, 1.03)	0.53		0.27	(-0.72, 1.26)	0.51		0.07	(-0.96, 1.09)	0.52	
	1943 or earlier; age 80+	-0.79	(-2.85, 1.27)	1.05		0.63	(-0.00, 1.27)	0.32		0.44	(-2.28, 3.16)	1.39	
Gender	(Ref: Male)				0.297				0.811				0.676
	Female	0.20	(-0.07, 0.47)	0.14		-0.06	(-0.31, 0.19)	0.13		-0.04	(-0.31, 0.22)	0.14	
	Other	0.52	(-0.48, 1.52)	0.51		0.10	(-0.53, 0.73)	0.32		-0.54	(-1.77, 0.68)	0.63	
Religious affiliation	(Ref: No religion/Atheist /Agnostic)				0.344				0.024				0.198
	Primal, Animist, or Folk religion	-0.59	(-1.51, 0.33)	0.47		0.13	(-1.04, 1.29)	0.59		-0.83	(-1.77, 0.10)	0.48	

		Cantril's Ladder				Life Satisfaction				Happiness			
Variable	Category	Est	95% CI	SE	Global p-value	Est	95% CI	SE	Global p-value	Est	95% CI	SE	Global p-value
	Christianity	0.08	(-0.66, 0.82)	0.38		0.74	(-0.20, 1.68)	0.48		-0.07	(-0.91, 0.77)	0.43	
	Collapsed affiliations with prevalence<3%	-0.05	(-1.02, 0.93)	0.50		1.49	(0.35, 2.63)	0.58		-0.31	(-1.61, 0.98)	0.66	
Race/ethnicity	(Ref: Plurality group)				0.552				0.667				0.580
	Non-plurality groups	-0.25	(-1.08, 0.58)	0.42		-0.13	(-0.74, 0.47)	0.31		-0.16	(-0.71, 0.40)	0.28	

Table S17d. Sensitivity to unmeasured confounding of childhood predictors in South Africa

		Cantril's Ladder		Life Satisfaction		Happiness	
Variable	Category	E-value for Estimate	E-value for 95% CI	E-value for Estimate	E-value for 95% CI	E-value for Estimate	E-value for 95% CI
Relationship with mother	(Ref: Very bad/somewhat bad)						
	Very good/somewhat good	1.58	1.00	1.96	1.14	1.70	1.00
Relationship with father	(Ref: Very bad/somewhat bad)						
	Very good/somewhat good	1.36	1.00	1.44	1.00	1.34	1.00
Parent marital status	(Ref: Parents married)						
	Divorced	1.78	1.00	1.15	1.00	1.48	1.00
	Parents were never married	1.11	1.00	1.13	1.00	1.27	1.00
	One or both parents had died	1.44	1.00	1.27	1.00	1.22	1.00
Subjective financial status of family growing up	(Ref: Got by)						
	Lived comfortably	1.35	1.00	1.28	1.00	1.11	1.00
	Found it difficult	1.64	1.15	1.53	1.00	1.43	1.00
	Found it very difficult	1.79	1.18	1.31	1.00	1.71	1.00
Abuse	(Ref: No)						
	Yes	1.97	1.60	1.79	1.42	1.74	1.33
Outsider growing up	(Ref: No)						
	Yes	1.28	1.00	1.20	1.00	1.09	1.00
Self-rated health growing up	(Ref: Good)						
	Excellent	1.25	1.00	1.24	1.00	1.41	1.00
	Very good	1.09	1.00	1.10	1.00	1.11	1.00
	Fair	1.63	1.00	1.56	1.00	1.12	1.00
	Poor	1.77	1.01	1.23	1.00	1.48	1.00
Immigration status	(Ref: Born in this country)						
	Born in another country	1.05	1.00	1.15	1.00	1.46	1.00
Age 12 religious service attendance	(Ref: Never)						
	At least 1/week	1.92	1.36	1.92	1.28	1.78	1.00
	1-3/month	1.57	1.00	1.68	1.00	1.50	1.00
	< 1/month	2.07	1.31	2.22	1.42	1.89	1.00
Year of birth	(Ref: 1998-2005; current age: 18-24)						
	1993-1998; age 25-29	1.28	1.00	1.39	1.00	1.57	1.00
	1983-1993; age 30-39	1.09	1.00	1.10	1.00	1.18	1.00
	1973-1983; age 40-49	1.33	1.00	1.40	1.00	1.64	1.16
	1963-1973; age 50-59	1.40	1.00	1.24	1.00	1.11	1.00

Variable	Category	Cantril's Ladder		Life Satisfaction		Happiness	
		E-value for Estimate	E-value for 95% CI	E-value for Estimate	E-value for 95% CI	E-value for Estimate	E-value for 95% CI
	1953-1963; age 60-69	1.54	1.00	1.28	1.00	1.23	1.00
	1943-1953; age 70-79	1.06	1.00	1.41	1.00	1.18	1.00
	1943 or earlier; age 80+	1.90	1.00	1.76	1.00	1.60	1.00
Gender	(Ref: Male)						
	Female	1.33	1.00	1.16	1.00	1.14	1.00
	Other	1.64	1.00	1.22	1.00	1.70	1.00
Religious affiliation	(Ref: No religion/Atheist/Agnostic)						
	Primal, Animist, or Folk religion	1.71	1.00	1.25	1.00	2.00	1.00
	Christianity	1.19	1.00	1.86	1.00	1.18	1.00
	Collapsed affiliations with prevalence<3%	1.14	1.00	2.63	1.49	1.47	1.00
Race/ethnicity	(Ref: Plurality group)						
	Non-plurality groups	1.38	1.00	1.26	1.00	1.30	1.00

Table S17e. Complete-case supplemental analysis of means by demographic category for South Africa

		Cantril's Ladder				Life Satisfaction				Happiness			
Variable	Category	Mean	95% CI	SE	Global p-value	Mean	95% CI	SE	Global p-value	Mean	95% CI	SE	Global p-value
Age group	18-24	6.64	(6.37,6.91)	0.14	0.722	6.81	(6.53,7.10)	0.14	0.141	7.32	(7.01,7.62)	0.16	0.152
	25-29	6.38	(6.12,6.63)	0.13		6.62	(6.35,6.88)	0.14		6.96	(6.72,7.21)	0.12	
	30-39	6.52	(6.27,6.77)	0.13		6.72	(6.47,6.96)	0.12		7.17	(6.94,7.40)	0.12	
	40-49	6.36	(6.09,6.62)	0.14		6.53	(6.27,6.79)	0.13		6.88	(6.63,7.12)	0.12	
	50-59	6.67	(6.31,7.03)	0.18		6.95	(6.52,7.38)	0.22		7.30	(6.85,7.76)	0.23	
	60-69	6.25	(5.55,6.96)	0.36		6.68	(6.00,7.35)	0.34		7.38	(6.70,8.06)	0.34	
	70-79	6.47	(5.47,7.46)	0.50		7.09	(6.19,7.99)	0.45		7.77	(7.05,8.48)	0.36	
	80 or older	5.90	(3.39,8.40)	1.04		7.45	(6.68,8.23)	0.32		7.92	(4.88,10.96)	1.27	
Gender	Female	6.51	(6.31,6.71)	0.10	0.864	6.66	(6.48,6.84)	0.09	0.151	7.14	(6.95,7.33)	0.10	0.357
	Male	6.45	(6.27,6.62)	0.09		6.80	(6.61,6.99)	0.10		7.21	(7.02,7.39)	0.10	
	Other	6.58	(0.00,8819.31)	0.51		6.29	(0.00,4412.65)	0.25		6.30	(0.00,10914.17)	0.63	
Marital status	Divorced	6.09	(5.23,6.96)	0.43	0.300	5.92	(5.04,6.80)	0.43	0.024	6.61	(5.64,7.58)	0.48	0.073
	Domestic partner	6.23	(5.78,6.68)	0.23		6.66	(6.30,7.02)	0.18		7.04	(6.66,7.43)	0.20	
	Married	6.67	(6.32,7.02)	0.18		6.92	(6.59,7.26)	0.17		7.47	(7.14,7.79)	0.16	
	Separated	6.86	(6.16,7.56)	0.35		7.35	(6.55,8.15)	0.40		7.46	(6.76,8.15)	0.35	
	Single/Never been married	6.41	(6.26,6.57)	0.08		6.61	(6.46,6.77)	0.08		7.06	(6.90,7.21)	0.08	
	Widowed	6.90	(6.20,7.61)	0.36		7.40	(6.57,8.23)	0.42		7.61	(6.81,8.42)	0.41	
Employment	Employed for an employer	6.76	(6.52,7.00)	0.12	0.170	7.04	(6.82,7.27)	0.11	0.031	7.32	(7.11,7.53)	0.11	0.056
	Homemaker	6.06	(5.32,6.80)	0.37		6.41	(5.68,7.14)	0.37		7.00	(6.40,7.60)	0.30	
	None of these/Other	6.72	(5.56,7.89)	0.58		6.69	(5.51,7.88)	0.59		7.80	(6.79,8.82)	0.51	
	Retired	6.50	(5.83,7.18)	0.34		7.00	(6.40,7.61)	0.31		7.65	(7.10,8.21)	0.28	
	Self-employed	6.43	(6.15,6.71)	0.14		6.73	(6.42,7.04)	0.16		7.05	(6.69,7.41)	0.18	
	Student	6.60	(6.22,6.97)	0.19		6.66	(6.22,7.09)	0.22		7.22	(6.82,7.62)	0.20	
	Unemployed and looking for a job	6.34	(6.12,6.57)	0.11		6.54	(6.36,6.73)	0.09		6.99	(6.79,7.18)	0.10	
Religious service attendance	A few times a year	6.57	(6.33,6.82)	0.13	0.182	6.43	(6.12,6.74)	0.16	0.095	6.86	(6.56,7.15)	0.15	0.060
	More than once a week	6.66	(6.31,7.00)	0.18		6.91	(6.56,7.26)	0.18		7.47	(7.13,7.80)	0.17	
	Never	6.41	(6.04,6.78)	0.19		6.51	(6.11,6.90)	0.20		7.03	(6.67,7.39)	0.18	
	Once a week	6.57	(6.36,6.78)	0.11		6.90	(6.69,7.12)	0.11		7.28	(7.06,7.49)	0.11	

		Cantril's Ladder				Life Satisfaction				Happiness			
Variable	Category	Mean	95% CI	SE	Global p-value	Mean	95% CI	SE	Global p-value	Mean	95% CI	SE	Global p-value
	One to three times a month	6.19	(5.88,6.50)	0.16		6.67	(6.39,6.95)	0.14		7.11	(6.80,7.43)	0.16	
Education	Up to 8 years	6.67	(6.25,7.08)	0.21	0.082	6.99	(6.64,7.34)	0.18	0.149	7.34	(6.97,7.71)	0.19	0.453
	16+ years	6.77	(6.40,7.14)	0.19		6.78	(6.41,7.15)	0.19		7.22	(6.83,7.61)	0.20	
	9 to 15 years	6.39	(6.25,6.53)	0.07		6.63	(6.49,6.76)	0.07		7.11	(6.96,7.25)	0.07	
Immigration status	Born in another country	6.55	(5.90,7.21)	0.33	0.809	6.76	(6.16,7.36)	0.30	0.918	6.78	(6.07,7.48)	0.36	0.256
	Born in this country	6.48	(6.33,6.62)	0.07		6.73	(6.59,6.87)	0.07		7.19	(7.05,7.34)	0.08	
Religious affiliation	Buddhism	6.17	(1.15,11.20)	0.31	*	7.04	(0.00,17.99)	0.67	< .001	6.62	(0.00,17.88)	0.69	< .001
	Christianity	6.49	(6.33,6.65)	0.08		6.71	(6.56,6.86)	0.08		7.16	(6.99,7.32)	0.08	
	Hinduism	6.00	*	*		7.00	*	*		8.00	*	*	
	Islam	6.05	(5.56,6.54)	0.24		7.39	(6.44,8.35)	0.47		7.11	(6.24,7.98)	0.43	
	No religion/Atheist/Agnostic	6.74	(6.35,7.13)	0.20		6.61	(6.24,6.98)	0.19		7.25	(6.90,7.59)	0.17	
	Primal, Animist, or Folk religion	6.03	(5.41,6.66)	0.31		6.94	(6.40,7.47)	0.27		7.33	(6.80,7.87)	0.27	
	Some other religion	3.93	*	*		5.33	*	*		6.38	*	*	
	Taoism	10.00	*	*		6.00	*	*		10.00	*	*	
	Jainism	9.00	*	*		8.00	*	*		7.00	*	*	
	Shinto	8.51	*	*		8.81	*	*		8.81	*	*	

Table S17f. Complete-case supplemental analysis of childhood predictors regression analysis results for South Africa

		Cantril's Ladder				Life Satisfaction				Happiness			
Variable	Category	Est	95% CI	SE	Global p-value	Est	95% CI	SE	Global p-value	Est	95% CI	SE	Global p-value
Relationship with mother	(Ref: Very bad/somewhat bad)				0.150				0.064				0.144
	Very good/somewhat good	0.49	(-0.17,1.15)	0.34		0.63	(-0.03,1.29)	0.34		0.45	(-0.15,1.05)	0.31	
Relationship with father	(Ref: Very bad/somewhat bad)				0.279				0.156				0.714
	Very good/somewhat good	0.20	(-0.16,0.55)	0.18		0.25	(-0.09,0.59)	0.17		0.07	(-0.31,0.45)	0.20	
Parent marital status	(Ref: Parents married)				0.113				0.208				0.553
	No, one or both of them had died	-0.40	(-1.13,0.34)	0.37		0.48	(-0.22,1.18)	0.36		-0.11	(-0.81,0.59)	0.36	
	No, they were never married	-0.61	(-1.14,-0.08)	0.27		-0.16	(-0.62,0.31)	0.24		-0.31	(-0.75,0.13)	0.23	
	Yes, married	-0.61	(-1.12,-0.10)	0.26		-0.12	(-0.60,0.36)	0.24		-0.23	(-0.68,0.22)	0.23	
Subjective financial status of family growing up	(Ref: Got by)				0.329				0.172				0.172
	Found it difficult	-0.26	(-0.65,0.12)	0.20		-0.02	(-0.41,0.38)	0.20		-0.14	(-0.53,0.24)	0.20	
	Found it very difficult	-0.44	(-1.00,0.12)	0.29		0.09	(-0.51,0.69)	0.31		-0.34	(-0.93,0.24)	0.30	
	Lived comfortably	-0.07	(-0.35,0.21)	0.14		0.28	(0.00,0.55)	0.14		0.19	(-0.07,0.44)	0.13	
Abuse	(Ref: No)				0.007				0.029				0.172
	Yes	-0.48	(-0.82,-0.13)	0.18		-0.38	(-0.73,-0.04)	0.17		-0.25	(-0.60,0.11)	0.18	
Outsider growing up	(Ref: No)				0.985				0.158				0.688
	Yes	0.00	(-0.36,0.37)	0.19		0.24	(-0.09,0.56)	0.17		0.07	(-0.26,0.40)	0.17	

		Cantril's Ladder				Life Satisfaction				Happiness			
Variable	Category	Est	95% CI	SE	Global p-value	Est	95% CI	SE	Global p-value	Est	95% CI	SE	Global p-value
Self-rated health growing up	(Ref: Good)				0.139				0.458				0.222
	Excellent	-0.08	(-0.46,0.31)	0.20		0.16	(-0.22,0.53)	0.19		0.21	(-0.14,0.55)	0.18	
	Fair	-0.23	(-0.76,0.31)	0.27		-0.18	(-0.71,0.36)	0.27		0.02	(-0.50,0.55)	0.27	
	Poor	-0.53	(-1.10,0.04)	0.29		-0.24	(-0.84,0.35)	0.30		-0.40	(-0.95,0.15)	0.28	
	Very good	0.19	(-0.26,0.64)	0.23		0.15	(-0.26,0.55)	0.21		0.25	(-0.14,0.64)	0.20	
Immigration status	(Ref: Born in this country)				0.891				0.936				0.286
	Born in another country	0.05	(-0.63,0.72)	0.34		-0.02	(-0.62,0.57)	0.30		-0.40	(-1.12,0.33)	0.37	
Age 12 religious service attendance	(Ref: Never)				0.334				0.040				0.463
	At least once a week	-0.40	(-0.96,0.17)	0.29		-0.56	(-1.15,0.03)	0.30		-0.16	(-0.73,0.41)	0.29	
	Less than once a month	-0.64	(-1.39,0.12)	0.38		-0.86	(-1.63,-0.09)	0.39		-0.12	(-0.83,0.59)	0.36	
	One to three times a month	-0.23	(-0.81,0.35)	0.30		-0.31	(-0.93,0.31)	0.32		0.09	(-0.47,0.65)	0.28	
Year of birth	(Ref: 1998-2005; current age: 18-24)				0.651				0.203				0.171
	1993-1998; age 25-29	-0.24	(-0.60,0.13)	0.19		-0.15	(-0.52,0.23)	0.19		-0.31	(-0.73,0.10)	0.21	
	1983-1993; age 30-39	-0.08	(-0.42,0.26)	0.17		-0.09	(-0.45,0.27)	0.18		-0.08	(-0.46,0.30)	0.19	
	1973-1983; age 40-49	-0.19	(-0.55,0.17)	0.18		-0.25	(-0.63,0.12)	0.19		-0.33	(-0.72,0.07)	0.20	
	1963-1973; age 50-59	0.15	(-0.28,0.58)	0.22		0.19	(-0.32,0.70)	0.26		0.11	(-0.41,0.62)	0.26	
	1953-1963; age 60-69	-0.24	(-0.99,0.51)	0.38		-0.12	(-0.82,0.57)	0.36		0.17	(-0.51,0.85)	0.35	
	1943-1953; age 70-79	-0.01	(-0.98,0.96)	0.50		0.23	(-0.70,1.16)	0.47		0.55	(-0.21,1.30)	0.39	

		Cantril's Ladder				Life Satisfaction				Happiness			
Variable	Category	Est	95% CI	SE	Global p-value	Est	95% CI	SE	Global p-value	Est	95% CI	SE	Global p-value
	1943 or earlier; age 80+	-0.88	(-2.95,1.18)	1.05		0.52	(-0.09,1.13)	0.31		0.63	(-2.13,3.38)	1.40	
Gender	(Ref: Male)				0.898				0.319				0.248
	Male	-0.05	(-0.28,0.17)	0.12		0.18	(-0.06,0.42)	0.12		0.13	(-0.10,0.37)	0.12	
	Other	-0.01	(-1.00,0.98)	0.50		-0.07	(-0.74,0.60)	0.34		-0.76	(-1.96,0.43)	0.61	
Religious affiliation	(Ref: No religion/Atheist/Agnostic)				0.275				0.267				0.488
	Christianity	-0.02	(-0.70,0.67)	0.35		0.48	(-0.32,1.27)	0.41		-0.55	(-1.28,0.18)	0.37	
	Christianity	-0.02	(-0.70,0.67)	0.35		0.48	(-0.32,1.27)	0.41		-0.55	(-1.28,0.18)	0.37	
	Primal, Animist, or Folk religion	-0.55	(-1.49,0.39)	0.48		0.16	(-0.89,1.21)	0.54		-0.58	(-1.39,0.22)	0.41	
	Collapsed affiliations with prevalence<3%	-0.51	(-1.42,0.41)	0.47		0.90	(-0.12,1.92)	0.52		-0.56	(-1.53,0.41)	0.49	
Race/ethnicity	(Ref: Plurality group)				0.853				0.927				0.652
	Non-plurality groups	-0.05	(-0.58,0.48)	0.27		-0.02	(-0.49,0.45)	0.24		0.12	(-0.40,0.64)	0.27	

Table S17g. Complete-case supplemental analysis of sensitivity to unmeasured confounding of childhood predictors in South Africa

		Cantril's Ladder		Life Satisfaction		Happiness	
Variable	Category	E-value for Estimate	E-value for 95% CI	E-value for Estimate	E-value for 95% CI	E-value for Estimate	E-value for 95% CI
Relationship with mother	(Ref: Very bad/somewhat bad)						
	Very good/somewhat good	1.68	1.00	1.85	1.00	1.66	1.00
Relationship with father	(Ref: Very bad/somewhat bad)						
	Very good/somewhat good	1.36	1.00	1.42	1.00	1.20	1.00
Parent marital status	(Ref: Parents married)						
	No, one or both of them had died	1.58	1.00	1.68	1.00	1.26	1.00
	No, they were never married	1.81	1.21	1.31	1.00	1.50	1.00
	Yes, married	1.81	1.23	1.26	1.00	1.41	1.00
Subjective financial status of family growing up	(Ref: Got by)						
	Found it difficult	1.44	1.00	1.08	1.00	1.30	1.00
	Found it very difficult	1.63	1.00	1.22	1.00	1.54	1.00
	Lived comfortably	1.19	1.00	1.46	1.03	1.36	1.00
Abuse	(Ref: No)						
	Yes	1.67	1.28	1.58	1.14	1.43	1.00
Outsider growing up	(Ref: No)						
	Yes	1.04	1.00	1.41	1.00	1.19	1.00
Self-rated health growing up	(Ref: Good)						
	Excellent	1.20	1.00	1.31	1.00	1.38	1.00
	Fair	1.39	1.00	1.34	1.00	1.10	1.00
	Poor	1.73	1.00	1.42	1.00	1.60	1.00
	Very good	1.35	1.00	1.30	1.00	1.43	1.00
Immigration status	(Ref: Born in this country)						
	Born in another country	1.15	1.00	1.11	1.00	1.60	1.00
Age 12 religious service attendance	(Ref: Never)						
	At least once a week	1.58	1.00	1.77	1.00	1.32	1.00
	Less than once a month	1.84	1.00	2.11	1.23	1.26	1.00
	One to three times a month	1.40	1.00	1.49	1.00	1.23	1.00
Year of birth	(Ref: 1998-2005; current age: 18-24)						

Variable	Category	Cantril's Ladder		Life Satisfaction		Happiness	
		E-value for Estimate	E-value for 95% CI	E-value for Estimate	E-value for 95% CI	E-value for Estimate	E-value for 95% CI
	1993-1998; age 25-29	1.40	1.00	1.30	1.00	1.51	1.00
	1983-1993; age 30-39	1.21	1.00	1.23	1.00	1.22	1.00
	1973-1983; age 40-49	1.35	1.00	1.43	1.00	1.52	1.00
	1963-1973; age 50-59	1.30	1.00	1.35	1.00	1.25	1.00
	1953-1963; age 60-69	1.41	1.00	1.27	1.00	1.34	1.00
	1943-1953; age 70-79	1.06	1.00	1.40	1.00	1.77	1.00
	1943 or earlier; age 80+	2.11	1.00	1.73	1.00	1.86	1.00
Gender	(Ref: Male)						
	Male	1.16	1.00	1.34	1.00	1.28	1.00
	Other	1.08	1.00	1.19	1.00	2.01	1.00
Religious affiliation	(Ref: No religion/Atheist/Agnostic)						
	Christianity	1.09	1.00	1.68	1.00	1.77	1.00
	Christianity	1.09	1.00	1.68	1.00	1.77	1.00
	Primal, Animist, or Folk religion	1.75	1.00	1.31	1.00	1.80	1.00
	Collapsed affiliations with prevalence<3%	1.70	1.00	2.15	1.00	1.78	1.00
Race/ethnicity	(Ref: Plurality group)						
	Non-plurality groups	1.16	1.00	1.10	1.00	1.27	1.00

Tables S18a-g: Spain

Table S18a. Nationally representative descriptive statistics for Spain

Characteristic	N = 6,290[1]
**Age group**	
1998-2005; age 18-24	594 (9.4%)
1993-1998; age 25-29	450 (7.2%)
1983-1993; age 30-39	1,111 (18%)
1973-1983; age 40-49	1,396 (22%)
1963-1973; age 50-59	1,252 (20%)
1953-1963; age 60-69	977 (16%)
1943-1953; age 70-79	467 (7.4%)
1943 or earlier; age 80+	43 (0.7%)
(Missing)	0 (0%)
**Gender**	
Male	3,142 (50%)
Female	3,119 (50%)
Other	6 (0.1%)
(Missing)	22 (0.4%)
**Respondent Marital status**	
Married	2,947 (47%)
Separated	237 (3.8%)
Divorced	518 (8.2%)
Widowed	189 (3.0%)
Single, never married	1,742 (28%)
Domestic Partner	589 (9.4%)
(Missing)	67 (1.1%)
**Employment**	
Employed for an employer	2,862 (45%)
Self-employed	576 (9.2%)
Retired	1,278 (20%)
Student	448 (7.1%)
Homemaker	345 (5.5%)
Unemployed and looking for a job	646 (10%)
None of these/Other	123 (2.0%)
(Missing)	11 (0.2%)
**Religious service attendance as an adult (now)**	
More than 1/week	317 (5.0%)
1/week	662 (11%)
1-3/month	437 (6.9%)
A few times a year	1,972 (31%)
Never	2,875 (46%)
(Missing)	27 (0.4%)
**Education (years)**	
Up to 8 years	802 (13%)
9-15 years	4,145 (66%)
16+ years	1,341 (21%)
(Missing)	2 (<0.1%)
**Immigration status**	
Born in this country	5,479 (87%)
Born in another country	788 (13%)
(Missing)	23 (0.4%)
**Religious affiliation as an adult (now)**	
Christianity	4,074 (65%)
Islam	135 (2.1%)
Hinduism	7 (0.1%)
Buddhism	36 (0.6%)
Judaism	4 (<0.1%)
Sikhism	3 (<0.1%)
Baha'i	2 (<0.1%)
Jainism	1 (<0.1%)
Shinto	0 (0%)
Taoism	5 (<0.1%)
Confucianism	3 (<0.1%)
Primal, Animist, or Folk religion	7 (0.1%)

Characteristic	N = 6,290[1]
Spiritism	0 (0%)
Umbanda, Candomble, and other African-derived religions	0 (0%)
Chinese folk/traditional religion	0 (0%)
Some other religion	27 (0.4%)
No religion/Atheist/Agnostic	1,932 (31%)
(Missing)	55 (0.9%)
**Relationship with mother growing up**	
Very good	4,557 (72%)
Somewhat good	1,258 (20%)
Somewhat bad	248 (3.9%)
Very bad	92 (1.5%)
Does not apply	107 (1.7%)
(Missing)	28 (0.4%)
**Relationship with father growing up**	
Very good	4,131 (66%)
Somewhat good	1,397 (22%)
Somewhat bad	309 (4.9%)
Very bad	178 (2.8%)
Does not apply	243 (3.9%)
(Missing)	33 (0.5%)
**Parent marital status at age 12**	
Parents married	5,285 (84%)
Divorced	378 (6.0%)
Parents were never married	312 (5.0%)
One or both parents had died	126 (2.0%)
(Missing)	188 (3.0%)
**Subjective financial status of family growing up**	
Lived comfortably	2,041 (32%)
Got by	2,956 (47%)
Found it difficult	1,154 (18%)
Found it very difficult	110 (1.7%)
(Missing)	29 (0.5%)
**Abuse**	
Yes	659 (10%)
No	5,510 (88%)
(Missing)	122 (1.9%)
**Outsider growing up**	
Yes	579 (9.2%)
No	5,637 (90%)
(Missing)	75 (1.2%)
**Self-rated health growing up**	
Excellent	2,450 (39%)
Very good	2,286 (36%)
Good	1,235 (20%)
Fair	164 (2.6%)
Poor	135 (2.1%)
(Missing)	20 (0.3%)
**Age 12 religious service attendance**	
At least 1/week	2,391 (38%)
1-3/month	1,132 (18%)
<1/month	1,287 (20%)
Never	1,445 (23%)
(Missing)	36 (0.6%)
**Religious affiliation at age 12**	
Christianity	5,119 (81%)
Islam	132 (2.1%)
Hinduism	5 (<0.1%)
Buddhism	8 (0.1%)
Judaism	5 (<0.1%)
Sikhism	2 (<0.1%)
Baha'i	0 (0%)
Jainism	0 (0%)
Shinto	0 (0%)
Taoism	0 (0%)
Confucianism	1 (<0.1%)

**Characteristic**	**N = 6,290**[1]
Primal, Animist, or Folk religion	4 (<0.1%)
Spiritism	0 (0%)
Umbanda, Candomble, and other African-derived religions	0 (0%)
Chinese folk/traditional religion	0 (0%)
Some other religion	13 (0.2%)
No religion/Atheist/Agnostic	972 (15%)
(Missing)	29 (0.5%)

[1]n (%)

Table S178. Means by demographic category for Spain

Variable	Category	Cantril's Ladder				Life Satisfaction				Happiness			
		Mean	95% CI	SE	Global p-value	Mean	95% CI	SE	Global p-value	Mean	95% CI	SE	Global p-value
Age group	18-24	6.79	(6.60,6.97)	0.09	0.200	6.73	(6.51,6.94)	0.11	0.646	6.75	(6.55,6.95)	0.10	0.162
	25-29	6.88	(6.70,7.06)	0.09		6.88	(6.65,7.11)	0.12		7.08	(6.89,7.28)	0.10	
	30-39	6.68	(6.56,6.80)	0.06		6.85	(6.71,6.99)	0.07		6.89	(6.77,7.02)	0.06	
	40-49	6.66	(6.55,6.76)	0.05		6.78	(6.66,6.91)	0.06		6.95	(6.83,7.06)	0.06	
	50-59	6.57	(6.43,6.72)	0.07		6.69	(6.54,6.85)	0.08		6.90	(6.75,7.04)	0.07	
	60-69	6.65	(6.45,6.84)	0.10		6.81	(6.58,7.04)	0.12		6.95	(6.76,7.15)	0.10	
	70-79	6.56	(6.19,6.94)	0.19		6.79	(6.38,7.20)	0.21		6.89	(6.50,7.28)	0.20	
	80 or older	7.04	(6.29,7.80)	0.37		7.34	(6.26,8.43)	0.52		7.69	(6.95,8.43)	0.36	
Gender	Male	6.72	(6.62,6.81)	0.05	0.243	6.89	(6.79,6.99)	0.05	0.008	7.00	(6.90,7.09)	0.05	0.049
	Female	6.62	(6.53,6.70)	0.04		6.68	(6.58,6.78)	0.05		6.84	(6.75,6.93)	0.05	
	Other	7.10	(3.74,10.0ǂ)	0.67		7.21	(5.19,9.24)	0.44		7.10	(5.54,8.66)	0.31	
Marital status	Married	6.81	(6.72,6.91)	0.05	< .001	6.97	(6.86,7.08)	0.06	< .001	7.12	(7.02,7.22)	0.05	< .001
	Separated	6.31	(5.95,6.68)	0.18		6.46	(6.08,6.84)	0.19		6.55	(6.22,6.89)	0.17	
	Divorced	6.41	(6.15,6.67)	0.13		6.58	(6.34,6.82)	0.12		6.74	(6.50,6.97)	0.12	
	Widowed	6.20	(5.70,6.70)	0.25		6.36	(5.80,6.93)	0.29		6.56	(6.07,7.05)	0.25	
	Never	6.60	(6.49,6.71)	0.06		6.62	(6.50,6.75)	0.06		6.71	(6.60,6.82)	0.06	
	Domestic Partner	6.64	(6.47,6.81)	0.09		6.81	(6.60,7.01)	0.10		6.98	(6.79,7.17)	0.10	
Employment	Employed for an employer	6.80	(6.73,6.88)	0.04	< .001	6.91	(6.83,7.00)	0.04	< .001	7.07	(6.99,7.15)	0.04	< .001
	Self-employed	6.74	(6.57,6.90)	0.09		6.77	(6.58,6.97)	0.10		6.87	(6.69,7.06)	0.09	
	Retired	6.68	(6.49,6.88)	0.10		6.81	(6.59,7.04)	0.11		6.91	(6.71,7.12)	0.10	
	Student	6.79	(6.57,7.00)	0.11		6.74	(6.49,6.99)	0.13		6.69	(6.46,6.92)	0.12	
	Homemaker	6.44	(6.09,6.79)	0.18		6.76	(6.40,7.13)	0.18		6.99	(6.70,7.28)	0.15	
	Unemployed and looking for a job	6.05	(5.87,6.23)	0.09		6.25	(6.04,6.46)	0.11		6.47	(6.27,6.67)	0.10	
	None of these/Other	6.36	(5.94,6.78)	0.21		6.75	(6.30,7.20)	0.23		6.83	(6.42,7.23)	0.20	
Religious service attendance	More than 1/week	7.21	(6.86,7.57)	0.18	< .001	7.21	(6.83,7.58)	0.19	< .001	7.41	(7.06,7.77)	0.18	< .001
	1/week	7.16	(6.99,7.33)	0.09		7.26	(7.07,7.45)	0.10		7.41	(7.23,7.60)	0.10	
	1-3/month	6.87	(6.66,7.09)	0.11		7.01	(6.76,7.25)	0.12		7.27	(7.04,7.50)	0.12	
	A few times a year	6.63	(6.51,6.75)	0.06		6.75	(6.62,6.88)	0.07		6.91	(6.80,7.03)	0.06	
	Never	6.48	(6.39,6.57)	0.05		6.63	(6.52,6.73)	0.05		6.70	(6.61,6.80)	0.05	
Education	Up to 8 years	6.49	(6.22,6.76)	0.14	< .001	6.74	(6.45,7.03)	0.15	< .001	6.95	(6.68,7.21)	0.14	< .001
	9-15 years	6.60	(6.53,6.68)	0.04		6.71	(6.63,6.79)	0.04		6.83	(6.76,6.91)	0.04	

Variable	Category	Cantril's Ladder				Life Satisfaction				Happiness			
		Mean	95% CI	SE	Global p-value	Mean	95% CI	SE	Global p-value	Mean	95% CI	SE	Global p-value
	16+ years	6.97	(6.86,7.07)	0.05		7.05	(6.92,7.18)	0.07		7.17	(7.06,7.29)	0.06	
Immigration status	Born in this country	6.62	(6.56,6.69)	0.03	< .001	6.72	(6.65,6.80)	0.04	< .001	6.86	(6.78,6.93)	0.04	< .001
	Born in another country	6.97	(6.80,7.14)	0.09		7.23	(7.05,7.41)	0.09		7.37	(7.21,7.54)	0.08	
Religious affiliation	Christianity	6.73	(6.65,6.81)	0.04	< .001	6.87	(6.78,6.96)	0.05	< .001	7.02	(6.94,7.11)	0.04	< .001
	Islam	7.08	(6.63,7.53)	0.23		6.78	(6.20,7.36)	0.29		6.94	(6.47,7.41)	0.24	
	Hinduism	6.41	*	*		5.11	*	*		6.73	*	*	
	Buddhism	6.44	(5.81,7.08)	0.31		6.29	(5.61,6.97)	0.33		6.57	(5.80,7.34)	0.37	
	Judaism	7.96	*	*		7.27	*	*		8.27	*	*	
	Sikhism	6.97	*	*		6.52	*	*		7.94	*	*	
	Baha'i	6.60	*	*		7.20	*	*		7.20	*	*	
	Jainism	6.00	*	*		4.00	*	*		4.00	*	*	
	Taoism	6.07	*	*		5.87	*	*		5.49	*	*	
	Confucianism	2.79	*	*		3.02	*	*		2.79	*	*	
	Primal, Animist, or Folk religion	6.48	*	*		6.05	*	*		6.01	*	*	
	Some other religion	7.07	(5.93,8.22)	0.52		7.16	(5.65,8.67)	0.69		7.47	(6.21,8.74)	0.58	
	No religion/Atheist /Agnostic	6.52	(6.41,6.62)	0.05		6.63	(6.51,6.75)	0.06		6.71	(6.60,6.82)	0.06	

Table S18c. Childhood predictors regression analysis results for Spain

Variable	Category	Cantril's Ladder				Life Satisfaction				Happiness			
		Est	95% CI	SE	Global p-value	Est	95% CI	SE	Global p-value	Est	95% CI	SE	Global p-value
Relationship with mother	(Ref: Very bad/somewhat bad)				<.001				<.001				<.001
	Very good/somewhat good	0.64	(0.36, 0.93)	0.15		0.56	(0.21, 0.91)	0.18		0.65	(0.35, 0.95)	0.15	
Relationship with father	(Ref: Very bad/somewhat bad)				0.396				0.201				0.242
	Very good/somewhat good	0.10	(-0.13, 0.33)	0.12		0.17	(-0.09, 0.43)	0.13		0.13	(-0.09, 0.35)	0.11	
Parent marital status	(Ref: Parents married)				0.497				0.129				0.136
	Divorced	0.13	(-0.13, 0.39)	0.13		0.12	(-0.18, 0.43)	0.16		0.03	(-0.23, 0.29)	0.13	
	Parents were never married	0.03	(-0.24, 0.29)	0.14		0.06	(-0.22, 0.33)	0.14		0.07	(-0.19, 0.33)	0.13	
	One or both parents had died	-0.20	(-0.58, 0.19)	0.20		-0.48	(-0.94, -0.03)	0.23		-0.46	(-0.92, 0.01)	0.23	
Subjective financial status of family growing up	(Ref: Got by)				<.001				0.002				0.120
	Lived comfortably	0.22	(0.08, 0.36)	0.07		0.22	(0.07, 0.38)	0.08		0.11	(-0.03, 0.26)	0.07	
	Found it difficult	-0.18	(-0.37, 0.00)	0.09		-0.18	(-0.40, 0.04)	0.11		-0.11	(-0.30, 0.09)	0.10	
	Found it very difficult	-0.44	(-0.98, 0.11)	0.28		-0.38	(-1.05, 0.29)	0.34		-0.30	(-0.85, 0.24)	0.28	
Abuse	(Ref: No)				0.004				<.001				<.001
	Yes	-0.29	(-0.50, -0.09)	0.11		-0.45	(-0.68, -0.22)	0.12		-0.42	(-0.62, -0.22)	0.10	
Outsider growing up	(Ref: No)				0.220				0.006				0.038
	Yes	-0.14	(-0.36, 0.08)	0.11		-0.38	(-0.66, -0.11)	0.14		-0.22	(-0.43, -0.01)	0.11	
Self-rated health growing up	(Ref: Good)				<.001				<.001				<.001
	Excellent	0.38	(0.21, 0.56)	0.09		0.46	(0.25, 0.67)	0.11		0.67	(0.49, 0.86)	0.10	

Variable	Category	Cantril's Ladder				Life Satisfaction				Happiness			
		Est	95% CI	SE	Global p-value	Est	95% CI	SE	Global p-value	Est	95% CI	SE	Global p-value
	Very good	0.22	(0.05, 0.39)	0.09		0.29	(0.08, 0.49)	0.10		0.35	(0.17, 0.53)	0.09	
	Fair	-0.14	(-0.56, 0.29)	0.22		-0.19	(-0.68, 0.29)	0.25		-0.03	(-0.47, 0.41)	0.23	
	Poor	-0.12	(-0.73, 0.50)	0.31		0.34	(-0.32, 1.01)	0.34		0.27	(-0.33, 0.86)	0.30	
Immigration status	(Ref: Born in this country)				<.001				<.001				<.001
	Born in another country	0.44	(0.26, 0.62)	0.09		0.62	(0.43, 0.82)	0.10		0.63	(0.45, 0.81)	0.09	
Age 12 religious service attendance	(Ref: Never)				0.002				0.007				0.002
	At least 1/week	0.13	(-0.06, 0.32)	0.10		0.12	(-0.09, 0.33)	0.11		0.21	(0.02, 0.40)	0.10	
	1-3/month	0.34	(0.14, 0.53)	0.10		0.24	(0.03, 0.45)	0.11		0.29	(0.09, 0.48)	0.10	
	< 1/month	0.06	(-0.13, 0.26)	0.10		-0.09	(-0.31, 0.13)	0.11		-0.01	(-0.21, 0.19)	0.10	
Year of birth	(Ref: 1998-2005; current age: 18-24)				0.331				0.769				0.228
	1993-1998; age 25-29	0.05	(-0.20, 0.30)	0.13		0.10	(-0.20, 0.40)	0.15		0.26	(-0.00, 0.53)	0.14	
	1983-1993; age 30-39	-0.13	(-0.34, 0.08)	0.11		0.08	(-0.17, 0.32)	0.13		0.08	(-0.14, 0.30)	0.11	
	1973-1983; age 40-49	-0.15	(-0.35, 0.06)	0.10		0.02	(-0.22, 0.26)	0.12		0.12	(-0.10, 0.34)	0.11	
	1963-1973; age 50-59	-0.21	(-0.43, 0.02)	0.12		-0.03	(-0.29, 0.23)	0.13		0.11	(-0.13, 0.35)	0.12	
	1953-1963; age 60-69	-0.10	(-0.37, 0.16)	0.14		0.09	(-0.22, 0.39)	0.16		0.17	(-0.10, 0.45)	0.14	
	1943-1953; age 70-79	-0.13	(-0.53, 0.27)	0.21		0.11	(-0.34, 0.57)	0.23		0.16	(-0.26, 0.58)	0.21	
	1943 or earlier; age 80+	0.26	(-0.48, 1.01)	0.38		0.67	(-0.23, 1.56)	0.46		0.87	(0.18, 1.57)	0.36	
Gender	(Ref: Male)				0.675				0.058				0.337
	Female	-0.05	(-0.17, 0.08)	0.06		-0.17	(-0.31, -0.03)	0.07		-0.10	(-0.22, 0.03)	0.06	
	Other	0.21	(-0.77, 1.19)	0.50		0.13	(-0.67, 0.93)	0.41		-0.10	(-0.96, 0.76)	0.44	
Religious affiliation	(Ref: No religion/Atheist /Agnostic)				0.081				0.764				0.040
	Christianity	0.14	(-0.04, 0.32)	0.09		0.08	(-0.13, 0.28)	0.10		0.23	(0.05, 0.41)	0.09	

		Cantril's Ladder				Life Satisfaction				Happiness			
Variable	Category	Est	95% CI	SE	Global p-value	Est	95% CI	SE	Global p-value	Est	95% CI	SE	Global p-value
Race/ethnicity	Collapsed affiliations with prevalence<3% (Ref: Plurality group)	0.47	(0.03, 0.91)	0.22		0.08	(-0.46, 0.63)	0.28		0.32	(-0.13, 0.77)	0.23	

Table S18d. Sensitivity to unmeasured confounding of childhood predictors in Spain

		Cantril's Ladder		Life Satisfaction		Happiness	
Variable	Category	E-value for Estimate	E-value for 95% CI	E-value for Estimate	E-value for 95% CI	E-value for Estimate	E-value for 95% CI
Relationship with mother	(Ref: Very bad/somewhat bad)						
	Very good/somewhat good	2.05	1.65	1.84	1.41	2.02	1.63
Relationship with father	(Ref: Very bad/somewhat bad)						
	Very good/somewhat good	1.27	1.00	1.35	1.00	1.31	1.00
Parent marital status	(Ref: Parents married)						
	Divorced	1.32	1.00	1.29	1.00	1.13	1.00
	Parents were never married	1.12	1.00	1.18	1.00	1.22	1.00
	One or both parents had died	1.42	1.00	1.74	1.12	1.76	1.03
Subjective financial status of family growing up	(Ref: Got by)						
	Lived comfortably	1.46	1.23	1.43	1.20	1.29	1.00
	Found it difficult	1.41	1.00	1.37	1.00	1.28	1.00
	Found it very difficult	1.76	1.00	1.62	1.00	1.56	1.00
Abuse	(Ref: No)						
	Yes	1.56	1.25	1.71	1.42	1.71	1.44
Outsider growing up	(Ref: No)						
	Yes	1.34	1.00	1.62	1.27	1.44	1.07
Self-rated health growing up	(Ref: Good)						
	Excellent	1.69	1.44	1.72	1.46	2.05	1.80
	Very good	1.46	1.19	1.51	1.23	1.62	1.37
	Fair	1.33	1.00	1.38	1.00	1.13	1.00
	Poor	1.30	1.00	1.58	1.00	1.51	1.00
Immigration status	(Ref: Born in this country)						
	Born in another country	1.76	1.51	1.92	1.69	1.99	1.75
Age 12 religious service attendance	(Ref: Never)						
	At least 1/week	1.32	1.00	1.28	1.00	1.43	1.10
	1-3/month	1.62	1.35	1.45	1.13	1.54	1.25
	< 1/month	1.20	1.00	1.24	1.00	1.06	1.00
Year of birth	(Ref: 1998-2005; current age: 18-24)						
	1993-1998; age 25-29	1.18	1.00	1.25	1.00	1.50	1.00
	1983-1993; age 30-39	1.32	1.00	1.22	1.00	1.23	1.00
	1973-1983; age 40-49	1.35	1.00	1.11	1.00	1.30	1.00
	1963-1973; age 50-59	1.44	1.00	1.13	1.00	1.28	1.00

Variable	Category	Cantril's Ladder		Life Satisfaction		Happiness	
		E-value for Estimate	E-value for 95% CI	E-value for Estimate	E-value for 95% CI	E-value for Estimate	E-value for 95% CI
	1953-1963; age 60-69	1.28	1.00	1.24	1.00	1.38	1.00
	1943-1953; age 70-79	1.32	1.00	1.27	1.00	1.36	1.00
	1943 or earlier; age 80+	1.52	1.00	1.97	1.00	2.34	1.39
Gender	(Ref: Male)						
	Female	1.18	1.00	1.35	1.12	1.26	1.00
	Other	1.44	1.00	1.30	1.00	1.27	1.00
Religious affiliation	(Ref: No religion/Atheist/Agnostic)						
	Christianity	1.34	1.00	1.22	1.00	1.46	1.17
	Collapsed affiliations with prevalence<3%	1.81	1.14	1.23	1.00	1.58	1.00
Race/ethnicity	(Ref: Plurality group)						

Table S18e. Complete-case supplemental analysis of means by demographic category for Spain

		Cantril's Ladder				Life Satisfaction				Happiness			
Variable	Category	Mean	95% CI	SE	Global p-value	Mean	95% CI	SE	Global p-value	Mean	95% CI	SE	Global p-value
Age group	18-24	6.86	(6.68,7.04)	0.09	0.183	6.81	(6.60,7.02)	0.11	0.537	6.81	(6.62,7.01)	0.10	0.143
	25-29	6.95	(6.78,7.13)	0.09		7.06	(6.85,7.27)	0.11		7.18	(6.99,7.36)	0.09	
	30-39	6.73	(6.62,6.85)	0.06		6.93	(6.80,7.07)	0.07		6.97	(6.85,7.09)	0.06	
	40-49	6.69	(6.59,6.80)	0.05		6.89	(6.77,7.00)	0.06		6.99	(6.88,7.10)	0.06	
	50-59	6.68	(6.56,6.81)	0.06		6.85	(6.72,6.99)	0.07		6.98	(6.85,7.11)	0.07	
	60-69	6.72	(6.53,6.90)	0.09		6.97	(6.77,7.17)	0.10		6.99	(6.80,7.18)	0.10	
	70-79	6.76	(6.43,7.08)	0.17		6.87	(6.47,7.27)	0.20		6.95	(6.57,7.32)	0.19	
	80 or older	7.14	(6.40,7.88)	0.36		7.53	(6.61,8.46)	0.45		7.69	(6.95,8.43)	0.36	
Gender	Female	6.70	(6.61,6.78)	0.04	0.255	6.83	(6.74,6.92)	0.05	0.059	6.91	(6.82,6.99)	0.04	0.050
	Male	6.79	(6.71,6.87)	0.04		6.99	(6.89,7.08)	0.05		7.06	(6.97,7.15)	0.05	
	Other	7.10	(3.74,10.45)	0.67		7.19	(4.93,9.44)	0.45		7.10	(5.54,8.66)	0.31	
Marital status	Divorced	6.55	(6.33,6.78)	0.12	0.003	6.74	(6.52,6.96)	0.11	0.002	6.83	(6.61,7.05)	0.11	< .001
	Domestic partner	6.71	(6.55,6.86)	0.08		6.91	(6.71,7.10)	0.10		7.06	(6.88,7.23)	0.09	
	Married	6.87	(6.78,6.96)	0.05		7.06	(6.96,7.16)	0.05		7.17	(7.07,7.27)	0.05	
	Separated	6.55	(6.24,6.86)	0.16		6.71	(6.34,7.07)	0.18		6.67	(6.34,7.00)	0.17	
	Single/Never been married	6.68	(6.58,6.78)	0.05		6.77	(6.66,6.88)	0.06		6.78	(6.67,6.89)	0.06	
	Widowed	6.23	(5.74,6.73)	0.25		6.60	(6.09,7.11)	0.26		6.61	(6.17,7.04)	0.22	
Employment	Employed for an employer	6.84	(6.77,6.92)	0.04	< .001	6.98	(6.90,7.06)	0.04	0.001	7.11	(7.03,7.19)	0.04	< .001
	Homemaker	6.66	(6.38,6.95)	0.14		7.00	(6.72,7.27)	0.14		7.04	(6.75,7.32)	0.14	
	None of these/Other	6.49	(6.12,6.86)	0.19		6.98	(6.60,7.35)	0.19		6.94	(6.59,7.28)	0.17	
	Retired	6.81	(6.63,6.98)	0.09		6.96	(6.75,7.17)	0.11		6.99	(6.79,7.18)	0.10	
	Self-employed	6.80	(6.64,6.96)	0.08		6.84	(6.66,7.03)	0.10		6.91	(6.73,7.09)	0.09	
	Student	6.86	(6.65,7.07)	0.11		6.83	(6.59,7.07)	0.12		6.76	(6.53,6.99)	0.12	
	Unemployed and looking for a job	6.14	(5.96,6.31)	0.09		6.53	(6.35,6.71)	0.09		6.63	(6.46,6.80)	0.09	
Religious service attendance	A few times a year	6.71	(6.59,6.82)	0.06	< .001	6.86	(6.74,6.98)	0.06	< .001	6.96	(6.84,7.07)	0.06	< .001
	More than once a week	7.40	(7.12,7.68)	0.14		7.43	(7.12,7.73)	0.15		7.50	(7.17,7.84)	0.17	
	Never	6.57	(6.49,6.65)	0.04		6.75	(6.65,6.85)	0.05		6.79	(6.70,6.88)	0.05	
	Once a week	7.16	(6.99,7.33)	0.09		7.38	(7.20,7.55)	0.09		7.47	(7.28,7.65)	0.09	

		Cantril's Ladder				Life Satisfaction				Happiness			
Variable	Category	Mean	95% CI	SE	Global p-value	Mean	95% CI	SE	Global p-value	Mean	95% CI	SE	Global p-value
	One to three times a month	6.93	(6.73,7.13)	0.10		7.09	(6.87,7.32)	0.12		7.30	(7.08,7.52)	0.11	
Education	Up to 8 years	6.70	(6.46,6.94)	0.12	< .001	6.95	(6.67,7.22)	0.14	0.002	7.04	(6.78,7.30)	0.13	< .001
	16+ years	7.00	(6.89,7.10)	0.05		7.10	(6.98,7.23)	0.06		7.20	(7.08,7.31)	0.06	
	9 to 15 years	6.67	(6.60,6.74)	0.03		6.84	(6.76,6.92)	0.04		6.91	(6.83,6.98)	0.04	
Immigration status	Born in another country	7.04	(6.88,7.19)	0.08	< .001	7.34	(7.17,7.51)	0.09	< .001	7.41	(7.25,7.57)	0.08	< .001
	Born in this country	6.70	(6.64,6.76)	0.03		6.85	(6.78,6.92)	0.04		6.92	(6.86,6.99)	0.03	
Religious affiliation	Buddhism	6.48	(5.84,7.11)	0.30	< .001	6.46	(5.81,7.11)	0.31	< .001	6.67	(5.93,7.40)	0.35	< .001
	Christianity	6.80	(6.72,6.87)	0.04		6.98	(6.90,7.07)	0.04		7.07	(6.99,7.15)	0.04	
	Confucianism	5.45	*	*		5.91	*	*		5.45	*	*	
	Hinduism	6.41	*	*		5.11	*	*		6.90	*	*	
	Islam	7.27	(6.86,7.67)	0.20		6.88	(6.31,7.45)	0.29		7.00	(6.55,7.46)	0.23	
	Judaism	7.96	*	*		7.27	*	*		8.27	*	*	
	No religion/Atheist/Agnostic	6.60	(6.50,6.70)	0.05		6.77	(6.66,6.88)	0.06		6.81	(6.70,6.91)	0.05	
	Primal, Animist, or Folk religion	6.63	*	*		7.21	*	*		7.26	*	*	
	Sikhism	6.97	*	*		6.52	*	*		7.94	*	*	
	Some other religion	7.05	(5.89,8.21)	0.53		7.74	(6.90,8.58)	0.38		7.48	(6.21,8.75)	0.58	
	Taoism	6.07	*	*		6.51	*	*		6.51	*	*	
	Baha'i	6.60	*	*		7.20	*	*		7.20	*	*	
	Jainism	6.00	*	*		4.00	*	*		4.00	*	*	

Table S18f. Complete-case supplemental analysis of childhood predictors regression analysis results for Spain

		Cantril's Ladder				Life Satisfaction				Happiness			
Variable	Category	Est	95% CI	SE	Global p-value	Est	95% CI	SE	Global p-value	Est	95% CI	SE	Global p-value
Relationship with mother	(Ref: Very bad/somewhat bad)				<.001				0.005				<.001
	Very good/somewhat good	0.47	(0.22,0.72)	0.13		0.44	(0.13,0.75)	0.16		0.54	(0.27,0.82)	0.14	
Relationship with father	(Ref: Very bad/somewhat bad)				0.226				0.417				0.321
	Very good/somewhat good	0.12	(-0.07,0.32)	0.10		0.10	(-0.14,0.35)	0.13		0.11	(-0.10,0.32)	0.11	
Parent marital status	(Ref: Parents married)				0.456				0.086				0.249
	No, one or both of them had died	-0.31	(-0.71,0.09)	0.20		-0.66	(-1.17,-0.14)	0.26		-0.41	(-0.86,0.03)	0.23	
	No, they were never married	-0.18	(-0.51,0.14)	0.16		-0.05	(-0.39,0.28)	0.17		0.03	(-0.28,0.35)	0.16	
	Yes, married	-0.14	(-0.38,0.10)	0.12		-0.12	(-0.39,0.15)	0.14		-0.03	(-0.27,0.21)	0.12	
Subjective financial status of family growing up	(Ref: Got by)				<.001				0.018				0.478
	Found it difficult	-0.20	(-0.37,-0.02)	0.09		-0.13	(-0.33,0.06)	0.10		-0.08	(-0.26,0.10)	0.09	
	Found it very difficult	-0.38	(-0.88,0.12)	0.25		-0.09	(-0.66,0.48)	0.29		-0.15	(-0.66,0.35)	0.26	
	Lived comfortably	0.19	(0.06,0.33)	0.07		0.19	(0.04,0.33)	0.08		0.06	(-0.07,0.20)	0.07	
Abuse	(Ref: No)				0.021				<.001				<.001
	Yes	-0.21	(-0.38,-0.03)	0.09		-0.42	(-0.63,-0.20)	0.11		-0.35	(-0.53,-0.16)	0.09	
Outsider growing up	(Ref: No)				0.416				0.116				0.134
	Yes	-0.09	(-0.30,0.12)	0.11		-0.17	(-0.39,0.04)	0.11		-0.15	(-0.34,0.05)	0.10	

		Cantril's Ladder				Life Satisfaction				Happiness			
Variable	Category	Est	95% CI	SE	Global p-value	Est	95% CI	SE	Global p-value	Est	95% CI	SE	Global p-value
Self-rated health growing up	(Ref: Good)				<.001				<.001				<.001
	Excellent	0.43	(0.26,0.60)	0.09		0.52	(0.32,0.72)	0.10		0.73	(0.55,0.91)	0.09	
	Fair	-0.15	(-0.57,0.28)	0.22		-0.19	(-0.65,0.27)	0.24		0.05	(-0.37,0.47)	0.21	
	Poor	0.27	(-0.20,0.73)	0.24		0.23	(-0.41,0.87)	0.33		0.25	(-0.33,0.83)	0.30	
	Very good	0.26	(0.10,0.42)	0.08		0.32	(0.13,0.51)	0.10		0.34	(0.17,0.52)	0.09	
Immigration status	(Ref: Born in this country)				<.001				<.001				<.001
	Born in another country	0.40	(0.23,0.57)	0.09		0.58	(0.40,0.76)	0.09		0.57	(0.40,0.75)	0.09	
Age 12 religious service attendance	(Ref: Never)				0.002				0.040				0.008
	At least once a week	0.11	(-0.06,0.28)	0.09		0.14	(-0.05,0.34)	0.10		0.20	(0.01,0.39)	0.10	
	Less than once a month	0.02	(-0.16,0.20)	0.09		-0.04	(-0.24,0.17)	0.10		0.02	(-0.17,0.21)	0.10	
	One to three times a month	0.30	(0.12,0.47)	0.09		0.19	(-0.01,0.39)	0.10		0.26	(0.07,0.45)	0.10	
Year of birth	(Ref: 1998-2005; current age: 18-24)				0.210				0.587				0.155
	1993-1998; age 25-29	0.05	(-0.19,0.29)	0.12		0.19	(-0.10,0.48)	0.15		0.30	(0.05,0.56)	0.13	
	1983-1993; age 30-39	-0.15	(-0.35,0.05)	0.10		0.08	(-0.16,0.32)	0.12		0.10	(-0.12,0.32)	0.11	
	1973-1983; age 40-49	-0.18	(-0.38,0.02)	0.10		0.04	(-0.19,0.27)	0.12		0.11	(-0.11,0.32)	0.11	
	1963-1973; age 50-59	-0.16	(-0.37,0.05)	0.11		0.05	(-0.19,0.30)	0.12		0.14	(-0.09,0.36)	0.12	
	1953-1963; age 60-69	-0.10	(-0.35,0.16)	0.13		0.18	(-0.11,0.46)	0.14		0.17	(-0.10,0.44)	0.14	
	1943-1953; age 70-79	-0.02	(-0.38,0.34)	0.18		0.14	(-0.30,0.58)	0.22		0.17	(-0.24,0.58)	0.21	

		Cantril's Ladder				Life Satisfaction				Happiness			
Variable	Category	Est	95% CI	SE	Global p-value	Est	95% CI	SE	Global p-value	Est	95% CI	SE	Global p-value
	1943 or earlier; age 80+	0.32	(-0.40,1.04)	0.37		0.76	(-0.15,1.67)	0.46		0.84	(0.15,1.52)	0.35	
Gender	(Ref: Male)				0.576				0.175				0.287
	Male	0.06	(-0.06,0.17)	0.06		0.12	(-0.01,0.25)	0.07		0.10	(-0.02,0.22)	0.06	
	Other	0.24	(-0.75,1.23)	0.51		0.18	(-0.60,0.96)	0.40		-0.06	(-0.88,0.75)	0.42	
Religious affiliation	(Ref: No religion/Atheist/Agnostic)				0.102				0.678				0.041
	Christianity	0.10	(-0.07,0.27)	0.09		0.08	(-0.11,0.27)	0.10		0.22	(0.05,0.40)	0.09	
	Christianity	0.10	(-0.07,0.27)	0.09		0.08	(-0.11,0.27)	0.10		0.22	(0.05,0.40)	0.09	
	Collapsed affiliations with prevalence<3 %	0.44	(0.02,0.85)	0.21		0.14	(-0.38,0.66)	0.26		0.31	(-0.13,0.75)	0.23	
Race/ethnicity	(Ref: Plurality group)												

Table S18g. Complete-case supplemental analysis of sensitivity to unmeasured confounding of childhood predictors in Spain

		Cantril's Ladder		Life Satisfaction		Happiness	
Variable	Category	E-value for Estimate	E-value for 95% CI	E-value for Estimate	E-value for 95% CI	E-value for Estimate	E-value for 95% CI
Relationship with mother	(Ref: Very bad/somewhat bad)						
	Very good/somewhat good	1.85	1.49	1.74	1.32	1.92	1.53
Relationship with father	(Ref: Very bad/somewhat bad)						
	Very good/somewhat good	1.32	1.00	1.27	1.00	1.29	1.00
Parent marital status	(Ref: Parents married)						
	No, one or both of them had died	1.61	1.00	2.03	1.33	1.74	1.00
	No, they were never married	1.42	1.00	1.18	1.00	1.14	1.00
	Yes, married	1.35	1.00	1.31	1.00	1.14	1.00
Subjective financial status of family growing up	(Ref: Got by)						
	Found it difficult	1.44	1.11	1.32	1.00	1.24	1.00
	Found it very difficult	1.72	1.00	1.25	1.00	1.36	1.00
	Lived comfortably	1.44	1.21	1.40	1.15	1.21	1.00
Abuse	(Ref: No)						
	Yes	1.46	1.14	1.71	1.42	1.64	1.38
Outsider growing up	(Ref: No)						
	Yes	1.26	1.00	1.38	1.00	1.35	1.00
Self-rated health growing up	(Ref: Good)						
	Excellent	1.79	1.54	1.85	1.59	2.19	1.93
	Fair	1.36	1.00	1.41	1.00	1.19	1.00
	Poor	1.55	1.00	1.46	1.00	1.50	1.00
	Very good	1.54	1.29	1.59	1.32	1.64	1.39
Immigration status	(Ref: Born in this country)						
	Born in another country	1.76	1.50	1.93	1.69	1.96	1.72
Age 12 religious service attendance	(Ref: Never)						
	At least once a week	1.30	1.00	1.33	1.00	1.44	1.09
	Less than once a month	1.11	1.00	1.14	1.00	1.12	1.00
	One to three times a month	1.60	1.32	1.41	1.00	1.52	1.22
Year of birth	(Ref: 1998-2005; current age: 18-24)						

Variable	Category	Cantril's Ladder		Life Satisfaction		Happiness	
		E-value for Estimate	E-value for 95% CI	E-value for Estimate	E-value for 95% CI	E-value for Estimate	E-value for 95% CI
	1993-1998; age 25-29	1.18	1.00	1.40	1.00	1.58	1.18
	1983-1993; age 30-39	1.37	1.00	1.24	1.00	1.28	1.00
	1973-1983; age 40-49	1.42	1.00	1.16	1.00	1.29	1.00
	1963-1973; age 50-59	1.39	1.00	1.18	1.00	1.34	1.00
	1953-1963; age 60-69	1.28	1.00	1.39	1.00	1.39	1.00
	1943-1953; age 70-79	1.11	1.00	1.33	1.00	1.38	1.00
	1943 or earlier; age 80+	1.63	1.00	2.18	1.00	2.35	1.37
Gender	(Ref: Male)						
	Male	1.20	1.00	1.31	1.00	1.27	1.00
	Other	1.51	1.00	1.39	1.00	1.20	1.00
Religious affiliation	(Ref: No religion/Atheist/Agnostic)						
	Christianity	1.29	1.00	1.24	1.00	1.47	1.17
	Christianity	1.29	1.00	1.24	1.00	1.47	1.17
	Collapsed affiliations with prevalence<3%	1.80	1.13	1.33	1.00	1.59	1.00
Race/ethnicity	(Ref: Plurality group)						

Tables S19a-g: Sweden

Table S19a. Nationally representative descriptive statistics for Sweden

Characteristic	N = 15,068[1]
**Age group**	
1998-2005; age 18-24	1,515 (10%)
1993-1998; age 25-29	1,399 (9.3%)
1983-1993; age 30-39	2,398 (16%)
1973-1983; age 40-49	2,221 (15%)
1963-1973; age 50-59	2,493 (17%)
1953-1963; age 60-69	2,168 (14%)
1943-1953; age 70-79	2,253 (15%)
1943 or earlier; age 80+	621 (4.1%)
(Missing)	0 (0%)
**Gender**	
Male	7,536 (50%)
Female	7,493 (50%)
Other	27 (0.2%)
(Missing)	12 (<0.1%)
**Respondent Marital status**	
Married	6,408 (43%)
Separated	426 (2.8%)
Divorced	801 (5.3%)
Widowed	433 (2.9%)
Single, never married	3,854 (26%)
Domestic Partner	3,073 (20%)
(Missing)	72 (0.5%)
**Employment**	
Employed for an employer	7,907 (52%)
Self-employed	1,243 (8.3%)
Retired	3,832 (25%)
Student	1,332 (8.8%)
Homemaker	75 (0.5%)
Unemployed and looking for a job	324 (2.2%)
None of these/Other	337 (2.2%)
(Missing)	18 (0.1%)
**Religious service attendance as an adult (now)**	
More than 1/week	236 (1.6%)
1/week	434 (2.9%)
1-3/month	486 (3.2%)
A few times a year	3,950 (26%)
Never	9,918 (66%)
(Missing)	45 (0.3%)
**Education (years)**	
Up to 8 years	252 (1.7%)
9-15 years	10,790 (72%)
16+ years	4,026 (27%)
(Missing)	0 (0%)
**Immigration status**	
Born in this country	13,922 (92%)
Born in another country	1,052 (7.0%)
(Missing)	94 (0.6%)
**Religious affiliation as an adult (now)**	
Christianity	8,346 (55%)
Islam	470 (3.1%)
Hinduism	22 (0.1%)
Buddhism	110 (0.7%)
Judaism	54 (0.4%)
Sikhism	4 (<0.1%)
Baha'i	6 (<0.1%)
Jainism	0 (0%)
Shinto	0 (<0.1%)
Taoism	4 (<0.1%)
Confucianism	0 (0%)
Primal, Animist, or Folk religion	83 (0.5%)
Spiritism	0 (0%)

Characteristic	N = 15,068[1]
Umbanda, Candomble, and other African-derived religions	0 (0%)
Chinese folk/traditional religion	0 (0%)
Some other religion	198 (1.3%)
No religion/Atheist/Agnostic	5,697 (38%)
(Missing)	74 (0.5%)
**Relationship with mother growing up**	
Very good	8,743 (58%)
Somewhat good	4,513 (30%)
Somewhat bad	1,194 (7.9%)
Very bad	371 (2.5%)
Does not apply	216 (1.4%)
(Missing)	30 (0.2%)
**Relationship with father growing up**	
Very good	7,134 (47%)
Somewhat good	4,885 (32%)
Somewhat bad	1,588 (11%)
Very bad	725 (4.8%)
Does not apply	720 (4.8%)
(Missing)	16 (0.1%)
**Parent marital status at age 12**	
Parents married	10,887 (72%)
Divorced	1,927 (13%)
Parents were never married	1,747 (12%)
One or both parents had died	362 (2.4%)
(Missing)	145 (1.0%)
**Subjective financial status of family growing up**	
Lived comfortably	5,951 (39%)
Got by	7,717 (51%)
Found it difficult	1,238 (8.2%)
Found it very difficult	140 (0.9%)
(Missing)	22 (0.1%)
**Abuse**	
Yes	2,288 (15%)
No	12,735 (85%)
(Missing)	45 (0.3%)
**Outsider growing up**	
Yes	1,867 (12%)
No	13,034 (86%)
(Missing)	168 (1.1%)
**Self-rated health growing up**	
Excellent	5,733 (38%)
Very good	5,124 (34%)
Good	2,669 (18%)
Fair	1,108 (7.4%)
Poor	397 (2.6%)
(Missing)	38 (0.2%)
**Age 12 religious service attendance**	
At least 1/week	955 (6.3%)
1-3/month	1,362 (9.0%)
<1/month	6,224 (41%)
Never	6,472 (43%)
(Missing)	54 (0.4%)
**Religious affiliation at age 12**	
Christianity	10,617 (70%)
Islam	462 (3.1%)
Hinduism	16 (0.1%)
Buddhism	41 (0.3%)
Judaism	51 (0.3%)
Sikhism	9 (<0.1%)
Baha'i	3 (<0.1%)
Jainism	0 (0%)
Shinto	1 (<0.1%)
Taoism	0 (0%)
Confucianism	4 (<0.1%)
Primal, Animist, or Folk religion	31 (0.2%)

Characteristic	N = 15,068[1]
Spiritism	0 (0%)
Umbanda, Candomble, and other African-derived religions	0 (0%)
Chinese folk/traditional religion	0 (0%)
Some other religion	69 (0.5%)
No religion/Atheist/Agnostic	3,738 (25%)
(Missing)	26 (0.2%)

[1]n (%)

Table S19b. Means by demographic category for Sweden

Variable	Category	Cantril's Ladder				Life Satisfaction				Happiness			
		Mean	95% CI	SE	Global p-value	Mean	95% CI	SE	Global p-value	Mean	95% CI	SE	Global p-value
Age group	18-24	6.65	(6.54,6.75)	0.05	< .001	6.44	(6.31,6.57)	0.07	< .001	6.45	(6.33,6.57)	0.06	< .001
	25-29	6.73	(6.61,6.84)	0.06		6.57	(6.43,6.70)	0.07		6.52	(6.39,6.65)	0.07	
	30-39	6.86	(6.78,6.94)	0.04		6.71	(6.61,6.81)	0.05		6.70	(6.61,6.79)	0.05	
	40-49	6.95	(6.85,7.04)	0.05		6.74	(6.63,6.85)	0.05		6.75	(6.64,6.85)	0.05	
	50-59	7.19	(7.11,7.27)	0.04		7.08	(6.98,7.17)	0.05		7.05	(6.96,7.14)	0.05	
	60-69	7.59	(7.51,7.67)	0.04		7.54	(7.45,7.63)	0.05		7.42	(7.33,7.51)	0.05	
	70-79	7.88	(7.80,7.96)	0.04		7.93	(7.84,8.01)	0.04		7.75	(7.67,7.83)	0.04	
	80 or older	7.98	(7.83,8.14)	0.08		8.10	(7.94,8.26)	0.08		7.85	(7.69,8.00)	0.08	
Gender	Male	7.19	(7.15,7.24)	0.02	0.005	7.09	(7.04,7.15)	0.03	< .001	7.01	(6.96,7.06)	0.03	< .001
	Female	7.21	(7.16,7.26)	0.03		7.10	(7.04,7.16)	0.03		7.06	(7.00,7.11)	0.03	
	Other	5.67	(4.67,6.66)	0.48		4.79	(3.52,6.06)	0.61		5.15	(4.38,5.91)	0.37	
Marital status	Married	7.73	(7.68,7.78)	0.02	< .001	7.73	(7.68,7.78)	0.03	< .001	7.62	(7.57,7.67)	0.03	< .001
	Separated	6.58	(6.36,6.81)	0.11		6.39	(6.15,6.64)	0.13		6.43	(6.19,6.66)	0.12	
	Divorced	7.01	(6.88,7.14)	0.07		6.91	(6.76,7.07)	0.08		6.82	(6.68,6.97)	0.07	
	Widowed	7.54	(7.35,7.72)	0.09		7.39	(7.19,7.59)	0.10		7.11	(6.92,7.31)	0.10	
	Never	6.42	(6.35,6.49)	0.04		6.14	(6.06,6.23)	0.04		6.16	(6.08,6.24)	0.04	
	Domestic Partner	7.16	(7.09,7.22)	0.03		7.06	(6.99,7.14)	0.04		7.03	(6.96,7.11)	0.04	
Employment	Employed for an employer	7.11	(7.07,7.16)	0.02	< .001	6.97	(6.92,7.02)	0.03	< .001	6.93	(6.88,6.98)	0.03	< .001
	Self-employed	7.61	(7.49,7.74)	0.06		7.50	(7.35,7.64)	0.07		7.39	(7.24,7.53)	0.07	
	Retired	7.72	(7.66,7.79)	0.03		7.76	(7.69,7.83)	0.03		7.60	(7.54,7.67)	0.03	
	Student	6.67	(6.56,6.78)	0.06		6.52	(6.39,6.66)	0.07		6.44	(6.31,6.56)	0.06	
	Homemaker	6.88	(6.36,7.41)	0.26		7.07	(6.56,7.59)	0.26		6.89	(6.32,7.45)	0.28	
	Unemployed and looking for a job	5.26	(5.02,5.50)	0.12		4.83	(4.55,5.11)	0.14		5.19	(4.92,5.47)	0.14	
	None of these/Other	5.71	(5.42,5.99)	0.14		5.29	(4.96,5.62)	0.17		5.73	(5.42,6.03)	0.15	
Religious service attendance	More than 1/week	7.76	(7.44,8.08)	0.16	< .001	7.82	(7.48,8.16)	0.17	< .001	7.73	(7.37,8.10)	0.19	< .001
	1/week	7.25	(7.03,7.47)	0.11		7.21	(6.94,7.47)	0.13		7.17	(6.92,7.42)	0.13	
	1-3/month	7.18	(6.98,7.38)	0.10		7.17	(6.96,7.38)	0.11		7.09	(6.89,7.29)	0.10	
	A few times a year	7.46	(7.39,7.52)	0.03		7.39	(7.32,7.47)	0.04		7.30	(7.23,7.37)	0.04	
	Never	7.08	(7.04,7.12)	0.02		6.95	(6.90,7.00)	0.02		6.90	(6.85,6.95)	0.02	
Education	Up to 8 years	7.39	(7.10,7.69)	0.15	< .001	7.41	(7.08,7.73)	0.17	< .001	7.25	(6.94,7.56)	0.16	< .001
	9-15 years	7.13	(7.08,7.17)	0.02		7.03	(6.98,7.07)	0.02		6.98	(6.94,7.03)	0.02	

		Cantril's Ladder				Life Satisfaction				Happiness			
Variable	Category	Mean	95% CI	SE	Global p-value	Mean	95% CI	SE	Global p-value	Mean	95% CI	SE	Global p-value
	16+ years	7.38	(7.32,7.44)	0.03		7.25	(7.18,7.32)	0.04		7.15	(7.08,7.22)	0.04	
Immigration status	Born in this country	7.20	(7.17,7.24)	0.02	0.477	7.09	(7.05,7.13)	0.02	0.230	7.03	(6.99,7.07)	0.02	0.923
	Born in another country	7.15	(7.01,7.29)	0.07		7.18	(7.03,7.34)	0.08		7.04	(6.89,7.19)	0.08	
Religious affiliation	Christianity	7.45	(7.41,7.49)	0.02	< .001	7.37	(7.32,7.42)	0.03	< .001	7.29	(7.24,7.34)	0.03	< .001
	Islam	6.86	(6.61,7.12)	0.13		7.04	(6.77,7.31)	0.14		6.91	(6.62,7.21)	0.15	
	Hinduism	7.43	(5.61,9.26)	0.79		6.50	(4.49,8.51)	0.86		6.49	(4.39,8.60)	0.90	
	Buddhism	6.65	(6.21,7.10)	0.22		6.51	(5.96,7.06)	0.28		6.57	(6.15,7.00)	0.21	
	Judaism	7.25	(6.63,7.88)	0.31		7.12	(6.08,8.16)	0.51		6.86	(5.95,7.77)	0.45	
	Sikhism	7.66	*	*		8.11	*	*		7.55	*	*	
	Baha'i	5.42	*	*		5.80	*	*		7.02	*	*	
	Shinto	6.00	*	*		6.00	*	*		5.60	*	*	
	Taoism	7.40	*	*		4.40	*	*		7.00	*	*	
	Primal, Animist, or Folk religion	6.23	(5.65,6.81)	0.29		6.14	(5.43,6.85)	0.36		5.72	(5.11,6.33)	0.30	
	Some other religion	6.05	(5.57,6.53)	0.24		5.81	(5.26,6.36)	0.28		5.67	(5.16,6.17)	0.26	
	No religion/Atheist /Agnostic	6.92	(6.87,6.97)	0.03		6.76	(6.71,6.82)	0.03		6.73	(6.68,6.79)	0.03	

Table S19c. Childhood predictors regression analysis results for Sweden

Variable	Category	Cantril's Ladder				Life Satisfaction				Happiness			
		Est	95% CI	SE	Global p-value	Est	95% CI	SE	Global p-value	Est	95% CI	SE	Global p-value
Relationship with mother	(Ref: Very bad/somewhat bad)				0.676				0.835				0.256
	Very good/somewhat good	0.02	(-0.10, 0.15)	0.06		0.01	(-0.13, 0.15)	0.07		0.08	(-0.06, 0.22)	0.07	
Relationship with father	(Ref: Very bad/somewhat bad)				0.133				0.147				0.140
	Very good/somewhat good	0.08	(-0.02, 0.18)	0.05		0.09	(-0.03, 0.21)	0.06		0.09	(-0.03, 0.20)	0.06	
Parent marital status	(Ref: Parents married)				0.081				0.020				0.002
	Divorced	0.03	(-0.07, 0.14)	0.05		0.06	(-0.06, 0.19)	0.06		0.13	(0.01, 0.24)	0.06	
	Parents were never married	-0.12	(-0.23, -0.02)	0.05		-0.17	(-0.30, -0.04)	0.07		-0.16	(-0.28, -0.03)	0.06	
	One or both parents had died	-0.02	(-0.25, 0.21)	0.12		0.07	(-0.19, 0.34)	0.13		0.10	(-0.16, 0.35)	0.13	
Subjective financial status of family growing up	(Ref: Got by)				<.001				<.001				<.001
	Lived comfortably	0.33	(0.26, 0.39)	0.03		0.25	(0.17, 0.33)	0.04		0.27	(0.19, 0.34)	0.04	
	Found it difficult	-0.04	(-0.17, 0.08)	0.07		-0.07	(-0.22, 0.08)	0.08		-0.03	(-0.18, 0.11)	0.07	
	Found it very difficult	0.06	(-0.38, 0.49)	0.22		0.10	(-0.41, 0.60)	0.26		0.20	(-0.26, 0.66)	0.24	
Abuse	(Ref: No)				<.001				<.001				<.001
	Yes	-0.19	(-0.29, -0.09)	0.05		-0.21	(-0.33, -0.09)	0.06		-0.25	(-0.36, -0.13)	0.06	
Outsider growing up	(Ref: No)				<.001				<.001				<.001
	Yes	-0.30	(-0.42, -0.18)	0.06		-0.41	(-0.56, -0.27)	0.07		-0.46	(-0.59, -0.32)	0.07	
Self-rated health growing up	(Ref: Good)				<.001				<.001				<.001
	Excellent	0.93	(0.83, 1.03)	0.05		1.06	(0.95, 1.18)	0.06		1.07	(0.96, 1.18)	0.06	

		Cantril's Ladder				Life Satisfaction				Happiness			
Variable	Category	Est	95% CI	SE	Global p-value	Est	95% CI	SE	Global p-value	Est	95% CI	SE	Global p-value
	Very good	0.47	(0.38, 0.57)	0.05		0.54	(0.43, 0.66)	0.06		0.54	(0.43, 0.64)	0.05	
	Fair	-0.19	(-0.34, -0.04)	0.08		-0.22	(-0.40, -0.04)	0.09		-0.18	(-0.34, -0.01)	0.09	
	Poor	-0.43	(-0.70, -0.17)	0.14		-0.72	(-1.06, -0.39)	0.17		-0.55	(-0.87, -0.24)	0.16	
Immigration status	(Ref: Born in this country)				0.123				0.004				0.091
	Born in another country	0.11	(-0.03, 0.25)	0.07		0.23	(0.07, 0.38)	0.08		0.13	(-0.02, 0.28)	0.08	
Age 12 religious service attendance	(Ref: Never)				0.012				0.006				0.042
	At least 1/week	0.25	(0.09, 0.40)	0.08		0.28	(0.11, 0.45)	0.09		0.23	(0.06, 0.40)	0.09	
	1-3/month	0.08	(-0.04, 0.20)	0.06		0.14	(0.00, 0.27)	0.07		0.09	(-0.04, 0.21)	0.06	
	< 1/month	0.04	(-0.03, 0.11)	0.04		0.04	(-0.04, 0.13)	0.04		0.05	(-0.03, 0.13)	0.04	
Year of birth	(Ref: 1998-2005; current age: 18-24)				<.001				<.001				<.001
	1993-1998; age 25-29	0.16	(0.02, 0.30)	0.07		0.20	(0.03, 0.37)	0.09		0.14	(-0.02, 0.31)	0.08	
	1983-1993; age 30-39	0.29	(0.17, 0.42)	0.06		0.34	(0.19, 0.50)	0.08		0.31	(0.17, 0.45)	0.07	
	1973-1983; age 40-49	0.30	(0.17, 0.44)	0.07		0.29	(0.13, 0.45)	0.08		0.29	(0.14, 0.44)	0.08	
	1963-1973; age 50-59	0.46	(0.32, 0.59)	0.07		0.53	(0.37, 0.69)	0.08		0.49	(0.34, 0.64)	0.08	
	1953-1963; age 60-69	0.79	(0.65, 0.92)	0.07		0.91	(0.76, 1.07)	0.08		0.79	(0.64, 0.94)	0.08	
	1943-1953; age 70-79	1.02	(0.89, 1.16)	0.07		1.24	(1.08, 1.40)	0.08		1.06	(0.91, 1.21)	0.08	
	1943 or earlier; age 80+	1.15	(0.97, 1.34)	0.09		1.44	(1.23, 1.65)	0.11		1.19	(1.00, 1.38)	0.10	
Gender	(Ref: Male)				<.001				<.001				<.001
	Female	0.14	(0.08, 0.21)	0.03		0.17	(0.09, 0.24)	0.04		0.22	(0.15, 0.29)	0.04	
	Other	-0.56	(-1.45, 0.33)	0.45		-1.17	(-2.20, -0.14)	0.53		-0.80	(-1.44, -0.15)	0.33	
Religious affiliation	(Ref: No religion/Atheist /Agnostic)				<.001				0.001				<.001
	Islam	0.13	(-0.12, 0.39)	0.13		0.36	(0.07, 0.65)	0.15		0.30	(0.02, 0.59)	0.14	
	Christianity	0.21	(0.13, 0.29)	0.04		0.17	(0.08, 0.27)	0.05		0.19	(0.09, 0.28)	0.05	

Variable	Category	Cantril's Ladder				Life Satisfaction				Happiness			
		Est	95% CI	SE	Global p-value	Est	95% CI	SE	Global p-value	Est	95% CI	SE	Global p-value
Race/ethnicity	Collapsed affiliations with prevalence<3% (Ref: Plurality group)	-0.04	(-0.39, 0.31)	0.18		0.12	(-0.29, 0.53)	0.21		0.29	(-0.04, 0.61)	0.16	

Table S19d. Sensitivity to unmeasured confounding of childhood predictors in Sweden

		Cantril's Ladder		Life Satisfaction		Happiness	
Variable	Category	E-value for Estimate	E-value for 95% CI	E-value for Estimate	E-value for 95% CI	E-value for Estimate	E-value for 95% CI
Relationship with mother	(Ref: Very bad/somewhat bad)						
	Very good/somewhat good	1.13	1.00	1.09	1.00	1.23	1.00
Relationship with father	(Ref: Very bad/somewhat bad)						
	Very good/somewhat good	1.25	1.00	1.24	1.00	1.25	1.00
Parent marital status	(Ref: Parents married)						
	Divorced	1.15	1.00	1.20	1.00	1.31	1.06
	Parents were never married	1.33	1.10	1.37	1.15	1.36	1.14
	One or both parents had died	1.12	1.00	1.22	1.00	1.27	1.00
Subjective financial status of family growing up	(Ref: Got by)						
	Lived comfortably	1.65	1.55	1.48	1.37	1.52	1.41
	Found it difficult	1.17	1.00	1.20	1.00	1.14	1.00
	Found it very difficult	1.21	1.00	1.26	1.00	1.42	1.00
Abuse	(Ref: No)						
	Yes	1.44	1.26	1.42	1.25	1.49	1.32
Outsider growing up	(Ref: No)						
	Yes	1.61	1.42	1.69	1.51	1.77	1.59
Self-rated health growing up	(Ref: Good)						
	Excellent	2.62	2.45	2.57	2.40	2.67	2.50
	Very good	1.87	1.73	1.86	1.71	1.89	1.74
	Fair	1.44	1.17	1.43	1.15	1.39	1.07
	Poor	1.81	1.41	2.09	1.66	1.91	1.48
Immigration status	(Ref: Born in this country)						
	Born in another country	1.31	1.00	1.44	1.21	1.32	1.00
Age 12 religious service attendance	(Ref: Never)						
	At least 1/week	1.53	1.28	1.52	1.28	1.47	1.21
	1-3/month	1.26	1.00	1.32	1.03	1.25	1.00
	< 1/month	1.18	1.00	1.16	1.00	1.18	1.00
Year of birth	(Ref: 1998-2005; current age: 18-24)						
	1993-1998; age 25-29	1.39	1.12	1.41	1.13	1.34	1.00
	1983-1993; age 30-39	1.60	1.41	1.60	1.39	1.58	1.38
	1973-1983; age 40-49	1.62	1.41	1.53	1.31	1.55	1.33
	1963-1973; age 50-59	1.85	1.65	1.84	1.64	1.82	1.62

Variable	Category	Cantril's Ladder		Life Satisfaction		Happiness	
		E-value for Estimate	E-value for 95% CI	E-value for Estimate	E-value for 95% CI	E-value for Estimate	E-value for 95% CI
	1953-1963; age 60-69	2.37	2.16	2.35	2.14	2.24	2.03
	1943-1953; age 70-79	2.79	2.55	2.84	2.60	2.65	2.42
	1943 or earlier; age 80+	3.03	2.69	3.17	2.83	2.87	2.55
Gender	(Ref: Male)						
	Female	1.36	1.25	1.36	1.25	1.45	1.34
	Other	2.00	1.00	2.73	1.32	2.25	1.35
Religious affiliation	(Ref: No religion/Atheist/Agnostic)						
	Islam	1.35	1.00	1.62	1.21	1.57	1.11
	Christianity	1.48	1.35	1.37	1.23	1.40	1.26
	Collapsed affiliations with prevalence<3%	1.17	1.00	1.29	1.00	1.54	1.00
Race/ethnicity	(Ref: Plurality group)						

Table S19e. Complete-case supplemental analysis of means by demographic category for Sweden

		Cantril's Ladder				Life Satisfaction				Happiness			
Variable	Category	Mean	95% CI	SE	Global p-value	Mean	95% CI	SE	Global p-value	Mean	95% CI	SE	Global p-value
Age group	18-24	6.65	(6.55,6.76)	0.05	< .001	6.52	(6.40,6.65)	0.06	< .001	6.48	(6.36,6.60)	0.06	< .001
	25-29	6.75	(6.65,6.86)	0.05		6.68	(6.56,6.81)	0.06		6.59	(6.47,6.72)	0.06	
	30-39	6.87	(6.79,6.95)	0.04		6.78	(6.68,6.88)	0.05		6.73	(6.64,6.82)	0.05	
	40-49	6.97	(6.87,7.06)	0.05		6.78	(6.68,6.89)	0.05		6.79	(6.68,6.89)	0.05	
	50-59	7.20	(7.12,7.29)	0.04		7.14	(7.05,7.23)	0.05		7.08	(6.99,7.17)	0.05	
	60-69	7.60	(7.52,7.68)	0.04		7.59	(7.51,7.68)	0.04		7.44	(7.36,7.53)	0.04	
	70-79	7.88	(7.80,7.96)	0.04		7.94	(7.86,8.02)	0.04		7.76	(7.67,7.84)	0.04	
	80 or older	7.99	(7.84,8.14)	0.08		8.10	(7.94,8.26)	0.08		7.85	(7.70,8.00)	0.08	
Gender	Female	7.22	(7.17,7.27)	0.02	0.006	7.16	(7.11,7.22)	0.03	< .001	7.09	(7.04,7.14)	0.03	< .001
	Male	7.20	(7.15,7.24)	0.02		7.15	(7.09,7.20)	0.03		7.04	(6.99,7.09)	0.03	
	Other	5.71	(4.71,6.70)	0.48		5.56	(4.91,6.21)	0.31		5.15	(4.38,5.91)	0.37	
Marital status	Divorced	7.03	(6.90,7.16)	0.07	< .001	6.99	(6.84,7.14)	0.08	< .001	6.84	(6.70,6.99)	0.07	< .001
	Domestic partner	7.16	(7.10,7.23)	0.03		7.11	(7.04,7.18)	0.04		7.05	(6.98,7.12)	0.04	
	Married	7.73	(7.68,7.78)	0.02		7.75	(7.70,7.80)	0.03		7.62	(7.57,7.68)	0.03	
	Separated	6.63	(6.43,6.84)	0.10		6.53	(6.30,6.76)	0.12		6.51	(6.29,6.73)	0.11	
	Single/Never been married	6.44	(6.37,6.51)	0.04		6.26	(6.18,6.34)	0.04		6.23	(6.15,6.31)	0.04	
	Widowed	7.53	(7.35,7.71)	0.09		7.39	(7.19,7.58)	0.10		7.11	(6.91,7.30)	0.10	
Employment	Employed for an employer	7.12	(7.08,7.17)	0.02	< .001	7.02	(6.97,7.07)	0.03	< .001	6.96	(6.91,7.00)	0.03	< .001
	Homemaker	6.89	(6.38,7.40)	0.25		7.11	(6.62,7.60)	0.25		7.12	(6.66,7.59)	0.23	
	None of these/Other	5.80	(5.52,6.07)	0.14		5.63	(5.32,5.93)	0.16		5.90	(5.62,6.19)	0.14	
	Retired	7.73	(7.66,7.79)	0.03		7.79	(7.72,7.86)	0.03		7.61	(7.55,7.68)	0.03	
	Self-employed	7.61	(7.49,7.74)	0.06		7.53	(7.39,7.67)	0.07		7.41	(7.27,7.55)	0.07	
	Student	6.67	(6.56,6.78)	0.06		6.61	(6.48,6.74)	0.06		6.47	(6.35,6.59)	0.06	
	Unemployed and looking for a job	5.34	(5.10,5.57)	0.12		5.08	(4.80,5.35)	0.14		5.34	(5.08,5.60)	0.13	
Religious service attendance	A few times a year	7.47	(7.41,7.53)	0.03	< .001	7.43	(7.36,7.50)	0.04	< .001	7.32	(7.25,7.39)	0.04	< .001
	More than once a week	7.76	(7.44,8.08)	0.16		7.85	(7.52,8.19)	0.17		7.85	(7.53,8.18)	0.16	
	Never	7.09	(7.05,7.13)	0.02		7.02	(6.97,7.06)	0.02		6.93	(6.89,6.98)	0.02	
	Once a week	7.25	(7.03,7.47)	0.11		7.25	(6.99,7.51)	0.13		7.20	(6.95,7.45)	0.13	

		Cantril's Ladder				Life Satisfaction				Happiness			
Variable	Category	Mean	95% CI	SE	Global p-value	Mean	95% CI	SE	Global p-value	Mean	95% CI	SE	Global p-value
	One to three times a month	7.19	(6.99,7.39)	0.10		7.19	(6.98,7.40)	0.11		7.11	(6.91,7.30)	0.10	
Education	Up to 8 years	7.41	(7.12,7.70)	0.15	< .001	7.52	(7.21,7.83)	0.16	< .001	7.30	(7.00,7.60)	0.15	< .001
	16+ years	7.38	(7.32,7.44)	0.03		7.29	(7.22,7.36)	0.04		7.17	(7.10,7.24)	0.03	
	9 to 15 years	7.14	(7.10,7.18)	0.02		7.09	(7.05,7.14)	0.02		7.01	(6.97,7.06)	0.02	
Immigration status	Born in another country	7.16	(7.02,7.29)	0.07	0.443	7.24	(7.09,7.39)	0.08	0.212	7.07	(6.93,7.22)	0.07	0.861
	Born in this country	7.21	(7.18,7.25)	0.02		7.14	(7.11,7.18)	0.02		7.06	(7.02,7.10)	0.02	
Religious affiliation	Buddhism	6.65	(6.21,7.10)	0.22	< .001	6.49	(5.94,7.04)	0.28	< .001	6.58	(6.16,7.01)	0.21	< .001
	Christianity	7.46	(7.41,7.50)	0.02		7.40	(7.35,7.45)	0.03		7.31	(7.27,7.36)	0.02	
	Hinduism	7.42	(5.53,9.31)	0.81		6.50	(4.49,8.51)	0.86		7.29	(6.12,8.46)	0.50	
	Islam	6.90	(6.66,7.15)	0.12		7.09	(6.83,7.36)	0.14		7.01	(6.73,7.28)	0.14	
	Judaism	7.25	(6.63,7.88)	0.31		7.12	(6.08,8.16)	0.51		6.86	(5.95,7.77)	0.45	
	No religion/Atheist/Agnostic	6.93	(6.88,6.98)	0.02		6.85	(6.79,6.90)	0.03		6.77	(6.71,6.82)	0.03	
	Primal, Animist, or Folk religion	6.23	(5.65,6.81)	0.29		6.34	(5.74,6.94)	0.30		5.71	(5.10,6.32)	0.31	
	Sikhism	7.66	*	*		8.11	*	*		7.55	*	*	
	Some other religion	6.19	(5.74,6.64)	0.23		6.11	(5.59,6.63)	0.26		5.77	(5.28,6.26)	0.25	
	Taoism	7.40	*	*		4.40	*	*		7.00	*	*	
	Baha'i	5.42	*	*		5.80	*	*		7.02	*	*	
	Shinto	6.00	*	*		6.00	*	*		5.00	*	*	

Table S19c. Complete-case supplemental analysis of childhood predictors regression analysis results for Sweden

		Cantril's Ladder				Life Satisfaction				Happiness			
Variable	Category	Est	95% CI	SE	Global p-value	Est	95% CI	SE	Global p-value	Est	95% CI	SE	Global p-value
Relationship with mother	(Ref: Very bad/somewhat bad)				0.813				0.987				0.331
	Very good/somewhat good	0.01	(-0.11,0.13)	0.06		-0.00	(-0.14,0.13)	0.07		0.07	(-0.07,0.20)	0.07	
Relationship with father	(Ref: Very bad/somewhat bad)				0.161				0.182				0.166
	Very good/somewhat good	0.07	(-0.03,0.17)	0.05		0.08	(-0.04,0.20)	0.06		0.08	(-0.03,0.19)	0.06	
Parent marital status	(Ref: Parents married)				0.073				0.021				0.009
	No, one or both of them had died	-0.05	(-0.28,0.17)	0.11		-0.05	(-0.33,0.22)	0.14		-0.02	(-0.27,0.23)	0.13	
	No, they were never married	-0.16	(-0.29,-0.03)	0.06		-0.23	(-0.37,-0.08)	0.08		-0.24	(-0.38,-0.10)	0.07	
	Yes, married	-0.04	(-0.14,0.06)	0.05		-0.06	(-0.17,0.06)	0.06		-0.10	(-0.21,0.02)	0.06	
Subjective financial status of family growing up	(Ref: Got by)				<.001				<.001				<.001
	Found it difficult	-0.06	(-0.19,0.06)	0.06		-0.05	(-0.19,0.09)	0.07		-0.04	(-0.18,0.10)	0.07	
	Found it very difficult	0.14	(-0.27,0.55)	0.21		0.12	(-0.37,0.60)	0.25		0.21	(-0.24,0.66)	0.23	
	Lived comfortably	0.32	(0.25,0.39)	0.03		0.26	(0.19,0.34)	0.04		0.26	(0.18,0.33)	0.04	
Abuse	(Ref: No)				<.001				<.001				<.001
	Yes	-0.18	(-0.29,-0.08)	0.05		-0.19	(-0.31,-0.08)	0.06		-0.24	(-0.35,-0.13)	0.06	
Outsider growing up	(Ref: No)				<.001				<.001				<.001
	Yes	-0.29	(-0.41,-0.17)	0.06		-0.36	(-0.50,-0.22)	0.07		-0.43	(-0.56,-0.30)	0.07	

		Cantril's Ladder				Life Satisfaction				Happiness			
Variable	Category	Est	95% CI	SE	Global p-value	Est	95% CI	SE	Global p-value	Est	95% CI	SE	Global p-value
Self-rated health growing up	(Ref: Good)				<.001				<.001				<.001
	Excellent	0.92	(0.83,1.02)	0.05		1.03	(0.92,1.14)	0.06		1.07	(0.96,1.18)	0.05	
	Fair	-0.19	(-0.34,-0.05)	0.07		-0.17	(-0.35,-0.00)	0.09		-0.17	(-0.34,-0.01)	0.08	
	Poor	-0.40	(-0.65,-0.14)	0.13		-0.51	(-0.83,-0.19)	0.16		-0.41	(-0.71,-0.11)	0.15	
	Very good	0.46	(0.37,0.55)	0.05		0.51	(0.40,0.62)	0.06		0.54	(0.44,0.64)	0.05	
Immigration status	(Ref: Born in this country)				0.183				0.008				0.086
	Born in another country	0.09	(-0.04,0.23)	0.07		0.21	(0.05,0.36)	0.08		0.13	(-0.02,0.27)	0.07	
Age 12 religious service attendance	(Ref: Never)				0.009				0.005				0.023
	At least once a week	0.25	(0.09,0.40)	0.08		0.27	(0.10,0.44)	0.09		0.24	(0.08,0.41)	0.08	
	Less than once a month	0.05	(-0.02,0.12)	0.03		0.03	(-0.04,0.11)	0.04		0.04	(-0.03,0.12)	0.04	
	One to three times a month	0.10	(-0.02,0.22)	0.06		0.14	(0.01,0.26)	0.06		0.09	(-0.04,0.21)	0.06	
Year of birth	(Ref: 1998-2005; current age: 18-24)				<.001				<.001				<.001
	1993-1998; age 25-29	0.18	(0.04,0.32)	0.07		0.24	(0.07,0.40)	0.08		0.19	(0.04,0.35)	0.08	
	1983-1993; age 30-39	0.29	(0.17,0.41)	0.06		0.33	(0.19,0.48)	0.08		0.31	(0.17,0.45)	0.07	
	1973-1983; age 40-49	0.32	(0.19,0.45)	0.07		0.28	(0.12,0.43)	0.08		0.31	(0.16,0.45)	0.08	
	1963-1973; age 50-59	0.46	(0.33,0.60)	0.07		0.54	(0.38,0.69)	0.08		0.50	(0.35,0.64)	0.07	
	1953-1963; age 60-69	0.79	(0.66,0.92)	0.07		0.92	(0.77,1.07)	0.08		0.79	(0.65,0.94)	0.07	
	1943-1953; age 70-79	1.02	(0.89,1.16)	0.07		1.22	(1.06,1.37)	0.08		1.04	(0.89,1.19)	0.08	

		Cantril’s Ladder				Life Satisfaction				Happiness			
Variable	Category	Est	95% CI	SE	Global p-value	Est	95% CI	SE	Global p-value	Est	95% CI	SE	Global p-value
	1943 or earlier; age 80+	1.16	(0.98,1.34)	0.09		1.41	(1.20,1.61)	0.10		1.17	(0.98,1.36)	0.10	
Gender	(Ref: Male)				<.001				<.001				<.001
	Male	-0.15	(-0.21,-0.08)	0.03		-0.16	(-0.23,-0.08)	0.04		-0.21	(-0.28,-0.14)	0.04	
	Other	-0.69	(-1.59,0.20)	0.46		-0.81	(-1.43,-0.19)	0.32		-1.07	(-1.72,-0.42)	0.33	
Religious affiliation	(Ref: No religion/Atheist/Agnostic)				<.001				0.001				<.001
	Christianity	0.21	(0.13,0.29)	0.04		0.17	(0.07,0.26)	0.05		0.18	(0.09,0.27)	0.05	
	Christianity	0.21	(0.13,0.29)	0.04		0.17	(0.07,0.26)	0.05		0.18	(0.09,0.27)	0.05	
	Islam	0.16	(-0.08,0.39)	0.12		0.36	(0.08,0.64)	0.14		0.32	(0.05,0.59)	0.14	
	Collapsed affiliations with prevalence<3%	0.02	(-0.31,0.35)	0.17		0.23	(-0.14,0.59)	0.19		0.25	(-0.07,0.58)	0.16	
Race/ethnicity	(Ref: Plurality group)												

Table S19g. Complete-case supplemental analysis of sensitivity to unmeasured confounding of childhood predictors in Sweden

		Cantril's Ladder		Life Satisfaction		Happiness	
Variable	Category	E-value for Estimate	E-value for 95% CI	E-value for Estimate	E-value for 95% CI	E-value for Estimate	E-value for 95% CI
Relationship with mother	(Ref: Very bad/somewhat bad)						
	Very good/somewhat good	1.09	1.00	1.02	1.00	1.21	1.00
Relationship with father	(Ref: Very bad/somewhat bad)						
	Very good/somewhat good	1.24	1.00	1.23	1.00	1.24	1.00
Parent marital status	(Ref: Parents married)						
	No, one or both of them had died	1.20	1.00	1.19	1.00	1.10	1.00
	No, they were never married	1.40	1.15	1.46	1.23	1.49	1.27
	Yes, married	1.16	1.00	1.19	1.00	1.27	1.00
Subjective financial status of family growing up	(Ref: Got by)						
	Found it difficult	1.22	1.00	1.18	1.00	1.16	1.00
	Found it very difficult	1.36	1.00	1.30	1.00	1.45	1.00
	Lived comfortably	1.65	1.55	1.51	1.40	1.51	1.40
Abuse	(Ref: No)						
	Yes	1.43	1.25	1.41	1.24	1.49	1.32
Outsider growing up	(Ref: No)						
	Yes	1.60	1.41	1.64	1.46	1.75	1.57
Self-rated health growing up	(Ref: Good)						
	Excellent	2.62	2.46	2.59	2.42	2.72	2.54
	Fair	1.45	1.19	1.38	1.03	1.39	1.07
	Poor	1.77	1.37	1.84	1.41	1.73	1.30
	Very good	1.86	1.72	1.85	1.70	1.91	1.76
Immigration status	(Ref: Born in this country)						
	Born in another country	1.28	1.00	1.43	1.19	1.32	1.00
Age 12 religious service attendance	(Ref: Never)						
	At least once a week	1.53	1.28	1.52	1.28	1.50	1.25
	Less than once a month	1.19	1.00	1.14	1.00	1.17	1.00
	One to three times a month	1.29	1.00	1.33	1.08	1.25	1.00
Year of birth	(Ref: 1998-2005; current age: 18-24)						

Variable	Category	Cantril's Ladder		Life Satisfaction		Happiness	
		E-value for Estimate	E-value for 95% CI	E-value for Estimate	E-value for 95% CI	E-value for Estimate	E-value for 95% CI
	1993-1998; age 25-29	1.43	1.17	1.47	1.22	1.42	1.15
	1983-1993; age 30-39	1.60	1.41	1.61	1.40	1.59	1.39
	1973-1983; age 40-49	1.64	1.44	1.53	1.31	1.58	1.37
	1963-1973; age 50-59	1.87	1.66	1.88	1.68	1.85	1.65
	1953-1963; age 60-69	2.40	2.18	2.43	2.20	2.27	2.06
	1943-1953; age 70-79	2.81	2.57	2.90	2.65	2.67	2.43
	1943 or earlier; age 80+	3.07	2.72	3.23	2.88	2.89	2.57
Gender	(Ref: Male)						
	Male	1.37	1.26	1.36	1.24	1.44	1.34
	Other	2.23	1.00	2.26	1.41	2.72	1.74
Religious affiliation	(Ref: No religion/Atheist/Agnostic)						
	Christianity	1.47	1.34	1.37	1.22	1.40	1.26
	Christianity	1.47	1.34	1.37	1.22	1.40	1.26
	Islam	1.39	1.00	1.64	1.24	1.60	1.18
	Collapsed affiliations with prevalence<3%	1.11	1.00	1.46	1.00	1.51	1.00
Race/ethnicity	(Ref: Plurality group)						

Tables S20a-g: Tanzania

Table S20a. Nationally representative descriptive statistics for Tanzania

Characteristic	N = 9,075[1]
**Age group**	
1998-2005; age 18-24	2,284 (25%)
1993-1998; age 25-29	1,349 (15%)
1983-1993; age 30-39	2,060 (23%)
1973-1983; age 40-49	1,503 (17%)
1963-1973; age 50-59	912 (10%)
1953-1963; age 60-69	575 (6.3%)
1943-1953; age 70-79	297 (3.3%)
1943 or earlier; age 80+	93 (1.0%)
(Missing)	2 (<0.1%)
**Gender**	
Male	4,299 (47%)
Female	4,776 (53%)
Other	0 (0%)
(Missing)	0 (0%)
**Race/Ethnicity**	
African	9,060 (100%)
Arab	11 (0.1%)
Indian	3 (<0.1%)
(Missing)	2 (<0.1%)
**Respondent Marital status**	
Married	5,577 (61%)
Separated	404 (4.5%)
Divorced	103 (1.1%)
Widowed	450 (5.0%)
Single, never married	2,260 (25%)
Domestic Partner	275 (3.0%)
(Missing)	7 (<0.1%)
**Employment**	
Employed for an employer	513 (5.6%)
Self-employed	4,625 (51%)
Retired	139 (1.5%)
Student	319 (3.5%)
Homemaker	1,796 (20%)
Unemployed and looking for a job	1,491 (16%)
None of these/Other	186 (2.1%)
(Missing)	6 (<0.1%)
**Religious service attendance as an adult (now)**	
More than 1/week	2,622 (29%)
1/week	4,268 (47%)
1-3/month	1,082 (12%)
A few times a year	814 (9.0%)
Never	288 (3.2%)
(Missing)	1 (<0.1%)
**Education (years)**	
Up to 8 years	6,699 (74%)
9-15 years	2,252 (25%)
16+ years	122 (1.3%)
(Missing)	2 (<0.1%)
**Immigration status**	
Born in this country	9,048 (100%)
Born in another country	25 (0.3%)
(Missing)	1 (<0.1%)
**Religious affiliation as an adult (now)**	
Christianity	5,647 (62%)
Islam	3,189 (35%)
Hinduism	0 (0%)
Buddhism	0 (0%)
Judaism	0 (0%)
Sikhism	0 (0%)
Baha'i	0 (0%)

Characteristic	N = 9,075[1]
Jainism	0 (0%)
Shinto	0 (0%)
Taoism	1 (<0.1%)
Confucianism	0 (0%)
Primal, Animist, or Folk religion	12 (0.1%)
Spiritism	0 (0%)
Umbanda, Candomble, and other African-derived religions	0 (0%)
Chinese folk/traditional religion	0 (0%)
Some other religion	0 (0%)
No religion/Atheist/Agnostic	216 (2.4%)
(Missing)	10 (0.1%)
**Relationship with mother growing up**	
Very good	7,739 (85%)
Somewhat good	796 (8.8%)
Somewhat bad	84 (0.9%)
Very bad	84 (0.9%)
Does not apply	303 (3.3%)
(Missing)	70 (0.8%)
**Relationship with father growing up**	
Very good	6,831 (75%)
Somewhat good	1,101 (12%)
Somewhat bad	203 (2.2%)
Very bad	247 (2.7%)
Does not apply	550 (6.1%)
(Missing)	142 (1.6%)
**Parent marital status at age 12**	
Parents married	6,929 (76%)
Divorced	678 (7.5%)
Parents were never married	751 (8.3%)
One or both parents had died	313 (3.4%)
(Missing)	404 (4.4%)
**Subjective financial status of family growing up**	
Lived comfortably	2,611 (29%)
Got by	2,909 (32%)
Found it difficult	2,679 (30%)
Found it very difficult	814 (9.0%)
(Missing)	61 (0.7%)
**Abuse**	
Yes	716 (7.9%)
No	8,328 (92%)
(Missing)	32 (0.3%)
**Outsider growing up**	
Yes	734 (8.1%)
No	8,320 (92%)
(Missing)	22 (0.2%)
**Self-rated health growing up**	
Excellent	2,406 (27%)
Very good	2,036 (22%)
Good	2,946 (32%)
Fair	1,177 (13%)
Poor	456 (5.0%)
(Missing)	54 (0.6%)
**Age 12 religious service attendance**	
At least 1/week	5,580 (61%)
1-3/month	2,383 (26%)
<1/month	333 (3.7%)
Never	595 (6.6%)
(Missing)	184 (2.0%)
**Religious affiliation at age 12**	
Christianity	5,651 (62%)
Islam	3,060 (34%)
Hinduism	0 (0%)
Buddhism	0 (0%)
Judaism	0 (0%)
Sikhism	0 (0%)

**Characteristic**	**N = 9,075**[1]
Baha'i	1 (<0.1%)
Jainism	0 (0%)
Shinto	0 (0%)
Taoism	0 (0%)
Confucianism	0 (0%)
Primal, Animist, or Folk religion	11 (0.1%)
Spiritism	0 (0%)
Umbanda, Candomble, and other African-derived religions	0 (0%)
Chinese folk/traditional religion	0 (0%)
Some other religion	0 (0%)
No religion/Atheist/Agnostic	345 (3.8%)
(Missing)	7 (<0.1%)

[1]n (%)

Table S20b. Means by demographic category for Tanzania

Variable	Category	Cantril's Ladder				Life Satisfaction				Happiness			
		Mean	95% CI	SE	Global p-value	Mean	95% CI	SE	Global p-value	Mean	95% CI	SE	Global p-value
Age group	18-24	4.97	(4.75,5.18)	0.11	< .001	6.13	(5.88,6.38)	0.13	< .001	7.28	(7.11,7.44)	0.09	< .001
	25-29	4.73	(4.47,4.99)	0.13		5.47	(5.16,5.77)	0.16		6.82	(6.56,7.08)	0.13	
	30-39	4.31	(4.10,4.51)	0.10		5.04	(4.81,5.27)	0.12		6.56	(6.38,6.74)	0.09	
	40-49	4.13	(3.89,4.37)	0.12		4.81	(4.56,5.07)	0.13		6.14	(5.91,6.37)	0.12	
	50-59	3.94	(3.66,4.21)	0.14		4.69	(4.41,4.97)	0.14		5.84	(5.60,6.09)	0.13	
	60-69	3.81	(3.39,4.24)	0.22		5.25	(4.80,5.70)	0.23		6.11	(5.67,6.55)	0.23	
	70-79	3.27	(2.60,3.94)	0.34		5.32	(4.60,6.04)	0.37		5.67	(4.93,6.41)	0.37	
	80 or older	4.00	(2.57,5.43)	0.72		5.46	(4.37,6.55)	0.55		6.34	(5.33,7.36)	0.51	
Gender	Male	4.28	(4.10,4.47)	0.09	0.009	4.92	(4.73,5.12)	0.10	< .001	6.49	(6.34,6.64)	0.08	0.058
	Female	4.51	(4.33,4.69)	0.09		5.70	(5.52,5.89)	0.09		6.66	(6.51,6.80)	0.07	
Marital status	Married	4.34	(4.15,4.53)	0.10	< .001	5.34	(5.15,5.53)	0.10	< .001	6.50	(6.35,6.66)	0.08	< .001
	Separated	3.90	(3.52,4.29)	0.20		4.16	(3.72,4.61)	0.23		5.69	(5.31,6.08)	0.19	
	Divorced	3.41	(2.57,4.24)	0.42		4.35	(3.49,5.21)	0.43		6.02	(5.29,6.75)	0.37	
	Widowed	3.77	(3.28,4.27)	0.25		5.22	(4.67,5.77)	0.28		5.91	(5.41,6.41)	0.25	
	Never	4.77	(4.58,4.97)	0.10		5.62	(5.38,5.86)	0.12		7.08	(6.91,7.25)	0.09	
	Domestic Partner	4.69	(4.20,5.19)	0.25		5.04	(4.54,5.53)	0.25		6.56	(6.10,7.01)	0.23	
Employment	Employed for an employer	4.76	(4.46,5.06)	0.15	< .001	5.85	(5.44,6.26)	0.21	< .001	6.98	(6.66,7.30)	0.16	< .001
	Self-employed	4.36	(4.15,4.56)	0.11		5.16	(4.96,5.35)	0.10		6.55	(6.40,6.71)	0.08	
	Retired	3.53	(2.88,4.17)	0.33		5.45	(4.73,6.17)	0.36		5.87	(5.20,6.55)	0.34	
	Student	5.37	(5.03,5.71)	0.17		6.37	(5.96,6.78)	0.21		7.71	(7.38,8.04)	0.17	
	Homemaker	4.34	(4.10,4.57)	0.12		5.80	(5.53,6.07)	0.14		6.49	(6.26,6.71)	0.12	
	Unemployed and looking for a job	4.49	(4.24,4.73)	0.13		5.10	(4.78,5.41)	0.16		6.53	(6.29,6.77)	0.12	
	None of these/Other	3.40	(2.50,4.30)	0.46		3.87	(3.01,4.72)	0.43		5.94	(5.03,6.84)	0.46	
Religious service attendance	More than 1/week	4.35	(4.16,4.53)	0.10	0.620	5.38	(5.16,5.59)	0.11	0.618	6.51	(6.33,6.69)	0.09	0.018
	1/week	4.40	(4.19,4.60)	0.11		5.36	(5.14,5.58)	0.11		6.73	(6.57,6.90)	0.09	
	1-3/month	4.60	(4.30,4.90)	0.15		5.32	(5.00,5.64)	0.16		6.48	(6.24,6.72)	0.12	
	A few times a year	4.33	(4.00,4.65)	0.17		5.08	(4.75,5.42)	0.17		6.20	(5.89,6.51)	0.16	
	Never	4.41	(3.80,5.01)	0.31		5.32	(4.68,5.95)	0.32		6.31	(5.76,6.86)	0.28	
Education	Up to 8 years	4.21	(4.01,4.40)	0.10	< .001	5.19	(5.00,5.39)	0.10	< .001	6.40	(6.26,6.55)	0.07	< .001
	9-15 years	4.94	(4.78,5.11)	0.08		5.72	(5.54,5.90)	0.09		7.07	(6.93,7.20)	0.07	
	16+ years	5.16	(4.78,5.54)	0.19		5.88	(5.28,6.48)	0.30		7.18	(6.69,7.67)	0.25	

		Cantril's Ladder				Life Satisfaction				Happiness			
Variable	Category	Mean	95% CI	SE	Global p-value	Mean	95% CI	SE	Global p-value	Mean	95% CI	SE	Global p-value
Immigration status	Born in this country	4.40	(4.24,4.56)	0.08	0.525	5.33	(5.17,5.50)	0.08	0.648	6.58	(6.46,6.70)	0.06	0.851
	Born in another country	3.92	(2.34,5.51)	0.76		5.87	(3.39,8.35)	1.19		6.72	(4.90,8.55)	0.88	
Religious affiliation	Christianity	4.57	(4.37,4.77)	0.10	< .001	5.41	(5.22,5.61)	0.10	< .001	6.74	(6.59,6.88)	0.07	< .001
	Islam	4.15	(3.92,4.38)	0.12		5.21	(4.95,5.47)	0.13		6.35	(6.16,6.54)	0.10	
	Taoism	3.00	*	*		0.00	*	*		5.00	*	*	
	Primal, Animist, or Folk religion	3.76	(0.50,7.02)	1.27		5.55	(1.67,9.42)	1.51		7.02	(4.94,9.09)	0.81	
	No religion/Atheist /Agnostic	3.71	(3.07,4.34)	0.32		4.97	(4.29,5.65)	0.34		5.75	(5.16,6.33)	0.30	
Race/Ethnicity	Indian	3.11	*	*	0.152	0.00	*	*	< .001	4.85	*	*	< .001
	Arab	5.96	(3.70,8.23)	0.91		6.28	(5.70,6.86)	0.23		7.25	(6.18,8.31)	0.43	
	African	4.40	(4.24,4.56)	0.08		5.33	(5.17,5.50)	0.08		6.58	(6.46,6.70)	0.06	

Table S20c. Childhood predictors regression analysis results for Tanzania

		Cantril's Ladder				Life Satisfaction				Happiness			
Variable	Category	Est	95% CI	SE	Global p-value	Est	95% CI	SE	Global p-value	Est	95% CI	SE	Global p-value
Relationship with mother	(Ref: Very bad/somewhat bad)				0.488				0.806				0.250
	Very good/somewhat good	-0.17	(-0.64, 0.31)	0.24		-0.07	(-0.62, 0.49)	0.28		0.28	(-0.21, 0.78)	0.25	
Relationship with father	(Ref: Very bad/somewhat bad)				0.040				0.739				0.304
	Very good/somewhat good	0.38	(0.02, 0.75)	0.19		0.06	(-0.35, 0.48)	0.21		-0.19	(-0.55, 0.18)	0.19	
Parent marital status	(Ref: Parents married)				0.664				0.017				0.480
	Divorced	0.19	(-0.18, 0.57)	0.19		-0.29	(-0.67, 0.09)	0.20		-0.23	(-0.55, 0.09)	0.16	
	Parents were never married	0.09	(-0.31, 0.49)	0.21		-0.11	(-0.51, 0.29)	0.20		0.05	(-0.26, 0.36)	0.16	
	One or both parents had died	-0.15	(-0.64, 0.34)	0.25		-0.79	(-1.37, -0.22)	0.29		0.01	(-0.44, 0.45)	0.23	
Subjective financial status of family growing up	(Ref: Got by)				<.001				<.001				<.001
	Lived comfortably	0.20	(-0.02, 0.42)	0.11		0.12	(-0.12, 0.35)	0.12		0.27	(0.06, 0.49)	0.11	
	Found it difficult	-0.28	(-0.51, -0.05)	0.12		-0.52	(-0.80, -0.24)	0.14		-0.32	(-0.50, -0.13)	0.10	
	Found it very difficult	-0.79	(-1.19, -0.40)	0.20		-1.02	(-1.44, -0.60)	0.22		-0.66	(-1.06, -0.26)	0.21	
Abuse	(Ref: No)				0.079				<.001				0.001
	Yes	-0.27	(-0.57, 0.03)	0.15		-0.63	(-0.99, -0.27)	0.18		-0.53	(-0.86, -0.21)	0.17	
Outsider growing up	(Ref: No)				0.365				0.455				0.268
	Yes	0.16	(-0.19, 0.52)	0.18		-0.15	(-0.56, 0.25)	0.21		-0.20	(-0.56, 0.16)	0.18	
Self-rated health growing up	(Ref: Good)				0.002				0.213				0.185
	Excellent	0.14	(-0.11, 0.39)	0.13		0.21	(-0.06, 0.47)	0.14		0.03	(-0.18, 0.25)	0.11	

		Cantril's Ladder				Life Satisfaction				Happiness			
Variable	Category	Est	95% CI	SE	Global p-value	Est	95% CI	SE	Global p-value	Est	95% CI	SE	Global p-value
	Very good	0.33	(0.07, 0.59)	0.13		0.16	(-0.13, 0.46)	0.15		0.16	(-0.09, 0.40)	0.13	
	Fair	-0.02	(-0.32, 0.27)	0.15		-0.03	(-0.36, 0.29)	0.17		-0.01	(-0.26, 0.23)	0.13	
	Poor	-0.56	(-1.00, -0.12)	0.23		-0.36	(-0.87, 0.16)	0.26		-0.41	(-0.82, -0.00)	0.21	
Immigration status	(Ref: Born in this country)				0.535				0.627				0.820
	Born in another country	-0.53	(-2.20, 1.14)	0.85		0.58	(-1.74, 2.89)	1.18		0.21	(-1.61, 2.03)	0.93	
Age 12 religious service attendance	(Ref: Never)				0.926				0.024				0.163
	At least 1/week	0.07	(-0.42, 0.57)	0.25		-0.81	(-1.34, -0.27)	0.27		-0.23	(-0.69, 0.23)	0.23	
	1-3/month	0.09	(-0.42, 0.60)	0.26		-0.77	(-1.31, -0.22)	0.28		-0.43	(-0.91, 0.06)	0.25	
	< 1/month	-0.06	(-0.65, 0.53)	0.30		-0.69	(-1.30, -0.09)	0.31		-0.28	(-0.93, 0.37)	0.33	
Year of birth	(Ref: 1998-2005; current age: 18-24)				<.001				<.001				<.001
	1993-1998; age 25-29	-0.21	(-0.49, 0.06)	0.14		-0.61	(-0.96, -0.26)	0.18		-0.42	(-0.70, -0.14)	0.14	
	1983-1993; age 30-39	-0.58	(-0.82, -0.35)	0.12		-0.96	(-1.23, -0.68)	0.14		-0.62	(-0.85, -0.40)	0.11	
	1973-1983; age 40-49	-0.71	(-0.97, -0.45)	0.13		-1.15	(-1.46, -0.84)	0.16		-1.01	(-1.27, -0.75)	0.13	
	1963-1973; age 50-59	-0.90	(-1.19, -0.60)	0.15		-1.30	(-1.62, -0.98)	0.17		-1.35	(-1.63, -1.07)	0.14	
	1953-1963; age 60-69	-1.14	(-1.57, -0.71)	0.22		-0.89	(-1.38, -0.40)	0.25		-1.18	(-1.65, -0.71)	0.24	
	1943-1953; age 70-79	-1.70	(-2.38, -1.02)	0.35		-0.82	(-1.52, -0.12)	0.36		-1.59	(-2.33, -0.86)	0.38	
	1943 or earlier; age 80+	-1.00	(-2.46, 0.46)	0.75		-0.67	(-1.79, 0.45)	0.57		-1.00	(-2.09, 0.09)	0.55	
Gender	(Ref: Male)				0.030				<.001				0.178
	Female	0.19	(0.02, 0.37)	0.09		0.73	(0.55, 0.91)	0.09		0.12	(-0.05, 0.29)	0.09	
Religious affiliation	(Ref: No religion/Atheist /Agnostic)				0.140				0.034				0.012
	Islam	-0.11	(-0.77, 0.56)	0.34		0.76	(0.04, 1.48)	0.37		0.45	(-0.26, 1.15)	0.36	
	Christianity	0.21	(-0.42, 0.84)	0.32		0.97	(0.26, 1.67)	0.36		0.75	(0.05, 1.44)	0.36	

Variable	Category	Cantril's Ladder				Life Satisfaction				Happiness			
		Est	95% CI	SE	Global p-value	Est	95% CI	SE	Global p-value	Est	95% CI	SE	Global p-value
Race/ethnicity	Collapsed affiliations with prevalence<3%	-0.51	(-2.82, 1.81)	1.18		1.05	(-1.53, 3.63)	1.32		1.33	(-0.31, 2.98)	0.84	
	(Ref: Plurality group)				0.288				0.557				0.570
	Non-plurality groups	0.81	(-0.69, 2.32)	0.77		-0.34	(-1.47, 0.79)	0.58		0.19	(-0.45, 0.82)	0.33	

Table S20d. Sensitivity to unmeasured confounding of childhood predictors in Tanzania

		Cantril's Ladder		Life Satisfaction		Happiness	
Variable	Category	E-value for Estimate	E-value for 95% CI	E-value for Estimate	E-value for 95% CI	E-value for Estimate	E-value for 95% CI
Relationship with mother	(Ref: Very bad/somewhat bad)						
	Very good/somewhat good	1.27	1.00	1.14	1.00	1.38	1.00
Relationship with father	(Ref: Very bad/somewhat bad)						
	Very good/somewhat good	1.46	1.08	1.14	1.00	1.29	1.00
Parent marital status	(Ref: Parents married)						
	Divorced	1.30	1.00	1.35	1.00	1.33	1.00
	Parents were never married	1.18	1.00	1.20	1.00	1.14	1.00
	One or both parents had died	1.25	1.00	1.71	1.30	1.05	1.00
Subjective financial status of family growing up	(Ref: Got by)						
	Lived comfortably	1.30	1.00	1.20	1.00	1.37	1.15
	Found it difficult	1.37	1.13	1.52	1.31	1.41	1.23
	Found it very difficult	1.79	1.47	1.88	1.58	1.70	1.36
Abuse	(Ref: No)						
	Yes	1.37	1.00	1.60	1.34	1.59	1.31
Outsider growing up	(Ref: No)						
	Yes	1.27	1.00	1.24	1.00	1.31	1.00
Self-rated health growing up	(Ref: Good)						
	Excellent	1.24	1.00	1.28	1.00	1.10	1.00
	Very good	1.42	1.16	1.24	1.00	1.26	1.00
	Fair	1.08	1.00	1.10	1.00	1.07	1.00
	Poor	1.61	1.22	1.40	1.00	1.49	1.02
Immigration status	(Ref: Born in this country)						
	Born in another country	1.58	1.00	1.56	1.00	1.31	1.00
Age 12 religious service attendance	(Ref: Never)						
	At least 1/week	1.17	1.00	1.73	1.34	1.33	1.00
	1-3/month	1.19	1.00	1.70	1.30	1.50	1.00
	< 1/month	1.14	1.00	1.64	1.17	1.38	1.00
Year of birth	(Ref: 1998-2005; current age: 18-24)						
	1993-1998; age 25-29	1.31	1.00	1.58	1.33	1.50	1.24
	1983-1993; age 30-39	1.63	1.43	1.83	1.64	1.66	1.48
	1973-1983; age 40-49	1.73	1.52	1.97	1.75	1.98	1.77
	1963-1973; age 50-59	1.88	1.64	2.08	1.85	2.27	2.03

Variable	Category	Cantril's Ladder		Life Satisfaction		Happiness	
		E-value for Estimate	E-value for 95% CI	E-value for Estimate	E-value for 95% CI	E-value for Estimate	E-value for 95% CI
	1953-1963; age 60-69	2.08	1.73	1.78	1.43	2.13	1.74
	1943-1953; age 70-79	2.57	1.98	1.73	1.21	2.49	1.86
	1943 or earlier; age 80+	1.96	1.00	1.63	1.00	1.97	1.00
Gender	(Ref: Male)						
	Female	1.29	1.08	1.67	1.54	1.22	1.00
Religious affiliation	(Ref: No religion/Atheist/Agnostic)						
	Islam	1.21	1.00	1.69	1.11	1.52	1.00
	Christianity	1.31	1.00	1.84	1.33	1.77	1.14
	Collapsed affiliations with prevalence<3%	1.56	1.00	1.90	1.00	2.26	1.00
Race/ethnicity	(Ref: Plurality group)						
	Non-plurality groups	1.81	1.00	1.39	1.00	1.29	1.00

Table S20e. Complete-case supplemental analysis of means by demographic category for Tanzania

		Cantril's Ladder				Life Satisfaction				Happiness			
Variable	Category	Mean	95% CI	SE	Global p-value	Mean	95% CI	SE	Global p-value	Mean	95% CI	SE	Global p-value
Age group	18-24	5.66	(5.47,5.84)	0.10	0.002	7.29	(7.07,7.51)	0.11	< .001	7.68	(7.53,7.83)	0.08	< .001
	25-29	5.61	(5.40,5.83)	0.11		6.93	(6.70,7.15)	0.11		7.38	(7.18,7.57)	0.10	
	30-39	5.28	(5.09,5.48)	0.10		6.51	(6.30,6.71)	0.10		7.12	(6.96,7.28)	0.08	
	40-49	5.28	(5.06,5.49)	0.11		6.44	(6.20,6.67)	0.12		6.83	(6.63,7.04)	0.10	
	50-59	5.26	(5.00,5.52)	0.13		6.41	(6.14,6.67)	0.14		6.78	(6.55,7.01)	0.12	
	60-69	5.24	(4.82,5.65)	0.21		6.69	(6.30,7.08)	0.20		7.00	(6.66,7.34)	0.17	
	70-79	5.40	(4.68,6.13)	0.37		7.04	(6.43,7.66)	0.31		7.14	(6.61,7.67)	0.27	
	80 or older	6.37	(5.00,7.73)	0.69		6.97	(5.89,8.05)	0.54		6.78	(5.87,7.68)	0.45	
Gender	Female	5.61	(5.44,5.78)	0.09	< .001	7.03	(6.85,7.21)	0.09	< .001	7.29	(7.16,7.42)	0.07	0.041
	Male	5.26	(5.09,5.42)	0.08		6.51	(6.35,6.67)	0.08		7.14	(7.00,7.27)	0.07	
Marital status	Divorced	5.01	(4.13,5.90)	0.45	0.202	6.26	(5.43,7.09)	0.42	0.003	6.88	(6.23,7.54)	0.33	< .001
	Domestic partner	5.47	(4.99,5.95)	0.25		6.61	(6.16,7.06)	0.23		6.86	(6.40,7.31)	0.23	
	Married	5.46	(5.29,5.62)	0.09		6.81	(6.64,6.99)	0.09		7.17	(7.03,7.30)	0.07	
	Separated	4.99	(4.57,5.42)	0.22		6.07	(5.62,6.53)	0.23		6.66	(6.31,7.00)	0.18	
	Single/Never been married	5.47	(5.28,5.65)	0.09		6.95	(6.74,7.16)	0.11		7.54	(7.38,7.70)	0.08	
	Widowed	5.60	(5.10,6.09)	0.25		6.55	(6.03,7.07)	0.27		6.93	(6.54,7.31)	0.19	
Employment	Employed for an employer	5.51	(5.21,5.80)	0.15	0.205	6.84	(6.51,7.17)	0.17	< .001	7.28	(6.99,7.57)	0.15	< .001
	Homemaker	5.55	(5.31,5.80)	0.13		7.13	(6.88,7.38)	0.13		7.25	(7.06,7.44)	0.10	
	None of these/Other	5.77	(4.96,6.57)	0.41		7.13	(6.35,7.91)	0.40		7.87	(7.36,8.38)	0.26	
	Retired	5.01	(4.42,5.61)	0.30		6.64	(6.10,7.18)	0.27		6.66	(6.11,7.21)	0.28	
	Self-employed	5.35	(5.17,5.53)	0.09		6.59	(6.41,6.77)	0.09		7.13	(6.99,7.27)	0.07	
	Student	5.66	(5.33,5.99)	0.17		7.33	(6.97,7.69)	0.18		7.89	(7.60,8.18)	0.15	
	Unemployed and looking for a job	5.52	(5.29,5.76)	0.12		6.84	(6.55,7.12)	0.15		7.25	(7.04,7.46)	0.11	
Religious service attendance	A few times a year	5.40	(5.12,5.68)	0.14	0.942	6.54	(6.27,6.82)	0.14	0.217	7.02	(6.78,7.26)	0.12	0.003
	More than once a week	5.41	(5.24,5.59)	0.09		6.87	(6.67,7.07)	0.10		7.16	(7.02,7.31)	0.07	
	Never	5.47	(4.90,6.03)	0.29		7.04	(6.44,7.65)	0.31		7.14	(6.65,7.64)	0.25	
	Once a week	5.44	(5.25,5.63)	0.10		6.81	(6.60,7.02)	0.11		7.37	(7.21,7.52)	0.08	
	One to three times a month	5.54	(5.25,5.82)	0.14		6.68	(6.40,6.95)	0.14		6.93	(6.71,7.15)	0.11	

Variable	Category	Cantril's Ladder				Life Satisfaction				Happiness			
		Mean	95% CI	SE	Global p-value	Mean	95% CI	SE	Global p-value	Mean	95% CI	SE	Global p-value
Education	Up to 8 years	5.42	(5.24,5.60)	0.09	0.544	6.75	(6.56,6.93)	0.09	0.129	7.15	(7.01,7.28)	0.07	0.006
	16+ years	5.34	(4.98,5.70)	0.18		6.72	(6.28,7.16)	0.22		7.40	(7.00,7.81)	0.20	
	9 to 15 years	5.51	(5.36,5.65)	0.08		6.93	(6.78,7.09)	0.08		7.40	(7.28,7.53)	0.06	
Immigration status	Born in another country	5.19	(4.13,6.25)	0.51	0.614	7.78	(6.07,9.49)	0.82	0.227	7.84	(6.70,8.98)	0.55	0.254
	Born in this country	5.44	(5.30,5.59)	0.07		6.79	(6.64,6.94)	0.08		7.22	(7.10,7.33)	0.06	
Religious affiliation	Christianity	5.51	(5.33,5.69)	0.09	< .001	6.89	(6.72,7.06)	0.09	0.235	7.32	(7.19,7.45)	0.07	< .001
	Islam	5.33	(5.14,5.52)	0.10		6.65	(6.39,6.91)	0.13		7.04	(6.87,7.21)	0.09	
	No religion/Atheist/Agnostic	5.10	(4.54,5.66)	0.28		6.43	(5.75,7.12)	0.35		6.92	(6.42,7.42)	0.25	
	Primal, Animist, or Folk religion	5.85	(2.92,8.78)	1.14		7.10	(4.69,9.51)	0.94		7.02	(4.94,9.09)	0.81	
	Taoism	3.00	*	*		0.00	*	*		5.00	*	*	

Table S20f. Complete-case supplemental analysis of childhood predictors regression analysis results for Tanzania

		Cantril's Ladder				Life Satisfaction				Happiness			
Variable	Category	Est	95% CI	SE	Global p-value	Est	95% CI	SE	Global p-value	Est	95% CI	SE	Global p-value
Relationship with mother	(Ref: Very bad/somewhat bad)				0.685				0.922				0.288
	Very good/somewhat good	-0.08	(-0.49,0.32)	0.21		-0.02	(-0.48,0.43)	0.23		0.22	(-0.18,0.62)	0.21	
Relationship with father	(Ref: Very bad/somewhat bad)				0.154				0.611				0.471
	Very good/somewhat good	0.25	(-0.09,0.59)	0.18		0.10	(-0.29,0.50)	0.20		-0.10	(-0.38,0.18)	0.14	
Parent marital status	(Ref: Parents married)				0.026				0.023				0.006
	No, one or both of them had died	0.04	(-0.57,0.65)	0.31		0.11	(-0.41,0.62)	0.26		0.17	(-0.31,0.66)	0.25	
	No, they were never married	0.61	(0.16,1.06)	0.23		0.59	(0.19,0.99)	0.21		0.62	(0.26,0.98)	0.18	
	Yes, married	0.09	(-0.25,0.43)	0.17		0.20	(-0.10,0.50)	0.15		0.23	(-0.05,0.52)	0.15	
Subjective financial status of family growing up	(Ref: Got by)				0.002				<.001				<.001
	Found it difficult	-0.12	(-0.33,0.09)	0.11		-0.13	(-0.35,0.10)	0.11		-0.06	(-0.23,0.12)	0.09	
	Found it very difficult	-0.06	(-0.47,0.35)	0.21		-0.25	(-0.60,0.10)	0.18		-0.10	(-0.45,0.24)	0.18	
	Lived comfortably	0.34	(0.14,0.55)	0.10		0.33	(0.13,0.54)	0.10		0.35	(0.15,0.54)	0.10	
Abuse	(Ref: No)				0.043				0.009				0.024
	Yes	-0.33	(-0.65,-0.01)	0.16		-0.47	(-0.82,-0.12)	0.18		-0.32	(-0.60,-0.04)	0.14	
Outsider growing up	(Ref: No)				0.174				0.634				0.183
	Yes	0.24	(-0.10,0.58)	0.18		-0.08	(-0.41,0.25)	0.17		-0.22	(-0.54,0.10)	0.16	

		Cantril's Ladder				Life Satisfaction				Happiness			
Variable	Category	Est	95% CI	SE	Global p-value	Est	95% CI	SE	Global p-value	Est	95% CI	SE	Global p-value
Self-rated health growing up	(Ref: Good)				0.050				0.641				0.151
	Excellent	0.09	(-0.14,0.33)	0.12		0.17	(-0.05,0.39)	0.11		0.12	(-0.08,0.32)	0.10	
	Fair	-0.02	(-0.27,0.24)	0.13		0.04	(-0.23,0.32)	0.14		0.05	(-0.18,0.27)	0.11	
	Poor	-0.28	(-0.70,0.15)	0.22		-0.00	(-0.43,0.43)	0.22		-0.16	(-0.53,0.22)	0.19	
	Very good	0.26	(0.02,0.50)	0.12		0.07	(-0.20,0.33)	0.14		0.23	(0.03,0.44)	0.11	
Immigration status	(Ref: Born in this country)				0.659				0.092				0.154
	Born in another country	-0.20	(-1.11,0.70)	0.46		1.06	(-0.17,2.28)	0.62		0.70	(-0.26,1.66)	0.49	
Age 12 religious service attendance	(Ref: Never)				0.842				0.413				0.623
	At least once a week	-0.01	(-0.50,0.47)	0.25		-0.37	(-0.84,0.10)	0.24		-0.22	(-0.61,0.18)	0.20	
	Less than once a month	-0.19	(-0.80,0.42)	0.31		-0.25	(-0.85,0.35)	0.31		-0.36	(-0.96,0.23)	0.31	
	One to three times a month	-0.00	(-0.52,0.52)	0.26		-0.28	(-0.81,0.24)	0.27		-0.27	(-0.70,0.17)	0.22	
Year of birth	(Ref: 1998-2005; current age: 18-24)				0.004				<.001				<.001
	1993-1998; age 25-29	-0.02	(-0.26,0.22)	0.12		-0.35	(-0.62,-0.08)	0.14		-0.30	(-0.51,-0.08)	0.11	
	1983-1993; age 30-39	-0.36	(-0.58,-0.14)	0.11		-0.72	(-0.96,-0.48)	0.12		-0.51	(-0.71,-0.32)	0.10	
	1973-1983; age 40-49	-0.34	(-0.57,-0.11)	0.12		-0.75	(-1.04,-0.47)	0.14		-0.77	(-1.01,-0.54)	0.12	
	1963-1973; age 50-59	-0.37	(-0.65,-0.09)	0.14		-0.80	(-1.09,-0.51)	0.15		-0.87	(-1.11,-0.63)	0.12	
	1953-1963; age 60-69	-0.45	(-0.85,-0.06)	0.20		-0.60	(-1.01,-0.19)	0.21		-0.70	(-1.05,-0.35)	0.18	
	1943-1953; age 70-79	-0.31	(-1.05,0.43)	0.38		-0.21	(-0.80,0.38)	0.30		-0.55	(-1.08,-0.03)	0.27	

		Cantril's Ladder				Life Satisfaction				Happiness			
Variable	Category	Est	95% CI	SE	Global p-value	Est	95% CI	SE	Global p-value	Est	95% CI	SE	Global p-value
	1943 or earlier; age 80+	0.67	(-0.70,2.03)	0.70		-0.22	(-1.28,0.83)	0.54		-0.93	(-1.84,-0.02)	0.46	
Gender	(Ref: Male)				<.001				<.001				0.105
	Male	-0.31	(-0.48,-0.15)	0.08		-0.49	(-0.65,-0.32)	0.08		-0.12	(-0.26,0.02)	0.07	
Religious affiliation	(Ref: No religion/Atheist/Agnostic)				0.335				0.082				0.080
	Christianity	-0.18	(-0.80,0.45)	0.32		0.61	(0.02,1.20)	0.30		0.40	(-0.15,0.95)	0.28	
	Christianity	-0.18	(-0.80,0.45)	0.32		0.61	(0.02,1.20)	0.30		0.40	(-0.15,0.95)	0.28	
	Islam	-0.35	(-1.01,0.31)	0.34		0.36	(-0.27,0.99)	0.32		0.16	(-0.40,0.73)	0.29	
	Collapsed affiliations with prevalence<3 %	0.71	(-0.90,2.32)	0.82		1.13	(-0.70,2.95)	0.93		0.45	(-1.25,2.16)	0.87	
Race/ethnicity	(Ref: Plurality group)				0.044				0.467				0.211
	Non-plurality groups	0.79	(0.03,1.56)	0.39		-0.24	(-0.88,0.40)	0.33		-0.42	(-1.06,0.23)	0.33	

Table S20g. Complete-case supplemental analysis of sensitivity to unmeasured confounding of childhood predictors in Tanzania

		Cantril's Ladder		Life Satisfaction		Happiness	
Variable	Category	E-value for Estimate	E-value for 95% CI	E-value for Estimate	E-value for 95% CI	E-value for Estimate	E-value for 95% CI
Relationship with mother	(Ref: Very bad/somewhat bad)						
	Very good/somewhat good	1.20	1.00	1.09	1.00	1.37	1.00
Relationship with father	(Ref: Very bad/somewhat bad)						
	Very good/somewhat good	1.39	1.00	1.22	1.00	1.23	1.00
Parent marital status	(Ref: Parents married)						
	No, one or both of them had died	1.13	1.00	1.22	1.00	1.32	1.00
	No, they were never married	1.73	1.29	1.70	1.32	1.78	1.41
	Yes, married	1.20	1.00	1.33	1.00	1.38	1.00
Subjective financial status of family growing up	(Ref: Got by)						
	Found it difficult	1.24	1.00	1.25	1.00	1.16	1.00
	Found it very difficult	1.16	1.00	1.39	1.00	1.23	1.00
	Lived comfortably	1.48	1.27	1.46	1.25	1.50	1.29
Abuse	(Ref: No)						
	Yes	1.47	1.07	1.59	1.24	1.48	1.14
Outsider growing up	(Ref: No)						
	Yes	1.38	1.00	1.19	1.00	1.37	1.00
Self-rated health growing up	(Ref: Good)						
	Excellent	1.21	1.00	1.29	1.00	1.25	1.00
	Fair	1.09	1.00	1.13	1.00	1.14	1.00
	Poor	1.41	1.00	1.02	1.00	1.30	1.00
	Very good	1.40	1.08	1.17	1.00	1.38	1.11
Immigration status	(Ref: Born in this country)						
	Born in another country	1.34	1.00	2.14	1.00	1.86	1.00
Age 12 religious service attendance	(Ref: Never)						
	At least once a week	1.07	1.00	1.50	1.00	1.36	1.00
	Less than once a month	1.32	1.00	1.38	1.00	1.52	1.00
	One to three times a month	1.02	1.00	1.42	1.00	1.42	1.00
Year of birth	(Ref: 1998-2005; current age: 18-24)						

Variable	Category	Cantril's Ladder		Life Satisfaction		Happiness	
		E-value for Estimate	E-value for 95% CI	E-value for Estimate	E-value for 95% CI	E-value for Estimate	E-value for 95% CI
	1993-1998; age 25-29	1.10	1.00	1.48	1.20	1.45	1.20
	1983-1993; age 30-39	1.50	1.27	1.82	1.60	1.67	1.47
	1973-1983; age 40-49	1.48	1.23	1.86	1.60	1.93	1.70
	1963-1973; age 50-59	1.51	1.21	1.90	1.63	2.03	1.79
	1953-1963; age 60-69	1.59	1.16	1.71	1.31	1.86	1.50
	1943-1953; age 70-79	1.45	1.00	1.34	1.00	1.71	1.11
	1943 or earlier; age 80+	1.79	1.00	1.35	1.00	2.10	1.10
Gender	(Ref: Male)						
	Male	1.45	1.28	1.61	1.46	1.25	1.00
Religious affiliation	(Ref: No religion/Atheist/Agnostic)						
	Christianity	1.31	1.00	1.73	1.10	1.56	1.00
	Christianity	1.31	1.00	1.73	1.10	1.56	1.00
	Islam	1.49	1.00	1.49	1.00	1.30	1.00
	Collapsed affiliations with prevalence<3%	1.83	1.00	2.21	1.00	1.61	1.00
Race/ethnicity	(Ref: Plurality group)						
	Non-plurality groups	1.91	1.10	1.37	1.00	1.57	1.00

Tables S21a-g: Turkey

Table S21a. Nationally representative descriptive statistics for Turkey

Characteristic	N = 1,473[1]
**Age group**	
1998-2005; age 18-24	222 (15%)
1993-1998; age 25-29	152 (10%)
1983-1993; age 30-39	315 (21%)
1973-1983; age 40-49	312 (21%)
1963-1973; age 50-59	225 (15%)
1953-1963; age 60-69	164 (11%)
1943-1953; age 70-79	65 (4.4%)
1943 or earlier; age 80+	18 (1.2%)
(Missing)	0 (0%)
**Gender**	
Male	754 (51%)
Female	719 (49%)
Other	0 (0%)
(Missing)	0 (0%)
**Race/Ethnicity**	
Albanian	8 (0.5%)
Arab	51 (3.5%)
Armenian	1 (<0.1%)
Azeri	9 (0.6%)
Bosnian	5 (0.3%)
Circassian	19 (1.3%)
Georgian	4 (0.3%)
Greek	1 (<0.1%)
Kurdish/Zaza	252 (17%)
Laz	25 (1.7%)
Other	58 (3.9%)
Turkish	1,030 (70%)
Uyghur	1 (<0.1%)
(Missing)	9 (0.6%)
**Respondent Marital status**	
Married	936 (64%)
Separated	13 (0.9%)
Divorced	64 (4.3%)
Widowed	64 (4.3%)
Single, never married	379 (26%)
Domestic Partner	0 (0%)
(Missing)	17 (1.1%)
**Employment**	
Employed for an employer	413 (28%)
Self-employed	255 (17%)
Retired	205 (14%)
Student	107 (7.3%)
Homemaker	347 (24%)
Unemployed and looking for a job	87 (5.9%)
None of these/Other	59 (4.0%)
(Missing)	0 (0%)
**Religious service attendance as an adult (now)**	
More than 1/week	493 (33%)
1/week	271 (18%)
1-3/month	174 (12%)
A few times a year	255 (17%)
Never	274 (19%)
(Missing)	6 (0.4%)
**Education (years)**	
Up to 8 years	436 (30%)
9-15 years	711 (48%)
16+ years	326 (22%)
(Missing)	0 (0%)
**Immigration status**	
Born in this country	1,415 (96%)

Characteristic	N = 1,473[1]
Born in another country	58 (4.0%)
(Missing)	0 (0%)
**Religious affiliation as an adult (now)**	
Christianity	2 (0.1%)
Islam	1,381 (94%)
Hinduism	0 (0%)
Buddhism	1 (<0.1%)
Judaism	1 (<0.1%)
Sikhism	1 (<0.1%)
Baha'i	0 (0%)
Jainism	0 (0%)
Shinto	0 (0%)
Taoism	0 (0%)
Confucianism	0 (0%)
Primal, Animist, or Folk religion	1 (<0.1%)
Spiritism	0 (0%)
Umbanda, Candomble, and other African-derived religions	0 (0%)
Chinese folk/traditional religion	0 (0%)
Some other religion	1 (<0.1%)
No religion/Atheist/Agnostic	66 (4.5%)
(Missing)	19 (1.3%)
**Relationship with mother growing up**	
Very good	970 (66%)
Somewhat good	401 (27%)
Somewhat bad	48 (3.2%)
Very bad	26 (1.8%)
Does not apply	21 (1.4%)
(Missing)	7 (0.5%)
**Relationship with father growing up**	
Very good	795 (54%)
Somewhat good	425 (29%)
Somewhat bad	73 (5.0%)
Very bad	95 (6.5%)
Does not apply	60 (4.1%)
(Missing)	25 (1.7%)
**Parent marital status at age 12**	
Parents married	1,325 (90%)
Divorced	57 (3.9%)
Parents were never married	7 (0.5%)
One or both parents had died	61 (4.1%)
(Missing)	23 (1.5%)
**Subjective financial status of family growing up**	
Lived comfortably	498 (34%)
Got by	647 (44%)
Found it difficult	218 (15%)
Found it very difficult	108 (7.3%)
(Missing)	2 (0.1%)
**Abuse**	
Yes	158 (11%)
No	1,290 (88%)
(Missing)	25 (1.7%)
**Outsider growing up**	
Yes	157 (11%)
No	1,306 (89%)
(Missing)	9 (0.6%)
**Self-rated health growing up**	
Excellent	377 (26%)
Very good	410 (28%)
Good	419 (28%)
Fair	220 (15%)
Poor	47 (3.2%)
(Missing)	0 (<0.1%)
**Age 12 religious service attendance**	
At least 1/week	609 (41%)
1-3/month	238 (16%)

**Characteristic**	**N = 1,473**[1]
<1/month	225 (15%)
Never	383 (26%)
(Missing)	18 (1.2%)
**Religious affiliation at age 12**	
Christianity	1 (<0.1%)
Islam	1,439 (98%)
Hinduism	0 (0%)
Buddhism	0 (0%)
Judaism	1 (<0.1%)
Sikhism	0 (0%)
Baha'i	0 (0%)
Jainism	0 (0%)
Shinto	0 (0%)
Taoism	0 (0%)
Confucianism	0 (0%)
Primal, Animist, or Folk religion	0 (0%)
Spiritism	0 (0%)
Umbanda, Candomble, and other African-derived religions	0 (0%)
Chinese folk/traditional religion	0 (0%)
Some other religion	0 (0%)
No religion/Atheist/Agnostic	13 (0.9%)
(Missing)	19 (1.3%)

[1]n (%)

Table S21b. Means by demographic category for Turkey

Variable	Category	Cantril's Ladder Mean	95% CI	SE	Global p-value	Life Satisfaction Mean	95% CI	SE	Global p-value	Happiness Mean	95% CI	SE	Global p-value
Age group	18-24	5.39	(5.06,5.72)	0.17	0.046	4.99	(4.56,5.41)	0.22	0.261	5.44	(5.05,5.83)	0.20	0.026
	25-29	5.20	(4.71,5.68)	0.25		4.85	(4.29,5.40)	0.28		5.39	(4.87,5.92)	0.27	
	30-39	4.80	(4.46,5.14)	0.17		5.07	(4.67,5.47)	0.20		5.24	(4.86,5.63)	0.20	
	40-49	5.01	(4.67,5.34)	0.17		5.19	(4.78,5.59)	0.21		5.36	(4.99,5.73)	0.19	
	50-59	5.72	(5.30,6.14)	0.21		5.83	(5.27,6.38)	0.28		6.25	(5.78,6.73)	0.24	
	60-69	5.18	(4.52,5.85)	0.34		5.11	(4.22,6.01)	0.45		5.73	(4.94,6.51)	0.40	
	70-79	5.21	(3.93,6.48)	0.64		5.04	(3.39,6.69)	0.82		5.16	(3.78,6.55)	0.69	
	80 or older	5.28	(3.39,7.17)	0.82		6.19	(3.86,8.52)	1.01		6.90	(4.95,8.84)	0.84	
Gender	Male	5.19	(4.97,5.40)	0.11	0.943	5.08	(4.82,5.35)	0.14	0.299	5.42	(5.18,5.67)	0.13	0.219
	Female	5.18	(4.91,5.44)	0.14		5.31	(4.98,5.63)	0.17		5.66	(5.37,5.95)	0.15	
Marital status	Married	5.14	(4.92,5.35)	0.11	0.903	5.33	(5.06,5.61)	0.14	0.148	5.64	(5.39,5.89)	0.13	0.324
	Separated	5.08	(4.03,6.14)	0.43		5.96	(3.50,8.42)	1.01		5.53	(3.83,7.22)	0.70	
	Divorced	5.39	(4.66,6.13)	0.37		4.81	(3.78,5.83)	0.51		5.22	(4.23,6.20)	0.49	
	Widowed	5.60	(4.31,6.89)	0.64		5.54	(4.04,7.03)	0.75		5.96	(4.80,7.13)	0.58	
	Never	5.19	(4.92,5.46)	0.14		4.83	(4.50,5.15)	0.17		5.26	(4.97,5.56)	0.15	
Employment	Employed for an employer	5.15	(4.90,5.40)	0.13	0.135	5.21	(4.86,5.57)	0.18	0.073	5.67	(5.34,5.99)	0.17	0.030
	Self-employed	5.22	(4.87,5.58)	0.18		5.20	(4.72,5.67)	0.24		5.63	(5.17,6.09)	0.23	
	Retired	5.66	(5.12,6.21)	0.28		5.30	(4.53,6.06)	0.39		5.73	(5.08,6.37)	0.33	
	Student	5.34	(5.04,5.63)	0.15		4.85	(4.39,5.31)	0.23		5.41	(5.03,5.79)	0.19	
	Homemaker	5.10	(4.66,5.55)	0.23		5.55	(5.06,6.05)	0.25		5.57	(5.11,6.02)	0.23	
	Unemployed and looking for a job	4.53	(3.83,5.22)	0.35		4.18	(3.39,4.97)	0.40		4.45	(3.84,5.06)	0.31	
	None of these/Other	4.67	(3.90,5.43)	0.38		4.67	(3.68,5.66)	0.49		5.30	(4.34,6.25)	0.47	
Religious service attendance	More than 1/week	5.42	(5.11,5.74)	0.16	< .001	5.73	(5.33,6.12)	0.20	< .001	6.07	(5.73,6.40)	0.17	< .001
	1/week	5.42	(4.99,5.84)	0.22		5.53	(5.03,6.03)	0.25		5.83	(5.38,6.28)	0.23	
	1-3/month	5.79	(5.34,6.24)	0.23		5.27	(4.66,5.87)	0.31		5.65	(5.07,6.24)	0.30	
	A few times a year	4.94	(4.63,5.26)	0.16		4.49	(4.05,4.92)	0.22		4.93	(4.53,5.33)	0.20	
	Never	4.35	(4.01,4.70)	0.18		4.51	(4.05,4.97)	0.23		4.80	(4.36,5.23)	0.22	
Education	Up to 8 years	5.15	(4.74,5.57)	0.21	0.058	5.27	(4.77,5.76)	0.25	0.047	5.63	(5.19,6.07)	0.22	0.153
	9-15 years	5.08	(4.86,5.30)	0.11		5.01	(4.71,5.30)	0.15		5.39	(5.11,5.66)	0.14	
	16+ years	5.44	(5.23,5.65)	0.11		5.50	(5.24,5.75)	0.13		5.74	(5.52,5.97)	0.11	
Immigration status	Born in this country	5.17	(5.00,5.34)	0.09	0.364	5.18	(4.96,5.40)	0.11	0.614	5.54	(5.34,5.74)	0.10	0.910

Variable	Category	Cantril's Ladder				Life Satisfaction				Happiness			
		Mean	95% CI	SE	Global p-value	Mean	95% CI	SE	Global p-value	Mean	95% CI	SE	Global p-value
	Born in another country	5.52	(4.77,6.26)	0.37		5.47	(4.34,6.61)	0.57		5.48	(4.49,6.48)	0.49	
Religious affiliation	Christianity	4.20	*	*	< .001	1.60	*	*	< .001	1.71	*	*	< .001
	Islam	5.19	(5.02,5.37)	0.09		5.22	(5.00,5.45)	0.11		5.56	(5.36,5.76)	0.10	
	Buddhism	9.00	*	*		9.00	*	*		9.00	*	*	
	Judaism	5.00	*	*		10.00	*	*		10.00	*	*	
	Sikhism	4.00	*	*		5.00	*	*		5.00	*	*	
	Primal, Animist, or Folk religion	3.39	*	*		3.96	*	*		5.39	*	*	
	Some other religion	6.71	*	*		7.08	*	*		7.40	*	*	
	No religion/Atheist /Agnostic	4.94	(4.49,5.38)	0.22		4.59	(4.05,5.13)	0.27		5.13	(4.57,5.70)	0.28	
Race/Ethnicity	Arab	5.52	(4.40,6.64)	0.55	< .001	4.33	(2.90,5.76)	0.70	< .001	5.18	(3.84,6.51)	0.66	< .001
	Turkish	5.32	(5.13,5.51)	0.10		5.39	(5.15,5.64)	0.12		5.76	(5.55,5.98)	0.11	
	Greek	3.57	*	*		1.01	*	*		3.43	*	*	
	Kurdish/Zaza	4.48	(4.02,4.94)	0.23		4.51	(3.97,5.05)	0.28		4.77	(4.25,5.28)	0.26	
	Laz	5.31	(4.00,6.62)	0.59		6.52	(4.94,8.11)	0.71		6.98	(5.39,8.56)	0.71	
	Circassian	3.73	(1.90,5.56)	0.66		3.42	(1.75,5.10)	0.61		4.32	(1.77,6.88)	0.92	
	Bosnian	4.99	*	*		5.25	*	*		7.55	*	*	
	Armenian	4.57	*	*		6.00	*	*		5.86	*	*	
	Georgian	5.60	*	*		5.99	*	*		6.57	*	*	
	Uyghur	0.00	*	*		5.00	*	*		5.00	*	*	
	Albanian	5.83	*	*		4.71	*	*		2.99	*	*	
	Azeri	6.62	*	*		2.40	*	*		3.53	*	*	
	Other	5.65	(4.68,6.61)	0.48		5.87	(4.75,6.99)	0.55		5.44	(4.26,6.62)	0.59	

Table S21c. Childhood predictors regression analysis results for Turkey

		Cantril's Ladder				Life Satisfaction				Happiness			
Variable	Category	Est	95% CI	SE	Global p-value	Est	95% CI	SE	Global p-value	Est	95% CI	SE	Global p-value
Relationship with mother	(Ref: Very bad/somewhat bad)				0.810				0.295				0.013
	Very good/somewhat good	0.03	(-0.73, 0.79)	0.39		-0.50	(-1.44, 0.45)	0.48		-1.03	(-1.85, -0.21)	0.42	
Relationship with father	(Ref: Very bad/somewhat bad)				0.426				0.140				0.319
	Very good/somewhat good	0.21	(-0.32, 0.73)	0.27		-0.47	(-1.10, 0.16)	0.32		-0.29	(-0.86, 0.29)	0.29	
Parent marital status	(Ref: Parents married)				0.312				<.001				0.003
	Divorced	0.61	(-0.27, 1.48)	0.45		1.78	(0.80, 2.76)	0.50		1.49	(0.61, 2.37)	0.45	
	Parents were never married	0.81	(-0.39, 2.01)	0.61		-0.88	(-2.14, 0.38)	0.64		0.75	(-0.65, 2.16)	0.72	
	One or both parents had died	0.01	(-1.01, 1.04)	0.52		0.88	(-0.16, 1.91)	0.53		0.66	(-0.33, 1.65)	0.50	
Subjective financial status of family growing up	(Ref: Got by)				0.002				0.001				0.016
	Lived comfortably	0.54	(0.16, 0.92)	0.20		0.60	(0.12, 1.07)	0.24		0.30	(-0.12, 0.72)	0.21	
	Found it difficult	-0.13	(-0.60, 0.33)	0.24		-0.24	(-0.81, 0.33)	0.29		-0.47	(-1.02, 0.07)	0.28	
	Found it very difficult	-0.88	(-1.77, 0.01)	0.45		-1.18	(-2.10, -0.27)	0.47		-0.83	(-1.64, -0.02)	0.41	
Abuse	(Ref: No)				0.114				<.001				0.056
	Yes	-0.38	(-0.88, 0.11)	0.25		-1.08	(-1.71, -0.44)	0.32		-0.58	(-1.18, 0.02)	0.31	
Outsider growing up	(Ref: No)				0.950				0.794				0.364
	Yes	0.00	(-0.55, 0.56)	0.28		0.08	(-0.56, 0.72)	0.33		-0.27	(-0.87, 0.33)	0.31	
Self-rated health growing up	(Ref: Good)				0.142				<.001				<.001
	Excellent	0.14	(-0.30, 0.59)	0.23		-0.07	(-0.61, 0.48)	0.28		0.06	(-0.43, 0.55)	0.25	

Variable	Category	Cantril's Ladder				Life Satisfaction				Happiness			
		Est	95% CI	SE	Global p-value	Est	95% CI	SE	Global p-value	Est	95% CI	SE	Global p-value
	Very good	0.51	(0.04, 0.98)	0.24		0.25	(-0.32, 0.81)	0.29		0.12	(-0.38, 0.62)	0.25	
	Fair	0.15	(-0.41, 0.70)	0.28		-0.44	(-1.10, 0.23)	0.34		-0.58	(-1.17, 0.01)	0.30	
	Poor	-0.61	(-1.75, 0.52)	0.58		-2.64	(-3.64, -1.65)	0.51		-1.98	(-2.94, -1.01)	0.49	
Immigration status	(Ref: Born in this country)				0.189				0.294				0.648
	Born in another country	0.56	(-0.28, 1.40)	0.43		0.55	(-0.48, 1.57)	0.52		0.22	(-0.73, 1.18)	0.49	
Age 12 religious service attendance	(Ref: Never)				0.232				0.014				0.006
	At least 1/week	0.29	(-0.15, 0.73)	0.23		0.58	(0.04, 1.11)	0.27		0.65	(0.17, 1.14)	0.25	
	1-3/month	0.54	(0.02, 1.07)	0.27		0.96	(0.33, 1.60)	0.32		0.95	(0.39, 1.51)	0.29	
	< 1/month	0.33	(-0.18, 0.84)	0.26		0.24	(-0.38, 0.87)	0.32		0.41	(-0.18, 1.00)	0.30	
Year of birth	(Ref: 1998-2005; current age: 18-24)				0.043				0.117				0.051
	1993-1998; age 25-29	-0.07	(-0.62, 0.49)	0.28		0.06	(-0.62, 0.75)	0.35		0.08	(-0.54, 0.70)	0.32	
	1983-1993; age 30-39	-0.44	(-0.91, 0.02)	0.24		0.41	(-0.15, 0.96)	0.28		0.02	(-0.51, 0.55)	0.27	
	1973-1983; age 40-49	-0.22	(-0.69, 0.26)	0.24		0.54	(-0.02, 1.09)	0.28		0.19	(-0.32, 0.70)	0.26	
	1963-1973; age 50-59	0.50	(-0.02, 1.02)	0.26		1.03	(0.34, 1.72)	0.35		0.95	(0.34, 1.56)	0.31	
	1953-1963; age 60-69	-0.13	(-0.85, 0.59)	0.37		0.32	(-0.61, 1.26)	0.48		0.42	(-0.42, 1.25)	0.42	
	1943-1953; age 70-79	-0.19	(-1.53, 1.15)	0.68		0.12	(-1.29, 1.53)	0.72		-0.26	(-1.49, 0.96)	0.62	
	1943 or earlier; age 80+	-0.08	(-1.89, 1.73)	0.92		1.22	(-0.76, 3.19)	1.01		1.45	(-0.54, 3.43)	1.01	
Gender	(Ref: Male)				0.876				0.228				0.126
	Female	-0.03	(-0.38, 0.33)	0.18		0.25	(-0.16, 0.67)	0.21		0.29	(-0.08, 0.67)	0.19	
Religious affiliation	(Ref: Islam)				0.160				0.318				0.619
	Collapsed affiliations with prevalence<3%	-0.61	(-1.47, 0.25)	0.44		-0.65	(-1.97, 0.66)	0.67		-0.29	(-1.55, 0.97)	0.64	
Race/ethnicity	(Ref: Plurality group)				0.050				0.007				0.021

		Cantril’s Ladder				Life Satisfaction				Happiness			
Variable	Category	Est	95% CI	SE	Global p-value	Est	95% CI	SE	Global p-value	Est	95% CI	SE	Global p-value
	Non-plurality groups	-0.41	(-0.88, 0.06)	0.24		-0.64	(-1.12, -0.15)	0.24		-0.52	(-0.98, -0.06)	0.23	

Table S21d. Sensitivity to unmeasured confounding of childhood predictors in Turkey

		Cantril's Ladder		Life Satisfaction		Happiness	
Variable	Category	E-value for Estimate	E-value for 95% CI	E-value for Estimate	E-value for 95% CI	E-value for Estimate	E-value for 95% CI
Relationship with mother	(Ref: Very bad/somewhat bad)						
	Very good/somewhat good	1.11	1.00	1.56	1.00	2.10	1.34
Relationship with father	(Ref: Very bad/somewhat bad)						
	Very good/somewhat good	1.36	1.00	1.54	1.00	1.41	1.00
Parent marital status	(Ref: Parents married)						
	Divorced	1.79	1.00	2.68	1.82	2.55	1.72
	Parents were never married	2.00	1.00	1.88	1.00	1.84	1.00
	One or both parents had died	1.07	1.00	1.88	1.00	1.75	1.00
Subjective financial status of family growing up	(Ref: Got by)						
	Lived comfortably	1.72	1.31	1.65	1.22	1.43	1.00
	Found it difficult	1.28	1.00	1.34	1.00	1.59	1.00
	Found it very difficult	2.08	1.00	2.14	1.37	1.91	1.08
Abuse	(Ref: No)						
	Yes	1.56	1.00	2.04	1.52	1.68	1.00
Outsider growing up	(Ref: No)						
	Yes	1.04	1.00	1.18	1.00	1.40	1.00
Self-rated health growing up	(Ref: Good)						
	Excellent	1.29	1.00	1.16	1.00	1.16	1.00
	Very good	1.68	1.13	1.35	1.00	1.24	1.00
	Fair	1.29	1.00	1.51	1.00	1.68	1.00
	Poor	1.79	1.00	3.62	2.56	3.10	2.08
Immigration status	(Ref: Born in this country)						
	Born in another country	1.74	1.00	1.61	1.00	1.35	1.00
Age 12 religious service attendance	(Ref: Never)						
	At least 1/week	1.45	1.00	1.63	1.12	1.75	1.29
	1-3/month	1.72	1.09	1.95	1.42	2.02	1.51
	< 1/month	1.50	1.00	1.35	1.00	1.53	1.00
Year of birth	(Ref: 1998-2005; current age: 18-24)						
	1993-1998; age 25-29	1.18	1.00	1.16	1.00	1.18	1.00
	1983-1993; age 30-39	1.62	1.00	1.49	1.00	1.08	1.00
	1973-1983; age 40-49	1.37	1.00	1.60	1.00	1.32	1.00
	1963-1973; age 50-59	1.67	1.00	2.01	1.44	2.02	1.47

Variable	Category	Cantril's Ladder		Life Satisfaction		Happiness	
		E-value for Estimate	E-value for 95% CI	E-value for Estimate	E-value for 95% CI	E-value for Estimate	E-value for 95% CI
	1953-1963; age 60-69	1.27	1.00	1.42	1.00	1.53	1.00
	1943-1953; age 70-79	1.34	1.00	1.22	1.00	1.39	1.00
	1943 or earlier; age 80+	1.20	1.00	2.16	1.00	2.51	1.00
Gender	(Ref: Male)						
	Female	1.11	1.00	1.36	1.00	1.42	1.00
Religious affiliation	(Ref: Islam)						
	Collapsed affiliations with prevalence<3%	1.79	1.00	1.69	1.00	1.42	1.00
Race/ethnicity	(Ref: Plurality group)						
	Non-plurality groups	1.58	1.00	1.68	1.26	1.63	1.16

Table S21e. Complete-case supplemental analysis of means by demographic category for Turkey

		Cantril's Ladder				Life Satisfaction				Happiness			
Variable	Category	Mean	95% CI	SE	Global p-value	Mean	95% CI	SE	Global p-value	Mean	95% CI	SE	Global p-value
Age group	18-24	5.55	(5.24,5.86)	0.16	0.244	5.57	(5.18,5.95)	0.20	0.051	5.78	(5.43,6.14)	0.18	0.076
	25-29	5.52	(5.08,5.96)	0.22		5.62	(5.10,6.14)	0.26		5.99	(5.53,6.44)	0.23	
	30-39	5.34	(5.06,5.61)	0.14		5.94	(5.57,6.30)	0.18		5.98	(5.66,6.29)	0.16	
	40-49	5.44	(5.15,5.73)	0.15		6.08	(5.75,6.42)	0.17		5.90	(5.58,6.22)	0.16	
	50-59	6.03	(5.65,6.41)	0.19		6.51	(6.07,6.95)	0.23		6.54	(6.13,6.95)	0.21	
	60-69	5.55	(4.96,6.14)	0.30		6.28	(5.55,7.00)	0.37		6.56	(5.98,7.15)	0.30	
	70-79	5.72	(4.55,6.89)	0.58		6.71	(5.34,8.09)	0.69		6.03	(4.80,7.25)	0.61	
	80 or older	5.54	(3.64,7.44)	0.82		6.19	(3.86,8.52)	1.01		6.90	(4.95,8.84)	0.84	
Gender	Female	5.60	(5.37,5.83)	0.12	0.603	6.15	(5.88,6.42)	0.14	0.194	6.23	(6.00,6.47)	0.12	0.109
	Male	5.52	(5.33,5.72)	0.10		5.92	(5.69,6.15)	0.12		5.97	(5.76,6.19)	0.11	
Marital status	Divorced	5.70	(4.99,6.40)	0.35	0.453	5.74	(4.82,6.66)	0.46	0.005	5.89	(4.98,6.80)	0.45	0.006
	Married	5.57	(5.39,5.76)	0.10		6.20	(5.98,6.43)	0.12		6.24	(6.04,6.45)	0.11	
	Separated	5.38	(4.62,6.14)	0.31		6.32	(3.87,8.76)	1.01		5.67	(3.96,7.37)	0.70	
	Single/Never been married	5.40	(5.14,5.66)	0.13		5.54	(5.25,5.83)	0.15		5.69	(5.42,5.95)	0.14	
	Widowed	6.33	(5.20,7.46)	0.56		6.85	(5.69,8.01)	0.58		6.83	(6.06,7.59)	0.38	
Employment	Employed for an employer	5.32	(5.09,5.56)	0.12	0.029	5.99	(5.69,6.29)	0.15	0.002	6.16	(5.88,6.44)	0.14	< .001
	Homemaker	5.79	(5.41,6.17)	0.19		6.41	(6.00,6.83)	0.21		6.24	(5.88,6.61)	0.19	
	None of these/Other	5.07	(4.50,5.63)	0.28		5.10	(4.09,6.11)	0.50		5.65	(4.70,6.59)	0.47	
	Retired	6.02	(5.52,6.53)	0.26		6.33	(5.71,6.95)	0.31		6.22	(5.70,6.75)	0.27	
	Self-employed	5.62	(5.31,5.93)	0.16		6.05	(5.65,6.44)	0.20		6.36	(5.98,6.73)	0.19	
	Student	5.41	(5.12,5.69)	0.14		5.31	(4.89,5.72)	0.21		5.62	(5.27,5.96)	0.17	
	Unemployed and looking for a job	5.12	(4.48,5.76)	0.32		5.61	(4.90,6.32)	0.36		5.12	(4.58,5.65)	0.27	
Religious service attendance	A few times a year	5.23	(4.95,5.52)	0.14	< .001	5.36	(5.01,5.72)	0.18	< .001	5.51	(5.20,5.82)	0.16	< .001
	More than once a week	5.77	(5.50,6.04)	0.14		6.57	(6.23,6.90)	0.17		6.59	(6.32,6.86)	0.14	
	Never	4.86	(4.57,5.14)	0.15		5.38	(4.99,5.77)	0.20		5.45	(5.07,5.83)	0.19	
	Once a week	5.94	(5.58,6.30)	0.18		6.40	(5.99,6.82)	0.21		6.34	(5.96,6.73)	0.19	
	One to three times a month	5.93	(5.50,6.37)	0.22		5.90	(5.37,6.43)	0.27		6.13	(5.61,6.65)	0.26	
Education	Up to 8 years	5.81	(5.45,6.17)	0.18	0.113	6.39	(5.97,6.81)	0.21	0.090	6.31	(5.93,6.69)	0.19	0.337
	16+ years	5.60	(5.40,5.79)	0.10		5.86	(5.62,6.10)	0.12		5.98	(5.77,6.20)	0.11	

Variable	Category	Cantril's Ladder				Life Satisfaction				Happiness			
		Mean	95% CI	SE	Global p-value	Mean	95% CI	SE	Global p-value	Mean	95% CI	SE	Global p-value
	9 to 15 years	5.40	(5.21,5.60)	0.10		5.92	(5.67,6.17)	0.13		6.03	(5.80,6.25)	0.11	
Immigration status	Born in another country	5.52	(4.77,6.26)	0.37	0.900	6.36	(5.37,7.34)	0.49	0.503	6.07	(5.23,6.90)	0.42	0.937
	Born in this country	5.56	(5.41,5.71)	0.08		6.02	(5.84,6.20)	0.09		6.10	(5.94,6.26)	0.08	
Religious affiliation	Buddhism	9.00	*	*	< .001	9.00	*	*	< .001	9.00	*	*	< .001
	Christianity	3.91	*	*		4.00	*	*		4.00	*	*	
	Islam	5.59	(5.44,5.75)	0.08		6.08	(5.89,6.26)	0.09		6.13	(5.96,6.29)	0.08	
	Judaism	5.00	*	*		10.00	*	*		10.00	*	*	
	No religion/Atheist/Agnostic	4.97	(4.53,5.41)	0.22		5.08	(4.60,5.57)	0.24		5.49	(5.00,5.98)	0.25	
	Primal, Animist, or Folk religion	6.00	*	*		7.00	*	*		5.39	*	*	
	Sikhism	4.00	*	*		5.00	*	*		5.00	*	*	
	Some other religion	6.58	*	*		7.16	*	*		7.16	*	*	

Table S21f. Complete-case supplemental analysis of childhood predictors regression analysis results for Turkey

		Cantril's Ladder				Life Satisfaction				Happiness			
Variable	Category	Est	95% CI	SE	Global p-value	Est	95% CI	SE	Global p-value	Est	95% CI	SE	Global p-value
Relationship with mother	(Ref: Very bad/somewhat bad)				0.499				0.712				0.086
	Very good/somewhat good	-0.22	(-0.86,0.42)	0.33		-0.16	(-1.01,0.69)	0.43		-0.65	(-1.39,0.09)	0.38	
Relationship with father	(Ref: Very bad/somewhat bad)				0.304				0.485				0.651
	Very good/somewhat good	0.25	(-0.23,0.74)	0.25		-0.20	(-0.77,0.36)	0.29		-0.12	(-0.62,0.39)	0.26	
Parent marital status	(Ref: Parents married)				0.593				0.011				0.059
	No, one or both of them had died	-0.43	(-1.66,0.79)	0.62		-1.04	(-2.31,0.23)	0.65		-0.75	(-1.91,0.41)	0.59	
	No, they were never married	0.22	(-1.14,1.59)	0.70		-2.46	(-3.92,-0.99)	0.75		-1.00	(-2.49,0.49)	0.76	
	Yes, married	-0.38	(-1.25,0.49)	0.44		-1.20	(-2.14,-0.26)	0.48		-1.15	(-2.02,-0.28)	0.44	
Subjective financial status of family growing up	(Ref: Got by)				0.041				0.007				0.041
	Found it difficult	-0.12	(-0.55,0.30)	0.22		-0.05	(-0.54,0.45)	0.25		-0.07	(-0.53,0.38)	0.23	
	Found it very difficult	-0.35	(-1.19,0.49)	0.43		-0.53	(-1.52,0.47)	0.51		-0.07	(-0.83,0.69)	0.39	
	Lived comfortably	0.43	(0.08,0.77)	0.18		0.60	(0.20,1.00)	0.20		0.47	(0.11,0.83)	0.18	
Abuse	(Ref: No)				0.006				0.002				0.084
	Yes	-0.67	(-1.14,-0.19)	0.24		-0.84	(-1.38,-0.30)	0.28		-0.47	(-1.00,0.06)	0.27	
Outsider growing up	(Ref: No)				0.593				0.997				0.177
	Yes	-0.15	(-0.70,0.40)	0.28		0.00	(-0.58,0.58)	0.30		-0.34	(-0.82,0.15)	0.25	

		Cantril's Ladder				Life Satisfaction				Happiness			
Variable	Category	Est	95% CI	SE	Global p-value	Est	95% CI	SE	Global p-value	Est	95% CI	SE	Global p-value
Self-rated health growing up	(Ref: Good)				0.672				0.010				<.001
	Excellent	0.13	(-0.25,0.51)	0.19		0.13	(-0.37,0.64)	0.26		0.00	(-0.42,0.42)	0.21	
	Fair	0.21	(-0.25,0.67)	0.24		-0.22	(-0.81,0.38)	0.30		-0.65	(-1.17,-0.13)	0.26	
	Poor	-0.02	(-0.99,0.95)	0.49		-1.59	(-2.66,-0.52)	0.55		-1.84	(-2.80,-0.89)	0.49	
	Very good	0.31	(-0.11,0.74)	0.22		0.30	(-0.18,0.78)	0.25		0.01	(-0.41,0.42)	0.21	
Immigration status	(Ref: Born in this country)				0.846				0.450				0.786
	Born in another country	0.08	(-0.69,0.84)	0.39		0.36	(-0.57,1.29)	0.47		0.11	(-0.69,0.92)	0.41	
Age 12 religious service attendance	(Ref: Never)				0.903				0.112				0.063
	At least once a week	0.06	(-0.33,0.44)	0.20		0.31	(-0.15,0.78)	0.24		0.50	(0.08,0.92)	0.21	
	Less than once a month	-0.02	(-0.47,0.43)	0.23		-0.16	(-0.70,0.39)	0.28		0.16	(-0.32,0.65)	0.25	
	One to three times a month	0.16	(-0.34,0.66)	0.25		0.42	(-0.13,0.97)	0.28		0.54	(0.04,1.04)	0.25	
Year of birth	(Ref: 1998-2005; current age: 18-24)				0.151				0.004				0.011
	1993-1998; age 25-29	0.03	(-0.50,0.55)	0.27		0.13	(-0.51,0.77)	0.32		0.23	(-0.34,0.80)	0.29	
	1983-1993; age 30-39	-0.17	(-0.59,0.24)	0.21		0.49	(-0.03,1.01)	0.26		0.31	(-0.17,0.78)	0.24	
	1973-1983; age 40-49	-0.06	(-0.49,0.36)	0.22		0.65	(0.15,1.15)	0.25		0.25	(-0.22,0.72)	0.24	
	1963-1973; age 50-59	0.55	(0.07,1.04)	0.25		1.19	(0.63,1.75)	0.28		0.93	(0.40,1.47)	0.27	
	1953-1963; age 60-69	0.01	(-0.64,0.66)	0.33		0.89	(0.10,1.68)	0.40		0.97	(0.33,1.60)	0.32	
	1943-1953; age 70-79	0.13	(-1.02,1.28)	0.58		1.05	(-0.29,2.39)	0.68		0.21	(-0.96,1.37)	0.59	

		Cantril's Ladder				Life Satisfaction				Happiness			
Variable	Category	Est	95% CI	SE	Global p-value	Est	95% CI	SE	Global p-value	Est	95% CI	SE	Global p-value
	1943 or earlier; age 80+	-0.08	(-1.78,1.62)	0.87		0.64	(-1.29,2.56)	0.98		1.13	(-0.63,2.88)	0.89	
Gender	(Ref: Male)				0.491				0.051				0.023
	Male	-0.11	(-0.43,0.21)	0.16		-0.35	(-0.71,0.00)	0.18		-0.38	(-0.70,-0.05)	0.17	
Religious affiliation	(Ref: Islam)				0.083				0.477				0.985
	Collapsed affiliations with prevalence<3 %	-0.77	(-1.65,0.10)	0.45		-0.39	(-1.47,0.69)	0.55		-0.01	(-0.94,0.92)	0.47	
Race/ethnicity	(Ref: Plurality group)												

Table S21g. Complete-case supplemental analysis of sensitivity to unmeasured confounding of childhood predictors in Turkey

		Cantril's Ladder		Life Satisfaction		Happiness	
Variable	Category	E-value for Estimate	E-value for 95% CI	E-value for Estimate	E-value for 95% CI	E-value for Estimate	E-value for 95% CI
Relationship with mother	(Ref: Very bad/somewhat bad)						
	Very good/somewhat good	1.42	1.00	1.30	1.00	1.86	1.00
Relationship with father	(Ref: Very bad/somewhat bad)						
	Very good/somewhat good	1.46	1.00	1.35	1.00	1.26	1.00
Parent marital status	(Ref: Parents married)						
	No, one or both of them had died	1.68	1.00	2.20	1.00	1.97	1.00
	No, they were never married	1.42	1.00	4.06	2.16	2.25	1.00
	Yes, married	1.61	1.00	2.38	1.41	2.43	1.46
Subjective financial status of family growing up	(Ref: Got by)						
	Found it difficult	1.29	1.00	1.14	1.00	1.20	1.00
	Found it very difficult	1.58	1.00	1.68	1.00	1.19	1.00
	Lived comfortably	1.67	1.22	1.75	1.35	1.67	1.25
Abuse	(Ref: No)						
	Yes	1.96	1.39	2.00	1.45	1.66	1.00
Outsider growing up	(Ref: No)						
	Yes	1.32	1.00	1.02	1.00	1.52	1.00
Self-rated health growing up	(Ref: Good)						
	Excellent	1.30	1.00	1.27	1.00	1.03	1.00
	Fair	1.40	1.00	1.36	1.00	1.86	1.28
	Poor	1.11	1.00	2.84	1.68	3.37	2.13
	Very good	1.53	1.00	1.45	1.00	1.06	1.00
Immigration status	(Ref: Born in this country)						
	Born in another country	1.21	1.00	1.51	1.00	1.25	1.00
Age 12 religious service attendance	(Ref: Never)						
	At least once a week	1.18	1.00	1.47	1.00	1.69	1.20
	Less than once a month	1.11	1.00	1.30	1.00	1.32	1.00
	One to three times a month	1.34	1.00	1.58	1.00	1.74	1.14
Year of birth	(Ref: 1998-2005; current age: 18-24)						

Variable	Category	Cantril's Ladder		Life Satisfaction		Happiness	
		E-value for Estimate	E-value for 95% CI	E-value for Estimate	E-value for 95% CI	E-value for Estimate	E-value for 95% CI
	1993-1998; age 25-29	1.11	1.00	1.26	1.00	1.40	1.00
	1983-1993; age 30-39	1.36	1.00	1.65	1.00	1.49	1.00
	1973-1983; age 40-49	1.19	1.00	1.80	1.29	1.42	1.00
	1963-1973; age 50-59	1.82	1.20	2.37	1.79	2.18	1.59
	1953-1963; age 60-69	1.07	1.00	2.05	1.23	2.21	1.52
	1943-1953; age 70-79	1.30	1.00	2.22	1.00	1.37	1.00
	1943 or earlier; age 80+	1.22	1.00	1.79	1.00	2.40	1.00
Gender	(Ref: Male)						
	Male	1.27	1.00	1.51	1.00	1.56	1.16
Religious affiliation	(Ref: Islam)						
	Collapsed affiliations with prevalence<3%	2.09	1.00	1.55	1.00	1.06	1.00
Race/ethnicity	(Ref: Plurality group)						

Tables S22a-g: United Kingdom

Table S22a. Nationally representative descriptive statistics for United Kingdom

Characteristic	N = 5,368[1]
**Age group**	
1998-2005; age 18-24	490 (9.1%)
1993-1998; age 25-29	391 (7.3%)
1983-1993; age 30-39	946 (18%)
1973-1983; age 40-49	827 (15%)
1963-1973; age 50-59	949 (18%)
1953-1963; age 60-69	889 (17%)
1943-1953; age 70-79	711 (13%)
1943 or earlier; age 80+	163 (3.0%)
(Missing)	1 (<0.1%)
**Gender**	
Male	2,557 (48%)
Female	2,789 (52%)
Other	14 (0.3%)
(Missing)	9 (0.2%)
**Race/Ethnicity**	
Asian	426 (7.9%)
Black	152 (2.8%)
Other	96 (1.8%)
White	4,647 (87%)
(Missing)	47 (0.9%)
**Respondent Marital status**	
Married	2,510 (47%)
Separated	114 (2.1%)
Divorced	435 (8.1%)
Widowed	294 (5.5%)
Single, never married	1,456 (27%)
Domestic Partner	512 (9.5%)
(Missing)	48 (0.9%)
**Employment**	
Employed for an employer	2,798 (52%)
Self-employed	469 (8.7%)
Retired	1,262 (24%)
Student	229 (4.3%)
Homemaker	184 (3.4%)
Unemployed and looking for a job	215 (4.0%)
None of these/Other	201 (3.7%)
(Missing)	11 (0.2%)
**Religious service attendance as an adult (now)**	
More than 1/week	291 (5.4%)
1/week	499 (9.3%)
1-3/month	293 (5.5%)
A few times a year	1,165 (22%)
Never	3,110 (58%)
(Missing)	10 (0.2%)
**Education (years)**	
Up to 8 years	1,314 (24%)
9-15 years	2,072 (39%)
16+ years	1,974 (37%)
(Missing)	8 (0.2%)
**Immigration status**	
Born in this country	4,659 (87%)
Born in another country	682 (13%)
(Missing)	27 (0.5%)
**Religious affiliation as an adult (now)**	
Christianity	2,750 (51%)
Islam	218 (4.1%)
Hinduism	61 (1.1%)
Buddhism	30 (0.6%)
Judaism	44 (0.8%)
Sikhism	29 (0.5%)

Characteristic	N = 5,368[1]
Baha'i	6 (0.1%)
Jainism	4 (<0.1%)
Shinto	0 (0%)
Taoism	4 (<0.1%)
Confucianism	2 (<0.1%)
Primal, Animist, or Folk religion	36 (0.7%)
Spiritism	0 (0%)
Umbanda, Candomble, and other African-derived religions	0 (0%)
Chinese folk/traditional religion	0 (0%)
Some other religion	61 (1.1%)
No religion/Atheist/Agnostic	2,099 (39%)
(Missing)	25 (0.5%)
**Relationship with mother growing up**	
Very good	3,435 (64%)
Somewhat good	1,338 (25%)
Somewhat bad	325 (6.1%)
Very bad	150 (2.8%)
Does not apply	92 (1.7%)
(Missing)	27 (0.5%)
**Relationship with father growing up**	
Very good	2,907 (54%)
Somewhat good	1,383 (26%)
Somewhat bad	407 (7.6%)
Very bad	321 (6.0%)
Does not apply	321 (6.0%)
(Missing)	29 (0.5%)
**Parent marital status at age 12**	
Parents married	4,343 (81%)
Divorced	481 (9.0%)
Parents were never married	315 (5.9%)
One or both parents had died	154 (2.9%)
(Missing)	75 (1.4%)
**Subjective financial status of family growing up**	
Lived comfortably	2,552 (48%)
Got by	1,933 (36%)
Found it difficult	632 (12%)
Found it very difficult	230 (4.3%)
(Missing)	22 (0.4%)
**Abuse**	
Yes	864 (16%)
No	4,455 (83%)
(Missing)	49 (0.9%)
**Outsider growing up**	
Yes	1,017 (19%)
No	4,308 (80%)
(Missing)	43 (0.8%)
**Self-rated health growing up**	
Excellent	2,154 (40%)
Very good	1,736 (32%)
Good	995 (19%)
Fair	332 (6.2%)
Poor	130 (2.4%)
(Missing)	20 (0.4%)
**Age 12 religious service attendance**	
At least 1/week	1,732 (32%)
1-3/month	733 (14%)
<1/month	903 (17%)
Never	1,972 (37%)
(Missing)	28 (0.5%)
**Religious affiliation at age 12**	
Christianity	3,461 (64%)
Islam	230 (4.3%)
Hinduism	88 (1.6%)
Buddhism	15 (0.3%)
Judaism	59 (1.1%)

Characteristic	N = 5,368[1]
Sikhism	30 (0.6%)
Baha'i	5 (<0.1%)
Jainism	0 (<0.1%)
Shinto	0 (0%)
Taoism	2 (<0.1%)
Confucianism	3 (<0.1%)
Primal, Animist, or Folk religion	22 (0.4%)
Spiritism	0 (0%)
Umbanda, Candomble, and other African-derived religions	0 (0%)
Chinese folk/traditional religion	0 (0%)
Some other religion	24 (0.5%)
No religion/Atheist/Agnostic	1,409 (26%)
(Missing)	21 (0.4%)

[1]n (%)

Table S22b. Means by demographic category for United Kingdom

Variable	Category	Cantril's Ladder				Life Satisfaction				Happiness			
		Mean	95% CI	SE	Global p-value	Mean	95% CI	SE	Global p-value	Mean	95% CI	SE	Global p-value
Age group	18-24	6.32	(6.03,6.60)	0.15	< .001	6.03	(5.66,6.39)	0.18	< .001	6.14	(5.82,6.46)	0.16	< .001
	25-29	6.77	(6.51,7.04)	0.14		6.59	(6.31,6.86)	0.14		6.86	(6.61,7.10)	0.13	
	30-39	6.58	(6.39,6.76)	0.09		6.41	(6.19,6.63)	0.11		6.62	(6.43,6.80)	0.10	
	40-49	6.31	(6.14,6.48)	0.09		6.32	(6.11,6.54)	0.11		6.52	(6.33,6.71)	0.10	
	50-59	6.44	(6.27,6.61)	0.09		6.41	(6.22,6.60)	0.10		6.63	(6.45,6.80)	0.09	
	60-69	6.60	(6.39,6.81)	0.11		6.59	(6.36,6.82)	0.12		6.83	(6.63,7.03)	0.10	
	70-79	6.88	(6.67,7.10)	0.11		6.95	(6.72,7.18)	0.12		7.16	(6.94,7.38)	0.11	
	80 or older	6.89	(6.44,7.33)	0.22		7.04	(6.46,7.63)	0.30		7.34	(6.83,7.85)	0.26	
Gender	Male	6.70	(6.60,6.81)	0.05	0.001	6.63	(6.50,6.76)	0.06	< .001	6.89	(6.78,7.00)	0.06	< .001
	Female	6.43	(6.31,6.54)	0.06		6.38	(6.25,6.50)	0.06		6.55	(6.44,6.66)	0.06	
	Other	5.50	(3.16,7.83)	1.02		4.39	(2.48,6.29)	0.83		4.53	(3.08,5.99)	0.64	
Marital status	Married	6.98	(6.87,7.08)	0.05	< .001	7.06	(6.94,7.17)	0.06	< .001	7.15	(7.05,7.26)	0.05	< .001
	Separated	5.59	(5.16,6.01)	0.21		5.54	(5.10,5.99)	0.22		5.84	(5.40,6.27)	0.22	
	Divorced	5.89	(5.60,6.19)	0.15		5.92	(5.58,6.26)	0.17		6.35	(6.07,6.63)	0.14	
	Widowed	6.71	(6.34,7.08)	0.19		6.44	(6.02,6.85)	0.21		6.68	(6.29,7.07)	0.20	
	Never	6.12	(5.97,6.27)	0.08		5.83	(5.65,6.01)	0.09		6.14	(5.98,6.31)	0.08	
	Domestic Partner	6.43	(6.19,6.66)	0.12		6.36	(6.06,6.67)	0.15		6.61	(6.37,6.84)	0.12	
Employment	Employed for an employer	6.60	(6.50,6.70)	0.05	< .001	6.55	(6.43,6.66)	0.06	< .001	6.74	(6.64,6.85)	0.05	< .001
	Self-employed	6.52	(6.26,6.77)	0.13		6.39	(6.07,6.70)	0.16		6.52	(6.23,6.82)	0.15	
	Retired	6.91	(6.76,7.07)	0.08		6.93	(6.76,7.11)	0.09		7.18	(7.03,7.33)	0.08	
	Student	6.41	(6.10,6.73)	0.16		6.11	(5.71,6.52)	0.20		6.22	(5.87,6.56)	0.18	
	Homemaker	6.70	(6.26,7.14)	0.22		6.63	(6.14,7.12)	0.25		6.80	(6.36,7.24)	0.22	
	Unemployed and looking for a job	5.48	(4.96,6.01)	0.27		5.36	(4.76,5.97)	0.31		5.79	(5.25,6.33)	0.27	
	None of these/Other	4.96	(4.59,5.32)	0.18		4.79	(4.29,5.28)	0.25		5.07	(4.58,5.56)	0.25	
Religious service attendance	More than 1/week	7.59	(7.31,7.86)	0.14	< .001	7.50	(7.22,7.79)	0.15	< .001	7.73	(7.48,7.99)	0.13	< .001
	1/week	7.10	(6.86,7.34)	0.12		7.01	(6.78,7.25)	0.12		7.29	(7.07,7.52)	0.11	
	1-3/month	7.05	(6.69,7.40)	0.18		7.07	(6.66,7.48)	0.21		7.35	(7.02,7.68)	0.17	
	A few times a year	6.62	(6.46,6.77)	0.08		6.66	(6.48,6.83)	0.09		6.77	(6.62,6.93)	0.08	
	Never	6.30	(6.20,6.41)	0.05		6.20	(6.08,6.32)	0.06		6.43	(6.32,6.54)	0.06	
Education	Up to 8 years	6.48	(6.25,6.71)	0.12	< .001	6.46	(6.20,6.72)	0.13	< .001	6.73	(6.50,6.96)	0.12	< .001
	9-15 years	6.41	(6.31,6.51)	0.05		6.34	(6.22,6.46)	0.06		6.55	(6.44,6.66)	0.06	

Variable	Category	Cantril's Ladder				Life Satisfaction				Happiness			
		Mean	95% CI	SE	Global p-value	Mean	95% CI	SE	Global p-value	Mean	95% CI	SE	Global p-value
	16+ years	6.76	(6.67,6.85)	0.05		6.68	(6.57,6.79)	0.06		6.86	(6.76,6.96)	0.05	
Immigration status	Born in this country	6.55	(6.46,6.63)	0.04	0.489	6.49	(6.39,6.58)	0.05	0.601	6.70	(6.61,6.78)	0.04	0.508
	Born in another country	6.62	(6.41,6.84)	0.11		6.55	(6.30,6.80)	0.13		6.78	(6.56,7.00)	0.11	
Religious affiliation	Christianity	6.82	(6.71,6.93)	0.06	< .001	6.82	(6.70,6.94)	0.06	< .001	7.01	(6.90,7.12)	0.06	< .001
	Islam	6.66	(6.28,7.04)	0.19		6.59	(6.15,7.03)	0.22		6.87	(6.50,7.25)	0.19	
	Hinduism	6.91	(6.15,7.67)	0.38		6.66	(5.90,7.42)	0.38		6.80	(6.13,7.46)	0.33	
	Buddhism	6.36	(5.27,7.45)	0.51		7.05	(6.29,7.80)	0.35		7.04	(6.43,7.65)	0.29	
	Judaism	6.50	(5.80,7.21)	0.34		6.60	(5.68,7.51)	0.44		6.70	(5.89,7.52)	0.40	
	Sikhism	7.12	(6.51,7.72)	0.28		6.94	(5.90,7.98)	0.48		7.14	(6.45,7.83)	0.32	
	Baha'i	5.46	*	*		5.29	*	*		5.43	*	*	
	Jainism	6.00	*	*		5.00	*	*		5.00	*	*	
	Taoism	5.35	*	*		4.67	*	*		5.20	*	*	
	Confucianism	6.00	*	*		10.00	*	*		9.00	*	*	
	Primal, Animist, or Folk religion	6.58	(5.97,7.19)	0.29		6.79	(5.98,7.59)	0.39		6.74	(5.82,7.66)	0.44	
	Some other religion	5.90	(5.31,6.49)	0.29		5.81	(5.23,6.38)	0.28		6.27	(5.68,6.85)	0.29	
	No religion/Atheist /Agnostic	6.21	(6.10,6.33)	0.06		6.06	(5.92,6.20)	0.07		6.30	(6.17,6.42)	0.06	
Race/Ethnicity	Asian	6.42	(6.12,6.73)	0.16	0.375	6.42	(6.06,6.78)	0.18	0.665	6.65	(6.34,6.96)	0.16	0.476
	Black	6.39	(5.99,6.80)	0.21		6.39	(5.95,6.83)	0.22		6.98	(6.56,7.41)	0.21	
	White	6.58	(6.50,6.66)	0.04		6.51	(6.42,6.61)	0.05		6.71	(6.62,6.79)	0.04	
	Other	6.21	(5.62,6.80)	0.30		6.15	(5.47,6.84)	0.35		6.50	(5.96,7.04)	0.27	

Table S22c. Childhood predictors regression analysis results for United Kingdom

		Cantril's Ladder				Life Satisfaction				Happiness			
Variable	Category	Est	95% CI	SE	Global p-value	Est	95% CI	SE	Global p-value	Est	95% CI	SE	Global p-value
Relationship with mother	(Ref: Very bad/somewhat bad)				0.064				0.121				0.183
	Very good/somewhat good	0.27	(-0.02, 0.56)	0.15		0.26	(-0.07, 0.59)	0.17		0.19	(-0.09, 0.48)	0.15	
Relationship with father	(Ref: Very bad/somewhat bad)				0.116				0.145				0.047
	Very good/somewhat good	0.17	(-0.05, 0.39)	0.11		0.19	(-0.07, 0.46)	0.14		0.24	(-0.00, 0.48)	0.12	
Parent marital status	(Ref: Parents married)				0.015				0.011				0.013
	Divorced	-0.11	(-0.36, 0.15)	0.13		-0.01	(-0.31, 0.29)	0.15		-0.16	(-0.42, 0.11)	0.14	
	Parents were never married	-0.53	(-0.89, -0.16)	0.19		-0.64	(-1.07, -0.22)	0.22		-0.54	(-0.92, -0.15)	0.19	
	One or both parents had died	-0.44	(-1.02, 0.14)	0.29		-0.48	(-1.05, 0.10)	0.29		-0.53	(-1.10, 0.05)	0.29	
Subjective financial status of family growing up	(Ref: Got by)				0.002				0.007				0.006
	Lived comfortably	0.23	(0.07, 0.40)	0.08		0.07	(-0.11, 0.25)	0.09		0.08	(-0.08, 0.24)	0.08	
	Found it difficult	-0.03	(-0.27, 0.22)	0.13		-0.09	(-0.37, 0.19)	0.14		-0.07	(-0.31, 0.16)	0.12	
	Found it very difficult	-0.44	(-0.88, -0.01)	0.22		-0.90	(-1.45, -0.34)	0.28		-0.82	(-1.33, -0.30)	0.26	
Abuse	(Ref: No)				<.001				<.001				<.001
	Yes	-0.45	(-0.67, -0.23)	0.11		-0.54	(-0.80, -0.27)	0.13		-0.41	(-0.64, -0.18)	0.12	
Outsider growing up	(Ref: No)				0.324				0.086				0.170
	Yes	-0.11	(-0.34, 0.12)	0.12		-0.22	(-0.47, 0.04)	0.13		-0.15	(-0.38, 0.07)	0.11	
Self-rated health growing up	(Ref: Good)				<.001				<.001				<.001
	Excellent	0.37	(0.16, 0.57)	0.10		0.57	(0.32, 0.81)	0.12		0.61	(0.40, 0.82)	0.11	

		Cantril's Ladder				Life Satisfaction				Happiness			
Variable	Category	Est	95% CI	SE	Global p-value	Est	95% CI	SE	Global p-value	Est	95% CI	SE	Global p-value
	Very good	0.33	(0.13, 0.54)	0.10		0.66	(0.43, 0.89)	0.12		0.54	(0.34, 0.73)	0.10	
	Fair	-0.40	(-0.75, -0.05)	0.18		-0.52	(-0.94, -0.11)	0.21		-0.60	(-0.97, -0.22)	0.19	
	Poor	-0.59	(-1.20, 0.02)	0.31		-0.52	(-1.28, 0.25)	0.39		-0.50	(-1.20, 0.21)	0.36	
Immigration status	(Ref: Born in this country)				0.365				0.429				0.424
	Born in another country	0.11	(-0.13, 0.35)	0.12		0.11	(-0.16, 0.38)	0.14		0.10	(-0.15, 0.34)	0.13	
Age 12 religious service attendance	(Ref: Never)				0.004				0.181				0.047
	At least 1/week	0.39	(0.18, 0.60)	0.11		0.25	(0.01, 0.49)	0.12		0.28	(0.06, 0.49)	0.11	
	1-3/month	0.27	(0.03, 0.52)	0.12		0.12	(-0.17, 0.42)	0.15		0.31	(0.06, 0.56)	0.13	
	< 1/month	0.20	(-0.00, 0.41)	0.11		0.21	(-0.04, 0.45)	0.13		0.18	(-0.04, 0.39)	0.11	
Year of birth	(Ref: 1998-2005; current age: 18-24)				0.040				0.061				<.001
	1993-1998; age 25-29	0.28	(-0.09, 0.66)	0.19		0.39	(-0.03, 0.80)	0.21		0.56	(0.18, 0.95)	0.20	
	1983-1993; age 30-39	0.11	(-0.20, 0.41)	0.15		0.22	(-0.16, 0.59)	0.19		0.35	(0.02, 0.68)	0.17	
	1973-1983; age 40-49	-0.17	(-0.47, 0.13)	0.15		0.10	(-0.28, 0.47)	0.19		0.22	(-0.12, 0.56)	0.17	
	1963-1973; age 50-59	-0.08	(-0.38, 0.23)	0.16		0.18	(-0.19, 0.55)	0.19		0.34	(-0.00, 0.68)	0.17	
	1953-1963; age 60-69	0.02	(-0.32, 0.36)	0.17		0.25	(-0.14, 0.64)	0.20		0.46	(0.11, 0.82)	0.18	
	1943-1953; age 70-79	0.20	(-0.15, 0.54)	0.18		0.52	(0.12, 0.91)	0.20		0.70	(0.34, 1.07)	0.19	
	1943 or earlier; age 80+	0.19	(-0.29, 0.68)	0.25		0.64	(0.03, 1.26)	0.31		0.91	(0.38, 1.45)	0.27	
Gender	(Ref: Male)				0.005				0.007				<.001
	Female	-0.24	(-0.39, -0.10)	0.07		-0.23	(-0.40, -0.06)	0.09		-0.30	(-0.45, -0.16)	0.08	
	Other	-0.47	(-2.28, 1.34)	0.92		-1.30	(-2.74, 0.14)	0.73		-1.34	(-2.51, -0.17)	0.60	
Religious affiliation	(Ref: No religion/Atheist /Agnostic)				0.035				0.003				0.012
	Islam	0.21	(-0.25, 0.67)	0.23		0.35	(-0.16, 0.87)	0.26		0.26	(-0.20, 0.73)	0.24	
	Christianity	0.29	(0.10, 0.49)	0.10		0.44	(0.21, 0.68)	0.12		0.33	(0.13, 0.54)	0.10	

		Cantril's Ladder				Life Satisfaction				Happiness			
Variable	Category	Est	95% CI	SE	Global p-value	Est	95% CI	SE	Global p-value	Est	95% CI	SE	Global p-value
Race/ethnicity	Collapsed affiliations with prevalence<3%	0.20	(-0.18, 0.57)	0.19		0.28	(-0.14, 0.71)	0.22		0.13	(-0.22, 0.49)	0.18	
	(Ref: Plurality group)				0.058				0.500				0.694
	Non-plurality groups	-0.30	(-0.66, 0.05)	0.18		-0.11	(-0.46, 0.23)	0.18		0.06	(-0.25, 0.37)	0.16	

Table S22d. Sensitivity to unmeasured confounding of childhood predictors in United Kingdom

		Cantril's Ladder		Life Satisfaction		Happiness	
Variable	Category	E-value for Estimate	E-value for 95% CI	E-value for Estimate	E-value for 95% CI	E-value for Estimate	E-value for 95% CI
Relationship with mother	(Ref: Very bad/somewhat bad)						
	Very good/somewhat good	1.52	1.00	1.45	1.00	1.40	1.00
Relationship with father	(Ref: Very bad/somewhat bad)						
	Very good/somewhat good	1.38	1.00	1.37	1.00	1.46	1.00
Parent marital status	(Ref: Parents married)						
	Divorced	1.27	1.00	1.06	1.00	1.35	1.00
	Parents were never married	1.85	1.36	1.90	1.40	1.84	1.34
	One or both parents had died	1.74	1.00	1.71	1.00	1.83	1.00
Subjective financial status of family growing up	(Ref: Got by)						
	Lived comfortably	1.46	1.22	1.20	1.00	1.23	1.00
	Found it difficult	1.12	1.00	1.23	1.00	1.22	1.00
	Found it very difficult	1.74	1.06	2.20	1.55	2.21	1.54
Abuse	(Ref: No)						
	Yes	1.75	1.46	1.78	1.47	1.68	1.38
Outsider growing up	(Ref: No)						
	Yes	1.29	1.00	1.40	1.00	1.34	1.00
Self-rated health growing up	(Ref: Good)						
	Excellent	1.64	1.36	1.81	1.53	1.93	1.66
	Very good	1.60	1.31	1.92	1.65	1.84	1.59
	Fair	1.69	1.18	1.76	1.26	1.92	1.44
	Poor	1.94	1.00	1.75	1.00	1.79	1.00
Immigration status	(Ref: Born in this country)						
	Born in another country	1.28	1.00	1.26	1.00	1.26	1.00
Age 12 religious service attendance	(Ref: Never)						
	At least 1/week	1.67	1.39	1.44	1.06	1.51	1.19
	1-3/month	1.52	1.14	1.28	1.00	1.55	1.18
	< 1/month	1.42	1.00	1.39	1.00	1.37	1.00
Year of birth	(Ref: 1998-2005; current age: 18-24)						
	1993-1998; age 25-29	1.53	1.00	1.60	1.00	1.87	1.37
	1983-1993; age 30-39	1.28	1.00	1.40	1.00	1.60	1.11
	1973-1983; age 40-49	1.38	1.00	1.24	1.00	1.44	1.00
	1963-1973; age 50-59	1.22	1.00	1.35	1.00	1.59	1.00

Variable	Category	Cantril's Ladder: E-value for Estimate	Cantril's Ladder: E-value for 95% CI	Life Satisfaction: E-value for Estimate	Life Satisfaction: E-value for 95% CI	Happiness: E-value for Estimate	Happiness: E-value for 95% CI
	1953-1963; age 60-69	1.11	1.00	1.44	1.00	1.75	1.28
	1943-1953; age 70-79	1.41	1.00	1.75	1.28	2.06	1.59
	1943 or earlier; age 80+	1.41	1.00	1.90	1.11	2.35	1.64
Gender	(Ref: Male)						
	Female	1.48	1.26	1.42	1.18	1.55	1.35
	Other	1.78	1.00	2.73	1.00	2.99	1.37
Religious affiliation	(Ref: No religion/Atheist/Agnostic)						
	Islam	1.43	1.00	1.57	1.00	1.49	1.00
	Christianity	1.54	1.26	1.67	1.39	1.58	1.31
	Collapsed affiliations with prevalence<3%	1.41	1.00	1.48	1.00	1.31	1.00
Race/ethnicity	(Ref: Plurality group)						
	Non-plurality groups	1.56	1.00	1.26	1.00	1.19	1.00

Table S22e. Complete-case supplemental analysis of means by demographic category for United Kingdom

Variable	Category	Cantril's Ladder: Mean	95% CI	SE	Global p-value	Life Satisfaction: Mean	95% CI	SE	Global p-value	Happiness: Mean	95% CI	SE	Global p-value
Age group	18-24	6.36	(6.08,6.64)	0.14	< .001	6.30	(5.98,6.62)	0.16	< .001	6.25	(5.94,6.56)	0.16	< .001
	25-29	6.80	(6.54,7.07)	0.13		6.68	(6.42,6.94)	0.13		6.91	(6.67,7.15)	0.12	
	30-39	6.62	(6.45,6.80)	0.09		6.51	(6.31,6.72)	0.10		6.71	(6.54,6.87)	0.09	
	40-49	6.40	(6.23,6.56)	0.08		6.53	(6.36,6.71)	0.09		6.66	(6.49,6.83)	0.09	
	50-59	6.52	(6.36,6.68)	0.08		6.56	(6.38,6.74)	0.09		6.71	(6.55,6.88)	0.08	
	60-69	6.67	(6.47,6.87)	0.10		6.75	(6.54,6.95)	0.11		6.93	(6.76,7.11)	0.09	
	70-79	6.94	(6.74,7.14)	0.10		7.07	(6.86,7.28)	0.11		7.22	(7.01,7.43)	0.11	
	80 or older	6.93	(6.48,7.37)	0.23		7.24	(6.77,7.70)	0.23		7.54	(7.21,7.88)	0.17	
Gender	Female	6.51	(6.40,6.61)	0.05	0.004	6.56	(6.45,6.67)	0.06	0.007	6.68	(6.57,6.78)	0.05	< .001
	Male	6.74	(6.64,6.85)	0.05		6.76	(6.64,6.87)	0.06		6.95	(6.85,7.06)	0.05	
	Other	5.50	(3.16,7.83)	1.02		5.26	(3.69,6.83)	0.69		4.90	(3.62,6.18)	0.56	
Marital status	Divorced	6.02	(5.74,6.31)	0.14	< .001	6.18	(5.88,6.49)	0.15	< .001	6.44	(6.16,6.72)	0.14	< .001
	Domestic partner	6.42	(6.19,6.66)	0.12		6.46	(6.17,6.74)	0.15		6.63	(6.40,6.86)	0.12	
	Married	7.06	(6.96,7.15)	0.05		7.16	(7.06,7.26)	0.05		7.22	(7.13,7.32)	0.05	
	Separated	5.67	(5.25,6.08)	0.21		5.65	(5.22,6.08)	0.22		5.89	(5.46,6.32)	0.22	
	Single/Never been married	6.16	(6.01,6.30)	0.07		6.05	(5.89,6.21)	0.08		6.30	(6.14,6.45)	0.08	
	Widowed	6.74	(6.37,7.11)	0.19		6.63	(6.26,7.00)	0.19		6.92	(6.62,7.22)	0.15	
Employment	Employed for an employer	6.65	(6.55,6.74)	0.05	< .001	6.66	(6.55,6.77)	0.06	< .001	6.82	(6.72,6.91)	0.05	< .001
	Homemaker	6.71	(6.26,7.15)	0.22		6.76	(6.29,7.23)	0.24		6.83	(6.39,7.27)	0.22	

		Cantril's Ladder				Life Satisfaction				Happiness			
Variable	Category	Mean	95% CI	SE	Global p-value	Mean	95% CI	SE	Global p-value	Mean	95% CI	SE	Global p-value
	None of these/Other	5.08	(4.73,5.43)	0.18		5.15	(4.68,5.62)	0.24		5.37	(4.92,5.83)	0.23	
	Retired	6.96	(6.82,7.11)	0.08		7.05	(6.89,7.20)	0.08		7.23	(7.09,7.38)	0.07	
	Self-employed	6.65	(6.42,6.87)	0.11		6.67	(6.42,6.92)	0.13		6.75	(6.53,6.98)	0.11	
	Student	6.41	(6.10,6.73)	0.16		6.13	(5.72,6.53)	0.20		6.23	(5.89,6.58)	0.18	
	Unemployed and looking for a job	5.69	(5.20,6.17)	0.25		5.96	(5.49,6.44)	0.24		6.04	(5.55,6.53)	0.25	
Religious service attendance	A few times a year	6.64	(6.49,6.79)	0.08	< .001	6.74	(6.58,6.91)	0.08	< .001	6.84	(6.70,6.99)	0.07	< .001
	More than once a week	7.60	(7.32,7.87)	0.14		7.51	(7.22,7.79)	0.15		7.73	(7.48,7.99)	0.13	
	Never	6.40	(6.30,6.49)	0.05		6.41	(6.31,6.52)	0.05		6.56	(6.46,6.66)	0.05	
	Once a week	7.12	(6.88,7.35)	0.12		7.06	(6.83,7.28)	0.12		7.33	(7.11,7.55)	0.11	
	One to three times a month	7.05	(6.70,7.40)	0.18		7.27	(6.90,7.63)	0.18		7.42	(7.12,7.73)	0.15	
Education	Up to 8 years	6.59	(6.37,6.80)	0.11	< .001	6.70	(6.47,6.93)	0.12	0.002	6.89	(6.68,7.09)	0.10	< .001
	16+ years	6.81	(6.72,6.90)	0.05		6.78	(6.67,6.88)	0.05		6.92	(6.83,7.01)	0.05	
	9 to 15 years	6.46	(6.36,6.56)	0.05		6.50	(6.39,6.62)	0.06		6.64	(6.54,6.75)	0.05	
Immigration status	Born in another country	6.68	(6.47,6.89)	0.11	0.558	6.69	(6.45,6.93)	0.12	0.742	6.82	(6.60,7.04)	0.11	0.857
	Born in this country	6.61	(6.53,6.69)	0.04		6.65	(6.56,6.73)	0.04		6.80	(6.72,6.88)	0.04	
Religious affiliation	Buddhism	6.19	(5.35,7.04)	0.41	< .001	6.75	(6.01,7.50)	0.36	< .001	6.92	(6.37,7.46)	0.26	< .001
	Christianity	6.88	(6.78,6.99)	0.05		6.95	(6.84,7.06)	0.06		7.10	(7.00,7.20)	0.05	
	Confucianism	6.00	*	*		10.00	*	*		9.00	*	*	
	Hinduism	6.91	(6.15,7.67)	0.38		6.75	(6.01,7.50)	0.37		6.85	(6.19,7.51)	0.33	
	Islam	6.78	(6.41,7.14)	0.19		6.86	(6.48,7.24)	0.19		7.00	(6.64,7.36)	0.18	
	Judaism	6.50	(5.79,7.20)	0.35		6.49	(5.68,7.30)	0.39		6.70	(5.89,7.52)	0.40	
	No religion/Atheist/Agnostic	6.27	(6.16,6.39)	0.06		6.25	(6.13,6.38)	0.06		6.41	(6.29,6.52)	0.06	
	Primal, Animist, or Folk religion	6.56	(5.98,7.15)	0.28		6.79	(5.98,7.59)	0.39		6.74	(5.82,7.66)	0.44	
	Sikhism	7.13	(6.53,7.73)	0.28		6.94	(5.90,7.98)	0.48		7.14	(6.45,7.83)	0.32	

		Cantril's Ladder				Life Satisfaction				Happiness			
Variable	Category	Mean	95% CI	SE	Global p-value	Mean	95% CI	SE	Global p-value	Mean	95% CI	SE	Global p-value
	Some other religion	6.09	(5.55,6.63)	0.27		5.98	(5.45,6.52)	0.26		6.26	(5.70,6.83)	0.28	
	Taoism	5.35	*	*		6.85	*	*		7.55	*	*	
	Baha'i	5.46	*	*		5.29	*	*		5.43	*	*	
	Jainism	6.00	*	*		5.00	*	*		5.00	*	*	

Table S22f. Complete-case supplemental analysis of childhood predictors regression analysis results for United Kingdom

		Cantril's Ladder				Life Satisfaction				Happiness			
Variable	Category	Est	95% CI	SE	Global p-value	Est	95% CI	SE	Global p-value	Est	95% CI	SE	Global p-value
Relationship with mother	(Ref: Very bad/somewhat bad)				0.121				0.330				0.194
	Very good/somewhat good	0.20	(-0.05,0.45)	0.13		0.14	(-0.14,0.41)	0.14		0.17	(-0.09,0.42)	0.13	
Relationship with father	(Ref: Very bad/somewhat bad)				0.095				0.110				0.086
	Very good/somewhat good	0.18	(-0.03,0.39)	0.11		0.19	(-0.04,0.43)	0.12		0.18	(-0.03,0.39)	0.11	
Parent marital status	(Ref: Parents married)				0.052				0.028				0.018
	No, one or both of them had died	-0.11	(-0.67,0.44)	0.28		-0.38	(-0.98,0.22)	0.31		-0.07	(-0.54,0.40)	0.24	
	No, they were never married	-0.29	(-0.68,0.10)	0.20		-0.42	(-0.85,0.02)	0.22		-0.30	(-0.70,0.11)	0.21	
	Yes, married	0.16	(-0.08,0.40)	0.12		0.09	(-0.19,0.37)	0.14		0.20	(-0.04,0.45)	0.13	
Subjective financial status of family growing up	(Ref: Got by)				<.001				0.012				0.004
	Found it difficult	0.03	(-0.21,0.27)	0.12		-0.14	(-0.39,0.12)	0.13		-0.04	(-0.26,0.18)	0.11	
	Found it very difficult	-0.42	(-0.85,0.02)	0.22		-0.65	(-1.10,-0.20)	0.23		-0.57	(-0.96,-0.19)	0.20	
	Lived comfortably	0.26	(0.11,0.41)	0.08		0.07	(-0.10,0.24)	0.09		0.12	(-0.03,0.27)	0.08	
Abuse	(Ref: No)				<.001				<.001				<.001
	Yes	-0.48	(-0.70,-0.27)	0.11		-0.48	(-0.72,-0.24)	0.12		-0.37	(-0.58,-0.16)	0.11	
Outsider growing up	(Ref: No)				0.859				0.399				0.163
	Yes	-0.02	(-0.21,0.18)	0.10		-0.10	(-0.32,0.13)	0.11		-0.14	(-0.34,0.06)	0.10	

Variable	Category	Cantril's Ladder				Life Satisfaction				Happiness			
		Est	95% CI	SE	Global p-value	Est	95% CI	SE	Global p-value	Est	95% CI	SE	Global p-value
Self-rated health growing up	(Ref: Good)				<.001				<.001				<.001
	Excellent	0.38	(0.18,0.58)	0.10		0.58	(0.36,0.80)	0.11		0.62	(0.43,0.81)	0.10	
	Fair	-0.41	(-0.75,-0.07)	0.17		-0.54	(-0.91,-0.16)	0.19		-0.63	(-0.98,-0.28)	0.18	
	Poor	-0.52	(-1.14,0.10)	0.32		-0.17	(-0.76,0.41)	0.30		-0.19	(-0.71,0.34)	0.27	
	Very good	0.34	(0.15,0.54)	0.10		0.57	(0.35,0.78)	0.11		0.49	(0.31,0.68)	0.09	
Immigration status	(Ref: Born in this country)				0.410				0.616				0.799
	Born in another country	0.10	(-0.13,0.32)	0.12		0.07	(-0.19,0.32)	0.13		0.03	(-0.21,0.27)	0.12	
Age 12 religious service attendance	(Ref: Never)				0.009				0.255				0.119
	At least once a week	0.33	(0.14,0.52)	0.10		0.21	(-0.01,0.42)	0.11		0.21	(0.02,0.40)	0.10	
	Less than once a month	0.10	(-0.10,0.31)	0.10		0.12	(-0.10,0.34)	0.11		0.05	(-0.14,0.25)	0.10	
	One to three times a month	0.18	(-0.05,0.41)	0.12		0.05	(-0.21,0.31)	0.13		0.20	(-0.04,0.43)	0.12	
Year of birth	(Ref: 1998-2005; current age: 18-24)				0.080				0.027				<.001
	1993-1998; age 25-29	0.27	(-0.10,0.64)	0.19		0.29	(-0.10,0.69)	0.20		0.53	(0.15,0.90)	0.19	
	1983-1993; age 30-39	0.12	(-0.18,0.41)	0.15		0.13	(-0.22,0.47)	0.18		0.34	(0.02,0.65)	0.16	
	1973-1983; age 40-49	-0.11	(-0.40,0.19)	0.15		0.13	(-0.21,0.47)	0.17		0.28	(-0.03,0.60)	0.16	
	1963-1973; age 50-59	-0.02	(-0.31,0.28)	0.15		0.16	(-0.18,0.51)	0.18		0.34	(0.01,0.67)	0.17	
	1953-1963; age 60-69	0.08	(-0.24,0.41)	0.17		0.25	(-0.11,0.62)	0.19		0.49	(0.15,0.82)	0.17	
	1943-1953; age 70-79	0.25	(-0.08,0.58)	0.17		0.51	(0.15,0.88)	0.19		0.69	(0.34,1.04)	0.18	

		Cantril’s Ladder				Life Satisfaction				Happiness			
Variable	Category	Est	95% CI	SE	Global p-value	Est	95% CI	SE	Global p-value	Est	95% CI	SE	Global p-value
	1943 or earlier; age 80+	0.23	(-0.25,0.72)	0.25		0.67	(0.13,1.22)	0.28		1.02	(0.58,1.45)	0.22	
Gender	(Ref: Male)				0.012				0.037				<.001
	Male	0.21	(0.07,0.35)	0.07		0.18	(0.03,0.34)	0.08		0.26	(0.13,0.40)	0.07	
	Other	-0.38	(-2.19,1.43)	0.92		-0.59	(-1.80,0.62)	0.62		-0.81	(-1.81,0.18)	0.51	
Religious affiliation	(Ref: No religion/Atheist/Agnostic)				0.025				0.002				0.003
	Christianity	0.29	(0.10,0.47)	0.09		0.38	(0.18,0.58)	0.10		0.34	(0.16,0.52)	0.09	
	Christianity	0.29	(0.10,0.47)	0.09		0.38	(0.18,0.58)	0.10		0.34	(0.16,0.52)	0.09	
	Islam	0.24	(-0.17,0.65)	0.21		0.33	(-0.13,0.79)	0.23		0.31	(-0.11,0.73)	0.21	
	Collapsed affiliations with prevalence<3%	0.19	(-0.16,0.54)	0.18		0.10	(-0.31,0.50)	0.21		0.12	(-0.22,0.47)	0.17	
Race/ethnicity	(Ref: Plurality group)				0.108				0.693				0.279
	Non-plurality groups	-0.21	(-0.47,0.05)	0.13		0.06	(-0.23,0.34)	0.14		0.14	(-0.12,0.41)	0.13	

Table S22g. Complete-case supplemental analysis of sensitivity to unmeasured confounding of childhood predictors in United Kingdom

		Cantril's Ladder		Life Satisfaction		Happiness	
Variable	Category	E-value for Estimate	E-value for 95% CI	E-value for Estimate	E-value for 95% CI	E-value for Estimate	E-value for 95% CI
Relationship with mother	(Ref: Very bad/somewhat bad)						
	Very good/somewhat good	1.43	1.00	1.32	1.00	1.38	1.00
Relationship with father	(Ref: Very bad/somewhat bad)						
	Very good/somewhat good	1.40	1.00	1.40	1.00	1.40	1.00
Parent marital status	(Ref: Parents married)						
	No, one or both of them had died	1.30	1.00	1.64	1.00	1.22	1.00
	No, they were never married	1.56	1.00	1.69	1.00	1.56	1.00
	Yes, married	1.37	1.00	1.25	1.00	1.43	1.00
Subjective financial status of family growing up	(Ref: Got by)						
	Found it difficult	1.13	1.00	1.32	1.00	1.16	1.00
	Found it very difficult	1.74	1.00	1.99	1.41	1.95	1.41
	Lived comfortably	1.52	1.29	1.21	1.00	1.30	1.00
Abuse	(Ref: No)						
	Yes	1.83	1.54	1.77	1.47	1.66	1.36
Outsider growing up	(Ref: No)						
	Yes	1.10	1.00	1.25	1.00	1.34	1.00
Self-rated health growing up	(Ref: Good)						
	Excellent	1.68	1.40	1.89	1.61	2.01	1.74
	Fair	1.73	1.23	1.84	1.36	2.03	1.55
	Poor	1.88	1.00	1.37	1.00	1.41	1.00
	Very good	1.63	1.35	1.88	1.61	1.84	1.59
Immigration status	(Ref: Born in this country)						
	Born in another country	1.27	1.00	1.20	1.00	1.14	1.00
Age 12 religious service attendance	(Ref: Never)						
	At least once a week	1.62	1.33	1.41	1.00	1.44	1.11
	Less than once a month	1.28	1.00	1.29	1.00	1.19	1.00
	One to three times a month	1.40	1.00	1.17	1.00	1.42	1.00
Year of birth	(Ref: 1998-2005; current age: 18-24)						

		Cantril's Ladder		Life Satisfaction		Happiness	
Variable	Category	E-value for Estimate	E-value for 95% CI	E-value for Estimate	E-value for 95% CI	E-value for Estimate	E-value for 95% CI
	1993-1998; age 25-29	1.53	1.00	1.53	1.00	1.88	1.36
	1983-1993; age 30-39	1.30	1.00	1.30	1.00	1.62	1.12
	1973-1983; age 40-49	1.29	1.00	1.31	1.00	1.55	1.00
	1963-1973; age 50-59	1.09	1.00	1.35	1.00	1.63	1.09
	1953-1963; age 60-69	1.25	1.00	1.48	1.00	1.83	1.36
	1943-1953; age 70-79	1.50	1.00	1.81	1.33	2.12	1.63
	1943 or earlier; age 80+	1.48	1.00	2.02	1.30	2.61	1.96
Gender	(Ref: Male)						
	Male	1.44	1.22	1.38	1.13	1.52	1.31
	Other	1.68	1.00	1.91	1.00	2.29	1.00
Religious affiliation	(Ref: No religion/Atheist/Agnostic)						
	Christianity	1.55	1.28	1.64	1.38	1.62	1.37
	Christianity	1.55	1.28	1.64	1.38	1.62	1.37
	Islam	1.49	1.00	1.57	1.00	1.58	1.00
	Collapsed affiliations with prevalence<3%	1.42	1.00	1.25	1.00	1.31	1.00
Race/ethnicity	(Ref: Plurality group)						
	Non-plurality groups	1.45	1.00	1.19	1.00	1.34	1.00

Tables S23a-g: United States

Table S23a. Nationally representative descriptive statistics for United States

Characteristic	N = 38,312[1]
**Age group**	
1998-2005; age 18-24	2,682 (7.0%)
1993-1998; age 25-29	3,540 (9.2%)
1983-1993; age 30-39	7,284 (19%)
1973-1983; age 40-49	5,649 (15%)
1963-1973; age 50-59	6,745 (18%)
1953-1963; age 60-69	6,832 (18%)
1943-1953; age 70-79	4,054 (11%)
1943 or earlier; age 80+	1,525 (4.0%)
(Missing)	0 (0%)
**Gender**	
Male	18,222 (48%)
Female	19,562 (51%)
Other	392 (1.0%)
(Missing)	136 (0.4%)
**Race/Ethnicity**	
Asian	2,466 (6.4%)
Black	4,501 (12%)
Hispanic	6,724 (18%)
Other	997 (2.6%)
White	23,605 (62%)
(Missing)	20 (<0.1%)
**Respondent Marital status**	
Married	20,360 (53%)
Separated	727 (1.9%)
Divorced	3,636 (9.5%)
Widowed	1,978 (5.2%)
Single, never married	9,431 (25%)
Domestic Partner	1,971 (5.1%)
(Missing)	207 (0.5%)
**Employment**	
Employed for an employer	19,502 (51%)
Self-employed	3,445 (9.0%)
Retired	9,016 (24%)
Student	1,145 (3.0%)
Homemaker	2,049 (5.3%)
Unemployed and looking for a job	1,777 (4.6%)
None of these/Other	1,292 (3.4%)
(Missing)	87 (0.2%)
**Religious service attendance as an adult (now)**	
More than 1/week	2,633 (6.9%)
1/week	5,887 (15%)
1-3/month	2,819 (7.4%)
A few times a year	8,870 (23%)
Never	17,975 (47%)
(Missing)	128 (0.3%)
**Education (years)**	
Up to 8 years	210 (0.5%)
9-15 years	25,322 (66%)
16+ years	12,705 (33%)
(Missing)	75 (0.2%)
**Immigration status**	
Born in this country	34,865 (91%)
Born in another country	3,020 (7.9%)
(Missing)	427 (1.1%)
**Religious affiliation as an adult (now)**	
Christianity	22,954 (60%)
Islam	205 (0.5%)
Hinduism	167 (0.4%)
Buddhism	336 (0.9%)
Judaism	638 (1.7%)

Characteristic	N = 38,312[1]
Sikhism	24 (<0.1%)
Baha'i	13 (<0.1%)
Jainism	18 (<0.1%)
Shinto	12 (<0.1%)
Taoism	93 (0.2%)
Confucianism	8 (<0.1%)
Primal, Animist, or Folk religion	240 (0.6%)
Spiritism	0 (0%)
Umbanda, Candomble, and other African-derived religions	0 (0%)
Chinese folk/traditional religion	0 (0%)
Some other religion	1,267 (3.3%)
No religion/Atheist/Agnostic	11,870 (31%)
(Missing)	467 (1.2%)
**Relationship with mother growing up**	
Very good	20,590 (54%)
Somewhat good	11,525 (30%)
Somewhat bad	3,523 (9.2%)
Very bad	1,874 (4.9%)
Does not apply	694 (1.8%)
(Missing)	106 (0.3%)
**Relationship with father growing up**	
Very good	15,313 (40%)
Somewhat good	12,665 (33%)
Somewhat bad	4,879 (13%)
Very bad	2,604 (6.8%)
Does not apply	2,811 (7.3%)
(Missing)	38 (0.1%)
**Parent marital status at age 12**	
Parents married	27,415 (72%)
Divorced	6,325 (17%)
Parents were never married	3,048 (8.0%)
One or both parents had died	1,024 (2.7%)
(Missing)	500 (1.3%)
**Subjective financial status of family growing up**	
Lived comfortably	15,116 (39%)
Got by	15,682 (41%)
Found it difficult	5,152 (13%)
Found it very difficult	2,342 (6.1%)
(Missing)	19 (<0.1%)
**Abuse**	
Yes	10,026 (26%)
No	28,045 (73%)
(Missing)	242 (0.6%)
**Outsider growing up**	
Yes	10,185 (27%)
No	27,714 (72%)
(Missing)	413 (1.1%)
**Self-rated health growing up**	
Excellent	16,866 (44%)
Very good	12,108 (32%)
Good	6,444 (17%)
Fair	2,303 (6.0%)
Poor	520 (1.4%)
(Missing)	71 (0.2%)
**Age 12 religious service attendance**	
At least 1/week	18,609 (49%)
1-3/month	6,644 (17%)
<1/month	5,829 (15%)
Never	7,085 (18%)
(Missing)	145 (0.4%)
**Religious affiliation at age 12**	
Christianity	30,444 (79%)
Islam	220 (0.6%)
Hinduism	203 (0.5%)
Buddhism	172 (0.4%)

Characteristic	N = 38,312[1]
Judaism	787 (2.1%)
Sikhism	47 (0.1%)
Baha'i	4 (<0.1%)
Jainism	18 (<0.1%)
Shinto	6 (<0.1%)
Taoism	17 (<0.1%)
Confucianism	8 (<0.1%)
Primal, Animist, or Folk religion	67 (0.2%)
Spiritism	0 (0%)
Umbanda, Candomble, and other African-derived religions	0 (0%)
Chinese folk/traditional religion	0 (0%)
Some other religion	359 (0.9%)
No religion/Atheist/Agnostic	5,845 (15%)
(Missing)	115 (0.3%)

[1] n (%)

Table S23b. Means by demographic category for United States

		Cantril's Ladder				Life Satisfaction				Happiness			
Variable	Category	Mean	95% CI	SE	Global p-value	Mean	95% CI	SE	Global p-value	Mean	95% CI	SE	Global p-value
Age group	18-24	6.18	(5.85,6.50)	0.16	< .001	6.13	(5.77,6.50)	0.19	< .001	6.24	(5.92,6.56)	0.16	< .001
	25-29	6.18	(5.95,6.41)	0.12		5.91	(5.63,6.18)	0.14		6.11	(5.83,6.40)	0.14	
	30-39	6.51	(6.39,6.62)	0.06		6.40	(6.26,6.54)	0.07		6.61	(6.48,6.73)	0.06	
	40-49	6.70	(6.58,6.83)	0.06		6.61	(6.48,6.75)	0.07		6.75	(6.64,6.85)	0.05	
	50-59	7.04	(6.97,7.12)	0.04		7.04	(6.95,7.12)	0.04		7.19	(7.12,7.27)	0.04	
	60-69	7.45	(7.39,7.50)	0.03		7.42	(7.36,7.48)	0.03		7.55	(7.49,7.60)	0.03	
	70-79	7.79	(7.72,7.86)	0.04		7.73	(7.66,7.80)	0.04		7.85	(7.79,7.91)	0.03	
	80 or older	7.90	(7.65,8.14)	0.13		7.73	(7.39,8.07)	0.17		7.89	(7.62,8.17)	0.14	
Gender	Male	6.92	(6.85,7.00)	0.04	< .001	6.86	(6.78,6.94)	0.04	< .001	7.02	(6.95,7.09)	0.04	0.049
	Female	6.96	(6.89,7.04)	0.04		6.88	(6.79,6.96)	0.04		7.02	(6.95,7.10)	0.04	
	Other	6.08	(5.70,6.46)	0.19		5.64	(5.14,6.14)	0.26		5.96	(5.11,6.81)	0.43	
Marital status	Married	7.40	(7.35,7.45)	0.02	< .001	7.39	(7.34,7.44)	0.03	< .001	7.45	(7.40,7.50)	0.02	< .001
	Separated	5.89	(5.31,6.47)	0.30		5.51	(4.84,6.18)	0.34		6.10	(5.60,6.60)	0.25	
	Divorced	6.71	(6.60,6.82)	0.06		6.73	(6.61,6.84)	0.06		6.97	(6.87,7.08)	0.05	
	Widowed	7.35	(7.16,7.55)	0.10		7.16	(6.90,7.42)	0.13		7.42	(7.21,7.63)	0.11	
	Never	6.10	(5.96,6.23)	0.07		5.92	(5.77,6.07)	0.08		6.19	(6.05,6.33)	0.07	
	Domestic Partner	6.54	(6.33,6.74)	0.11		6.22	(5.90,6.53)	0.16		6.37	(6.08,6.66)	0.15	
Employment	Employed for an employer	6.88	(6.83,6.94)	0.03	< .001	6.79	(6.72,6.86)	0.03	< .001	6.93	(6.87,7.00)	0.03	< .001
	Self-employed	6.95	(6.74,7.15)	0.10		7.02	(6.86,7.19)	0.08		7.19	(7.06,7.32)	0.07	
	Retired	7.66	(7.60,7.72)	0.03		7.60	(7.52,7.68)	0.04		7.70	(7.63,7.77)	0.03	
	Student	6.11	(5.70,6.53)	0.21		5.75	(5.16,6.34)	0.30		6.01	(5.64,6.39)	0.19	
	Homemaker	6.91	(6.69,7.14)	0.12		6.81	(6.56,7.07)	0.13		6.93	(6.71,7.16)	0.11	
	Unemployed and looking for a job	5.33	(4.89,5.77)	0.22		5.09	(4.60,5.59)	0.25		5.34	(4.94,5.75)	0.21	
	None of these/Other	5.60	(5.32,5.89)	0.15		5.70	(5.21,6.19)	0.25		6.18	(5.71,6.66)	0.24	
Religious service attendance	More than 1/week	7.71	(7.57,7.86)	0.08	< .001	7.85	(7.72,7.98)	0.07	< .001	7.98	(7.85,8.11)	0.07	< .001
	1/week	7.53	(7.44,7.62)	0.04		7.65	(7.56,7.74)	0.04		7.70	(7.62,7.78)	0.04	
	1-3/month	7.11	(6.88,7.33)	0.12		7.17	(6.99,7.35)	0.09		7.33	(7.19,7.47)	0.07	
	A few times a year	7.10	(7.02,7.18)	0.04		7.05	(6.95,7.14)	0.05		7.20	(7.10,7.29)	0.05	
	Never	6.52	(6.44,6.60)	0.04		6.31	(6.21,6.40)	0.05		6.50	(6.42,6.58)	0.04	
Education	Up to 8 years	7.10	(6.27,7.94)	0.42	< .001	6.81	(5.56,8.05)	0.63	< .001	6.72	(5.98,7.46)	0.37	< .001
	9-15 years	6.72	(6.65,6.80)	0.04		6.68	(6.60,6.77)	0.04		6.90	(6.82,6.97)	0.04	

		Cantril’s Ladder				Life Satisfaction				Happiness			
Variable	Category	Mean	95% CI	SE	Global p-value	Mean	95% CI	SE	Global p-value	Mean	95% CI	SE	Global p-value
	16+ years	7.35	(7.32,7.39)	0.02		7.20	(7.16,7.24)	0.02		7.24	(7.20,7.27)	0.02	
Immigration status	Born in this country	6.92	(6.87,6.97)	0.03	0.063	6.84	(6.78,6.90)	0.03	0.063	7.00	(6.94,7.05)	0.03	0.130
	Born in another country	7.11	(6.92,7.31)	0.10		7.05	(6.83,7.27)	0.11		7.15	(6.96,7.35)	0.10	
Religious affiliation	Christianity	7.22	(7.16,7.28)	0.03	< .001	7.22	(7.16,7.29)	0.03	< .001	7.37	(7.31,7.43)	0.03	< .001
	Islam	6.62	(6.01,7.23)	0.31		5.90	(4.98,6.83)	0.47		6.11	(5.34,6.87)	0.39	
	Hinduism	7.31	(7.03,7.60)	0.15		7.22	(6.79,7.66)	0.22		7.29	(6.89,7.69)	0.20	
	Buddhism	7.02	(6.64,7.41)	0.20		6.71	(6.15,7.27)	0.28		7.03	(6.64,7.41)	0.20	
	Judaism	7.38	(7.18,7.58)	0.10		7.27	(7.05,7.48)	0.11		7.21	(7.00,7.43)	0.11	
	Sikhism	7.18	(6.18,8.19)	0.43		7.31	(6.36,8.27)	0.41		7.55	(6.60,8.51)	0.41	
	Baha'i	7.50	(1.98,10.0ǂ)	0.75		6.96	(0.81,10.0ǂ)	0.85		7.39	(3.25,10.0ǂ)	0.56	
	Jainism	5.15	(2.06,8.23)	0.81		6.33	(5.12,7.55)	0.32		5.14	(2.01,8.28)	0.82	
	Shinto	8.60	(7.13,10.0ǂ)	0.67		8.49	(6.80,10.0ǂ)	0.77		8.59	(7.05,10.0ǂ)	0.70	
	Taoism	5.06	(4.11,6.00)	0.47		4.39	(2.94,5.83)	0.72		6.39	(5.51,7.27)	0.44	
	Confucianism	6.78	*	*		7.25	*	*		7.33	*	*	
	Primal, Animist, or Folk religion	5.07	(4.15,5.98)	0.46		4.81	(3.57,6.04)	0.63		5.77	(5.09,6.45)	0.35	
	Some other religion	6.10	(5.67,6.53)	0.22		6.01	(5.66,6.35)	0.18		6.35	(5.98,6.71)	0.18	
	No religion/Atheist /Agnostic	6.50	(6.40,6.59)	0.05		6.28	(6.18,6.39)	0.05		6.40	(6.30,6.51)	0.05	
Race/Ethnicity	Asian	7.02	(6.81,7.22)	0.10	< .001	6.84	(6.60,7.07)	0.12	< .001	6.95	(6.76,7.13)	0.10	< .001
	Black	6.71	(6.54,6.88)	0.09		6.64	(6.44,6.85)	0.11		6.84	(6.63,7.04)	0.10	
	White	7.11	(7.07,7.16)	0.02		6.99	(6.93,7.04)	0.03		7.12	(7.07,7.16)	0.02	
	Other	6.92	(6.72,7.12)	0.10		6.74	(6.51,6.96)	0.11		6.98	(6.77,7.19)	0.11	
	Hispanic	6.44	(6.26,6.63)	0.09		6.56	(6.35,6.78)	0.11		6.78	(6.59,6.96)	0.09	

Table S23c. Childhood predictors regression analysis results for United States

		Cantril's Ladder				Life Satisfaction				Happiness			
Variable	Category	Est	95% CI	SE	Global p-value	Est	95% CI	SE	Global p-value	Est	95% CI	SE	Global p-value
Relationship with mother	(Ref: Very bad/somewhat bad)				0.038				0.076				0.004
	Very good/somewhat good	0.18	(0.01, 0.34)	0.09		0.17	(-0.02, 0.36)	0.10		0.24	(0.08, 0.41)	0.08	
Relationship with father	(Ref: Very bad/somewhat bad)				0.320				0.025				0.002
	Very good/somewhat good	0.06	(-0.07, 0.19)	0.07		0.18	(0.02, 0.33)	0.08		0.21	(0.07, 0.34)	0.07	
Parent marital status	(Ref: Parents married)				0.303				0.491				0.893
	Divorced	0.02	(-0.13, 0.17)	0.08		-0.02	(-0.19, 0.15)	0.09		0.05	(-0.10, 0.19)	0.07	
	Parents were never married	-0.19	(-0.45, 0.06)	0.13		-0.17	(-0.51, 0.18)	0.18		-0.02	(-0.33, 0.29)	0.16	
	One or both parents had died	-0.20	(-0.60, 0.19)	0.20		-0.27	(-0.65, 0.12)	0.20		0.05	(-0.29, 0.38)	0.17	
Subjective financial status of family growing up	(Ref: Got by)				<.001				<.001				<.001
	Lived comfortably	0.18	(0.09, 0.27)	0.05		0.20	(0.08, 0.32)	0.06		0.16	(0.05, 0.26)	0.05	
	Found it difficult	-0.03	(-0.17, 0.12)	0.07		0.22	(0.06, 0.38)	0.08		0.20	(0.06, 0.34)	0.07	
	Found it very difficult	-0.16	(-0.40, 0.09)	0.12		-0.09	(-0.36, 0.18)	0.14		-0.20	(-0.47, 0.06)	0.13	
Abuse	(Ref: No)				<.001				<.001				<.001
	Yes	-0.31	(-0.43, -0.19)	0.06		-0.40	(-0.55, -0.26)	0.07		-0.31	(-0.44, -0.19)	0.06	
Outsider growing up	(Ref: No)				<.001				<.001				<.001
	Yes	-0.36	(-0.49, -0.23)	0.07		-0.54	(-0.70, -0.38)	0.08		-0.49	(-0.62, -0.36)	0.07	
Self-rated health growing up	(Ref: Good)				<.001				<.001				<.001
	Excellent	0.72	(0.58, 0.86)	0.07		0.77	(0.59, 0.94)	0.09		0.78	(0.62, 0.94)	0.08	

Variable	Category	Cantril's Ladder				Life Satisfaction				Happiness			
		Est	95% CI	SE	Global p-value	Est	95% CI	SE	Global p-value	Est	95% CI	SE	Global p-value
	Very good	0.40	(0.25, 0.54)	0.07		0.38	(0.20, 0.56)	0.09		0.41	(0.25, 0.57)	0.08	
	Fair	-0.20	(-0.48, 0.08)	0.14		-0.19	(-0.55, 0.17)	0.19		-0.14	(-0.43, 0.15)	0.15	
	Poor	-0.44	(-1.08, 0.21)	0.33		-0.53	(-1.30, 0.23)	0.39		-0.43	(-1.11, 0.26)	0.35	
Immigration status	(Ref: Born in this country)				0.001				0.022				0.068
	Born in another country	0.31	(0.12, 0.51)	0.10		0.25	(0.03, 0.47)	0.11		0.18	(-0.01, 0.37)	0.10	
Age 12 religious service attendance	(Ref: Never)				0.011				0.043				0.142
	At least 1/week	0.22	(0.07, 0.36)	0.08		0.18	(-0.01, 0.37)	0.10		0.17	(-0.00, 0.35)	0.09	
	1-3/month	0.24	(0.08, 0.40)	0.08		0.22	(0.02, 0.43)	0.10		0.11	(-0.08, 0.29)	0.10	
	< 1/month	0.11	(-0.07, 0.28)	0.09		0.03	(-0.18, 0.24)	0.11		0.06	(-0.13, 0.25)	0.10	
Year of birth	(Ref: 1998-2005; current age: 18-24)				<.001				<.001				<.001
	1993-1998; age 25-29	0.05	(-0.30, 0.40)	0.18		-0.17	(-0.58, 0.24)	0.21		-0.08	(-0.44, 0.28)	0.19	
	1983-1993; age 30-39	0.27	(-0.04, 0.58)	0.16		0.20	(-0.15, 0.55)	0.18		0.31	(0.02, 0.61)	0.15	
	1973-1983; age 40-49	0.38	(0.07, 0.70)	0.16		0.30	(-0.05, 0.66)	0.18		0.35	(0.07, 0.64)	0.15	
	1963-1973; age 50-59	0.57	(0.28, 0.87)	0.15		0.57	(0.23, 0.90)	0.17		0.65	(0.38, 0.93)	0.14	
	1953-1963; age 60-69	0.88	(0.58, 1.17)	0.15		0.85	(0.52, 1.18)	0.17		0.91	(0.64, 1.19)	0.14	
	1943-1953; age 70-79	1.18	(0.88, 1.48)	0.15		1.10	(0.77, 1.44)	0.17		1.17	(0.89, 1.45)	0.14	
	1943 or earlier; age 80+	1.26	(0.88, 1.63)	0.19		1.06	(0.59, 1.52)	0.24		1.17	(0.79, 1.55)	0.19	
Gender	(Ref: Male)				0.001				<.001				0.007
	Female	0.15	(0.06, 0.23)	0.04		0.16	(0.06, 0.26)	0.05		0.14	(0.05, 0.22)	0.04	
	Other	-0.11	(-0.44, 0.22)	0.17		-0.40	(-0.86, 0.06)	0.23		-0.24	(-1.06, 0.58)	0.42	
Religious affiliation	(Ref: No religion/Atheist /Agnostic)				0.085				0.050				0.052
	Christianity	0.16	(-0.01, 0.32)	0.09		0.24	(0.02, 0.45)	0.11		0.24	(0.05, 0.43)	0.10	

		Cantril's Ladder				Life Satisfaction				Happiness			
Variable	Category	Est	95% CI	SE	Global p-value	Est	95% CI	SE	Global p-value	Est	95% CI	SE	Global p-value
	Collapsed affiliations with prevalence<3%	0.00	(-0.25, 0.25)	0.13		0.09	(-0.19, 0.36)	0.14		0.19	(-0.04, 0.43)	0.12	
Race/ethnicity	(Ref: Plurality group)				0.002				0.792				0.527
	Non-plurality groups	-0.16	(-0.26, -0.06)	0.05		-0.02	(-0.14, 0.11)	0.06		0.04	(-0.07, 0.15)	0.06	

Table S23d. Sensitivity to unmeasured confounding of childhood predictors in United States

		Cantril's Ladder		Life Satisfaction		Happiness	
Variable	Category	E-value for Estimate	E-value for 95% CI	E-value for Estimate	E-value for 95% CI	E-value for Estimate	E-value for 95% CI
Relationship with mother	(Ref: Very bad/somewhat bad)						
	Very good/somewhat good	1.40	1.07	1.36	1.00	1.49	1.24
Relationship with father	(Ref: Very bad/somewhat bad)						
	Very good/somewhat good	1.21	1.00	1.37	1.10	1.44	1.23
Parent marital status	(Ref: Parents married)						
	Divorced	1.10	1.00	1.10	1.00	1.17	1.00
	Parents were never married	1.43	1.00	1.36	1.00	1.11	1.00
	One or both parents had died	1.45	1.00	1.48	1.00	1.17	1.00
Subjective financial status of family growing up	(Ref: Got by)						
	Lived comfortably	1.41	1.26	1.40	1.23	1.36	1.19
	Found it difficult	1.12	1.00	1.43	1.19	1.43	1.21
	Found it very difficult	1.37	1.00	1.24	1.00	1.43	1.00
Abuse	(Ref: No)						
	Yes	1.60	1.43	1.66	1.48	1.59	1.42
Outsider growing up	(Ref: No)						
	Yes	1.67	1.48	1.82	1.63	1.83	1.66
Self-rated health growing up	(Ref: Good)						
	Excellent	2.20	1.98	2.11	1.89	2.25	2.02
	Very good	1.73	1.52	1.63	1.40	1.72	1.50
	Fair	1.44	1.00	1.39	1.00	1.34	1.00
	Poor	1.78	1.00	1.82	1.00	1.75	1.00
Immigration status	(Ref: Born in this country)						
	Born in another country	1.61	1.31	1.46	1.12	1.39	1.00
Age 12 religious service attendance	(Ref: Never)						
	At least 1/week	1.46	1.22	1.37	1.00	1.39	1.00
	1-3/month	1.50	1.25	1.43	1.11	1.29	1.00
	< 1/month	1.29	1.00	1.13	1.00	1.20	1.00
Year of birth	(Ref: 1998-2005; current age: 18-24)						
	1993-1998; age 25-29	1.19	1.00	1.36	1.00	1.24	1.00
	1983-1993; age 30-39	1.55	1.00	1.40	1.00	1.59	1.10
	1973-1983; age 40-49	1.71	1.22	1.53	1.00	1.65	1.22
	1963-1973; age 50-59	1.98	1.55	1.86	1.44	2.07	1.68

Variable	Category	Cantril's Ladder E-value for Estimate	Cantril's Ladder E-value for 95% CI	Life Satisfaction E-value for Estimate	Life Satisfaction E-value for 95% CI	Happiness E-value for Estimate	Happiness E-value for 95% CI
	1953-1963; age 60-69	2.45	2.00	2.23	1.80	2.46	2.05
	1943-1953; age 70-79	2.97	2.46	2.58	2.11	2.88	2.43
	1943 or earlier; age 80+	3.11	2.46	2.51	1.89	2.88	2.27
Gender	(Ref: Male)						
	Female	1.36	1.21	1.35	1.19	1.33	1.18
	Other	1.30	1.00	1.65	1.00	1.49	1.00
Religious affiliation	(Ref: No religion/Atheist/Agnostic)						
	Christianity	1.37	1.00	1.45	1.11	1.49	1.17
	Collapsed affiliations with prevalence<3%	1.01	1.00	1.23	1.00	1.42	1.00
Race/ethnicity	(Ref: Plurality group)						
	Non-plurality groups	1.38	1.20	1.09	1.00	1.15	1.00

Table S23e. Complete-case supplemental analysis of means by demographic category for United States

		Cantril's Ladder				Life Satisfaction				Happiness			
Variable	Category	Mean	95% CI	SE	Global p-value	Mean	95% CI	SE	Global p-value	Mean	95% CI	SE	Global p-value
Age group	18-24	6.26	(5.96,6.57)	0.16	< .001	6.24	(5.89,6.60)	0.18	< .001	6.32	(6.02,6.62)	0.15	< .001
	25-29	6.23	(6.00,6.45)	0.12		6.22	(5.99,6.44)	0.12		6.30	(6.05,6.55)	0.13	
	30-39	6.53	(6.41,6.64)	0.06		6.49	(6.35,6.63)	0.07		6.64	(6.52,6.77)	0.06	
	40-49	6.75	(6.63,6.87)	0.06		6.74	(6.61,6.87)	0.06		6.78	(6.68,6.89)	0.05	
	50-59	7.07	(6.99,7.14)	0.04		7.12	(7.05,7.20)	0.04		7.24	(7.18,7.31)	0.03	
	60-69	7.46	(7.41,7.52)	0.03		7.47	(7.42,7.53)	0.03		7.57	(7.51,7.62)	0.03	
	70-79	7.80	(7.73,7.87)	0.04		7.76	(7.69,7.83)	0.04		7.86	(7.80,7.92)	0.03	
	80 or older	7.91	(7.66,8.16)	0.13		7.88	(7.63,8.13)	0.13		7.89	(7.62,8.17)	0.14	
Gender	Female	7.01	(6.94,7.08)	0.04	< .001	7.01	(6.93,7.08)	0.04	< .001	7.09	(7.03,7.16)	0.03	0.030
	Male	6.94	(6.87,7.01)	0.04		6.95	(6.88,7.02)	0.04		7.05	(6.98,7.12)	0.04	
	Other	6.09	(5.71,6.47)	0.19		5.70	(5.19,6.20)	0.26		5.99	(5.14,6.84)	0.43	
Marital status	Divorced	6.75	(6.64,6.86)	0.06	< .001	6.81	(6.70,6.92)	0.06	< .001	7.01	(6.91,7.11)	0.05	< .001
	Domestic partner	6.55	(6.34,6.76)	0.11		6.42	(6.18,6.65)	0.12		6.48	(6.26,6.70)	0.11	
	Married	7.40	(7.36,7.45)	0.02		7.44	(7.39,7.48)	0.02		7.47	(7.42,7.51)	0.02	
	Separated	6.00	(5.45,6.55)	0.28		5.78	(5.12,6.44)	0.34		6.35	(5.91,6.79)	0.23	
	Single/Never been married	6.17	(6.04,6.30)	0.07		6.12	(5.98,6.25)	0.07		6.28	(6.15,6.41)	0.07	
	Widowed	7.38	(7.19,7.57)	0.10		7.33	(7.14,7.52)	0.10		7.44	(7.23,7.65)	0.11	
Employment	Employed for an employer	6.90	(6.84,6.95)	0.03	< .001	6.84	(6.78,6.91)	0.03	< .001	6.96	(6.89,7.02)	0.03	< .001
	Homemaker	6.95	(6.72,7.18)	0.12		7.01	(6.81,7.22)	0.10		7.06	(6.86,7.25)	0.10	

Variable	Category	Cantril's Ladder				Life Satisfaction				Happiness			
		Mean	95% CI	SE	Global p-value	Mean	95% CI	SE	Global p-value	Mean	95% CI	SE	Global p-value
	None of these/Other	5.72	(5.44,6.00)	0.14		6.17	(5.73,6.61)	0.22		6.43	(6.01,6.84)	0.21	
	Retired	7.67	(7.60,7.73)	0.03		7.66	(7.59,7.72)	0.03		7.71	(7.64,7.78)	0.03	
	Self-employed	6.97	(6.77,7.17)	0.10		7.06	(6.90,7.22)	0.08		7.22	(7.09,7.34)	0.06	
	Student	6.18	(5.79,6.58)	0.20		6.00	(5.43,6.56)	0.29		6.04	(5.66,6.41)	0.19	
	Unemployed and looking for a job	5.53	(5.11,5.95)	0.21		5.57	(5.13,6.01)	0.22		5.62	(5.28,5.96)	0.17	
Religious service attendance	A few times a year	7.12	(7.04,7.19)	0.04	< .001	7.08	(6.99,7.18)	0.05	< .001	7.22	(7.12,7.31)	0.05	< .001
	More than once a week	7.73	(7.59,7.88)	0.07		7.88	(7.74,8.01)	0.07		8.00	(7.87,8.13)	0.07	
	Never	6.56	(6.48,6.64)	0.04		6.49	(6.40,6.57)	0.04		6.58	(6.50,6.65)	0.04	
	Once a week	7.55	(7.46,7.63)	0.04		7.69	(7.61,7.78)	0.04		7.73	(7.66,7.80)	0.04	
	One to three times a month	7.12	(6.89,7.34)	0.12		7.19	(7.02,7.37)	0.09		7.36	(7.22,7.49)	0.07	
Education	Up to 8 years	7.10	(6.27,7.94)	0.42	< .001	7.25	(6.12,8.38)	0.57	< .001	6.72	(5.99,7.46)	0.37	< .001
	16+ years	7.36	(7.33,7.39)	0.02		7.24	(7.20,7.27)	0.02		7.25	(7.22,7.29)	0.02	
	9 to 15 years	6.77	(6.70,6.84)	0.04		6.83	(6.75,6.90)	0.04		6.97	(6.89,7.04)	0.04	
Immigration status	Born in another country	7.14	(6.98,7.31)	0.09	0.031	7.21	(7.02,7.39)	0.09	0.007	7.22	(7.06,7.38)	0.08	0.045
	Born in this country	6.95	(6.90,7.00)	0.03		6.94	(6.89,7.00)	0.03		7.05	(7.00,7.10)	0.03	
Religious affiliation	Buddhism	7.02	(6.63,7.41)	0.20	< .001	6.84	(6.40,7.28)	0.22	< .001	7.03	(6.64,7.41)	0.20	< .001
	Christianity	7.23	(7.17,7.29)	0.03		7.30	(7.24,7.36)	0.03		7.40	(7.34,7.46)	0.03	
	Confucianism	6.91	*	*		7.25	*	*		7.34	*	*	
	Hinduism	7.32	(7.03,7.61)	0.14		7.26	(6.83,7.69)	0.22		7.33	(6.93,7.72)	0.20	
	Islam	6.67	(6.07,7.27)	0.31		6.40	(5.53,7.28)	0.44		6.16	(5.39,6.93)	0.39	
	Judaism	7.38	(7.19,7.56)	0.09		7.32	(7.12,7.52)	0.10		7.26	(7.08,7.45)	0.09	
	No religion/Atheist/Agnostic	6.57	(6.49,6.66)	0.04		6.45	(6.36,6.55)	0.05		6.49	(6.40,6.58)	0.05	
	Primal, Animist, or Folk religion	5.06	(4.15,5.98)	0.46		4.82	(3.57,6.07)	0.63		5.77	(5.08,6.45)	0.35	
	Sikhism	7.20	(6.19,8.20)	0.43		7.31	(6.36,8.27)	0.41		7.55	(6.60,8.51)	0.41	

		Cantril's Ladder				Life Satisfaction				Happiness			
Variable	Category	Mean	95% CI	SE	Global p-value	Mean	95% CI	SE	Global p-value	Mean	95% CI	SE	Global p-value
	Some other religion	6.11	(5.69,6.53)	0.21		6.20	(5.89,6.51)	0.16		6.52	(6.28,6.75)	0.12	
	Taoism	5.10	(4.12,6.09)	0.49		4.46	(2.94,5.97)	0.76		6.54	(5.64,7.43)	0.45	
	Baha'i	7.50	(1.98,13.02)	0.75		7.07	(0.64,13.51)	0.87		7.39	(3.25,11.54)	0.56	
	Jainism	5.15	(2.06,8.23)	0.81		6.33	(5.12,7.55)	0.32		5.14	(2.01,8.28)	0.82	
	Shinto	8.60	(7.13,10.07)	0.67		8.49	(6.80,10.18)	0.77		8.59	(7.05,10.14)	0.70	

Table S23f. Complete-case supplemental analysis of childhood predictors regression analysis results for United States

		Cantril's Ladder				Life Satisfaction				Happiness			
Variable	Category	Est	95% CI	SE	Global p-value	Est	95% CI	SE	Global p-value	Est	95% CI	SE	Global p-value
Relationship with mother	(Ref: Very bad/somewhat bad)				0.033				0.662				0.002
	Very good/somewhat good	0.18	(0.01,0.34)	0.08		0.03	(-0.12,0.19)	0.08		0.23	(0.08,0.38)	0.08	
Relationship with father	(Ref: Very bad/somewhat bad)				0.365				0.077				0.002
	Very good/somewhat good	0.06	(-0.07,0.19)	0.06		0.14	(-0.02,0.29)	0.08		0.21	(0.08,0.34)	0.07	
Parent marital status	(Ref: Parents married)				0.237				0.204				0.917
	No, one or both of them had died	-0.18	(-0.56,0.20)	0.19		-0.25	(-0.54,0.05)	0.15		0.02	(-0.30,0.33)	0.16	
	No, they were never married	-0.21	(-0.47,0.06)	0.14		-0.17	(-0.48,0.14)	0.16		-0.02	(-0.30,0.26)	0.14	
	Yes, married	0.03	(-0.12,0.17)	0.07		0.04	(-0.12,0.20)	0.08		-0.04	(-0.18,0.09)	0.07	
Subjective financial status of family growing up	(Ref: Got by)				<.001				0.007				0.001
	Found it difficult	-0.06	(-0.20,0.08)	0.07		0.14	(-0.01,0.28)	0.07		0.19	(0.06,0.32)	0.07	
	Found it very difficult	-0.12	(-0.36,0.11)	0.12		-0.03	(-0.25,0.20)	0.11		-0.04	(-0.28,0.19)	0.12	
	Lived comfortably	0.15	(0.06,0.24)	0.05		0.17	(0.06,0.28)	0.05		0.15	(0.06,0.25)	0.05	
Abuse	(Ref: No)				<.001				<.001				<.001
	Yes	-0.32	(-0.43,-0.21)	0.06		-0.36	(-0.49,-0.23)	0.07		-0.29	(-0.40,-0.17)	0.06	
Outsider growing up	(Ref: No)				<.001				<.001				<.001
	Yes	-0.31	(-0.44,-0.18)	0.07		-0.46	(-0.60,-0.31)	0.08		-0.42	(-0.54,-0.29)	0.06	

		Cantril's Ladder				Life Satisfaction				Happiness			
Variable	Category	Est	95% CI	SE	Global p-value	Est	95% CI	SE	Global p-value	Est	95% CI	SE	Global p-value
Self-rated health growing up	(Ref: Good)				<.001				<.001				<.001
	Excellent	0.70	(0.56,0.84)	0.07		0.73	(0.57,0.89)	0.08		0.81	(0.66,0.96)	0.08	
	Fair	-0.25	(-0.53,0.03)	0.14		-0.33	(-0.68,0.03)	0.18		-0.16	(-0.44,0.12)	0.14	
	Poor	-0.07	(-0.58,0.44)	0.26		-0.05	(-0.67,0.57)	0.32		-0.07	(-0.66,0.53)	0.30	
	Very good	0.36	(0.22,0.50)	0.07		0.36	(0.20,0.51)	0.08		0.43	(0.28,0.58)	0.08	
Immigration status	(Ref: Born in this country)				<.001				<.001				0.026
	Born in another country	0.32	(0.14,0.49)	0.09		0.31	(0.13,0.49)	0.09		0.19	(0.02,0.36)	0.09	
Age 12 religious service attendance	(Ref: Never)				0.043				0.008				0.031
	At least once a week	0.16	(0.01,0.30)	0.07		0.17	(-0.00,0.34)	0.09		0.18	(0.02,0.33)	0.08	
	Less than once a month	0.04	(-0.13,0.21)	0.09		-0.04	(-0.24,0.15)	0.10		0.03	(-0.15,0.20)	0.09	
	One to three times a month	0.19	(0.04,0.34)	0.08		0.18	(-0.00,0.37)	0.09		0.09	(-0.08,0.27)	0.09	
Year of birth	(Ref: 1998-2005; current age: 18-24)				<.001				<.001				<.001
	1993-1998; age 25-29	0.01	(-0.32,0.34)	0.17		-0.01	(-0.39,0.36)	0.19		0.01	(-0.33,0.34)	0.17	
	1983-1993; age 30-39	0.21	(-0.09,0.50)	0.15		0.16	(-0.17,0.50)	0.17		0.26	(-0.02,0.54)	0.14	
	1973-1983; age 40-49	0.35	(0.05,0.65)	0.15		0.31	(-0.03,0.65)	0.17		0.31	(0.04,0.59)	0.14	
	1963-1973; age 50-59	0.52	(0.24,0.81)	0.15		0.56	(0.24,0.88)	0.16		0.63	(0.37,0.89)	0.13	
	1953-1963; age 60-69	0.82	(0.54,1.10)	0.14		0.82	(0.51,1.14)	0.16		0.87	(0.61,1.13)	0.13	
	1943-1953; age 70-79	1.11	(0.83,1.40)	0.15		1.07	(0.74,1.39)	0.17		1.13	(0.87,1.39)	0.13	

		Cantril's Ladder				Life Satisfaction				Happiness			
Variable	Category	Est	95% CI	SE	Global p-value	Est	95% CI	SE	Global p-value	Est	95% CI	SE	Global p-value
	1943 or earlier; age 80+	1.19	(0.83,1.55)	0.18		1.12	(0.70,1.53)	0.21		1.12	(0.75,1.49)	0.19	
Gender	(Ref: Male)				<.001				<.001				<.001
	Male	-0.17	(-0.25,-0.09)	0.04		-0.16	(-0.25,-0.07)	0.05		-0.16	(-0.24,-0.07)	0.04	
	Other	-0.32	(-0.64,0.00)	0.16		-0.67	(-1.12,-0.21)	0.23		-0.48	(-1.29,0.33)	0.41	
Religious affiliation	(Ref: No religion/Atheist/Agnostic)				0.036				0.045				0.028
	Christianity	0.17	(0.01,0.34)	0.08		0.19	(-0.00,0.38)	0.10		0.23	(0.06,0.41)	0.09	
	Christianity	0.17	(0.01,0.34)	0.08		0.19	(-0.00,0.38)	0.10		0.23	(0.06,0.41)	0.09	
	Collapsed affiliations with prevalence<3%	-0.01	(-0.26,0.24)	0.13		0.01	(-0.24,0.26)	0.13		0.14	(-0.09,0.36)	0.11	
Race/ethnicity	(Ref: Plurality group)				0.002				0.823				0.411
	Non-plurality groups	-0.16	(-0.26,-0.06)	0.05		-0.01	(-0.13,0.10)	0.06		0.04	(-0.06,0.15)	0.05	

Table S23g. Complete-case supplemental analysis of sensitivity to unmeasured confounding of childhood predictors in United States

		Cantril's Ladder		Life Satisfaction		Happiness	
Variable	Category	E-value for Estimate	E-value for 95% CI	E-value for Estimate	E-value for 95% CI	E-value for Estimate	E-value for 95% CI
Relationship with mother	(Ref: Very bad/somewhat bad)						
	Very good/somewhat good	1.41	1.09	1.14	1.00	1.49	1.25
Relationship with father	(Ref: Very bad/somewhat bad)						
	Very good/somewhat good	1.21	1.00	1.33	1.00	1.46	1.25
Parent marital status	(Ref: Parents married)						
	No, one or both of them had died	1.41	1.00	1.49	1.00	1.09	1.00
	No, they were never married	1.46	1.00	1.38	1.00	1.11	1.00
	Yes, married	1.13	1.00	1.15	1.00	1.17	1.00
Subjective financial status of family growing up	(Ref: Got by)						
	Found it difficult	1.20	1.00	1.33	1.00	1.43	1.21
	Found it very difficult	1.32	1.00	1.13	1.00	1.17	1.00
	Lived comfortably	1.38	1.22	1.38	1.21	1.37	1.20
Abuse	(Ref: No)						
	Yes	1.63	1.46	1.64	1.46	1.57	1.40
Outsider growing up	(Ref: No)						
	Yes	1.61	1.42	1.77	1.57	1.76	1.58
Self-rated health growing up	(Ref: Good)						
	Excellent	2.20	1.99	2.15	1.93	2.36	2.12
	Fair	1.53	1.00	1.60	1.00	1.38	1.00
	Poor	1.23	1.00	1.18	1.00	1.22	1.00
	Very good	1.69	1.48	1.64	1.43	1.78	1.57
Immigration status	(Ref: Born in this country)						
	Born in another country	1.63	1.36	1.57	1.31	1.43	1.12
Age 12 religious service attendance	(Ref: Never)						
	At least once a week	1.38	1.09	1.37	1.00	1.41	1.11
	Less than once a month	1.16	1.00	1.16	1.00	1.13	1.00
	One to three times a month	1.43	1.15	1.40	1.00	1.27	1.00
Year of birth	(Ref: 1998-2005; current age: 18-24)						

Variable	Category	Cantril's Ladder		Life Satisfaction		Happiness	
		E-value for Estimate	E-value for 95% CI	E-value for Estimate	E-value for 95% CI	E-value for Estimate	E-value for 95% CI
	1993-1998; age 25-29	1.08	1.00	1.08	1.00	1.06	1.00
	1983-1993; age 30-39	1.46	1.00	1.37	1.00	1.54	1.00
	1973-1983; age 40-49	1.67	1.18	1.57	1.00	1.61	1.17
	1963-1973; age 50-59	1.93	1.51	1.91	1.48	2.08	1.69
	1953-1963; age 60-69	2.40	1.95	2.28	1.84	2.46	2.05
	1943-1953; age 70-79	2.90	2.40	2.65	2.16	2.90	2.45
	1943 or earlier; age 80+	3.05	2.41	2.74	2.11	2.88	2.26
Gender	(Ref: Male)						
	Male	1.40	1.26	1.36	1.21	1.38	1.24
	Other	1.63	1.00	2.06	1.44	1.85	1.00
Religious affiliation	(Ref: No religion/Atheist/Agnostic)						
	Christianity	1.41	1.07	1.41	1.00	1.49	1.20
	Christianity	1.41	1.07	1.41	1.00	1.49	1.20
	Collapsed affiliations with prevalence<3%	1.09	1.00	1.06	1.00	1.34	1.00
Race/ethnicity	(Ref: Plurality group)						
	Non-plurality groups	1.39	1.21	1.08	1.00	1.17	1.00

Tables S24a-c: Population weighted meta-analysis results

Table S24a. Population weighted meta-analysis of demographic means

		Cantril's Ladder			Life Satisfaction			Happiness		
Variable	Category	Est	95% CI	SE	Est	95% CI	SE	Est	95% CI	SE
Age group										
	18-24	6.63	(6.55,6.72)	0.044	7.46	(7.38,7.53)	0.036	7.37	(7.29,7.44)	0.037
	25-29	6.50	(6.42,6.58)	0.042	7.37	(7.30,7.45)	0.037	7.27	(7.20,7.35)	0.037
	30-39	6.48	(6.43,6.54)	0.029	7.34	(7.28,7.39)	0.027	7.22	(7.17,7.27)	0.027
	40-49	6.44	(6.38,6.51)	0.034	7.29	(7.23,7.35)	0.031	7.16	(7.10,7.22)	0.031
	50-59	6.56	(6.48,6.64)	0.041	7.33	(7.26,7.41)	0.040	7.18	(7.11,7.26)	0.039
	60-69	6.70	(6.60,6.81)	0.055	7.48	(7.37,7.58)	0.054	7.31	(7.21,7.42)	0.053
	70-79	6.85	(6.66,7.05)	0.098	7.68	(7.51,7.84)	0.084	7.58	(7.40,7.75)	0.089
	80 or older	7.06	(6.62,7.50)	0.224	7.85	(7.45,8.25)	0.204	7.80	(7.41,8.20)	0.201
Gender										
	Male	6.49	(6.44,6.53)	0.023	7.38	(7.34,7.42)	0.021	7.24	(7.19,7.28)	0.021
	Female	6.72	(6.67,6.77)	0.024	7.51	(7.47,7.55)	0.020	7.40	(7.36,7.45)	0.021
	Other	5.85	(5.39,6.31)	0.234	6.20	(5.86,6.54)	0.173	6.05	(5.39,6.72)	0.341
Marital status										
	Married	6.74	(6.70,6.78)	0.020	7.59	(7.55,7.62)	0.017	7.45	(7.41,7.48)	0.018
	Separated	5.97	(5.38,6.56)	0.299	6.83	(6.30,7.36)	0.271	6.91	(6.46,7.36)	0.230
	Divorced	5.46	(4.82,6.11)	0.329	7.25	(6.59,7.92)	0.338	6.51	(5.91,7.11)	0.305
	Widowed	6.53	(6.37,6.70)	0.084	7.30	(7.14,7.46)	0.082	7.17	(7.01,7.33)	0.082
	Domestic partner	6.30	(6.04,6.56)	0.133	7.29	(6.96,7.62)	0.169	7.15	(6.91,7.39)	0.122
	Single, never married	6.48	(6.41,6.55)	0.036	7.31	(7.24,7.37)	0.033	7.21	(7.14,7.27)	0.033
Employment status										
	Employed for an employer	6.47	(6.40,6.54)	0.036	7.32	(7.26,7.39)	0.033	7.22	(7.16,7.28)	0.032
	Self-employed	6.61	(6.55,6.67)	0.031	7.49	(7.43,7.55)	0.030	7.35	(7.29,7.41)	0.031

Variable	Category	Cantril's Ladder			Life Satisfaction			Happiness		
		Est	95% CI	SE	Est	95% CI	SE	Est	95% CI	SE
	Retired	6.77	(6.57,6.97)	0.100	7.59	(7.40,7.78)	0.096	7.47	(7.30,7.64)	0.087
	Student	6.64	(6.51,6.77)	0.065	7.35	(7.24,7.47)	0.058	7.27	(7.16,7.37)	0.053
	Homemaker	6.69	(6.63,6.76)	0.034	7.53	(7.47,7.58)	0.030	7.40	(7.34,7.46)	0.029
	Unemployed and looking for a job	6.11	(5.99,6.24)	0.063	7.01	(6.90,7.13)	0.059	6.92	(6.81,7.02)	0.054
	None of these/other	6.44	(6.29,6.59)	0.076	7.27	(7.14,7.41)	0.069	7.17	(7.03,7.32)	0.075
Education										
	Up to 8 years	6.59	(6.49,6.69)	0.051	7.49	(7.36,7.62)	0.065	7.29	(7.20,7.38)	0.045
	9-15 years	6.48	(6.41,6.55)	0.037	7.42	(7.35,7.48)	0.033	7.30	(7.22,7.37)	0.037
	16+ years	6.67	(6.51,6.84)	0.086	7.52	(7.39,7.66)	0.070	7.52	(7.37,7.67)	0.078
Religious service attendance										
	>1/week	6.86	(6.79,6.93)	0.035	7.77	(7.71,7.83)	0.032	7.61	(7.55,7.67)	0.032
	1/week	6.82	(6.75,6.88)	0.032	7.70	(7.64,7.75)	0.028	7.56	(7.51,7.62)	0.029
	1-3/month	6.63	(6.56,6.70)	0.038	7.45	(7.38,7.51)	0.033	7.36	(7.30,7.42)	0.033
	A few times a year	6.49	(6.43,6.56)	0.033	7.32	(7.26,7.39)	0.033	7.20	(7.13,7.26)	0.034
	Never	6.48	(6.37,6.58)	0.052	7.21	(7.12,7.30)	0.046	7.14	(7.05,7.22)	0.044
Immigration status										
	Born in another country	6.44	(6.18,6.70)	0.133	7.11	(6.72,7.50)	0.199	6.77	(6.51,7.04)	0.133
	Born in this country	6.60	(6.57,6.64)	0.018	7.44	(7.41,7.47)	0.016	7.32	(7.29,7.35)	0.016

Table S24b. Population weighted meta-analysis of childhood predictors

		Cantril's Ladder			Life Satisfaction			Happiness		
Variable	Category	Est	95% CI	SE	Est	95% CI	SE	Est	95% CI	SE
Relationship with mother	(Ref: Very bad/somewhat bad)									
	Very good/somewhat good	0.03	(-0.18,0.23)	0.104	0.16	(-0.02,0.34)	0.091	0.09	(-0.10,0.27)	0.095
Relationship with father	(Ref: Very bad/somewhat bad)									
	Very good/somewhat good	0.10	(-0.08,0.29)	0.094	0.13	(-0.03,0.28)	0.079	0.07	(-0.09,0.24)	0.084
Parent marital status	(Ref: Parents married)									
	No, one or both of them had died	0.03	(-0.19,0.24)	0.110	0.06	(-0.12,0.23)	0.089	0.01	(-0.16,0.18)	0.086
	Single, never married	-0.15	(-0.36,0.07)	0.111	-0.16	(-0.34,0.02)	0.091	-0.22	(-0.40,-0.04)	0.091
	Yes, married	-0.08	(-0.26,0.11)	0.095	-0.13	(-0.29,0.02)	0.077	-0.15	(-0.30,-0.00)	0.076
Subjective financial status of family growing up	(Ref: Got by)									
	Lived comfortably	0.27	(0.19,0.34)	0.039	0.17	(0.11,0.23)	0.032	0.16	(0.09,0.23)	0.035
	Found it difficult	-0.18	(-0.27,-0.09)	0.045	-0.11	(-0.19,-0.02)	0.042	-0.15	(-0.23,-0.07)	0.041
	Found it very difficult	-0.30	(-0.42,-0.18)	0.062	-0.25	(-0.35,-0.15)	0.051	-0.30	(-0.40,-0.20)	0.052
Abuse	(Ref: No)									
	Yes	0.02	(-0.07,0.11)	0.045	-0.19	(-0.27,-0.11)	0.042	-0.11	(-0.19,-0.03)	0.042
Outsider growing up	(Ref: No)									
	Yes	-0.03	(-0.13,0.07)	0.049	-0.11	(-0.19,-0.03)	0.040	-0.14	(-0.23,-0.06)	0.042

		Cantril's Ladder			Life Satisfaction			Happiness		
Variable	Category	Est	95% CI	SE	Est	95% CI	SE	Est	95% CI	SE
Self-rated health growing up	(Ref: Good)									
	Excellent	0.12	(0.03,0.20)	0.045	0.23	(0.15,0.32)	0.044	0.27	(0.18,0.35)	0.043
	Very good	0.07	(-0.00,0.15)	0.040	0.14	(0.08,0.21)	0.034	0.16	(0.09,0.22)	0.035
	Fair	-0.25	(-0.34,-0.16)	0.048	-0.23	(-0.32,-0.14)	0.045	-0.26	(-0.34,-0.18)	0.043
	Poor	-0.30	(-0.50,-0.10)	0.103	-0.23	(-0.43,-0.04)	0.101	-0.28	(-0.47,-0.09)	0.095
Immigration status	(Ref: Born in this country)									
	Born in another country	-0.07	(-0.31,0.18)	0.125	-0.22	(-0.58,0.15)	0.185	-0.42	(-0.66,-0.18)	0.121
Age 12 religious service attendance	(Ref: Never)									
	At least 1/week	0.10	(-0.01,0.21)	0.056	0.16	(0.07,0.25)	0.047	0.15	(0.06,0.25)	0.049
	1-3/month	0.13	(0.01,0.25)	0.062	0.14	(0.04,0.25)	0.052	0.10	(-0.01,0.20)	0.053
	Less than 1/month	0.04	(-0.08,0.16)	0.062	0.05	(-0.06,0.15)	0.055	0.01	(-0.09,0.12)	0.055
Year of birth	(Ref: 1998-2005; age 18-24)									
	1993-1998; age 25-29	-0.11	(-0.22,0.00)	0.056	-0.07	(-0.17,0.02)	0.048	-0.08	(-0.18,0.01)	0.048
	1983-1993; age 30-39	-0.12	(-0.22,-0.03)	0.048	-0.11	(-0.19,-0.03)	0.042	-0.13	(-0.22,-0.05)	0.043
	1973-1983; age 40-49	-0.16	(-0.26,-0.05)	0.054	-0.16	(-0.25,-0.07)	0.045	-0.20	(-0.29,-0.11)	0.046
	1963-1973; age 50-59	-0.05	(-0.16,0.07)	0.058	-0.12	(-0.23,-0.02)	0.053	-0.18	(-0.28,-0.07)	0.052
	1953-1963; age 60-69	0.09	(-0.05,0.22)	0.068	0.00	(-0.12,0.13)	0.064	-0.06	(-0.19,0.06)	0.063
	1943-1953; age 70-79	0.22	(0.01,0.43)	0.109	0.17	(-0.00,0.35)	0.089	0.17	(-0.01,0.36)	0.092
	1943 or earlier; age 80+	0.39	(-0.05,0.83)	0.225	0.31	(-0.08,0.71)	0.202	0.38	(-0.01,0.77)	0.200
Gender	(Ref: Male)									

		Cantril's Ladder			Life Satisfaction			Happiness		
Variable	Category	Est	95% CI	SE	Est	95% CI	SE	Est	95% CI	SE
	Male	-0.26	(-0.32,-0.20)	0.030	-0.16	(-0.21,-0.11)	0.026	-0.19	(-0.25,-0.14)	0.028
	Other	-0.70	(-1.12,-0.27)	0.215	-0.60	(-0.90,-0.30)	0.152	-0.93	(-1.52,-0.33)	0.304

Table S24c. Sensitivity to unmeasured confounding of population weighted meta-analysis of childhood predictors

		Cantril's Ladder		Life Satisfaction		Happiness	
Variable	Category	E-value for Estimate	E-value for 95% CI	E-value for Estimate	E-value for 95% CI	E-value for Estimate	E-value for 95% CI
Relationship with mother	(Ref: Very bad/somewhat bad)						
	Very good/somewhat good	1.11	1.00	1.31	1.00	1.22	1.00
Relationship with father	(Ref: Very bad/somewhat bad)						
	Very good/somewhat good	1.25	1.00	1.27	1.00	1.20	1.00
Parent marital status	(Ref: Parents married)						
	No, one or both of them had died	1.11	1.00	1.17	1.00	1.07	1.00
	Single, never married	1.31	1.00	1.31	1.00	1.40	1.15
	Yes, married	1.21	1.00	1.28	1.00	1.32	1.03
Subjective financial status of family growing up	(Ref: Got by)						
	Lived comfortably	1.46	1.36	1.33	1.24	1.33	1.23
	Found it difficult	1.35	1.23	1.24	1.10	1.31	1.19
	Found it very difficult	1.50	1.35	1.42	1.30	1.50	1.38
Abuse	(Ref: No)						
	Yes	1.09	1.00	1.35	1.24	1.26	1.12
Outsider growing up	(Ref: No)						
	Yes	1.12	1.00	1.24	1.12	1.31	1.18
Self-rated health growing up	(Ref: Good)						
	Excellent	1.27	1.12	1.40	1.30	1.46	1.36
	Very good	1.20	1.00	1.29	1.20	1.32	1.22
	Fair	1.44	1.32	1.40	1.29	1.45	1.35
	Poor	1.50	1.24	1.40	1.13	1.48	1.24
Immigration status	(Ref: Born in this country)						

Variable	Category	Cantril's Ladder		Life Satisfaction		Happiness	
		E-value for Estimate	E-value for 95% CI	E-value for Estimate	E-value for 95% CI	E-value for Estimate	E-value for 95% CI
	Born in another country	1.19	1.00	1.38	1.00	1.64	1.36
Age 12 religious service attendance	(Ref: Never)						
	At least 1/week	1.24	1.00	1.31	1.18	1.32	1.17
	1-3/month	1.29	1.06	1.29	1.14	1.24	1.00
	Less than 1/month	1.14	1.00	1.15	1.00	1.08	1.00
Year of birth	(Ref: 1998-2005; age 18-24)						
	1993-1998; age 25-29	1.26	1.00	1.19	1.00	1.22	1.00
	1983-1993; age 30-39	1.28	1.12	1.25	1.11	1.29	1.16
	1973-1983; age 40-49	1.32	1.17	1.31	1.19	1.37	1.25
	1963-1973; age 50-59	1.16	1.00	1.26	1.09	1.35	1.20
	1953-1963; age 60-69	1.22	1.00	1.04	1.00	1.18	1.00
	1943-1953; age 70-79	1.40	1.06	1.33	1.00	1.35	1.00
	1943 or earlier; age 80+	1.60	1.00	1.49	1.00	1.59	1.00
Gender	(Ref: Male)						
	Male	1.45	1.38	1.31	1.24	1.37	1.30
	Other	1.95	1.47	1.79	1.47	2.23	1.54

Tables S25a-b. CL data in the GFS, GWP, and WHR

Table S25a. Cantril ladder data in the GFS, the Gallup World Poll, and the World Happiness Report

Nation	GFS 2023				GWP 2021		GWP 2022		GWP 2023				WHR (2021-2023)		WHR rank w/ GFS	WHR: GFS
	Sampling (probability)	Sampling (non-probability)	Score	Rank (/22)	Score	Rank (/122)	Score	Rank (/142)	Sampling	Score	GFS differ	Rank (/142)	Score	Rank (/143)		
Israel	11/7/22 – 11/23/23		7.33	1	7.57	3	7.66	2	10/17/23 – 12/2/23	6.78	- 0.55	22	7.34	5	6	↓1
Sweden		1/16/23 – 2/22/23	7.20	2	7.49	4	7.35	8	5/26/23 – 7/19/23	7.16	- 0.04	7	7.34	4	8	↓4
Poland	12/14/22 - 10/13/23	6/10/23 – 10/16/23	7.12	3	5.95	55	6.67	26	8/27/23 – 10/29/23	6.68	- 0.44	24	6.44	35	9	↑26
Mexico	10/29/22 – 12/13/22	5/9/23 – 11/20/23	7.10	4	5.96	52	7.04	12	8/5/23 – 11/7/23	7.01	- 0.09	11	6.68	25	9	↑16
Indonesia	11/7/23 - 12/27/23		6.97	5	5.34	76	5.58	78	8/23/23 – 9/20/23	5.69	- 1.28	79	5.57	80	12	↑68
US	8/4/22 – 4/4/23		6.94	6	7.69	15	6.83	20	5/16/23 – 6/27/23	6.52	- 0.42	34	6.72	23	14	↑9
Hong Kong	10/10/23 - 11/24/23		6.85	7	5.44	74	5.42	84	8/11/23 – 10/9/23	5.61	- 1.24	81	5.32	86	17	↑69
Australia	3/21/22 – 9/26/23	4/12/23 – 9/26/23	6.79	8	6.94	11	6.97	14	4/15/23 – 7/8/23	7.02	+ 0.23	9	7.06	10	20	↓10
Argentina	11/29/22 – 11/30/23	4/11/23 – 11/14/23	6.75	9	6.14	41	6.26	41	8/5/23 – 10/12/23	6.39.	- 0.36	41	6.19	48	20	↑28
Germany	8/11/22 – 11/16/23	6/12/23 – 8/27/23	6.74	10	6.50	28	6.69	24	4/24/23 – 7/12/23	6.79	+ 0.05	20	6.72	24	22	↑2
Spain	8/17/22 – 11/8/23	6/22/23 – 8/30/23	6.67	11	5.82	58	6.37	36	4/24/23 – 7/12/23	6.46	- 0.21	37	6.42	36	26	↑10
Brazil	11/23/22 – 11/26/23	4/11/23 - 11/7/23	6.59	12	5.95	53	6.26	42	9/11/23 – 11/5/23	6.55	- 0.04	31	6.27	44	29	↑15
UK	4/6/22 – 11/20/23	6/12/23 – 8/30/23	6.56	13	6.73	17	6.63	27	4/24/23 – 6/26/23	6.66	+ 0.10	26	6.75	20	30	↓10
Philippines	4/4/23 – 1/5/24		6.38	14	5.61	65	5.99	57	10/3/23 – 12/12/23	6.18	- 0.20	53	6.05	53	38	↑15
South Africa	2/26/23 – 12/8/23		6.11	15	5.32	77	5.58	77	10/19/23 – 2/1/24	5.08	- 1.03	96	5.42	83	50	↑33
Japan		12/13/22 – 6/30/23	5.90	16	6.08	44	6.19	44	6/13/23 – 9/14/23	5.91	+ 0.01	65	6.06	51	65	↓14
Nigeria	5/16/23 – 11/7/23		5.73	17	3.92	112	5.29	88	9/13/23 – 10/12/23	4.87	- 0.94	102	4.88	102	74	↑28
India	4/25/23 - 12/8/23		5.63	18	3.47	117	3.93	127	9/12/23 – 12/27/23	4.68	- 0.95	106	4.05	126	79	↑47
Kenya	4/13/23 – 11/21/23		5.51	19	4.32	103	4.45	111	10/16/23 – 11/10/23	4.50	- 1.01	112	4.47	114	81	↑33

Turkey	4/15/23 – 1/15/24	5.18	20	4.02	111	5.09	94	9/7/23 – 12/7/23	5.46	+ 0.28	86	4.97	98	92	↑6
Egypt	3/9/23 – 9/17/23	5.04	21	4.05	110	4.02	125	9/11/23 – 9/26/23	3.88	- 1.16	131	3.98	127	97	↑30
Tanzania	2/17/23 – 11/30/23	4.40	22	3.39	118	3.62	130	12/7/23 – 12/23/23	4.04	- 0.36	130	3.78	131	116	↑15

Table S25b. Summary of random-effects meta-analytic estimates (and pooled standard deviation across countries)

	Mean	95% CI	SE	Tau	Pooled SD
GFS	6.34	6.01, 6.67	0.17	0.79	2.46
GWP (all countries)	5.60	5.41, 5.78	0.09	1.13	2.59
GWP (GFS countries)	5.91	5.49, 6.33	0.21	1.00	2.47

Standardized mean difference between GFS and GWP:
GFS - GWP (all countries): 0.29
GFS - GWP (gfs countries): 0.17

Tables S26a-c. SWB and employment status across countries

Table S26a. Mean levels of Cantril's Ladder by employment status across 22 countries

Country	Employed for employer		Self employed		Retired		Student		Homemaker		Unemployed	
	Mean	95% CIs	Mean	95% CIs	Mean	95% CIs	Mean	95% CIs	Mean	95% CIs	Mean	95% CIs
Argentina	6.94	6.83, 7.06	6.73	6.58, 6.88	6.71	6.44, 6.97	6.78	6.52, 7.03	6.82	6.55, 7.09	6.11	5.80, 6.42
Australia	6.74	6.65, 6.83	6.99	6.76, 7.22	7.36	7.23, 7.50	6.26	5.92, 6.61	6.59	6.13, 7.04	5.54	4.99, 6.08
Brazil	6.83	6.76, 6.91	6.66	6.56, 6.76	7.18	6.99, 7.37	6.59	6.41, 6.78	6.60	6.41, 6.78	5.78	5.64, 5.92
Egypt	4.96	4.74, 5.18	4.70	4.46, 4.94	4.92	4.35, 5.49	5.60	5.27, 5.94	5.23	5.08, 5.38	4.78	4.33, 5.22
Germany	6.84	6.78, 6.90	6.96	6.81, 7.10	6.71	6.61, 6.81	6.73	6.55, 6.91	6.14	5.81, 6.46	5.46	5.14, 5.77
Hong Kong	6.92	6.82, 7.01	7.50	7.06, 7.94	6.60	6.26, 6.94	6.23	5.72, 6.74	6.51	5.99, 7.03	5.63	4.74, 6.53
India	5.20	5.01, 5.38	5.70	5.54, 5.86	5.87	5.37, 6.37	6.20	5.84, 6.56	5.87	5.73, 6.01	5.20	4.90, 5.50
Indonesia	6.89	6.71, 7.08	7.00	6.86, 7.14	7.39	6.93, 7.85	6.92	6.56, 7.28	7.13	6.98, 7.27	6.41	6.07, 6.75
Israel	7.46	7.31, 7.60	7.39	7.12, 7.65	6.79	6.51, 7.07	7.89	7.59, 8.20	6.94	6.68, 7.20	6.60	6.17, 7.04
Japan	5.75	5.70, 5.80	5.92	5.80, 6.04	6.49	6.40, 6.57	6.08	5.90, 6.26	6.39	6.27, 6.50	4.24	4.06, 4.41
Kenya	5.69	5.48, 5.89	5.39	5.25, 5.54	5.48	4.88, 6.07	6.03	5.85, 6.22	5.51	5.28, 5.73	5.38	5.21, 5.55
Mexico	7.25	7.14, 7.37	7.04	6.89, 7.18	7.60	7.34, 7.86	6.97	6.66, 7.28	7.07	6.91, 7.23	6.59	6.35, 6.82
Nigeria	5.97	5.71, 6.22	5.68	5.53, 5.84	5.99	5.25, 6.72	5.75	5.51, 6.00	5.64	5.26, 6.03	5.53	5.21, 5.86
Philippines	6.41	6.24, 6.58	6.34	6.17, 6.50	6.44	5.88, 7.00	6.51	6.30, 6.71	6.33	6.18, 6.49	6.36	6.10, 6.61
Poland	7.14	7.05, 7.23	7.26	7.12, 7.39	7.20	7.04, 7.37	7.24	6.92, 7.56	6.94	6.59, 7.30	6.26	5.76, 6.75
South Africa	6.53	6.25, 6.81	6.13	5.78, 6.48	5.94	5.24, 6.64	6.37	5.96, 6.78	5.72	4.96, 6.49	5.89	5.63, 6.15
Spain	6.80	6.73, 6.88	6.74	6.57, 6.90	6.68	6.49, 6.88	6.79	6.57, 7.00	6.44	6.09, 6.79	6.05	5.87, 6.23
Sweden	7.11	7.07, 7.16	7.61	7.49, 7.74	7.72	7.66, 7.79	6.67	6.56, 6.78	6.88	6.36, 7.41	5.26	5.02, 5.50
Tanzania	4.76	4.46, 5.06	4.36	4.15, 4.56	3.53	2.88, 4.17	5.37	5.03, 5.71	4.34	4.10, 4.57	4.49	4.24, 4.73
Türkiye	5.15	4.90, 5.40	5.22	4.87, 5.58	5.66	5.12, 6.21	5.34	5.04, 5.63	5.10	4.66, 5.55	4.53	3.83, 5.22
UK	6.60	6.50, 6.70	6.52	6.26, 6.77	6.91	6.76, 7.07	6.41	6.10, 6.73	6.70	6.26, 7.14	5.48	4.96, 6.01
US	6.88	6.83, 6.94	6.95	6.74, 7.15	7.66	7.60, 7.72	6.11	5.70, 6.53	6.91	6.69, 7.14	5.33	4.89, 5.77

Table S26b. Mean levels of life satisfaction by employment status across 22 countries

Country	Employed for employer		Self employed		Retired		Student		Homemaker		Unemployed	
	Mean	95% CIs	Mean	95% CIs	Mean	95% CIs	Mean	95% CIs	Mean	95% CIs	Mean	95% CIs
Argentina	7.31	7.19, 7.44	7.35	7.19, 7.50	7.28	7.00, 7.56	6.90	6.61, 7.20	7.41	7.11, 7.70	6.50	6.17, 6.84
Australia	6.66	6.55, 6.77	6.98	6.72, 7.23	7.49	7.34, 7.64	6.06	5.60, 6.52	6.62	6.14, 7.11	5.02	4.38, 5.65
Brazil	7.38	7.29, 7.47	7.25	7.14, 7.36	7.79	7.60, 7.98	6.98	6.75, 7.20	7.18	6.97, 7.38	6.34	6.19, 6.49
Egypt	7.56	7.34, 7.77	7.72	7.48, 7.96	7.43	6.95, 7.91	7.90	7.52, 8.28	7.83	7.67, 7.98	7.19	6.72, 7.67
Germany	7.01	6.94, 7.08	7.10	6.92, 7.28	7.06	6.95, 7.16	6.69	6.47, 6.92	6.46	6.11, 6.81	5.43	5.09, 5.77
Hong Kong	7.07	6.96, 7.17	7.36	6.90, 7.83	7.20	6.88, 7.52	6.65	6.11, 7.20	6.70	6.11, 7.29	5.54	4.77, 6.31
India	6.71	6.54, 6.89	7.14	6.97, 7.30	6.97	6.42, 7.52	7.20	6.86, 7.54	7.17	7.04, 7.30	6.62	6.30, 6.94
Indonesia	7.82	7.63, 8.01	8.09	7.97, 8.22	8.45	8.07, 8.82	7.72	7.45, 8.00	8.17	8.05, 8.28	7.46	7.15, 7.78
Israel	7.54	7.37, 7.71	7.29	6.96, 7.61	7.03	6.69, 7.36	8.19	7.91, 8.47	7.51	7.10, 7.92	6.80	6.39, 7.22
Japan	5.87	5.82, 5.93	5.99	5.85, 6.12	6.69	6.61, 6.78	6.25	6.04, 6.45	6.54	6.42, 6.66	4.27	4.08, 4.46
Kenya	5.96	5.76, 6.15	5.98	5.83, 6.14	6.22	5.63, 6.80	6.23	6.01, 6.45	6.29	6.05, 6.53	5.69	5.52, 5.86
Mexico	7.85	7.73, 7.98	7.92	7.77, 8.08	8.37	8.16, 8.58	7.52	7.20, 7.85	8.00	7.85, 8.15	7.19	6.96, 7.43
Nigeria	6.54	6.29, 6.79	6.58	6.43, 6.74	6.63	5.75, 7.52	6.55	6.25, 6.84	6.47	6.07, 6.87	6.11	5.75, 6.48
Philippines	7.64	7.48, 7.81	7.39	7.21, 7.57	7.77	7.22, 8.32	7.58	7.39, 7.78	7.57	7.41, 7.72	7.28	7.01, 7.55
Poland	7.59	7.49, 7.69	7.61	7.45, 7.77	7.35	7.17, 7.52	8.19	7.90, 8.48	7.51	7.14, 7.88	6.61	6.02, 7.21
South Africa	6.73	6.45, 7.01	6.45	6.11, 6.80	6.57	5.89, 7.24	6.54	6.09, 6.99	6.17	5.45, 6.90	6.04	5.82, 6.26
Spain	6.91	6.83, 7.00	6.77	6.58, 6.97	6.81	6.59, 7.04	6.74	6.49, 6.99	6.76	6.40, 7.13	6.25	6.04, 6.46
Sweden	6.97	6.92, 7.02	7.50	7.35, 7.64	7.76	7.69, 7.83	6.52	6.39, 6.66	7.07	6.56, 7.59	4.83	4.55, 5.11
Tanzania	5.85	5.44, 6.26	5.16	4.96, 5.35	5.45	4.73, 6.17	6.37	5.96, 6.78	5.80	5.53, 6.07	5.10	4.78, 5.41
Türkiye	5.21	4.86, 5.57	5.20	4.72, 5.67	5.30	4.53, 6.06	4.85	4.39, 5.31	5.55	5.06, 6.05	4.18	3.39, 4.97
UK	6.55	6.43, 6.66	6.39	6.07, 6.70	6.93	6.76, 7.11	6.11	5.71, 6.52	6.63	6.14, 7.12	5.36	4.76, 5.97
US	6.79	6.72, 6.86	7.02	6.86, 7.19	7.60	7.52, 7.68	5.75	5.16, 6.34	6.81	6.56, 7.07	5.09	4.60, 5.59

Table S26c. Mean levels of happiness by employment status across 22 countries

Country	Employed for employer		Self employed		Retired		Student		Homemaker		Unemployed	
	Mean	95% CIs	Mean	95% CIs	Mean	95% CIs	Mean	95% CIs	Mean	95% CIs	Mean	95% CIs
Argentina	7.48	7.37, 7.59	7.49	7.34, 7.63	7.15	6.89, 7.41	7.07	6.79, 7.35	7.59	7.34, 7.84	6.88	6.57, 7.19
Australia	6.80	6.70, 6.89	7.11	6.90, 7.32	7.61	7.48, 7.74	6.17	5.80, 6.54	6.71	6.27, 7.16	5.71	5.16, 6.26
Brazil	7.51	7.43, 7.59	7.44	7.33, 7.54	7.82	7.66, 7.99	7.23	7.03, 7.44	7.40	7.22, 7.58	6.65	6.51, 6.79
Egypt	6.06	5.82, 6.30	6.03	5.77, 6.29	6.30	5.79, 6.80	6.40	6.10, 6.70	6.29	6.13, 6.46	6.04	5.58, 6.50
Germany	6.97	6.91, 7.04	7.09	6.92, 7.25	6.98	6.88, 7.08	6.68	6.48, 6.89	6.46	6.10, 6.81	5.81	5.48, 6.14
Hong Kong	7.23	7.13, 7.32	7.70	7.37, 8.03	7.12	6.84, 7.41	6.64	6.10, 7.17	6.94	6.40, 7.48	5.68	4.84, 6.53
India	6.18	6.01, 6.35	6.70	6.54, 6.85	6.68	6.14, 7.22	7.00	6.68, 7.32	6.47	6.34, 6.61	6.25	5.96, 6.54
Indonesia	7.92	7.75, 8.09	8.08	7.96, 8.21	7.91	7.47, 8.36	7.74	7.46, 8.02	8.28	8.16, 8.40	7.51	7.17, 7.85
Israel	7.84	7.71, 7.96	7.82	7.54, 8.11	7.39	7.17, 7.61	8.24	7.94, 8.54	7.37	7.07, 7.66	6.99	6.52, 7.45
Japan	6.05	6.00, 6.10	6.27	6.15, 6.40	6.72	6.64, 6.80	6.29	6.11, 6.48	6.68	6.56, 6.79	4.65	4.47, 4.83
Kenya	7.12	6.95, 7.29	7.17	7.03, 7.31	7.12	6.57, 7.66	7.58	7.41, 7.75	7.48	7.26, 7.69	7.29	7.16, 7.43
Mexico	7.84	7.73, 7.96	7.76	7.61, 7.92	8.06	7.81, 8.31	7.49	7.20, 7.78	7.98	7.83, 8.12	7.30	7.07, 7.52
Nigeria	7.24	7.03, 7.45	7.09	6.95, 7.24	7.51	6.73, 8.28	7.07	6.85, 7.28	6.87	6.50, 7.23	6.93	6.60, 7.26
Philippines	7.38	7.21, 7.55	7.35	7.19, 7.51	7.51	6.95, 8.08	7.31	7.13, 7.49	7.34	7.19, 7.49	7.20	6.95, 7.45
Poland	7.61	7.51, 7.71	7.68	7.54, 7.82	7.43	7.28, 7.58	8.13	7.88, 8.38	7.37	6.92, 7.83	6.88	6.43, 7.34
South Africa	7.22	6.99, 7.44	6.85	6.46, 7.23	7.16	6.44, 7.88	7.16	6.76, 7.55	6.90	6.27, 7.53	6.74	6.52, 6.95
Spain	7.07	6.99, 7.15	6.87	6.69, 7.06	6.91	6.71, 7.12	6.69	6.46, 6.92	6.99	6.70, 7.28	6.47	6.27, 6.67
Sweden	6.93	6.88, 6.98	7.39	7.24, 7.53	7.60	7.54, 7.67	6.44	6.31, 6.56	6.89	6.32, 7.45	5.19	4.92, 5.47
Tanzania	6.98	6.66, 7.30	6.55	6.40, 6.71	5.87	5.20, 6.55	7.71	7.38, 8.04	6.49	6.26, 6.71	6.53	6.29, 6.77
Türkiye	5.67	5.34, 5.99	5.63	5.17, 6.09	5.73	5.08, 6.37	5.41	5.03, 5.79	5.57	5.11, 6.02	4.45	3.84, 5.06
UK	6.74	6.64, 6.85	6.52	6.23, 6.82	7.18	7.03, 7.33	6.22	5.87, 6.56	6.80	6.36, 7.24	5.79	5.25, 6.33
US	6.93	6.87, 7.00	7.19	7.06, 7.32	7.70	7.63, 7.77	6.01	5.64, 6.39	6.93	6.71, 7.16	5.34	4.94, 5.75

Tables S27a-c. SWB and childhood health across countries

Table S27a. Association between childhood self-rated health and Cantril's Ladder across 22 countries (reference group: Good health)

Country	Excellent			Very good			Fair			Poor		
	Est	95% CIs	E-value	Est	95% CIs	E-value	Est	95% CIs	E-value	Est	95% CIs	E-value
Argentina	0.35	0.16, 0.55	1.59	0.16	-0.04, 0.35	1.34	-0.05	-0.39, 0.28	1.17	-0.56	-1.34, 0.22	1.84
Australia	0.45	0.25, 0.65	1.83	0.08	-0.13, 0.30	1.26	-0.27	-0.60, 0.06	1.56	-0.43	-1.10, 0.23	1.80
Brazil	0.26	0.12, 0.40	1.46	0.07	-0.07, 0.21	1.19	-0.42	-0.63, -0.20	1.64	-0.57	-1.08, -0.07	1.82
Egypt	0.10	-0.21, 0.42	1.22	-0.04	-0.35, 0.28	1.12	-0.15	-0.62, 0.31	1.28	-0.27	-0.93, 0.40	1.40
Germany	0.40	0.26, 0.54	1.74	0.17	0.05, 0.29	1.41	-0.30	-0.52, -0.08	1.60	0.45	-0.08, 0.97	1.81
Hong Kong	1.37	1.01, 1.74	3.12	0.69	0.51, 0.87	2.07	-0.60	-0.86, -0.34	1.95	-1.44	-2.22, -0.66	3.23
India	-0.17	-0.41, 0.07	1.26	-0.10	-0.30, 0.11	1.18	-0.45	-0.68, -0.22	1.49	-0.99	-1.46, -0.51	1.89
Indonesia	0.18	-0.07, 0.44	1.34	-0.02	-0.21, 0.17	1.08	-0.18	-0.40, 0.04	1.33	-0.19	-1.37, 1.00	1.34
Israel	0.51	0.23, 0.78	1.90	0.34	0.08, 0.60	1.66	0.00	-0.49, 0.49	1.03	0.38	-0.83, 1.60	1.72
Japan	0.98	0.86, 1.09	2.40	0.46	0.39, 0.54	1.73	-0.47	-0.57, -0.38	1.74	-0.91	-1.12, -0.70	2.30
Kenya	0.23	0.01, 0.44	1.33	0.25	0.03, 0.48	1.35	-0.03	-0.32, 0.26	1.10	-0.18	-0.73, 0.37	1.28
Mexico	0.50	0.32, 0.68	1.78	0.47	0.29, 0.65	1.75	0.07	-0.20, 0.33	1.20	0.10	-0.45, 0.65	1.26
Nigeria	0.26	-0.06, 0.58	1.40	0.02	-0.26, 0.30	1.09	-0.26	-0.77, 0.24	1.40	-0.13	-1.07, 0.81	1.25
Philippines	0.17	-0.05, 0.39	1.34	-0.05	-0.32, 0.23	1.15	-0.14	-0.34, 0.07	1.29	-0.32	-0.71, 0.06	1.51
Poland	0.53	0.35, 0.71	2.03	0.27	0.11, 0.43	1.61	-0.34	-0.65, -0.03	1.72	0.41	-0.31, 1.12	1.82
South Africa	-0.13	-0.54, 0.29	1.25	0.02	-0.51, 0.54	1.09	-0.51	-1.14, 0.12	1.63	-0.65	-1.30, 0.00	1.77
Spain	0.38	0.21, 0.56	1.69	0.22	0.05, 0.39	1.46	-0.14	-0.56, 0.29	1.33	-0.12	-0.73, 0.50	1.30
Sweden	0.93	0.83, 1.03	2.62	0.47	0.38, 0.57	1.87	-0.19	-0.34, -0.04	1.44	-0.43	-0.70, -0.17	1.81
Tanzania	0.14	-0.11, 0.39	1.24	0.33	0.07, 0.59	1.42	-0.02	-0.32, 0.27	1.08	-0.56	-1.00, -0.12	1.61
Türkiye	0.14	-0.30, 0.59	1.29	0.51	0.04, 0.98	1.68	0.15	-0.41, 0.70	1.29	-0.61	-1.75, 0.52	1.79
UK	0.37	0.16, 0.57	1.64	0.33	0.13, 0.54	1.60	-0.40	-0.75, -0.05	1.69	-0.59	-1.20, 0.02	1.94
US	0.72	0.58, 0.86	2.20	0.40	0.25, 0.54	1.73	-0.20	-0.48, 0.08	1.44	-0.44	-1.08, 0.21	1.78

Table S27b. Association between childhood self-rated health and life satisfaction across 22 countries (reference group: Good health)

Country	Excellent			Very good			Fair			Poor		
	Est	95% CIs	E-value	Est	95% CIs	E-value	Est	95% CIs	E-value	Est	95% CIs	E-value
Argentina	0.34	0.13, 0.55	1.54	0.09	-0.12, 0.30	1.22	-0.21	-0.61, 0.20	1.38	-0.90	-1.74, -0.06	2.17
Australia	0.67	0.42, 0.92	2.01	0.21	-0.05, 0.47	1.41	-0.34	-0.78, 0.09	1.59	-0.33	-1.02, 0.35	1.58
Brazil	0.31	0.17, 0.45	1.49	0.00	-0.15, 0.15	1.01	-0.47	-0.70, -0.24	1.67	-0.75	-1.33, -0.17	1.96
Egypt	-0.07	-0.34, 0.19	1.18	-0.27	-0.60, 0.06	1.41	-0.07	-0.50, 0.37	1.17	-0.07	-0.77, 0.63	1.17
Germany	0.60	0.44, 0.76	1.92	0.32	0.19, 0.45	1.57	-0.44	-0.69, -0.20	1.72	0.43	-0.07, 0.93	1.71
Hong Kong	1.50	1.13, 1.87	3.26	0.75	0.53, 0.97	2.12	-0.72	-1.01, -0.44	2.08	-1.14	-1.92, -0.36	2.68
India	0.03	-0.21, 0.26	1.10	0.07	-0.11, 0.25	1.16	-0.31	-0.53, -0.08	1.38	-0.55	-0.99, -0.11	1.58
Indonesia	0.27	0.06, 0.49	1.47	0.06	-0.09, 0.22	1.19	-0.23	-0.43, -0.03	1.42	-0.49	-1.76, 0.78	1.72
Israel	0.54	0.21, 0.86	1.87	0.42	0.14, 0.69	1.71	-0.19	-0.71, 0.34	1.40	0.15	-0.93, 1.23	1.35
Japan	1.10	0.98, 1.22	2.46	0.51	0.43, 0.59	1.75	-0.51	-0.61, -0.40	1.74	-0.95	-1.18, -0.72	2.26
Kenya	0.08	-0.13, 0.29	1.17	0.08	-0.15, 0.30	1.16	-0.24	-0.51, 0.03	1.33	-0.62	-1.18, -0.06	1.63
Mexico	0.30	0.12, 0.48	1.53	0.23	0.05, 0.41	1.44	-0.10	-0.37, 0.17	1.25	-0.13	-0.71, 0.45	1.30
Nigeria	0.20	-0.13, 0.54	1.34	0.03	-0.32, 0.38	1.11	-0.10	-0.57, 0.36	1.22	-0.02	-0.88, 0.84	1.08
Philippines	0.14	-0.06, 0.34	1.30	0.15	-0.12, 0.42	1.31	-0.54	-0.76, -0.31	1.74	-0.50	-0.93, -0.08	1.71
Poland	0.70	0.51, 0.89	2.25	0.27	0.11, 0.44	1.57	-0.29	-0.68, 0.10	1.60	-0.04	-0.85, 0.77	1.17
South Africa	0.12	-0.29, 0.53	1.24	-0.03	-0.51, 0.46	1.10	-0.42	-1.05, 0.20	1.56	-0.11	-0.74, 0.52	1.23
Spain	0.46	0.25, 0.67	1.72	0.29	0.08, 0.49	1.51	-0.19	-0.68, 0.29	1.38	0.34	-0.32, 1.01	1.58
Sweden	1.06	0.95, 1.18	2.57	0.54	0.43, 0.66	1.86	-0.22	-0.40, -0.04	1.43	-0.72	-1.06, -0.39	2.09
Tanzania	0.21	-0.06, 0.47	1.28	0.16	-0.13, 0.46	1.24	-0.03	-0.36, 0.29	1.10	-0.36	-0.87, 0.16	1.40
Türkiye	-0.07	-0.61, 0.48	1.16	0.25	-0.32, 0.81	1.35	-0.44	-1.10, 0.23	1.51	-2.64	-3.64, -1.65	3.62
UK	0.57	0.32, 0.81	1.81	0.66	0.43, 0.89	1.92	-0.52	-0.94, -0.11	1.76	-0.52	-1.28, 0.25	1.75
US	0.77	0.59, 0.94	2.11	0.38	0.20, 0.56	1.63	-0.19	-0.55, 0.17	1.39	-0.53	-1.30, 0.23	1.82

Table S27c. Association between childhood self-rated health and happiness across 22 countries (reference group: Good health)

Country	Excellent			Very good			Fair			Poor		
	Est	95% CIs	E-value	Est	95% CIs	E-value	Est	95% CIs	E-value	Est	95% CIs	E-value
Argentina	0.39	0.19, 0.59	1.64	0.04	-0.15, 0.24	1.16	-0.12	-0.48, 0.23	1.29	-0.74	-1.54, 0.07	2.08
Australia	0.57	0.35, 0.78	1.99	0.16	-0.06, 0.39	1.39	-0.17	-0.53, 0.19	1.40	-0.41	-0.93, 0.11	1.75
Brazil	0.41	0.27, 0.54	1.63	0.07	-0.07, 0.22	1.20	-0.33	-0.54, -0.11	1.53	-0.18	-0.67, 0.30	1.36
Egypt	0.05	-0.26, 0.37	1.15	-0.02	-0.34, 0.30	1.09	-0.41	-0.87, 0.05	1.53	-0.20	-0.94, 0.55	1.32
Germany	0.68	0.53, 0.82	2.10	0.35	0.23, 0.48	1.65	-0.35	-0.58, -0.12	1.64	0.37	-0.15, 0.88	1.67
Hong Kong	1.58	1.28, 1.88	3.53	0.81	0.62, 1.01	2.26	-0.74	-1.00, -0.49	2.16	-1.53	-2.42, -0.63	3.42
India	0.06	-0.20, 0.32	1.14	-0.01	-0.19, 0.18	1.04	-0.36	-0.59, -0.13	1.42	-0.65	-1.09, -0.22	1.65
Indonesia	0.33	0.16, 0.51	1.56	0.12	-0.04, 0.28	1.28	-0.15	-0.34, 0.05	1.32	-0.28	-1.43, 0.86	1.50
Israel	0.44	0.20, 0.69	1.87	0.36	0.13, 0.59	1.74	-0.07	-0.44, 0.31	1.23	0.58	-1.51, 2.66	2.09
Japan	1.10	0.99, 1.21	2.57	0.50	0.43, 0.57	1.78	-0.47	-0.57, -0.37	1.74	-0.85	-1.06, -0.64	2.23
Kenya	0.21	0.03, 0.39	1.33	0.15	-0.05, 0.35	1.27	-0.08	-0.32, 0.15	1.19	-0.55	-1.08, -0.03	1.65
Mexico	0.45	0.28, 0.61	1.75	0.28	0.11, 0.45	1.52	-0.33	-0.59, -0.06	1.58	-0.24	-0.81, 0.32	1.48
Nigeria	0.15	-0.13, 0.43	1.29	-0.11	-0.38, 0.17	1.24	-0.18	-0.69, 0.33	1.33	-0.27	-1.00, 0.45	1.44
Philippines	0.28	0.08, 0.47	1.47	0.31	0.08, 0.54	1.51	-0.35	-0.56, -0.15	1.56	-0.42	-0.81, -0.03	1.64
Poland	0.65	0.47, 0.84	2.26	0.30	0.15, 0.46	1.66	-0.38	-0.79, 0.03	1.79	0.43	-0.23, 1.08	1.87
South Africa	0.26	-0.18, 0.70	1.41	0.03	-0.48, 0.54	1.11	0.03	-0.55, 0.62	1.12	-0.32	-0.96, 0.32	1.48
Spain	0.67	0.49, 0.86	2.05	0.35	0.17, 0.53	1.62	-0.03	-0.47, 0.41	1.13	0.27	-0.33, 0.86	1.51
Sweden	1.07	0.96, 1.18	2.67	0.54	0.43, 0.64	1.89	-0.18	-0.34, -0.01	1.39	-0.55	-0.87, -0.24	1.91
Tanzania	0.03	-0.18, 0.25	1.10	0.16	-0.09, 0.40	1.26	-0.01	-0.26, 0.23	1.07	-0.41	-0.82, -0.00	1.49
Türkiye	0.06	-0.43, 0.55	1.16	0.12	-0.38, 0.62	1.24	-0.58	-1.17, 0.01	1.68	-1.98	-2.94, -1.01	3.10
UK	0.61	0.40, 0.82	1.93	0.54	0.34, 0.73	1.84	-0.60	-0.97, -0.22	1.92	-0.50	-1.20, 0.21	1.79
US	0.78	0.62, 0.94	2.25	0.41	0.25, 0.57	1.72	-0.14	-0.43, 0.15	1.34	-0.43	-1.11, 0.26	1.75

Figures S28a-f. Scatter plots for GDP per capita and Gini Index

Figure S28a. Scatter plot of CL and GDP per capita

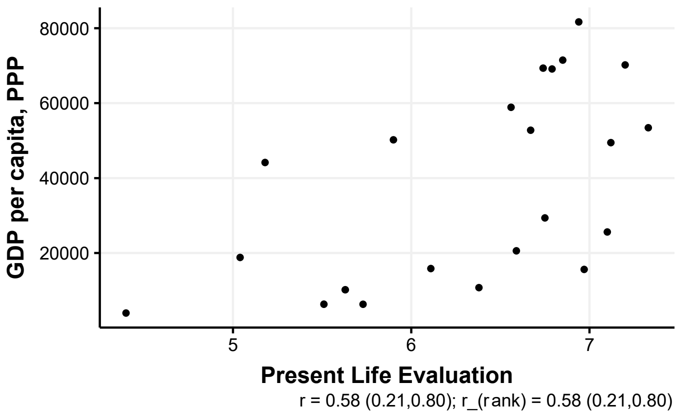

Figure S28b. Scatter plot of CL and Gini index

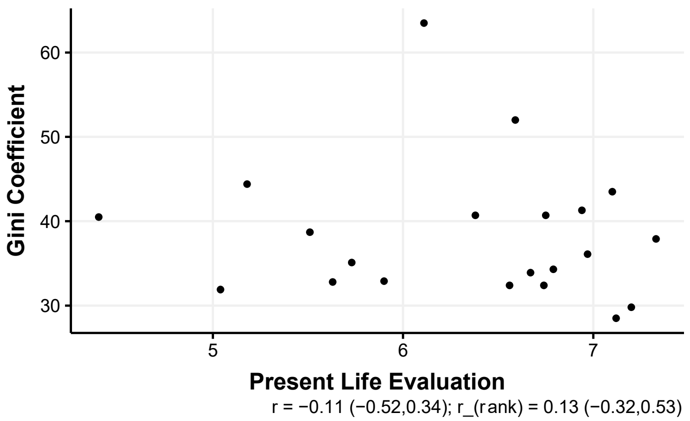

Figure S28c. Scatter plot of LS and GDP per capita

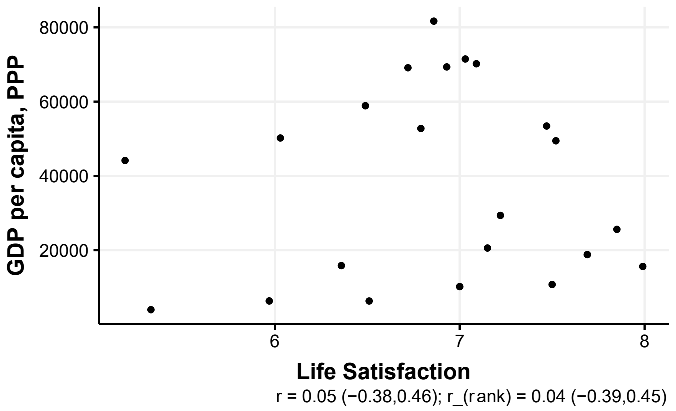

Figure S28d. Scatter plot of LS and Gini index

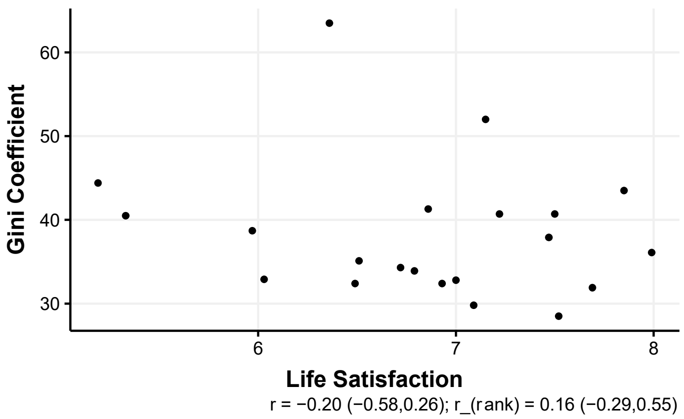

Figure S28e. Scatter plot of H and GDP per capita

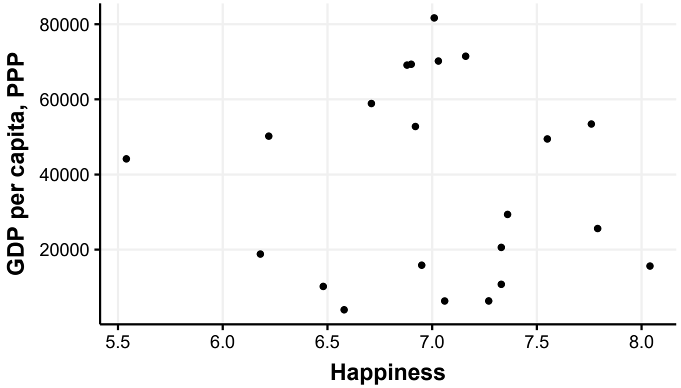

r = −0.08 (−0.48,0.36); r_(rank) = −0.08 (−0.49,0.35)

Figure S28f. Scatter plot of H and Gini index

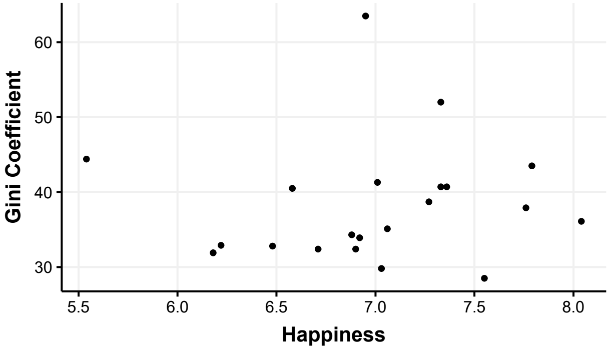

r = 0.07 (−0.38,0.48); r_(rank) = −0.24 (−0.61,0.21)

Figures S29(1-102). Forest plots for demographic variation analyses

The following forest plots are from the imputation-based results. Our pre-registrations were to conduct multiple imputations to account for any missingness; however, the tables reported above are based on the complete-case analyses conducted by reviewer request. We additionally report the imputation-based results to be transparent about the results used to inform our discussion

Cantril's Ladder

Figure S1. Forest plot for `Age group`-`18-24`

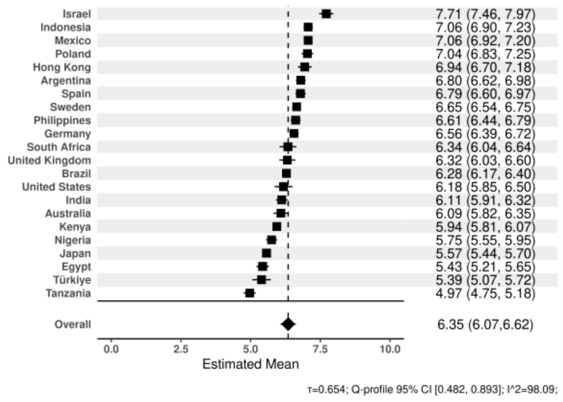

Life Satisfaction

Figure S1. Forest plot for `Age group`-`18-24`

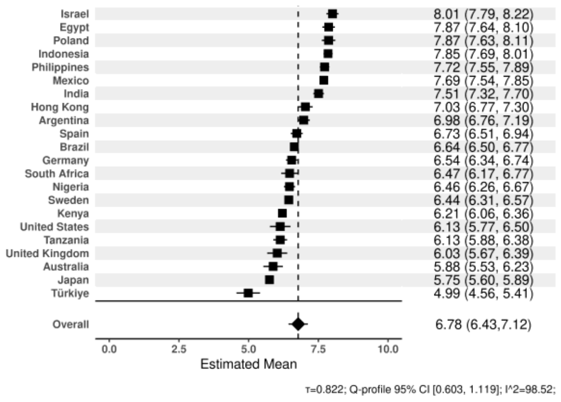

Happiness

Figure S1. Forest plot for `Age group`-`18-24`

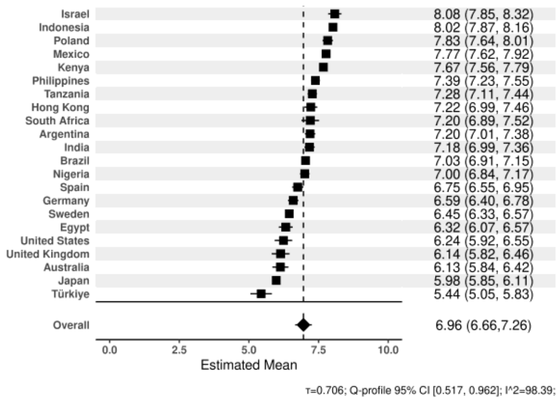

Cantril's Ladder

Figure S2. Forest plot for `Age group`-`25-29`

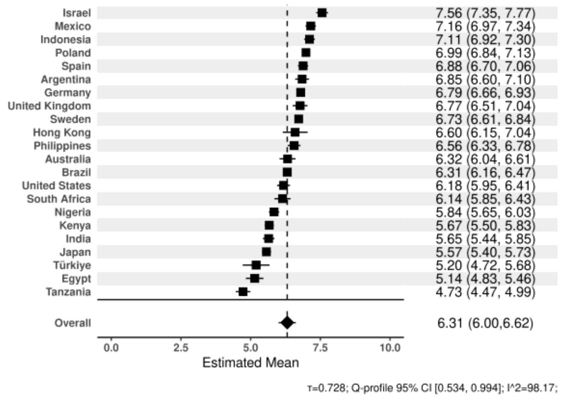

Life Satisfaction

Figure S2. Forest plot for `Age group`-`25-29`

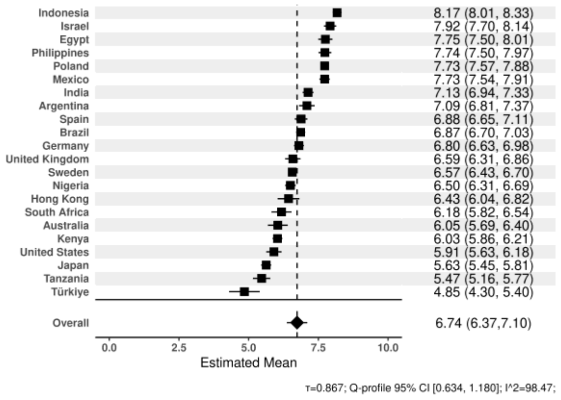

Happiness

Figure S2. Forest plot for `Age group`-`25-29`

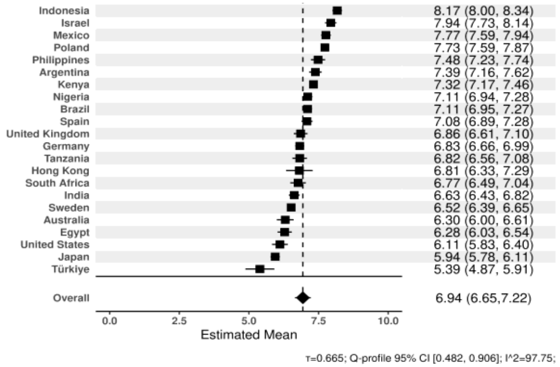

Cantril's Ladder

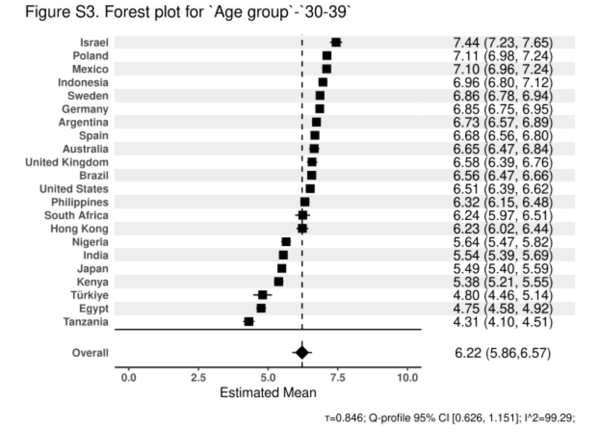

Life Satisfaction

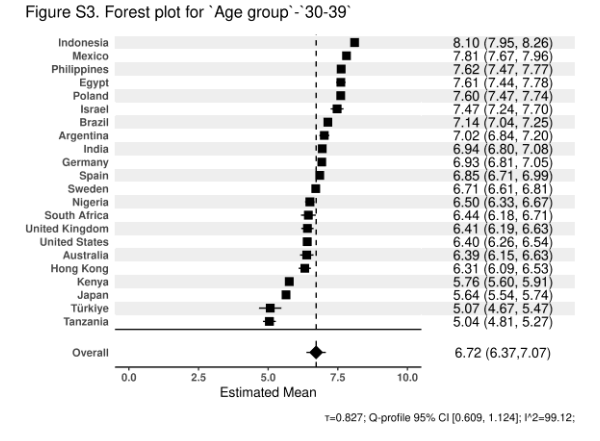

Happiness

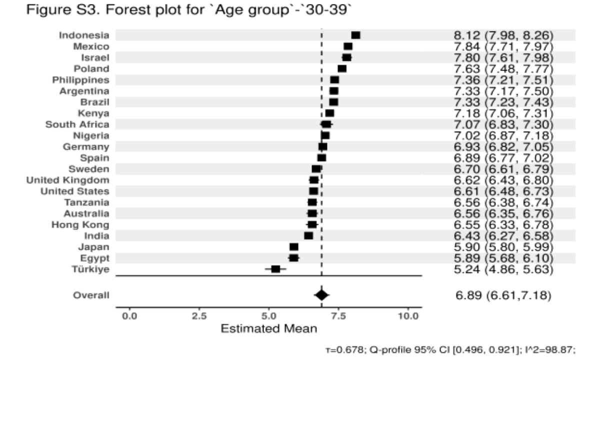

Cantril's Ladder

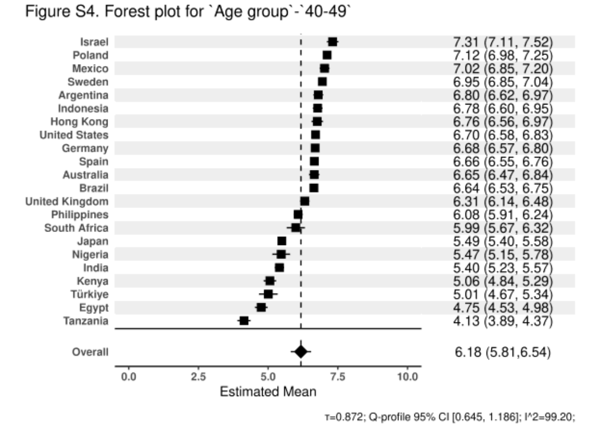

Life Satisfaction

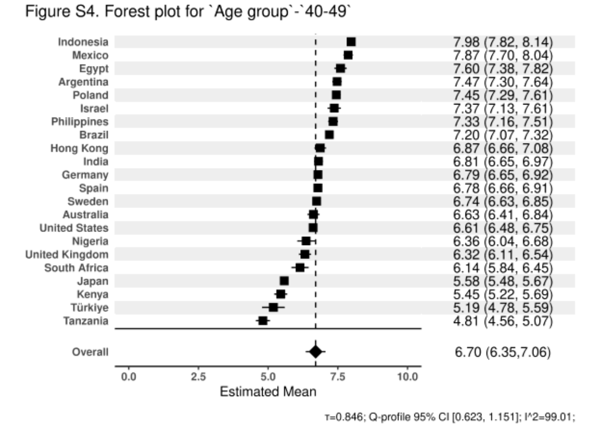

Happiness

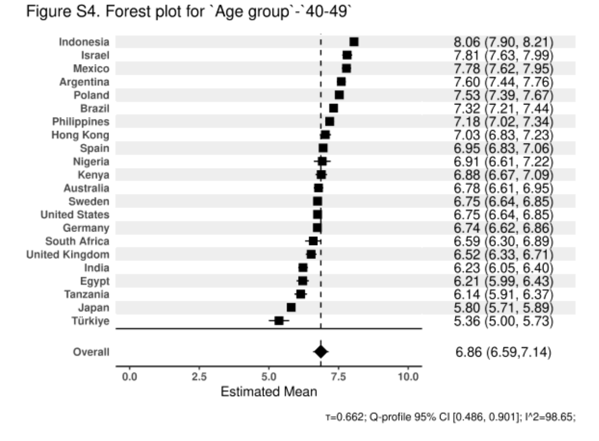

Cantril's Ladder	Life Satisfaction	Happiness

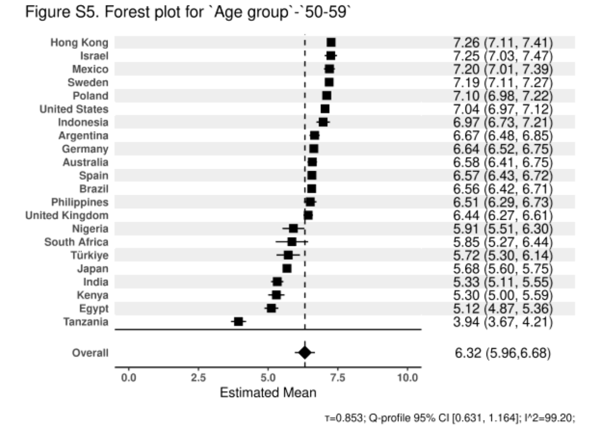

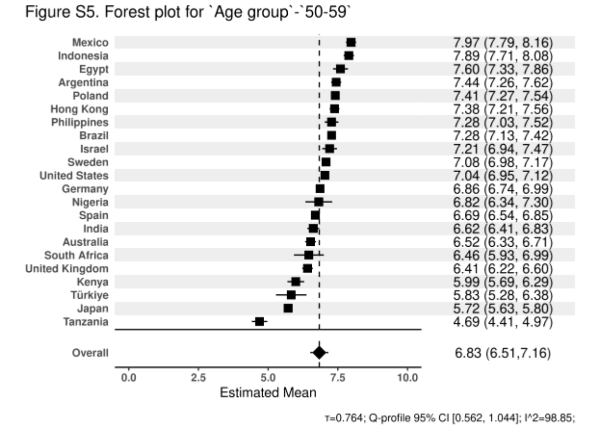

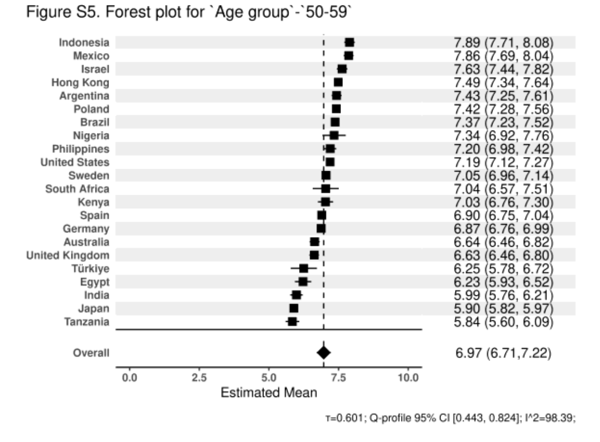

Cantril's Ladder	Life Satisfaction	Happiness

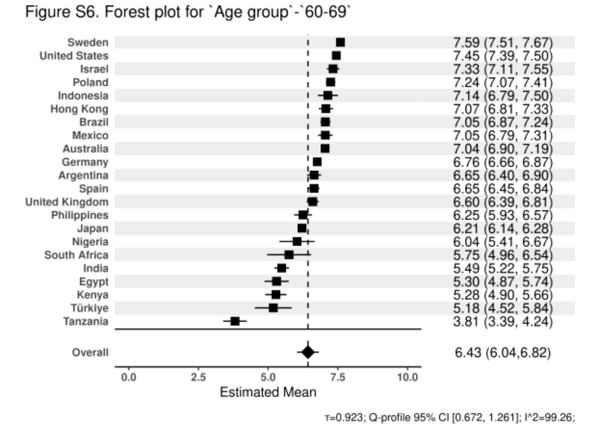

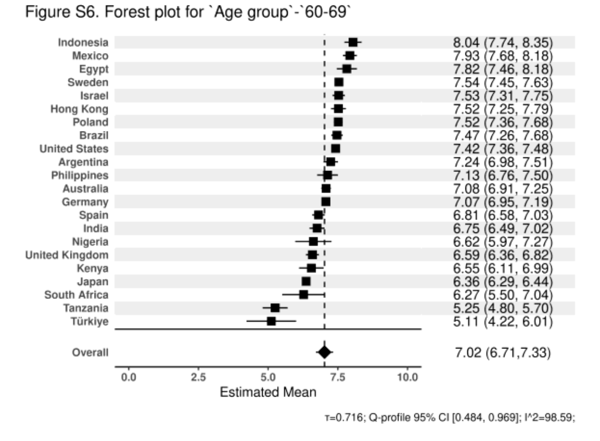

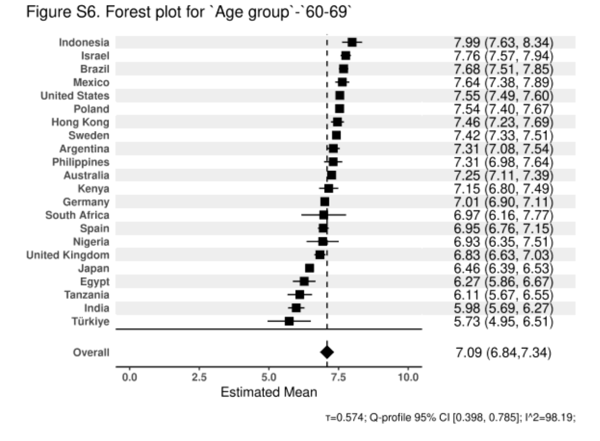

Cantril's Ladder	Life Satisfaction	Happiness

Figure S7. Forest plot for `Age group`-`70-79`

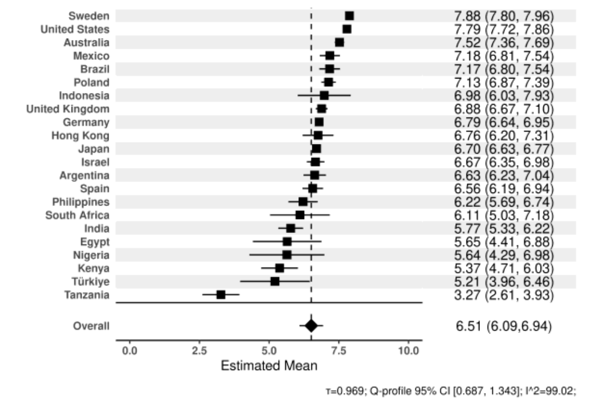

Figure S7. Forest plot for `Age group`-`70-79`

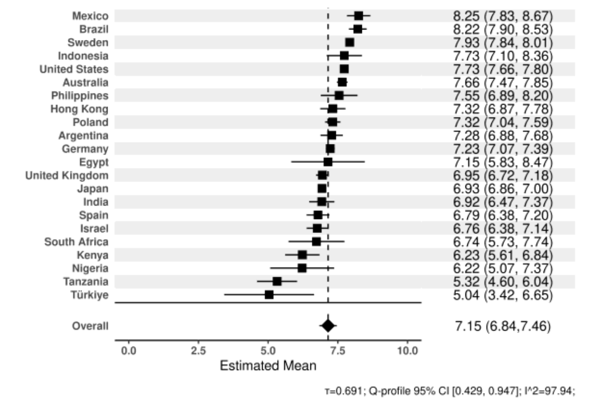

Figure S7. Forest plot for `Age group`-`70-79`

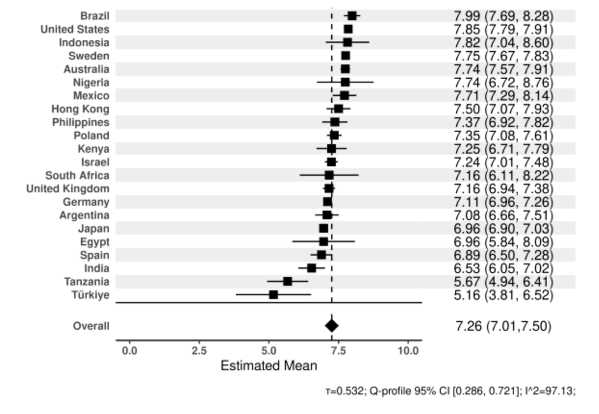

Cantril's Ladder	Life Satisfaction	Happiness

Figure S8. Forest plot for `Age group`-`80 or older`

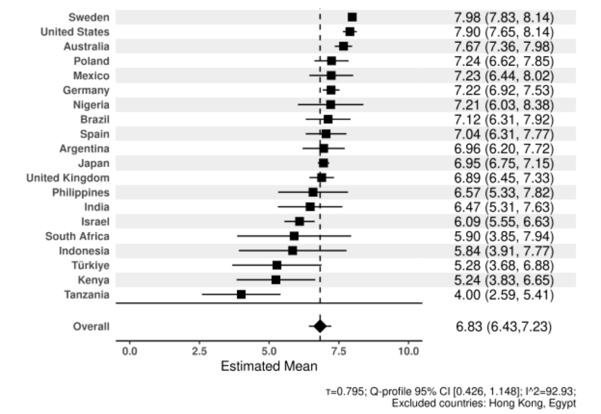

Figure S8. Forest plot for `Age group`-`80 or older`

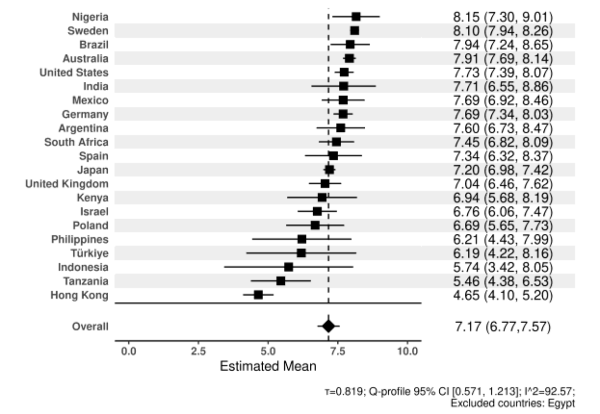

Figure S8. Forest plot for `Age group`-`80 or older`

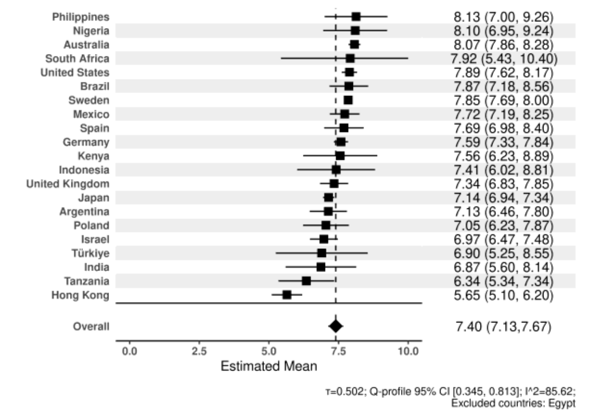

Cantril's Ladder

Figure S9. Forest plot for `Gender`-`Male`

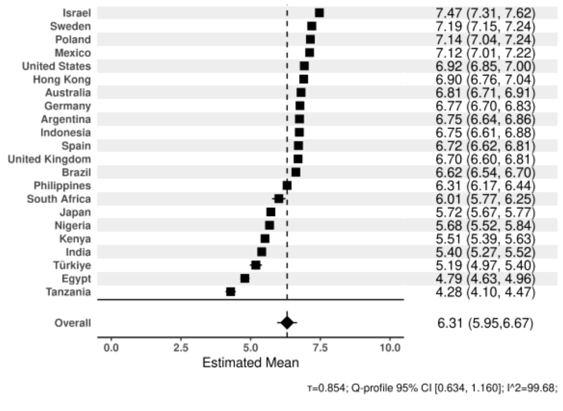

Life Satisfaction

Figure S9. Forest plot for `Gender`-`Male`

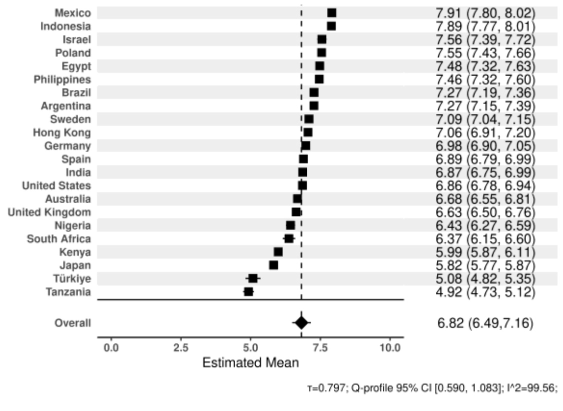

Happiness

Figure S9. Forest plot for `Gender`-`Male`

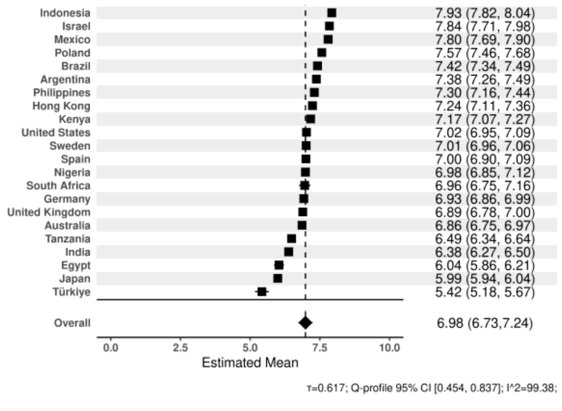

Cantril's Ladder

Figure S10. Forest plot for `Gender`-`Female`

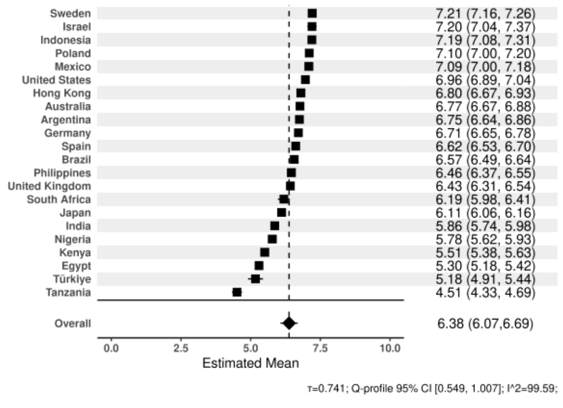

Life Satisfaction

Figure S10. Forest plot for `Gender`-`Female`

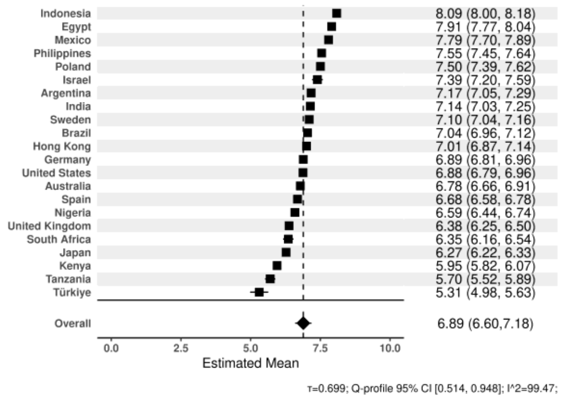

Happiness

Figure S10. Forest plot for `Gender`-`Female`

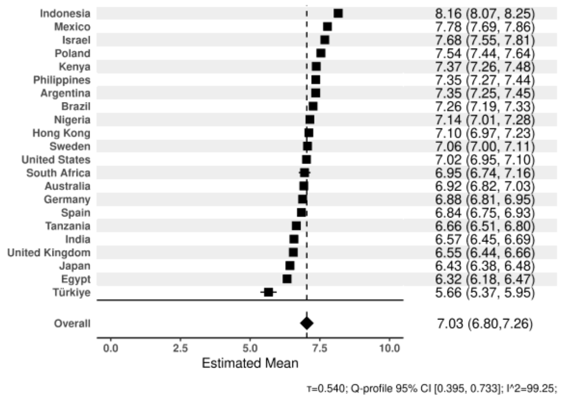

Cantril's Ladder	Life Satisfaction	Happiness

Figure S11. Forest plot for `Gender`-`Other`

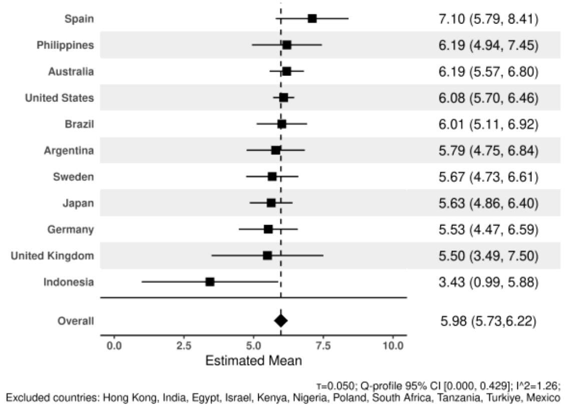

Figure S11. Forest plot for `Gender`-`Other`

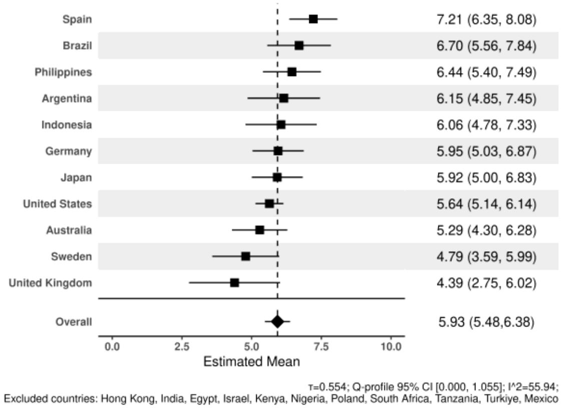

Figure S11. Forest plot for `Gender`-`Other`

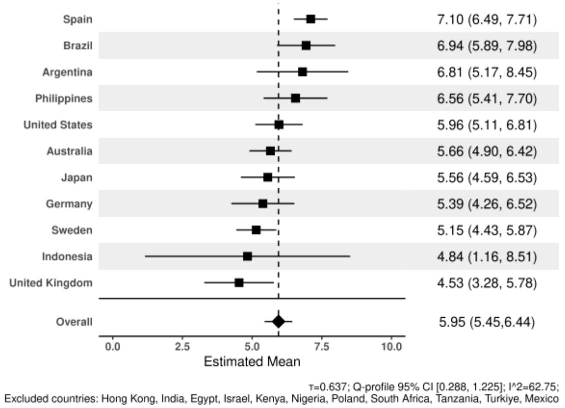

Cantril's Ladder	Life Satisfaction	Happiness

Figure S12. Forest plot for `Marital status`-`Married`

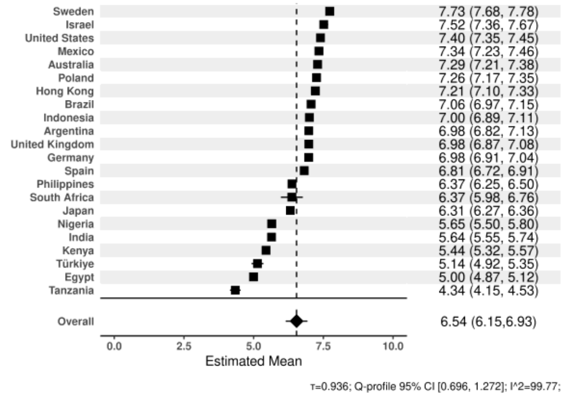

Figure S12. Forest plot for `Marital status`-`Married`

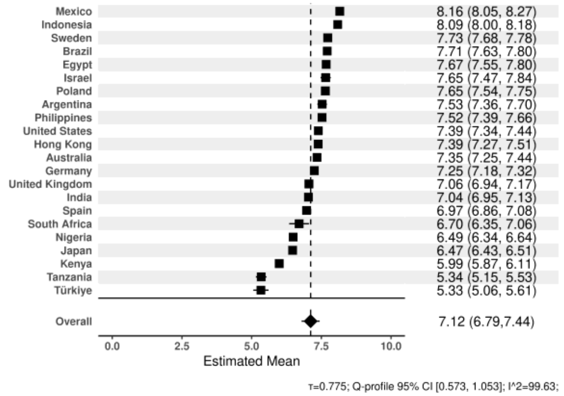

Figure S12. Forest plot for `Marital status`-`Married`

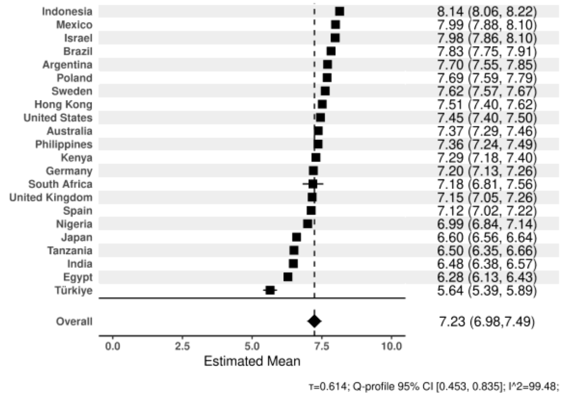

Cantril’s Ladder	Life Satisfaction	Happiness

Figure S13. Forest plot for `Marital status`-`Separated`

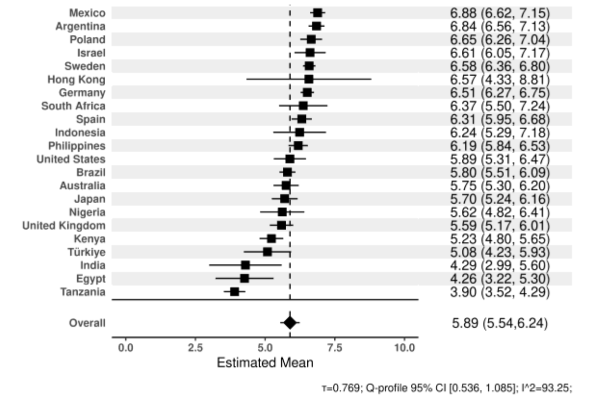

Figure S13. Forest plot for `Marital status`-`Separated`

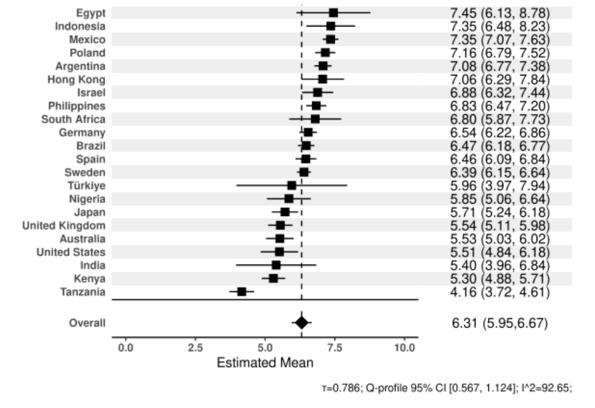

Figure S13. Forest plot for `Marital status`-`Separated`

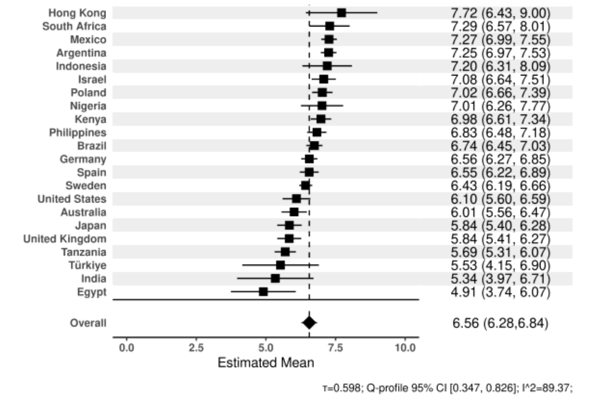

Cantril’s Ladder	Life Satisfaction	Happiness

Figure S14. Forest plot for `Marital status`-`Divorced`

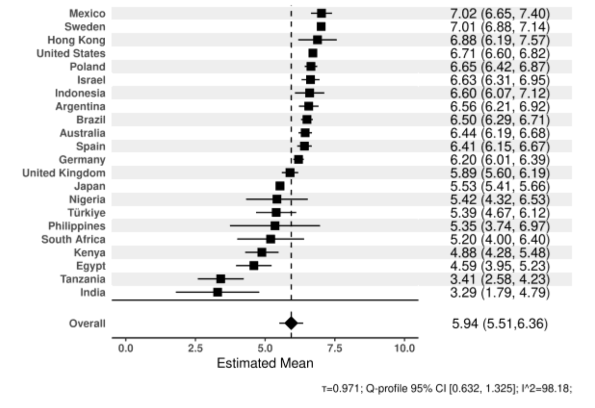

Figure S14. Forest plot for `Marital status`-`Divorced`

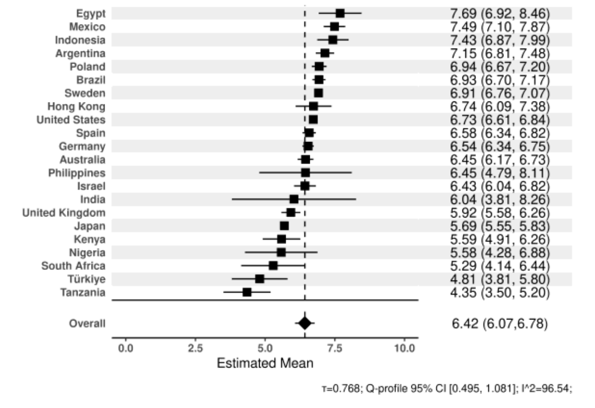

Figure S14. Forest plot for `Marital status`-`Divorced`

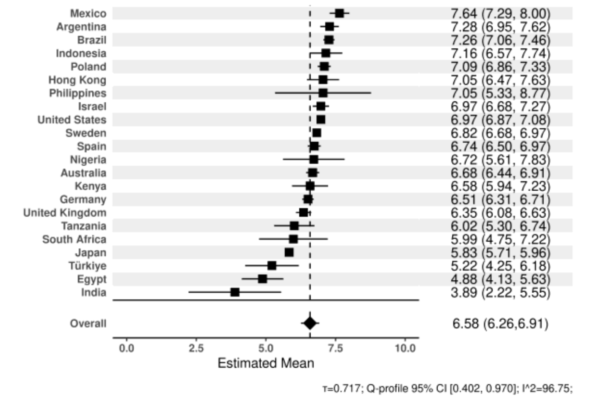

Cantril's Ladder

Figure S15. Forest plot for `Marital status`-`Widowed`

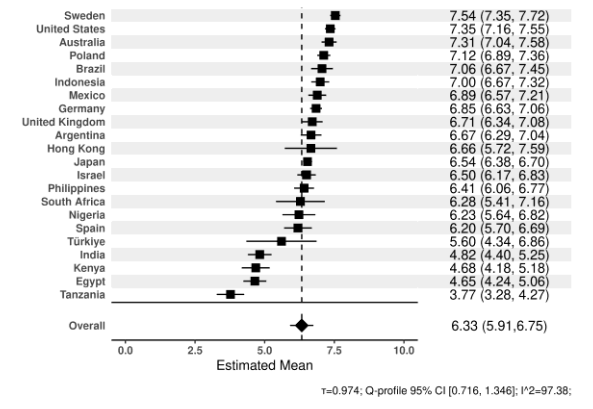

Life Satisfaction

Figure S15. Forest plot for `Marital status`-`Widowed`

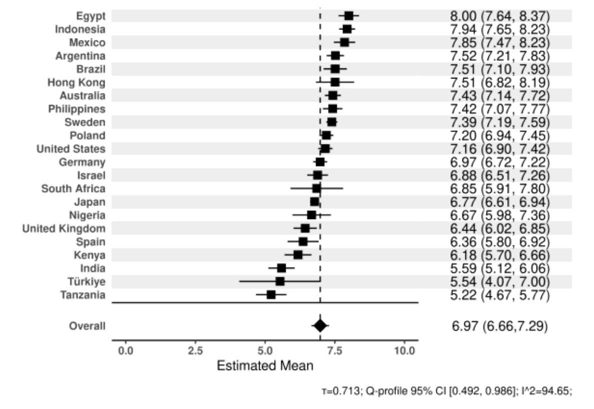

Happiness

Figure S15. Forest plot for `Marital status`-`Widowed`

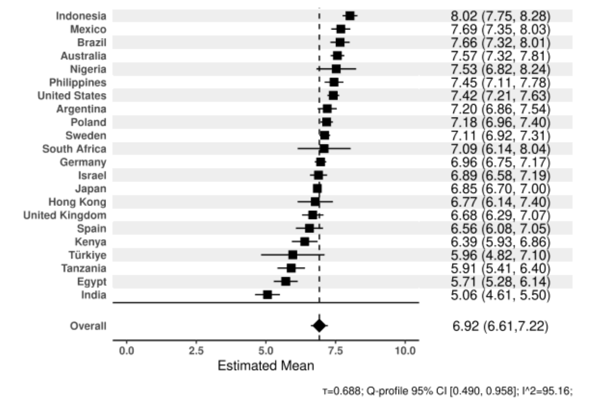

Cantril's Ladder

Figure S16. Forest plot for `Marital status`-`Single, never married`

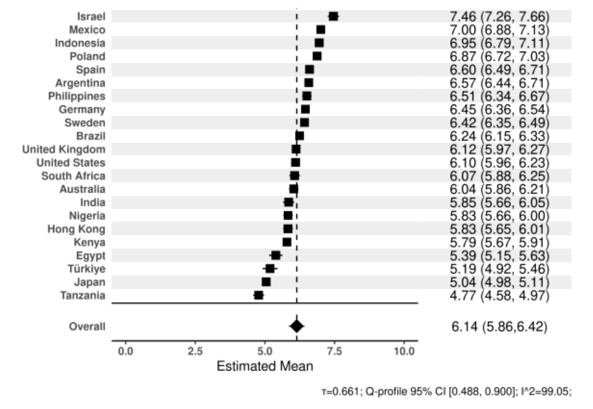

Life Satisfaction

Figure S16. Forest plot for `Marital status`-`Single, never married`

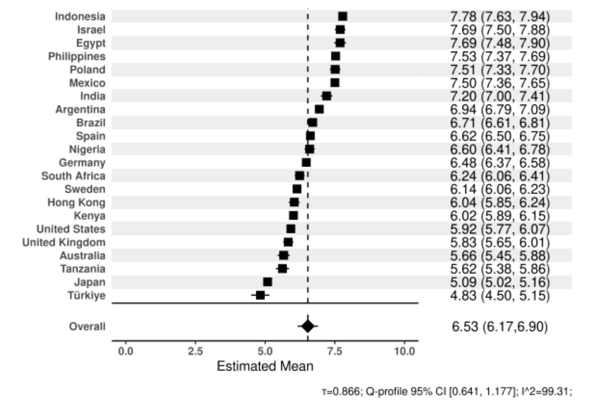

Happiness

Figure S16. Forest plot for `Marital status`-`Single, never married`

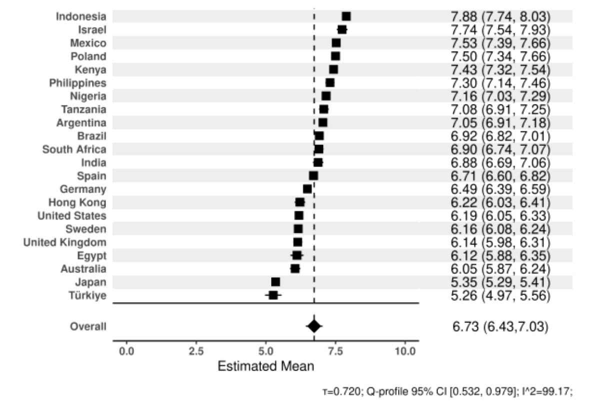

Cantril's Ladder

Figure S17. Forest plot for `Marital status`-`Domestic partner`

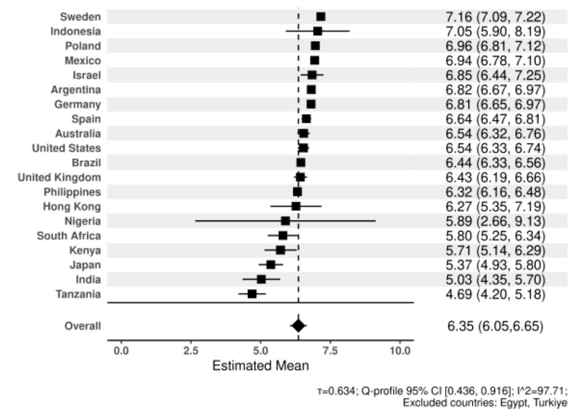

Life Satisfaction

Figure S17. Forest plot for `Marital status`-`Domestic partner`

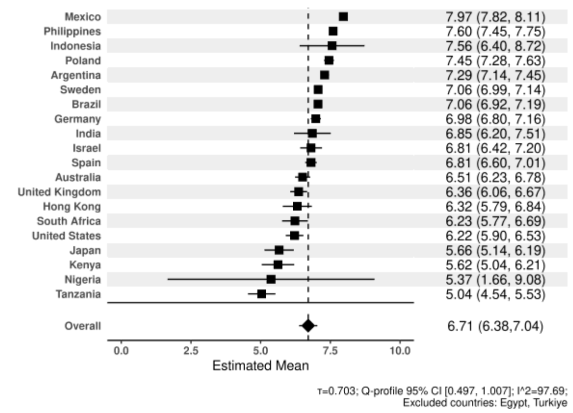

Happiness

Figure S17. Forest plot for `Marital status`-`Domestic partner`

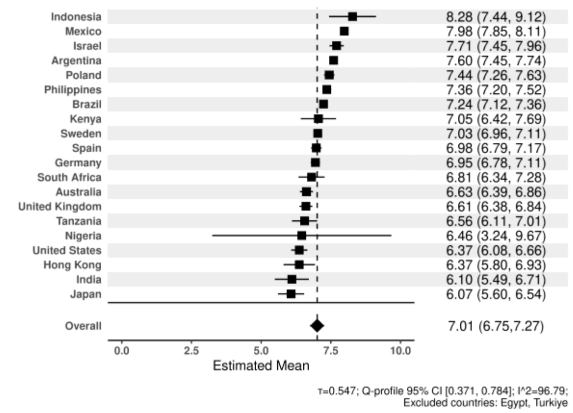

Cantril's Ladder

Figure S18. Forest plot for `Employment status`-`Employed for an employer`

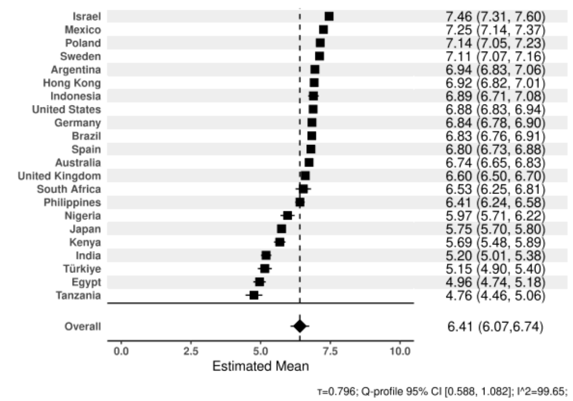

Life Satisfaction

Figure S18. Forest plot for `Employment status`-`Employed for an employer`

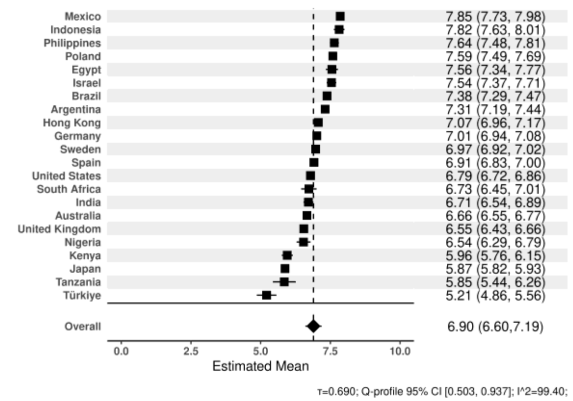

Happiness

Figure S18. Forest plot for `Employment status`-`Employed for an employer`

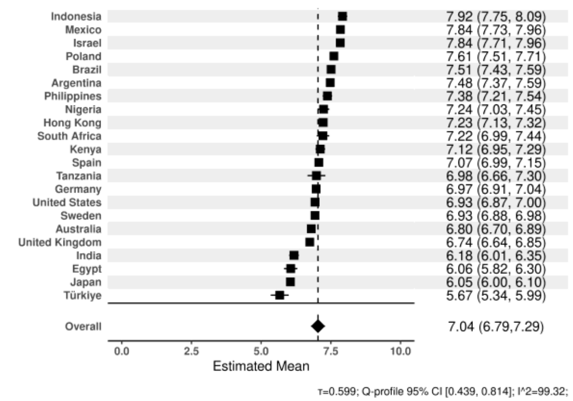

Cantril's Ladder	Life Satisfaction	Happiness

Figure S19. Forest plot for `Employment status`-`Self-employed`

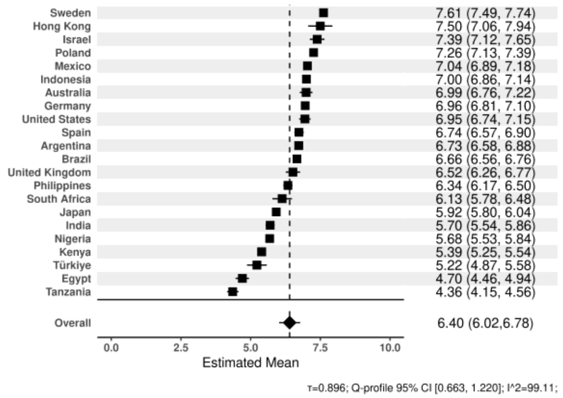

Figure S19. Forest plot for `Employment status`-`Self-employed`

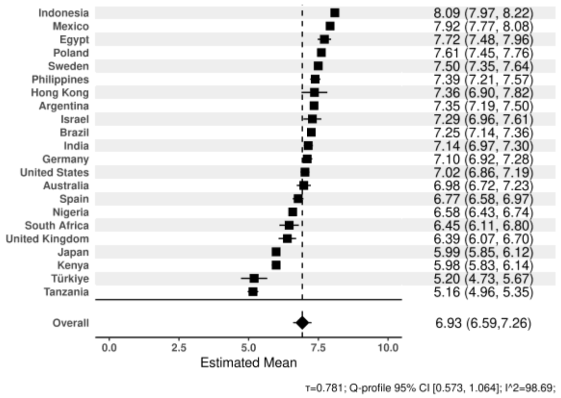

Figure S19. Forest plot for `Employment status`-`Self-employed`

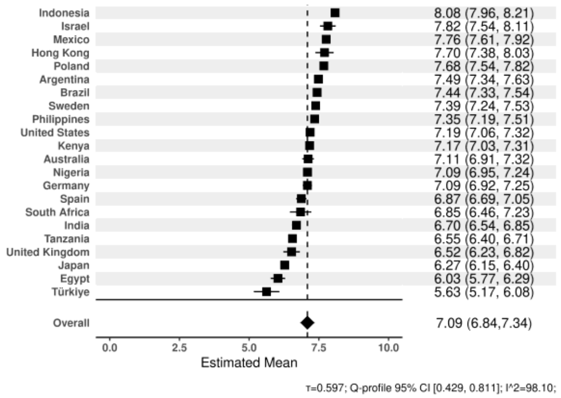

Cantril's Ladder	Life Satisfaction	Happiness

Figure S20. Forest plot for `Employment status`-`Retired`

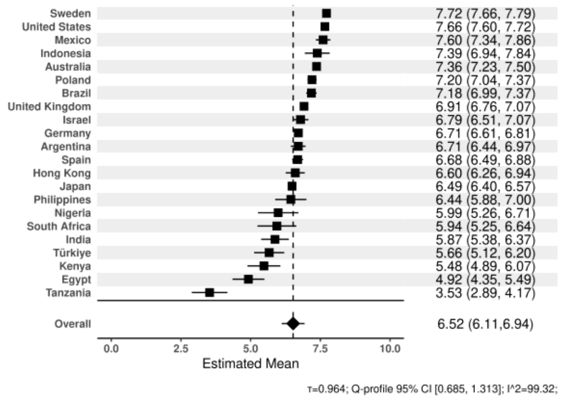

Figure S20. Forest plot for `Employment status`-`Retired`

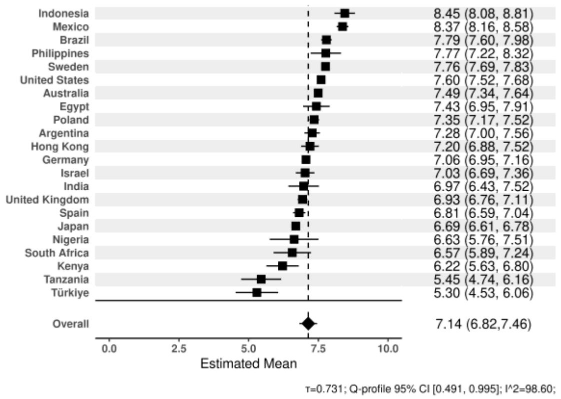

Figure S20. Forest plot for `Employment status`-`Retired`

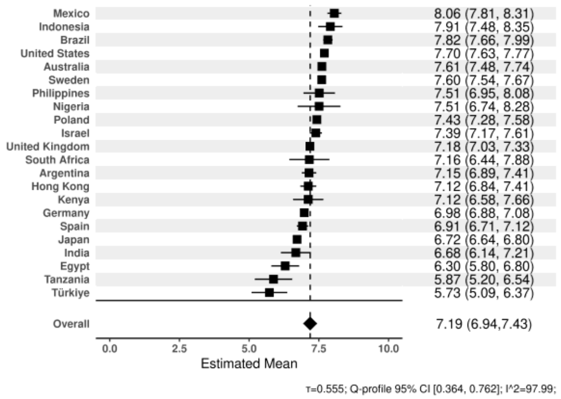

Cantril's Ladder	Life Satisfaction	Happiness

Figure S21. Forest plot for `Employment status`-`Student`

Cantril's Ladder

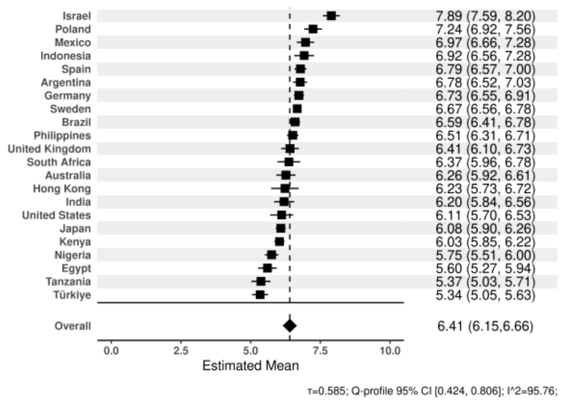

Life Satisfaction

Figure S21. Forest plot for `Employment status`-`Student`

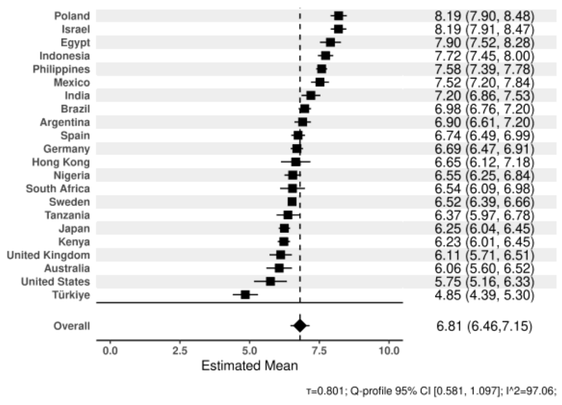

Happiness

Figure S21. Forest plot for `Employment status`-`Student`

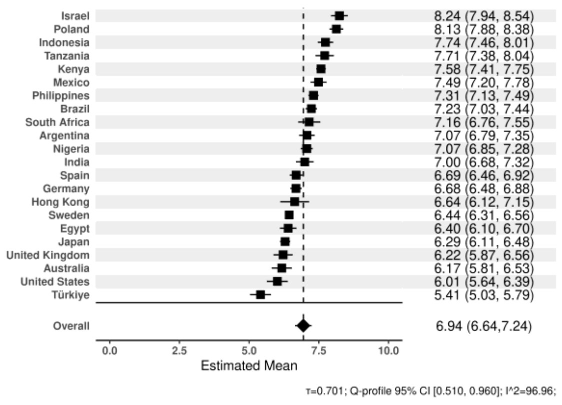

Cantril's Ladder	Life Satisfaction	Happiness

Cantril's Ladder

Figure S22. Forest plot for `Employment status`-`Homemaker`

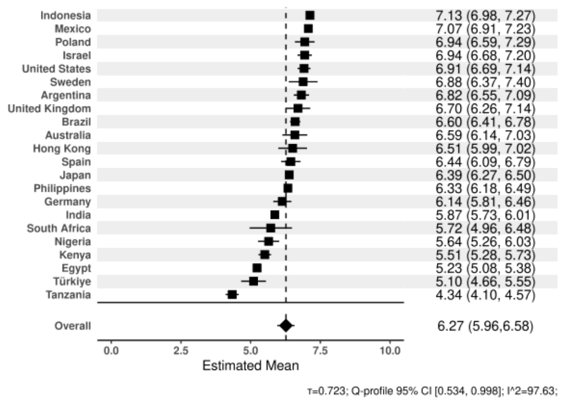

Life Satisfaction

Figure S22. Forest plot for `Employment status`-`Homemaker`

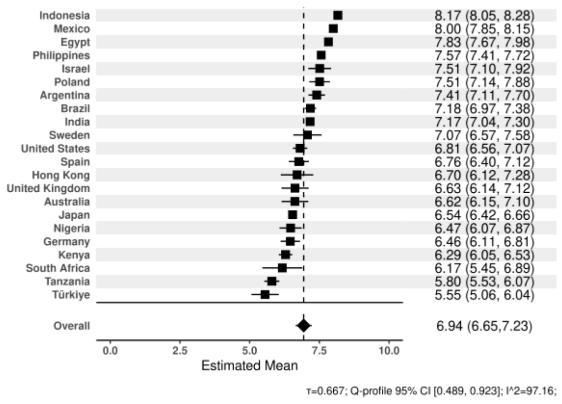

Happiness

Figure S22. Forest plot for `Employment status`-`Homemaker`

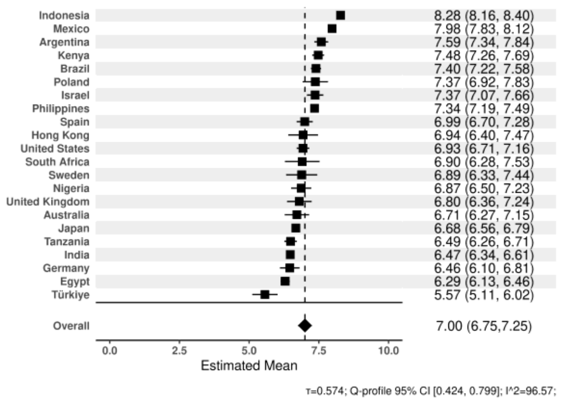

Cantril's Ladder

Figure S23. Forest plot for `Employment status`-`Unemployed and looking for a job`

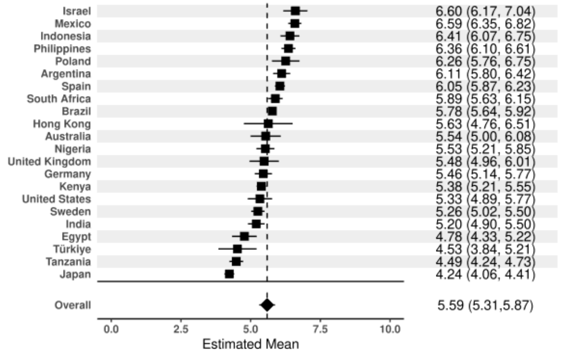

τ=0.650; Q-profile 95% CI [0.475, 0.904]; I^2=95.71;

Life Satisfaction

Figure S23. Forest plot for `Employment status`-`Unemployed and looking for a job`

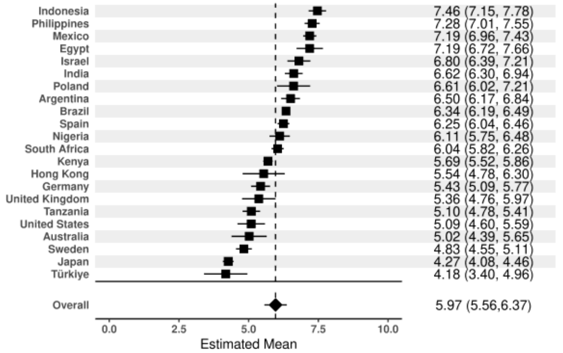

τ=0.943; Q-profile 95% CI [0.687, 1.293]; I^2=97.64;

Happiness

Figure S23. Forest plot for `Employment status`-`Unemployed and looking for a job`

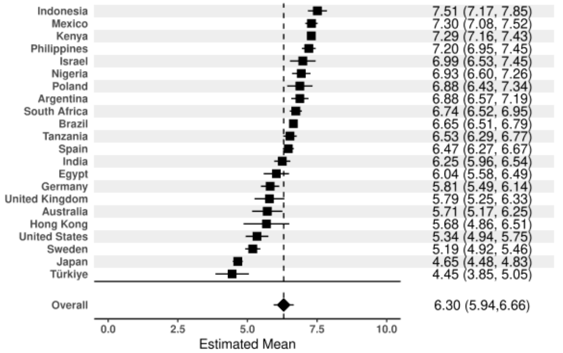

τ=0.843; Q-profile 95% CI [0.615, 1.158]; I^2=97.53;

Cantril's Ladder

Figure S24. Forest plot for `Employment status`-`None of these/other`

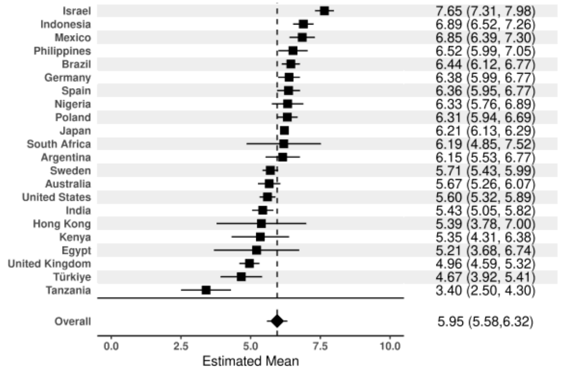

τ=0.818; Q-profile 95% CI [0.563, 1.148]; I^2=95.48;

Life Satisfaction

Figure S24. Forest plot for `Employment status`-`None of these/other`

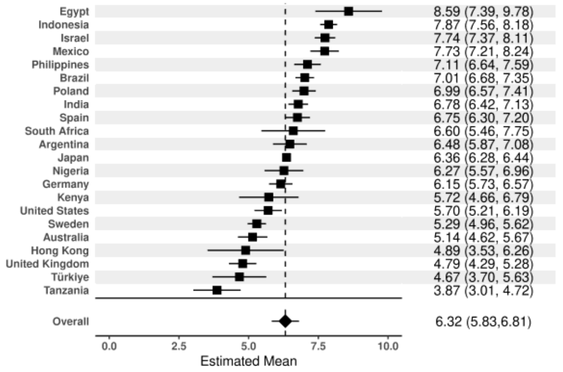

τ=1.121; Q-profile 95% CI [0.782, 1.541]; I^2=97.06;

Happiness

Figure S24. Forest plot for `Employment status`-`None of these/other`

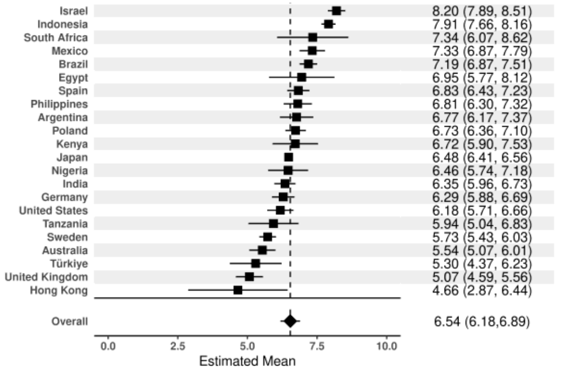

τ=0.781; Q-profile 95% CI [0.550, 1.097]; I^2=94.97;

Cantril's Ladder

Figure S25. Forest plot for `Religious service attendance`-`>1/week`

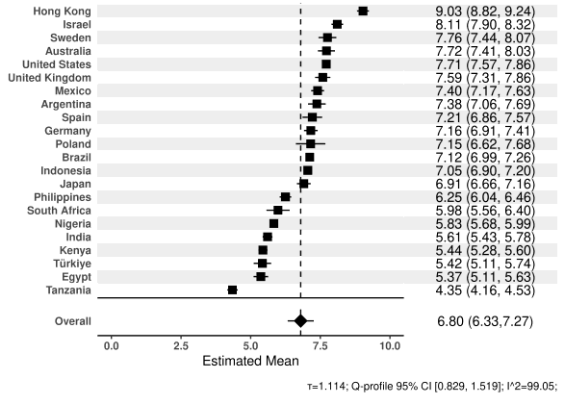

Life Satisfaction

Figure S25. Forest plot for `Religious service attendance`-`>1/week`

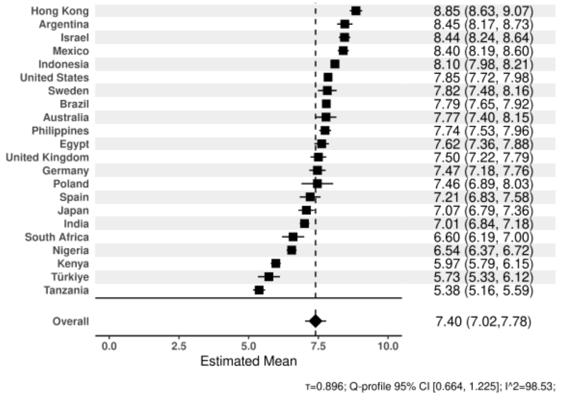

Happiness

Figure S25. Forest plot for `Religious service attendance`-`>1/week`

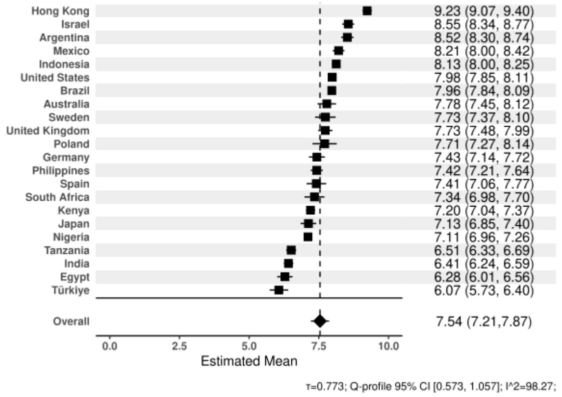

Cantril's Ladder

Figure S26. Forest plot for `Religious service attendance`-`1/week`

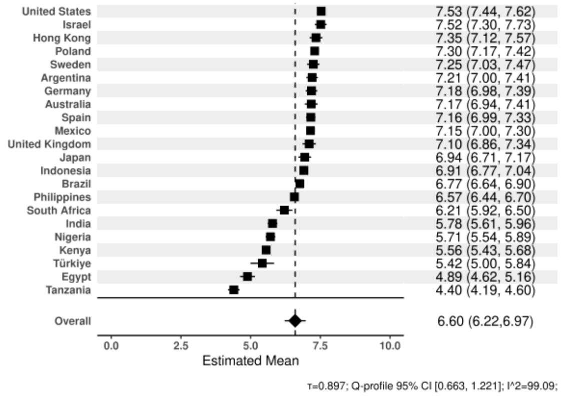

Life Satisfaction

Figure S26. Forest plot for `Religious service attendance`-`1/week`

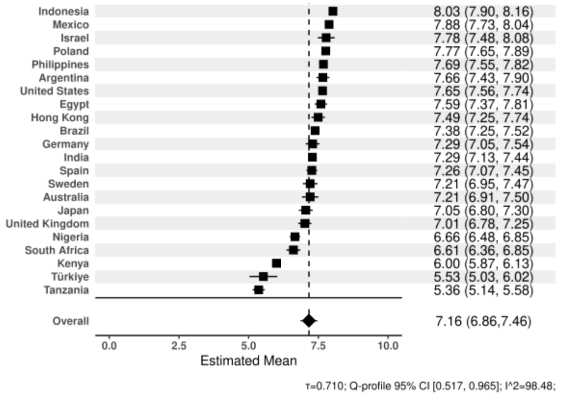

Happiness

Figure S26. Forest plot for `Religious service attendance`-`1/week`

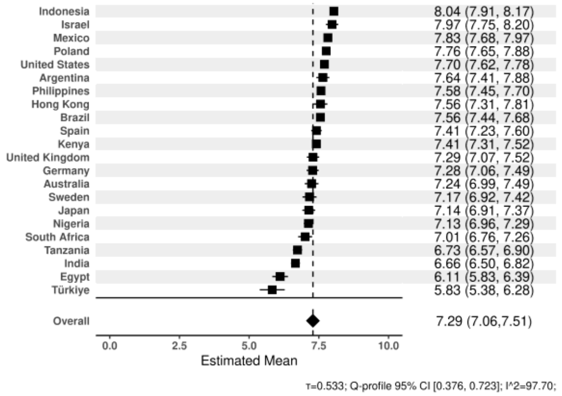

Cantril's Ladder

Figure S27. Forest plot for `Religious service attendance`-`1-3/month`

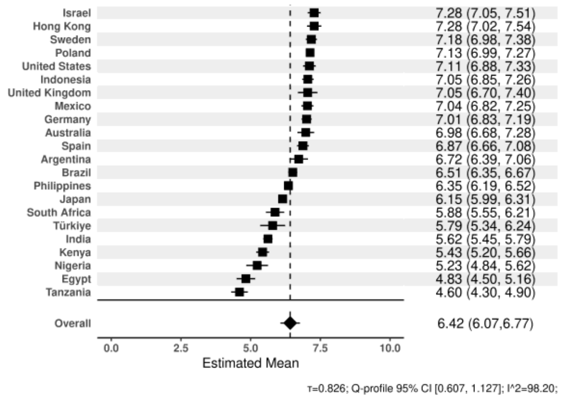

Life Satisfaction

Figure S27. Forest plot for `Religious service attendance`-`1-3/month`

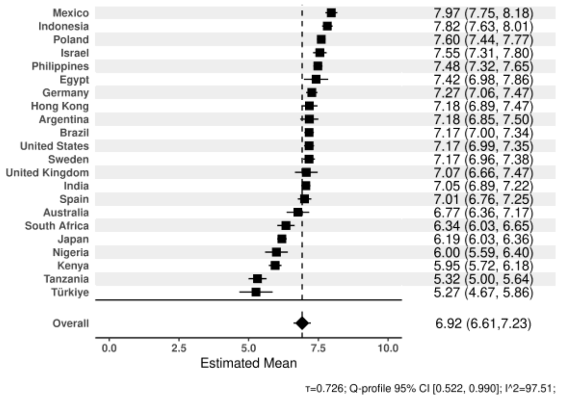

Happiness

Figure S27. Forest plot for `Religious service attendance`-`1-3/month`

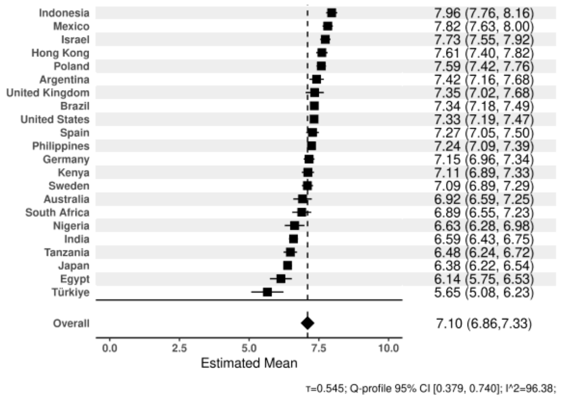

Cantril's Ladder

Figure S28. Forest plot for `Religious service attendance`-`A few times a year`

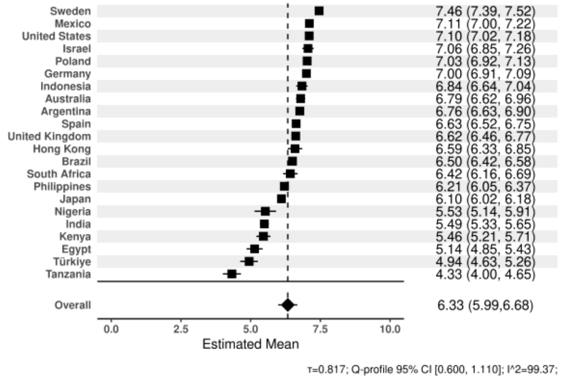

Life Satisfaction

Figure S28. Forest plot for `Religious service attendance`-`A few times a year`

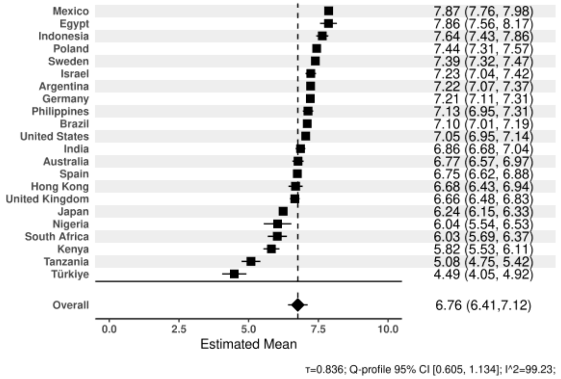

Happiness

Figure S28. Forest plot for `Religious service attendance`-`A few times a year`

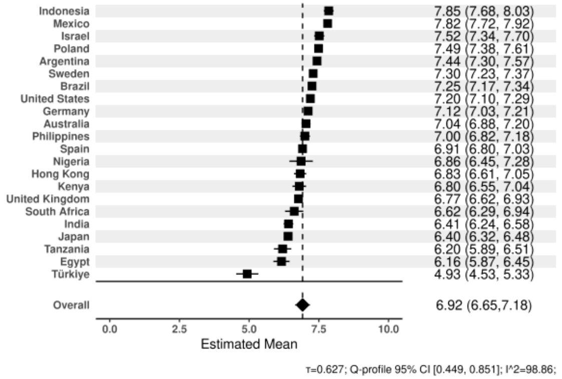

Cantril's Ladder

Figure S29. Forest plot for `Religious service attendance`-`Never`

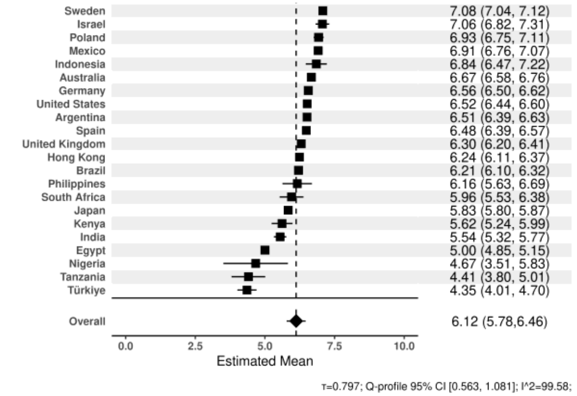

Life Satisfaction

Figure S29. Forest plot for `Religious service attendance`-`Never`

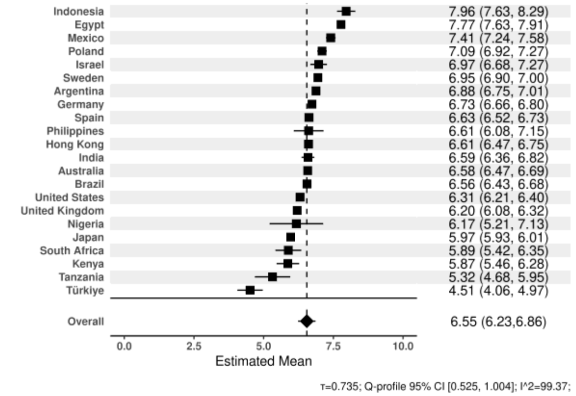

Happiness

Figure S29. Forest plot for `Religious service attendance`-`Never`

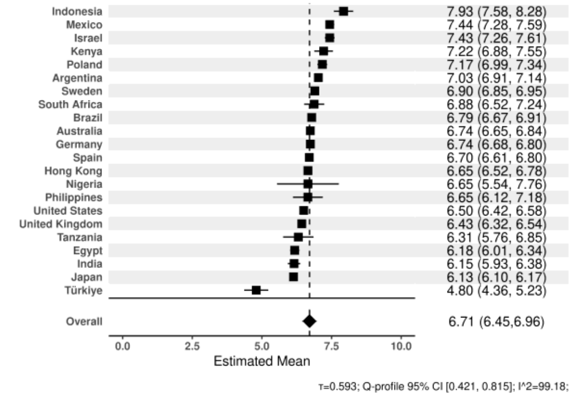

Cantril's Ladder

Figure S30. Forest plot for `Education`-`Up to 8 years`

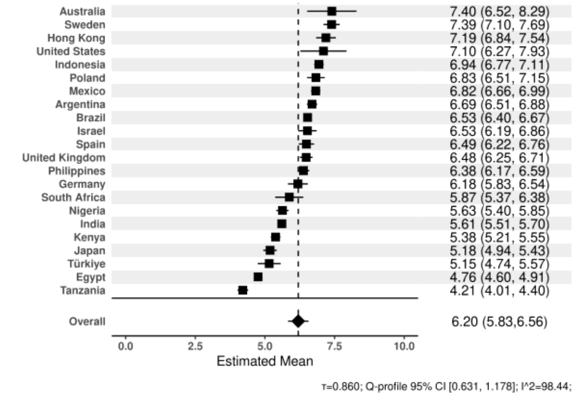

Life Satisfaction

Figure S30. Forest plot for `Education`-`Up to 8 years`

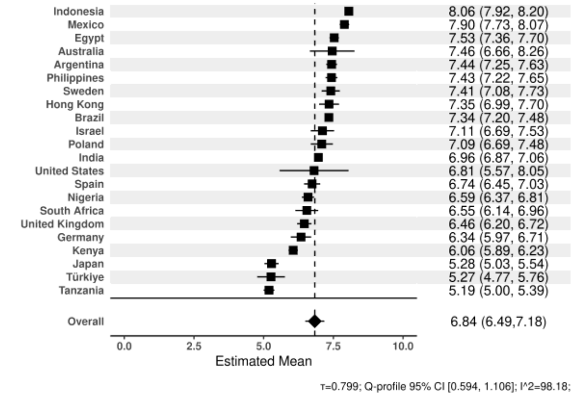

Happiness

Figure S30. Forest plot for `Education`-`Up to 8 years`

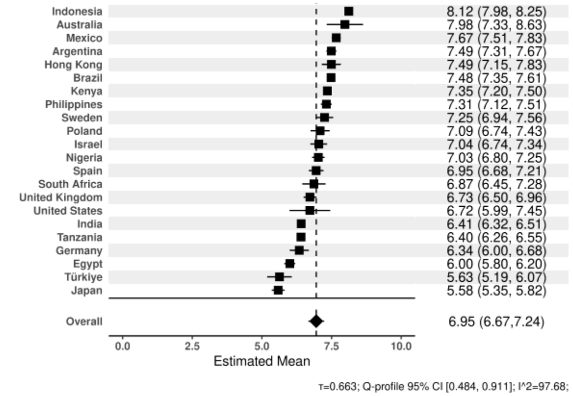

Cantril’s Ladder

Figure S31. Forest plot for `Education`-`9-15 years`

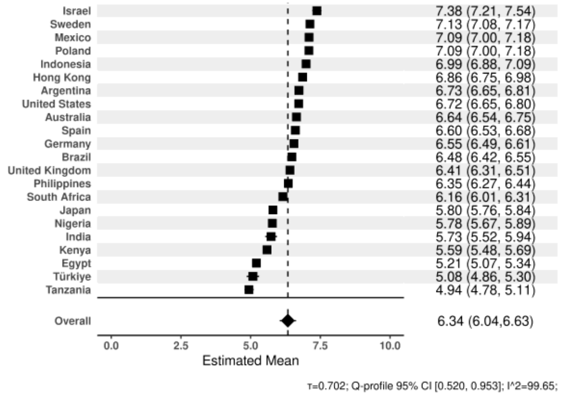

Life Satisfaction

Figure S31. Forest plot for `Education`-`9-15 years`

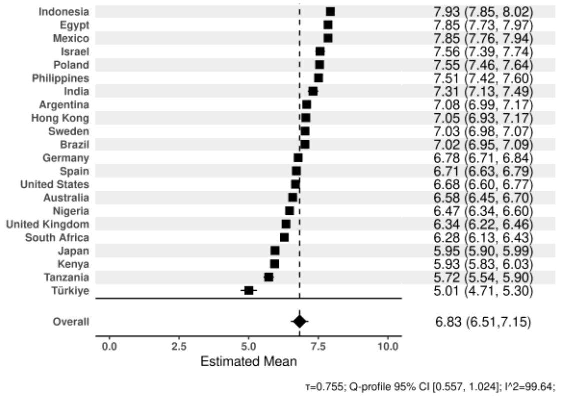

Happiness

Figure S31. Forest plot for `Education`-`9-15 years`

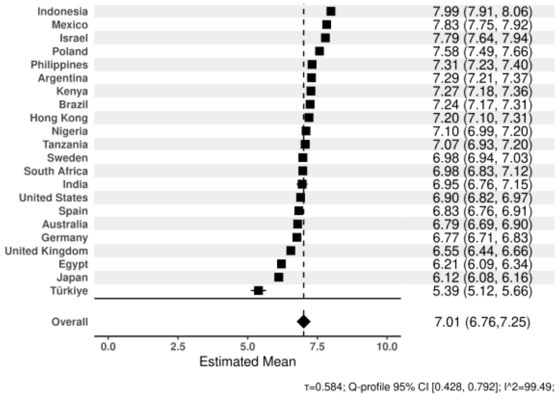

Cantril’s Ladder

Figure S32. Forest plot for `Education`-`16+ years`

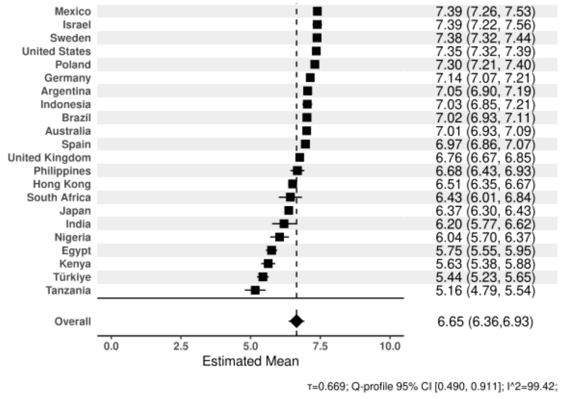

Life Satisfaction

Figure S32. Forest plot for `Education`-`16+ years`

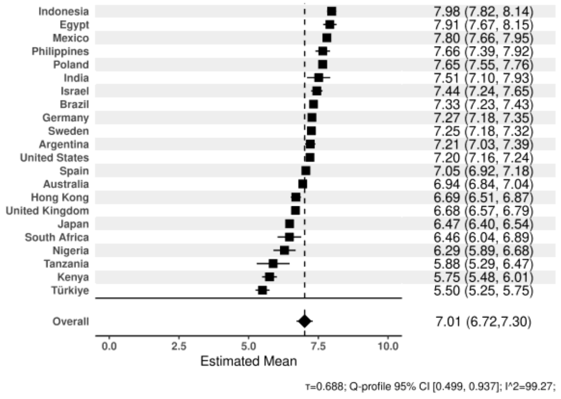

Happiness

Figure S32. Forest plot for `Education`-`16+ years`

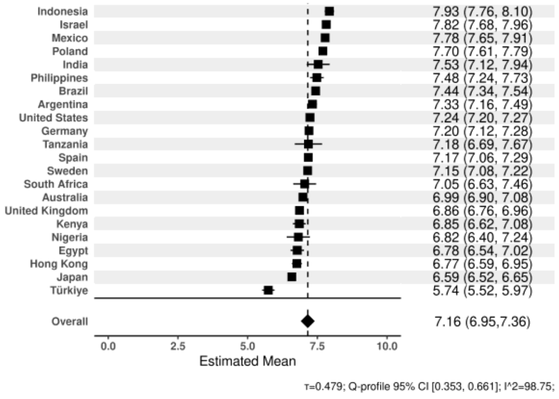

Cantril’s Ladder	Life Satisfaction	Happiness

Figure S33. Forest plot for `Immigration status`-`Born in this country`

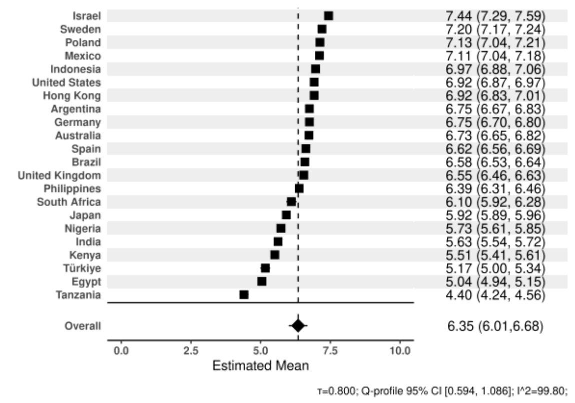

Figure S33. Forest plot for `Immigration status`-`Born in this country`

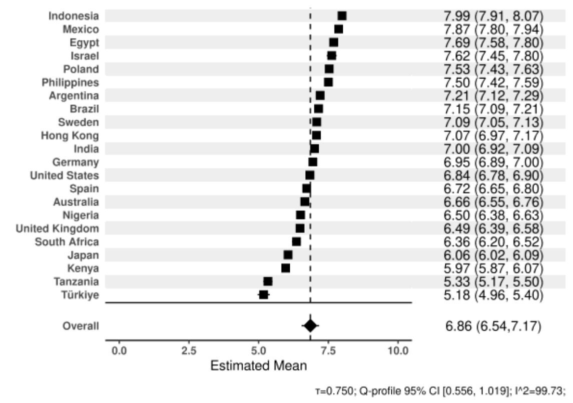

Figure S33. Forest plot for `Immigration status`-`Born in this country`

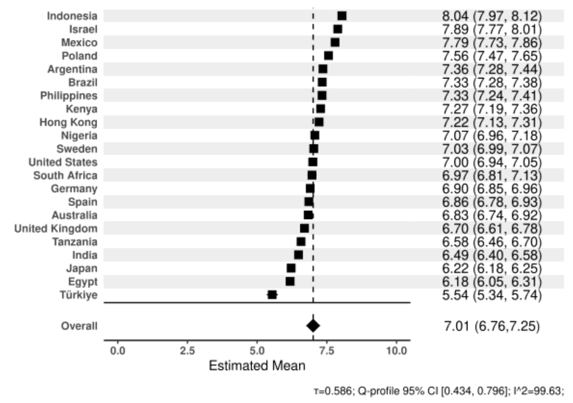

Cantril’s Ladder	Life Satisfaction	Happiness

Figure S34. Forest plot for `Immigration status`-`Born in another country`

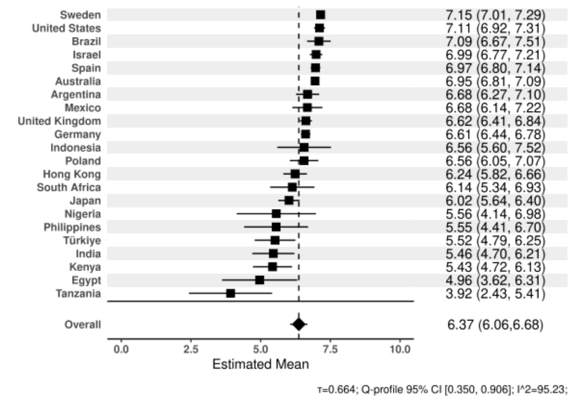

Figure S34. Forest plot for `Immigration status`-`Born in another country`

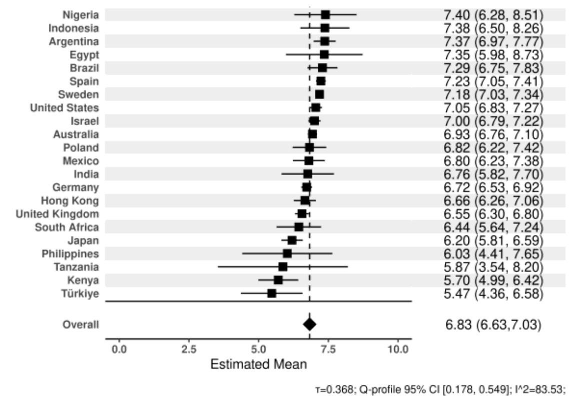

Figure S34. Forest plot for `Immigration status`-`Born in another country`

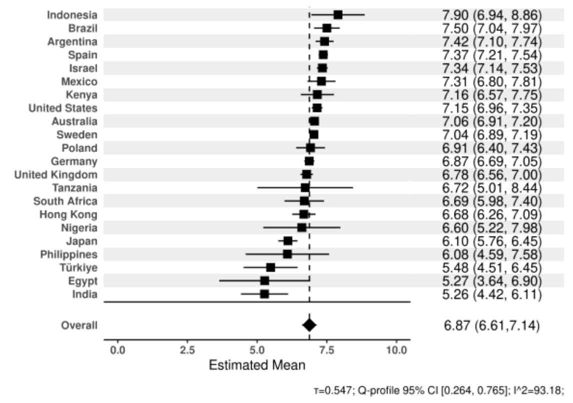

Figures S30(1-81). Forest plots for childhood predictor analyses

Cantril's Ladder

Figure S1. Forest plot for `Relationship with mother`-`Very good/somewhat good` effect

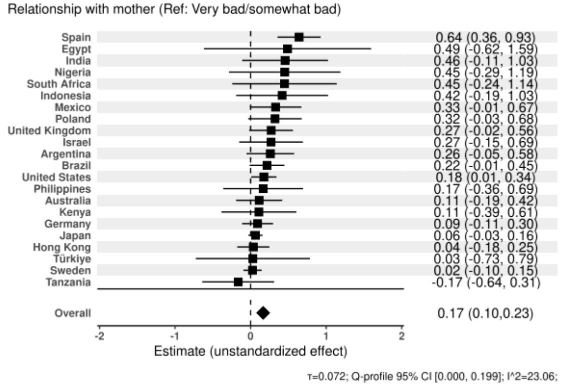

Life Satisfaction

Figure S1. Forest plot for `Relationship with mother`-`Very good/somewhat good` effect

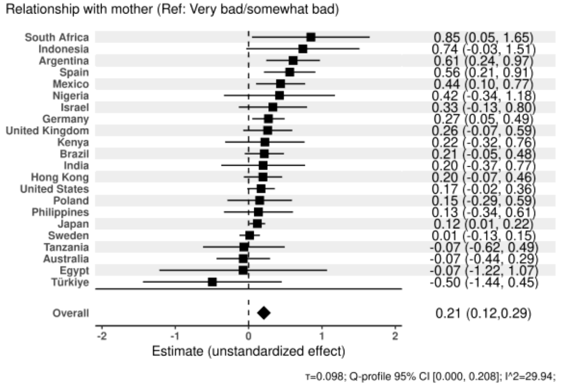

Happiness

Figure S1. Forest plot for `Relationship with mother`-`Very good/somewhat good` effect

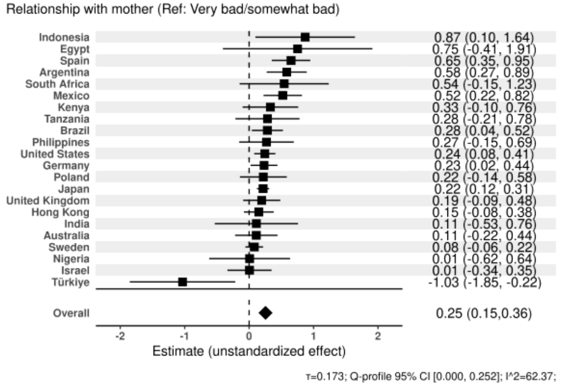

Cantril's Ladder

Figure S2. Forest plot for `Relationship with father`-`Very good/somewhat good` effect

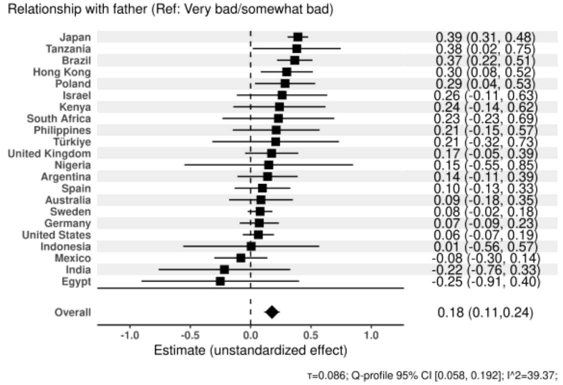

Life Satisfaction

Figure S2. Forest plot for `Relationship with father`-`Very good/somewhat good` effect

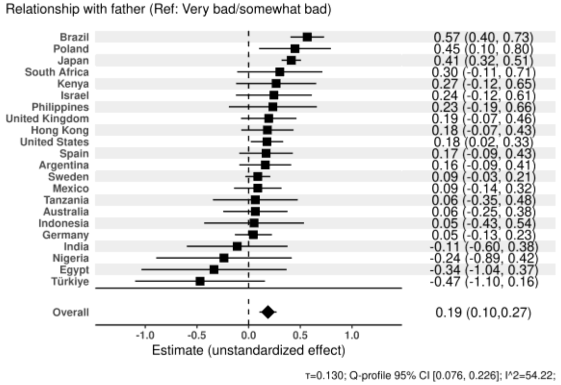

Happiness

Figure S2. Forest plot for `Relationship with father`-`Very good/somewhat good` effect

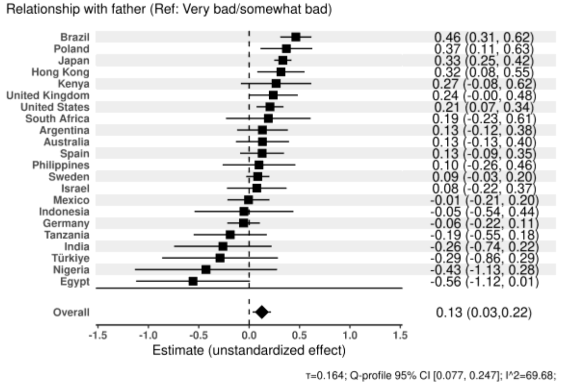

Cantril's Ladder	Life Satisfaction	Happiness

Cantril's Ladder

Figure S3. Forest plot for `Parent marital status`-`Divorced` effect

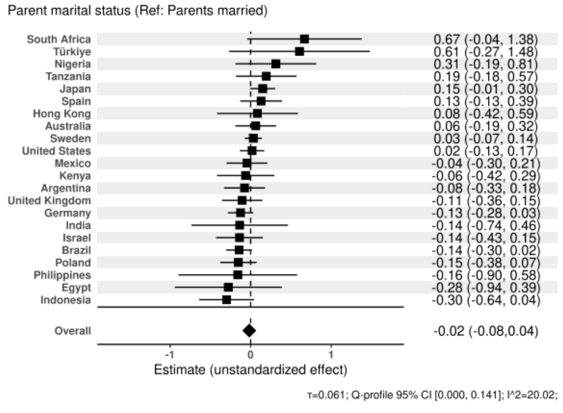

Life Satisfaction

Figure S3. Forest plot for `Parent marital status`-`Divorced` effect

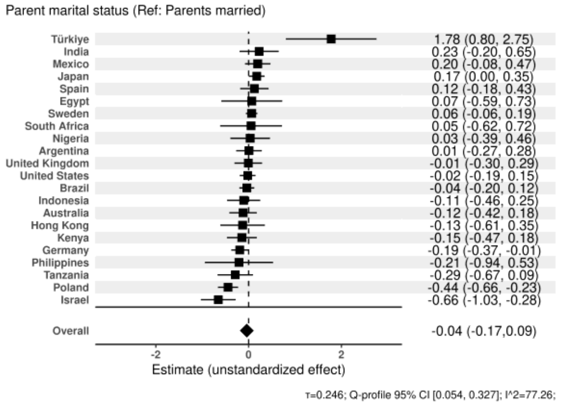

Happiness

Figure S3. Forest plot for `Parent marital status`-`Divorced` effect

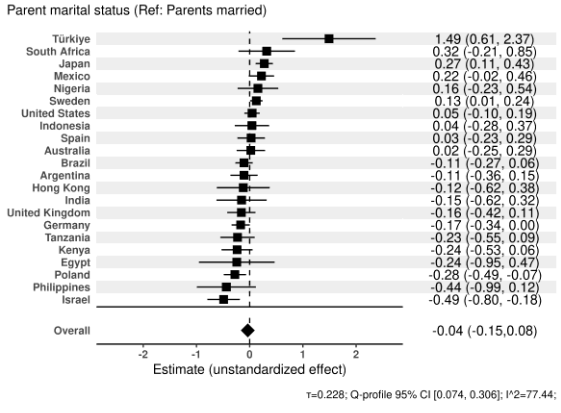

Cantril's Ladder

Figure S4. Forest plot for `Parent marital status`-`Single, never married` effect

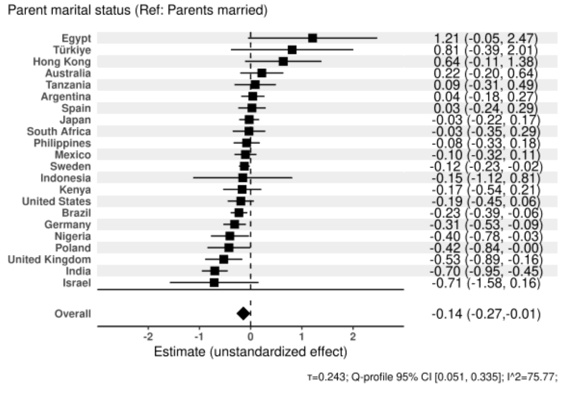

Life Satisfaction

Figure S4. Forest plot for `Parent marital status`-`Single, never married` effect

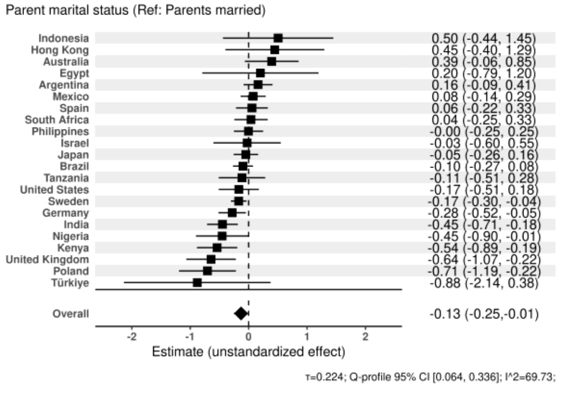

Happiness

Figure S4. Forest plot for `Parent marital status`-`Single, never married` effect

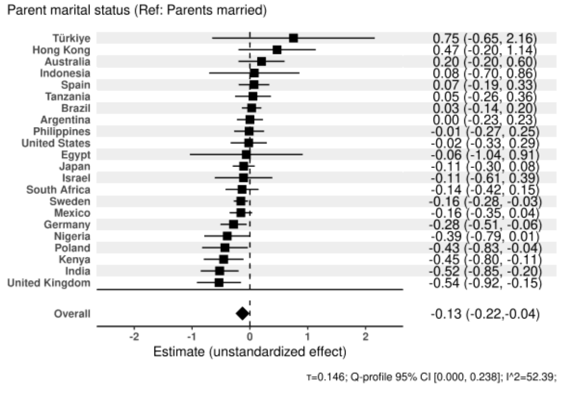

Cantril’s Ladder

Figure S5. Forest plot for `Parent marital status`-`One or both parents had died` effect

Parent marital status (Ref: Parents married)

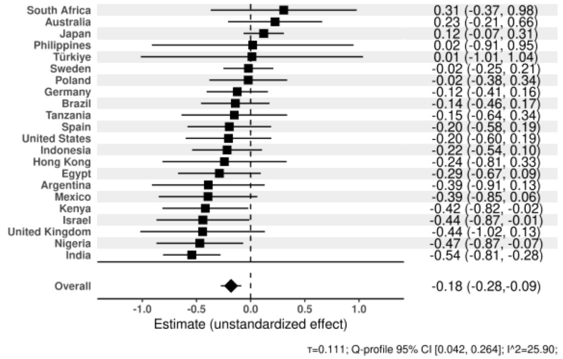

Life Satisfaction

Figure S5. Forest plot for `Parent marital status`-`One or both parents had died` effect

Parent marital status (Ref: Parents married)

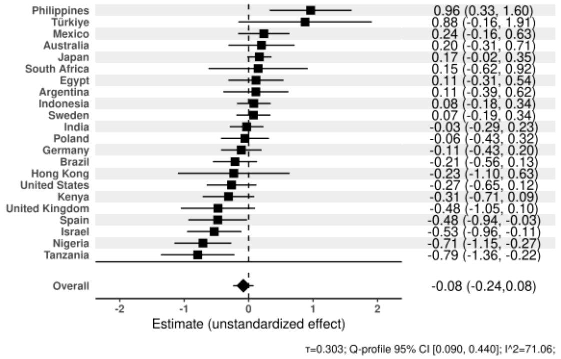

Happiness

Figure S5. Forest plot for `Parent marital status`-`One or both parents had died` effect

Parent marital status (Ref: Parents married)

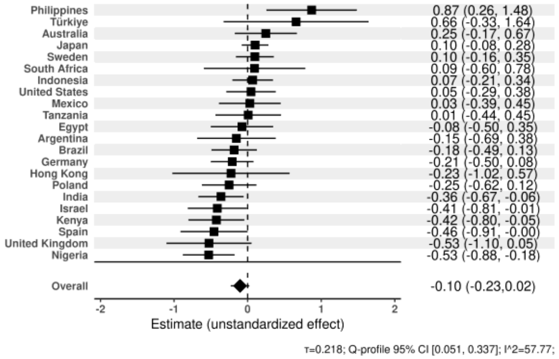

Cantril’s Ladder

Figure S6. Forest plot for `Subjective financial status of family growing up`-`Lived comfortably` effect

Subjective financial status of family growing up (Ref: Got by)

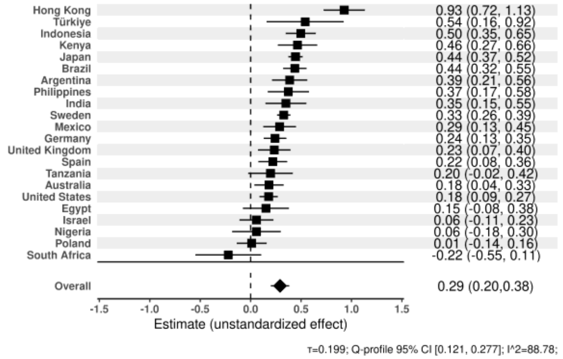

Life Satisfaction

Figure S6. Forest plot for `Subjective financial status of family growing up`-`Lived comfortably` effect

Subjective financial status of family growing up (Ref: Got by)

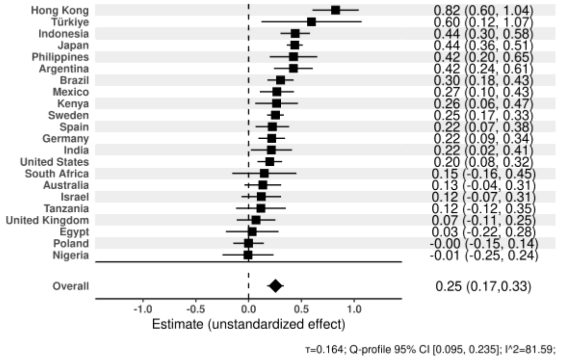

Happiness

Figure S6. Forest plot for `Subjective financial status of family growing up`-`Lived comfortably` effect

Subjective financial status of family growing up (Ref: Got by)

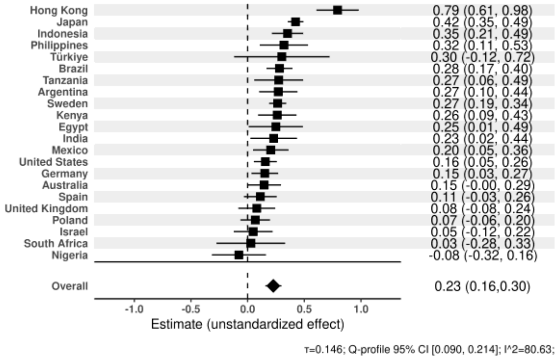

Cantril’s Ladder	Life Satisfaction	Happiness

Figure S7. Forest plot for `Subjective financial status of family growing up`-`Found it difficult` effect

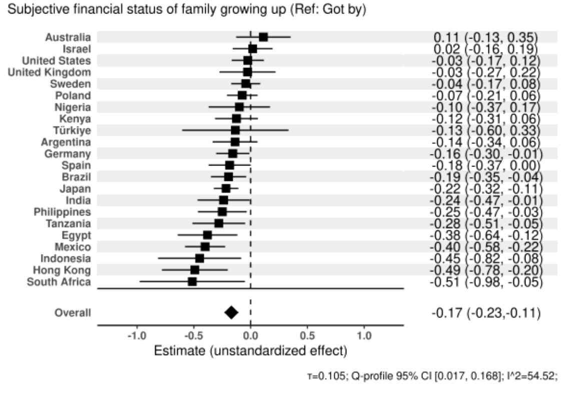

Figure S7. Forest plot for `Subjective financial status of family growing up`-`Found it difficult` effect

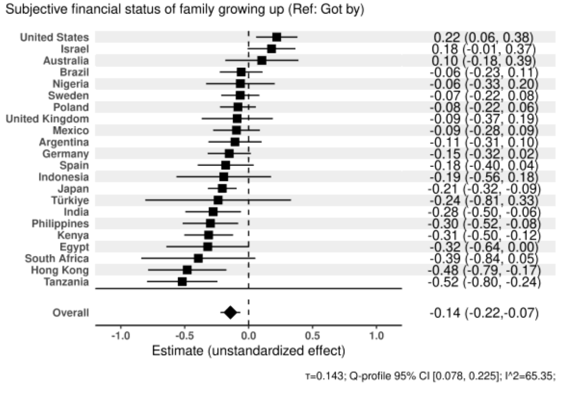

Figure S7. Forest plot for `Subjective financial status of family growing up`-`Found it difficult` effect

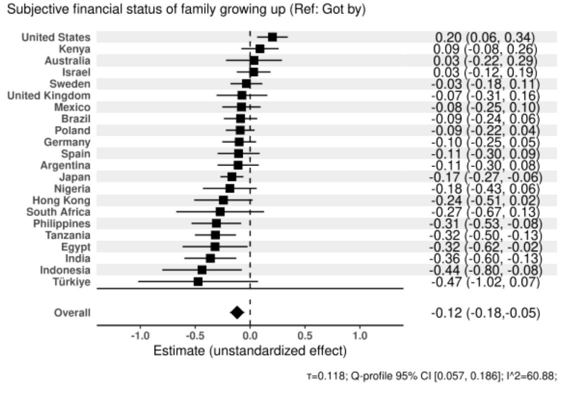

Cantril’s Ladder	Life Satisfaction	Happiness

Figure S8. Forest plot for `Subjective financial status of family growing up`-`Found it very difficult` effect

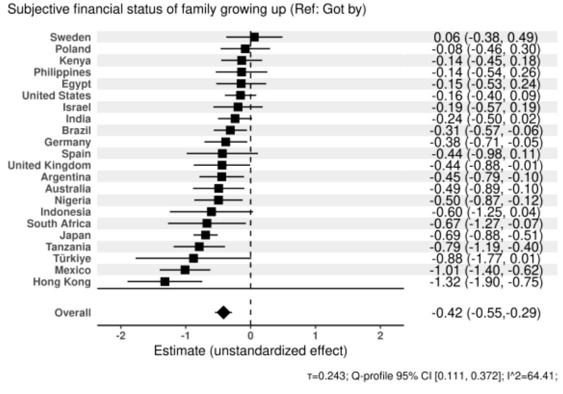

Figure S8. Forest plot for `Subjective financial status of family growing up`-`Found it very difficult` effect

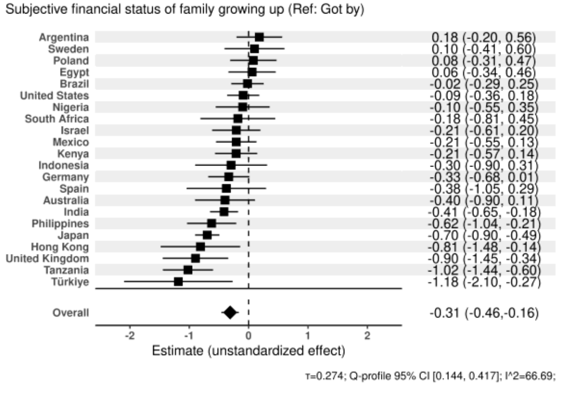

Figure S8. Forest plot for `Subjective financial status of family growing up`-`Found it very difficult` effect

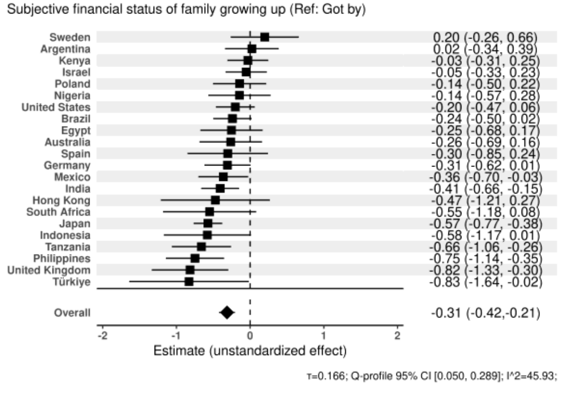

Cantril's Ladder

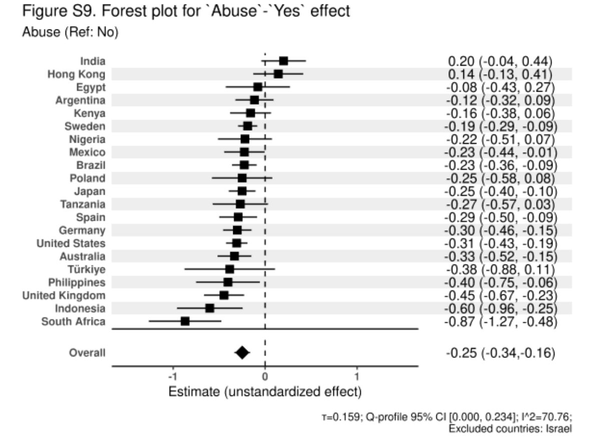

Life Satisfaction

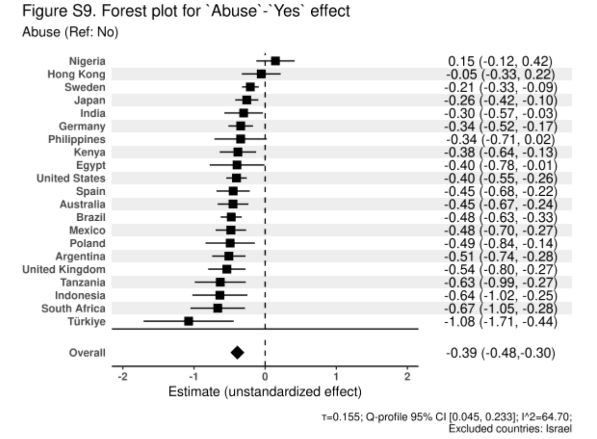

Happiness

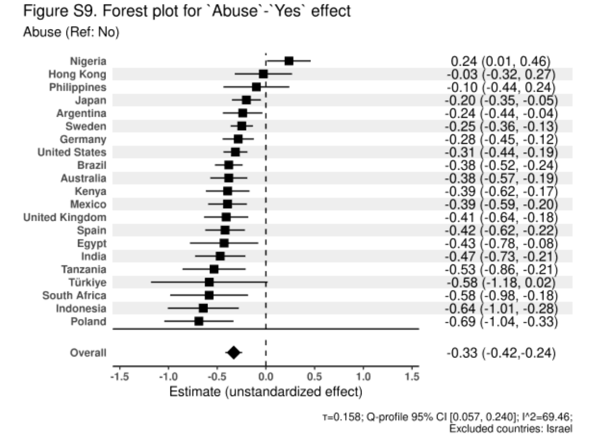

Cantril's Ladder

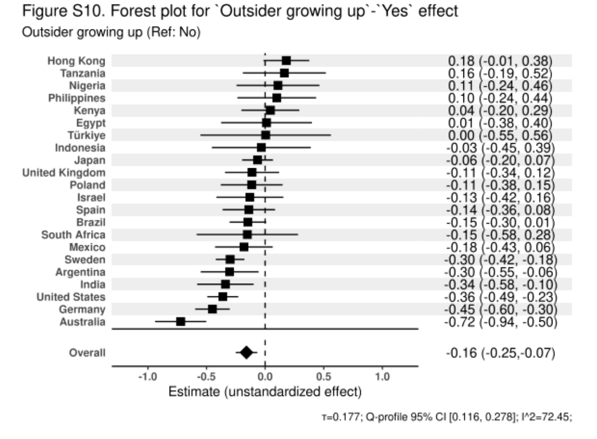

Life Satisfaction

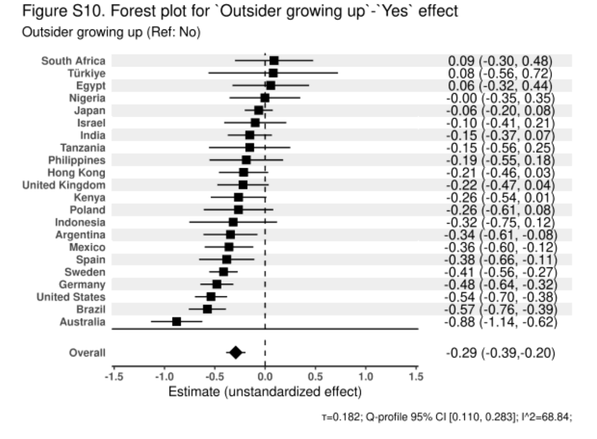

Happiness

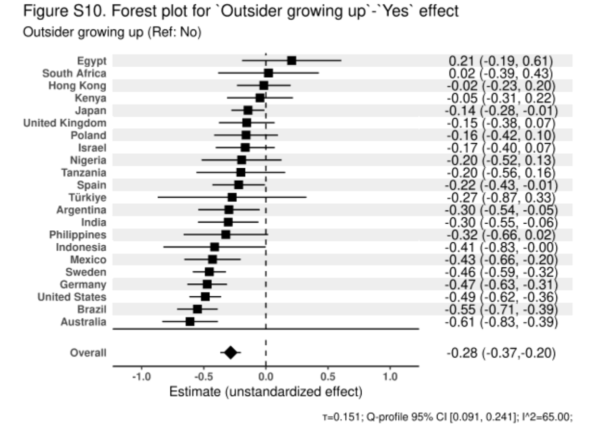

\\

Cantril's Ladder

Figure S11. Forest plot for `Self-rated health growing up`-`Excellent` effect

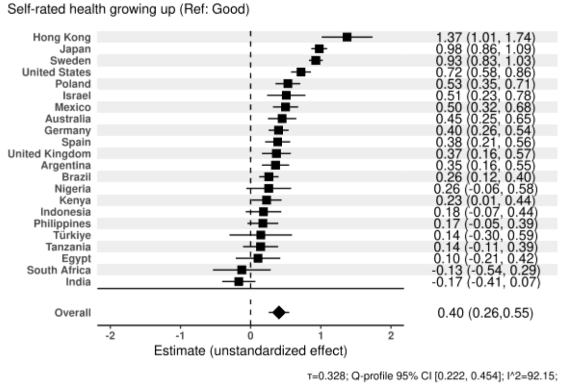

Life Satisfaction

Figure S11. Forest plot for `Self-rated health growing up`-`Excellent` effect

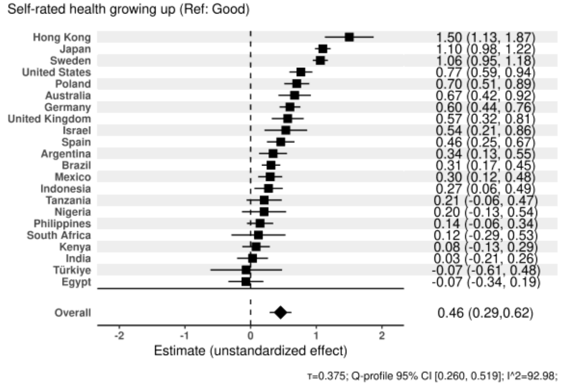

Happiness

Figure S11. Forest plot for `Self-rated health growing up`-`Excellent` effect

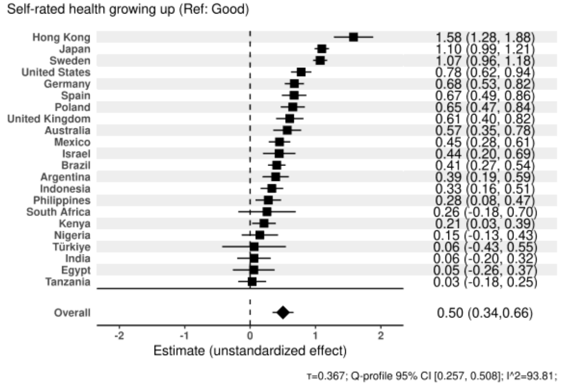

Cantril's Ladder

Figure S12. Forest plot for `Self-rated health growing up`-`Very good` effect

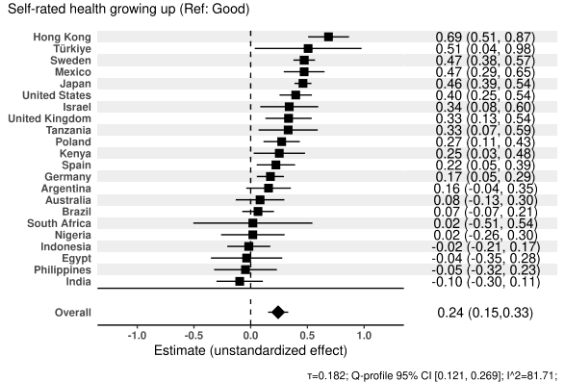

Life Satisfaction

Figure S12. Forest plot for `Self-rated health growing up`-`Very good` effect

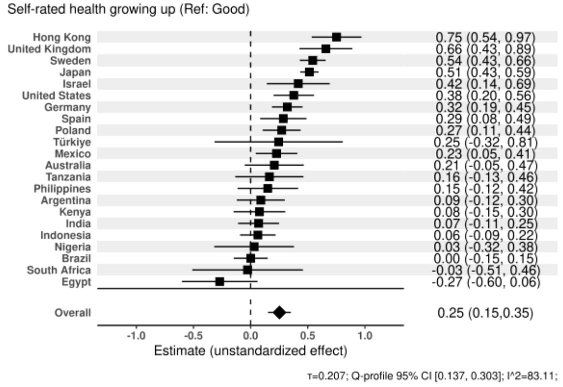

Happiness

Figure S12. Forest plot for `Self-rated health growing up`-`Very good` effect

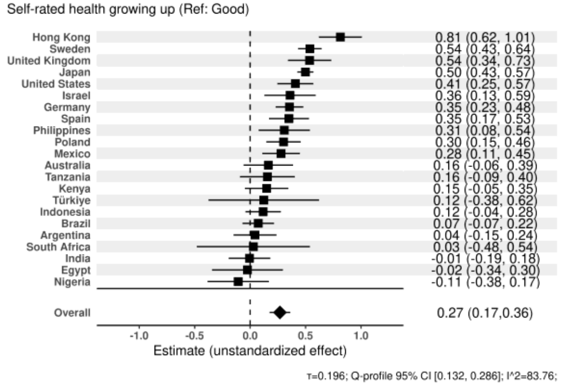

Cantril's Ladder

Figure S13. Forest plot for `Self-rated health growing up`-`Fair` effect

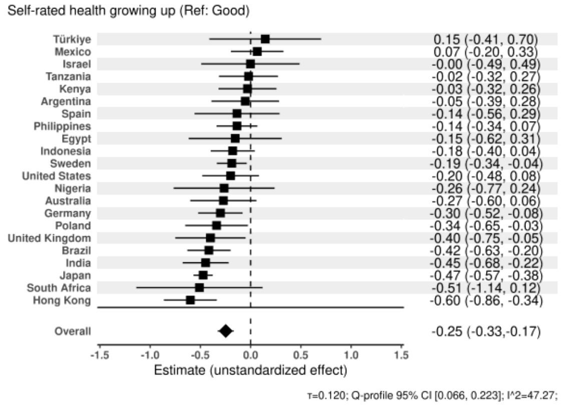

Life Satisfaction

Figure S13. Forest plot for `Self-rated health growing up`-`Fair` effect

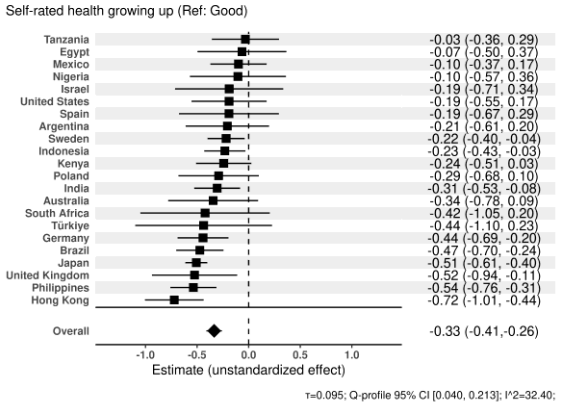

Happiness

Figure S13. Forest plot for `Self-rated health growing up`-`Fair` effect

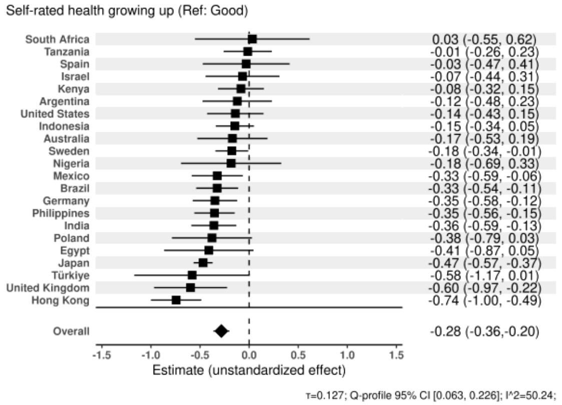

Cantril's Ladder

Figure S14. Forest plot for `Self-rated health growing up`-`Poor` effect

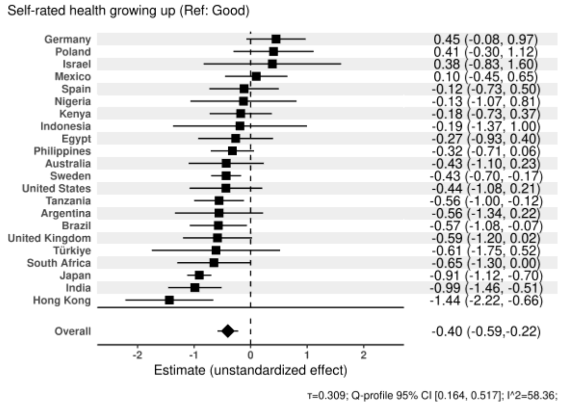

Life Satisfaction

Figure S14. Forest plot for `Self-rated health growing up`-`Poor` effect

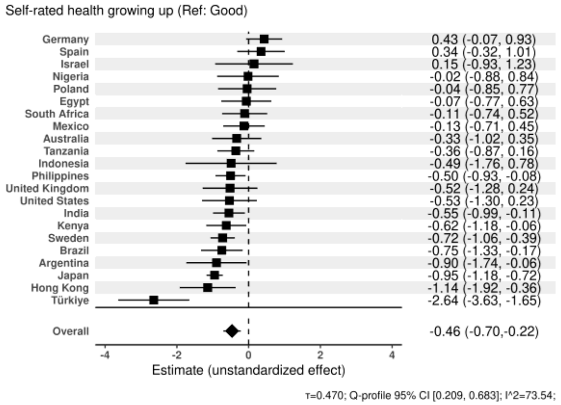

Happiness

Figure S14. Forest plot for `Self-rated health growing up`-`Poor` effect

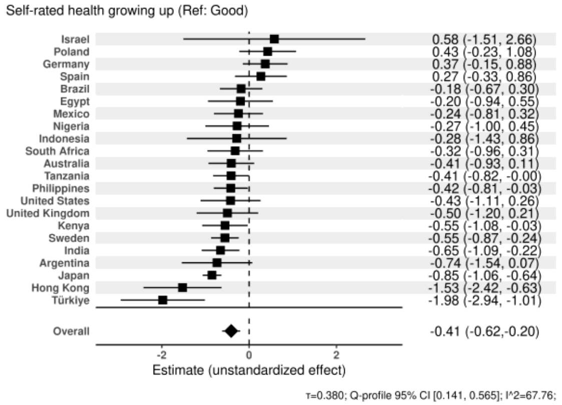

Cantril's Ladder

Figure S15. Forest plot for `Immigration status`-`Born in another country` effect

Immigration status (Ref: Born in this country)

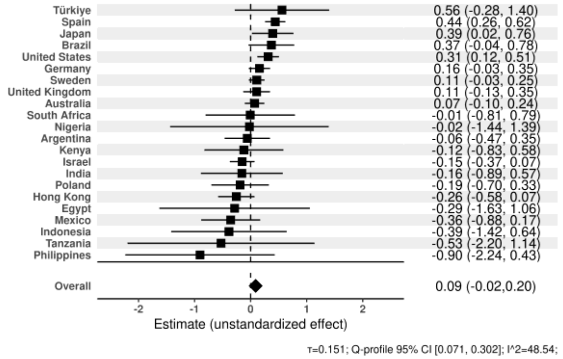

Life Satisfaction

Figure S15. Forest plot for `Immigration status`-`Born in another country` effect

Immigration status (Ref: Born in this country)

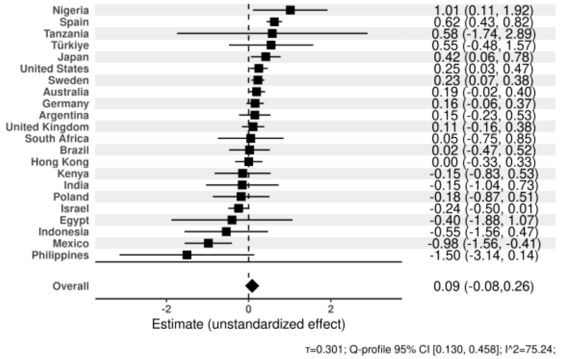

Happiness

Figure S15. Forest plot for `Immigration status`-`Born in another country` effect

Immigration status (Ref: Born in this country)

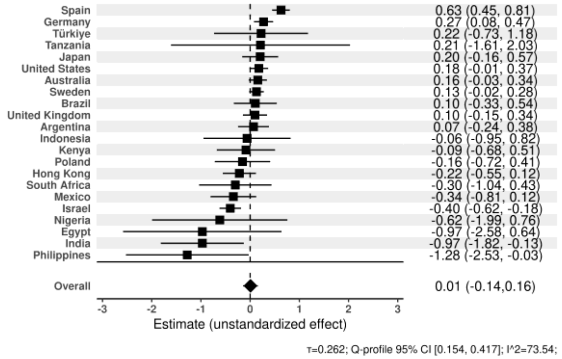

Cantril's Ladder

Figure S16. Forest plot for `Age 12 religious service attendance`-`At least 1/week` effect

Age 12 religious service attendance (Ref: Never)

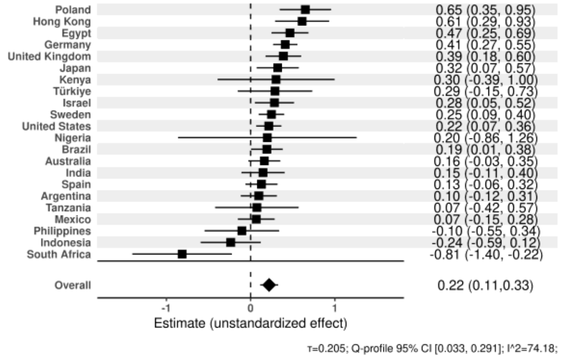

Life Satisfaction

Figure S16. Forest plot for `Age 12 religious service attendance`-`At least 1/week` effect

Age 12 religious service attendance (Ref: Never)

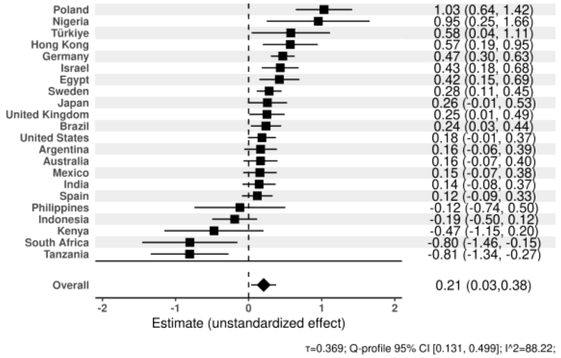

Happiness

Figure S16. Forest plot for `Age 12 religious service attendance`-`At least 1/week` effect

Age 12 religious service attendance (Ref: Never)

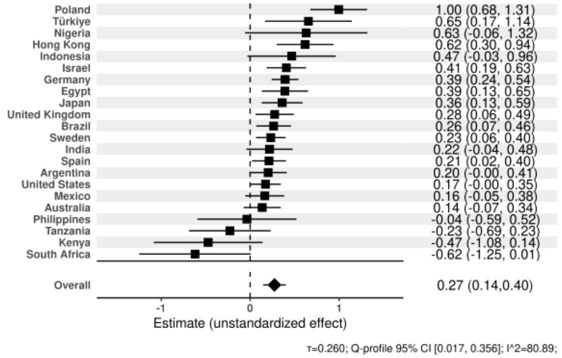

Cantril’s Ladder	Life Satisfaction	Happiness

Cantril’s Ladder

Figure S17. Forest plot for `Age 12 religious service attendance`-`1-3/month` effect

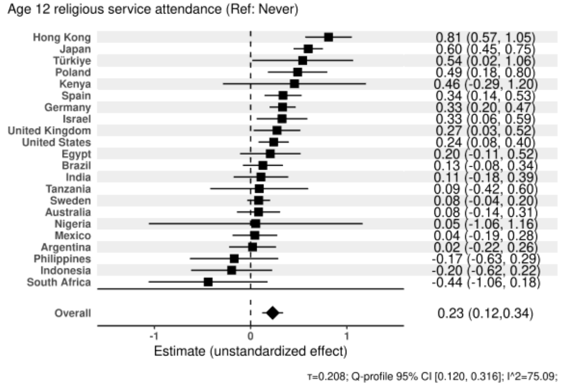

Life Satisfaction

Figure S17. Forest plot for `Age 12 religious service attendance`-`1-3/month` effect

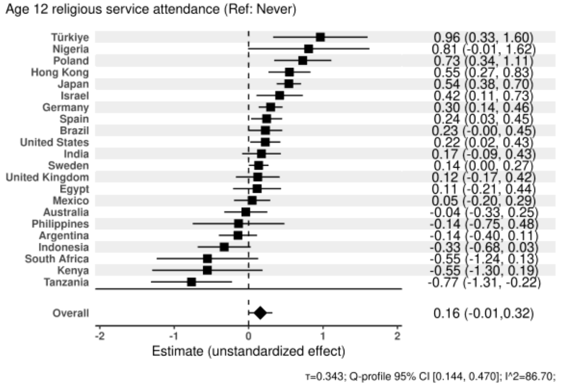

Happiness

Figure S17. Forest plot for `Age 12 religious service attendance`-`1-3/month` effect

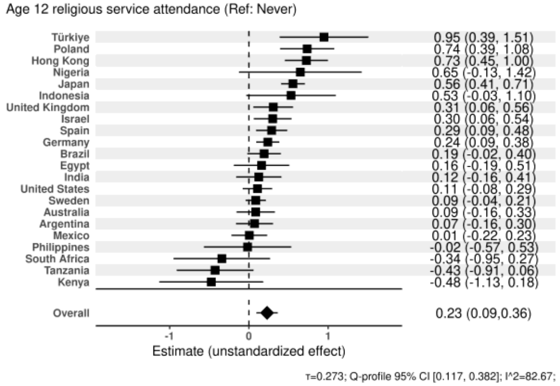

Cantril’s Ladder	Life Satisfaction	Happiness

Cantril’s Ladder

Figure S18. Forest plot for `Age 12 religious service attendance`-`< 1/month` effect

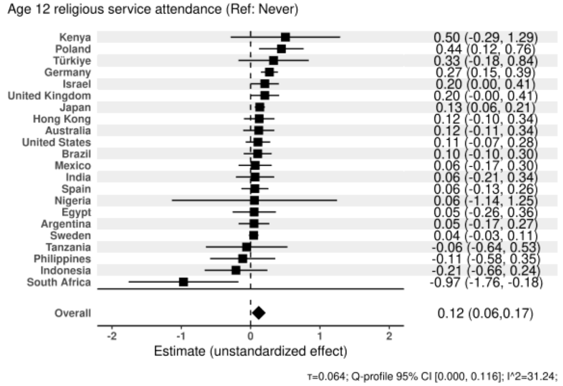

Life Satisfaction

Figure S18. Forest plot for `Age 12 religious service attendance`-`< 1/month` effect

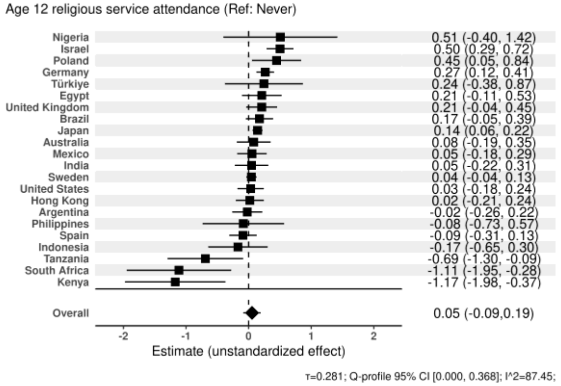

Happiness

Figure S18. Forest plot for `Age 12 religious service attendance`-`< 1/month` effect

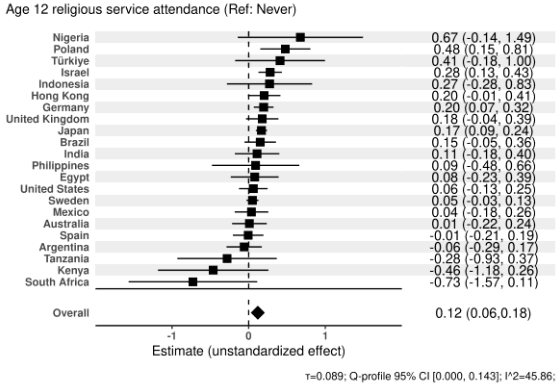

Cantril's Ladder	Life Satisfaction	Happiness

Cantril's Ladder

Figure S19. Forest plot for `Year of birth`-`1993-1998; age 25-29` effect

Year of birth (Ref: 1998-2005; age 18-24)

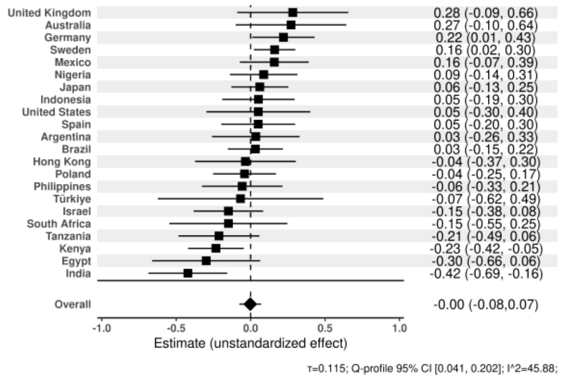

Life Satisfaction

Figure S19. Forest plot for `Year of birth`-`1993-1998; age 25-29` effect

Year of birth (Ref: 1998-2005; age 18-24)

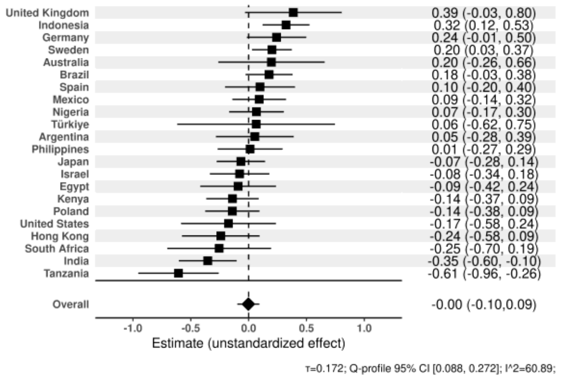

Happiness

Figure S19. Forest plot for `Year of birth`-`1993-1998; age 25-29` effect

Year of birth (Ref: 1998-2005; age 18-24)

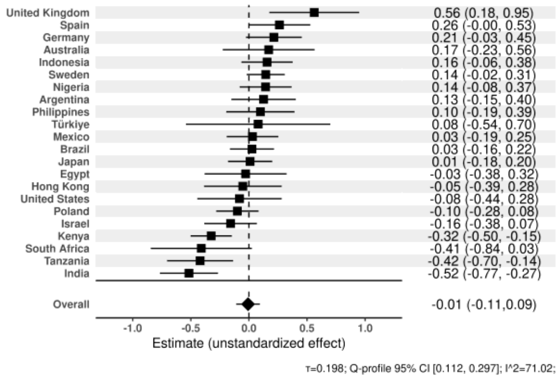

Cantril's Ladder	Life Satisfaction	Happiness

Cantril's Ladder

Figure S20. Forest plot for `Year of birth`-`1983-1993; age 30-39` effect

Year of birth (Ref: 1998-2005; age 18-24)

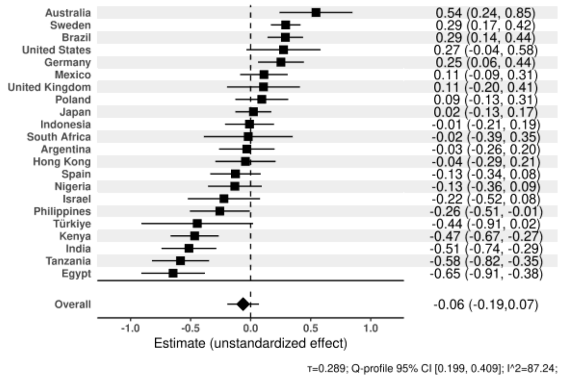

Life Satisfaction

Figure S20. Forest plot for `Year of birth`-`1983-1993; age 30-39` effect

Year of birth (Ref: 1998-2005; age 18-24)

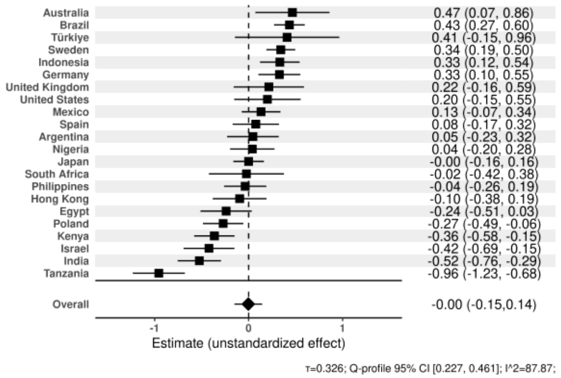

Happiness

Figure S20. Forest plot for `Year of birth`-`1983-1993; age 30-39` effect

Year of birth (Ref: 1998-2005; age 18-24)

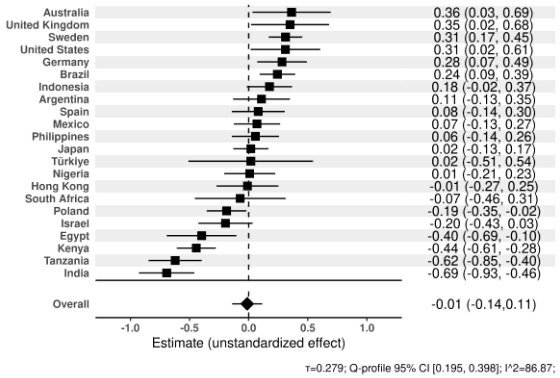

Cantril's Ladder

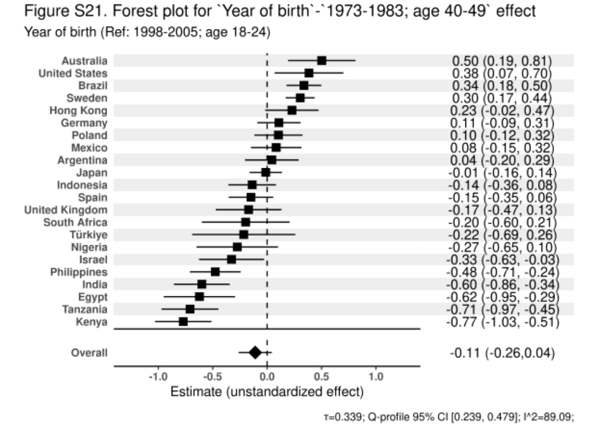

Life Satisfaction

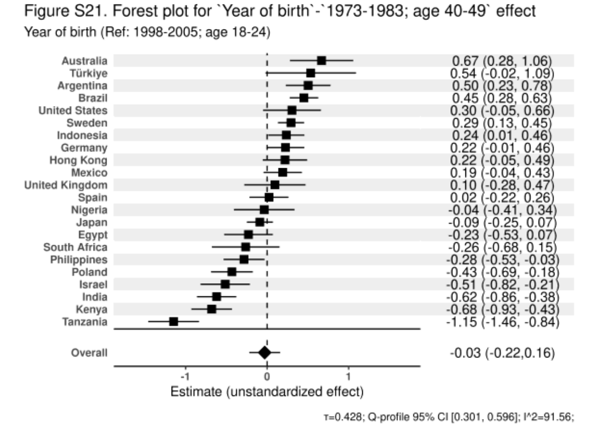

Happiness

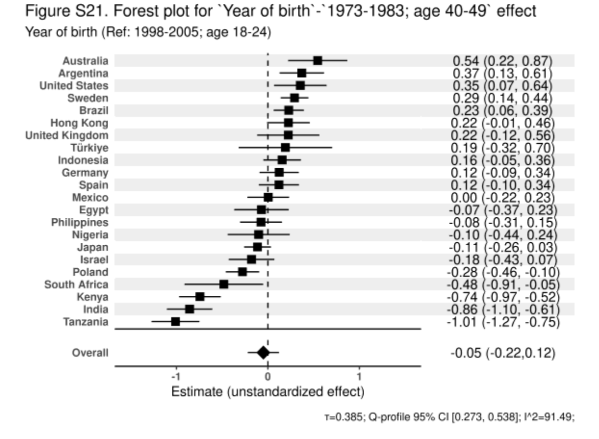

Cantril's Ladder

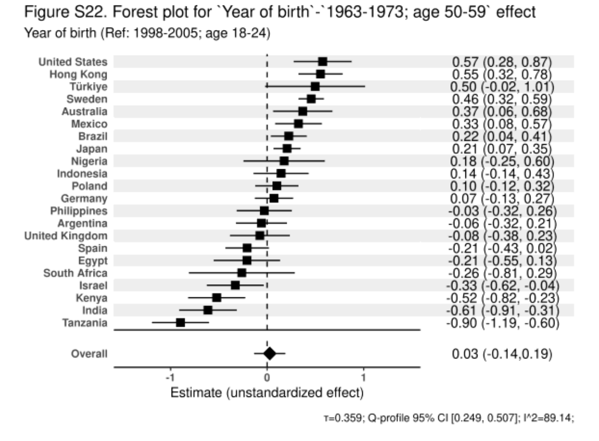

Life Satisfaction

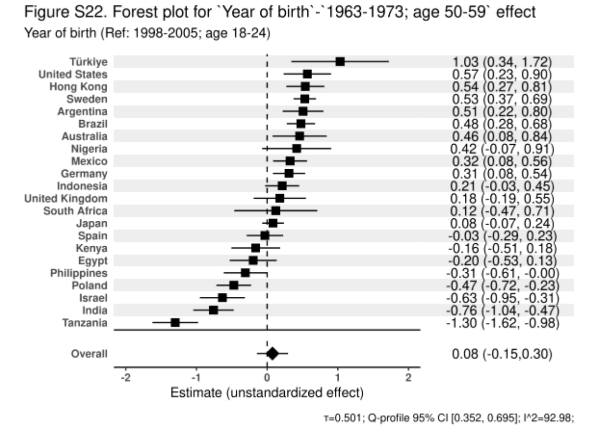

Happiness

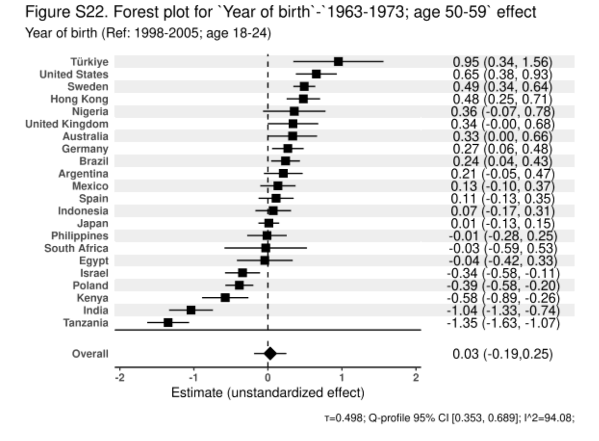

Cantril's Ladder

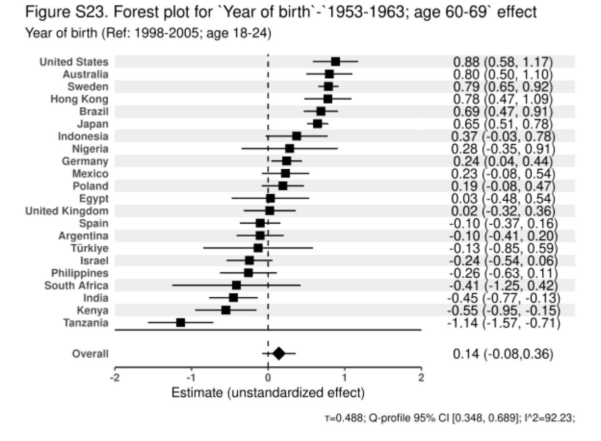

Life Satisfaction

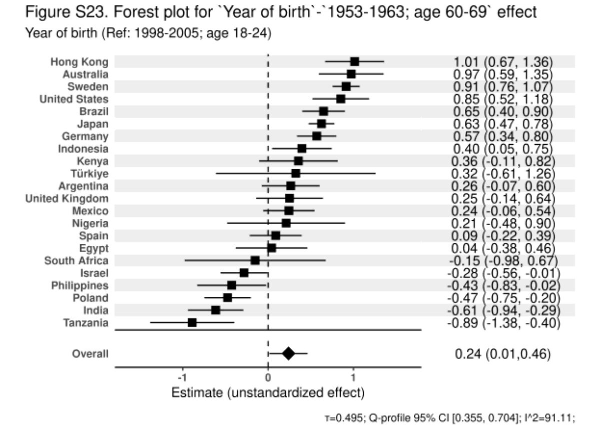

Happiness

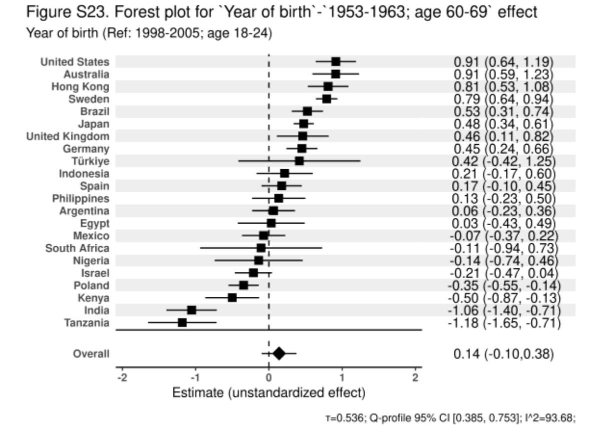

Cantril's Ladder

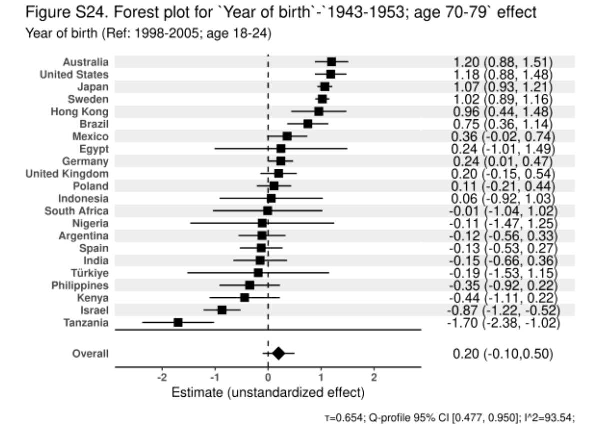

Life Satisfaction

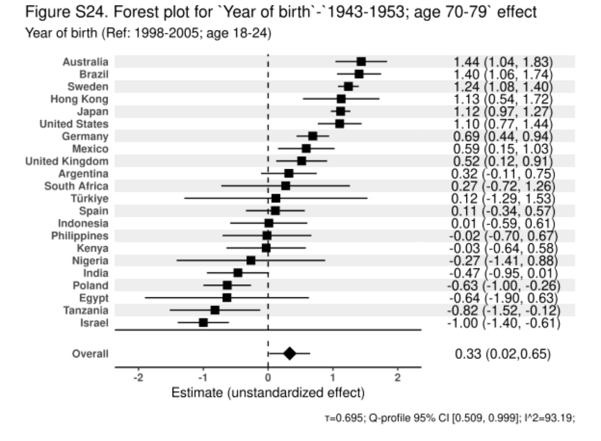

Happiness

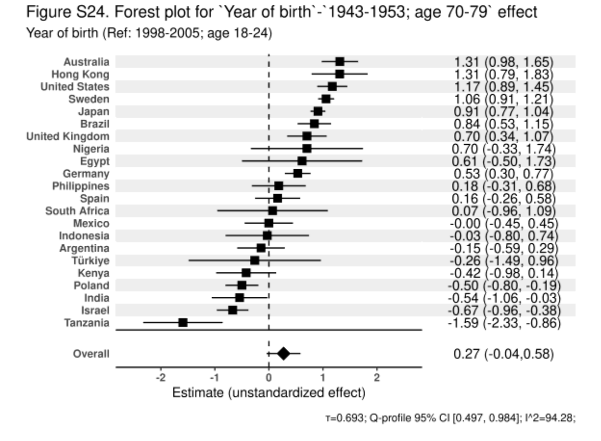

Cantril’s Ladder

Figure S25. Forest plot for `Year of birth`-`1943 or earlier; age 80+` effect

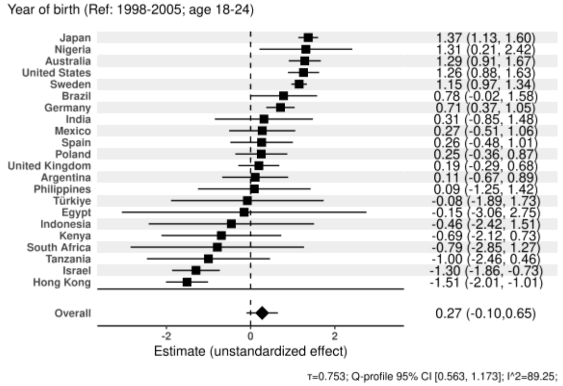

Life Satisfaction

Figure S25. Forest plot for `Year of birth`-`1943 or earlier; age 80+` effect

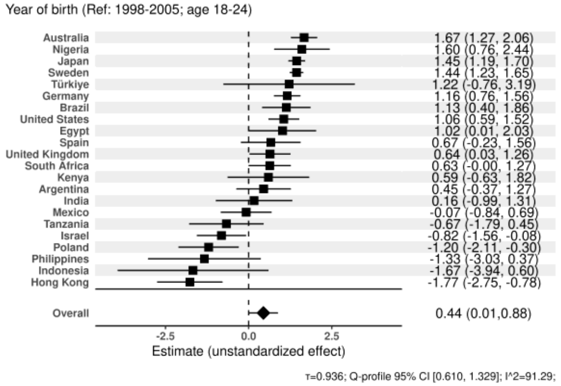

Happiness

Figure S25. Forest plot for `Year of birth`-`1943 or earlier; age 80+` effect

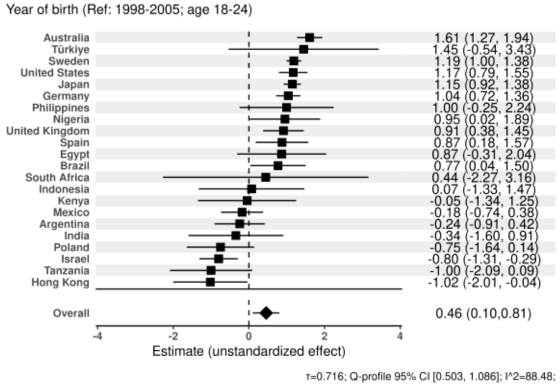

Cantril’s Ladder

Figure S26. Forest plot for `Gender`-`Female` effect

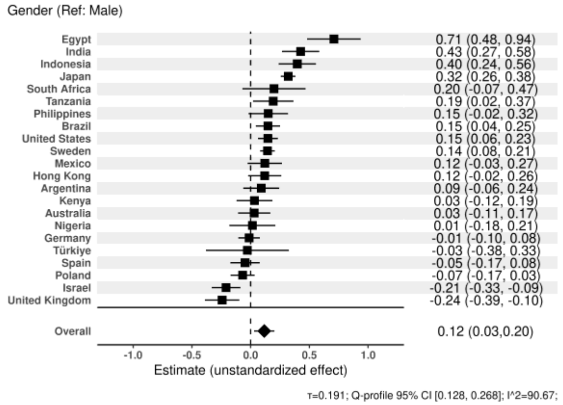

Life Satisfaction

Figure S26. Forest plot for `Gender`-`Female` effect

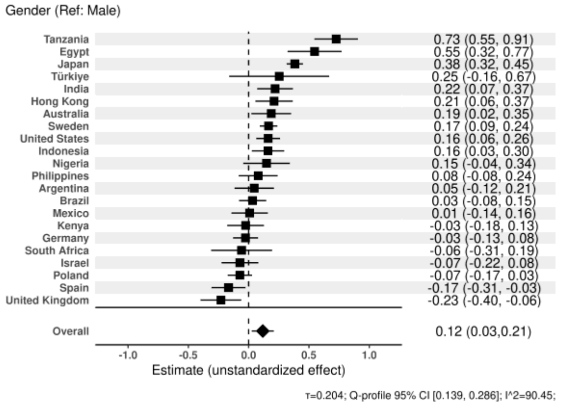

Happiness

Figure S26. Forest plot for `Gender`-`Female` effect

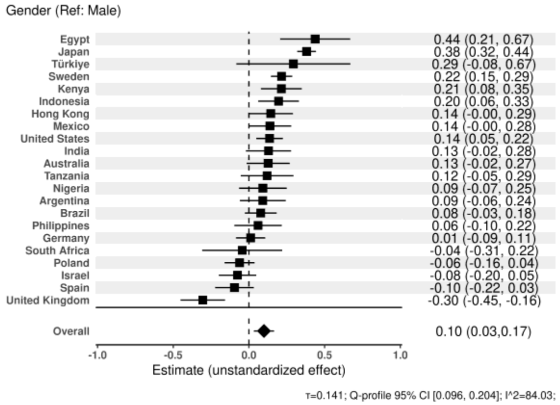

Cantril's Ladder

Figure S27. Forest plot for `Gender`-`Other` effect

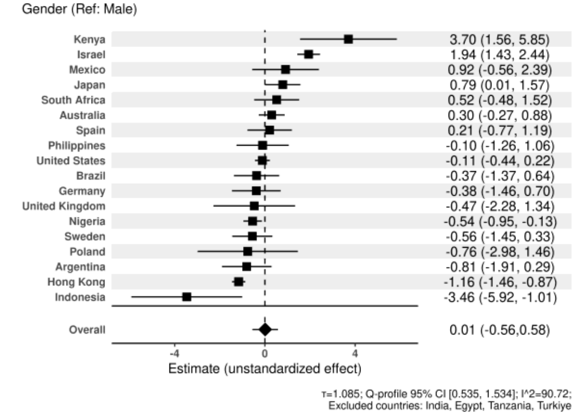

Life Satisfaction

Figure S27. Forest plot for `Gender`-`Other` effect

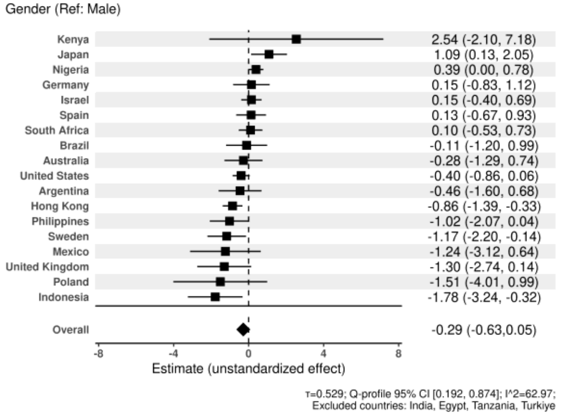

Happiness

Figure S27. Forest plot for `Gender`-`Other` effect

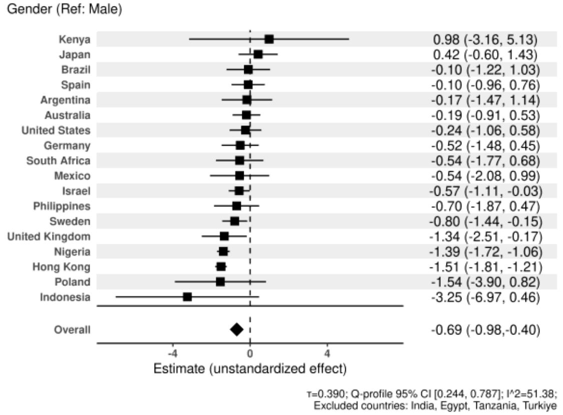

Figures 31a-c. Pairwise standardized mean differences in country means

Figure S31a. Pairwise standardized mean differences in country specific means on Present Cantril's Ladder

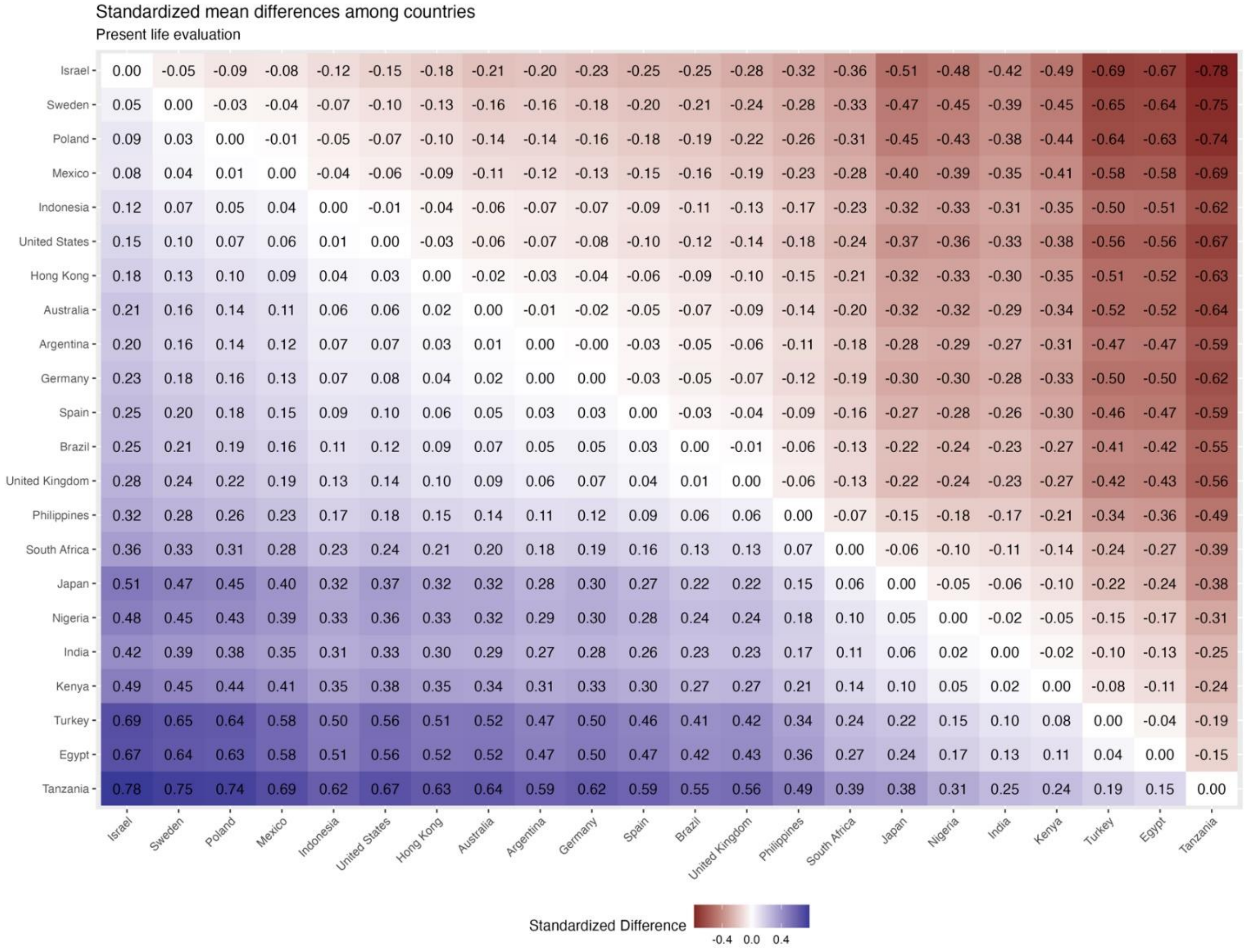

Figure S31b. Pairwise standardized mean differences in country specific means on Life Satisfaction

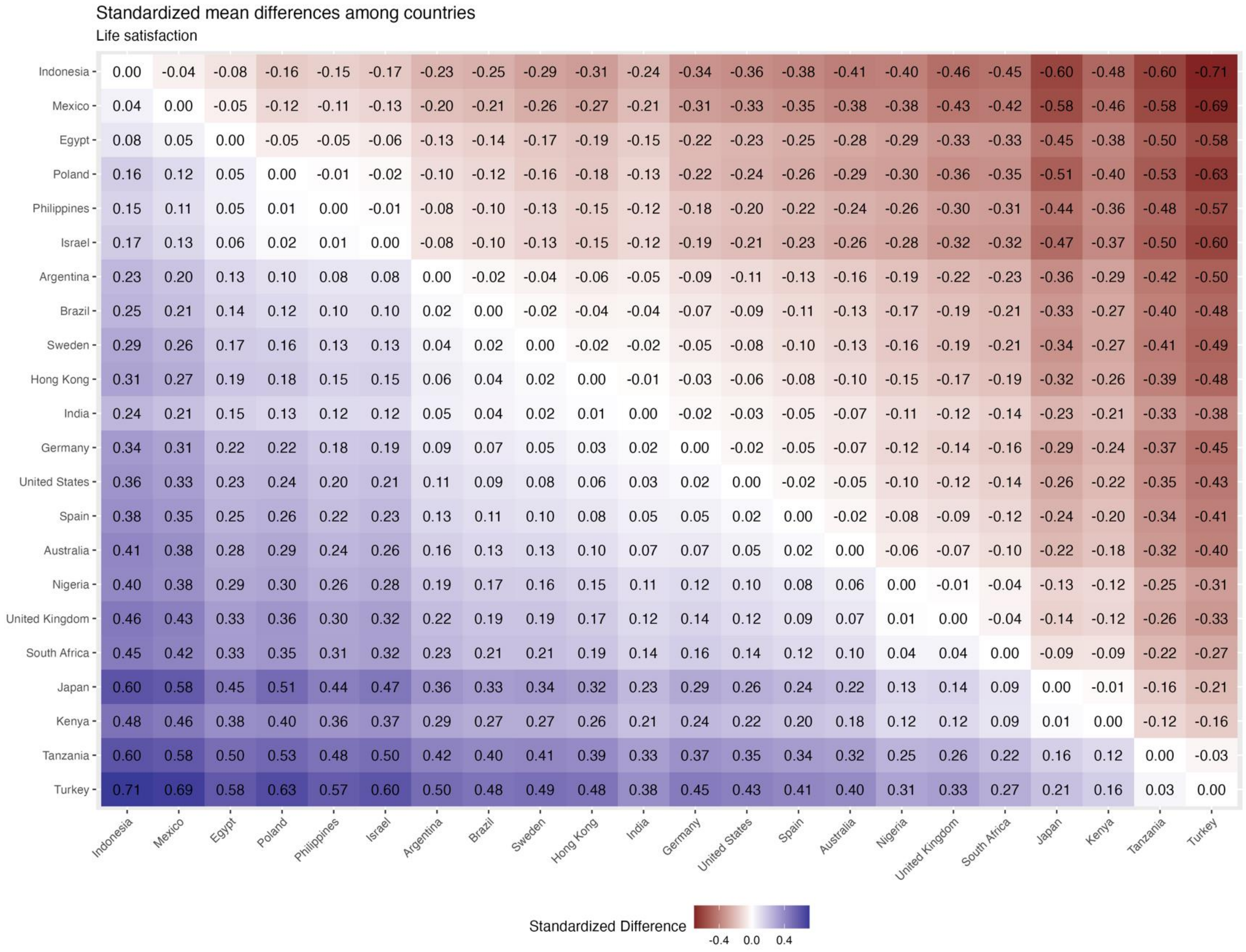

Figure S31c. Pairwise standardized mean differences in country specific means on Happiness

Standardized mean differences among countries

Happiness

	Indonesia	Mexico	Israel	Poland	Argentina	Brazil	Philippines	Kenya	Hong Kong	Nigeria	Sweden	United States	South Africa	Spain	Germany	Australia	United Kingdom	Tanzania	India	Japan	Egypt	Turkey
Indonesia	0.00	-0.08	-0.10	-0.18	-0.22	-0.22	-0.22	-0.21	-0.30	-0.29	-0.34	-0.35	-0.32	-0.38	-0.39	-0.41	-0.44	-0.37	-0.37	-0.60	-0.51	-0.68
Mexico	0.08	0.00	-0.01	-0.09	-0.15	-0.15	-0.15	-0.14	-0.22	-0.22	-0.27	-0.28	-0.25	-0.31	-0.32	-0.33	-0.37	-0.32	-0.32	-0.53	-0.45	-0.63
Israel	0.10	0.01	0.00	-0.09	-0.15	-0.15	-0.15	-0.14	-0.23	-0.23	-0.28	-0.30	-0.26	-0.32	-0.34	-0.36	-0.40	-0.32	-0.33	-0.57	-0.47	-0.66
Poland	0.18	0.09	0.09	0.00	-0.07	-0.08	-0.08	-0.08	-0.15	-0.16	-0.21	-0.22	-0.19	-0.25	-0.26	-0.28	-0.32	-0.27	-0.27	-0.50	-0.41	-0.60
Argentina	0.22	0.15	0.15	0.07	0.00	-0.01	-0.01	-0.02	-0.07	-0.09	-0.11	-0.12	-0.12	-0.15	-0.16	-0.17	-0.22	-0.20	-0.21	-0.38	-0.32	-0.50
Philippines	0.22	0.15	0.15	0.08	0.01	0.00	0.00	-0.02	-0.06	-0.08	-0.10	-0.11	-0.11	-0.13	-0.14	-0.15	-0.20	-0.19	-0.20	-0.35	-0.31	-0.48
Brazil	0.22	0.15	0.15	0.08	0.01	0.00	0.00	-0.02	-0.06	-0.08	-0.10	-0.11	-0.11	-0.13	-0.14	-0.15	-0.20	-0.19	-0.20	-0.35	-0.31	-0.48
Kenya	0.21	0.14	0.14	0.08	0.02	0.02	0.02	0.00	-0.03	-0.05	-0.07	-0.07	-0.08	-0.10	-0.11	-0.11	-0.15	-0.16	-0.17	-0.29	-0.26	-0.42
Hong Kong	0.30	0.22	0.23	0.15	0.07	0.06	0.06	0.03	0.00	-0.03	-0.05	-0.05	-0.06	-0.08	-0.09	-0.10	-0.16	-0.15	-0.17	-0.32	-0.28	-0.46
Nigeria	0.29	0.22	0.23	0.16	0.09	0.08	0.08	0.05	0.03	0.00	-0.01	-0.02	-0.03	-0.04	-0.05	-0.06	-0.11	-0.12	-0.13	-0.25	-0.22	-0.39
Sweden	0.34	0.27	0.28	0.21	0.11	0.10	0.10	0.07	0.05	0.01	0.00	-0.01	-0.02	-0.04	-0.05	-0.06	-0.11	-0.12	-0.14	-0.28	-0.24	-0.42
United States	0.35	0.28	0.30	0.22	0.12	0.11	0.11	0.07	0.05	0.02	0.01	0.00	-0.02	-0.03	-0.04	-0.05	-0.11	-0.11	-0.13	-0.28	-0.24	-0.42
South Africa	0.32	0.25	0.26	0.19	0.12	0.11	0.11	0.08	0.06	0.03	0.02	0.02	0.00	-0.01	-0.02	-0.02	-0.07	-0.09	-0.11	-0.21	-0.19	-0.36
Spain	0.38	0.31	0.32	0.25	0.15	0.13	0.13	0.10	0.08	0.04	0.04	0.03	0.01	0.00	-0.01	-0.01	-0.07	-0.09	-0.11	-0.24	-0.21	-0.39
Germany	0.39	0.32	0.34	0.26	0.16	0.14	0.14	0.11	0.09	0.05	0.05	0.04	0.02	0.01	0.00	-0.01	-0.07	-0.08	-0.10	-0.24	-0.21	-0.39
Australia	0.41	0.33	0.36	0.28	0.17	0.15	0.15	0.11	0.10	0.06	0.06	0.05	0.02	0.01	0.01	0.00	-0.06	-0.08	-0.10	-0.24	-0.20	-0.39
United Kingdom	0.44	0.37	0.40	0.32	0.22	0.20	0.20	0.15	0.16	0.11	0.11	0.11	0.07	0.07	0.07	0.06	0.00	-0.03	-0.06	-0.16	-0.15	-0.33
Tanzania	0.37	0.32	0.32	0.27	0.20	0.19	0.19	0.16	0.15	0.12	0.12	0.11	0.09	0.09	0.08	0.08	0.03	0.00	-0.02	-0.09	-0.09	-0.24
India	0.37	0.32	0.33	0.27	0.21	0.20	0.20	0.17	0.17	0.13	0.14	0.13	0.11	0.11	0.10	0.10	0.06	0.02	0.00	-0.06	-0.06	-0.20
Japan	0.60	0.53	0.57	0.50	0.38	0.35	0.35	0.29	0.32	0.25	0.28	0.28	0.21	0.24	0.24	0.24	0.16	0.09	0.06	0.00	-0.01	-0.19
Egypt	0.51	0.45	0.47	0.41	0.32	0.31	0.31	0.26	0.28	0.22	0.24	0.24	0.19	0.21	0.21	0.20	0.15	0.09	0.06	0.01	0.00	-0.15
Turkey	0.68	0.63	0.66	0.60	0.50	0.48	0.48	0.42	0.46	0.39	0.42	0.42	0.36	0.39	0.39	0.39	0.33	0.24	0.20	0.19	0.15	0.00

Standardized Difference: -0.4 0.0 0.4
